# Supplementary material for: One pot conversion of phenols and anilines to aldehydes and ketones exploiting α gem boryl carbanions
Source: Nat Commun. 2024 May 7;15:3794. doi: 10.1038/s41467-024-47156-0 (PMC11076505; doi:10.1038/s41467-024-47156-0)
Supplement: Supplementary file 1 — Supplementary Information [file 41467_2024_47156_MOESM1_ESM.pdf]

Supplementary information

One-pot Conversion of Phenols and Anilines to Aldehydes and Ketones Exploiting α-gem-Boryl Carbanions

Kanak Kanti Das, Debasis Aich, Sutapa Dey, Santanu Panda*

Indian Institute of Technology, Kharagpur, 721302, India

spanda@chem.iitkgp.ac.in

Table of Contents:

**1. General information**	**S2**
**2. Synthesis of starting materials**	**S2-S7**
**3. General procedures for the synthesis of aldehydes and ketones**	**S8-S10**
**3a. Synthesis of aldehydes**	**S11-S16**
**3b. Synthesis of ketones from different phenols**	**S17-S23**
**3c. Synthesis of ketones from different germinal B(pin)**	**S23-S25**
**3d. Synthesis of acetophenones**	**S25-S27**
**3e. Synthesis of α,β-unsaturated ketones**	**S27-S29**
**3f. Synthesis of aldehydes and ketones from anilines**	**S29-S33**
**4. Functionalization of bioactive molecules**	**S33-S46**
**5. Synthesis of bioactive molecules and regioselective acetophenone synthesis**	**S45-S47**
**6. Synthesis and application of photocatalyst**	**S47-S49**
**7. Crystallographic data**	**S50-S74**
**8. Boron-NMr study and UV-Vis spectroscopy**	**S74-S75**
**9. Steady-state emission spectroscopy**	**S76-S78**
**10. Fluorescence lifetime**	**S79-S80**
**11. Fluorescence quantum yield**	**S80-S81**
**12. Phosphorescence spectra and lifetime and details of 10b**	**S81-S82**
**13. Electrochemical measurement**	**S82-S84**
**14. HOMO-LUMO energy calculation and excited state potential calculation**	**S84**
**15. Computational method**	**S84-S91**

**16. Boron NMR**	**S91**
**17. Other results**	**S91-S93**
**18. NMR**	**8S94-S198**
**19. Supplementary References**	**S199-S200**

1(a). General Information:

General Methods: All the solvents were distilled prior to use. Dry solvents were prepared according to the standard procedures. All other reagents were used as received from either Aldrich or Lancaster chemical companies. Reactions requiring inert atmosphere were carried out under argon atmosphere. Infrared (IR) spectra were recorded on a JASCO 4100 FT-IR spectrometer. ^{1}H NMR spectra were measured on Bruker AVANCE 400 MHz and 500 MHz spectrometers. Chemical shifts were reported in ppm relative to solvent signals. ^{13}C NMR spectra were recorded on Bruker 100 MHz and 125 MHz spectrometers with complete proton decoupling. Chemical shifts were reported in ppm from the residual solvent as an internal standard. The high-resolution mass spectra (HRMS) were performed on Micromass QTOF micro mass spectrometer equipped with a Harvard apparatus syringe pump. X-ray crystallographic data were recorded using Bruker-AXS Kappa CCD-Diffractometer with graphite monochromator $Mo_{K\alpha}$ radiation (λ=0.7107 A). The structures were solved by direct methods (SHELXS-97) and refined by full-matrix least squares techniques against *F2* (SHELXL-97). Hydrogen atoms were inserted from geometry consideration using the HFIX option of the program. For thin layer chromatography (TLC) analysis throughout this work, E-merck precoated TLC plates (silica gel 60 F254 grade, 0.25 mm) were used. Acme (India) silica gel (100-200 mesh) was used for column chromatography.

For the experimental Set-up of this photo-catalytic reactions were set up in a light bath which is described below. Description of light: Blue Kessil LED, PR160L-427nm; S/N:L4M4G20258 KSPR160-427; 19V-40W; Taiwan. Here the reaction was set up on the table lamp stand, which is fixed on a Cardboard Rectangle Corrugated Paper Box. The reaction was set-up top of a magnetic stirrer. A lid which rest on the top was fashioned from cardboard and holes were made such that reaction tubes (18 x 150 mm, 27 ml borosilicate tube) were held firmly in the cardboard lid which was placed on the top of bath. All the reactions were performed at room temperature.

2. Synthesis of starting materials:

2(a). Synthesis of quinketals:

Here all the quinketals are synthesized from the reported procedure. The quinketals for the bioactive molecules have been synthesized from corresponding phenols which are synthesized from 4-hydroxy phenyl acetic acid by Mitsunobu reaction.

General Procedure (**GP-A**): To a oven dried reaction tube 4-hydroxy phenyl acetic acid (1 equiv, 0.5 mmol), corresponding alchole (1 equiv), PPh_3 (1 equiv) are taken and dry THF (3 mL/0.5 mmol of acid) was added. The reaction mixture was cooled to 0 ^oC and slowly DIAD (1 equiv) was added to it. Then the reaction mixture was allowed to stir at rt for 24 h. Next the reaction mixture was quenched by water. To the reaction mixture brine (10 mL) was then added and the aqueous layer was extracted with EtOAc (3×10 mL). The combined organic layers were dried with anhydrous Na_2SO_4 and the solvent was removed under reduced pressure. The resultant crude product material was purified by flash chromatography using 10% to 15% EtOAc/Hexane with the bellow mentioned yields and went for the next step.

General Procedure (**GP-B**): To a oven dried reaction tube the corresponding phenol (1 equiv, 0.5 mmol), was taken and dry MeOH (2 mL/0.5 mmol of acid) was added. The reaction mixture was cooled to 0 ^oC and slowly PIDA (1.1 equiv) was added to it. Then the reaction mixture was allowed to stir at rt for 15 min. The reaction mixture was cooled to 0 ^oC and to that 2.2 equivalent solid dry potassium carbonate was added. The reaction mixture was allowed to stir for 5 mints at the same temperature. Next the reaction was filtered on celite pad and the organic layer was concentrated. To the crude residue water was added and the aqueous layer was extracted with EtOAc (3×10 mL). Then the solvent was removed under reduced pressure. The resultant crude product material was purified by flash chromatography on nurtal alumina using 10% to 15% EtOAc/Hexane with the bellow mentioned yields and went for the next step.

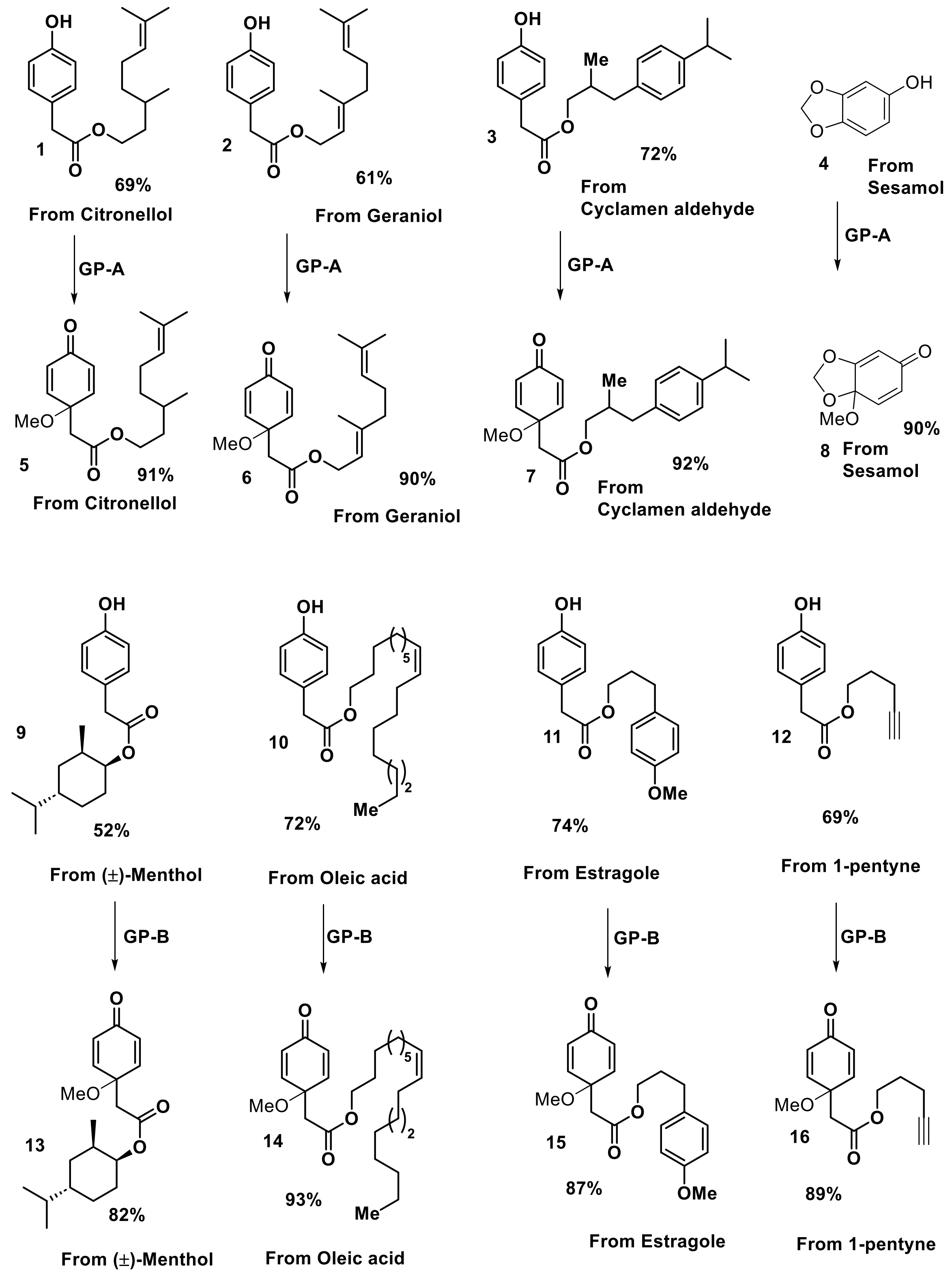

Supplementary Figure 1 : Synthesis of starting materials from phenols

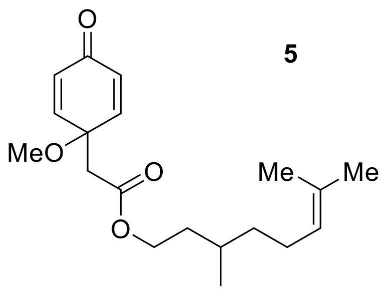

5

3,7-dimethyloct-6-en-1-yl 2-(1-methoxy-4-oxocyclohexa-2,5-dien-1-yl)acetate ($C_{19}H_{28}O_4$) (5): Synthesized using general procedure **GP-B** (with 0.5 mmol of corresponding phenol), purified by silica gel chromatography (10 to 15% EtOAc/hexane), colourless liquid, yield (145 mg) 91% from phenol (**1**). IR (Neat) cm^{-1} : 3053, 2972, 2927, 2856, 2830, 1738, 1670, 1640, 1610, 1455, 1380, 1272, 1236, 1160, 1100, 1079, 987, 860. ^{1}H NMR (400 MHz, $CDCl_3$) δ ppm 6.91 (d, *J* = 10.0 Hz, 2H), 6.37 (d, *J* = 9.8 Hz, 2H), 5.07 (s, 1H), 4.10 (s, 2H), 3.19 (s, 3H), 2.69 (s, 2H), 1.95 (brs, 2H), 1.67 (s, 3H), 1.59 (s, 3H), 1.44 – 1.35 (m, 3H), 1.31 (d, *J* = 6.2 Hz, 1H), 1.20 – 1.15 (m, 1H), 0.89 (d, *J* = 5.4 Hz, 3H). ^{13}C NMR (101 MHz, $CDCl_3$) δ ppm 185.02, 168.60, 149.22, 131.68, 131.51, 124.60, 72.92, 63.76, 53.12, 44.85, 37.03, 35.45, 29.52, 25.83, 25.47, 19.40, 17.76. $[M+H]^+$ calculated for $C_{19}H_{28}O_4$ is 321.2060 and found 321.2065.

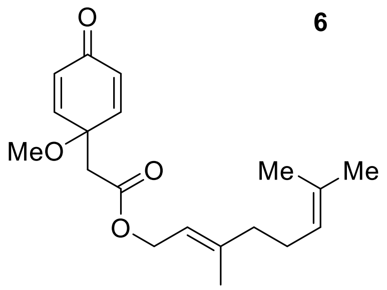

6

(*E*)-3,7-dimethylocta-2,6-dien-1-yl 2-(1-methoxy-4-oxocyclohexa-2,5-dien-1-yl)acetate ($C_{19}H_{26}O_4$) (6): Synthesized using general procedure **GP-B** (with 0.5 mmol of corresponding phenol), purified by silica gel chromatography (10 to 15% EtOAc/hexane), colourless liquid, yield (143 mg) 90% from phenol (**2**). IR (Neat) cm^{-1} : 3054, 2967, 2931, 2859, 2820, 1761, 1672, 1641, 1572, 1439, 1379, 1234, 1149, 1097, 1076, 955, 860. ^{1}H NMR (400 MHz, $CDCl_3$) δ ppm 6.87 (d, *J* = 9.5 Hz, 2H), 6.38 – 6.18 (m, 2H), 5.25 (t, *J* = 6.8 Hz, 1H), 5.02 (brs, 1H), 4.54 (d, *J* = 7.1 Hz, 2H), 3.14 (s, 3H), 2.66 (s, 2H), 2.04 – 1.98 (m, 4H), 1.62 (s, 3H), 1.62 (s, 3H), 1.54 (s, 3H). ^{13}C NMR (101 MHz, $CDCl_3$) δ ppm 184.82, 168.34, 149.04, 142.82, 131.79, 131.55, 123.69, 117.82, 72.84, 61.85, 52.96, 44.65, 39.48, 26.24, 25.67, 17.68, 16.45. $[M+H]^+$ calculated for $C_{19}H_{26}O_4$ is 319.1904 and found 319.1904.

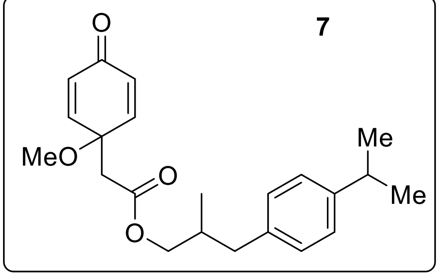

7

3-(4-isopropylphenyl)-2-methylpropyl 2-(1-methoxy-4-oxocyclohexa-2,5-dien-1-yl)acetate ($C_{22}H_{28}O_4$) (7): Synthesized using general procedure **GP-B** (with 0.5 mmol of corresponding phenol), purified by silica gel chromatography (10 to 15% EtOAc/hexane), colourless

liquid, yield (146 mg) 92% from phenol (**3**). IR (Neat) cm^{-1} : 3062, 2971, 2928, 2871, 2836, 1747, 1673, 1639, 1604, 1521, 1462, 1382, 1283, 1237, 1166, 1105, 1066, 1001, 931. ^{1}H NMR (400 MHz, $CDCl_3$) δ ppm 7.14 (d, *J* = 7.9 Hz, 3H), 7.07 (t, *J* = 7.0 Hz, 2H), 6.94 (d, *J* = 10.1 Hz, 2H), 6.39 (d, *J* = 10.1 Hz, 2H), 3.95 (qd, *J* = 10.8, 6.2 Hz, 2H), 3.20 (s, 3H), 2.88 (dd, *J* = 13.8, 6.9 Hz, 1H), 2.73 – 2.64 (m, 3H), 2.45 – 2.40 (m, 1H), 2.17 - 2.04 (m, 1H), 1.24 (s, 3H), 1.23 (s, 3H), 0.92 (dd, *J* = 6.7, 2.4 Hz, 3H). ^{13}C NMR (101 MHz, $CDCl_3$) δ ppm 185.03, 168.66, 149.25, 146.75, 146.54, 138.00, 137.19, 131.72, 129.17, 129.14, 126.49, 126.43, 72.90, 69.52, 67.90, 53.15, 44.86, 39.46, 37.94, 34.57, 33.82, 24.18, 16.88, 16.71. $[M+H]^+$ calculated for $C_{22}H_{28}O_4$ is 357.2060 and found 357.2061.

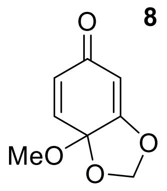

8

7a-methoxybenzo[*d*][1,3]dioxol-5(7a*H*)-one ($C_8H_8O_4$) (8): Synthesized using general procedure **GP-B** (with 0.5 mmol of corresponding phenol), purified by silica gel chromatography (10 to 15% EtOAc/hexane), colourless liquid, yield (76 mg) 90% from phenol (**4**). IR (Neat) cm^{-1} : 3070, 2985, 2921, 2848, 1679, 1657, 1610, 1472, 1413, 1343, 1297, 1195, 1084, 1025, 974, 916. ^{1}H NMR (400 MHz, $CDCl_3$) δ ppm 6.81 (d, *J* = 10.1 Hz, 1H), 6.15 (dd, *J* = 10.0, 1.4 Hz, 1H), 5.55 (d, *J* = 11.5 Hz, 2H), 5.47 (d, *J* = 1.4 Hz, 1H), 3.23 (s, 3H). ^{13}C NMR (101 MHz, $CDCl_3$) δ ppm 186.76, 168.11, 132.90, 132.44, 99.03, 98.77, 97.09, 51.29. $[M+H]^+$ calculated for $C_8H_8O_4$ is169.0495 and found 169.0498.

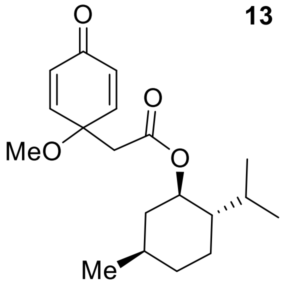

13

(1*R*,2*S*,5*R*)-2-isopropyl-5-methylcyclohexyl 2-(1-methoxy-4-oxocyclohexa-2,5-dien-1-yl)acetate ($C_{19}H_{28}O_4$) (13): Synthesized using general procedure **GP-B** (with 0.5 mmol of corresponding phenol), purified by silica gel chromatography (10 to 15% EtOAc/hexane), colourless liquid, yield (131 mg) 82% from phenol (**9**). IR (Neat) cm^{-1} : 3058, 2948, 2928, 2872, 2842, 1736, 1676, 1641, 1457, 1370, 1274, 1239, 1146, 1095, 1071, 964, 921. ^{1}H NMR (400 MHz, $CDCl_3$) δ ppm 6.90 (ddd, *J* = 7.7, 5.1, 3.0 Hz, 2H), 6.35 (d, *J* = 10.1 Hz, 2H), 5.18 (s, 1H), 3.15 (s, 3H), 2.67 (s, 2H), 1.86 (d, *J* = 14.2 Hz, 1H), 1.72 (d, *J* = 11.8 Hz, 2H), 1.55 (dd, *J* = 6.3, 3.2 Hz, 1H), 1.40 – 1.22 (m, 2H), 1.03 – 0.77 (m, 11H). ^{13}C NMR (101 MHz, $CDCl_3$) δ ppm 184.95, 168.13, 149.26, 149.21, 131.63, 131.60, 72.86,

72.35, 52.90, 46.70, 45.30, 39.04, 34.76, 29.15, 26.63, 25.28, 22.20, 21.05, 20.75. [M+H]$^+$ calculated for $C_{19}H_{28}O_4$ is 321.2060 and found 321.2064.

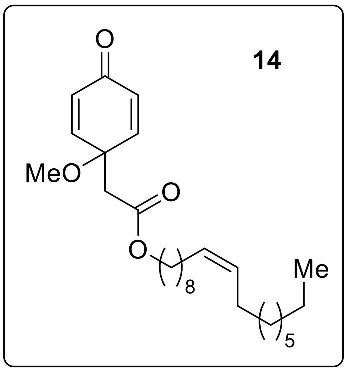

(1*R*,2*S*,5*R*)-2-isopropyl-5-methylcyclohexyl 2-(1-methoxy-4-oxocyclohexa-2,5-dien-1-yl)acetate ($C_{27}H_{44}O_4$) (14): Synthesized using general procedure **GP-B** (with 0.5 mmol of corresponding phenol), purified by silica gel chromatography (10 to 15% EtOAc/hexane), colourless liquid, yield (154 mg) 93% from phenol (**10**). IR (Neat) cm^{-1} : 3021, 2941, 2854, 1738, 1675, 1640, 1465, 1391, 1348, 1237, 1157, 1072, 860. ^{1}H NMR (400 MHz, $CDCl_3$) δ ppm 6.91 (d, *J* = 10.1 Hz, 2H), 6.37 (d, *J* = 10.1 Hz, 2H), 5.44 – 5.23 (m, 2H), 4.06 (t, *J* = 6.7 Hz, 2H), 3.19 (s, 3H), 2.69 (s, 2H), 2.00 (d, *J* = 5.7 Hz, 3H), 1.64 – 1.54 (m, 2H), 1.28 – 1.25 (m, 23H), 0.86 (t, *J* = 6.4 Hz, 3H). ^{13}C NMR (101 MHz, $CDCl_3$) δ ppm 185.00, 168.59, 149.20, 131.68, 130.11, 129.87, 72.91, 65.35, 53.11, 44.85, 32.02, 29.88, 29.84, 29.63, 29.53, 29.43, 29.31, 29.29, 28.62, 27.33, 27.29, 25.97, 22.80, 14.23. [M+H]$^+$ calculated for $C_{27}H_{44}O_4$ is 433.3319 and found 433.3312.

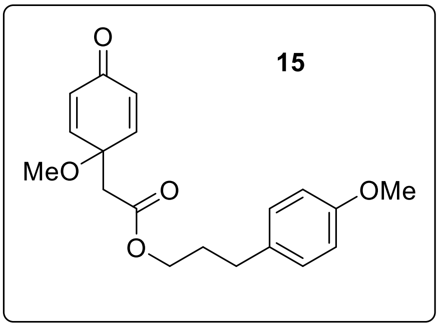

3-(4-methoxyphenyl)propyl 2-(1-methoxy-4-oxocyclohexa-2,5-dien-1-yl)acetate ($C_{19}H_{22}O_5$) (15): Synthesized using general procedure **GP-B** (with 0.5 mmol of corresponding phenol), purified by silica gel chromatography (10 to 15% EtOAc/hexane), colourless liquid, yield (143 mg) 87% from phenol (**11**). IR (Neat) cm^{-1} : 3072, 3063, 2983, 2938, 2836, 1732, 1683, 1613, 1513, 1466, 1376, 1246, 1179, 1106, 1035, 930. ^{1}H NMR (400 MHz, $CDCl_3$) δ 7.43 – 7.41 (m, 2H), 7.28 – 7.26 (m, 2H), 7.17 (brs, 2H), 6.74 – 6.71 (m, 2H), 4.42 (brs, 2H), 4.12 (s, 3H), 3.54 (s, 3H), 3.04 (s, 2H), 2.94 (brs, 2H), 2.24 – 2.23 (m, 2H). ^{13}C NMR (101 MHz, $CDCl_3$) δ ppm 184.87, 168.44, 157.90, 149.11, 132.99, 131.53, 129.25, 113.85, 72.77, 64.37, 55.21, 52.99, 44.62, 31.11, 30.27. [M+H]$^+$ calculated for $C_{19}H_{22}O_5$ is 331.1540 and found 331.1546.

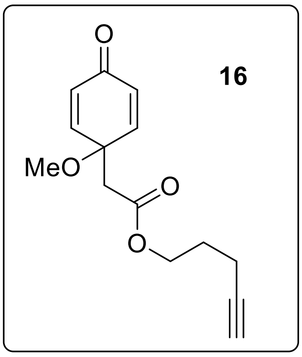

Pent-4-yn-1-yl 2-(1-methoxy-4-oxocyclohexa-2,5-dien-1-yl)acetate ($C_{14}H_{16}O_4$) (16): Synthesized using general procedure **GP-B** (with 0.5 mmol of corresponding phenol), purified by silica

gel chromatography (10 to 15% EtOAc/hexane), colourless liquid, yield (110 mg) 89% from phenol (**12**). IR (Neat) cm^{-1} : 3287, 3040, 2983, 2939, 2831, 2235, 1734, 1647, 1640, 1601, 1524, 1466, 1386, 1280, 1239, 1158, 1095, 1079, 1036, 920. ^{1}H NMR (400 MHz, $CDCl_3$) δ ppm 6.88 (d, *J* = 10.0 Hz, 2H), 6.34 (d, *J* = 9.9 Hz, 2H), 4.16 – 4.14 (m, 2H), 3.16 (s, 3H), 2.67 (s, 2H), 2.23 (d, *J* = 4.1 Hz, 2H), 1.94 (s, 1H), 1.81 – 1.78 (m, 2H). ^{13}C NMR (101 MHz, $CDCl_3$) δ ppm 184.96, 168.38, 149.04, 131.63, 82.80, 72.79, 69.27, 63.53, 53.05, 44.66, 27.36, 15.12. $[M+H]^+$ calculated for $C_{14}H_{16}O_4$ is 249.1121 and found 249.1124.

3. Synthesis of aldehydes and ketones from phenols:

Here all the phenols are purchased from BLD Chemicals. Dry MeOH used for the PIDA oxidation reaction. Dry potassium carbonate engaged for the acetic acid quenching.

General Procedure (**GP-C**): To a flame-dried reaction tube the corresponding phenol (1 equiv, 0.5 mmol), was taken and dry MeOH (1 mL/0.5 mmol of phenol) was added. The reaction mixture was cooled to 0 ^{o}C and slowly PIDA (1.1 equiv) was added to it. Then the reaction mixture was allowed to stir at rt for 15 min. The reaction mixture was again cooled to 0 ^{o}C and to that 2.2 equivalent solid dry potassium carbonate was added. The reaction mixture was allowed to stir for 5 minutes at the same temperature. Then the MeOH was removed in a vacuum and it was azeotropically dried by dry toluene. Next dry THF was added (2 mL) and cooled to 0 ^{o}C. Further, 1.5 equiv of pre-prepared lithiated germinal B(pin) (pre-cooled at 0 ^{o}C) was added to it. The reaction mixture was allowed to stir for 5 minutes at the same temperature. Next 3 equiv of solid $NaBO_3.4H_2O$ followed by 2 mL of water were added to it. The reaction mixture was allowed to stir at rt for 5 minutes. Next EA was added and the aqueous layer was extracted with EtOAc (3×10 mL). Then the solvent was removed under reduced pressure. The resultant crude product was purified by flash chromatography on silica using 10% to 15% EtOAc/Hexane.

General Procedure (**GP-D**): To a flame-dried reaction tube the corresponding phenol (1 equiv, 0.5 mmol), was taken and dry MeOH (1 mL/0.5 mmol of phenol) was added. The reaction mixture was cooled to 0 ^{o}C and slowly PIDA (1.1 equiv) was added to it. Then the reaction mixture was allowed to stir at rt for 15 min. The reaction mixture was again cooled to 0 ^{o}C and to that 2.2 equivalent solid dry potassium carbonate was added. The reaction mixture was allowed to stir for 5 minutes at the same temperature. Then the MeOH was

removed in a vacuum and it was azeotropically dried by dry toluene. Next dry THF was added (2 mL) and cooled to 0 ^{o}C. Further, 1.5 equiv of pre-prepared lithiated germinal B(pin) (pre-cooled at 0 ^{o}C) was added to it. The reaction mixture was allowed to stir for 5 minutes at the same temperature. Next 3 equiv of solid $NaBO_3.4H_2O$ followed by 2 mL of water were added to it. The reaction mixture was allowed to stir at rt for 15 minutes. Next EA was added and the aqueous layer was extracted with EtOAc (3×10 mL). Then the solvent was removed under reduced pressure. The resultant crude product was purified by flash chromatography on silica using 10% to 15% EtOAc/Hexane.

General Procedure (**GP-E**): To a flame-dried reaction tube the corresponding phenol (1 equiv, 0.5 mmol), was taken and dry MeOH (1 mL/0.5 mmol of phenol) was added. The reaction mixture was cooled to 0 ^{o}C and slowly PIDA (1.1 equiv) was added to it. Then the reaction mixture was allowed to stir at rt for 15 min. The reaction mixture was again cooled to 0 ^{o}C and to that 2.2 equivalent solid dry potassium carbonate was added. The reaction mixture was allowed to stir for 5 minutes at the same temperature. Then the MeOH was removed in a vacuum and it was azeotropically dried by dry toluene. Next dry THF was added (2 mL) and cooled to 0 ^{o}C. Further, 1.5 equiv of pre-prepared lithiated germinal B(pin) (pre-cooled at 0 ^{o}C) was added to it. The reaction mixture was allowed to stir for 5 minutes at the same temperature. Next 3 equiv of solid $NaBO_3.4H_2O$ followed by 2 mL of water were added to it. The reaction mixture was allowed to stir at rt for 10 minutes. Next EA was added and the aqueous layer was extracted with EtOAc (3×10 mL). Then the solvent was removed under reduced pressure. The resultant crude product was purified by flash chromatography on silica using 10% to 15% EtOAc/Hexane.

General Procedure (**GP-F**): To a flame-dried reaction tube the corresponding phenol (1 equiv, 0.5 mmol), was taken and dry MeOH (1 mL/0.5 mmol of phenol) was added. The reaction mixture was cooled to 0 ^{o}C and slowly PIDA (1.1 equiv) was added to it. Then the reaction mixture was allowed to stir at rt for 15 min. The reaction mixture was again cooled to 0 ^{o}C and to that 2.2 equivalent solid dry potassium carbonate was added. The reaction mixture was allowed to stir for 5 minutes at the same temperature. Then the MeOH was removed in a vacuum and it was azeotropically dried by dry toluene. Next dry THF was added (2 mL) and cooled to -78 ^{o}C. Further, 1.0 equiv of pre-prepared lithiated germinal B(pin) (pre-cooled at 0 ^{o}C) was added to it. The reaction mixture was allowed to stir for 5 minutes at room temperature. Next 3 equiv of solid $NaBO_3.4H_2O$ followed by 2 mL of water were added to it at 0 ^{o}C. The reaction mixture was allowed to stir at rt for 5 minutes. Next EA

was added and the aqueous layer was extracted with EtOAc (3×10 mL). Then the solvent was removed under reduced pressure. The resultant crude product was purified by flash chromatography on silica using 10% to 15% EtOAc/Hexane.

General Procedure (**GP-G**): To a flame-dried reaction tube the corresponding phenol (1 equiv, 0.5 mmol), was taken and dry MeOH (1 mL/0.5 mmol of phenol) was added. The reaction mixture was cooled to 0 ^{o}C and slowly PIDA (1.1 equiv) was added to it. Then the reaction mixture was allowed to stir at rt for 15 min. The reaction mixture was again cooled to 0 ^{o}C and to that 2.2 equivalent solid dry potassium carbonate was added. The reaction mixture was allowed to stir for 5 minutes at the same temperature. Then the MeOH was removed in a vacuum and it was azeotropically dried by dry toluene. Next dry THF was added (2 mL) and cooled to -78 ^{o}C. Further, 1.0 equiv of pre-prepared lithiated germinal B(pin) (pre-cooled at 0 ^{o}C) was added to it. The reaction mixture was allowed to stir for 5 minutes at room temperature. Next 3 equiv of solid $NaBO_3.4H_2O$ followed by 2 mL of water were added to it at 0 ^{o}C. The reaction mixture was allowed to stir at rt for 15 minutes. Next EA was added and the aqueous layer was extracted with EtOAc (3×10 mL). Then the solvent was removed under reduced pressure. The resultant crude product was purified by flash chromatography on silica using 10% to 15% EtOAc/Hexane.

General Procedure (**GP-H**): To a flame-dried reaction tube the corresponding acetophenone (1 equiv, 0.1 mmol), was taken and dry THF (1 mL/0.1 mmol of acetophenone) was added. The reaction mixture was cooled to 0 ^{o}C and slowly NaOEt (2 equiv) was added to it. Then the reaction mixture was allowed to stir at rt for 15 min. The reaction mixture was again cooled to 0 ^{o}C and to that 2.2 equivalent diethyl oxalate (in 1 mL dry THF) was added slowly. The reaction mixture was allowed to stir for 10 hours at the room temperature. Then the reaction mixture was quenched with dilute hydrochloric acid (to make pH ~ 6) followed by 2 mL of water were added to it. Next EA was added and the aqueous layer was extracted with EtOAc (3×10 mL). Then the solvent was removed under reduced pressure. The resultant crude product was purified by flash chromatography on silica using 20% to 25% EtOAc/Hexane.

3(a). Synthesis of aldehydes:

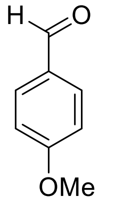

2a

4-methoxybenzaldehyde ($C_8H_8O_2$) (2a): **ref-1** Synthesized using general procedure **GP-C** (with 0.2 mmol of corresponding phenol), purified by silica gel chromatography (10 to 15% EtOAc/hexane), colourless liquid, yield (24 mg) 88% from corresponding phenol. We have also performed the large scale synthesis of **2a** starting from 8 mmol of 4-methoxy phenol, which ended up with 79% of **2a**. ^{1}H NMR (400 MHz, $CDCl_3$) δ ppm 9.80 (s, 1H), 7.75 (d, *J* = 8.6 Hz, 2H), 6.92 (d, *J* = 8.7 Hz, 2H), 3.79 (s, 3H). ^{13}C NMR (126 MHz, $CDCl_3$) δ ppm 189.73, 163.58, 130.94, 128.97, 113.32, 54.64.

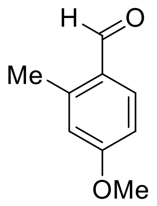

2b

4-methoxy-2-methylbenzaldehyde ($C_9H_{10}O_2$) (2b): **ref-1** Synthesized using general procedure **GP-C** (with 0.2 mmol of corresponding phenol), purified by silica gel chromatography (10 to 15% EtOAc/hexane), colourless liquid, yield (20 mg) 68% from corresponding phenol. We have also synthesized it from its corresponding quinketal which resulted the desire product with 83% isolated yield calculated from the quinketal. ^{1}H NMR (400 MHz, $CDCl_3$) δ ppm 10.11 (s, 1H), 7.75 (d, *J* = 8.4 Hz, 1H), 6.83 (d, *J* = 8.4 Hz, 1H), 6.74 (s, 1H), 3.86 (s, 3H), 2.65 (s, 3H). ^{13}C NMR (101 MHz, $CDCl_3$) δ ppm 191.33, 163.77, 143.43, 134.86, 128.07, 117.12, 111.62, 55.57, 20.03.

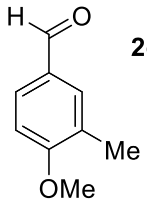

2c

4-methoxy-3-methylbenzaldehyde ($C_9H_{10}O_2$) (2c): **ref-1** Synthesized using general procedure **GP-C** (with 0.2 mmol of corresponding phenol), purified by silica gel chromatography (10 to 15% EtOAc/hexane), colourless liquid, yield (24 mg) 79% from corresponding phenol. ^{1}H NMR (400 MHz, $CDCl_3$) δ ppm 9.85 (s, 1H), 7.75 – 7.70 (m, 1H), 7.68 (brs, 1H), 6.93 (d, *J* = 8.3 Hz, 1H), 3.92 (s, 3H), 2.26 (s, 3H). ^{13}C NMR (101 MHz, $CDCl_3$) δ ppm 191.18, 162.88, 131.42, 130.69, 129.41, 127.59, 109.62, 55.64, 16.18.

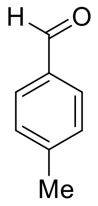
2d

4-methylbenzaldehyde (C_8H_8O) (2d): ref-1 Synthesized using general procedure **GP-C** (with 0.2 mmol of corresponding phenol), purified by silica gel chromatography (10 to 15% EtOAc/hexane), colourless liquid, yield (20 mg) 84% from corresponding phenol. ^{1}H NMR (400 MHz, $CDCl_3$) δ ppm 9.95 (s, 1H), 7.76 (d, *J* = 7.9 Hz, 2H), 7.32 (d, *J* = 7.8 Hz, 2H), 2.42 (s, 3H). ^{13}C NMR (101 MHz, $CDCl_3$) δ ppm 192.13, 145.66, 134.26, 129.94, 129.80, 21.97.

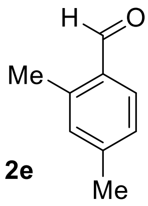
2e

2,4-dimethylbenzaldehyde ($C_9H_{10}O$) (2e): ref-1 Synthesized using general procedure **GP-C** (with 0.2 mmol of corresponding phenol), purified by silica gel chromatography (10 to 15% EtOAc/hexane), colourless liquid, yield (16 mg) 60% from corresponding phenol. We have also synthesized it from its corresponding quinketal which resulted the desire product with 81% isolated yield calculated from the quinketal. ^{1}H NMR (400 MHz, $CDCl_3$) δ ppm 10.20 (s, 1H), 7.68 (d, *J* = 7.6 Hz, 1H), 7.16 (d, *J* = 7.6 Hz, 1H), 7.06 (s, 1H), 2.63 (s, 3H), 2.38 (s, 3H). ^{13}C NMR (126 MHz, $CDCl_3$) δ ppm 192.47, 144.71, 140.78, 132.70, 132.53, 127.23, 109.84, 21.79, 19.62.

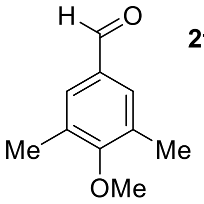
2f

4-methoxy-3,5-dimethylbenzaldehyde ($C_{10}H_{12}O_2$) (2f): ref-1 Synthesized using general procedure **GP-C** (with 0.2 mmol of corresponding phenol), purified by silica gel chromatography (10 to 15% EtOAc/hexane), colourless liquid, yield (25 mg) 76% from corresponding phenol. ^{1}H NMR (400 MHz, $CDCl_3$) δ ppm 9.87 (s, 1H), 7.56 (s, 2H), 3.78 (s, 3H), 2.35 (s, 6H). ^{13}C NMR (126 MHz, $CDCl_3$) δ ppm 191.71, 162.60, 132.49, 132.08, 130.86, 59.85, 16.30.

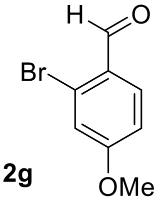
2g

2-bromo-4-methoxybenzaldehyde ($C_8H_7BrO_2$) (2g): ref-2 Synthesized using general procedure **GP-C** (with 0.2 mmol of corresponding quinketal), purified by silica gel chromatography (10 to 15% EtOAc/hexane), colourless liquid, yield (26 mg) 61% from corresponding quinketal. ^{1}H NMR (500 MHz, $CDCl_3$) δ ppm 10.22 (s, 1H), 7.89 (d, J = 8.7 Hz, 1H), 7.14 (d, J = 2.3 Hz, 1H), 6.94 (dd, J = 8.7, 1.7 Hz, 1H), 3.88 (s, 3H). ^{13}C NMR (126 MHz, $CDCl_3$) δ ppm 190.65, 164.73, 131.55, 128.83, 127.31, 118.75, 114.32, 56.04.

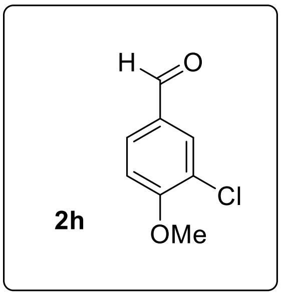

3-chloro-4-methoxybenzaldehyde ($C_8H_7ClO_2$) (2h): [ref-2] Synthesized using general procedure **GP-C** (with 0.2 mmol of corresponding quinketal), purified by silica gel chromatography (10 to 15% EtOAc/hexane), colourless liquid, yield (24 mg) 72% from corresponding quinketal. ^{1}H NMR (500 MHz, $CDCl_3$) δ ppm 9.85 (s, 1H), 7.91 (d, J = 1.2 Hz, 1H), 7.77 (d, J = 7.5 Hz, 1H), 7.04 (d, J = 8.4 Hz, 1H), 3.99 (s, 3H).^{13}C NMR (126 MHz, $CDCl_3$) δ ppm 189.67, 159.98, 131.34, 130.53, 126.49, 123.89, 111.82, 56.56.

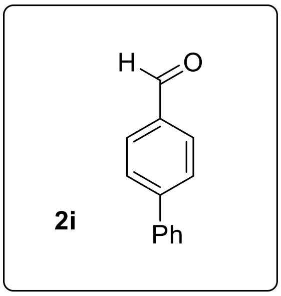

[1,1'-biphenyl]-4-carbaldehyde ($C_{13}H_{10}O$) (2i): [ref-2] Synthesized using general procedure **GP-C** (with 0.2 mmol of corresponding phenol), purified by silica gel chromatography (10 to 15% EtOAc/hexane), colourless liquid, yield (27 mg) 74% from corresponding phenol. ^{1}H NMR (400 MHz, $CDCl_3$) δ ppm 10.06 (d, J = 2.4 Hz, 1H), 7.96 (d, J = 8.2 Hz, 2H), 7.76 (d, J = 8.2 Hz, 2H), 7.64 (d, J = 7.6 Hz, 2H), 7.50 – 7.47 (m, 2H), 7.44 – 7.40 (m, 1H). ^{13}C NMR (101 MHz, $CDCl_3$) δ ppm 192.00, 147.27, 139.79, 135.28, 130.35, 129.10, 128.56, 127.76, 127.45.

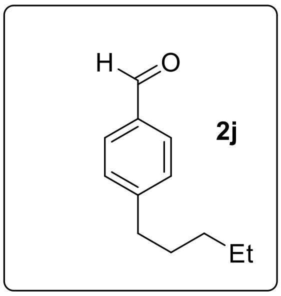

4-pentylbenzaldehyde ($C_{12}H_{16}O$) (2j): [ref-2] Synthesized using general procedure **GP-C** (with 0.2 mmol of corresponding phenol), purified by silica gel chromatography (10 to 15% EtOAc/hexane), colourless liquid, yield (28 mg) 79% from corresponding phenol. ^{1}H NMR (400 MHz, $CDCl_3$) δ ppm 9.97 (s, 1H), 7.79 (d, J = 8.0 Hz, 2H), 7.33 (d, J = 7.5 Hz, 2H), 2.68 (t, J = 7.7 Hz, 2H), 1.66 – 1.60 (m, 2H), 1.33 – 1.32 (m, 4H), 0.90 – 0.87 (m, 3H). ^{13}C NMR (101 MHz, $CDCl_3$) δ ppm 192.18, 150.63, 134.49, 130.01, 129.20, 36.30, 31.54, 30.89, 22.60, 14.11.

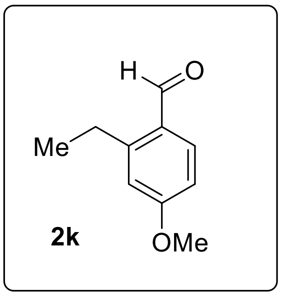

2-ethyl-4-methoxybenzaldehyde ($C_{10}H_{12}O_2$) (2k): [ref-2] Synthesized using general procedure **GP-C** (with 0.2 mmol of corresponding phenol), purified by silica gel chromatography (10 to 15% EtOAc/hexane), colourless liquid, yield (24 mg) 73% from corresponding phenol. ^{1}H NMR (400 MHz, $CDCl_3$) δ ppm 6.80 –

6.77 (m, 1H), 6.53 (d, *J* = 1.5 Hz, 1H), 6.26 (d, *J* = 10.3 Hz, 1H), 3.37 (s, 3H), 2.33 (q, *J* = 7.3 Hz, 2H), 1.08 (t, *J* = 7.3 Hz, 3H). ^{13}C NMR (101 MHz, $CDCl_3$) δ ppm 185.58, 142.75, 142.39, 137.02, 130.45, 93.24, 50.47, 22.07, 12.30.

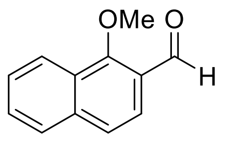

2l

1-methoxy-2-naphthaldehyde ($C_{12}H_{10}O_2$) (2l): [ref-2] Synthesized using general procedure **GP-C** (with 0.2 mmol of corresponding phenol), purified by silica gel chromatography (10 to 15% EtOAc/hexane), colourless liquid, yield (24 mg) 65% from corresponding phenol. ^{1}H NMR (400 MHz, $CDCl_3$) δ ppm 10.60 (s, 1H), 8.26 (d, *J* = 7.8 Hz, 1H), 7.88 – 7.85 (m, 2H), 7.66 – 7.57 (m, 3H), 4.15 (s, 3H). ^{13}C NMR (101 MHz, $CDCl_3$) δ ppm 189.85, 162.76, 138.27, 129.53, 128.55, 127.97, 126.98, 124.99, 124.86, 123.32, 122.82, 65.84.

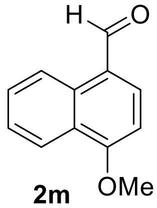

2m

4-methoxy-1-naphthaldehyde ($C_{12}H_{10}O_2$) (2m): [ref-3] Synthesized using general procedure **GP-C** (with 0.2 mmol of corresponding phenol), purified by silica gel chromatography (10 to 15% EtOAc/hexane), colourless liquid, yield (23 mg) 61% from corresponding phenol. ^{1}H NMR (400 MHz, $CDCl_3$) δ ppm 10.19 (s, 1H), 9.30 (d, *J* = 8.5 Hz, 1H), 8.33 (d, *J* = 8.4 Hz, 1H), 7.91 (d, *J* = 8.1 Hz, 1H), 7.69 (dd, *J* = 11.6, 4.1 Hz, 1H), 7.56 (d, *J* = 7.7 Hz, 1H), 6.90 (d, *J* = 8.1 Hz, 1H), 4.09 (s, 3H). ^{13}C NMR (126 MHz, $CDCl_3$) δ ppm 192.34, 161.03, 139.61, 132.09, 129.64, 126.52, 125.72, 125.23, 124.98, 122.52, 103.06, 56.10.

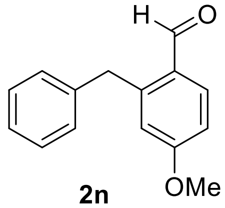

2n

2-benzyl-4-methoxybenzaldehyde ($C_{15}H_{14}O_2$) (2n): [ref-4] Synthesized using general procedure **GP-C** (with 0.2 mmol of corresponding phenol), purified by silica gel chromatography (10 to 15% EtOAc/hexane), colourless liquid, yield (34 mg) 76% from corresponding phenol. ^{1}H NMR (400 MHz, $CDCl_3$) δ ppm 10.15 (s, 1H), 7.87 (d, *J* = 8.5 Hz, 1H), 7.34 – 7.30 (m, 2H), 7.25 – 7.18 (m, 3H), 6.94 (d, *J* = 7.4 Hz, 1H), 6.78 (s, 1H), 4.47 (s, 2H), 3.88 (s, 3H). ^{13}C NMR (126 MHz, $CDCl_3$) δ ppm 190.69, 163.81, 145.52, 139.94, 134.81, 128.71, 128.47, 127.56, 126.22, 117.13, 111.74, 55.36, 38.08.

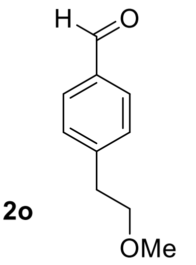

2o

4-(2-methoxyethyl)benzaldehyde ($C_{10}H_{12}O_2$) (2o): [ref-4] Synthesized using general procedure **GP-C** (with 0.2 mmol of corresponding phenol), purified by silica gel chromatography (10 to 15% EtOAc/hexane), colourless liquid, yield (24 mg) 73% from corresponding phenol. ^{1}H NMR (500 MHz, $CDCl_3$) δ ppm 9.97 (s, 1H), 7.80 (d, J = 8.0 Hz, 2H), 7.38 (d, J = 8.0 Hz, 2H), 3.63 (t, J = 6.7 Hz, 2H), 3.34 (s, 3H), 2.95 (t, J = 6.7 Hz, 2H). ^{13}C NMR (126 MHz, $CDCl_3$) δ ppm 191.95, 146.75, 135.01, 129.96, 129.64, 72.86, 58.79, 36.51.

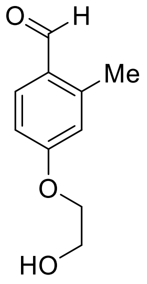

2p

4-(2-hydroxyethoxy)-2-methylbenzaldehyde ($C_{10}H_{12}O_3$) (2p): Synthesized using general procedure **GP-C** (with 0.2 mmol of corresponding phenol), purified by silica gel chromatography (10 to 15% EtOAc/hexane), colourless liquid, yield (22 mg) 60% from corresponding phenol. IR (Neat) cm^{-1} : 3402, 3066, 3052, 2960, 2924, 2853, 2741, 1726, 1681, 1601, 1562, 1499, 1454, 1380, 1299, 1256, 1126, 1051, 965. ^{1}H NMR (500 MHz, $CDCl_3$) δ ppm 10.12 (s, 1H), 7.75 (d, J = 8.5 Hz, 1H), 6.86 (dd, J = 8.4, 1.5 Hz, 1H), 6.77 (brs, 1H), 4.17 – 4.13 (m, 2H), 4.00 (brs, 3H), 2.65 (s, 3H). ^{13}C NMR (126 MHz, $CDCl_3$) δ ppm 191.31, 162.97, 143.61, 134.95, 128.62, 117.82, 112.30, 69.68, 61.51, 20.02. $[M+H]^+$ calculated for $C_{10}H_{12}O_3$ is 181.0859 and found 181.0861.

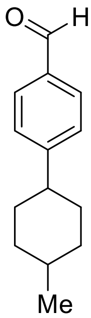

2q

4-(4-methylcyclohexyl)benzaldehyde ($C_{14}H_{18}O$) (2q): Synthesized using general procedure **GP-C** (with 0.2 mmol of corresponding phenol), purified by silica gel chromatography (10 to 15% EtOAc/hexane), colourless liquid, yield (28 mg) 70% from corresponding phenol. IR (Neat) cm^{-1} : 3045, 2922, 2849, 2723, 1708, 1607, 1570, 1514, 1447, 1305, 1212, 1169, 1078, 1016, 969, 920. ^{1}H NMR (400 MHz, $CDCl_3$) δ ppm 9.96 (s, 1H), 7.81 (brs, 2H), 7.36 (d, J = 4.8 Hz, 2H), 2.60 – 2.50 (m, 1H), 1.89 – 1.81 (m, 3H), 1.52 – 1.46 (m, 3H), 1.13 – 1.04 (m, 2H), 0.94 (brs, 4H). ^{13}C NMR (126 MHz, $CDCl_3$) δ ppm 191.90, 155.15, 134.60, 129.90, 127.53, 44.56, 35.36, 33.96, 32.30, 22.57. $[M+H]^+$ calculated for $C_{14}H_{18}O$ is 203.1430 and found 203.1435.

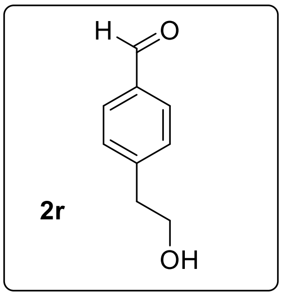

4-(2-hydroxyethyl)benzaldehyde ($C_9H_{10}O_2$) (2r): [ref-4] Synthesized using general procedure **GP-C** (with 0.2 mmol of corresponding phenol), purified by silica gel chromatography (20 to 25% EtOAc/hexane), colourless liquid, yield (16 mg) 53% from corresponding phenol. We have also synthesized it from its corresponding quinketal which resulted the desire product with 73% isolated yield calculated from the quinketal. ^{1}H NMR (500 MHz, $CDCl_3$) δ ppm 9.99 (s, 1H), 7.84 (d, *J* = 8.0 Hz, 2H), 7.41 (d, *J* = 7.9 Hz, 2H), 3.92 (t, *J* = 6.5 Hz, 2H), 2.96 (t, *J* = 6.5 Hz, 2H). ^{13}C NMR (126 MHz, $CDCl_3$) δ ppm 191.99, 146.28, 135.23, 130.18, 129.86, 63.28, 39.50.

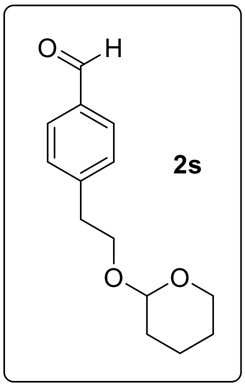

4-(2-((tetrahydro-2H-pyran-2-yl)oxy)ethyl)benzaldehyde ($C_{14}H_{18}O_3$) (2s): Synthesized using general procedure **GP-C** (with 0.2 mmol of corresponding phenol), purified by silica gel chromatography (10 to 15% EtOAc/hexane), colourless liquid, yield (27 mg) 58% from corresponding phenol. We have also synthesized it from its corresponding quinketal which resulted the desire product with 76% isolated yield calculated from the quinketal. IR (Neat) cm^{-1} : 3060, 2972, 2942, 2870, 2735, 1739, 1699, 1607, 1510, 1453, 1353, 1201, 1120, 1079, 1031, 971. ^{1}H NMR (400 MHz, $CDCl_3$) δ ppm 9.98 (s, 1H), 7.81 (d, *J* = 7.8 Hz, 2H), 7.41 (d, *J* = 7.9 Hz, 2H), 4.58 (s, 1H), 3.98 (dt, *J* = 9.2, 7.0 Hz, 1H), 3.71 – 3.61 (m, 2H), 3.45 – 3.42 (m, 1H), 3.00 – 2.96 (m, 2H), 1.78 – 1.67 (m, 1H), 1.56 – 1.48 (m, 5H). ^{13}C NMR (126 MHz, $CDCl_3$) δ ppm 192.11, 147.01, 134.98, 129.94, 129.85, 98.92, 67.59, 62.32, 36.74, 30.76, 25.57, 19.57. $[M+H]^+$ calculated for $C_{14}H_{18}O_3$ is 235.1329 and found 235.1344.

3(b). Synthesis of ketones (w.r.t phenol variation):

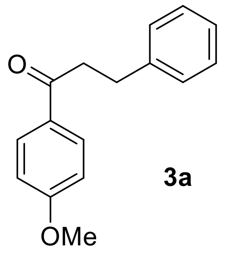

3a

1-(4-methoxyphenyl)-3-phenylpropan-1-one ($C_{16}H_{16}O_2$) (3a): [ref-5] Synthesized using general procedure **GP-D** (with 0.2 mmol of corresponding phenol), purified by silica gel chromatography (20 to 25% EtOAc/hexane), colourless liquid, yield (43 mg) 90% from corresponding phenol. ^{1}H NMR (400 MHz, $CDCl_3$) δ ppm 7.94 (d, *J* = 8.7 Hz, 2H), 7.32 – 7.24 (m, 4H), 7.20 (t, *J* = 7.3 Hz, 1H), 6.92 (d, *J* = 8.7 Hz, 2H), 3.85 (s, 3H), 3.26 – 3.22 (m, 2H), 3.07 – 3.03 (m, 2H). ^{13}C NMR (101 MHz, $CDCl_3$) δ ppm 197.49, 163.08, 141.10, 129.96, 129.57, 128.16, 128.08, 125.73, 113.37, 55.15, 39.77, 29.96.

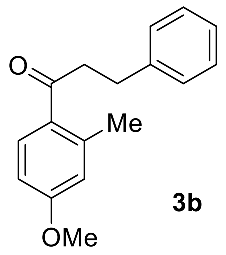

3b

1-(4-methoxy-2-methylphenyl)-3-phenylpropan-1-one ($C_{17}H_{18}O_2$) (3b): Synthesized using general procedure **GP-D** (with 0.2 mmol of corresponding phenol), purified by silica gel chromatography (10 to 15% EtOAc/hexane), colourless liquid, yield (36 mg) 72% from corresponding phenol. IR (Neat) cm^{-1} : 3071, 3027, 2959, 2926, 2840, 1712, 1675, 1570, 1503, 1454, 1362, 1266, 1141, 1029, 998. ^{1}H NMR (400 MHz, $CDCl_3$) δ ppm 7.67 (d, *J* = 8.3 Hz, 1H), 7.29 – 7.18 (m, 5H), 6.73 (brs, 2H), 3.80 (s, 3H), 3.20 – 3.17 (m, 2H), 3.03 – 2.99 (m, 2H), 2.53 (s, 3H). ^{13}C NMR (126 MHz, $CDCl_3$) δ ppm 200.59, 161.49, 141.61, 141.14, 131.10, 129.78, 128.14, 128.08, 125.71, 117.20, 110.29, 54.97, 42.19, 30.31, 21.94. $[M+H]^+$ calculated for $C_{17}H_{18}O_2$ is 255.1380 and found is 255.1385.

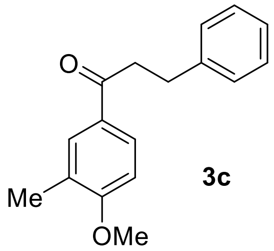

3c

1-(4-methoxy-3-methylphenyl)-3-phenylpropan-1-one ($C_{17}H_{18}O_2$) (3c): [ref-5] Synthesized using general procedure **GP-D** (with 0.2 mmol of corresponding phenol), purified by silica gel chromatography (20 to 25% EtOAc/hexane), colourless liquid, yield (42 mg) 82% from corresponding phenol. ^{1}H NMR (400 MHz, $CDCl_3$) δ ppm 7.82 (d, *J* = 8.5 Hz, 1H), 7.77 (s, 1H), 7.32 – 7.27 (m, 2H), 7.25 – 7.24 (m, 2H), 7.19 – 7.18 (m, 1H), 6.83 (d, *J* = 8.4 Hz, 1H), 3.88 (s, 3H), 3.25 – 3.22 (m, 2H), 3.04 (t, *J* = 7.6 Hz, 2H), 2.23 (s, 3H). ^{13}C NMR (101 MHz, $CDCl_3$) δ

ppm 197.48, 161.04, 140.87, 130.01, 128.73, 127.83, 127.77, 127.46, 126.07, 125.39, 108.54, 54.91, 39.45, 29.71, 15.62.

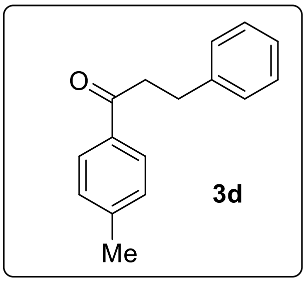

3-phenyl-1-(p-tolyl)propan-1-one ($C_{16}H_{16}O$) (3d): [ref-5] Synthesized using general procedure **GP-D** (with 0.2 mmol of corresponding phenol), purified by silica gel chromatography (10 to 15% EtOAc/hexane), colourless liquid, yield (38 mg) 86% from corresponding phenol. ^{1}H NMR (400 MHz, $CDCl_3$) δ ppm 7.85 – 7.82 (m, 2H), 7.30 – 7.17 (m, 7H), 3.27 – 3.22 (m, 2H), 3.06 – 3.01 (m, 2H), 2.39 (s, 3H). ^{13}C NMR (101 MHz, $CDCl_3$) δ ppm 198.91, 143.84, 141.42, 134.41, 129.30, 128.54, 128.45, 128.19, 126.12, 40.36, 30.24, 21.65.

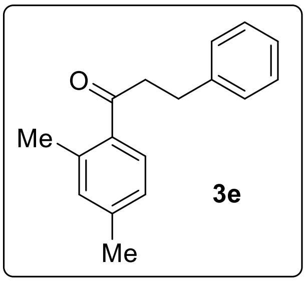

1-(2,4-dimethylphenyl)-3-phenylpropan-1-one ($C_{17}H_{18}O$) (3e): [ref-5] Synthesized using general procedure **GP-D** (with 0.2 mmol of corresponding phenol), purified by silica gel chromatography (10 to 15% EtOAc/hexane), colourless liquid, yield (30 mg) 63% from corresponding phenol. We have also synthesized it from its corresponding quinketal which resulted the desire product with 81% isolated yield calculated from the quinketal. ^{1}H NMR (400 MHz, $CDCl_3$) δ ppm 7.58 (d, J = 7.6 Hz, 1H), 7.29 – 7.265 (m, 5H), 7.07 – 7.02 (m, 2H), 3.25 – 3.21 (m, 2H), 3.07 – 3.03 (m, 2H), 2.49 (s, 3H), 2.36 (s, 3H). ^{13}C NMR (101 MHz, $CDCl_3$) δ ppm 202.73, 142.03, 141.47, 138.84, 134.91, 133.03, 129.13, 128.62, 128.55, 126.41, 126.19, 43.05, 30.59, 21.67, 21.48.

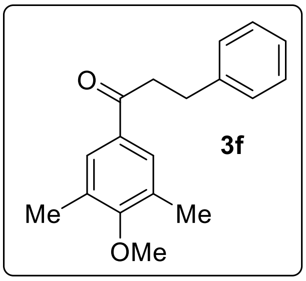

1-(4-methoxy-3,5-dimethylphenyl)-3-phenylpropan-1-one ($C_{18}H_{20}O_2$) (3f): [ref-5] Synthesized using general procedure **GP-D** (with 0.2 mmol of corresponding phenol), purified by silica gel chromatography (10 to 15% EtOAc/hexane), colourless liquid, yield (42 mg) 80% from corresponding phenol. ^{1}H NMR (400 MHz, $CDCl_3$) δ ppm 7.66 (brs, 2H), 7.33 – 7.20 (m, 5H), 3.77 (s, 3H), 3.27 (t, J = 7.6 Hz, 2H), 3.06 (t, J = 7.6 Hz, 2H), 2.33 (s, 6H). ^{13}C NMR (126 MHz, $CDCl_3$) δ ppm 198.65, 161.31, 141.55, 132.71, 131.20, 129.18, 128.55, 128.48, 126.13, 59.70, 40.36, 30.37, 16.27.

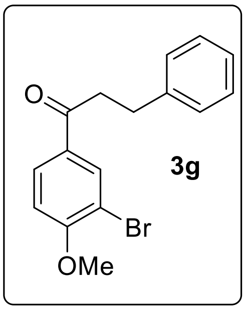

1-(3-bromo-4-methoxyphenyl)-3-phenylpropan-1-one ($C_{16}H_{15}BrO_2$) (3g): Synthesized using general procedure **GP-D** (with 0.2 mmol of corresponding quinketal), purified by silica gel chromatography (10 to 15% EtOAc/hexane), colourless liquid, yield (40 mg) 63% from corresponding quinketal. IR (Neat) cm^{-1} : 3079, 3027, 2918, 2849, 1738, 1686, 1595, 1565, 1496, 1454, 1405, 1360, 1313, 1270, 1193, 1051, 906, 816, 747, 698. ^{1}H NMR (400 MHz, $CDCl_3$) δ ppm 8.17 (s, 1H), 7.91 (d, *J* = 8.6 Hz, 1H), 7.31 (t, *J* = 7.2 Hz, 2H), 7.27 – 7.20 (m, 3H), 6.92 (d, *J* = 8.6 Hz, 1H), 3.96 (s, 3H), 3.24 (t, *J* = 7.5 Hz, 2H), 3.06 (t, *J* = 7.5 Hz, 2H). ^{13}C NMR (126 MHz, $CDCl_3$) δ ppm 196.84, 159.72, 141.32, 133.76, 131.13, 129.30, 128.68, 128.55, 126.32, 112.10, 111.28, 56.60, 40.25, 30.32. $[M+H]^+$ calculated for $C_{16}H_{15}BrO_2$ is 319.0328 and found 319.0329.

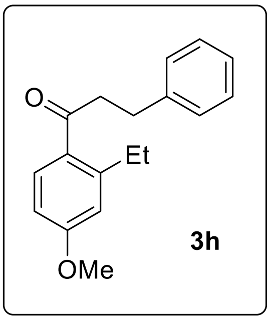

1-(2-ethyl-4-methoxyphenyl)-3-phenylpropan-1-one ($C_{18}H_{20}O_2$) (3h): Synthesized using general procedure **GP-D** (with 0.2 mmol of corresponding phenol), purified by silica gel chromatography (10 to 15% EtOAc/hexane), colourless liquid, yield (41 mg) 78% from corresponding phenol. IR (Neat) cm^{-1} : 3066, 3027, 2963, 2929, 2865, 1703, 1687, 1601, 1563, 1495, 1454, 1310, 1233, 1207, 1126, 1077, 1029, 969. ^{1}H NMR (400 MHz, $CDCl_3$) δ ppm 7.65 (d, *J* = 8.7 Hz, 1H), 7.30 – 7.27 (m, 2H), 7.24 – 7.17 (m, 3H), 6.78 (brs, 1H), 6.73 (dd, *J* = 8.6, 2.3 Hz, 1H), 3.84 (s, 3H), 3.21 (t, *J* = 7.8 Hz, 2H), 3.05 – 3.01 (m, 2H), 2.91 – 2.86 (m, 2H), 1.20 (t, *J* = 7.4 Hz, 3H). ^{13}C NMR (126 MHz, $CDCl_3$) δ ppm 201.54, 162.09, 148.05, 141.59, 131.45, 130.28, 128.62, 128.57, 126.19, 116.23, 110.57, 55.41, 43.08, 30.81, 27.78, 15.85. $[M+H]^+$ calculated for $C_{18}H_{20}O_2$ is 269.1536 and found 269.1539.

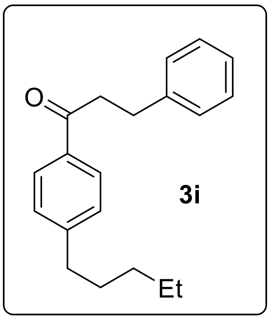

1-(4-pentylphenyl)-3-phenylpropan-1-one ($C_{20}H_{24}O$) (3i): Synthesized using general procedure **GP-D** (with 0.2 mmol of corresponding phenol), purified by silica gel chromatography (10 to 15% EtOAc/hexane), colourless liquid, yield (46 mg) 82% from corresponding phenol. IR (Neat) cm^{-1} : 3062, 3028, 2963, 2928, 2857, 1708, 1689, 1606, 1581, 1496, 1454, 1413, 1362, 1290, 1180, 1118, 1077, 1031, 977. ^{1}H NMR (400 MHz, $CDCl_3$) δ ppm 7.87 (d, *J* = 8.1 Hz, 2H), 7.31 – 7.17

(m, 7H), 3.26 (dd, *J* = 9.9, 5.5 Hz, 2H), 3.07 – 3.03 (m, 2H), 2.64 (t, *J* = 7.7 Hz, 2H), 1.63 – 1.59 (m, 2H), 1.31 – 1.30 (m, 4H), 0.88 (t, *J* = 6.7 Hz, 3H). ^{13}C NMR (101 MHz, $CDCl_3$) δ ppm 198.97, 148.87, 141.52, 134.66, 128.73, 128.60, 128.53, 128.28, 126.18, 40.45, 36.04, 31.52, 30.90, 30.29, 22.60, 14.11. $[M+H]^+$ calculated for $C_{20}H_{24}O$ is 281.1900 and found 281.1906.

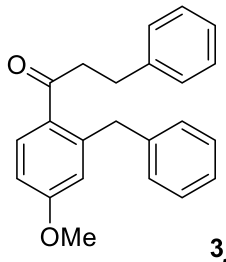

3j

1-(2-benzyl-4-methoxyphenyl)-3-phenylpropan-1-one ($C_{23}H_{22}O_2$) (3j): Synthesized using general procedure **GP-D** (with 0.2 mmol of corresponding phenol), purified by silica gel chromatography (10 to 15% EtOAc/hexane), colourless liquid, yield (36 mg) 71% from corresponding phenol. IR (Neat) cm^{-1} : 3023, 2925, 2855, 1682, 1605, 1564, 1485, 1456, 1330, 1295, 1215, 1181, 1128, 1079, 1038, 1019, 975, 921. ^{1}H NMR (500 MHz, $CDCl_3$) δ ppm 7.61 (d, *J* = 8.5 Hz, 1H), 7.25 – 7.21 (m, 4H), 7.15 – 7.11 (m, 6H), 6.74 – 6.17 (m, 2H), 4.28 (s, 2H), 3.76 (s, 3H), 3.06 (t, *J* = 7.7 Hz, 2H), 2.88 (t, *J* = 7.5 Hz, 2H). ^{13}C NMR (101 MHz, $CDCl_3$) δ ppm 201.60, 161.71, 143.92, 141.34, 140.77, 131.20, 130.52, 129.10, 128.44, 128.35, 128.26, 126.00, 125.92, 117.67, 110.69, 55.25, 42.94, 39.62, 30.43. $[M+K]^+$ calculated for $C_{23}H_{22}O_2$ is 369.1251 and found 369.1264.

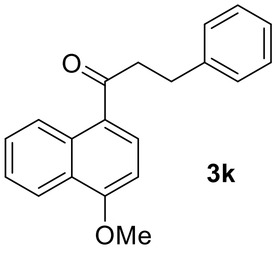

3k

1-(4-methoxynaphthalen-1-yl)-3-phenylpropan-1-one ($C_{20}H_{18}O_2$) (3k): Synthesized using general procedure **GP-D** (with 0.2 mmol of corresponding phenol), purified by silica gel chromatography (10 to 15% EtOAc/hexane), colourless liquid, yield (36 mg) 62% from corresponding phenol. IR (Neat) cm^{-1} : 3092, 3026, 2925, 2850, 1716, 1669, 1578, 1517, 1465, 1424, 1326, 1231, 1164, 1105, 1030, 988. ^{1}H NMR (500 MHz, $CDCl_3$) δ ppm 8.84 (d, *J* = 8.7 Hz, 1H), 8.30 (d, *J* = 8.4 Hz, 1H), 7.91 (d, *J* = 8.2 Hz, 1H), 7.60 – 7.57 (m, 1H), 7.51 – 7.48 (m, 1H), 7.27 – 7.23 (m, 4H), 7.19 – 7.16 (m, 1H), 6.75 (d, *J* = 8.2 Hz, 1H), 4.04 (s, 3H), 3.36 – 3.33 (m, 2H), 3.12 – 3.09 (m, 2H). ^{13}C NMR (126 MHz, $CDCl_3$) δ ppm 201.72, 159.20, 141.64, 132.20, 130.59, 128.73, 128.66, 128.63, 128.43, 127.95, 126.47, 126.23, 125.96, 122.27, 102.20, 55.93, 43.11, 31.19. $[M+H]^+$ calculated for $C_{20}H_{18}O_2$ is 291.1380 and found 291.1386.

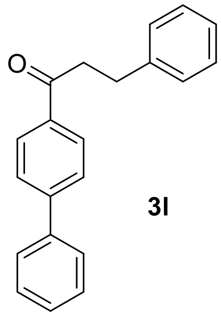

3l

1-([1,1'-biphenyl]-4-yl)-3-phenylpropan-1-one ($C_{21}H_{18}O$) (3l): ref-5 Synthesized using general procedure **GP-D** (with 0.2 mmol of corresponding phenol), purified by silica gel chromatography (10 to 15% EtOAc/hexane), colourless liquid, yield (43 mg) 76% from corresponding phenol. ^{1}H NMR (400 MHz, $CDCl_3$) δ ppm 8.04 (d, J = 8.3 Hz, 2H), 7.68 (d, J = 8.3 Hz, 2H), 7.64 – 7.62 (m, 2H), 7.49 – 7.46 (m, 2H), 7.42 – 7.40 (m, 1H), 7.32 – 7.27 (m, 4H), 7.24 – 7.22 (m, 1H), 3.34 (t, J = 7.7 Hz, 2H), 3.12 – 3.08 (m, 2H). ^{13}C NMR (101 MHz, $CDCl_3$) δ ppm 198.97, 145.87, 141.44, 139.99, 135.67, 129.08, 128.77, 128.68, 128.57, 128.36, 127.39, 127.38, 126.28, 40.65, 30.33.

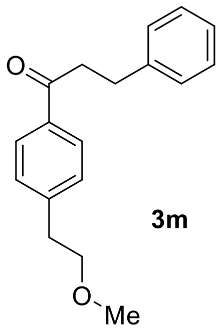

3m

1-(4-(2-methoxyethyl)phenyl)-3-phenylpropan-1-one ($C_{18}H_{20}O_2$) (3m): Synthesized using general procedure **GP-D** (with 0.2 mmol of corresponding phenol), purified by silica gel chromatography (10 to 15% EtOAc/hexane), colourless liquid, yield (40 mg) 75% from corresponding phenol. IR (Neat) cm^{-1} : 3075, 3028, 2926, 2869, 2833, 1702, 1693, 1607, 1572, 1495, 1453, 1413, 1363, 1291, 1218, 1180, 1115, 977. ^{1}H NMR (500 MHz, $CDCl_3$) δ ppm 7.88 (d, J = 8.1 Hz, 2H), 7.28 (dd, J = 9.7, 8.1 Hz, 4H), 7.24 (d, J = 6.7 Hz, 2H), 7.18 (t, J = 7.1 Hz, 1H), 3.61 (t, J = 6.8 Hz, 2H), 3.33 (s, 3H), 3.27 – 3.24 (m, 2H), 3.05 (t, J = 7.7 Hz, 2H), 2.92 (t, J = 6.7 Hz, 2H). ^{13}C NMR (101 MHz, $CDCl_3$) δ ppm 198.89, 144.95, 141.41, 135.09, 129.14, 128.56, 128.47, 128.29, 126.15, 72.93, 58.76, 40.40, 36.23, 30.21. $[M+H]^+$ calculated for $C_{18}H_{20}O_2$ is 269.1536 and found 269.1538.

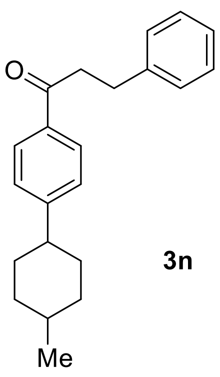

3n

1-(4-(4-methylcyclohexyl)phenyl)-3-phenylpropan-1-one ($C_{22}H_{26}O_2$) (3n): Synthesized using general procedure **GP-D** (with 0.2 mmol of corresponding phenol), purified by silica gel chromatography (10 to 15% EtOAc/hexane), colourless liquid, yield (42 mg) 69% from corresponding phenol. IR (Neat) cm^{-1} : 3026, 2926, 2851, 1684, 1606, 1499, 1454, 1410, 1364, 1292, 1182, 1080, 1031, 977, 920. ^{1}H NMR (500 MHz, $CDCl_3$) δ ppm 7.87 (d, J = 8.2 Hz, 2H), 7.29 – 7.22 (m, 6H), 7.18 (t, J = 7.1 Hz, 1H), 3.26 (m, 2H), 3.04 (t, J = 7.7 Hz, 2H), 2.51 – 2.46 (m, 1H), 1.87 – 1.79 (m, 4H), 1.50 – 1.39 (m, 3H), 1.11 – 1.06 (m, 2H), 0.93 (d, J = 6.5 Hz, 3H). ^{13}C NMR (101 MHz, $CDCl_3$) δ ppm 198.94, 153.62,

141.53, 134.84, 128.60, 128.52, 128.35, 127.20, 126.17, 44.42, 40.44, 35.50, 34.10, 32.42, 30.31, 22.76. $[M+NH_4]^+$ calculated for $C_{22}H_{26}O_2$ is 324.2322 and found 324.2333.

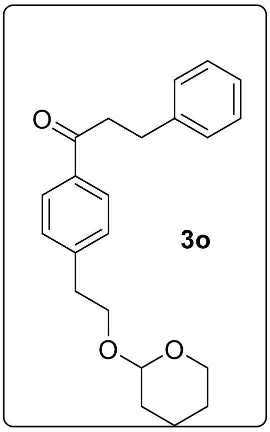

3-phenyl-1-(4-(2-((tetrahydro-2H-pyran-2-yl)oxy)ethyl)phenyl)propan-1-one ($C_{22}H_{26}O_3$) (3o): Synthesized using general procedure **GP-D** (with 0.2 mmol of corresponding phenol), purified by silica gel chromatography (10 to 15% EtOAc/hexane), colourless liquid, yield (41 mg) 61% from corresponding phenol. We have also synthesized it from its corresponding quinketal which resulted the desire product with 73% isolated yield calculated from the quinketal. IR (Neat) cm^{-1} : 3067, 3027, 2935, 2868, 1715, 1683, 1633, 1604, 1496, 1454, 1380, 1253, 1201, 1121, 1080, 1041, 970. ^1H NMR (400 MHz, $CDCl_3$) δ ppm 7.89 (d, J = 8.0 Hz, 2H), 7.33 (d, J = 7.9 Hz, 2H), 7.29 (d, J = 7.4 Hz, 2H), 7.23 (d, J = 8.4 Hz, 2H), 7.22 – 7.18 (m, 1H), 4.58 (brs, 1H), 3.97 (dd, J = 16.5, 6.9 Hz, 1H), 3.71 (t, J = 8.5 Hz, 1H), 3.63 (dd, J = 16.4, 6.8 Hz, 1H), 3.45 – 3.43 (m, 1H), 3.28 (t, J = 7.7 Hz, 2H), 3.06 (t, J = 7.7 Hz, 2H), 2.96 (t, J = 6.8 Hz, 2H), 1.79 – 1.65 (m, 1H), 1.53 – 1.48 (m, 5H). ^{13}C NMR (101 MHz, $CDCl_3$) δ ppm 199.12, 145.25, 141.51, 135.13, 129.40, 128.66, 128.56, 128.28, 126.25, 98.88, 67.72, 62.33, 40.55, 36.49, 30.75, 30.34, 25.55, 19.57. $[M+NH_4]^+$ calculated for $C_{22}H_{26}O_3$ is 356.2220 and found 356.2227.

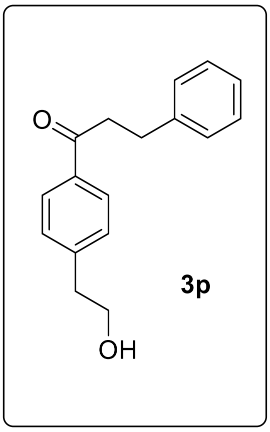

1-(4-(2-hydroxyethyl)phenyl)-3-phenylpropan-1-one ($C_{17}H_{18}O_2$) (3p): Synthesized using general procedure **GP-D** (with 0.2 mmol of corresponding phenol), purified by silica gel chromatography (10 to 15% EtOAc/hexane), colourless liquid, yield (31 mg) 62% from corresponding phenol. IR (Neat) cm^{-1} : 3403, 3070, 3027, 2931, 2869, 1704, 1687, 1607, 1496, 1454, 1413, 1365, 1292, 1182, 1047, 978. ^1H NMR (400 MHz, $CDCl_3$) δ ppm 7.91 (d, J = 7.9 Hz, 2H), 7.32 (d, J = 8.0 Hz, 2H), 7.28 – 7.19 (m, 5H), 3.89 (t, J = 6.4 Hz, 2H), 3.28 (t, J = 7.5 Hz, 2H), 3.06 (t, J = 8.0 Hz, 2H), 2.93 (t, J = 6.4 Hz, 2H). ^{13}C NMR (126 MHz, $CDCl_3$) δ ppm 199.02, 144.55, 141.47, 135.49, 129.41, 128.67, 128.56, 128.54, 126.27, 63.34, 40.52, 39.28, 30.35. $[M+H]^+$ calculated for $C_{17}H_{18}O_2$ is 255.1380 and found 255.1381.

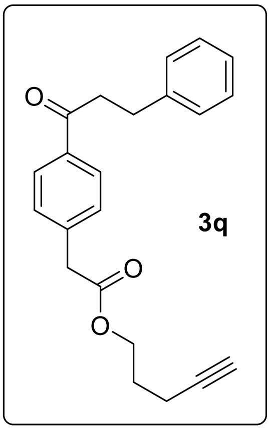

pent-4-yn-1-yl 2-(4-(3-phenylpropanoyl)phenyl)acetate ($C_{22}H_{22}O_3$) (3q): Synthesized using general procedure **GP-G** (with 0.2 mmol of corresponding phenol), purified by silica gel chromatography (10 to 15% EtOAc/hexane), colourless liquid, yield (44 mg) 66% from corresponding phenol. IR (Neat) cm^{-1} : 3292, 3092, 3028, 2959, 2927, 2851, 2252, 1734, 1705, 1638, 1608, 1571, 1496, 1464, 1416, 1340, 1297, 1253, 1225, 1160, 1030, 979. ^1H NMR (500 MHz, $CDCl_3$) δ ppm 7.93 (d, J = 8.2 Hz, 2H), 7.37 (d, J = 8.2 Hz, 2H), 7.32 – 7.28 (m, 2H), 7.27 - 7.24 (m, 1H), 7.22 – 7.18 (m, 2H), 4.22 (t, J = 6.3 Hz, 2H), 3.68 (s, 2H), 3.30 – 3.27 (m, 2H), 3.07 (t, J = 7.7 Hz, 2H), 2.24 (td, J = 7.0, 2.6 Hz, 2H), 1.96 (t, J = 2.6 Hz, 1H), 1.85 (p, J = 6.7 Hz, 2H). ^{13}C NMR (126 MHz, $CDCl_3$) δ ppm 198.87, 170.14, 141.43, 141.33, 137.28, 128.69, 128.56, 128.52, 127.42, 126.32, 82.30, 69.34, 64.07, 57.78, 40.68, 30.25, 27.48, 15.14. $[M+H]^+$ calculated for $C_{22}H_{22}O_3$ is 335.1642 and found 335.1647.

3(c). Synthesis of ketones (w.r.t germinal B(pin) variation):

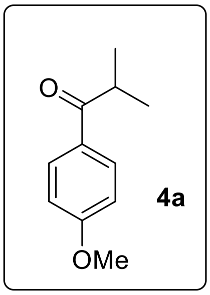

1-(4-methoxyphenyl)-2-methylpropan-1-one ($C_{11}H_{14}O_2$) (4a): [ref-5] Synthesized using general procedure **GP-D** (with 0.2 mmol of corresponding phenol), purified by silica gel chromatography (10 to 15% EtOAc/hexane), colourless liquid, yield (28 mg) 79% from corresponding phenol. ^1H NMR (400 MHz, $CDCl_3$) δ ppm 8.01 (d, J = 8.4 Hz, 2H), 7.00 (d, J = 8.4 Hz, 2H), 3.93 (s, 3H), 3.58 (dt, J = 13.3, 6.6 Hz, 1H), 1.28 (s, 3H), 1.26 (s, 3H). ^{13}C NMR (101 MHz, $CDCl_3$) δ ppm 201.85, 161.99, 129.31, 127.83, 112.50, 54.30, 33.66, 18.06.

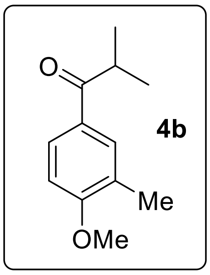

1-(4-methoxy-3-methylphenyl)-2-methylpropan-1-one ($C_{12}H_{16}O_2$) (4b): [ref-5] Synthesized using general procedure **GP-D** (with 0.2 mmol of corresponding phenol), purified by silica gel chromatography (10 to 15% EtOAc/hexane), colourless liquid, yield (29 mg) 76% from corresponding phenol. ^1H NMR (400 MHz, $CDCl_3$) δ ppm 7.62 (d, J = 8.6 Hz, 1H), 6.75 – 6.73 (d, J = 7.4 Hz, 2H), 3.84 (s, 3H), 3.41 (dd, J = 13.6, 6.8 Hz, 1H), 2.48 (s, 3H), 1.16 (s, 3H), 1.15 (s, 3H). ^{13}C NMR (101 MHz, $CDCl_3$) δ ppm 207.14, 161.53, 141.73, 130.70, 130.33, 117.47, 110.64, 55.41, 37.75, 22.07, 19.16.

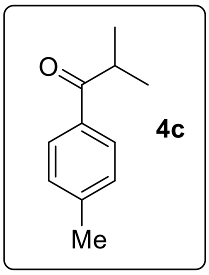

2-methyl-1-(p-tolyl)propan-1-one ($C_{11}H_{14}O$) (4c): [ref-5] Synthesized using general procedure **GP-D** (with 0.2 mmol of corresponding phenol), purified by silica gel chromatography (10 to 15% EtOAc/hexane), colourless liquid, yield (24 mg) 75% from corresponding phenol. ^{1}H NMR (400 MHz, $CDCl_3$) δ ppm 7.86 (d, J = 8.1 Hz, 2H), 7.27 – 7.25 (m, 2H), 3.54 (dt, J = 13.7, 6.9 Hz, 1H), 2.41 (s, 3H), 1.22 (s, 3H), 1.19 (s, 3H). ^{13}C NMR (126 MHz, $CDCl_3$) δ ppm 204.25, 143.60, 133.93, 129.41, 128.58, 35.36, 21.68, 19.34.

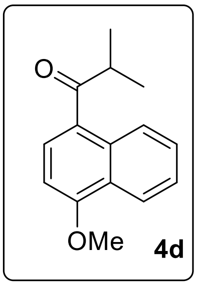

1-(4-methoxynaphthalen-1-yl)-2-methylpropan-1-one ($C_{15}H_{16}O_2$) (4d): Synthesized using general procedure **GP-D** (with 0.2 mmol of corresponding phenol), purified by silica gel chromatography (10 to 15% EtOAc/hexane), colourless liquid, yield (28 mg) 62% from corresponding phenol. IR (Neat) cm^{-1} : 3079, 2976, 2927, 2853, 1711, 1669, 1578, 1512, 1464, 1425, 1377, 1326, 1227, 1161, 1097, 989. ^{1}H NMR (400 MHz, $CDCl_3$) δ ppm 8.60 (d, J = 8.2 Hz, 1H), 8.31 (d, J = 8.4 Hz, 1H), 7.86 (d, J = 8.1 Hz, 1H), 7.59 (ddd, J = 8.5, 6.9, 1.4 Hz, 1H), 7.55 – 7.48 (m, 1H), 6.80 (d, J = 8.1 Hz, 1H), 4.06 (s, 3H), 3.58 (dt, J = 13.7, 6.8 Hz, 1H), 1.25 (s, 3H), 1.23 (s, 3H). ^{13}C NMR (101 MHz, $CDCl_3$) δ ppm 207.56, 158.59, 132.37, 129.03, 128.42, 128.37, 126.04, 125.90, 122.22, 102.16, 55.88, 38.48, 19.36. $[M+H]^+$ calculated for $C_{15}H_{16}O_2$ is 229.1223 and found 229.1226.

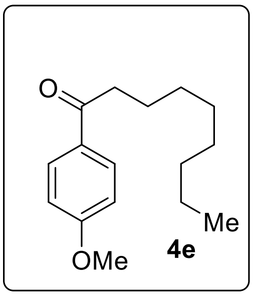

1-(4-methoxyphenyl)nonan-1-one ($C_{16}H_{24}O_2$) (4e): [ref-5] Synthesized using general procedure **GP-D** (with 0.2 mmol of corresponding phenol), purified by silica gel chromatography (10 to 15% EtOAc/hexane), colourless liquid, yield (40 mg) 81% from corresponding phenol. ^{1}H NMR (400 MHz, $CDCl_3$) δ ppm 7.80 (d, J = 8.8 Hz, 2H), 6.77 (d, J = 8.8 Hz, 2H), 3.69 (s, 3H), 2.75 (t, J = 7.4 Hz, 2H), 1.62 – 1.54 (m, 2H), 1.20 – 1.15 (m, 10H), 0.76 (t, J = 6.6 Hz, 3H). ^{13}C NMR (101 MHz, $CDCl_3$) δ ppm 198.69, 163.14, 130.08, 130.00, 113.46, 55.14, 38.07, 31.74, 29.39, 29.32, 29.09, 24.45, 22.54, 13.95.

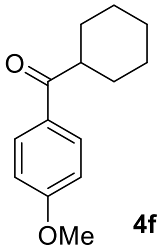

4f

cyclohexyl(4-methoxyphenyl)methanone ($C_{14}H_{18}O_2$) (4f): [ref-5] Synthesized using general procedure **GP-D** (with 0.2 mmol of corresponding phenol), purified by silica gel chromatography (10 to 15% EtOAc/hexane), colourless liquid, yield (32 mg) 74% from corresponding phenol. ^{1}H NMR (400 MHz, $CDCl_3$) δ ppm 7.96 (d, *J* = 8.7 Hz, 2H), 6.95 (d, *J* = 8.7 Hz, 2H), 3.88 (s, 3H), 3.24 (ddd, *J* = 11.4, 7.4, 2.9 Hz, 1H), 1.90 – 1.84 (m, 4H), 1.77 – 1.74 (m, 1H), 1.53 – 1.47 (m, 2H), 1.46 – 1.35 (m, 2H), 1.34 – 1.24 (m, 2H). ^{13}C NMR (101 MHz, $CDCl_3$) δ ppm 201.94, 162.65, 129.94, 128.64, 113.15, 54.92, 44.72, 28.98, 25.42, 25.35.

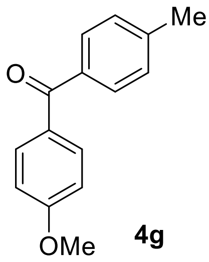

4g

(4-methoxyphenyl)(p-tolyl)methanone ($C_{15}H_{14}O_2$) (4g): [ref-5] Synthesized using general procedure **GP-D** (with 0.2 mmol of corresponding phenol), purified by silica gel chromatography (10 to 15% EtOAc/hexane), colourless liquid, yield (29 mg) 65% from corresponding phenol. ^{1}H NMR (400 MHz, $CDCl_3$) δ ppm 7.83 – 7.80 (m, 2H), 7.70 – 7.66 (m, 2H), 7.33 – 7.26 (m, 2H), 6.98 – 6.94 (m, 2H), 3.88 (s, 3H), 2.43 (s, 3H). ^{13}C NMR (101 MHz, $CDCl_3$) δ ppm 195.37, 163.06, 142.63, 135.53, 132.45, 130.49, 130.02, 128.90, 113.51, 55.50, 21.63.

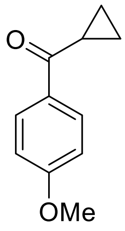

4h

cyclopropyl(4-methoxyphenyl)methanone ($C_{11}H_{12}O_2$) (4h): [ref-5] Synthesized using general procedure **GP-D** (with 0.2 mmol of corresponding phenol), purified by silica gel chromatography (10 to 15% EtOAc/hexane), colourless liquid, yield (23 mg) 67% from corresponding phenol. ^{1}H NMR (400 MHz, $CDCl_3$) δ ppm 8.01 (d, *J* = 8.8 Hz, 2H), 6.95 (d, *J* = 8.8 Hz, 2H), 3.87 (s, 3H), 2.66 – 2.60 (m, 1H), 1.20 – 1.19 (m, 2H), 1.01 – 0.98 (m, 2H). ^{13}C NMR (101 MHz, $CDCl_3$) δ ppm 199.07, 163.30, 131.03, 130.25, 113.65, 55.48, 16.64, 11.25.

3(d). Synthesis of acetophenones:

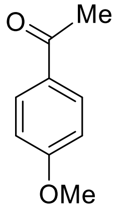

5a

1-(4-methoxyphenyl)ethan-1-one ($C_9H_{10}O_2$) (5a): [ref-5] Synthesized using general procedure **GP-E** (with 0.2 mmol of corresponding

phenol), purified by silica gel chromatography (10 to 15% EtOAc/hexane), colourless liquid, yield (22 mg) 72% from corresponding phenol. ^{1}H NMR (500 MHz, $CDCl_3$) δ ppm 7.80 (d, J = 8.8 Hz, 2H), 6.80 (d, J = 8.8 Hz, 2H), 3.72 (s, 3H), 2.41 (s, 3H). ^{13}C NMR (126 MHz, $CDCl_3$) δ ppm 196.33, 163.29, 130.32, 130.12, 113.47, 55.18, 25.99.

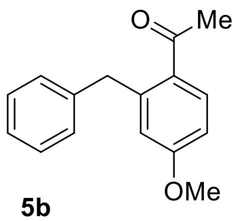

5b

1-(2-benzyl-4-methoxyphenyl)ethan-1-one ($C_{16}H_{16}O_2$) (5b): **ref-5** Synthesized using general procedure **GP-E** (with 0.2 mmol of corresponding phenol), purified by silica gel chromatography (10 to 15% EtOAc/hexane), colourless liquid, yield (31 mg) 65% from corresponding phenol. ^{1}H NMR (400 MHz, $CDCl_3$) δ ppm 7.76 (d, J = 8.5 Hz, 1H), 7.25 (d, J = 8.0 Hz, 2H), 7.16 (t, J = 8.8 Hz, 3H), 6.79 (d, J = 8.8 Hz, 1H), 6.71 (brs, 1H), 4.35 (s, 2H), 3.80 (s, 3H), 2.49 (s, 3H). ^{13}C NMR (126 MHz, $CDCl_3$) δ ppm 199.94, 162.15, 144.45, 140.96, 132.56, 130.42, 129.23, 128.44, 126.07, 117.87, 110.91, 55.44, 39.95, 29.39.

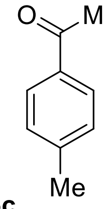

5c

1-(p-tolyl)ethan-1-one ($C_9H_{10}O$) (5c): **ref-5** Synthesized using general procedure **GP-E** (with 0.2 mmol of corresponding phenol), purified by silica gel chromatography (10 to 15% EtOAc/hexane), colourless liquid, yield (19 mg) 68% from corresponding phenol. ^{1}H NMR (500 MHz, $CDCl_3$) δ ppm 7.79 (d, J = 8.2 Hz, 2H), 7.18 (d, J = 8.1 Hz, 2H), 2.50 (s, 3H), 2.34 (s, 3H). ^{13}C NMR (126 MHz, $CDCl_3$) δ ppm 197.54, 143.69, 134.67, 129.12, 128.32, 26.31, 21.44.

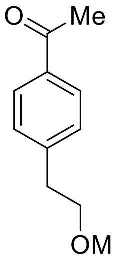

5d

1-(4-(2-methoxyethyl)phenyl)ethan-1-one ($C_{11}H_{14}O_2$) (5d): Synthesized using general procedure **GP-E** (with 0.2 mmol of corresponding phenol), purified by silica gel chromatography (10 to 15% EtOAc/hexane), colourless liquid, yield (25 mg) 70% from corresponding phenol. IR (Neat) cm^{-1} : 3056, 3021, 2926, 2871, 2822, 1708, 1687, 1608, 1567, 1414, 1358, 1269, 1183, 1118, 1018, 957. ^{1}H NMR (400 MHz, $CDCl_3$) δ ppm 7.89 (brs, 2H), 7.32 (brs, 2H), 3.64 – 3.61 (m, 2H), 3.35 (s, 3H), 2.95 – 2.93 (m, 2H), 2.58 (s, 3H). ^{13}C NMR (126 MHz, $CDCl_3$) δ ppm 197.92, 145.09, 135.58, 129.20,

128.65, 73.06, 58.85, 36.35, 26.66. [M+H]$^+$ calculated for $C_{11}H_{14}O_2$ is 179.1066 and found 179.1065.

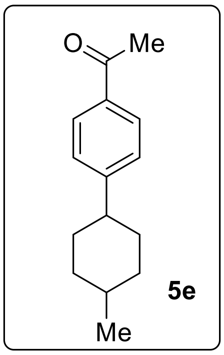

1-(4-(4-methylcyclohexyl)phenyl)ethan-1-one ($C_{15}H_{20}O_2$) (5e): Synthesized using general procedure **GP-E** (with 0.2 mmol of corresponding phenol), purified by silica gel chromatography (10 to 15% EtOAc/hexane), colourless liquid, yield (28 mg) 65% from corresponding phenol. IR (Neat) cm^{-1} : 3055, 2951, 2925, 2850, 1705, 1685, 1607, 1447, 1420, 1358, 1267, 1184, 1079, 1016, 956. ^{1}H NMR (400 MHz, $CDCl_3$) δ 7.89 – 7.88 (m, 2H), 7.30 – 7.25 (m, 2H), 2.60 – 2.57 (m, 3H), 1.85 – 1.84 (m, 4H), 1.47 – 1.46 (m, 2H), 1.26 – 1.25 (m, 1H), 1.09 – 1.08 (m, 2H), 0.95 – 0.92 (m, 4H). ^{13}C NMR (126 MHz, $CDCl_3$) δ ppm 197.99, 153.73, 135.30, 128.68, 127.22, 44.52, 35.58, 34.18, 32.50, 26.65, 22.77. [M+H]$^+$ calculated for $C_{15}H_{20}O_2$ is 217.1587 and found 217.1586.

3(e). Synthesis of α,β-unsaturated ketone:

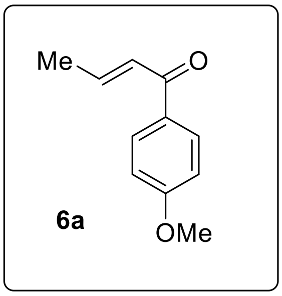

(*E*)-1-(4-methoxyphenyl)but-2-en-1-one ($C_{11}H_{12}O_2$) (6a): ref-6 Synthesized using general procedure **GP-E** (with 0.2 mmol of corresponding phenol), purified by silica gel chromatography (10 to 15% EtOAc/hexane), colourless liquid, yield (21 mg) 61% from corresponding phenol. ^{1}H NMR (500 MHz, $CDCl_3$) δ ppm 7.94 (d, *J* = 8.7 Hz, 2H), 7.07 – 7.00 (m, 1H), 6.95 – 6.90(m, 3H), 3.87 (s, 3H), 1.99 (d, *J* = 7.1 Hz, 3H). ^{13}C NMR (126 MHz, $CDCl_3$) δ ppm 189.15, 163.44, 144.00, 130.93, 127.36, 114.48, 113.89, 55.59, 18.64.

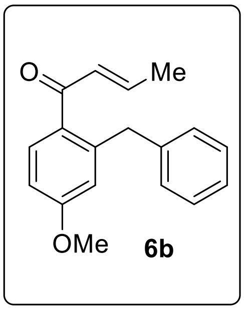

(*E*)-1-(2-benzyl-4-methoxyphenyl)but-2-en-1-one ($C_{18}H_{18}O_2$) (6b): Synthesized using general procedure **GP-E** (with 0.2 mmol of corresponding phenol), purified by silica gel chromatography (10 to 15% EtOAc/hexane), colourless liquid, yield (31 mg) 58% from

corresponding phenol. IR (Neat) cm^{-1} : 3068, 3028, 2969, 2918, 2849, 1675, 1657, 1606, 1567, 1494, 1454, 1329, 1295, 1249, 1230, 1161, 1102, 1031, 971. ^{1}H NMR (400 MHz, $CDCl_3$) δ ppm 7.29 – 7.22 (m, 3H), 7.16 – 7.13 (m, 3H), 6.76 6.63 (m, 3H), 6.54 – 6.44 (m, 1H), 4.18 (s, 2H), 3.78 (s, 3H), 1.88 (d, *J* = 6.6 Hz, 3H). ^{13}C NMR (126 MHz, $CDCl_3$) δ ppm 195.22, 161.42, 145.46, 143.21, 140.87, 132.25, 131.75, 131.06, 129.32, 128.45, 126.11, 117.20, 110.74, 55.41, 39.22, 18.48. [M+H]$^+$ calculated for $C_{18}H_{18}O_2$ is 267.1380 and found 267.1392.

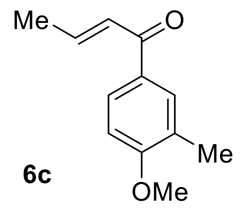

6c

(*E*)-1-(4-methoxy-3-methylphenyl)but-2-en-1-one ($C_{12}H_{14}O_2$) (6c): [ref-6] Synthesized using general procedure **GP-E** (with 0.2 mmol of corresponding phenol), purified by silica gel chromatography (10 to 15% EtOAc/hexane), colourless liquid, yield (20 mg) 52% from corresponding phenol. ^{1}H NMR (500 MHz, $CDCl_3$) δ ppm 7.82 (dd, *J* = 8.5, 2.1 Hz, 1H), 7.78 (brs, 1H), 7.04 (dq, *J* = 13.5, 6.7 Hz, 1H), 6.92 (dd, *J* = 15.2, 1.4 Hz, 1H), 6.86 (d, *J* = 8.5 Hz, 1H), 3.89 (s, 3H), 2.25 (s, 3H), 1.99 (dd, *J* = 6.7, 1.3 Hz, 3H). ^{13}C NMR (126 MHz, $CDCl_3$) δ ppm 189.42, 161.74, 143.69, 131.37, 130.48, 128.74, 127.47, 127.00, 109.39, 55.66, 18.63, 16.37.

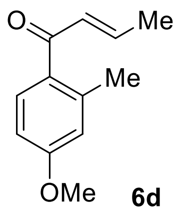

6d

(*E*)-1-(4-methoxy-2-methylphenyl)but-2-en-1-one ($C_{12}H_{14}O_2$) (6d): Synthesized using general procedure **GP-E** (with 0.2 mmol of corresponding phenol), purified by silica gel chromatography (10 to 15% EtOAc/hexane), colourless liquid, yield (20 mg) 54% from corresponding phenol. IR (Neat) cm^{-1} : 3068, 3021, 2973, 2927, 2850, 1668, 1653, 1610, 1571, 1506, 1443, 1377, 1321, 1295, 1243, 1222, 1134, 1055, 971. ^{1}H NMR (500 MHz, $CDCl_3$) δ ppm 7.48 (d, *J* = 8.4 Hz, 1H), 6.80 – 6.73 (m, 3H), 6.58 (d, *J* = 15.4 Hz, 1H), 3.83 (s, 3H), 2.45 (s, 3H), 1.95 (dd, *J* = 6.8, 0.6 Hz, 3H). ^{13}C NMR (126 MHz, $CDCl_3$) δ ppm 194.66, 161.45, 144.82, 140.70, 131.92, 131.50, 131.13, 117.11, 110.55, 55.42, 21.19, 18.52. [M+H]$^+$ calculated for $C_{12}H_{14}O_2$ is 191.1067 and found 191.1070.

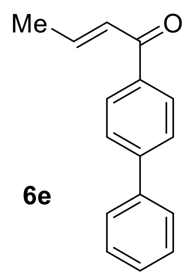

6e

(*E*)-1-([1,1'-biphenyl]-4-yl)but-2-en-1-one ($C_{16}H_{14}O$) (6e): Synthesized using general procedure **GP-E** (with 0.2 mmol of corresponding phenol), purified by silica gel chromatography (10 to

15% EtOAc/hexane), colourless liquid, yield (26 mg) 60% from corresponding phenol. ^{1}H NMR (500 MHz, $CDCl_3$) δ ppm 8.01 (d, *J* = 8.2 Hz, 2H), 7.69 (d, *J* = 8.2 Hz, 2H), 7.64 (d, *J* = 7.3 Hz, 2H), 7.47 (t, *J* = 7.5 Hz, 2H), 7.40 (t, *J* = 7.3 Hz, 1H), 7.11 (dt, *J* = 21.9, 6.8 Hz, 1H), 6.96 (d, *J* = 15.2 Hz, 1H), 2.02 (d, *J* = 6.8 Hz, 3H). ^{13}C NMR (126 MHz, $CDCl_3$) δ ppm 190.37, 145.53, 145.04, 140.20, 136.82, 129.28, 129.09, 128.30, 127.68, 127.44, 127.35, 18.74.

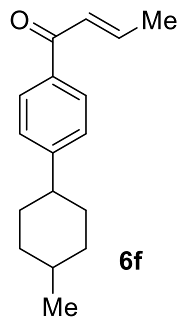

6f

(*E*)-1-(4-(4-methylcyclohexyl)phenyl)but-2-en-1-one ($C_{17}H_{22}O$) (6f): Synthesized using general procedure **GP-E** (with 0.2 mmol of corresponding phenol), purified by silica gel chromatography (10 to 15% EtOAc/hexane), colourless liquid, yield (30 mg) 63% from corresponding phenol. IR (Neat) cm^{-1} : 3055, 2955, 2921, 2850, 1671, 1623, 1607, 1446, 1375, 1298, 1223, 1185, 1078, 1034, 967. ^{1}H NMR (500 MHz, $CDCl_3$) δ ppm 7.86 (d, *J* = 8.2 Hz, 2H), 7.29 (d, *J* = 8.1 Hz, 2H), 7.09 – 6.99 (m, 1H), 6.90 (d, *J* = 15.2 Hz, 1H), 1.99 (d, *J* = 6.6 Hz, 3H), 1.87 – 1.81 (m, 4H), 1.50 - 1.45 (m, 3H), 1.01 – 1.06 (m, 3H), 0.94 (d, *J* = 6.5 Hz, 3H). ^{13}C NMR (126 MHz, $CDCl_3$) δ 190.49, 153.24, 144.47, 135.93, 128.88, 127.76, 127.19, 44.53, 35.62, 34.21, 32.52, 22.77, 18.66. $[M+H]^+$ calculated for $C_{17}H_{22}O$ is 243.1743 and found 243.1749.

3(f). Synthesis of aldehydes and ketones from aniline:

For the synthesis of aldehydes and the ketones from anilines, we followed the following procedure. Here first we have performed the Cbz protection. Then we have oxidized it to the imine ketal and engaged for the reaction. But we observed very small amount of the product along with several undesired by products. The report by the HHH group indicate that, while oxidizing the Cbz-protected anilines, the use of equivalent of silica convert them in quinketal. Therefore we converted all the all the anilines to corresponding quinketal and engaged for reaction. However, here we tried to carried out the reaction in one pot manner both starting from aniline and Cbz-protected aniline but in both the cases, we observed around 10 to 15% yield of our desired products. Therefor we prepared all the quinketals from the anilines and purified by the reported procedure and utilized for our reaction.

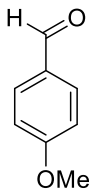

7a

4-methoxybenzaldehyde ($C_8H_8O_2$) (7a): Synthesized using general procedure **GP-C** (with 0.2 mmol of corresponding quinketal), purified by silica gel chromatography (10 to 15% EtOAc/hexane), colourless liquid, yield (25 mg) 92% from corresponding quinketal. All the data found to be matched with compound **2a**. For data and the spectrum please see the details of compound **2a**.

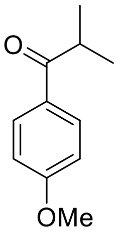

7b

1-(4-methoxyphenyl)-2-methylpropan-1-one ($C_{11}H_{14}O_2$) (7b): Synthesized using general procedure **GP-D** (with 0.2 mmol of corresponding quinketal), purified by silica gel chromatography (10 to 15% EtOAc/hexane), colourless liquid, yield (30 mg) 85% from corresponding quinketal. All the data found to be matched with compound **4a**. For data and the spectrum please see the details of compound **4a**.

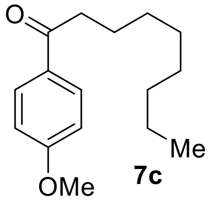

7c

1-(4-methoxyphenyl)nonan-1-one ($C_{16}H_{24}O_2$) (7c): Synthesized using general procedure **GP-D** (with 0.2 mmol of corresponding quinketal), purified by silica gel chromatography (10 to 15% EtOAc/hexane), colourless liquid, yield (42 mg) 86% from corresponding quinketal. All the data found to be matched with compound **4e**. For data and the spectrum please see the details of compound **4e**.

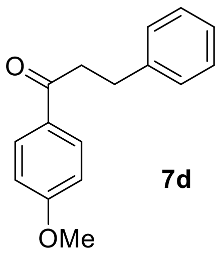

7d

1-(4-methoxyphenyl)-3-phenylpropan-1-one ($C_{16}H_{16}O_2$) (7d): Synthesized using general procedure **GP-D** (with 0.2 mmol of corresponding quinketal), purified by silica gel chromatography (20 to 25% EtOAc/hexane), colourless liquid, yield (45 mg) 95% from corresponding quinketal. All the data found to be matched with compound **3a**. For data and the spectrum please see the details of compound **3a**.

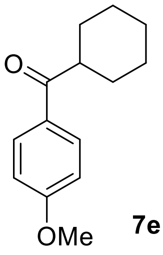

7e

cyclohexyl(4-methoxyphenyl)methanone ($C_{14}H_{18}O_2$) (7e): Synthesized using general procedure **GP-D** (with 0.2 mmol of corresponding quinketal), purified by silica gel chromatography (10 to

15% EtOAc/hexane), colourless liquid, yield (34 mg) 80% from corresponding quinketal. All the data found to be matched with compound **4f**. For data and the spectrum please see the details of compound **4f**.

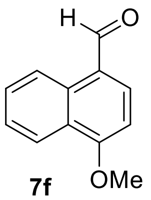

7f

4-methoxy-1-naphthaldehyde ($C_{12}H_{10}O_2$) (7f): Synthesized using general procedure **GP-C** (with 0.2 mmol of corresponding quinketal), purified by silica gel chromatography (10 to 15% EtOAc/hexane), colourless liquid, yield (23 mg) 69% from corresponding quinketal. All the data found to be matched with compound **2m**. For data and the spectrum please see the details of compound **2m**.

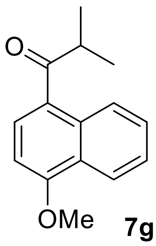

7g

1-(4-methoxynaphthalen-1-yl)-2-methylpropan-1-one ($C_{15}H_{16}O_2$) (7g): Synthesized using general procedure **GP-D** (with 0.2 mmol of corresponding quinketal), purified by silica gel chromatography (10 to 15% EtOAc/hexane), colourless liquid, yield (32 mg) 71% from corresponding quinketal. All the data found to be matched with compound **4d**. For data and the spectrum please see the details of compound **4d**.

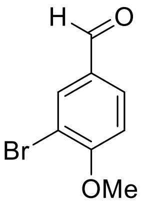

7h

3-bromo-4-methoxybenzaldehyde ($C_8H_7BrO_2$) (7h): [ref-7] Synthesized using general procedure **GP-C** (with 0.2 mmol of corresponding quinketal), purified by silica gel chromatography (10 to 15% EtOAc/hexane), colourless liquid, yield (26 mg) 62% from corresponding quinketal. ^{1}H NMR (400 MHz, $CDCl_3$) δ ppm 9.85 (s, 1H), 8.08 (s, 1H), 7.82 (d, *J* = 8.3 Hz, 1H), 7.02 (d, *J* = 8.3 Hz, 1H), 3.99 (s, 2H). ^{13}C NMR (126 MHz, $CDCl_3$) δ ppm 189.61, 160.80, 134.71, 131.25, 130.94, 112.80, 111.70, 56.73.

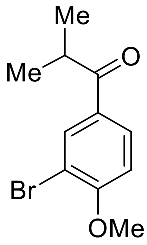

7i

1-(3-bromo-4-methoxyphenyl)-2-methylpropan-1-one ($C_{11}H_{13}BrO_2$) (7i): [ref-7] Synthesized using general procedure **GP-D** (with 0.2 mmol of corresponding quinketal), purified by silica gel chromatography (10 to 15% EtOAc/hexane), colourless liquid, yield (35 mg) 69% from corresponding quinketal. ^{1}H NMR (400 MHz, $CDCl_3$) δ ppm 8.16 (s, 1H), 7.92 (d, *J* = 7.3 Hz, 1H), 6.93 (d, *J* = 8.5

Hz, 1H), 3.96 (s, 3H), 3.47 – 3.43 (m, 1H), 1.20 – 1.18 (m, 6H). ^{13}C NMR (126 MHz, $CDCl_3$) δ ppm 202.06, 159.51, 133.97, 130.35, 129.54, 112.10, 111.31, 56.57, 35.19, 19.30.

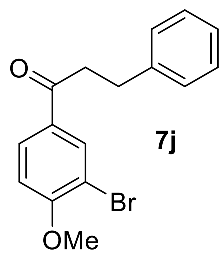

7j

1-(3-bromo-4-methoxyphenyl)-3-phenylpropan-1-one ($C_{16}H_{15}BrO_2$) (7j): Synthesized using general procedure **GP-D** (with 0.2 mmol of corresponding quinketal), purified by silica gel chromatography (10 to 15% EtOAc/hexane), colourless liquid, yield (41 mg) 64% from corresponding quinketal. All the data found to be matched with compound **3g**. For data and the spectrum please see the details of compound **3g**.

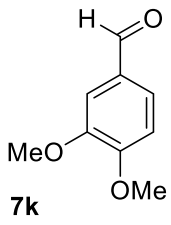

7k

3,4-dimethoxybenzaldehyde ($C_9H_{10}O_3$) (7k): [ref-7] Synthesized using general procedure **GP-C** (with 0.2 mmol of corresponding quinketal), purified by silica gel chromatography (10 to 15% EtOAc/hexane), colourless liquid, yield (21 mg) 64% from corresponding quinketal. ^{1}H NMR (400 MHz, $CDCl_3$) δ ppm 9.45 (s, 1H), 7.05 (d, J = 8.2 Hz, 1H), 6.98 (s, 1H), 6.59 (d, J = 8.2 Hz, 1H), 3.56 (s, 3H), 3.52 (s, 3H). ^{13}C NMR (101 MHz, $CDCl_3$) δ ppm 190.00, 153.67, 148.77, 129.35, 125.86, 109.75, 108.19, 55.28, 55.02.

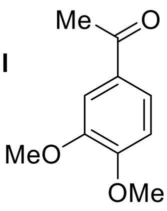

7l

1-(3,4-dimethoxyphenyl)ethan-1-one ($C_{10}H_{12}O_3$) (7l): [ref-7] Synthesized using general procedure **GP-E** (with 0.2 mmol of corresponding quinketal), purified by silica gel chromatography (10 to 15% EtOAc/hexane), colourless liquid, yield (25 mg) 70% from corresponding quinketal. ^{1}H NMR (500 MHz, $CDCl_3$) δ ppm 7.42 (d, J = 8.4 Hz, 1H), 7.38 (s, 1H), 6.75 (d, J = 8.4 Hz, 1H), 3.80 (s, 3H), 3.78 (s, 3H), 2.42 (s, 3H). ^{13}C NMR (126 MHz, $CDCl_3$) δ ppm 196.50, 190.62, 153.17, 148.84, 130.32, 123.10, 109.90, 77.42, 55.85, 55.76, 25.95.

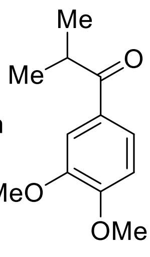

7m

1-(3,4-dimethoxyphenyl)-2-methylpropan-1-one ($C_{12}H_{16}O_3$) (7m): [ref-7] Synthesized using general procedure **GP-D** (with 0.2 mmol of corresponding quinketal), purified by silica gel

chromatography (10 to 15% EtOAc/hexane), colourless liquid, yield (27 mg) 66% from corresponding quinketal. ^{1}H NMR (500 MHz, $CDCl_3$) δ ppm 7.47 (d, *J* = 8.4 Hz, 1H), 7.43 (s, 1H), 6.78 (d, *J* = 8.2 Hz, 1H), 3.81 (s, 3H), 3.80 (s, 3H), 3.44 – 3.38 (m, 1H), 1.09 (s, 3H), 1.08 (s, 3H). ^{13}C NMR (126 MHz, $CDCl_3$) δ ppm 202.86, 152.97, 148.99, 129.14, 122.52, 110.55, 109.96, 55.82, 55.74, 34.60, 19.24.

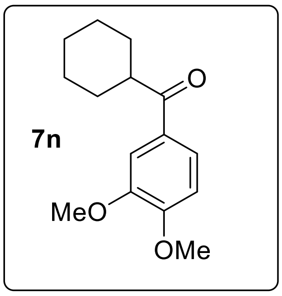

Cyclohexyl(3,4-dimethoxyphenyl)methanone ($C_{15}H_{20}O_3$) (7n): **ref-7** Synthesized using general procedure **GP-D** (with 0.2 mmol of corresponding quinketal), purified by silica gel chromatography (10 to 15% EtOAc/hexane), colourless liquid, yield (33 mg) 67% from corresponding quinketal. ^{1}H NMR (400 MHz, $CDCl_3$) δ ppm 7.47 (d, *J* = 8.4 Hz, 1H), 7.42 (s, 1H), 6.77 (d, *J* = 8.3 Hz, 1H), 3.81 (s, 3H), 3.15 – 3.10 (m, 1H), 1.73 (t, *J* = 11.4 Hz, 4H), 1.61 (d, *J* = 12.3 Hz, 1H), 1.43 – 1.34 (m, 2H), 1.33 – 1.20 (m, 2H), 1.18 – 1.11 (m, 1H). ^{13}C NMR (101 MHz, $CDCl_3$) δ ppm 202.08, 152.78, 148.85, 129.09, 122.37, 110.25, 109.72, 55.70, 55.61, 44.84, 29.44, 25.72, 25.62.

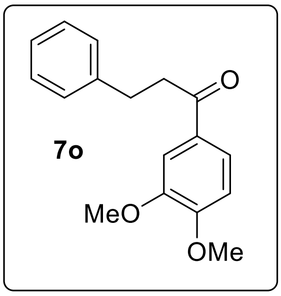

1-(3,4-dimethoxyphenyl)-3-phenylpropan-1-one ($C_{17}H_{18}O_3$) (7o): **ref-7** Synthesized using general procedure **GP-D** (with 0.2 mmol of corresponding quinketal), purified by silica gel chromatography (10 to 15% EtOAc/hexane), colourless liquid, yield (43 mg) 80% from corresponding quinketal. ^{1}H NMR (500 MHz,) δ ppm 7.53 – 7.51 (m, 1H), 7.48 (d, *J* = 1.3 Hz, 1H), 7.24 – 7.19 (m, 4H), 7.15 (t, *J* = 6.9 Hz, 1H), 6.81 (d, *J* = 8.4 Hz, 1H), 3.87 (s, 3H), 3.86 (s, 3H), 3.21 – 3.18 (m, 2H), 3.02 – 2.99 (m, 2H). ^{13}C NMR (126 MHz, $CDCl_3$) δ ppm 197.82, 153.26, 149.02, 141.39, 130.10, 128.46, 128.39, 128.34, 128.29, 126.05, 122.62, 55.97, 55.90, 39.89, 30.42.

4. Bioactive molecules functionalization:

Here we have functionalized several bioactive molecules both in directly to the phenolic core and the side chain of a bioactive molecules. For the side chain functionalization, we first attached the bioactive core with 4-hydroxy phenyl acetic acid and the resulting phenols were subjected to the carbonylation.

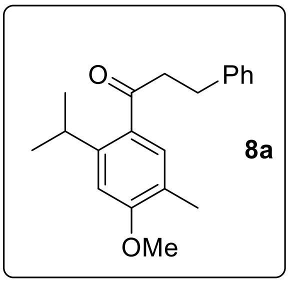

1-(2-isopropyl-4-methoxy-5-methylphenyl)-3-phenylpropan-1-one ($C_{20}H_{24}O_2$) (8a): Synthesized using general procedure **GP-D** (with 0.2 mmol of corresponding phenol), purified by silica gel chromatography (10 to 15% EtOAc/hexane), colourless liquid, yield (41 mg) 65% from corresponding phenol. IR (Neat) cm^{-1} : 3066, 3023, 2962, 2932, 2875, 1709, 1677, 1610, 1565, 1504, 1454, 1357, 1313, 1257, 1152, 1118, 1065, 946. ^1H NMR (400 MHz, $CDCl_3$) δ ppm 7.51 (s, 1H), 7.24 – 7.14 (m, 5H), 6.64 (s, 1H), 3.81 (s, 3H), 3.28 – 3.14 (m, 3H), 3.03 – 2.97 (m, 2H), 2.51 (s, 3H), 1.17 (brs, 3H), 1.16 (brs, 3H). ^{13}C NMR (101 MHz, $CDCl_3$) δ ppm 201.28, 158.92, 141.49, 139.00, 133.80, 129.43, 128.50, 128.40, 127.51, 125.95, 113.62, 55.28, 42.61, 30.70, 26.46, 22.47, 22.11. $[M+Na]^+$ calculated for $C_{20}H_{24}O_2$ is 319.1669 and found 319.1668.

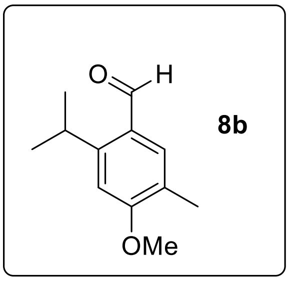

2-isopropyl-4-methoxy-5-methylbenzaldehyde ($C_{12}H_{16}O_2$) (8b): [ref-8] Synthesized using general procedure **GP-C** (with 0.2 mmol of corresponding phenol), purified by silica gel chromatography (10 to 15% EtOAc/hexane), colourless liquid, yield (23 mg) 61% from corresponding phenol. ^1H NMR (400 MHz, $CDCl_3$) δ ppm 10.15 (s, 1H), 7.66 (s, 1H), 6.67 (s, 1H), 3.90 (s, 3H), 3.26 (m, 1H), 2.65 (s, 3H), 1.23 (brs, 3H), 1.21 (brs, 3H). ^{13}C NMR (101 MHz, $CDCl_3$) δ ppm 191.42, 161.08, 140.97, 135.14, 129.88, 127.34, 113.01, 55.59, 26.45, 22.48, 19.44.

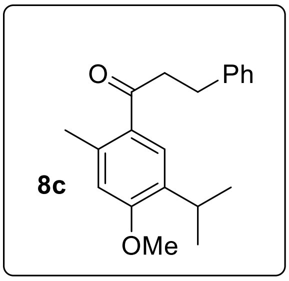

1-(5-isopropyl-4-methoxy-2-methylphenyl)-3-phenylpropan-1-one ($C_{20}H_{24}O_2$) (8c): Synthesized using general procedure **GP-D** (with 0.2 mmol of corresponding phenol), purified by silica gel chromatography (10 to 15% EtOAc/hexane), colourless liquid, yield (44 mg) 69% from corresponding phenol. IR (Neat) cm^{-1} : 3061, 3022, 2960, 2934, 2871, 1706, 1670, 1612, 1561, 1501, 1453, 1356, 1312, 1256, 1150, 1114, 1061, 945. ^1H NMR (400 MHz, $CDCl_3$) δ 7.35 – 7.34 (m, 6H), 6.88 (d, J = 3.4 Hz, 1H), 3.92 (d, J = 3.4 Hz, 3H), 3.64 (ddd, J = 10.3, 6.8, 3.3 Hz, 1H), 3.23 (td, J = 7.7, 3.5 Hz, 2H), 3.08 (td, J = 7.7, 3.3 Hz, 2H), 2.24 - 2.22 (m, 3H), 1.27 – 1.26 (m, 6H). ^{13}C NMR (101 MHz, $CDCl_3$) δ ppm 203.15, 160.08, 149.24, 141.52, 130.98, 130.13, 128.58,

128.55, 126.16, 123.51, 107.67, 55.36, 43.88, 30.78, 29.27, 24.34, 15.90. [M+Na]$^+$ calculated for $C_{20}H_{24}O_2$ is 319.1669 and found 319.1668.

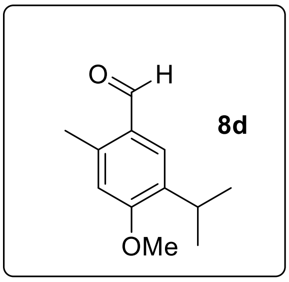

5-isopropyl-4-methoxy-2-methylbenzaldehyde ($C_{12}H_{16}O_2$) (8d): [ref-8] Synthesized using general procedure **GP-C** (with 0.2 mmol of corresponding phenol), purified by silica gel chromatography (10 to 15% EtOAc/hexane), colourless liquid, yield (24 mg) 62% from corresponding phenol. ^{1}H NMR (400 MHz, $CDCl_3$) δ ppm 10.20 (s, 1H), 7.60 (s, 1H), 6.82 (s, 1H), 4.05 – 3.95 (m, 1H), 3.93 (s, 3H), 2.20 (s, 3H), 1.30 (d, J = 2.3 Hz, 3H), 1.29 (d, J = 2.2 Hz, 3H). ^{13}C NMR (101 MHz, $CDCl_3$) δ ppm 190.87, 162.56, 152.35, 134.00, 125.94, 124.69, 106.86, 55.52, 27.68, 24.05, 15.67.

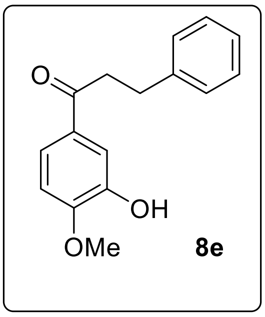

1-(3-hydroxy-4-methoxyphenyl)-3-phenylpropan-1-one ($C_{16}H_{16}O_3$) (8e): Synthesized using general procedure **GP-D** (with 0.2 mmol of corresponding phenol), purified by silica gel chromatography (15 to 25% EtOAc/hexane), colourless liquid, yield (36 mg) 70% from corresponding phenol. IR (Neat) cm^{-1} : 3502, 3023, 2923, 2851, 1676, 1607, 1583, 1512, 1454, 1364, 1275, 1211, 1171, 1011, 956. ^{1}H NMR (400 MHz, $CDCl_3$) δ ppm 7.60 – 7.53 (m, 2H), 7.32 – 7.28 (m, 2H), 7.24 – 7.18 (m, 3H), 6.87 (d, J = 8.4 Hz, 1H), 6.08 (brs, 1H), 3.92 (s, 3H), 3.23 (dd, J = 10.0, 5.5 Hz, 2H), 3.09 – 2.99 (m, 2H). ^{13}C NMR (101 MHz, $CDCl_3$) δ ppm 198.24, 150.83, 145.54, 141.40, 130.55, 128.51, 128.43, 126.09, 121.58, 114.29, 109.99, 56.06, 40.11, 30.35. [M+H]$^+$ calculated for $C_{16}H_{16}O_3$ is 257.1172 and found 257.1184.

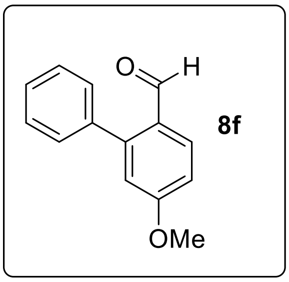

5-methoxy-[1,1'-biphenyl]-2-carbaldehyde ($C_{14}H_{12}O_2$) (8f): [ref-8] Synthesized using general procedure **GP-C** (with 0.2 mmol of corresponding phenol), purified by silica gel chromatography (10 to 15% EtOAc/hexane), colourless liquid, yield (28 mg) 66% from corresponding phenol. ^{1}H NMR (400 MHz, $CDCl_3$) δ ppm 9.84 (s, 1H), 8.03 (d, J = 8.7 Hz, 1H), 7.49 – 7.43 (m, 3H), 7.39 (dd, J = 5.7, 1.5 Hz, 2H), 7.00 (d, J = 8.2 Hz, 1H), 6.88 (d, J = 1.6 Hz, 1H), 3.90 (s, 3H). ^{13}C NMR (126 MHz, $CDCl_3$) δ ppm 159.16, 142.68, 141.05, 138.08, 129.94, 128.73, 128.29, 127.24, 126.46, 115.25, 113.98, 55.55.

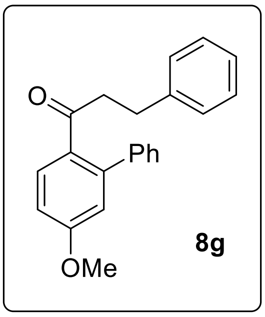

1-(5-methoxy-[1,1'-biphenyl]-2-yl)-3-phenylpropan-1-one ($C_{22}H_{20}O_2$) (8g): Synthesized using general procedure **GP-D** (with 0.2 mmol of corresponding phenol), purified by silica gel chromatography (15 to 25% EtOAc/hexane), colourless liquid, yield (44 mg) 70% from corresponding phenol. IR (Neat) cm^{-1} : 3026, 2926, 2852, 1681, 1603, 1564, 1486,1455, 1329, 1297, 1216, 1180, 1130, 1078, 1037, 1018, 978, 923. ^1H NMR (400 MHz, $CDCl_3$) δ ppm 7.52 (d, J = 8.5 Hz, 1H), 7.40 (brs, 3H), 7.31 (d, J = 3.5 Hz, 2H), 7.18 – 7.12 (m, 3H), 6.91 - 6.86 (m, 4H), 3.87 (s, 3H), 2.79 – 2.69 (m, 2H), 2.55 (dd, J = 10.3, 5.0 Hz, 2H). ^{13}C NMR (101 MHz, $CDCl_3$) δ ppm 205.23, 161.68, 143.07, 141.09, 133.38, 130.52, 129.71, 128.85, 128.81, 128.40, 128.10, 126.02, 115.88, 113.07, 100.28, 55.62, 44.38, 31.02. $[M+NH_4]^+$ calculated for $C_{22}H_{20}O_2$ is 334.1802 and found 334.1805.

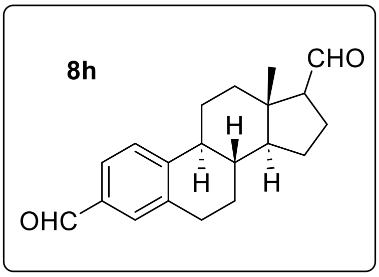

(8*S*,9*S*,13*S*,14*S*)-13-methyl-7,8,9,11,12,13,14,15,16,17-decahydro-6*H*-cyclopenta[a]phenanthrene-3,17-dicarbaldehyde ($C_{20}H_{24}O_2$) (8h): Synthesized using general procedure **GP-F** (with 0.2 mmol of corresponding phenol and 2.5 equiv. of lithiated germinal B(pin)), purified by silica gel chromatography (15 to 25% EtOAc/hexane), colourless liquid, yield (32 mg) 55% from corresponding phenol. IR (Neat) cm^{-1} : 3049, 2929, 2859, 2722, 1739, 1698, 1605, 1572, 1454, 1382, 1334, 1228, 1136, 1009, 908. ^1H NMR (500 MHz, $CDCl_3$) δ ppm 9.95 (s, 1H), 9.82 (d, J = 1.6 Hz, 1H), 7.64 (d, J = 7.7 Hz, 1H), 7.59 (s, 1H), 7.45 (d, J = 7.9 Hz, 1H), 2.99 – 2.91 (m, 3H), 2.41 – 2.35 (m, 2H), 2.20 – 2.17 (m, 2H), 1.97 – 1.95 (m, 1H), 1.91 – 1.88 (m, 1H), 1.85 – 1.78 (m, 1H), 1.72 – 1.63 (m, 2H), 1.50 – 1.43 (m, 2H), 1.39 – 1.36 (m, 2H), 0.81 (s, 3H). ^{13}C NMR (126 MHz, $CDCl_3$) δ ppm 204.69, 192.40, 147.60, 137.79, 134.41, 130.42, 127.25, 126.14, 62.96, 55.59, 45.05, 44.87, 38.50, 37.81, 29.41, 27.50, 25.95, 24.71, 21.31, 13.99. $[M+H]^+$ calculated for $C_{20}H_{24}O_2$ is 297.1849 and found 297.1852.

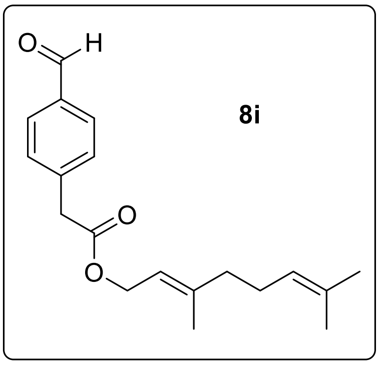

(*E*)-3,7-dimethylocta-2,6-dien-1-yl 2-(4-formylphenyl)acetate ($C_{19}H_{24}O_3$) (8i): Synthesized using general procedure **GP-F** (with 0.2 mmol of corresponding

phenol), purified by silica gel chromatography (15 to 25% EtOAc/hexane), colourless liquid, yield (34 mg) 58% from corresponding phenol. We have also synthesized it from its corresponding quinketal which resulted the desire product with 65% isolated yield calculated from the quinketal. IR (Neat) cm^{-1} : 3062, 2976, 2922, 2855, 2735, 1745, 1738, 1692, 1609, 1581, 1444, 1378, 1305, 1215, 1158, 1098, 973. ^{1}H NMR (500 MHz, $CDCl_3$) δ ppm 9.99 (s, 1H), 7.84 (d, *J* = 8.1 Hz, 2H), 7.45 (d, *J* = 8.1 Hz, 2H), 5.32 (dt, *J* = 10.8, 8.0 Hz, 1H), 5.06 (dd, *J* = 9.3, 3.8 Hz, 1H), 4.63 (d, *J* = 7.1 Hz, 2H), 3.70 (s, 2H), 2.10 – 2.06 (m, 2H), 2.05 – 2.00 (m, 2H), 1.68 (brs, 6H), 1.59 (s, 3H). ^{13}C NMR (126 MHz, $CDCl_3$) δ ppm 191.91, 170.70, 142.97, 141.18, 135.53, 131.99, 130.17, 130.07, 123.81, 118.07, 62.24, 41.61, 39.64, 26.43, 25.78, 17.80, 16.60. $[M+Na]^+$ calculated for $C_{19}H_{24}O_3$ is 323.1618 and found 323.1617.

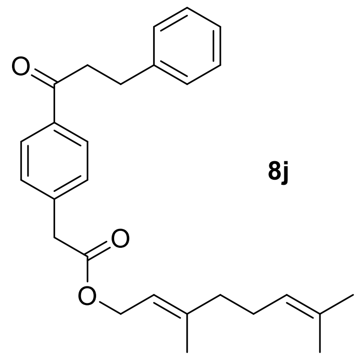

8j

(*E*)-3,7-dimethylocta-2,6-dien-1-yl 2-(4-(3-phenylpropanoyl)phenyl)acetate ($C_{27}H_{32}O_3$) (8j): Synthesized using general procedure **GP-G** (with 0.2 mmol of corresponding phenol), purified by silica gel chromatography (15 to 25% EtOAc/hexane), colourless liquid, yield (50 mg) 62% from corresponding phenol. We have also synthesized it from its corresponding quinketal which resulted the desire product with 68% isolated yield calculated from the quinketal.IR (Neat) cm^{-1} : 3071, 3028, 2967, 2925, 2864, 1742, 1702, 1693, 1608, 1577, 1496, 1453, 1376, 1305, 1244, 1154, 979. ^{1}H NMR (500 MHz, $CDCl_3$) δ ppm 7.91 (d, *J* = 8.2 Hz, 2H), 7.37 (d, *J* = 8.1 Hz, 2H), 7.29 (t, *J* = 7.5 Hz, 2H), 7.24 (d, *J* = 8.9 Hz, 2H), 7.20 (t, *J* = 7.2 Hz, 1H), 5.32 (t, *J* = 7.0 Hz, 1H), 5.07 (t, *J* = 6.2 Hz, 1H), 4.62 (d, *J* = 7.1 Hz, 2H), 3.67 (s, 2H), 3.31 – 3.22 (m, 2H), 3.06 (t, *J* = 7.7 Hz, 2H), 2.10 – 2.06 (m, 2H), 2.05 – 2.01 (m, 2H), 1.68 (brs, 6H), 1.59 (s, 3H). ^{13}C NMR (126 MHz, $CDCl_3$) δ ppm 198.90, 170.92, 142.89, 141.43, 139.63, 135.90, 132.00, 129.73, 128.67, 128.56, 128.48, 126.28, 123.84, 118.14, 62.19, 41.45, 40.57, 39.66, 30.30, 26.45, 25.80, 17.82, 16.62. $[M+H]^+$ calculated for $C_{27}H_{32}O_3$ is 405.2424 and found 405.2429.

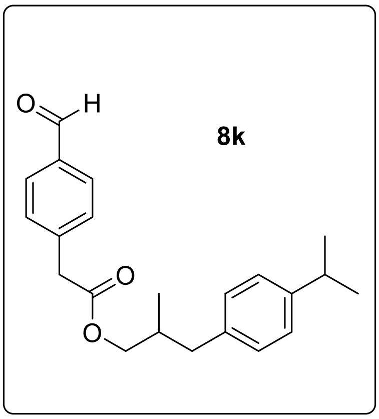

3-(4-isopropylphenyl)-2-methylpropyl 2-(4-formylphenyl)acetate ($C_{22}H_{26}O_3$) (8k): Synthesized using general procedure **GP-F** (with 0.2 mmol of corresponding phenol), purified by silica gel chromatography (15 to 25% EtOAc/hexane), colourless liquid, yield (39 mg) 59% from corresponding phenol. We have also synthesized it from its corresponding quinketal which resulted the desire product with 65% isolated yield calculated from the quinketal. IR (Neat) cm^{-1} : 3045, 3027, 2960, 2920, 2865, 2736, 1747, 1696, 1608, 1571, 1512, 1462, 1426, 1387, 1259, 1220, 1169, 1006. ^1H NMR (400 MHz, $CDCl_3$) δ ppm 10.00 (s, 1H), 7.86 (d, *J* = 7.9 Hz, 2H), 7.47 (d, *J* = 7.8 Hz, 2H), 7.12 (d, *J* = 7.9 Hz, 2H), 7.00 (d, *J* = 7.8 Hz, 2H), 3.96 (ddd, *J* = 25.5, 10.8, 6.2 Hz, 2H), 3.70 (s, 2H), 2.87 (dt, *J* = 13.8, 7.0 Hz, 1H), 2.59 (dd, *J* = 13.5, 6.6 Hz, 1H), 2.39 (dd, *J* = 13.5, 7.6 Hz, 1H), 2.07 (dq, *J* = 13.2, 6.5 Hz, 1H), 1.24 (s, 3H), 1.22 (s, 3H), 0.88 (d, *J* = 6.7 Hz, 3H). ^{13}C NMR (126 MHz, $CDCl_3$) δ ppm 191.90, 170.67, 146.79, 141.18, 137.18, 135.59, 130.22, 130.12, 129.12, 126.49, 69.53, 41.74, 39.49, 34.69, 33.83, 24.17, 16.87. $[M+NH_4]^+$ calculated for $C_{22}H_{26}O_3$ is 356.2220 and found 356.2225.

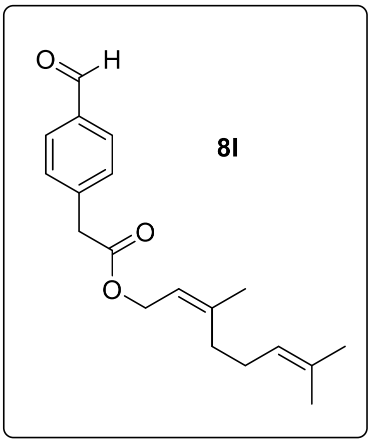

(*Z*)-3,7-dimethylocta-2,6-dien-1-yl 2-(4-formylphenyl)acetate ($C_{19}H_{24}O_3$) (8l): Synthesized using general procedure **GP-F** (with 0.2 mmol of corresponding phenol), purified by silica gel chromatography (15 to 25% EtOAc/hexane), colourless liquid, yield (33 mg) 56% from corresponding phenol. We have also synthesized it from its corresponding quinketal which resulted the desire product with 61% isolated yield calculated from the quinketal. IR (Neat) cm^{-1} : 3069, 2971, 2921, 2845, 2732, 1741, 1732, 1690, 1608, 1574, 1440, 1371, 1304, 1210, 1152, 1091, 970. ^1H NMR (400 MHz, $CDCl_3$) δ ppm 10.00 (s, 1H), 7.84 (d, *J* = 7.8 Hz, 2H), 7.46 (d, *J* = 7.7 Hz, 2H), 5.34 (s, 1H), 5.07 (s, 1H), 4.60 (d, *J* = 7.2 Hz, 2H), 3.70 (s, 2H), 2.08 – 2.05 (m, 4H), 1.76 (s, 3H), 1.67 (s, 3H), 1.58 (s, 3H). ^{13}C NMR (126 MHz, $CDCl_3$) δ ppm 191.92, 170.71, 143.29, 141.17, 135.58, 132.37, 130.20, 130.10, 123.69, 118.96, 62.02, 41.65, 32.36, 26.81, 25.81, 23.64, 17.79. $[M+Na]^+$ calculated for $C_{19}H_{24}O_3$ is 301.1618 and found 323.1626.

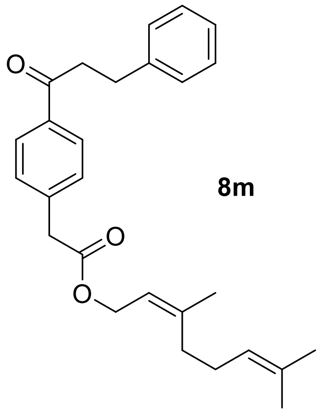

8m

(*Z*)-3,7-dimethylocta-2,6-dien-1-yl 2-(4-(3-phenylpropanoyl)phenyl)acetate ($C_{27}H_{32}O_3$) (8m): Synthesized using general procedure **GP-G** (with 0.2 mmol of corresponding phenol), purified by silica gel chromatography (15 to 25% EtOAc/hexane), colourless liquid, yield (48 mg) 60% from corresponding phenol. We have also synthesized it from its corresponding quinketal which resulted the desire product with 66% isolated yield calculated from the quinketal. IR (Neat) cm^{-1} : 3074, 3029, 2961, 2924, 2863, 1741, 1705, 1691, 1607, 1572, 1491, 1450, 1373, 1306, 1241, 1152, 969. ^{1}H NMR (400 MHz, $CDCl_3$) δ 7.94 (d, *J* = 7.8 Hz, 2H), 7.39 (d, *J* = 8.0 Hz, 2H), 7.34 – 7.30 (m, 3H), 7.27 – 7.21 (m, 2H), 5.38 – 5.34 (m, 1H), 5.10 (brs, 1H), 4.61 (d, *J* = 7.5 Hz, 2H), 3.69 (brs, 2H), 3.31 (t, *J* = 7.7 Hz, 2H), 3.10 – 3.07 (m, 2H), 2.14 – 2.07 (m, 4H), 1.79 (d, *J* = 7.1 Hz, 3H), 1.70 (d, *J* = 5.6 Hz, 3H), 1.62 (brs, 3H).^{13}C NMR (126 MHz, $CDCl_3$) δ ppm 198.94, 170.93, 143.19, 141.44, 139.61, 135.91, 132.36, 129.75, 128.69, 128.57, 128.49, 126.29, 123.69, 118.99, 61.95, 41.46, 40.58, 32.34, 30.31, 26.80, 25.82, 23.65, 17.79. $[M+H]^+$ calculated for $C_{27}H_{32}O_3$ is 405.2424 and found 405.2416.

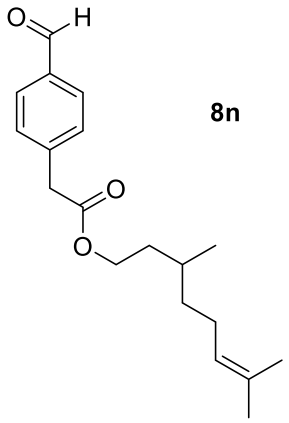

8n

3,7-dimethyloct-6-en-1-yl 2-(4-formylphenyl)acetate ($C_{19}H_{26}O_3$) (8n): Synthesized using general procedure **GP-F** (with 0.2 mmol of corresponding phenol), purified by silica gel chromatography (15 to 25% EtOAc/hexane), colourless liquid, yield (33 mg) 55% from corresponding phenol. We have also synthesized it from its corresponding quinketal which resulted the desire product with 60% isolated yield calculated from the quinketal. IR (Neat) cm^{-1} : 3024, 2958, 2925, 2854, 2739, 1735, 1720, 1608, 1581, 1458, 1428, 1385, 1336, 1311, 1255, 1215, 1169, 983. ^{1}H NMR (400 MHz, $CDCl_3$) δ ppm 10.00 (s, 1H), 7.85 (d, *J* = 7.9 Hz, 2H), 7.45 (d, *J* = 7.9 Hz, 2H), 5.13 – 5.02 (m, 1H), 4.19 – 4.09 (m, 2H), 3.69 (s, 2H), 1.99 – 1.87 (m, 2H), 1.68 (s, 3H), 1.59 (s, 3H), 1.51 – 1.38 (m, 3H), 1.34 – 1.29 (m, 1H), 1.18 – 1.11 (m, 1H), 0.88 (d, *J* = 6.3 Hz, 3H). ^{13}C NMR (101 MHz, $CDCl_3$) δ ppm 191.95, 170.76, 141.16, 135.49, 131.53, 130.16, 130.09, 124.60, 63.94, 41.70, 37.05, 35.49, 29.62, 25.85, 25.51, 19.49, 17.79. $[M+H]^+$ calculated for $C_{19}H_{26}O_3$ is 303.1955 calculated and found 303.1955.

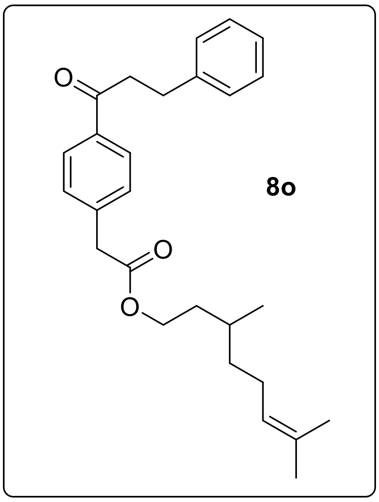

3,7-dimethyloct-6-en-1-yl 2-(4-(3-phenylpropanoyl)phenyl)acetate ($C_{27}H_{34}O_3$) (8o): Synthesized using general procedure **GP-G** (with 0.2 mmol of corresponding phenol), purified by silica gel chromatography (15 to 25% EtOAc/hexane), colourless liquid, yield (49 mg) 61% from corresponding phenol. We have also synthesized it from its corresponding quinketal which resulted the desire product with 68% isolated yield calculated from the quinketal. IR (Neat) cm^{-1} : 3075, 2967, 2925, 2854, 1743, 1708, 1694, 1609, 1513, 1454, 1296, 1247, 1192, 1159, 1033, 980. ^{1}H NMR (400 MHz, $CDCl_3$) δ ppm 7.92 (d, J = 8.0 Hz, 2H), 7.37 (d, J = 8.1 Hz, 2H), 7.34 – 7.27 (m, 3H), 7.26 – 7.18 (m, 2H), 5.06 (brs, 1H), 4.15 – 4.10 (m, 2H), 3.66 (s, 2H), 3.29 (t, J = 7.6 Hz, 2H), 3.17 – 2.97 (m, 2H), 2.02 – 1.88 (m, 2H), 1.68 (s, 3H), 1.59 (s, 3H), 1.53 – 1.38 (m, 3H), 1.30 – 1.28 (m, 1H), 1.19 – 1.16 (m, 1H), 0.88 (d, J = 6.4 Hz, 3H). ^{13}C NMR (126 MHz, $CDCl_3$) δ ppm 198.90, 170.97, 141.44, 139.64, 135.92, 131.53, 129.73, 128.69, 128.57, 128.50, 126.30, 124.66, 63.90, 41.56, 40.58, 37.09, 35.54, 29.68, 25.84, 25.55, 19.53, 17.79. $[M+H]^+$ calculated for $C_{27}H_{34}O_3$ is 407.2581 found 407.2585.

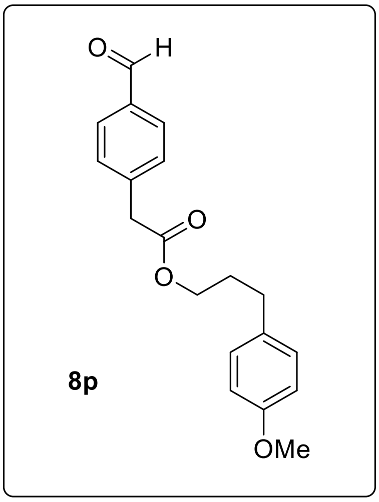

3-(4-methoxyphenyl)propyl 2-(4-formylphenyl)acetate ($C_{19}H_{20}O_4$) (8p): Synthesized using general procedure **GP-F** (with 0.2 mmol of corresponding phenol), purified by silica gel chromatography (15 to 25% EtOAc/hexane), colourless liquid, yield (39 mg) 63% from corresponding phenol. We have also synthesized it from its corresponding quinketal which resulted the desire product with 70% isolated yield calculated from the quinketal. IR (Neat) cm^{-1} : 3042, 2951, 2930, 2837, 2734, 1742, 1736, 1693, 1609, 1585, 1516, 1465, 1387, 1305, 1253, 1170, 1034, 832. ^{1}H NMR (400 MHz, $CDCl_3$) δ ppm 10.01 (s, 1H), 7.86 (d, J = 8.0 Hz, 2H), 7.46 (d, J = 7.7 Hz, 2H), 7.03 (d, J = 8.3 Hz, 2H), 6.81 (d, J = 8.4 Hz, 2H), 4.11 (t, J = 6.5 Hz, 2H), 3.78 (s, 3H), 3.70 (s, 2H), 2.59 – 2.55 (m, 2H), 1.94 – 1.89 (m, 2H). ^{13}C NMR (126 MHz, $CDCl_3$) δ ppm 191.91, 170.69, 158.15, 141.15, 135.59, 133.14, 130.19, 130.13, 129.40, 114.07, 64.66, 55.42, 41.69, 31.28, 30.46. $[M+H]^+$ calculated for $C_{19}H_{20}O_2$ is 313.1434 and found 313.1437.

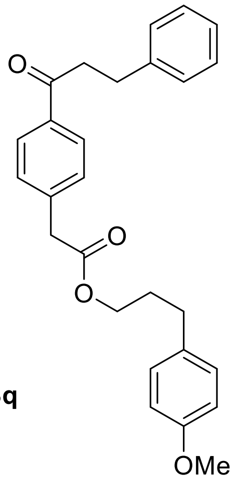

8q

3-(4-methoxyphenyl)propyl 2-(4-(3-phenylpropanoyl)phenyl)acetate ($C_{27}H_{28}O_4$) (8q): Synthesized using general procedure **GP-G** (with 0.2 mmol of corresponding phenol), purified by silica gel chromatography (15 to 25% EtOAc/hexane), colourless liquid, yield (56 mg) 68% from corresponding phenol. We have also synthesized it from its corresponding quinketal which resulted the desire product with 71% isolated yield calculated from the quinketal. IR (Neat) cm^{-1} : 3062, 3028, 2959, 2933, 2855, 1745, 1706, 1683, 1609, 1525, 1454, 1309, 1244, 1179, 1162, 1033, 980. ^{1}H NMR (400 MHz, $CDCl_3$) δ ppm 7.96 (d, *J* = 8.2 Hz, 2H), 7.40 (d, *J* = 8.2 Hz, 2H), 7.34 – 7.30 (m, 3H), 7.25 – 7.21 (m, 2H), 7.05 (d, *J* = 8.4 Hz, 2H), 6.83 (d, *J* = 8.4 Hz, 2H), 4.12 (t, *J* = 6.5 Hz, 2H), 3.80 (s, 3H), 3.70 (s, 2H), 3.31 (t, *J* = 7.7 Hz, 2H), 3.11 – 3.07 (m, 2H), 2.61 – 2.57 (m, 2H), 1.96 – 1.89 (m, 2H). ^{13}C NMR (126 MHz, $CDCl_3$) δ ppm 198.89, 170.89, 158.13, 141.42, 139.60, 135.96, 133.18, 129.74, 129.40, 128.68, 128.56, 128.52, 126.29, 114.06, 64.57, 55.42, 41.53, 40.59, 31.28, 30.47, 30.30. $[M+H]^+$ calculated for $C_{27}H_{28}O_4$ is 417.2060 and found 417.2066.

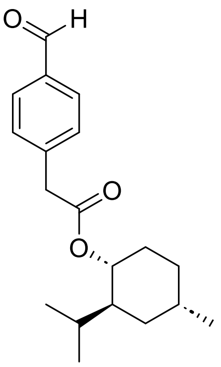

8r

(±)(1*R*,2*S*,4*S*)-2-isopropyl-4-methylcyclohexyl 2-(4-formylphenyl)acetate ($C_{19}H_{26}O_3$) (8r): Synthesized using general procedure **GP-F** (with 0.2 mmol of corresponding phenol), purified by silica gel chromatography (15 to 25% EtOAc/hexane), colourless liquid, yield (32 mg) 54% from corresponding phenol. We have also synthesized it from its corresponding quinketal which resulted the desire product with 64% isolated yield calculated from the quinketal. IR (Neat) cm^{-1} : 3058, 2959, 2924, 2868, 2838, 2732, 1745, 1732, 1688, 1608, 1577, 1456, 1370, 1318, 1255, 1227, 1169, 1145, 1010, 967. ^{1}H NMR (400 MHz, $CDCl_3$) δ ppm 10.00 (s, 1H), 7.84 (d, *J* = 8.0 Hz, 2H), 7.45 (d, *J* = 7.9 Hz, 2H), 5.19 (s, 1H), 3.69 (s, 2H), 1.90 (dd, *J* = 14.1, 2.2 Hz, 1H), 1.69 – 1.68 (m, 2H), 1.49 – 1.38 (m, 1H), 1.25 – 1.18 (m, 2H), 1.05 – 1.01 (m, 1H), 0.97 – 0.87 (m, 2H), 0.82 – 0.79 (m, 6H), 0.76 (d, *J* = 6.6 Hz, 3H). ^{13}C NMR (101 MHz, $CDCl_3$) δ ppm 192.00, 170.10, 141.52, 135.42, 130.16, 130.04, 72.38, 46.84, 42.31, 39.16,

34.82, 29.31, 26.66, 25.19, 22.24, 21.02, 20.76. $[M+H]^+$ calculated for $C_{19}H_{26}O_3$ is 303.1955 and found 303.1967.

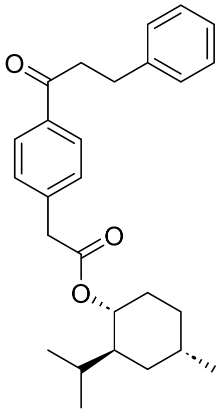

8s

(±)(1*R*,2*S*,4*S*)-2-isopropyl-4-methylcyclohexyl 2-(4-(3-phenylpropanoyl)phenyl)acetate ($C_{27}H_{34}O_3$) (8s): Synthesized using general procedure **GP-G** (with 0.2 mmol of corresponding phenol), purified by silica gel chromatography (15 to 25% EtOAc/hexane), colourless liquid, yield (47 mg) 58% from corresponding phenol. We have also synthesized it from its corresponding quinketal which resulted the desire product with 69% isolated yield calculated from the quinketal. IR (Neat) cm^{-1} : 3062, 3028, 2950, 2924, 2868, 2842, 1741, 1704, 1689, 1608, 1495, 1454, 1426, 1369, 1256, 1171, 1149, 1024, 968. ^1H NMR (500 MHz, $CDCl_3$) δ ppm 7.92 (d, J = 8.2 Hz, 2H), 7.37 (d, J = 8.2 Hz, 2H), 7.33 – 7.28 (m, 2H), 7.24 – 7.19 (m, 3H), 5.19 (s, 1H), 3.66 (s, 2H), 3.29 (dd, J = 9.8, 5.5 Hz, 2H), 3.08 – 3.05 (m, 2H), 1.91 – 1.89 (m, 1H), 1.72 – 1.67 (m, 2H), 1.48 – 1.40 (m, 1H), 1.25 – 1.20 (m, 2H), 1.04 – 0.98 (m, 1H), 0.97 – 0.86 (m, 2H), 0.83 (d, J = 6.9 Hz, 3H), 0.81 (d, J = 6.8 Hz, 3H), 0.78 (d, J = 6.7 Hz, 3H). ^{13}C NMR (126 MHz, $CDCl_3$) δ ppm 198.93, 170.30, 141.42, 139.96, 135.81, 129.71, 128.67, 128.55, 128.41, 126.29, 72.28, 46.90, 42.12, 40.59, 39.21, 34.88, 30.33, 29.32, 26.66, 25.19, 22.24, 21.02, 20.78. $[M+H]^+$ calculated for $C_{27}H_{34}O_3$ is 407.2581 found 407.2585.

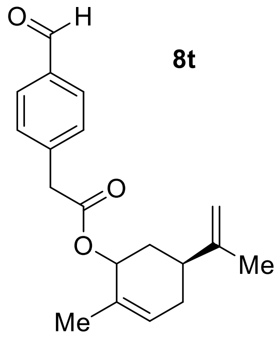

8t

(5*S*)-2-methyl-5-(prop-1-en-2-yl)cyclohex-2-en-1-yl 2-(4-formylphenyl)acetate ($C_{19}H_{22}O_3$) (8t): Synthesized using general procedure **GP-F** (with 0.2 mmol of corresponding phenol), purified by silica gel chromatography (15 to 25% EtOAc/hexane), colourless liquid, yield (31 mg) 52% from corresponding phenol. We have also synthesized it from its corresponding quinketal which resulted the desire product with 63% isolated yield calculated from the quinketal. IR (Neat) cm^{-1} : 3080, 2963, 2918, 2854, 2737, 1735, 1698, 1644, 1607, 1581, 1438, 1350, 1304, 1250, 1212, 1168, 1151, 1018, 952, 911. ^1H NMR (500 MHz, $CDCl_3$) δ ppm 10.00 (s, 1H), 7.84 (d, J = 8.1 Hz, 2H), 7.47 (d, J = 8.0 Hz, 2H), 5.73 (s, 1H), 5.26 (s, 1H), 4.73 (s, 1H), 4.66 (s, 1H), 3.72 (s, 2H), 2.24 – 2.19 (m, 1H), 2.16 (s, 1H), 1.89 - 1.86 (m, 2H), 1.66 (s, 3H), 1.64 –

1.58 (m, 4H). ^{13}C NMR (126 MHz, $CDCl_3$) δ ppm 191.91, 170.51, 148.60, 141.35, 135.55, 130.72, 130.12, 130.08, 128.42, 109.47, 77.41, 42.13, 36.05, 33.84, 30.92, 20.85, 20.69. $[M+H]^+$ calculated for $C_{19}H_{22}O_3$ is 299.1642 found 299.1646.

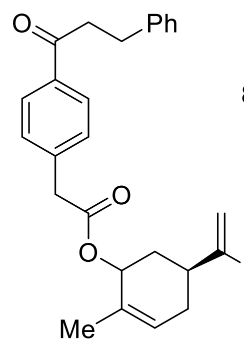

8u

(5*S*)-2-methyl-5-(prop-1-en-2-yl)cyclohex-2-en-1-yl 2-(4-(3-phenylpropanoyl)phenyl)acetate ($C_{27}H_{30}O_3$) (8u): Synthesized using general procedure **GP-G** (with 0.2 mmol of corresponding phenol), purified by silica gel chromatography (15 to 25% EtOAc/hexane), colourless liquid, yield (44 mg) 55% from corresponding phenol. We have also synthesized it from its corresponding quinketal which resulted the desire product with 67% isolated yield calculated from the quinketal. IR (Neat) cm^{-1} : 3088, 3028, 2967, 2920, 2859, 1742, 1706, 1693, 1608, 1496, 1453, 1413, 1361, 1292, 1252, 1151, 1021, 979. ^{1}H NMR (400 MHz, $CDCl_3$) δ ppm 7.92 (d, J = 8.3 Hz, 2H), 7.39 (d, J = 8.2 Hz, 2H), 7.32 – 7.28 (m, 2H), 7.24 – 7.19 (m, 3H), 5.73 (s, 1H), 5.25 (s, 1H), 4.72 (s, 1H), 4.66 (s, 1H), 3.68 (s, 2H), 3.28 (t, J = 7.7 Hz, 2H), 3.06 (t, J = 7.7 Hz, 2H), 2.26 – 2.14 (m, 2H), 1.90 – 1.82 (m, 2H), 1.62 (brs, 3H), 1.58 (brs, 4H). ^{13}C NMR (126 MHz, $CDCl_3$) δ ppm 198.90, 170.73, 148.66, 141.43, 139.79, 135.92, 130.79, 129.63, 128.69, 128.56, 128.52, 128.36, 126.30, 109.44, 71.73, 41.96, 40.58, 36.04, 33.85, 30.93, 30.31, 20.88, 20.71. $[M+H]^+$ calculated for $C_{27}H_{30}O_3$ is 403.2262 found 403.2267.

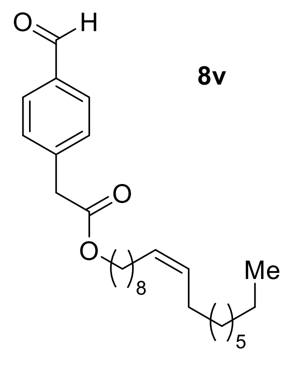

8v

(*Z*)-octadec-9-en-1-yl 2-(4-formylphenyl)acetate ($C_{27}H_{42}O_3$) (8v): Synthesized using general procedure **GP-F** (with 0.2 mmol of corresponding phenol), purified by silica gel chromatography (15 to 25% EtOAc/hexane), colourless liquid, yield (53 mg) 64% from corresponding phenol. We have also synthesized it from its corresponding quinketal which resulted the desire product with 69% isolated yield calculated from the quinketal. IR (Neat) cm^{-1} : 3004, 2928, 2853, 2731, 1739, 1720, 1708, 1609, 1583, 1465, 1387, 1336, 1305, 1252, 1213, 1173, 1004, 960. ^{1}H NMR (400 MHz, $CDCl_3$) δ 10.00 (s, 1H), 7.85 (d, J = 8.2 Hz, 2H), 7.46 (d, J = 8.1 Hz, 2H), 5.39 – 5.33 (m, 2H), 4.09 (t, J = 6.7 Hz, 2H), 3.70 (s, 2H), 2.01- 1.98 (m, 3H), 1.63 – 1.59 (m, 2H), 1.27 (brs, 231H), 0.91 – 0.86 (m, 3H). ^{13}C NMR (126 MHz, $CDCl_3$) δ ppm 191.89, 170.75, 141.20, 135.55, 130.16, 130.08, 129.89, 126.78, 65.50, 41.68, 32.04,

29.90, 29.85, 29.65, 29.52, 29.46, 29.44, 29.31, 29.28, 28.67, 27.36, 27.32, 25.95, 22.81, 14.22. $[M+H]^+$ calculated for $C_{27}H_{42}O_3$ is 415.3207 and found 415.3220.

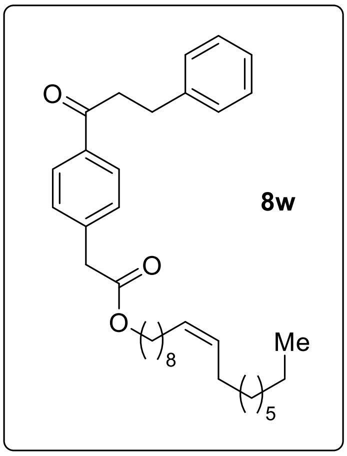

(*Z*)-octadec-9-en-1-yl 2-(4-(3-phenylpropanoyl)phenyl)acetate ($C_{35}H_{50}O_3$) (8w): Synthesized using general procedure **GP-G** (with 0.2 mmol of corresponding phenol), purified by silica gel chromatography (15 to 25% EtOAc/hexane), colourless liquid, yield (71 mg) 69% from corresponding phenol. We have also synthesized it from its corresponding quinketal which resulted the desire product with 72% isolated yield calculated from the quinketal. IR (Neat) cm^{-1} : 3030, 3007, 2928, 2853, 1736, 1686, 1608, 1494, 1454, 1415, 1367, 1334, 1293, 1250, 1179, 1161, 980. ^1H NMR (400 MHz, $CDCl_3$) δ ppm 7.92 (d, J = 8.0 Hz, 2H), 7.37 (d, J = 7.9 Hz, 2H), 7.32 – 7.28 (m, 2H), 7.24 – 7.19 (m, 3H), 5.35 (s, 2H), 4.09 (t, J = 6.6 Hz, 2H), 3.67 (s, 2H), 3.29 (t, J = 7.5 Hz, 2H), 3.06 (t, J = 7.5 Hz, 2H), 2.01 – 2.00 (m, 3H), 1.61 (brs, 2H), 1.27 (brs, 22H), 0.88 (t, J = 6.4 Hz, 3H). ^{13}C NMR (126 MHz, $CDCl_3$) δ ppm 198.88, 170.96, 141.44, 139.66, 135.95, 130.17, 129.91, 129.72, 128.68, 128.56, 128.49, 126.29, 65.44, 41.53, 40.57, 32.05, 30.33, 29.92, 29.87, 29.67, 29.53, 29.48, 29.45, 29.33, 29.30, 28.70, 27.38, 27.34, 25.98, 22.82, 14.22. $[M+NH_4]^+$ calculated for $C_{35}H_{50}O_3$ is 519.3833 and found 519.3856.

5. regioselective acetophenone synthesis:

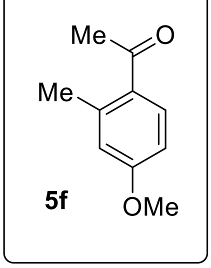

1-(4-methoxy-2-methylphenyl)ethan-1-one ($C_{10}H_{12}O_2$) (5f): [ref-5] Synthesized using general procedure **GP-E** (with 0.2 mmol of corresponding phenol), purified by silica gel chromatography (10 to 20% EtOAc/hexane), colourless liquid, yield (22 mg) 69% from corresponding phenol. ^1H NMR (400 MHz, $CDCl_3$) δ ppm 7.76 (s, 1H), 6.75 (brs, 2H), 3.85 (s, 3H), 2.56 (s, 3H), 2.55 (s, 3H). ^{13}C NMR (126 MHz, $CDCl_3$) δ ppm 199.64, 162.12, 142.36, 132.63, 130.23, 117.65, 110.76, 55.46, 29.22, 22.71.

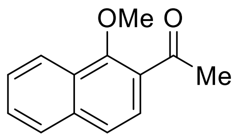

5h

1-(1-methoxynaphthalen-2-yl)ethan-1-one ($C_{13}H_{12}O_2$) (5h): **ref-5** Synthesized using general procedure **GP-E** (with 0.2 mmol of corresponding phenol), purified by silica gel chromatography (10 to 20% EtOAc/hexane), colourless liquid, yield (21 mg) 54% from corresponding phenol. ^{1}H NMR (500 MHz, $CDCl_3$) δ ppm 8.23 (d, *J* = 8.2 Hz, 1H), 7.86 (d, *J* = 7.2 Hz, 1H), 7.74 (d, *J* = 8.6 Hz, 1H), 7.63 (d, *J* = 8.6 Hz, 1H), 7.60 – 7.55 (m, 2H), 4.01 (s, 3H), 2.78 (s, 3H). ^{13}C NMR (126 MHz, $CDCl_3$) δ ppm 200.29, 157.66, 137.10, 128.41, 128.27, 128.24, 128.10, 126.75, 125.68, 124.30, 123.55, 64.00, 30.95.

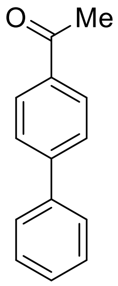

5j

1-([1,1'-biphenyl]-4-yl)ethan-1-one ($C_{14}H_{12}O$) (5j): **ref-5** Synthesized using general procedure **GP-E** (with 0.2 mmol of corresponding phenol), purified by silica gel chromatography (10 to 20% EtOAc/hexane), colourless liquid, yield (24 mg) 63% from corresponding phenol. ^{1}H NMR (400 MHz, $CDCl_3$) δ ppm 8.03 (d, *J* = 3.7 Hz, 2H), 7.68 – 7.63 (m, 4H), 7.47 – 7.40 (m, 3H), 2.64 (s, 3H). ^{13}C NMR (126 MHz, $CDCl_3$) δ ppm 197.84, 145.97, 140.08, 136.10, 129.11, 129.06, 128.38, 127.43, 127.39, 26.76.

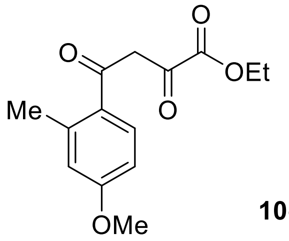

10c

ethyl 4-(4-methoxy-2-methylphenyl)-2,4-dioxobutanoate ($C_{14}H_{16}O_5$) (10c): **ref-10** Synthesized using general procedure **GP-H** (with 0.1 mmol of corresponding acetophenone), purified by silica gel chromatography (20 to 25% EtOAc/hexane), colourless liquid, yield (21 mg) 82% from corresponding acetophenone. ^{1}H NMR (500 MHz, $CDCl_3$) δ ppm 15.30 (s, 1H), 7.70 (d, *J* = 8.3 Hz, 1H), 6.87 – 6.79 (m, 2H), 4.39 (q, *J* = 7.1 Hz, 2H), 3.86 (s, 3H), 2.59 (s, 3H), 1.40 (t, *J* = 7.1 Hz, 3H). ^{13}C NMR (126 MHz, $CDCl_3$) δ ppm 194.91, 166.73, 162.89, 162.71, 142.22, 131.95, 128.44, 117.80, 111.38, 101.42, 62.56, 55.56, 22.17, 14.26.

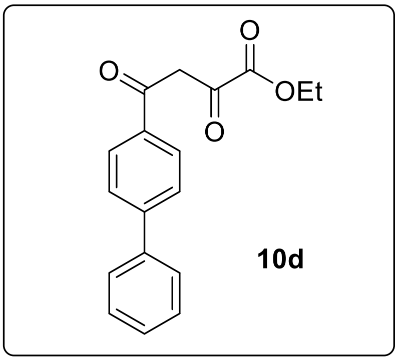

ethyl 4-([1,1'-biphenyl]-4-yl)-2,4-dioxobutanoate ($C_{18}H_{16}O_4$) (10d):[ref-10] Synthesized using general procedure **GP-H** (with 0.1 mmol of corresponding acetophenone), purified by silica gel chromatography (20 to 25% EtOAc/hexane), colourless liquid, yield (47 mg) 80% from corresponding acetophenone. ^{1}H NMR (500 MHz, $CDCl_3$) δ ppm 15.34 (s, 1H), 8.08 (d, *J* = 8.4 Hz, 2H), 7.73 (d, *J* = 8.4 Hz, 2H), 7.65 (d, *J* = 7.3 Hz, 2H), 7.49 (t, *J* = 7.6 Hz, 2H), 7.42 (t, *J* = 7.3 Hz, 1H), 4.42 (q, *J* = 7.1 Hz, 2H), 1.43 (t, *J* = 7.1 Hz, 3H). ^{13}C NMR (126 MHz, $CDCl_3$) δ ppm 190.23, 170.21, 162.45, 146.75, 139.82, 133.82, 129.19, 128.65, 127.68, 127.44, 98.08, 62.75, 14.26.

6. Synthesis and application of photocatalyst:

While working with BINOL, we found that in the step of quinketal formation, an internal cyclazitation occurs with the one β-napthol unit to the other.(H. Zhang, T. Wirth, Chem. Eur. J. 2022, 28, e202200181) Next when this quinketal was subjected to our standered reaction condition, it leads to a photo-active material. However, we have performed the acylation in one-pot starting from BINOL.

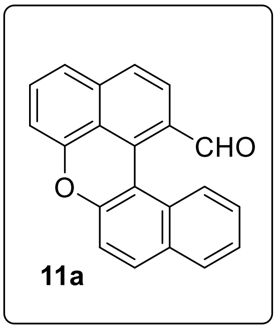

dibenzo[*a,kl*]xanthene-1-carbaldehyde ($C_{21}H_{12}O_2$) (11a): Synthesized using general procedure **GP-C** (with 0.2 mmol of corresponding BINOL), purified by silica gel chromatography (5 to 15% EtOAc/hexane), colourless liquid, yield (33 mg) 56% from corresponding BINOL. IR (Neat) cm^{-1} : 3059, 3019, 2926, 2857, 2742, 1739, 1679, 1627, 1597, 1571, 1512, 1447, 1421, 1375, 1362, 1263, 1229, 1212, 1142, 1045, 985. 928. ^{1}H NMR (400 MHz, $CDCl_3$) δ ppm 9.99 (s, 1H), 8.45 (d, *J* = 8.2 Hz, 1H), 8.01 (d, *J* = 8.7 Hz, 1H), 7.88 (d, *J* = 8.9 Hz, 1H), 7.79 (d, *J* = 8.4 Hz, 1H), 7.62 (d, *J* = 8.7 Hz, 1H), 7.52 (t, *J* = 7.8 Hz, 1H), 7.44 – 7.37 (m, 4H), 7.09 (d, *J* = 7.7 Hz, 1H). ^{13}C NMR (126 MHz, $CDCl_3$) δ ppm 188.91, 153.52, 151.20, 136.49, 132.77, 132.54, 132.21, 130.90, 129.54, 128.33, 127.68, 126.37, 125.50, 125.27, 124.75, 124.39,

121.42, 119.88, 117.07, 113.18, 108.87. $[M+H]^+$ calculated for $C_{21}H_{12}O_2$ is 297.0910 value and found 297.0919.

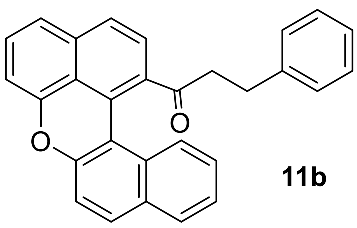

11b

1-(dibenzo[*a,kl*]xanthen-1-yl)-3-phenylpropan-1-one ($C_{29}H_{20}O_2$) (11b): Synthesized using general procedure **GP-D** (with 0.2 mmol of corresponding BINOL), purified by silica gel chromatography (5 to 15% EtOAc/hexane), colourless liquid, yield (51 mg) 64% from corresponding BINOL. IR (Neat) cm^{-1} : 3093, 3064, 3028, 2929, 2860, 2825, 1708, 1667, 1584, 1540, 1497, 1454, 1389, 1257, 1179, 1082, 1012, 843. ^1H NMR (400 MHz, $CDCl_3$) δ ppm 8.22 (d, *J* = 8.3 Hz, 1H), 7.86 (t, *J* = 7.9 Hz, 2H), 7.69 (d, *J* = 8.6 Hz, 1H), 7.63 (d, *J* = 8.6 Hz, 1H), 7.50 – 7.43 (m, 4H), 7.39 (d, *J* = 8.8 Hz, 1H), 7.10 – 6.99 (m, 4H), 6.62 (d, *J* = 7.5 Hz, 2H), 2.76 – 2.65 (m, 1H), 2.55 – 2.52 (m, 1H), 2.35 – 2.27 (m, 1H), 2.05 – 1.97 (m, 1H). ^{13}C NMR (101 MHz, $CDCl_3$) δ ppm 204.66, 153.37, 151.31, 141.00, 135.61, 132.15, 131.99, 131.82, 130.99, 128.67, 128.29, 128.19, 127.83, 127.64, 127.49, 125.84, 125.64, 125.31, 125.11, 122.56, 119.92, 117.60, 115.00, 108.80, 44.03, 30.86. $[M+H]^+$ calculated for $C_{29}H_{20}O_2$ is 401.1536 and found 401.1541.

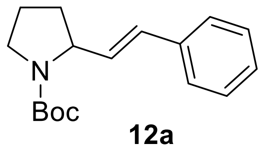

12a

(*E*)-*tert*-butyl 2-styrylpyrrolidine-1-carboxylate ($C_{17}H_{23}NO_2$) (12a): To a dry 20 mL vial equipped with a stir bar was added **11a** (0.003 mmol, 0.01 equiv.), (*E*)-(2-(phenylsulfonyl)vinyl)benzene (73 mg, 0.30 mmol, 1.0 equiv.), Cs_2CO_3 (195mg, 0.60 mmol, 2 equiv.) and the *N*-Boc-α-amino acid (0.60 mmol, 2 equiv.). The vial was sealed and then DMA (5 ml) was added to the vial and the resulting mixture degassed by freeze-pump-thaw under nitrogen (three times). The vial was then placed in sunlight for 8 hour. The reaction mixture was diluted with H_2O and workup by using EtOAc and the organic residue concentrated in vacuo. Purification by flash column chromatography or preparative TLC afforded the (*E*)-*tert*-butyl 2-styrylpyrrolidine-1-carboxylate in (50 mg, 62%, 100:0 *E:Z*). IR (Neat) cm^{-1} : 3011, 2978, 2937, 2875, 2821, 1620, 1503, 1398, 1362, 1264, 1188, 1015, 966. ^1H NMR (500 MHz, $CDCl_3$) δ 7.35 (d, *J* = 7.5 Hz, 2H), 7.30 – 7.26 (m, 2H), 7.21 (t, *J* = 6.7 Hz, 1H), 6.40 (d, *J* = 15.3 Hz, 1H), 6.10 (brs, 1H), 4.40 (brs, 1H), 3.47 (brs, 2H), 2.08 (brs, 1H) 1.95 – 1.89 (m, 1H), 1.86 – 1.83 (m, 1H), 1.82 – 1.76 (m, 1H), 1.43 (s, 9H). ^{13}C NMR (126 MHz, $CDCl_3$) δ 154.73, 137.19, 130.84, 129.52, 128.57, 127.32, 126.37, 79.22, 59.00,

46.41, 32.64, 28.58, 23.19. $[M+H]^+$ calculated for $C_{17}H_{23}NO_2$ is 274.1802 and found 274.1815.

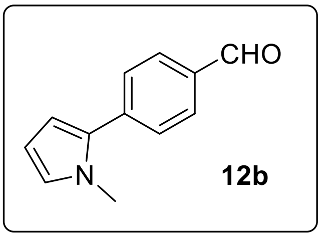

4-(1-methyl-1*H*-pyrrol-2-yl)benzaldehyde ($C_{12}H_{11}NO$) (12b):[ref-10] To a dry 20 mL vial equipped with a stir bar was added **11a** (0.003 mmol, 0.01 equiv.), 4-bromo benzaldehyde (1 equiv) and Cs_2CO_3 (1.2 equiv.). The flask was put under N_2 and 2 mL of DMA were added, followed by *N*-methylpyrrole (5 equiv). The flask was sealed, and three cycles of freeze-pump-thaw were performed before refilling with N_2. The reaction mixture was then placed in sunlight for 8 hour. The reaction mixture was diluted with DCM and filtered over celite. The solution was evaporated to dryness, and the residue purified by flash column chromatography to obtain the desired compound as a yellow oil (22 mg, 60% yield). ^{1}H NMR (500 MHz, $CDCl_3$) δ 10.02 (s, 1H), 7.90 (d, *J* = 7.9 Hz, 2H), 7.57 (d, *J* = 8.1 Hz, 2H), 6.79 (s, 1H), 6.38 (s, 1H), 6.24 – 6.22 (m, 1H), 3.74 (s, 3H). ^{13}C NMR (101 MHz, $CDCl_3$) δ 191.91, 139.39, 134.35, 133.38, 130.12, 128.35, 125.92, 110.87, 108.67, 35.70.

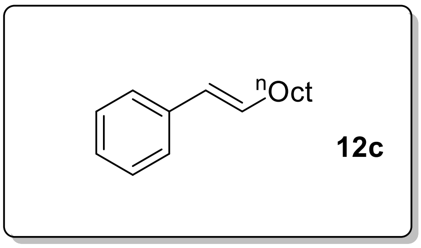

(*E*)-dec-1-en-1-ylbenzene ($C_{16}H_{24}$) (12c):[ref-10] To a dry 20 mL vial equipped with a magnetic stir bar was added **11a** (5 mol%), n-octyl B(pin) (1 equiv), phenyl vinyl sulfone (1 equiv) and 1 equiv of NaO^{t}Bu. The vial was sealed and then DMA (0.1 M w.r.t boronic acid) was added to the vial and the resulting mixture was degassed by freeze-pump-thaw under nitrogen (three times). Then, the vial was placed in sunlight for 8 hour. After that the reaction mixture was diluted with H_2O (4 mL) and workup by using Et_2O (3 X 5 mL). Purification by flash column chromatography to obtain the desired compound as a yellow oil (22 mg) 52% (*E*:*Z* = 100:0). ^{1}H NMR (500 MHz, $CDCl_3$) δ ppm 7.35 (d, *J* = 7.4 Hz, 2H), 7.29 (t, *J* = 7.6 Hz, 2H), 7.19 (t, *J* = 7.3 Hz, 1H), 6.39 (d, *J* = 15.8 Hz, 1H), 6.23 (dt, *J* = 15.7, 6.9 Hz, 1H), 2.21 (td, *J* = 7.8, 1.0 Hz, 2H), 1.52 – 1.44 (m, 2H), 1.39 – 1.27 (m, 10H), 0.90 (t, *J* = 6.9 Hz, 3H). ^{13}C NMR (126 MHz, $CDCl_3$) δ ppm 138.20, 131.42, 129.90, 128.61, 126.88, 126.08, 33.20, 32.06, 29.65, 29.57, 29.44, 29.41, 22.82, 14.22.

7. Crystallographic Data and Structure Refinements:

All the crystals grew as colorless prisms by slow evaporation from pet ether and ethyl acetate. First, the compound was taken in a small glass vial and dissolved in a minimum amount of ethyl acetate. Then dropwise pet ether was added until the solution became faint opaque. Next, the vial was closed with a plastic cap containing a small hole for slow solvent evaporation. After one week the growing crystal was found to be visible and submitted for further characterization. Good quality single crystals of each compounds were sorted out with the help of a polarizing microscope and immersed in paratone oil, which was then mounted on the tip of glass fiber and cemented using epoxy resin. The single-crystal XRD diffraction data were collected at 298 K on a Bruker AXS (D8 Quest System) X-ray diffractometer, equipped with a PHOTON 100 CMOS detector using graphite-monochromated Mo-K_{α} radiation (0.71073 Å). The linear absorption coefficients, scattering factors for the atoms, and the anomalous dispersion corrections were taken from International Tables for X-ray Crystallography. Bruker Apex III software was used for data collection, unit cell measurements, absorption corrections, scaling, and integration.[11] The data were reduced and an empirical absorption correction was applied with the help of SAINTPLUS software and SADABS programs using XPREP, respectively.[12-14] The structures were solved by the direct method using SHELXL-2014 in the WinGx programs. For all the cases, nonhydrogen atoms were refined anisotropically. All other hydrogen atoms were geometrically fixed using the riding atom model and assigned fixed isotropic displacement parameters. The “ACTA” command was used to generate the Crystallographic Information File (CIF). The structural details of all the compounds are presented here. CCDC: 2304923, 2304924 contain the crystallographic data of these compounds 3337, 3562 respectively. Here 3337 and 11a both are same compound. Here 3562 and 11b both are same compound. These data are available from The Cambridge Crystallographic Data Center (CCDC) via

www.ccdc.cam.ac.uk/data_request/cif.

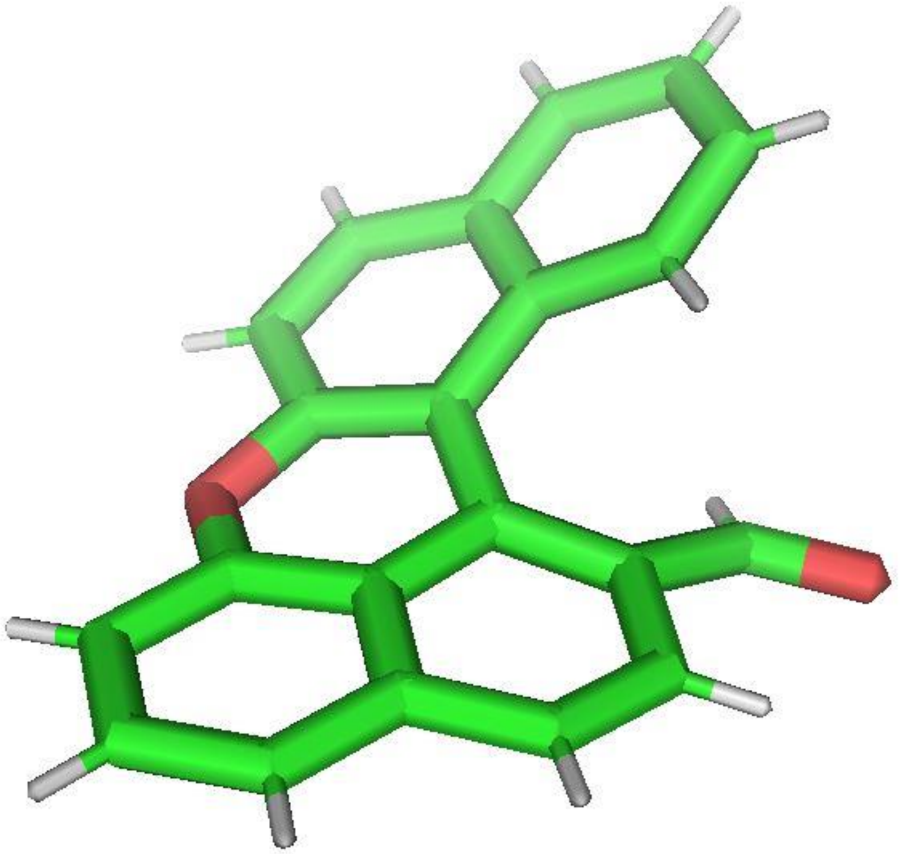

Supplementary Figure 2: Structure of 3337 (11a)

Datablock shelx - ellipsoid plot

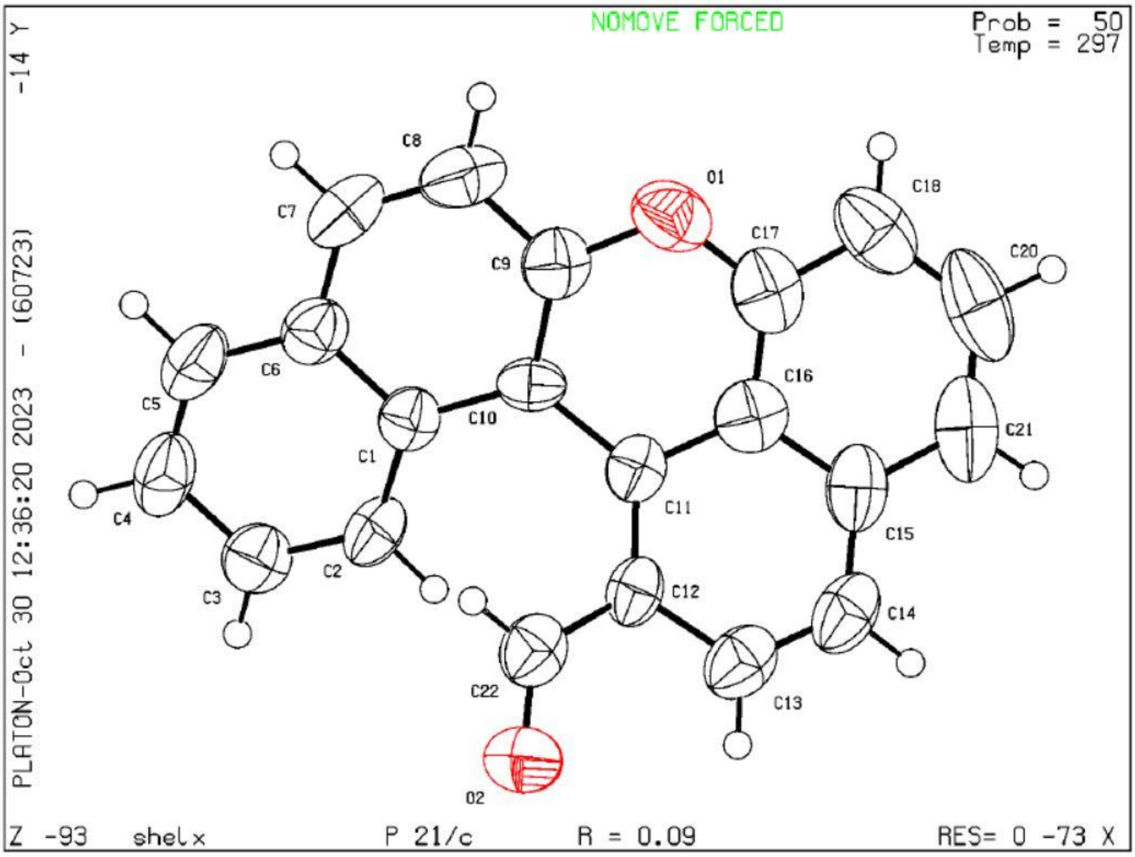

Supplementary Figure 3: Perspective view of 3337 (11a) with 50% thermal ellipsoid probability.

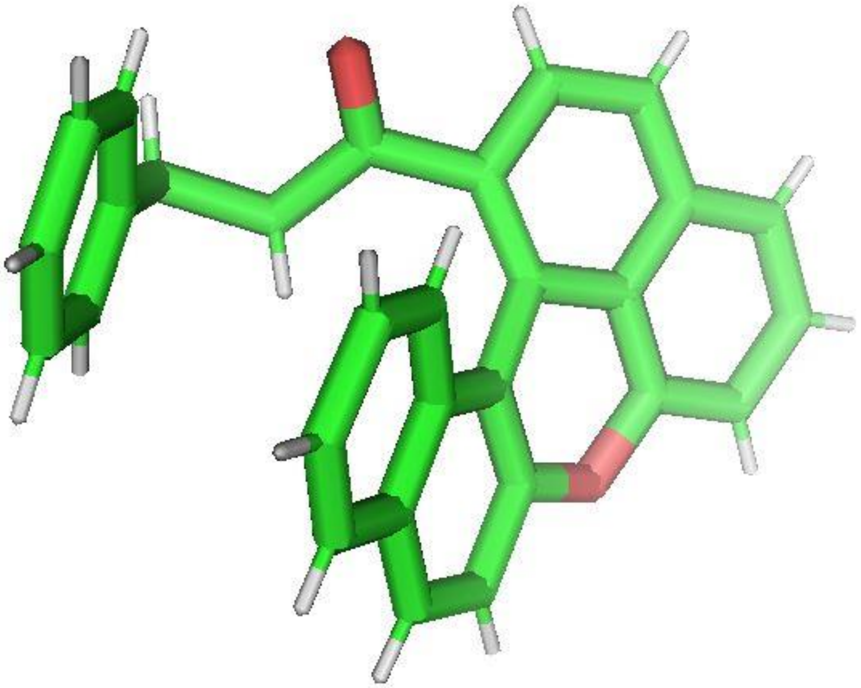

Supplementary Figure 4: Structure of 3562 (11b).

Datablock shelx - ellipsoid plot

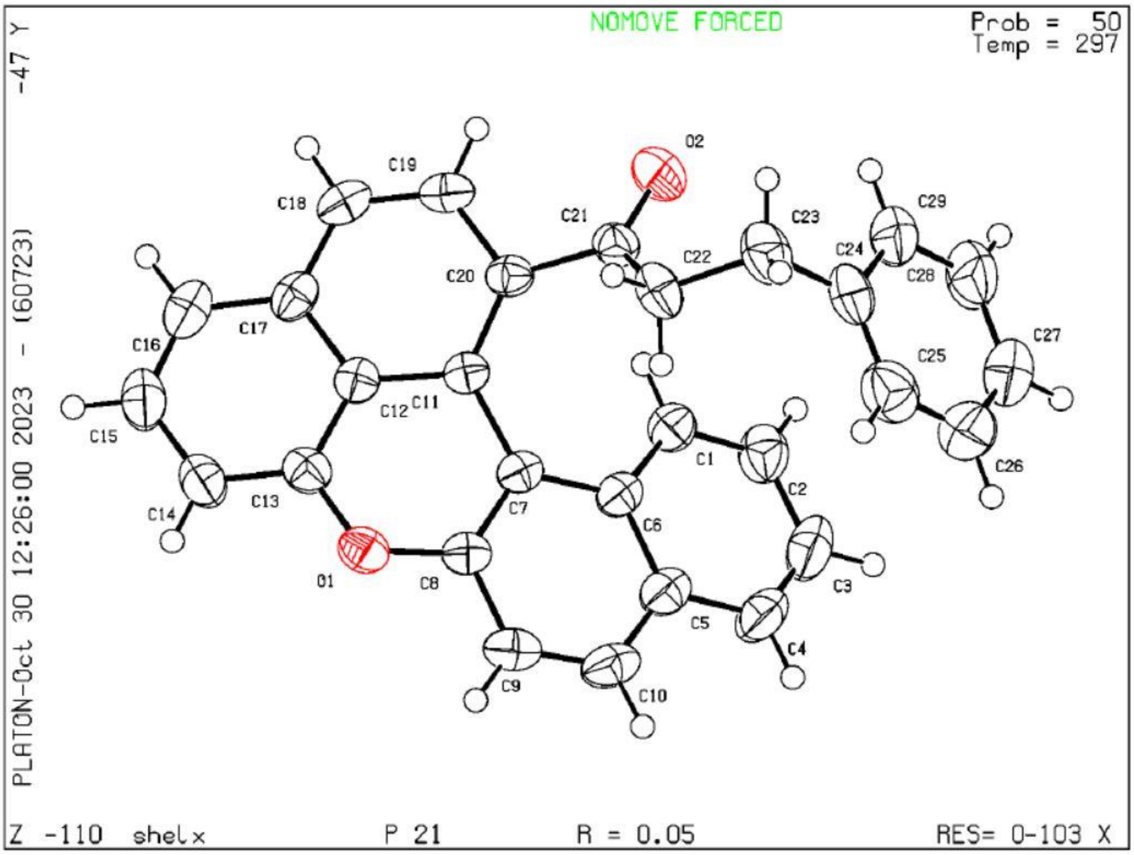

Supplementary Figure 5. Perspective view of 3562 (11b) with 50% thermal ellipsoid probability.

Supplementary Table 1: Crystal data and structure refinements for the molecules 3337 (11a).

Identification code	shelx	
Empirical formula	C21 H12 O2	
Formula weight	296.31	
Temperature	297(2) K	
Wavelength	0.71073 Å	
Crystal system	Monoclinic	
Space group	P 21/c	
Unit cell dimensions	a = 17.477(3) Å	α= 90°.
	b = 4.1941(6) Å	β= 114.434(10)°.
	c = 21.076(4) Å	γ = 90°.
Volume	1406.5(4) $Å^3$	
Z	4	
Density (calculated)	1.399 Mg/m^3	
Absorption coefficient	0.089 mm^{-1}	
F(000)	616	
Crystal size	0.350 x 0.250 x 0.180 mm^3	
Theta range for data collection	2.897 to 23.249°.	
Index ranges	-19<=h<=17, 0<=k<=4, 0<=l<=23	
Reflections collected	2012	
Independent reflections	2012 [R(int) = ?]	
Completeness to theta = 23.249°	99.5 %	
Absorption correction	Semi-empirical from equivalents	
Max. and min. transmission	0.745 and 0.597	
Refinement method	Full-matrix least-squares on F^2	
Data / restraints / parameters	2012 / 0 / 209	
Goodness-of-fit on F^2	1.170	
Final R indices [I>2sigma(I)]	R1 = 0.0927, wR2 = 0.1085	
R indices (all data)	R1 = 0.1762, wR2 = 0.1378	
Extinction coefficient	n/a	
Largest diff. peak and hole	0.213 and -0.232 e.$Å^{-3}$	

Supplementary Table 2. Atomic coordinates (x 10^4) and equivalent isotropic displacement parameters (Å^{2}x 10^3)
for 3337 (11a). U(eq) is defined as one third of the trace of the orthogonalized U^{ij} tensor.

	x	y	z	U(eq)
C(1)	2173(4)	1996(15)	8569(3)	48(2)
C(2)	1394(4)	431(15)	8219(3)	54(2)
C(3)	955(4)	594(15)	7513(3)	61(2)
C(4)	1264(5)	2325(17)	7101(4)	70(2)
C(5)	2035(5)	3708(16)	7413(4)	68(2)
C(6)	2505(4)	3539(15)	8131(4)	57(2)
C(7)	3333(5)	4765(17)	8441(4)	73(2)
C(8)	3827(5)	4355(17)	9126(4)	75(2)
C(9)	3500(4)	2741(16)	9551(4)	58(2)
C(10)	2669(4)	1853(13)	9304(3)	46(2)
C(11)	2371(4)	669(14)	9825(3)	48(2)
C(12)	1556(4)	631(15)	9774(3)	50(2)
C(13)	1365(5)	-1066(16)	10273(4)	66(2)
C(14)	1959(5)	-2589(17)	10817(4)	68(2)
C(15)	2803(5)	-2260(16)	10947(4)	63(2)
C(16)	3004(5)	-551(15)	10452(4)	58(2)
C(17)	3851(5)	103(17)	10632(4)	68(2)
C(18)	4512(5)	-922(19)	11236(4)	82(3)
C(20)	4291(6)	-2800(20)	11695(4)	96(3)
C(21)	3463(6)	-3427(17)	11548(4)	84(3)
C(22)	888(5)	2686(16)	9283(3)	61(2)
O(1)	4079(3)	2158(12)	10220(3)	74(1)
O(2)	145(3)	2557(14)	9177(2)	87(2)

Supplementary Table 3. Bond lengths [Å] and angles [°] for 3337 (11a)

C(1)-C(2)	1.413(7)
C(1)-C(10)	1.430(8)
C(1)-C(6)	1.433(8)
C(2)-C(3)	1.366(7)
C(2)-H(2)	0.9300
C(3)-C(4)	1.401(8)
C(3)-H(3)	0.9300
C(4)-C(5)	1.360(8)
C(4)-H(4)	0.9300
C(5)-C(6)	1.393(8)
C(5)-H(5)	0.9300
C(6)-C(7)	1.416(8)
C(7)-C(8)	1.351(9)
C(7)-H(7)	0.9300
C(8)-C(9)	1.419(8)
C(8)-H(8)	0.9300
C(9)-O(1)	1.375(7)
C(9)-C(10)	1.375(8)
C(10)-C(11)	1.480(8)
C(11)-C(12)	1.385(8)
C(11)-C(16)	1.422(8)
C(12)-C(13)	1.418(8)
C(12)-C(22)	1.476(8)
C(13)-C(14)	1.348(8)
C(13)-H(13)	0.9300
C(14)-C(15)	1.390(8)
C(14)-H(14)	0.9300
C(15)-C(21)	1.402(9)
C(15)-C(16)	1.425(8)
C(16)-C(17)	1.395(9)
C(17)-C(18)	1.386(9)
C(17)-O(1)	1.394(8)
C(18)-C(20)	1.419(10)
C(18)-H(18)	0.9300
C(20)-C(21)	1.374(9)
C(20)-H(20)	0.9300

C(21)-H(21)	0.9300
C(22)-O(2)	1.223(6)
C(22)-H(22)	0.9300

C(2)-C(1)-C(10)	124.6(6)
C(2)-C(1)-C(6)	115.6(6)
C(10)-C(1)-C(6)	119.4(6)
C(3)-C(2)-C(1)	122.1(6)
C(3)-C(2)-H(2)	118.9
C(1)-C(2)-H(2)	118.9
C(2)-C(3)-C(4)	121.0(7)
C(2)-C(3)-H(3)	119.5
C(4)-C(3)-H(3)	119.5
C(5)-C(4)-C(3)	118.7(7)
C(5)-C(4)-H(4)	120.7
C(3)-C(4)-H(4)	120.7
C(4)-C(5)-C(6)	121.7(7)
C(4)-C(5)-H(5)	119.2
C(6)-C(5)-H(5)	119.2
C(5)-C(6)-C(7)	120.9(7)
C(5)-C(6)-C(1)	120.6(6)
C(7)-C(6)-C(1)	118.4(6)
C(8)-C(7)-C(6)	121.8(7)
C(8)-C(7)-H(7)	119.1
C(6)-C(7)-H(7)	119.1
C(7)-C(8)-C(9)	119.3(7)
C(7)-C(8)-H(8)	120.4
C(9)-C(8)-H(8)	120.4
O(1)-C(9)-C(10)	123.6(6)
O(1)-C(9)-C(8)	114.5(6)
C(10)-C(9)-C(8)	121.9(7)
C(9)-C(10)-C(1)	118.1(6)
C(9)-C(10)-C(11)	116.8(6)
C(1)-C(10)-C(11)	125.1(6)
C(12)-C(11)-C(16)	116.5(6)
C(12)-C(11)-C(10)	127.8(6)
C(16)-C(11)-C(10)	115.6(6)
C(11)-C(12)-C(13)	119.9(6)

C(11)-C(12)-C(22)	122.5(6)
C(13)-C(12)-C(22)	116.9(6)
C(14)-C(13)-C(12)	122.3(7)
C(14)-C(13)-H(13)	118.9
C(12)-C(13)-H(13)	118.9
C(13)-C(14)-C(15)	120.1(7)
C(13)-C(14)-H(14)	119.9
C(15)-C(14)-H(14)	119.9
C(14)-C(15)-C(21)	123.6(8)
C(14)-C(15)-C(16)	117.9(7)
C(21)-C(15)-C(16)	118.4(8)
C(17)-C(16)-C(11)	120.8(7)
C(17)-C(16)-C(15)	117.1(7)
C(11)-C(16)-C(15)	121.9(7)
C(18)-C(17)-O(1)	114.9(8)
C(18)-C(17)-C(16)	125.2(8)
O(1)-C(17)-C(16)	119.7(7)
C(17)-C(18)-C(20)	116.1(8)
C(17)-C(18)-H(18)	121.9
C(20)-C(18)-H(18)	121.9
C(21)-C(20)-C(18)	120.6(8)
C(21)-C(20)-H(20)	119.7
C(18)-C(20)-H(20)	119.7
C(20)-C(21)-C(15)	122.3(8)
C(20)-C(21)-H(21)	118.8
C(15)-C(21)-H(21)	118.8
O(2)-C(22)-C(12)	125.1(7)
O(2)-C(22)-H(22)	117.5
C(12)-C(22)-H(22)	117.5
C(9)-O(1)-C(17)	118.1(6)

Symmetry transformations used to generate equivalent atoms:

Supplementary Table 4. Anisotropic displacement parameters ($Å^2 x 10^3$)for 3337 (11a). The anisotropic displacement factor exponent takes the form: $-2\pi^2[h^2a^{*2}U^{11} + ... + 2 h k a^* b^* U^{12}]$

	U^{11}	U^{22}	U^{33}	U^{23}	U^{13}	U^{12}
C(1)	53(4)	47(4)	49(4)	0(4)	24(4)	4(4)
C(2)	74(5)	51(4)	50(4)	-4(4)	38(4)	-6(4)
C(3)	63(5)	65(5)	58(5)	-9(4)	30(4)	2(4)
C(4)	90(6)	78(5)	55(5)	11(5)	44(5)	25(5)
C(5)	85(6)	73(5)	66(6)	12(4)	50(5)	10(5)
C(6)	61(5)	59(5)	59(5)	1(4)	33(4)	6(4)
C(7)	90(6)	71(5)	80(6)	2(5)	59(5)	-10(5)
C(8)	67(5)	84(6)	90(6)	-17(5)	49(5)	-16(4)
C(9)	67(5)	57(5)	54(5)	-13(4)	29(4)	0(4)
C(10)	40(4)	38(4)	60(5)	-6(3)	21(4)	-8(3)
C(11)	61(4)	40(4)	45(4)	-5(3)	24(4)	-3(3)
C(12)	66(5)	46(4)	41(4)	-3(4)	27(4)	-1(4)
C(13)	83(6)	65(5)	62(5)	-15(4)	42(5)	-15(4)
C(14)	99(6)	61(5)	56(5)	-11(4)	45(5)	-18(5)
C(15)	94(6)	48(5)	47(5)	-3(4)	28(5)	7(5)
C(16)	71(5)	49(4)	61(5)	-18(4)	33(5)	1(4)
C(17)	85(6)	60(5)	55(5)	-17(4)	25(5)	11(5)
C(18)	72(6)	86(6)	69(6)	-25(5)	10(6)	15(5)
C(20)	119(8)	81(7)	51(6)	-16(5)	-1(6)	34(6)
C(21)	130(8)	59(5)	52(5)	4(4)	26(6)	15(6)
C(22)	74(5)	59(5)	54(5)	-10(4)	31(4)	-7(5)
O(1)	58(3)	88(4)	68(3)	-18(3)	19(3)	-4(3)
O(2)	57(3)	129(4)	77(4)	3(4)	28(3)	-8(4)

Supplementary Table 5. Hydrogen coordinates (x 10^4) and isotropic displacement parameters ($Å^2$x 10^3) for 3337 (11a)

	x	y	z	U(eq)
H(2)	1173	-748	8478	65
H(3)	442	-458	7302	73
H(4)	949	2523	6623	84
H(5)	2253	4795	7140	82
H(7)	3543	5883	8166	87
H(8)	4375	5123	9316	90
H(13)	811	-1130	10222	79
H(14)	1805	-3863	11106	81
H(18)	5067	-409	11335	99
H(20)	4711	-3611	12100	115
H(21)	3337	-4670	11857	101
H(22)	1043	4195	9035	73

Supplementary Table 6. Torsion angles [°] for 3337 (11a)

C(10)-C(1)-C(2)-C(3)	-178.5(6)
C(6)-C(1)-C(2)-C(3)	-5.0(9)
C(1)-C(2)-C(3)-C(4)	0.4(10)
C(2)-C(3)-C(4)-C(5)	3.4(10)
C(3)-C(4)-C(5)-C(6)	-2.3(10)
C(4)-C(5)-C(6)-C(7)	174.8(6)
C(4)-C(5)-C(6)-C(1)	-2.5(10)
C(2)-C(1)-C(6)-C(5)	6.1(9)
C(10)-C(1)-C(6)-C(5)	179.9(6)
C(2)-C(1)-C(6)-C(7)	-171.4(6)
C(10)-C(1)-C(6)-C(7)	2.5(9)
C(5)-C(6)-C(7)-C(8)	-173.6(7)
C(1)-C(6)-C(7)-C(8)	3.8(11)
C(6)-C(7)-C(8)-C(9)	-2.0(11)
C(7)-C(8)-C(9)-O(1)	173.5(7)
C(7)-C(8)-C(9)-C(10)	-6.5(11)
O(1)-C(9)-C(10)-C(1)	-167.4(6)
C(8)-C(9)-C(10)-C(1)	12.6(9)
O(1)-C(9)-C(10)-C(11)	9.8(9)
C(8)-C(9)-C(10)-C(11)	-170.2(6)
C(2)-C(1)-C(10)-C(9)	162.8(6)
C(6)-C(1)-C(10)-C(9)	-10.5(9)
C(2)-C(1)-C(10)-C(11)	-14.0(10)
C(6)-C(1)-C(10)-C(11)	172.7(6)
C(9)-C(10)-C(11)-C(12)	156.7(6)
C(1)-C(10)-C(11)-C(12)	-26.4(10)
C(9)-C(10)-C(11)-C(16)	-22.8(8)
C(1)-C(10)-C(11)-C(16)	154.1(6)
C(16)-C(11)-C(12)-C(13)	-10.9(9)
C(10)-C(11)-C(12)-C(13)	169.6(6)
C(16)-C(11)-C(12)-C(22)	159.6(5)
C(10)-C(11)-C(12)-C(22)	-19.9(10)
C(11)-C(12)-C(13)-C(14)	2.2(10)
C(22)-C(12)-C(13)-C(14)	-168.8(6)
C(12)-C(13)-C(14)-C(15)	6.8(10)
C(13)-C(14)-C(15)-C(21)	171.7(6)

C(13)-C(14)-C(15)-C(16)	-6.2(10)
C(12)-C(11)-C(16)-C(17)	-163.6(6)
C(10)-C(11)-C(16)-C(17)	16.0(8)
C(12)-C(11)-C(16)-C(15)	11.4(9)
C(10)-C(11)-C(16)-C(15)	-169.1(6)
C(14)-C(15)-C(16)-C(17)	172.1(6)
C(21)-C(15)-C(16)-C(17)	-5.9(9)
C(14)-C(15)-C(16)-C(11)	-3.0(9)
C(21)-C(15)-C(16)-C(11)	179.0(6)
C(11)-C(16)-C(17)-C(18)	178.7(6)
C(15)-C(16)-C(17)-C(18)	3.6(10)
C(11)-C(16)-C(17)-O(1)	4.6(9)
C(15)-C(16)-C(17)-O(1)	-170.6(6)
O(1)-C(17)-C(18)-C(20)	174.9(6)
C(16)-C(17)-C(18)-C(20)	0.5(11)
C(17)-C(18)-C(20)-C(21)	-2.2(11)
C(18)-C(20)-C(21)-C(15)	-0.2(12)
C(14)-C(15)-C(21)-C(20)	-173.5(8)
C(16)-C(15)-C(21)-C(20)	4.4(11)
C(11)-C(12)-C(22)-O(2)	173.6(6)
C(13)-C(12)-C(22)-O(2)	-15.7(10)
C(10)-C(9)-O(1)-C(17)	11.2(9)
C(8)-C(9)-O(1)-C(17)	-168.8(6)
C(18)-C(17)-O(1)-C(9)	166.6(6)
C(16)-C(17)-O(1)-C(9)	-18.7(9)

Symmetry transformations used to generate equivalent atoms:

Supplementary Table 7: Crystal data and structure refinements for the molecules 3562.

Identification code	shelx	
Empirical formula	C29 H20 O2	
Formula weight	400.45	
Temperature	297(2) K	
Wavelength	0.71073 Å	
Crystal system	Monoclinic	
Space group	P 21	
Unit cell dimensions	a = 6.2038(9) Å	α= 90°.
	b = 10.9918(14) Å	β= 93.428(4)°.
	c = 14.756(2) Å	γ = 90°.
Volume	1004.4(2) Å^3	
Z	2	
Density (calculated)	1.324 Mg/m^3	
Absorption coefficient	0.082 mm^{-1}	
F(000)	420	
Crystal size	0.350 x 0.250 x 0.150 mm^3	
Theta range for data collection	2.312 to 27.881°.	
Index ranges	-8<=h<=8, 0<=k<=14, 0<=l<=19	
Reflections collected	2506	
Independent reflections	2506 [R(int) = ?]	
Completeness to theta = 25.242°	99.7 %	
Absorption correction	Semi-empirical from equivalents	
Max. and min. transmission	0.745 and 0.546	
Refinement method	Full-matrix least-squares on F^2	
Data / restraints / parameters	2506 / 1 / 281	
Goodness-of-fit on F^2	1.151	
Final R indices [I>2sigma(I)]	R1 = 0.0490, wR2 = 0.0966	
R indices (all data)	R1 = 0.0701, wR2 = 0.1066	
Absolute structure parameter	-0.6(17)	
Extinction coefficient	n/a	
Largest diff. peak and hole	0.156 and -0.161 e.Å^{-3}	

Supplementary Table 8. Atomic coordinates (x 10^4) and equivalent isotropic displacement parameters ($Å^2$x 10^3)
for 3562. U(eq) is defined as one third of the trace of the orthogonalized U^{ij} tensor.

	x	y	z	U(eq)
C(1)	3561(6)	3904(3)	7531(2)	41(1)
C(2)	3525(7)	3295(4)	8336(2)	53(1)
C(3)	1680(8)	2631(4)	8551(3)	62(1)
C(4)	-63(7)	2564(4)	7942(3)	57(1)
C(5)	-45(6)	3149(3)	7091(2)	44(1)
C(6)	1781(5)	3867(3)	6878(2)	36(1)
C(7)	1790(5)	4444(3)	5999(2)	33(1)
C(8)	230(5)	4067(3)	5352(2)	35(1)
C(9)	-1605(6)	3376(3)	5564(3)	44(1)
C(10)	-1747(6)	2968(3)	6423(3)	48(1)
C(11)	3472(5)	5283(3)	5704(2)	32(1)
C(12)	3826(5)	5235(3)	4754(2)	33(1)
C(13)	2276(5)	4711(3)	4135(2)	39(1)
C(14)	2557(7)	4637(3)	3224(2)	49(1)
C(15)	4464(8)	5090(3)	2891(2)	54(1)
C(16)	5989(6)	5642(3)	3456(2)	50(1)
C(17)	5696(5)	5759(3)	4392(2)	38(1)
C(18)	7116(5)	6407(3)	4998(2)	42(1)
C(19)	6624(5)	6589(3)	5876(2)	41(1)
C(20)	4761(5)	6072(3)	6243(2)	35(1)
C(21)	4298(6)	6559(3)	7166(2)	39(1)
C(22)	1990(6)	6908(3)	7330(2)	46(1)
C(23)	1656(7)	7432(4)	8275(2)	58(1)
C(24)	1799(7)	6511(4)	9034(2)	55(1)
C(25)	55(7)	5765(5)	9188(3)	66(1)
C(26)	161(9)	4910(5)	9876(3)	73(1)
C(27)	2008(9)	4799(5)	10428(3)	76(1)
C(28)	3755(8)	5544(6)	10289(3)	80(2)
C(29)	3648(8)	6390(5)	9601(3)	68(1)
O(1)	330(4)	4309(2)	4440(2)	42(1)
O(2)	5783(4)	6826(3)	7709(2)	60(1)

Supplementary Table 9. Bond lengths [Å] and angles [°] for 3562

C(1)-C(2)	1.366(5)
C(1)-C(6)	1.422(5)
C(1)-H(1)	0.9300
C(2)-C(3)	1.410(6)
C(2)-H(2)	0.9300
C(3)-C(4)	1.366(6)
C(3)-H(3)	0.9300
C(4)-C(5)	1.411(5)
C(4)-H(4)	0.9300
C(5)-C(10)	1.415(5)
C(5)-C(6)	1.432(5)
C(6)-C(7)	1.443(4)
C(7)-C(8)	1.382(4)
C(7)-C(11)	1.478(4)
C(8)-O(1)	1.376(4)
C(8)-C(9)	1.419(5)
C(9)-C(10)	1.352(5)
C(9)-H(9)	0.9300
C(10)-H(10)	0.9300
C(11)-C(20)	1.396(4)
C(11)-C(12)	1.432(4)
C(12)-C(13)	1.409(4)
C(12)-C(17)	1.428(4)
C(13)-C(14)	1.368(5)
C(13)-O(1)	1.386(4)
C(14)-C(15)	1.399(6)
C(14)-H(14)	0.9300
C(15)-C(16)	1.366(5)
C(15)-H(15)	0.9300
C(16)-C(17)	1.409(5)
C(16)-H(16)	0.9300
C(17)-C(18)	1.411(5)
C(18)-C(19)	1.362(5)
C(18)-H(18)	0.9300
C(19)-C(20)	1.424(4)
C(19)-H(19)	0.9300

C(20)-C(21)	1.506(4)
C(21)-O(2)	1.220(4)
C(21)-C(22)	1.516(5)
C(22)-C(23)	1.535(5)
C(22)-H(22A)	0.9700
C(22)-H(22B)	0.9700
C(23)-C(24)	1.507(5)
C(23)-H(23A)	0.9700
C(23)-H(23B)	0.9700
C(24)-C(29)	1.385(6)
C(24)-C(25)	1.388(6)
C(25)-C(26)	1.381(7)
C(25)-H(25)	0.9300
C(26)-C(27)	1.372(7)
C(26)-H(26)	0.9300
C(27)-C(28)	1.383(7)
C(27)-H(27)	0.9300
C(28)-C(29)	1.376(6)
C(28)-H(28)	0.9300
C(29)-H(29)	0.9300
C(2)-C(1)-C(6)	121.5(4)
C(2)-C(1)-H(1)	119.2
C(6)-C(1)-H(1)	119.2
C(1)-C(2)-C(3)	120.4(4)
C(1)-C(2)-H(2)	119.8
C(3)-C(2)-H(2)	119.8
C(4)-C(3)-C(2)	120.1(4)
C(4)-C(3)-H(3)	120.0
C(2)-C(3)-H(3)	120.0
C(3)-C(4)-C(5)	120.9(4)
C(3)-C(4)-H(4)	119.6
C(5)-C(4)-H(4)	119.6
C(4)-C(5)-C(10)	120.7(3)
C(4)-C(5)-C(6)	119.7(4)
C(10)-C(5)-C(6)	119.4(3)
C(1)-C(6)-C(5)	117.3(3)
C(1)-C(6)-C(7)	123.5(3)

C(5)-C(6)-C(7)	119.0(3)
C(8)-C(7)-C(6)	116.7(3)
C(8)-C(7)-C(11)	117.4(3)
C(6)-C(7)-C(11)	125.4(3)
O(1)-C(8)-C(7)	123.2(3)
O(1)-C(8)-C(9)	113.7(3)
C(7)-C(8)-C(9)	123.1(3)
C(10)-C(9)-C(8)	118.8(4)
C(10)-C(9)-H(9)	120.6
C(8)-C(9)-H(9)	120.6
C(9)-C(10)-C(5)	121.3(3)
C(9)-C(10)-H(10)	119.4
C(5)-C(10)-H(10)	119.4
C(20)-C(11)-C(12)	117.7(3)
C(20)-C(11)-C(7)	127.6(3)
C(12)-C(11)-C(7)	114.7(3)
C(13)-C(12)-C(17)	117.4(3)
C(13)-C(12)-C(11)	120.7(3)
C(17)-C(12)-C(11)	121.8(3)
C(14)-C(13)-O(1)	117.7(3)
C(14)-C(13)-C(12)	122.6(3)
O(1)-C(13)-C(12)	119.6(3)
C(13)-C(14)-C(15)	119.0(4)
C(13)-C(14)-H(14)	120.5
C(15)-C(14)-H(14)	120.5
C(16)-C(15)-C(14)	120.8(3)
C(16)-C(15)-H(15)	119.6
C(14)-C(15)-H(15)	119.6
C(15)-C(16)-C(17)	121.0(3)
C(15)-C(16)-H(16)	119.5
C(17)-C(16)-H(16)	119.5
C(16)-C(17)-C(18)	123.8(3)
C(16)-C(17)-C(12)	119.0(3)
C(18)-C(17)-C(12)	117.1(3)
C(19)-C(18)-C(17)	120.7(3)
C(19)-C(18)-H(18)	119.7
C(17)-C(18)-H(18)	119.7
C(18)-C(19)-C(20)	122.4(3)

C(18)-C(19)-H(19)	118.8
C(20)-C(19)-H(19)	118.8
C(11)-C(20)-C(19)	118.7(3)
C(11)-C(20)-C(21)	127.1(3)
C(19)-C(20)-C(21)	113.9(3)
O(2)-C(21)-C(20)	120.1(3)
O(2)-C(21)-C(22)	121.1(3)
C(20)-C(21)-C(22)	117.9(3)
C(21)-C(22)-C(23)	114.9(3)
C(21)-C(22)-H(22A)	108.6
C(23)-C(22)-H(22A)	108.6
C(21)-C(22)-H(22B)	108.6
C(23)-C(22)-H(22B)	108.6
H(22A)-C(22)-H(22B)	107.5
C(24)-C(23)-C(22)	114.7(3)
C(24)-C(23)-H(23A)	108.6
C(22)-C(23)-H(23A)	108.6
C(24)-C(23)-H(23B)	108.6
C(22)-C(23)-H(23B)	108.6
H(23A)-C(23)-H(23B)	107.6
C(29)-C(24)-C(25)	118.0(4)
C(29)-C(24)-C(23)	121.5(4)
C(25)-C(24)-C(23)	120.5(4)
C(26)-C(25)-C(24)	121.3(4)
C(26)-C(25)-H(25)	119.3
C(24)-C(25)-H(25)	119.3
C(27)-C(26)-C(25)	119.9(5)
C(27)-C(26)-H(26)	120.1
C(25)-C(26)-H(26)	120.1
C(26)-C(27)-C(28)	119.5(5)
C(26)-C(27)-H(27)	120.2
C(28)-C(27)-H(27)	120.2
C(29)-C(28)-C(27)	120.4(5)
C(29)-C(28)-H(28)	119.8
C(27)-C(28)-H(28)	119.8
C(28)-C(29)-C(24)	120.8(5)
C(28)-C(29)-H(29)	119.6
C(24)-C(29)-H(29)	119.6

C(8)-O(1)-C(13)	118.0(2)

Symmetry transformations used to generate equivalent atoms:

Supplementary Table 10. Anisotropic displacement parameters ($Å^2 x 10^3$)for 3562. The anisotropic displacement factor exponent takes the form: $-2\pi^2[h^2a^{*2}U^{11} + ... + 2 h k a^* b^* U^{12}]$

	U^{11}	U^{22}	U^{33}	U^{23}	U^{13}	U^{12}
C(1)	44(2)	37(2)	41(2)	5(2)	2(2)	2(2)
C(2)	67(3)	47(2)	44(2)	6(2)	4(2)	6(2)
C(3)	83(3)	55(2)	48(2)	11(2)	25(2)	4(2)
C(4)	68(3)	45(2)	61(3)	7(2)	26(2)	-9(2)
C(5)	47(2)	32(2)	54(2)	-3(2)	17(2)	1(2)
C(6)	38(2)	29(2)	43(2)	-1(1)	11(2)	1(1)
C(7)	31(2)	28(1)	42(2)	-1(1)	7(1)	1(1)
C(8)	32(2)	30(2)	44(2)	-1(1)	1(1)	2(1)
C(9)	34(2)	35(2)	62(2)	-8(2)	1(2)	-1(1)
C(10)	38(2)	38(2)	70(3)	-4(2)	16(2)	-4(2)
C(11)	31(2)	26(1)	37(2)	2(1)	1(1)	3(1)
C(12)	36(2)	24(1)	37(2)	3(1)	3(1)	7(1)
C(13)	45(2)	29(2)	41(2)	0(1)	-2(2)	3(1)
C(14)	69(2)	41(2)	36(2)	-3(2)	-3(2)	1(2)
C(15)	79(3)	48(2)	36(2)	2(2)	10(2)	7(2)
C(16)	55(2)	45(2)	50(2)	10(2)	19(2)	8(2)
C(17)	42(2)	28(1)	45(2)	8(1)	11(2)	6(1)
C(18)	33(2)	36(2)	58(2)	8(2)	9(2)	-2(1)
C(19)	36(2)	32(2)	54(2)	3(2)	-4(2)	-6(2)
C(20)	34(2)	29(1)	40(2)	3(1)	-2(1)	-1(1)
C(21)	47(2)	34(2)	35(2)	2(2)	-5(2)	-4(2)
C(22)	55(2)	44(2)	37(2)	-7(2)	-4(2)	11(2)
C(23)	75(3)	51(2)	47(2)	-11(2)	7(2)	12(2)
C(24)	67(3)	60(2)	38(2)	-16(2)	11(2)	6(2)
C(25)	56(2)	80(3)	60(3)	-12(3)	3(2)	3(3)
C(26)	73(3)	81(3)	68(3)	-9(3)	19(2)	-17(3)
C(27)	90(4)	93(4)	47(3)	8(2)	13(2)	-13(3)
C(28)	81(3)	115(4)	41(2)	9(3)	-8(2)	-15(4)
C(29)	72(3)	92(3)	39(2)	1(2)	2(2)	-19(3)
O(1)	35(1)	47(1)	44(1)	-2(1)	-5(1)	-2(1)
O(2)	57(2)	75(2)	45(1)	-4(2)	-12(1)	-11(2)

Supplementary Table 11. Hydrogen coordinates (x 10^4) and isotropic displacement parameters (Å^{2}x 10^3) for 3562

	x	y	z	U(eq)
H(1)	4779	4354	7407	49
H(2)	4724	3318	8745	63
H(3)	1649	2239	9109	74
H(4)	-1276	2126	8089	68
H(9)	-2692	3208	5120	53
H(10)	-2987	2561	6577	58
H(14)	1498	4292	2833	58
H(15)	4695	5014	2276	65
H(16)	7238	5945	3221	60
H(18)	8401	6713	4797	51
H(19)	7539	7068	6248	49
H(22A)	1085	6193	7241	55
H(22B)	1505	7504	6878	55
H(23A)	2731	8058	8406	69
H(23B)	248	7817	8264	69
H(25)	-1211	5842	8822	79
H(26)	-1019	4410	9963	88
H(27)	2085	4227	10894	92
H(28)	5009	5471	10663	95
H(29)	4831	6888	9516	81

Supplementary Table 12. Torsion angles [°] for 3562

C(6)-C(1)-C(2)-C(3)	-1.6(6)
C(1)-C(2)-C(3)-C(4)	2.0(6)
C(2)-C(3)-C(4)-C(5)	0.2(6)
C(3)-C(4)-C(5)-C(10)	172.9(4)
C(3)-C(4)-C(5)-C(6)	-2.7(6)
C(2)-C(1)-C(6)-C(5)	-0.9(5)
C(2)-C(1)-C(6)-C(7)	-176.7(3)
C(4)-C(5)-C(6)-C(1)	3.0(5)
C(10)-C(5)-C(6)-C(1)	-172.7(3)
C(4)-C(5)-C(6)-C(7)	179.0(3)
C(10)-C(5)-C(6)-C(7)	3.3(4)
C(1)-C(6)-C(7)-C(8)	162.9(3)
C(5)-C(6)-C(7)-C(8)	-12.8(4)
C(1)-C(6)-C(7)-C(11)	-9.1(5)
C(5)-C(6)-C(7)-C(11)	175.2(3)
C(6)-C(7)-C(8)-O(1)	-164.3(3)
C(11)-C(7)-C(8)-O(1)	8.4(4)
C(6)-C(7)-C(8)-C(9)	14.7(4)
C(11)-C(7)-C(8)-C(9)	-172.7(3)
O(1)-C(8)-C(9)-C(10)	172.7(3)
C(7)-C(8)-C(9)-C(10)	-6.4(5)
C(8)-C(9)-C(10)-C(5)	-4.2(5)
C(4)-C(5)-C(10)-C(9)	-170.2(3)
C(6)-C(5)-C(10)-C(9)	5.4(5)
C(8)-C(7)-C(11)-C(20)	158.2(3)
C(6)-C(7)-C(11)-C(20)	-29.9(5)
C(8)-C(7)-C(11)-C(12)	-23.7(4)
C(6)-C(7)-C(11)-C(12)	148.2(3)
C(20)-C(11)-C(12)-C(13)	-164.0(3)
C(7)-C(11)-C(12)-C(13)	17.7(4)
C(20)-C(11)-C(12)-C(17)	13.2(4)
C(7)-C(11)-C(12)-C(17)	-165.1(3)
C(17)-C(12)-C(13)-C(14)	3.3(4)
C(11)-C(12)-C(13)-C(14)	-179.4(3)
C(17)-C(12)-C(13)-O(1)	-173.0(3)
C(11)-C(12)-C(13)-O(1)	4.3(4)

O(1)-C(13)-C(14)-C(15)	176.9(3)
C(12)-C(13)-C(14)-C(15)	0.6(5)
C(13)-C(14)-C(15)-C(16)	-2.6(6)
C(14)-C(15)-C(16)-C(17)	0.7(6)
C(15)-C(16)-C(17)-C(18)	-174.5(3)
C(15)-C(16)-C(17)-C(12)	3.3(5)
C(13)-C(12)-C(17)-C(16)	-5.1(4)
C(11)-C(12)-C(17)-C(16)	177.6(3)
C(13)-C(12)-C(17)-C(18)	172.8(3)
C(11)-C(12)-C(17)-C(18)	-4.5(4)
C(16)-C(17)-C(18)-C(19)	173.3(3)
C(12)-C(17)-C(18)-C(19)	-4.4(5)
C(17)-C(18)-C(19)-C(20)	4.5(5)
C(12)-C(11)-C(20)-C(19)	-12.9(4)
C(7)-C(11)-C(20)-C(19)	165.2(3)
C(12)-C(11)-C(20)-C(21)	160.4(3)
C(7)-C(11)-C(20)-C(21)	-21.5(5)
C(18)-C(19)-C(20)-C(11)	4.5(5)
C(18)-C(19)-C(20)-C(21)	-169.7(3)
C(11)-C(20)-C(21)-O(2)	151.4(4)
C(19)-C(20)-C(21)-O(2)	-35.0(4)
C(11)-C(20)-C(21)-C(22)	-39.1(5)
C(19)-C(20)-C(21)-C(22)	134.5(3)
O(2)-C(21)-C(22)-C(23)	-8.9(5)
C(20)-C(21)-C(22)-C(23)	-178.3(3)
C(21)-C(22)-C(23)-C(24)	-73.0(5)
C(22)-C(23)-C(24)-C(29)	100.6(5)
C(22)-C(23)-C(24)-C(25)	-80.1(5)
C(29)-C(24)-C(25)-C(26)	-1.2(6)
C(23)-C(24)-C(25)-C(26)	179.5(4)
C(24)-C(25)-C(26)-C(27)	0.9(7)
C(25)-C(26)-C(27)-C(28)	-0.3(8)
C(26)-C(27)-C(28)-C(29)	0.0(8)
C(27)-C(28)-C(29)-C(24)	-0.3(8)
C(25)-C(24)-C(29)-C(28)	0.8(7)
C(23)-C(24)-C(29)-C(28)	-179.8(4)
C(7)-C(8)-O(1)-C(13)	14.4(4)
C(9)-C(8)-O(1)-C(13)	-164.6(3)

C(14)-C(13)-O(1)-C(8)	162.7(3)
C(12)-C(13)-O(1)-C(8)	-20.8(4)

Symmetry transformations used to generate equivalent atoms:

8. UV-Visible Spectroscopy:[15]

We have performed the UV measurement with n the range of 200 to 800 nm for both 11a and 11b compounds. First we have recorded the UV of $1x10^{-2}$ (M), $1x10^{-3}$ (M), $1x10^{-4}$ (M), $0.5x10^{-4}$ (M), $0.25x10^{-4}$ (M), $0.125x10^{-4}$ (M), 10^{-5} (M), 10^{-6} (M) of 10a in DCM medium. A comparison spectrum has been given in supplementary figure 6. We have also recorded the UV at 10^{-4} (M) concentration of 11a in various polar to non-polar solvents. Based on the dielectric constant of the solvent a prominent hypocromic-hypercromic shift as well as red-blue shift were observed (supplementary figure 7 and 8). The electronic absorption of each sample was measured with a Shimadzu UV-2450 spectrophotometer.

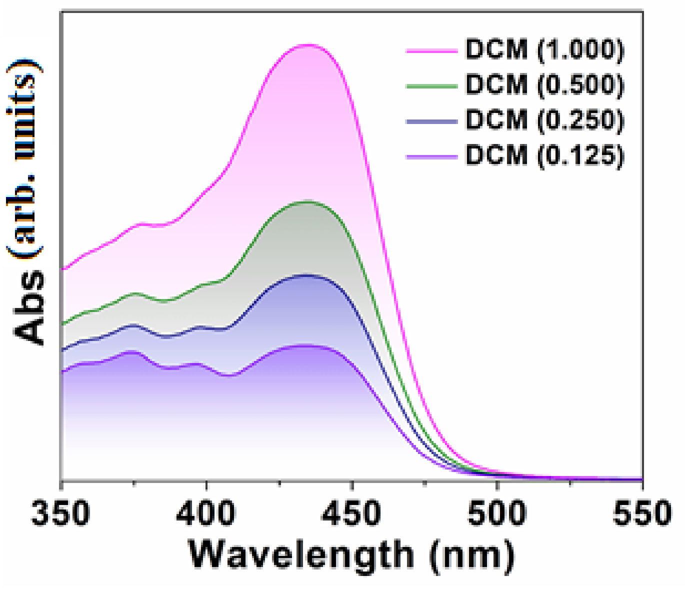

Supplementary figure 6. UV of **11a** at different concentration of DCM

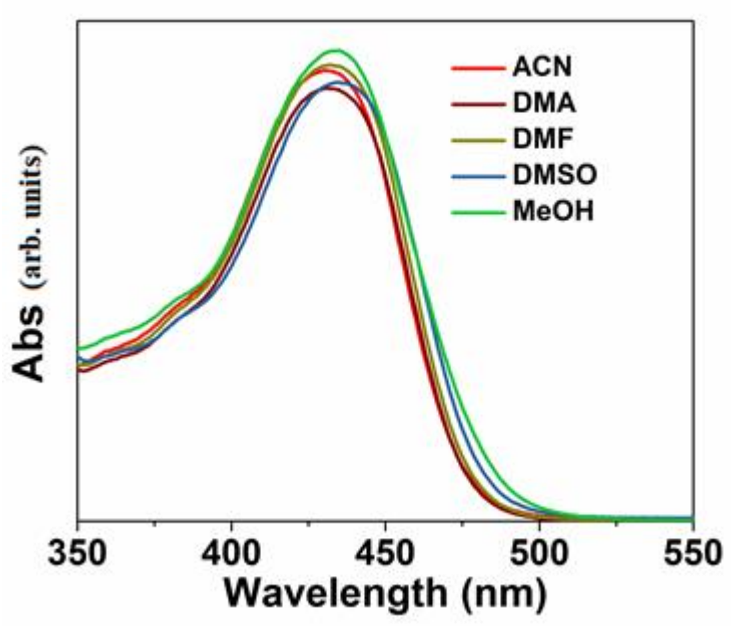

Supplementary figure 7. UV of **11a** at 10^{-4} (M) of different solvent

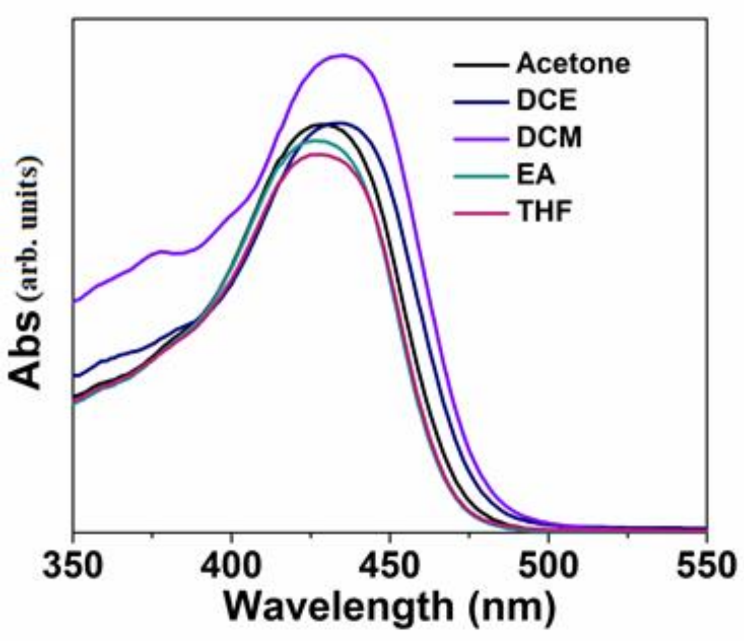

Supplementary figure 8. UV of **11a** at 10^{-4} (M) of different solvent

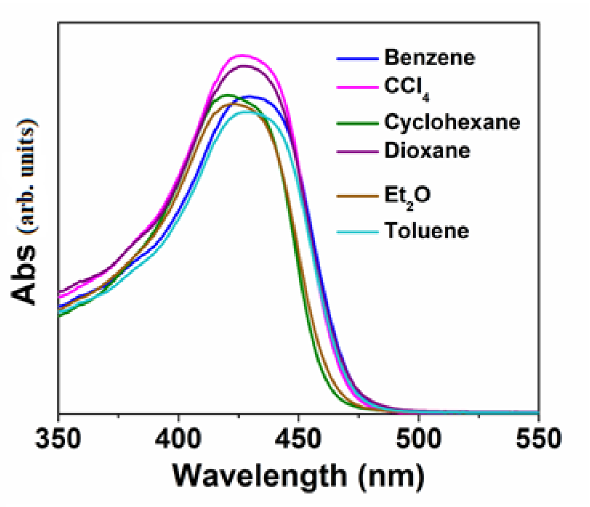

Supplementary figure 9. UV of **11a** at 10^{-4} (M) of different solvent

For the compound 11b, we have recorded the UV of $1x10^{-2}$ (M), $1x10^{-3}$ (M), $0.5x10^{-3}$ (M), $0.25x10^{-3}$ (M), $0.125x10^{-3}$ (M), 10^{-4} (M), 10^{-5} (M) of 11b in DCM medium. A comparison spectra has been given in supplementary figure 10.

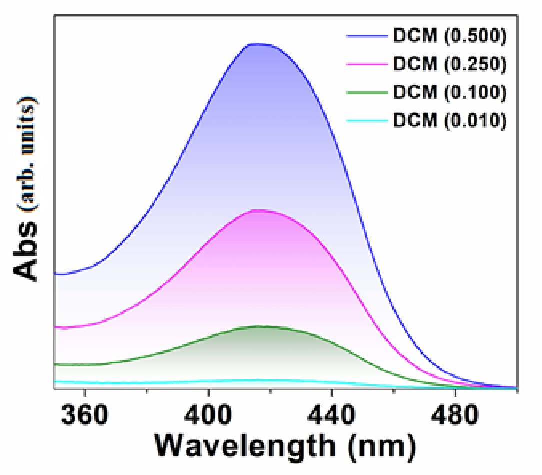

Supplementary figure 10. UV of **11b** at 10^{-4} (M) concentration of different solvent

9. **Steady-State Emission Spectroscopy:**[15]

Samples for emission spectroscopy were similarly prepared as described for absorption spectra. Fluorescence emission spectra for photocatalysts 11a and 11b were also determined in the aprotic solvents like DMSO, DCM, and acetonitrile, and in the protic solvents like MeOH Shimadzu RF-6000 spectrofluorometer. The corresponding emission maxima are listed in the experimental section of the corresponding compound. The fluorescence emission spectra of 11a and 11b are shown below. The change in emission intensity of both the 11a and 11b have been observed in DCM with varying concentrations.

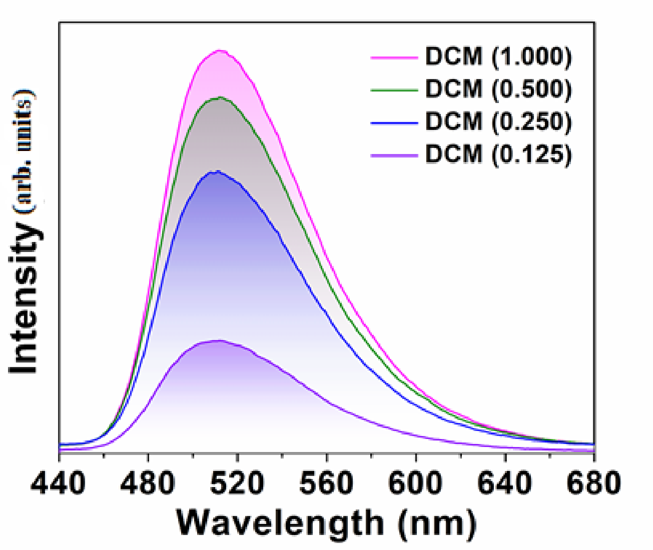

Supplementary figure 11. Fluorescence of **11a** at different concentration of DCM

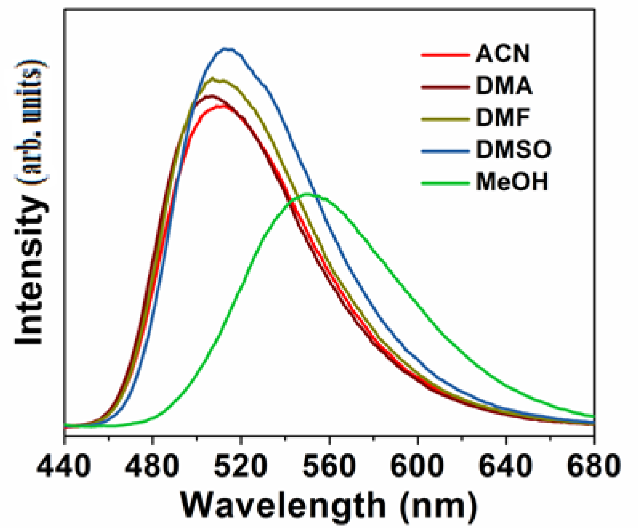

Supplementary figure 12. Fluorescence of **11a** at 10^{-4} (M) concentration of different solvent

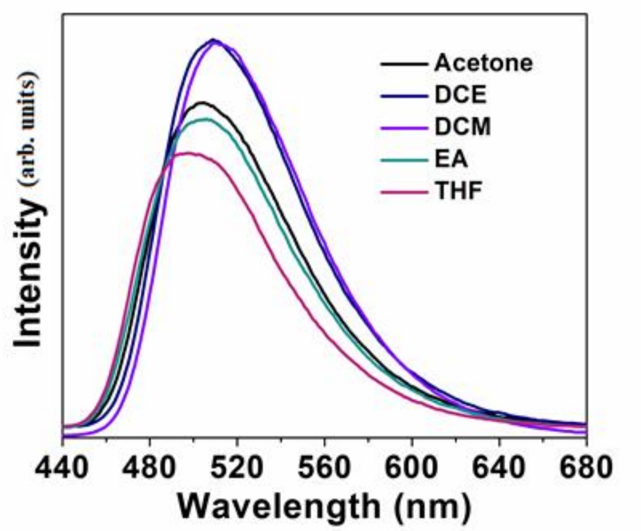

Supplementary figure 13. Fluorescence of **11a** at 10^{-4} (M) concentration of different solvent

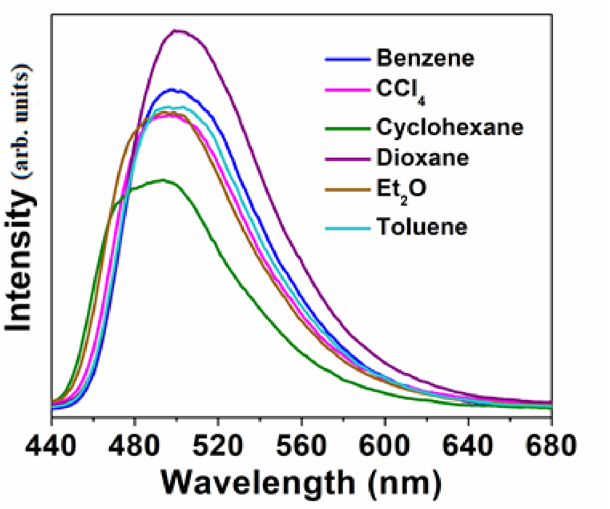

Supplementary figure 14. Fluorescence of **11a** at 10^{-4} (M) concentration of different solvent

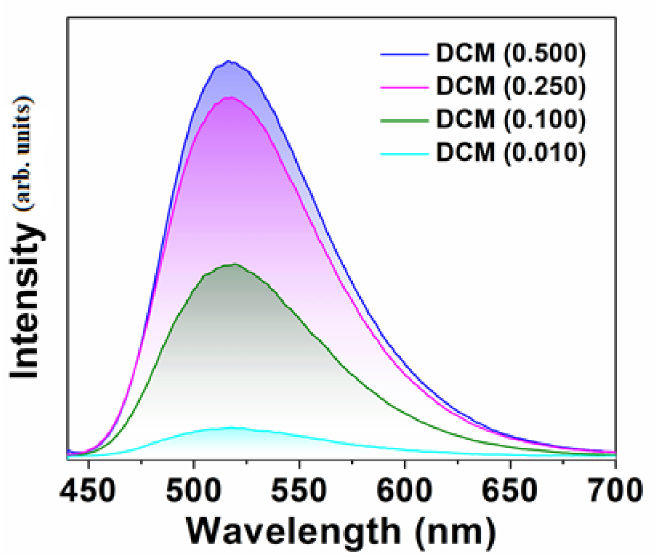

Supplementary figure 15. Fluorescence of **11b** at different concentration of DCM

10. Fluorescence Lifetimes (τ_F):[15]

The TCSPC measurement, which is actually a time-resolved emission measurement, gives us the excited state decay dynamics of a fluorescent molecule through the excited state lifetime information. In the TCSPC measurement module, we have used a picosecond diode LASER unit with its peak wavelength at 420 nm excitation LASER source. The fluorescence signals generated are detected using the Hamamatsu MCP PMT (3809U) detector at the polarizer angle of ~54.7°, which is called magic angle polarization. The TCSPC decays are collected in forward mode and analyzed with respect to the prompt (or instrument response function (IRF)) that has been taken. The IBH DAS-6 software has been utilized to fit the collected decays using the respective order of exponential equation to get the subsequent lifetime values and contributions. The average lifetime (τ_{avg}) has been calculated using the obtained lifetime information following the supplementary equation below, where τ_i is the lifetime component, and a_i is the respective amplitude. Contributions with i represent the order of the exponential equation used to fit the lifetime decay trace, and $\Sigma\, a_i = 1$.

$\tau_{ave} = \Sigma\, a_i\, \tau_i$ ....(1)

The monitored emission wavelength for 11a is 512 nm and 515 nm. The corresponding fluorescence lifetimes (τ_F) are 9.8 ± 0.01844 ns and 10.5 ± 0.019897 ns. The Fig. 16 and Fig. 17 are belongs to the 11a and 11b respectively.

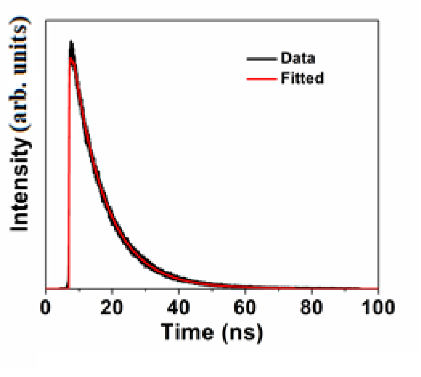

Supplementary figure 16. TCSPC decay profile of **11a** at 10^{-4} (M) concentration of DCM

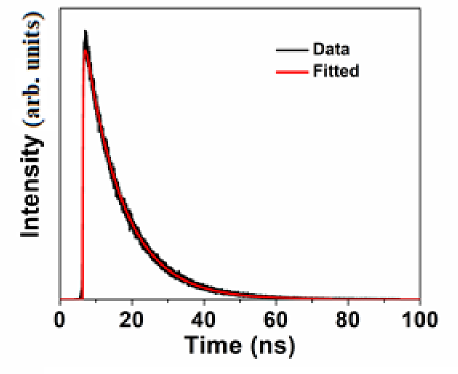

Supplementary figure 17. TCSPC decay profile of **11b** at $0.5x10^{-3}$ (M) concentration of DCM

11. Fluorescence Quantum Yields (Φ_F):[15]

The fluorescence quantum yield (Φ_F) is defined as the fraction of the number of quanta absorbed by a molecule that is emitted as fluorescence. The Φ_F of 11a and 11b were determined by the reference point method.[15] A solution of quinine sulfate (Φ_F = 0.546 in 0.5 M H_2SO_4) was used as the standard for the fluorescence quantum yield determinations following the supplementary equation given below. An error of 5% is estimated for the fluorescence quantum yields. The absorbance values of the solutions at the excitation wavelength were measured with a UV-Vis spectrophotometer. Photoluminescence (PL) emission spectra of all the sample solutions were recorded by Shimadzu RF-6000 spectrofluorophotometer at 420 nm excitation wavelength of compounds. Where Φ_F represents the fluorescence quantum yield, "A" is the absorbance monitored at the excitation wavelength, "F" designates the areas under the fluorescence emission curves, and "η" is the refractive index of the medium. For DCM solvent, the refractive index is 1.4244, and for 0.5 M H_2SO_4, the refractive index is 1.346. The subscripts "S" and "R" denote the corresponding parameters for the sample and reference, respectively. We found that the Φ_F of 11a is 0.55 and of 11b is 0.61.

$$\Phi_{F(S)} = \Phi_{F\circledR} \times (F_S/F_R) \times (A_R/A_S) \times (\eta_S/\eta_R)^2 \quad \dots\ \dots\ \dots\ (2)$$

12. Phosphorescence Spectra and Lifetimes:[15]

Phosphorescence decay time of the sample was measured using a PMT detector attached with the steady state photoluminescence (SSPL) where the excitation source is a Zenon flash lamp. The instrument model is HORIBA QuataMaster. The pulse width and the repetition rate of the excitation source were 100 ns and 300 MHz, respectively. The raw data were analyzed with OriginPro 2016 software by using the exponential function to obtain the phosphorescence lifetime. Each 10a (10^{-4} M) and 10b (10^{-4} M) in DCM were taken to record phosphorescence emission and time-resolved spectra with an excitation wavelength λ_{ex} = 420 nm. The 3 mL of each solution is taken in quartz cuvette for the measurement. The solution is purged with Ar gas prior to recording phosphorescence and the experiment was carried out at 300 K.

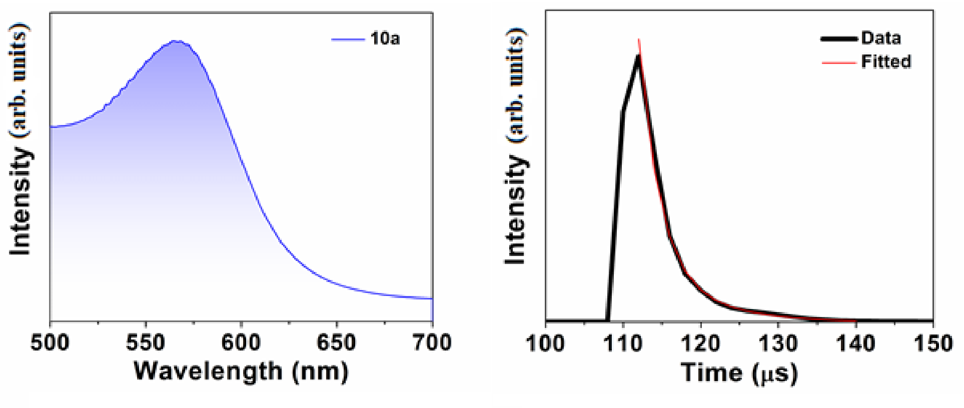

Supplementary figure 18. Photoluminescence spectra of 11a in desecrated DCM glassy matrix and emission decay at 10^{-4} (M) concentration of DCM. Excited at 420 nm. Emission spectra has λ_{mas} at 560 nm and τ_P = 4.0 μs.

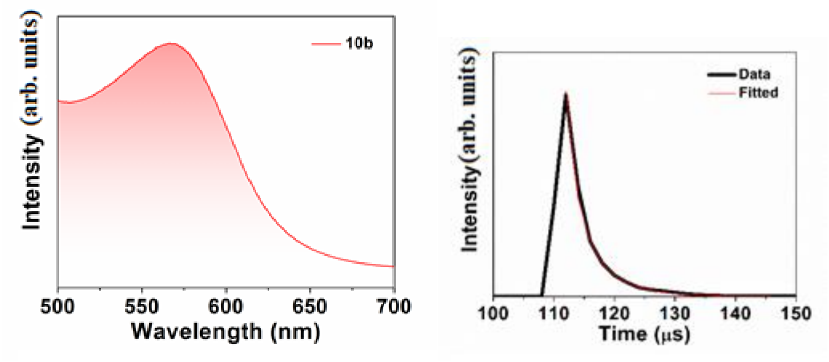

Supplementary figure 19. Photoluminescence spectra of 11b in desecrated DCM glassy matrix and emission decay at $0.5x10^{-3}$ (M) concentration of DCM. Excited at 420 nm. Emission spectra has λ_{mas} at 570 nm and $\tau_P = 8.0$ μs.

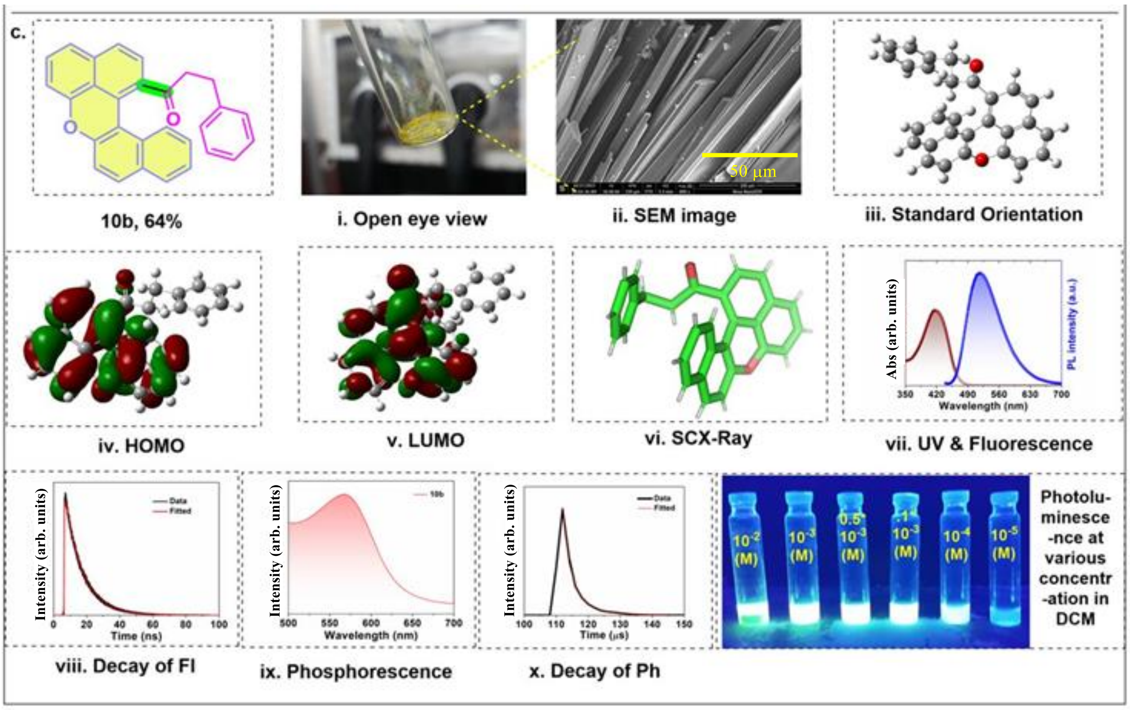

Supplementary figure 20: Details of 11b

13. Electrochemical Measurements:[15]

All electrochemical experiments were performed using CH Instruments (model CHI700E electrochemical analyzer). Cyclic Voltammetry (CV) of all compounds was recorded inside using degassed ACN. The glassy carbon electrode was taken as the working electrode, standard double-junction Ag/AgCl (saturated KCl) as the reference electrode, and a Pt was taken as the counter electrode. 4 mL of 0.5 mM compound solution in anhydrous CH_3CN was taken in the presence of 0.1 M Tetrabutylammonium hexafluorophosphate (TBAP) as the supporting electrolyte, and the scan rate was 50 mV/s. The red coloured line is belongs to compound while the black coloured line is belongs to only ACN solvent.

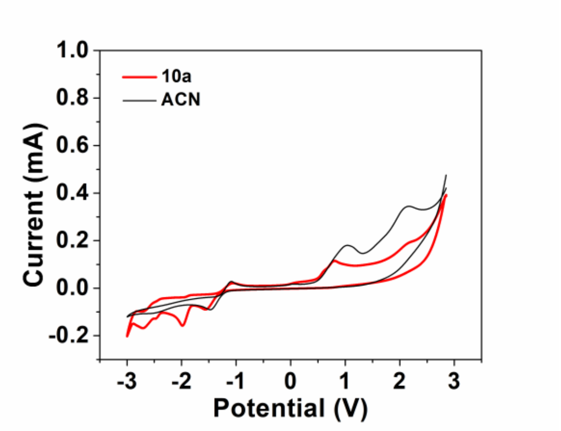

Supplementary figure 21. CV of 11a in CAN and the potential are given w.r.t NHE. We found that $E_{1/2}(11a)/(11a)^{+}$ = 0.80 V whereas $E_{1/2}(11a)/(11a)^{-}$ = -1.67 V, $E_{1/2}(11a)^{-}/(11a)^{2-}$ = -2.22 V and $E_{1/2}(11a)^{2-}/(11a)^{3-}$ = -2.39 V.

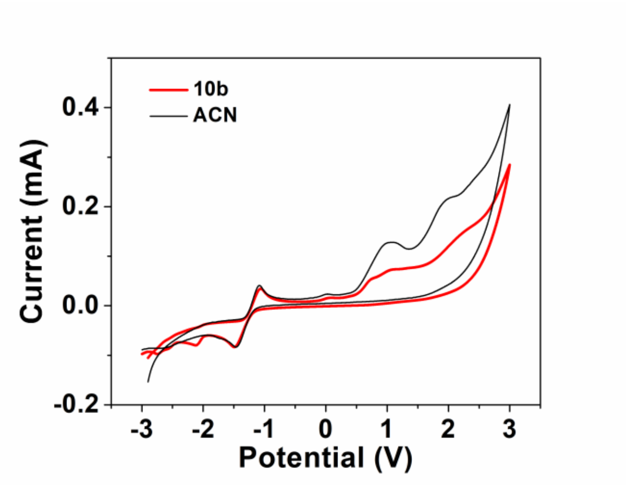

Supplementary figure 22. CV of 11b in CAN and the potential are given w.r.t NHE. We found that $E_{1/2}(11b)/(11b)^{+}$ = 0.76 V whereas $E_{1/2}(11b)/(11b)^{-}$ = -1.83 V, $E_{1/2}(11b)^{-}/(11b)^{2-}$ = -2.29 V and $E_{1/2}(11b)^{2-}/(11b)^{3-}$ = -2.49 V.

14. HOMO-LUMO energy calculation and excited state potential calculation from Electrochemical Measurements:

We have engaged the following two supplementary equation for the HOMO-LUMO energy calculation.[16]

$E_{HOMO} = -(E_{ox\ vs\ NHE} + 4.75)$ eV …………………eq 3

$E_{LUMO} = -(E_{red\ vs\ NHE} + 4.75)$ eV ……………….. eq 4

Therefore for the compound 11a we found the E_{HOMO} = -5.55 eV and the LUMO = -3.08 eV whereas for the compound 11b E_{HOMO} = -5.51 eV and the LUMO = -2.92 eV. Hence for 11a the E_{gap} = 2.47 and for 11b E_{gap} = 2.59 w.r.t electrochemical measurement. Further the excited state potential have been calculated according to the following supplementary equations.[17]

$E_{1/2}(11a^{*})/(11a)^{-} = [E_{1/2}(11a)/(11a)^{-}]_{vs\ SCE} + E_{0,0}$ …………….eq 5

$E_{1/2}(11b^{*})/(11b)^{-} = [E_{1/2}(11b)/(11b)^{-}]_{vs\ SCE} + E_{0,0}$ …………….eq 6

Here $E_{0,0}$ for 11a is ~ 2.81 eV, hence $E_{1/2}(11a^{*})/(11a)^{-}$ ~ 0.9 V vs SCE. Next for the $E_{0,0}$ for 11b is ~ 2.8 eV, hence $E_{1/2}(11b^{*})/(11b)^{-}$ ~ 0.8 V vs SCE.

15. Computational Methods:

For geometry calculations, all the calculations were carried out using DFT (Density Functional Theory) with the Gaussian 09 program package.[19] Geometry optimizations were carried out with B3LYP[8] level of theory and 6-311G* basis set was used for the Li, B, C, O, H elements using the polarizable continuum model (PCM), using THF as a solvent. To learn more about the reactant, we used the NBO tool in Gaussian 09. The reported energies are

Gibbs free energies, which include zero-point vibrational corrections, thermal corrections, and entropy corrections at 298 K. The latter are calculated as single-point corrections on the optimized structures with the same basis set combination used for the geometry optimizations. Based on the optimized geometries, all energies were also corrected with single-point dispersion effects using the DFT method.[20-22]

Supplementary Table 13: The absolute energies and Thermal correction to Gibbs free energy (a.u.)

Structure	**Absolute energies B3LYP/ 6-311+G***	**Thermal correction to Gibbs free energy (353.15 K)**
IM-1	-536.493539	0.130836
IM-2	-868.743434	0.329170
IM-3	-1405.240664	0.486345
IM-4	-1405.237295	0.487650
IM-5	-911.165984	0.311467
2a	-460.116489	0.107831

Cartesian coordinates of optimized structures for IM-1.

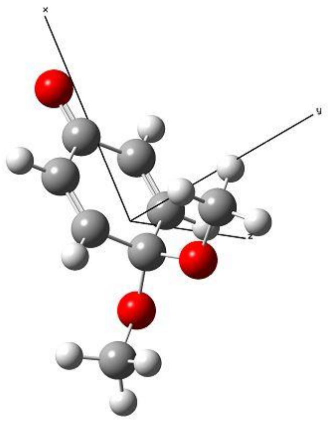

Standard orientation

Supplementary figure 23. NBO calculation

Zero-point correction= 0.167807 (Hartree/Particle)
Thermal correction to Energy= 0.178825
Thermal correction to Enthalpy= 0.179769
Thermal correction to Gibbs Free Energy= 0.130836
Sum of electronic and zero-point Energies= -536.456568

Sum of electronic and thermal Energies= -536.445550
Sum of electronic and thermal Enthalpies= -536.444606
Sum of electronic and thermal Free Energies= -536.493539

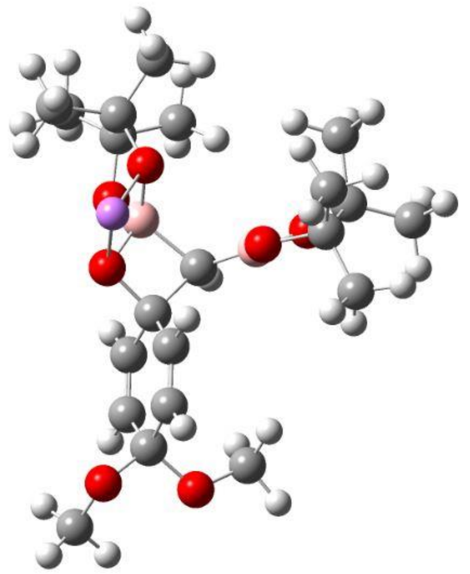

IM-4, ΔG = 2.48 kcal/mol

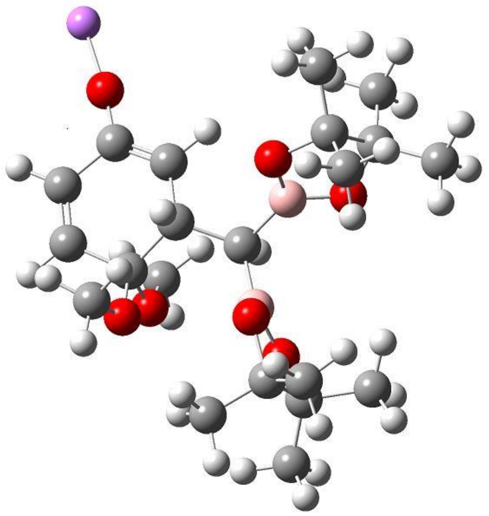

IM-4, ΔG = 3.22 kcal/mol

Supplementary figure 24. Optimized intermediate structures and calculated barriers (kcal/mol) for the **IM-3 and IM-4**

Cartesian coordinates of optimized structures for IM-2

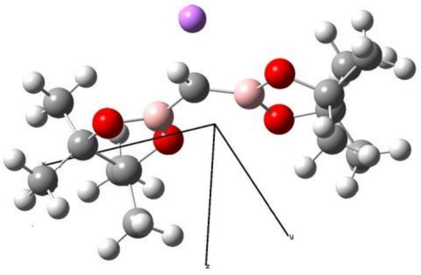

Standard orientation

Zero-point correction= 0.378301 (Hartree/Particle)
Thermal correction to Energy= 0.400564
Thermal correction to Enthalpy= 0.401509

Thermal correction to Gibbs Free Energy= 0.329170
Sum of electronic and zero-point Energies= -868.694303
Sum of electronic and thermal Energies= -868.672040
Sum of electronic and thermal Enthalpies= -868.671096
Sum of electronic and thermal Free Energies= -868.743434

Cartesian coordinates of optimized structures for IM-3

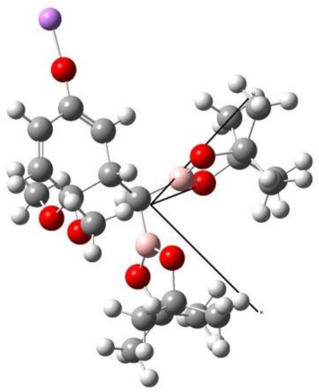

Standard orientation

Zero-point correction= 0.550639 (Hartree/Particle)
Thermal correction to Energy= 0.584519
Thermal correction to Enthalpy= 0.585463
Thermal correction to Gibbs Free Energy= 0.486345
Sum of electronic and zero-point Energies= -1405.176370
Sum of electronic and thermal Energies= -1405.142490
Sum of electronic and thermal Enthalpies= -1405.141546
Sum of electronic and thermal Free Energies= -1405.240664

Cartesian coordinates of optimized structures for IM-4

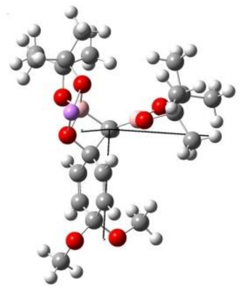

Standard orientation

Zero-point correction= 0.550250 (Hartree/Particle)
Thermal correction to Energy= 0.583349

Thermal correction to Enthalpy= 0.584293
Thermal correction to Gibbs Free Energy= 0.487650
Sum of electronic and zero-point Energies= -1405.174695
Sum of electronic and thermal Energies= -1405.141596
Sum of electronic and thermal Enthalpies= -1405.140652
Sum of electronic and thermal Free Energies= -1405.237295

Cartesian coordinates of optimized structures for IM-5

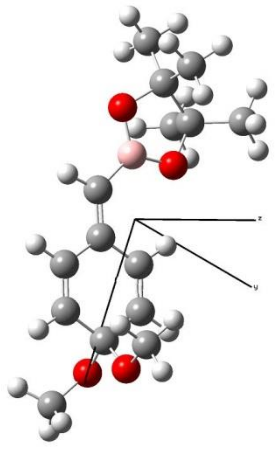

Standard orientation

Zero-point correction= 0.363042 (Hartree/Particle)
Thermal correction to Energy= 0.384362
Thermal correction to Enthalpy= 0.385306
Thermal correction to Gibbs Free Energy= 0.311467
Sum of electronic and zero-point Energies= -911.114409
Sum of electronic and thermal Energies= -911.093088
Sum of electronic and thermal Enthalpies= -911.092144
Sum of electronic and thermal Free Energies= -911.165984

Cartesian coordinates of optimized structures for 2a

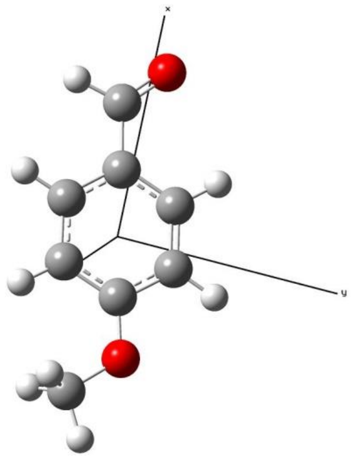

Standard orientation

Zero-point correction= 0.141971 (Hartree/Particle)
Thermal correction to Energy= 0.150895
Thermal correction to Enthalpy= 0.151839
Thermal correction to Gibbs Free Energy= 0.107831
Sum of electronic and zero-point Energies= -460.082348
Sum of electronic and thermal Energies= -460.073425
Sum of electronic and thermal Enthalpies= -460.072480
Sum of electronic and thermal Free Energies= -460.116489

Cartesian coordinates of optimized structures for 11a

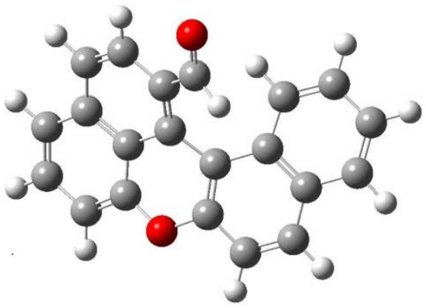

Standard Orientation

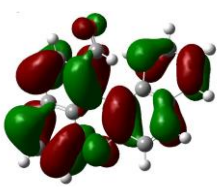

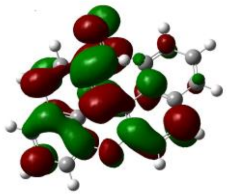

HOMO of 11a **LUMO of 11a**

Zero-point correction= 0.267313 (Hartree/Particle)
Thermal correction to Energy= 0.282944
Thermal correction to Enthalpy= 0.283889
Thermal correction to Gibbs Free Energy= 0.224881
Sum of electronic and zero-point Energies= -957.630069
Sum of electronic and thermal Energies= -957.614438
Sum of electronic and thermal Enthalpies= -957.613494
Sum of electronic and thermal Free Energies= -957.672501

Cartesian coordinates of optimized structures for 11b

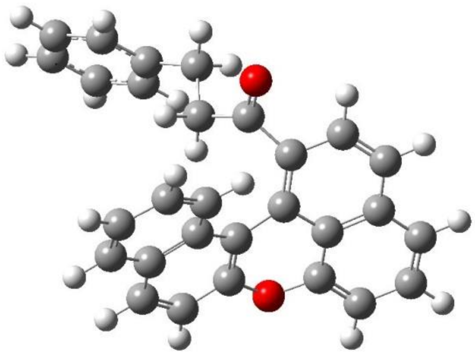

Standard Orientation

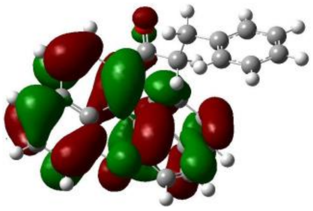

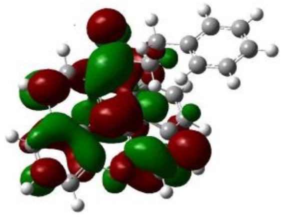

HOMO of 11b **LUMO of 11b**

Zero-point correction= 0.405742 (Hartree/Particle)
Thermal correction to Energy= 0.428719
Thermal correction to Enthalpy= 0.429663
Thermal correction to Gibbs Free Energy= 0.351077
Sum of electronic and zero-point Energies= -1267.169207

Sum of electronic and thermal Energies= -1267.146230
Sum of electronic and thermal Enthalpies= -1267.145286
Sum of electronic and thermal Free Energies= -1267.223872

16. Boron-NMR for the reaction and other result:

We have also carried out the boron NMR study to confirm the formation of IM-6 and IM-7. After the immediate addition of the lithiated germinal B(pin) to the quinketal, we recorded a boron NMR of the reaction mixture with $CDCl_3$ capillary. We observed three different peaks. One peak at 10.85 ppm belongs to the oxygen coordinated boron. The peak at 36.22 ppm belongs to the another B(pin) of the germinal B(pin) unit. The another peak at 33.04 ppm is the peak for the in situ generated vinyl B(pin) intermediate (IM-7). This can be explained by the instant formation of IM-7 due to the very short reaction time. However after 5 mints, we performed the reaction work-up and recorded the boron NMR of the crude reaction mixture where we found only the peak at 33.04 ppm which is belongs to the intermediate IM-7. This confirm the reaction pathway towards the exclusive 1,2-addition.

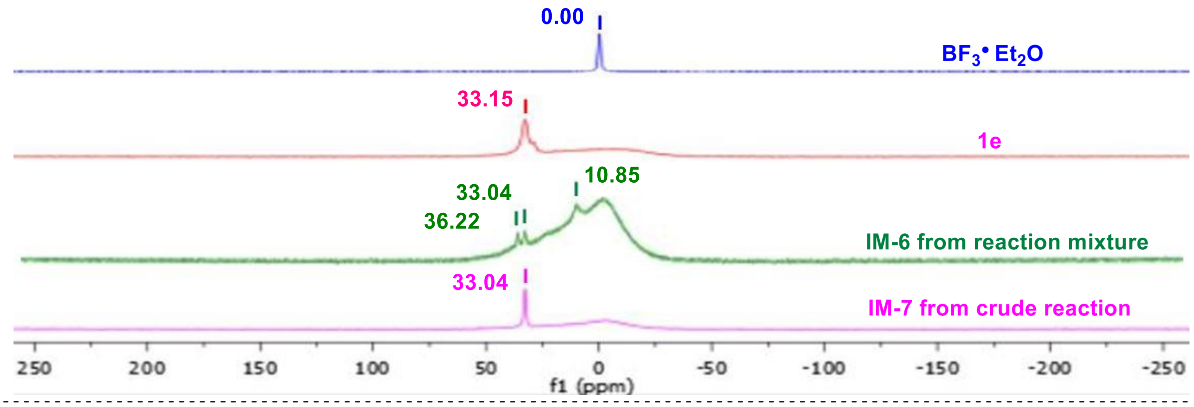

f. ^{11}B NMR spectrum

Supplementary figure 25. Boron NMR study

17. Other result:

In our method the compatablity of any functional group depends on the three stages. Step 1: Successful quinketal formation and unreactive towards the in situ formed acetic acid. Step 2: Unreactive towards the lithiated germinal B(pin). Step 3: Stable under the condition of the in situ formed hydrogen peroxide mediated oxidation. Here are the details of some substrates which failed under our reaction condition.

Substrates	Reason
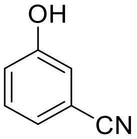 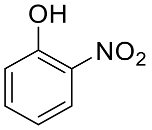	Formation of the quinketals found to be messy without any distinct spot.
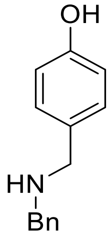	Non-separable multiple spots formed while synthesizing quinketals.
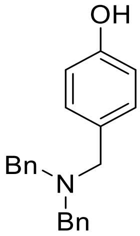	Non-separable multiple spots formed while synthesizing quinketals.
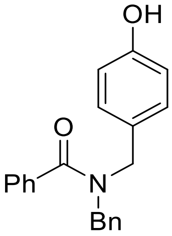	Formation of the quinketals found to be messy without any distinct spot.
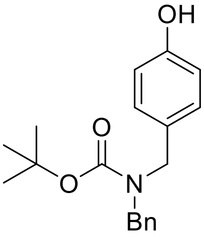	After sodium perborate oxidation, a non-separable impurity found to observe along with desire product with low yield.
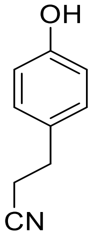	For this compound, although we got the NMR of the desire product but we did not get the unit mass both by HRMS and GC-MS. Here are all the data provided (sp-kkd-4052)

Supplementary Table 14: Unsuccessful substrates.

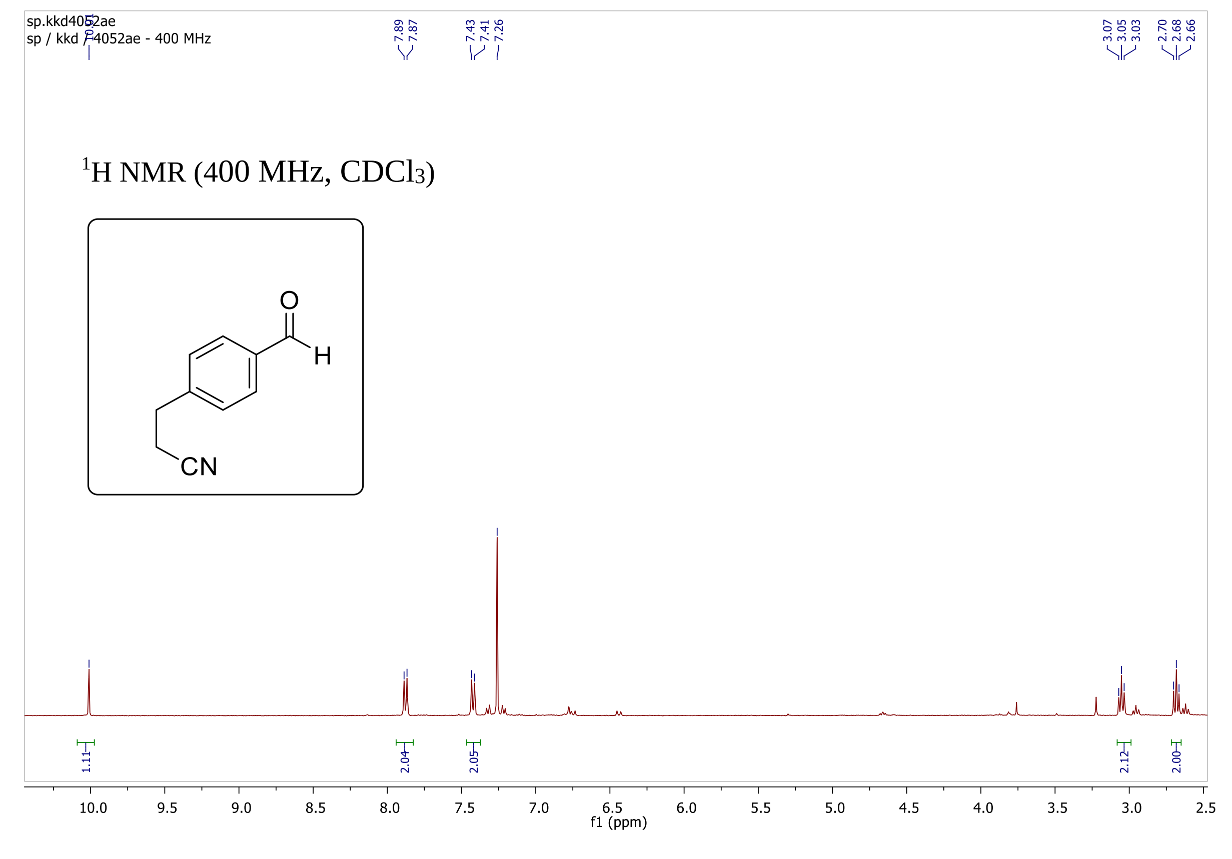
sp.kkd4052ae
sp / kkd / 4052ae - 400 MHz
7.89
7.87
7.43
7.41
7.26
3.07
3.05
3.03
2.70
2.68
2.66
1H NMR (400 MHz, CDCl3)
O
H
CN
1.11
2.04
2.05
2.12
2.00
10.0
9.5
9.0
8.5
8.0
7.5
7.0
6.5
6.0
5.5
5.0
4.5
4.0
3.5
3.0
2.5
f1 (ppm)

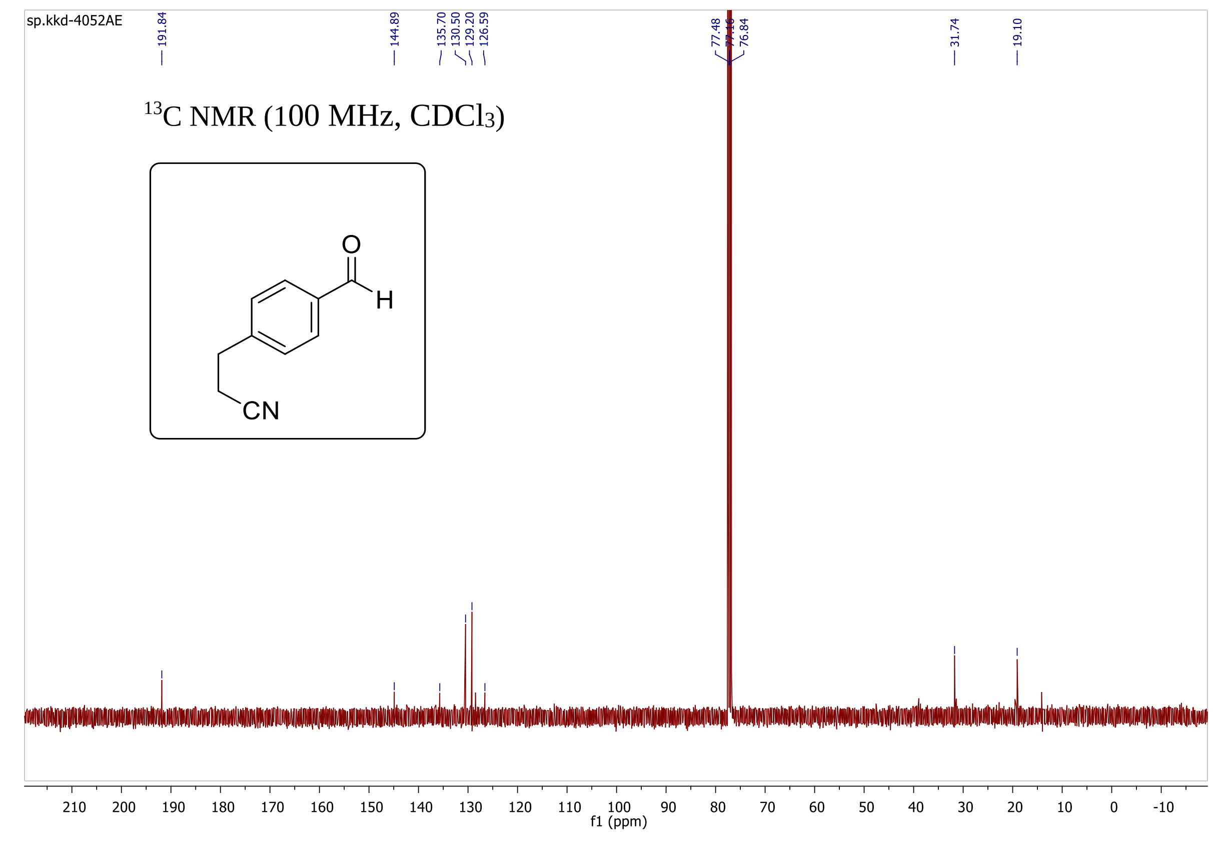
sp.kkd-4052AE
191.84
144.89
135.70
130.50
129.20
126.59
77.48
76.84
31.74
19.10
13C NMR (100 MHz, CDCl3)
O
H
CN
210
200
190
180
170
160
150
140
130
120
110
100
90
80
70
60
50
40
30
20
10
0
-10
f1 (ppm)

17. NMR spectrum

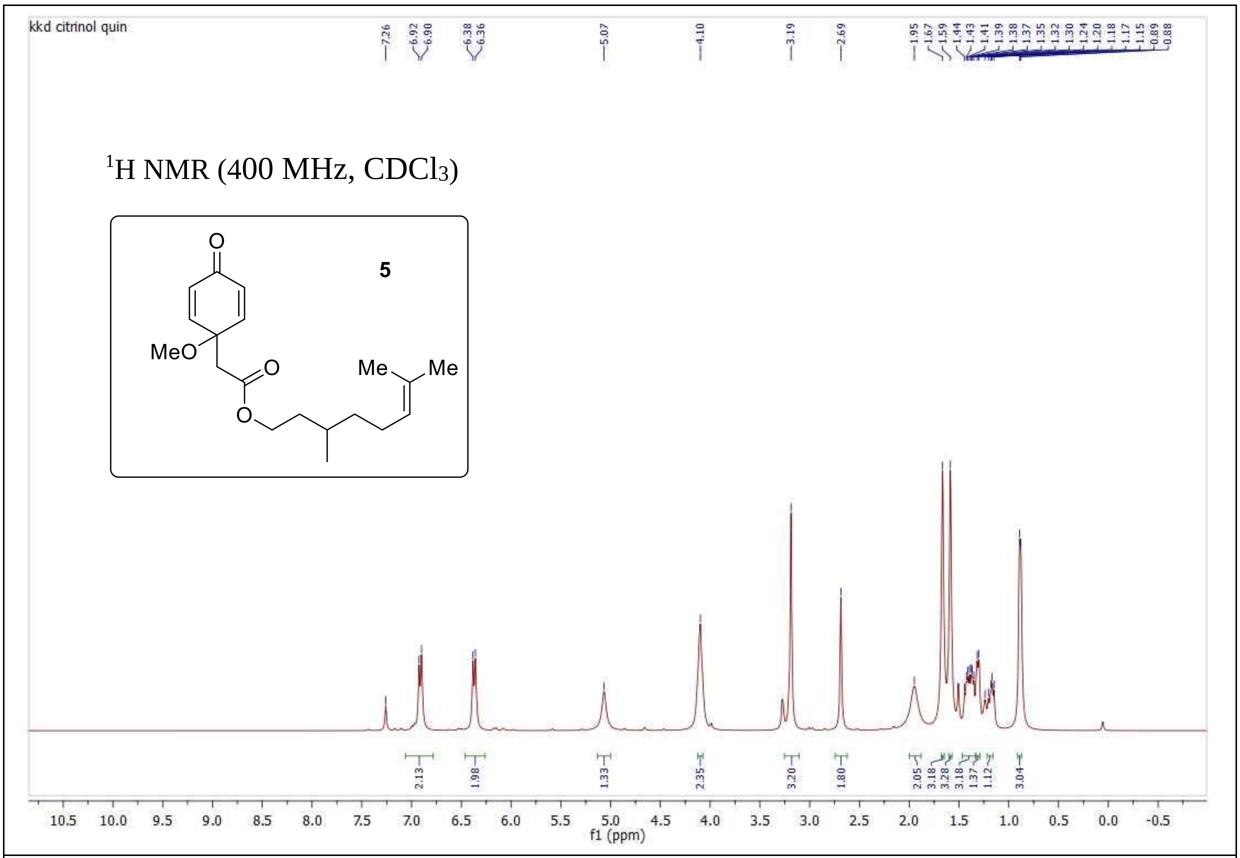

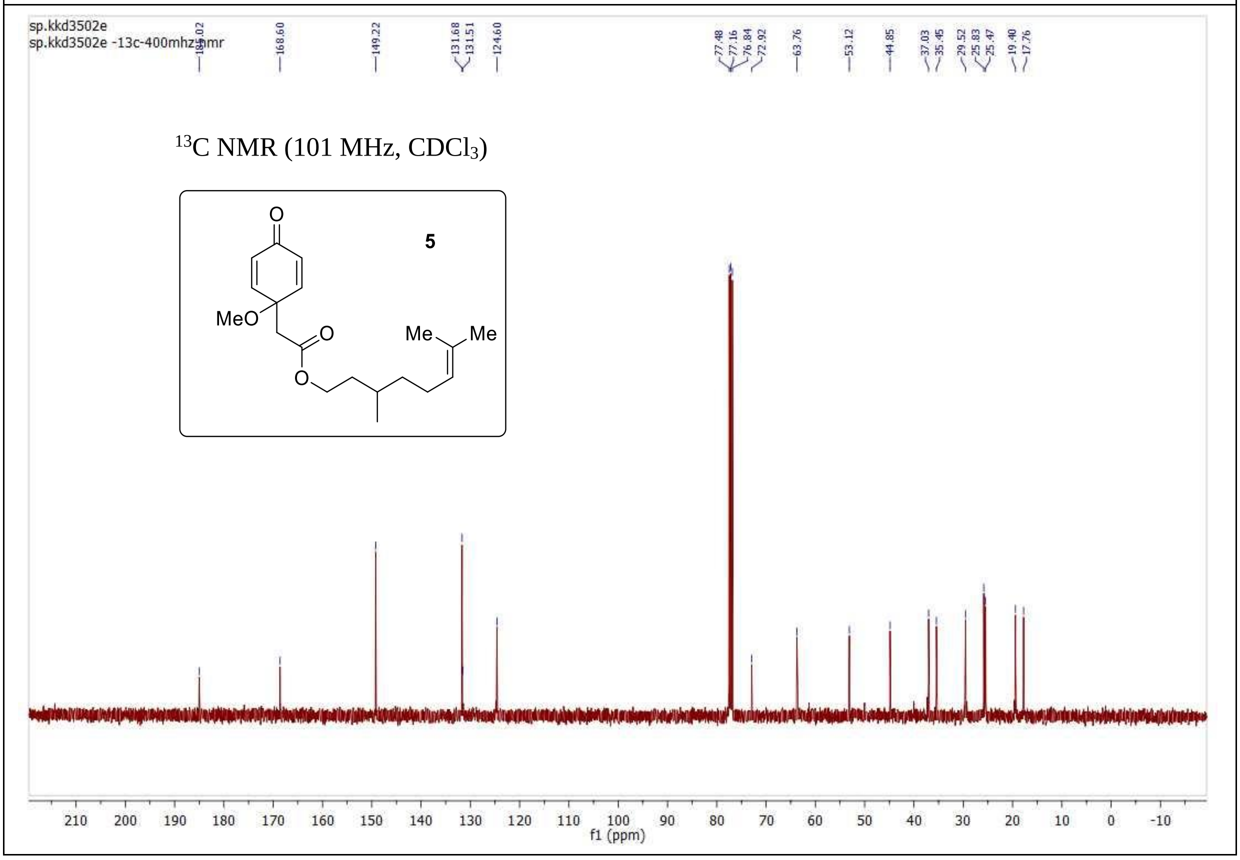

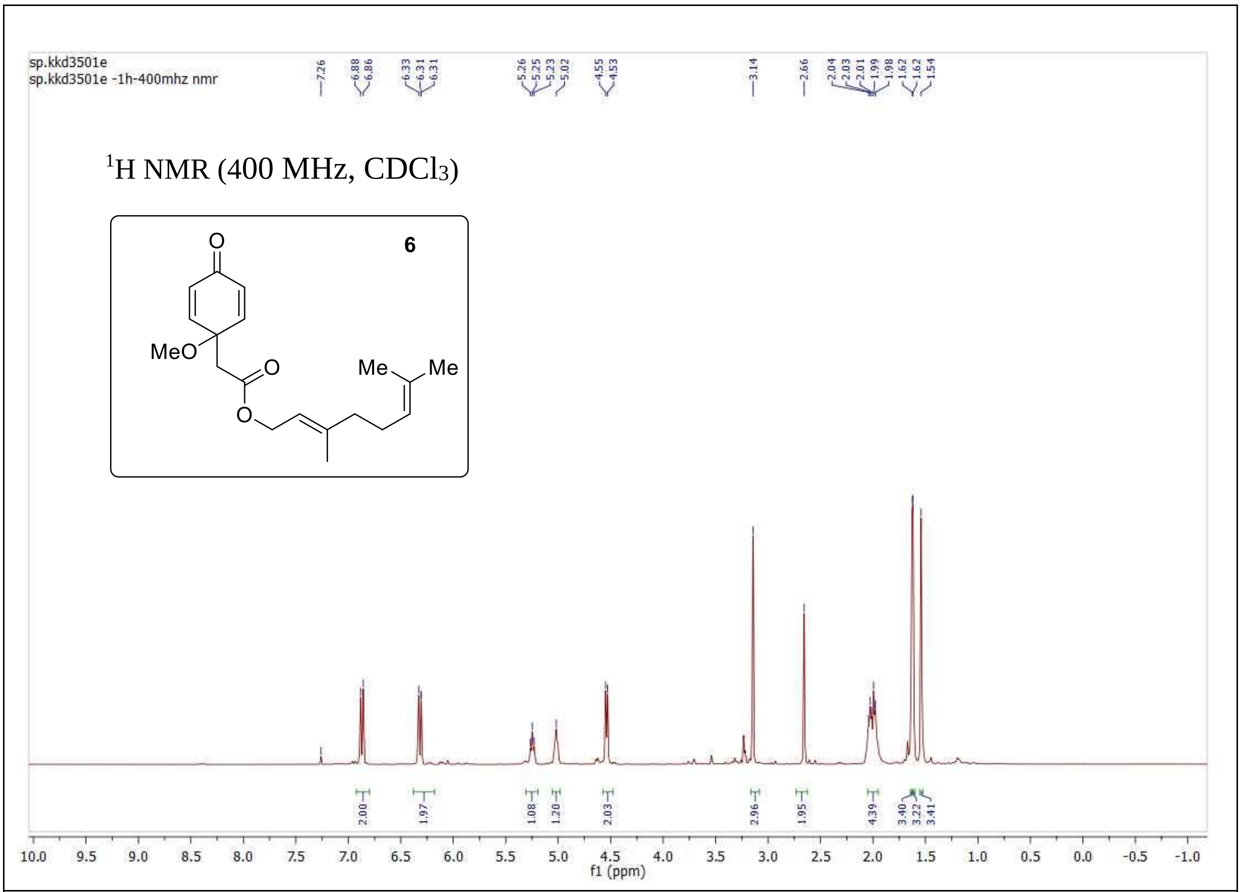
sp.kkd3501e
sp.kkd3501e -1h-400mhz nmr
7.26
6.88 6.86
6.33 6.31 6.31
5.26 5.25 5.23 5.02
4.55 4.53
3.14
2.66
2.04 2.03 2.01 1.99 1.98 1.62 1.62 1.54
1H NMR (400 MHz, CDCl3)
6
O
MeO
O
O
Me
Me
2.00
1.97
1.08
1.20
2.03
2.96
1.95
4.39
3.40 3.22 3.41
10.0 9.5 9.0 8.5 8.0 7.5 7.0 6.5 6.0 5.5 5.0 4.5 4.0 3.5 3.0 2.5 2.0 1.5 1.0 0.5 0.0 -0.5 -1.0
f1 (ppm)

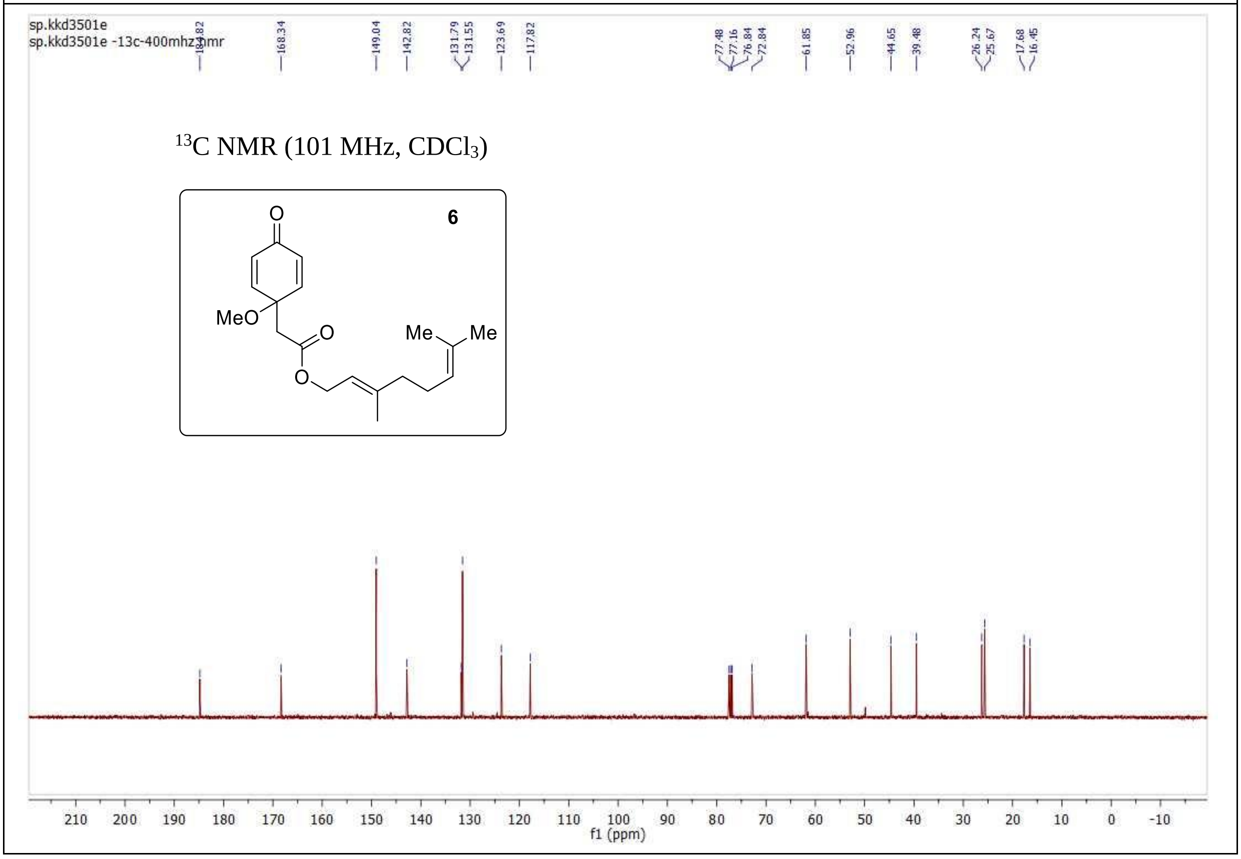
sp.kkd3501e
sp.kkd3501e -13c-400mhz nmr
183.82
168.34
149.04
142.82
131.79 131.55
123.69
117.82
77.48 77.16 76.84 72.84
61.85
52.96
44.65
39.48
26.24 25.67
17.68 16.45
13C NMR (101 MHz, CDCl3)
6
O
MeO
O
O
Me
Me
210 200 190 180 170 160 150 140 130 120 110 100 90 80 70 60 50 40 30 20 10 0 -10
f1 (ppm)

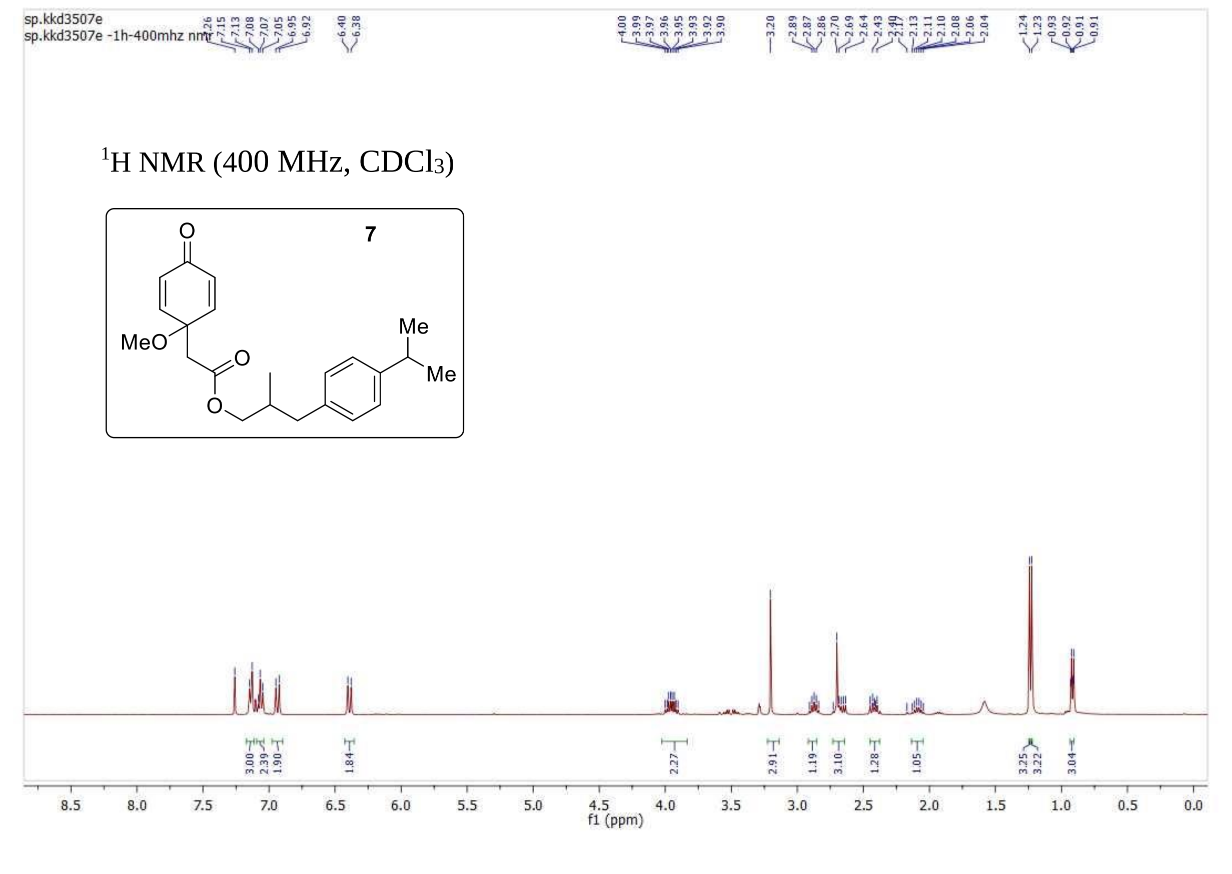

^{1}H NMR (400 MHz, CDCl3)
7
MeO
Me
Me

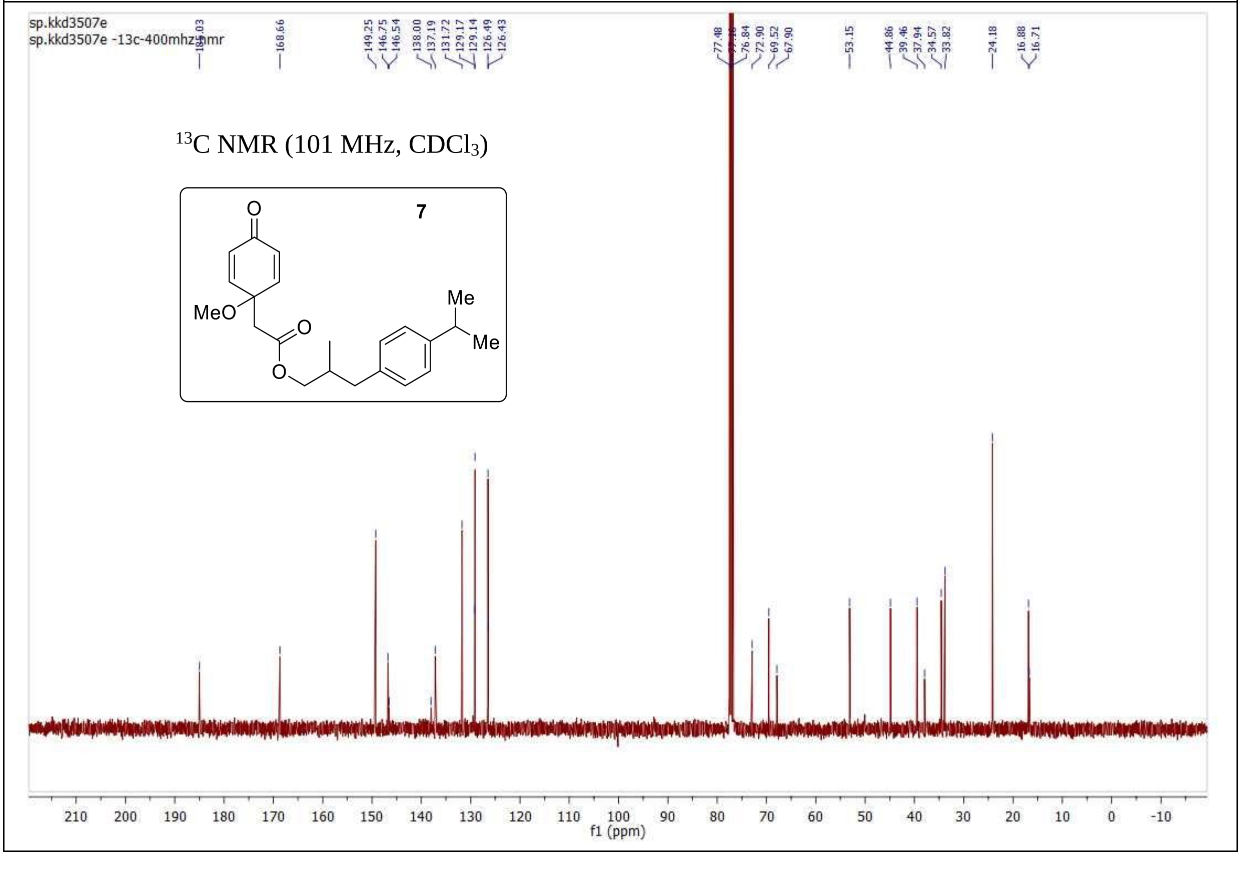

^{13}C NMR (101 MHz, CDCl3)
7
MeO
Me
Me

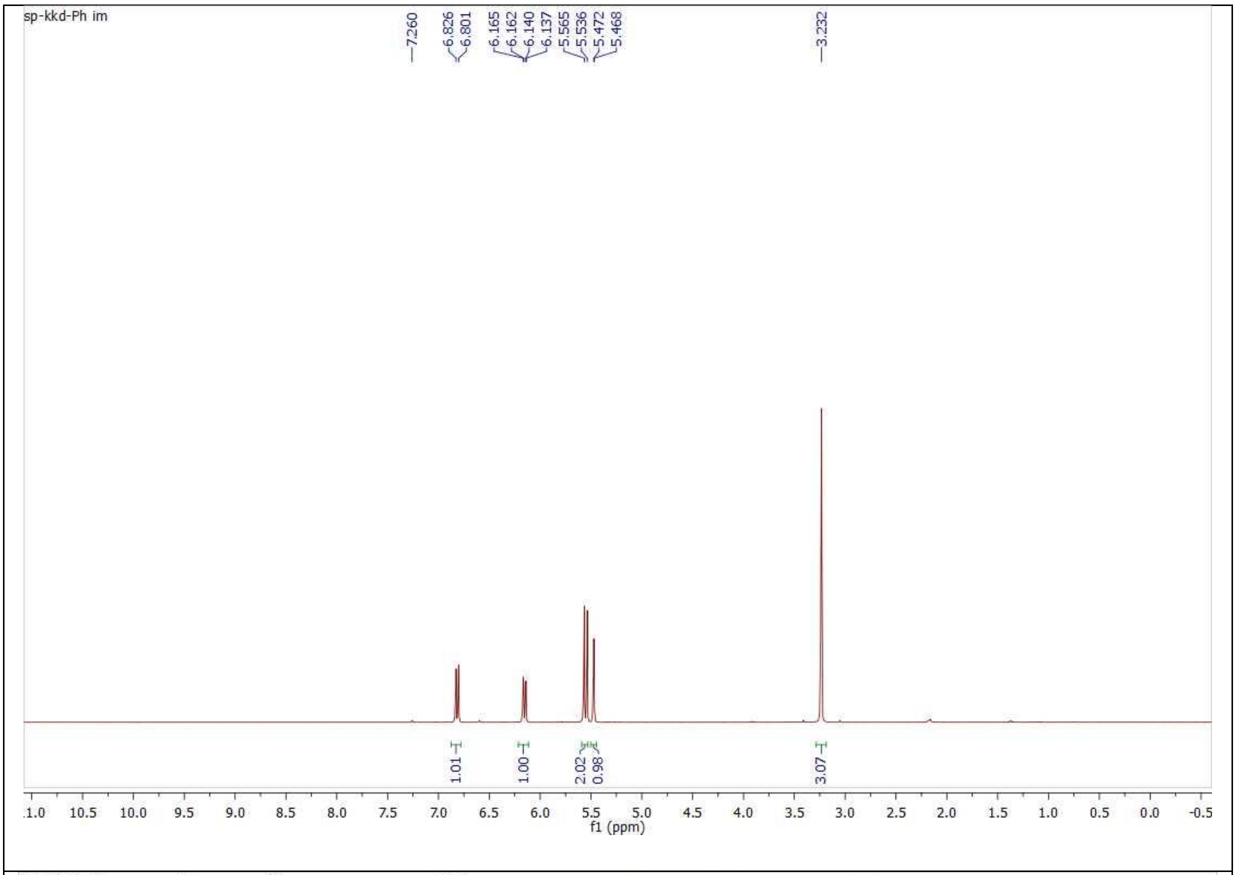
sp-kkd-Ph im
7.260
6.826
6.801
6.165
6.162
6.140
6.137
5.565
5.536
5.472
5.468
3.232
1.01
1.00
2.02
0.98
3.07
f1 (ppm)

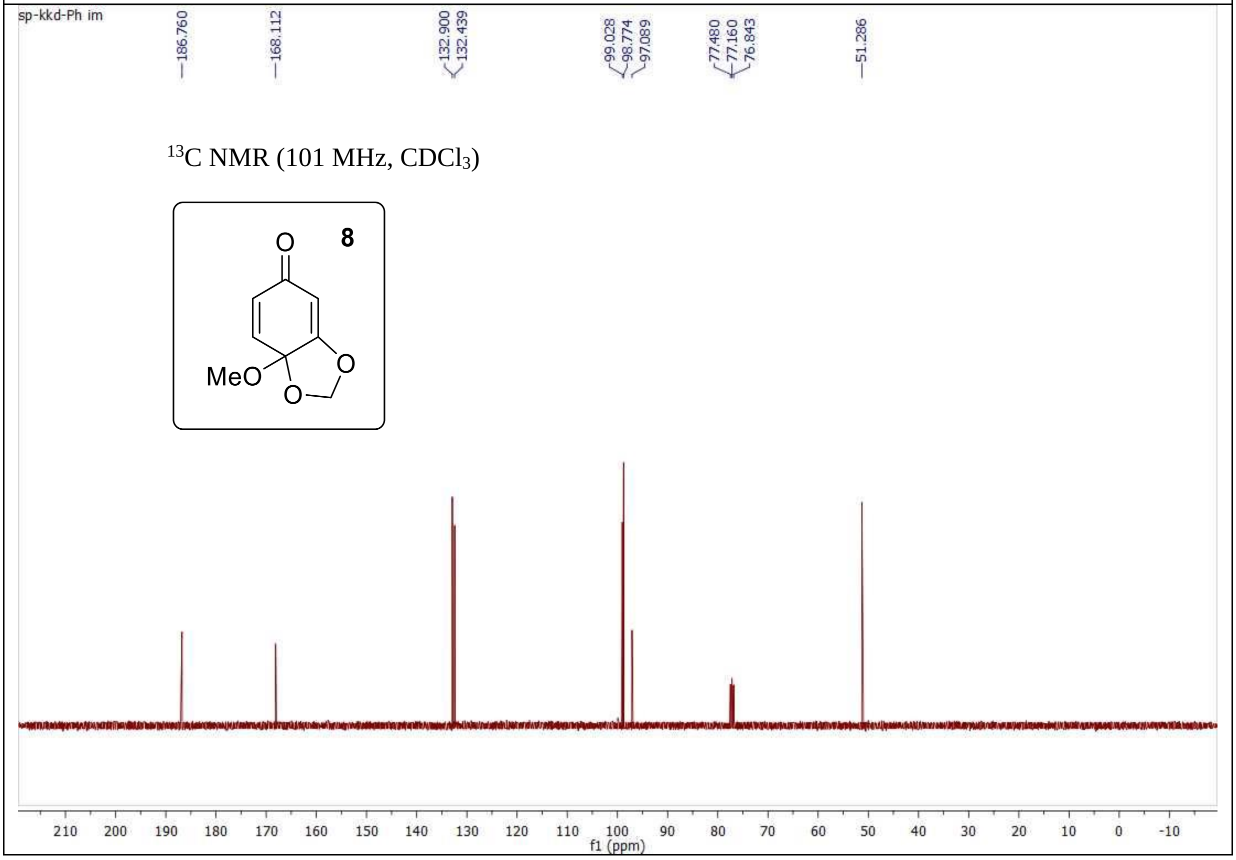
sp-kkd-Ph im
186.760
168.112
132.900
132.439
99.028
98.774
97.089
77.480
77.160
76.843
51.286
13C NMR (101 MHz, CDCl3)
8
O
MeO
O
O
f1 (ppm)

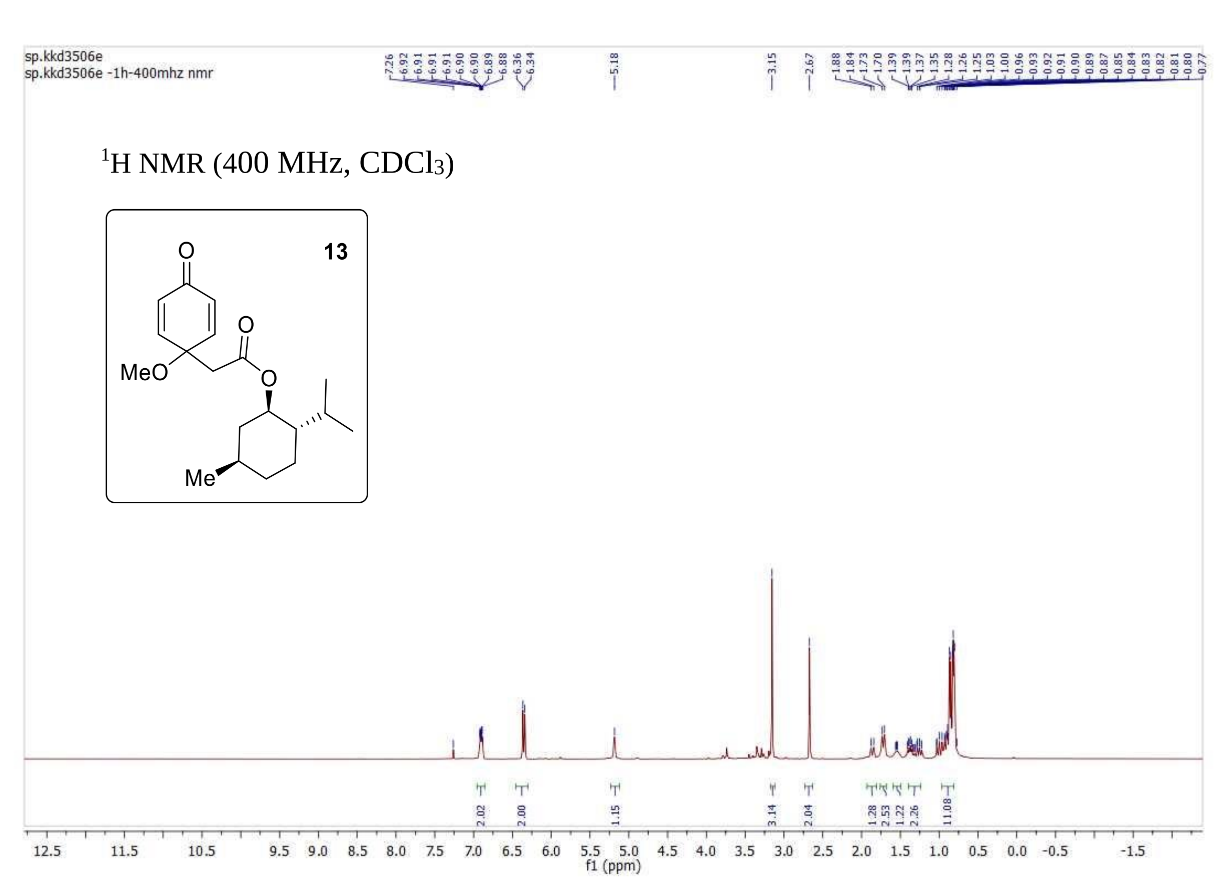
sp.kkd3506e
sp.kkd3506e -1h-400mhz nmr
¹H NMR (400 MHz, CDCl3)
13
MeO
O
Me
f1 (ppm)

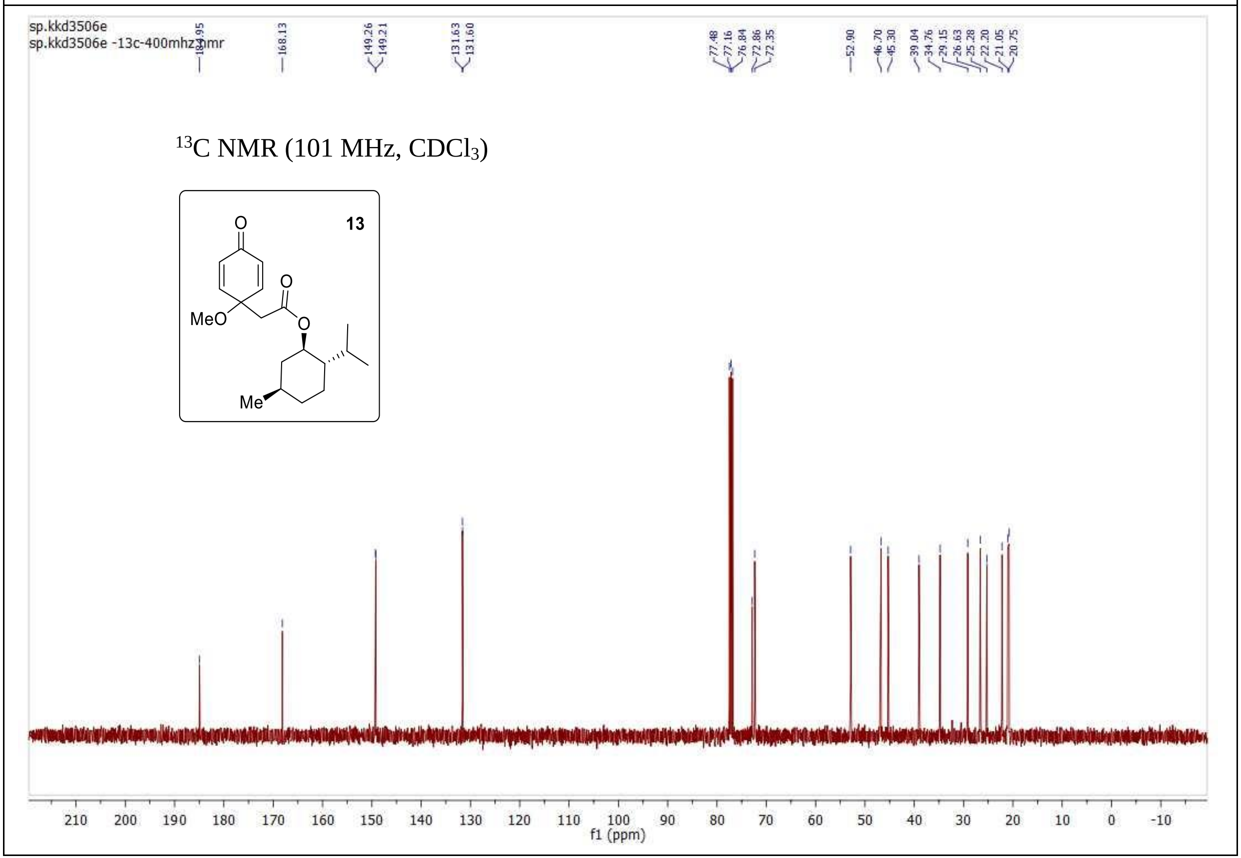
sp.kkd3506e
sp.kkd3506e -13c-400mhz nmr
¹³C NMR (101 MHz, CDCl3)
13
MeO
O
Me
f1 (ppm)

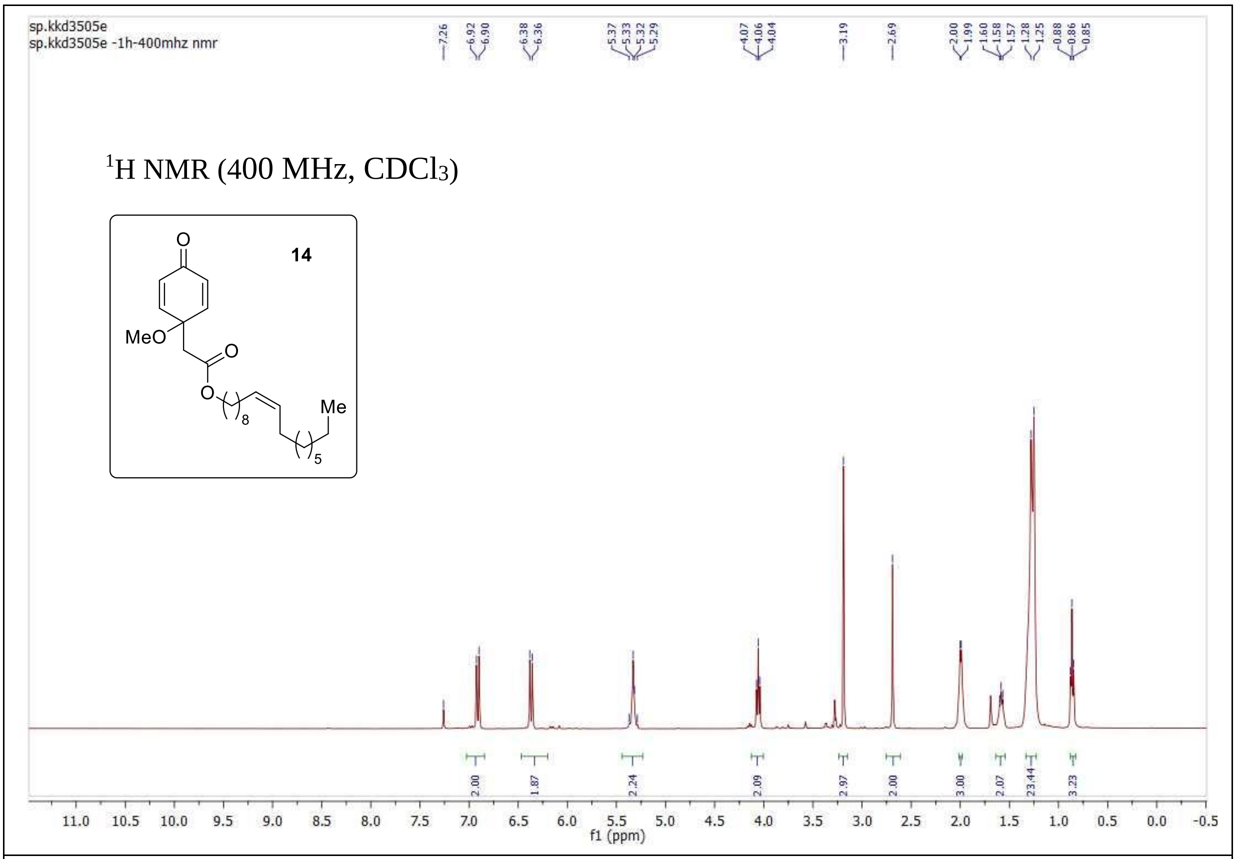
sp.kkd3505e
sp.kkd3505e -1h-400mhz nmr
1H NMR (400 MHz, CDCl3)
14
MeO
Me
7.26
6.92
6.90
6.38
6.36
5.37
5.33
5.32
5.29
4.07
4.06
4.04
3.19
2.69
2.00
1.99
1.60
1.58
1.57
1.28
1.25
0.88
0.86
0.85
2.00
1.87
2.24
2.09
2.97
2.00
3.00
2.07
23.44
3.23
f1 (ppm)

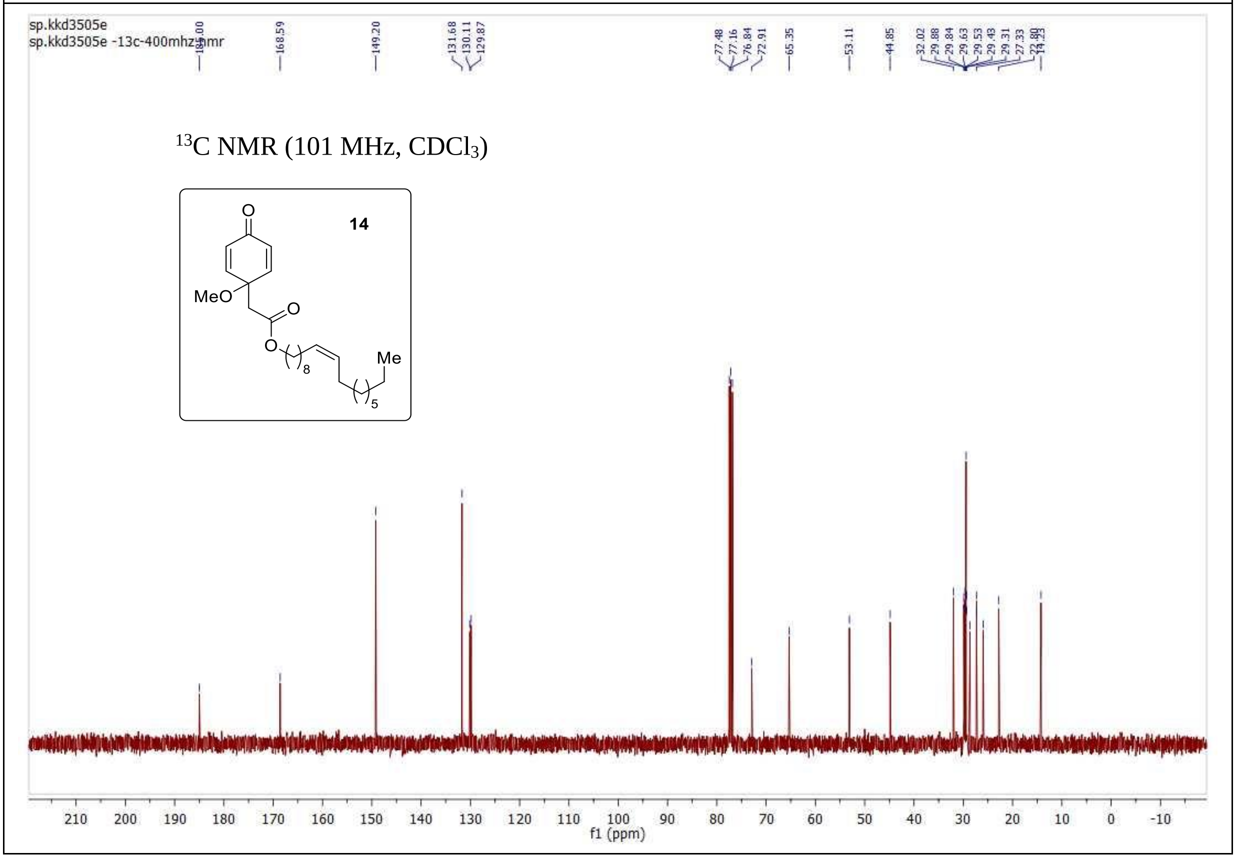
sp.kkd3505e
sp.kkd3505e -13c-400mhz nmr
13C NMR (101 MHz, CDCl3)
14
MeO
Me
168.59
149.20
131.68
130.11
129.87
77.48
77.16
76.84
72.91
65.35
53.11
44.85
32.02
29.88
29.84
29.63
29.53
29.43
29.31
27.33
f1 (ppm)

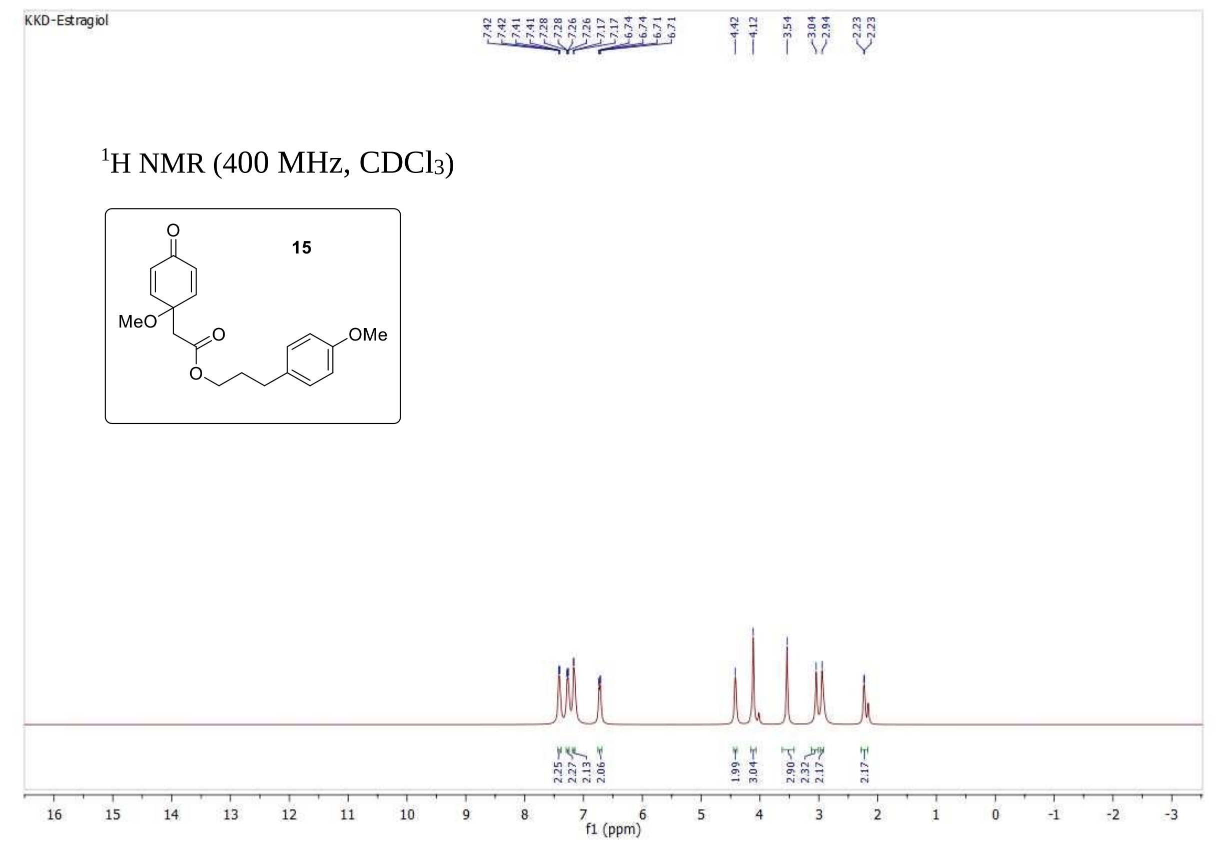
KKD-Estragiol
7.42 7.42 7.41 7.41 7.28 7.28 7.26 7.26 7.17 7.17 6.74 6.74 6.71 6.71
4.42 4.12 3.54 3.04 2.94 2.23 2.23
1H NMR (400 MHz, CDCl3)
15
O
MeO
O
O
OMe
2.25 2.27 2.13 2.06 1.99 3.04 2.90 2.32 2.17 2.17
f1 (ppm)

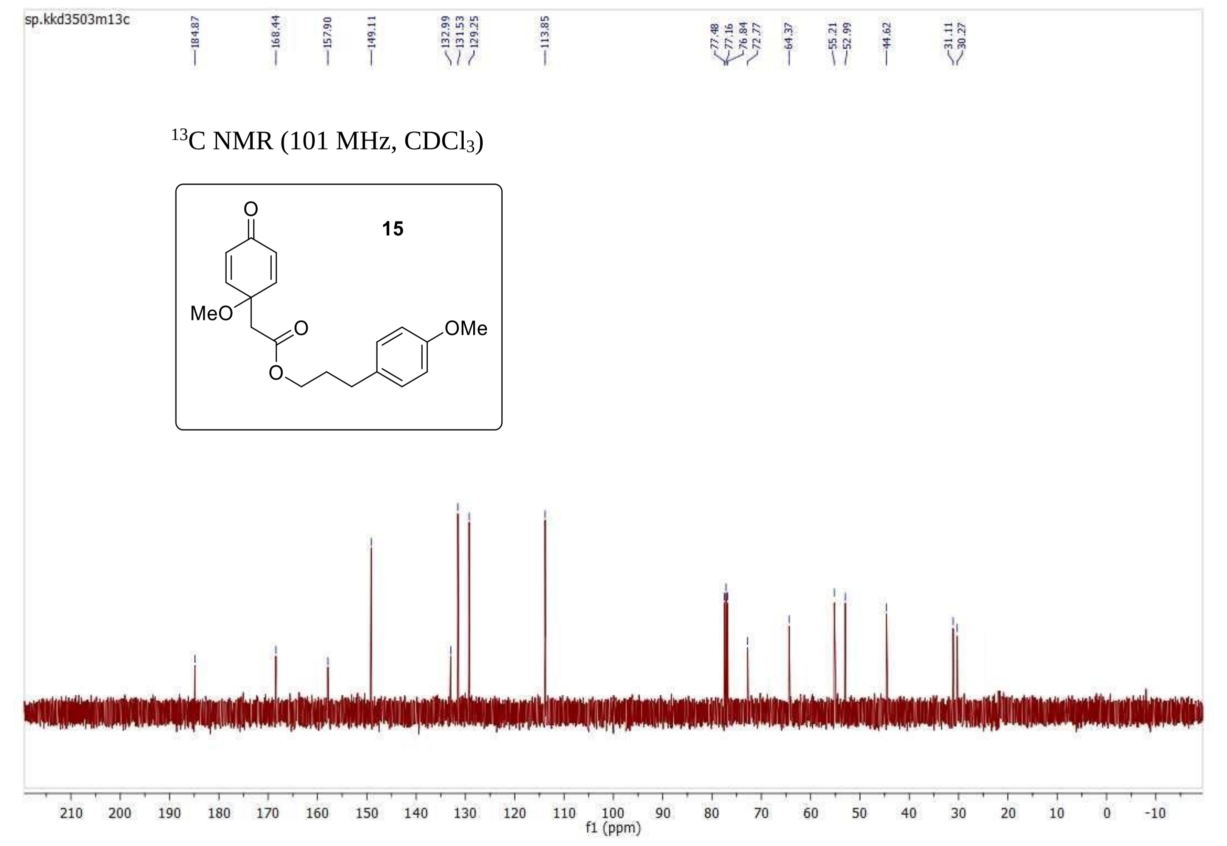
sp.kkd3503m13c
184.87 168.44 157.90 149.11 132.99 131.53 129.25 113.85 77.48 77.16 76.84 72.77 64.37 55.21 52.99 44.62 31.11 30.27
13C NMR (101 MHz, CDCl3)
15
O
MeO
O
O
OMe
f1 (ppm)

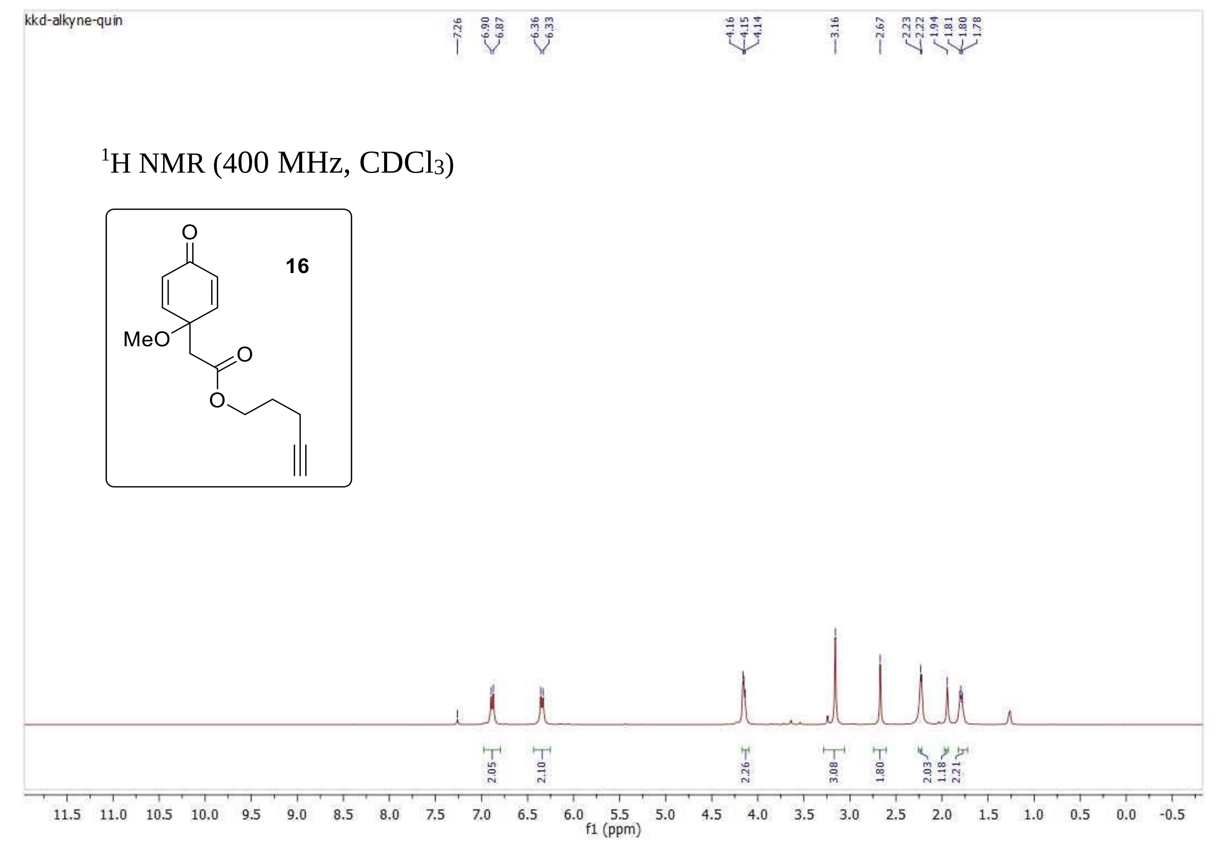
kkd-alkyne-quin
7.26
6.90
6.87
6.36
6.33
4.16
4.15
4.14
3.16
2.67
2.23
2.22
1.94
1.81
1.80
1.78
^{1}H NMR (400 MHz, CDCl$_3$)
16
MeO
O
2.05
2.10
2.26
3.08
1.80
2.03
1.18
2.21
f1 (ppm)

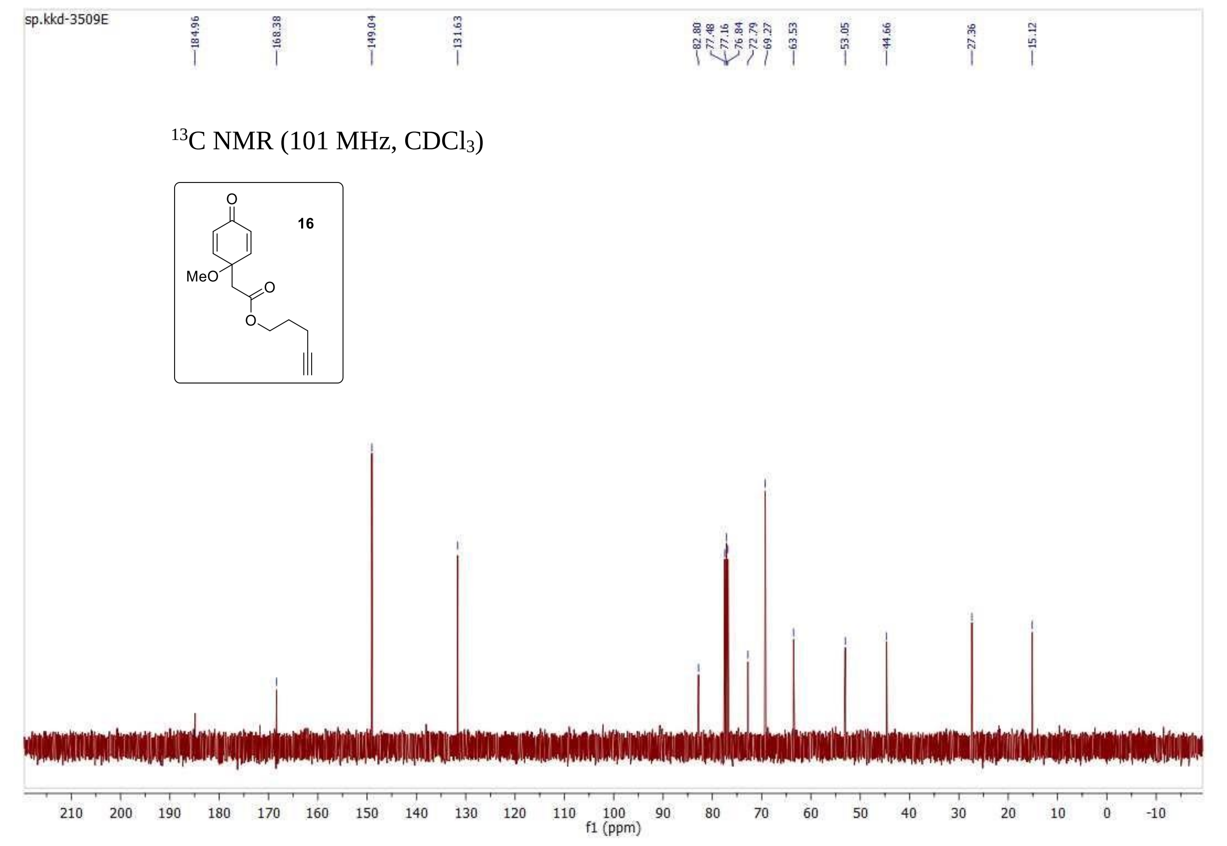
sp.kkd-3509E
184.96
168.38
149.04
131.63
82.80
77.48
77.16
76.84
72.79
69.27
63.53
53.05
44.66
27.36
15.12
^{13}C NMR (101 MHz, CDCl$_3$)
16
MeO
O
f1 (ppm)

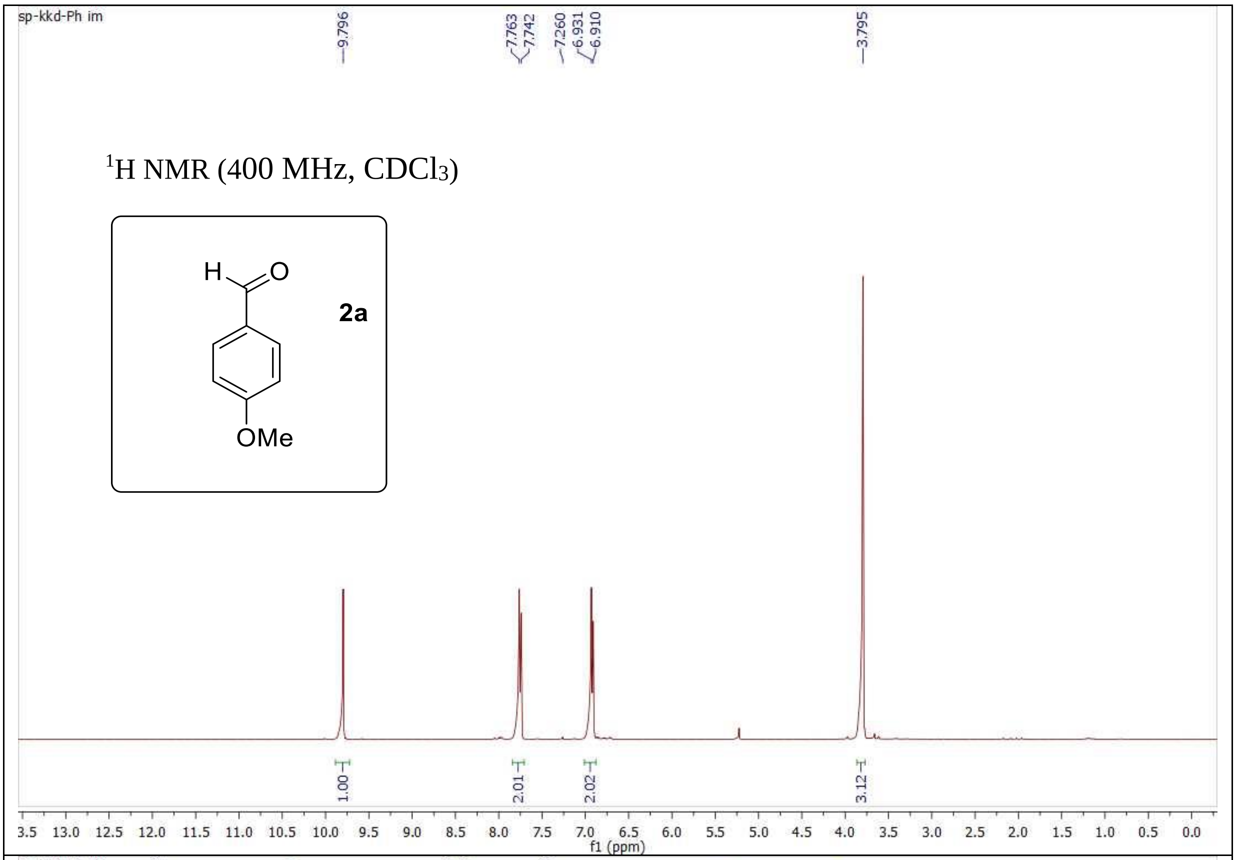
sp-kkd-Ph im
9.796
7.763
7.742
7.260
6.931
6.910
3.795
1H NMR (400 MHz, CDCl3)
H
O
2a
OMe
1.00
2.01
2.02
3.12
f1 (ppm)

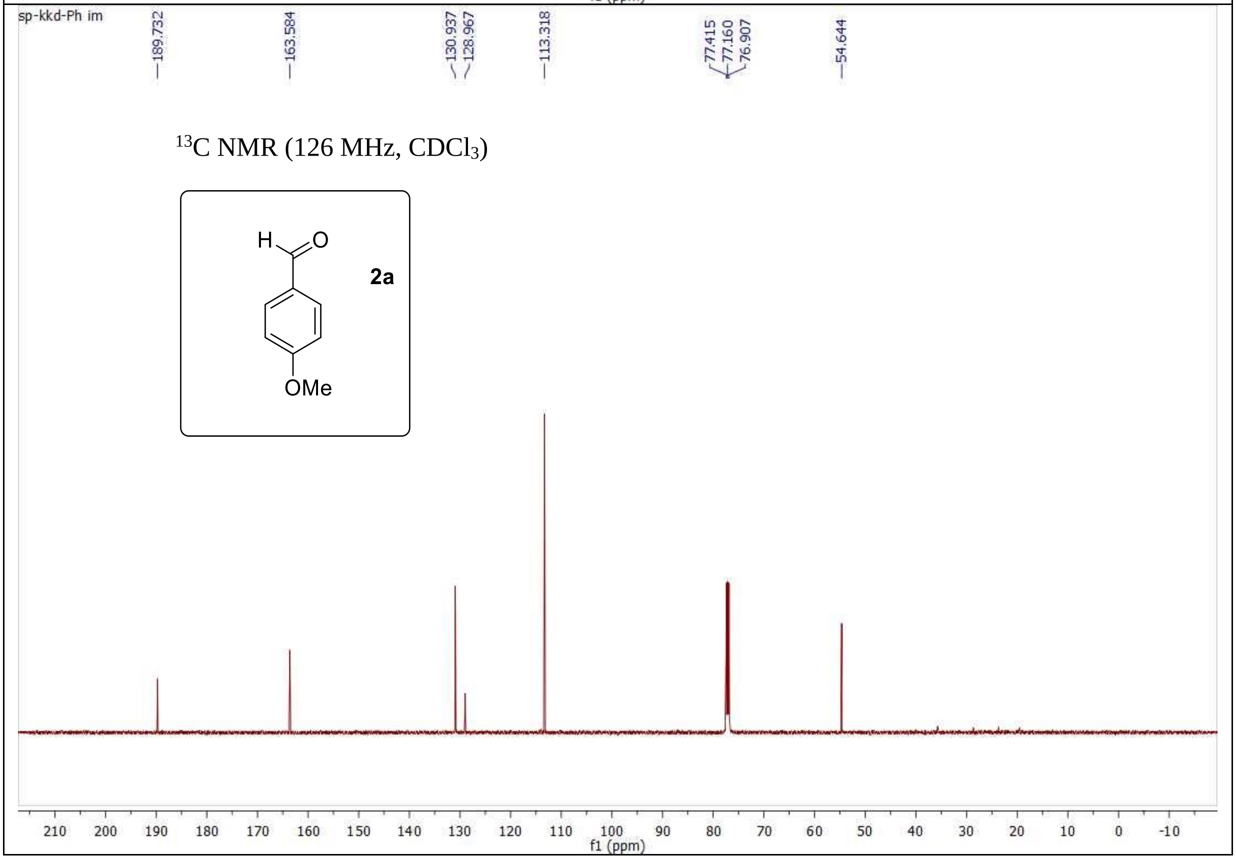
sp-kkd-Ph im
189.732
163.584
130.937
128.967
113.318
77.415
77.160
76.907
54.644
13C NMR (126 MHz, CDCl3)
H
O
2a
OMe
f1 (ppm)

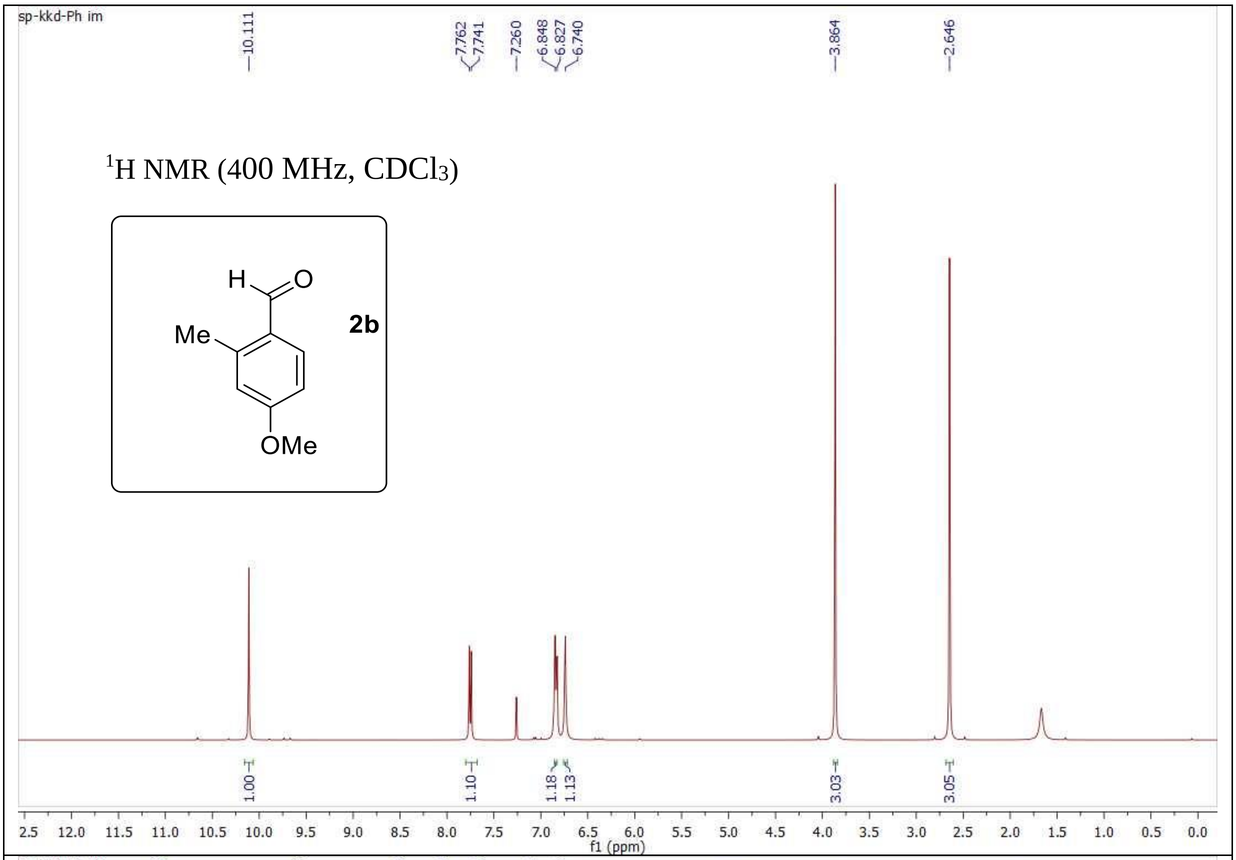
sp-kkd-Ph im
10.111
7.762
7.741
7.260
6.848
6.827
6.740
3.864
2.646
1H NMR (400 MHz, CDCl3)
H
O
Me
2b
OMe
1.00
1.10
1.18
1.13
3.03
3.05
f1 (ppm)

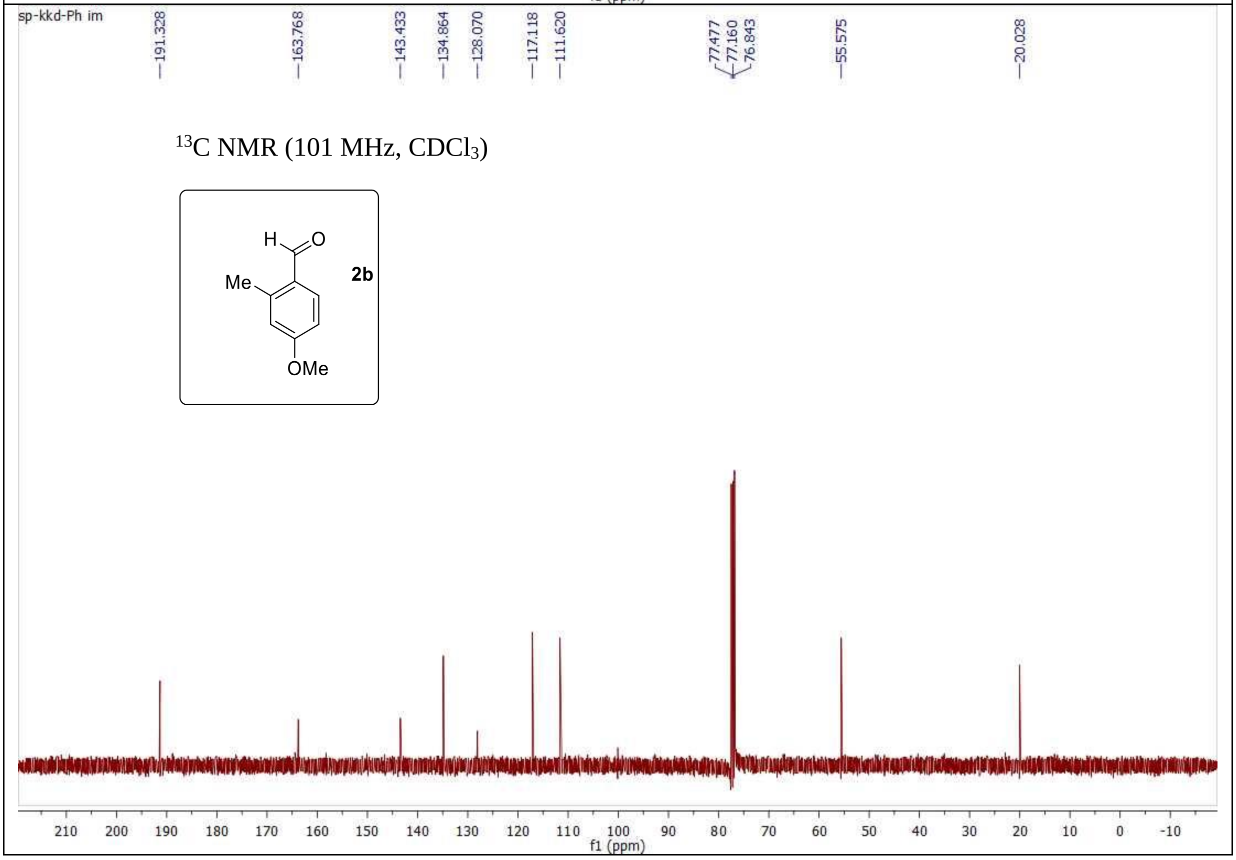
sp-kkd-Ph im
191.328
163.768
143.433
134.864
128.070
117.118
111.620
77.477
77.160
76.843
55.575
20.028
13C NMR (101 MHz, CDCl3)
H
O
Me
2b
OMe
f1 (ppm)

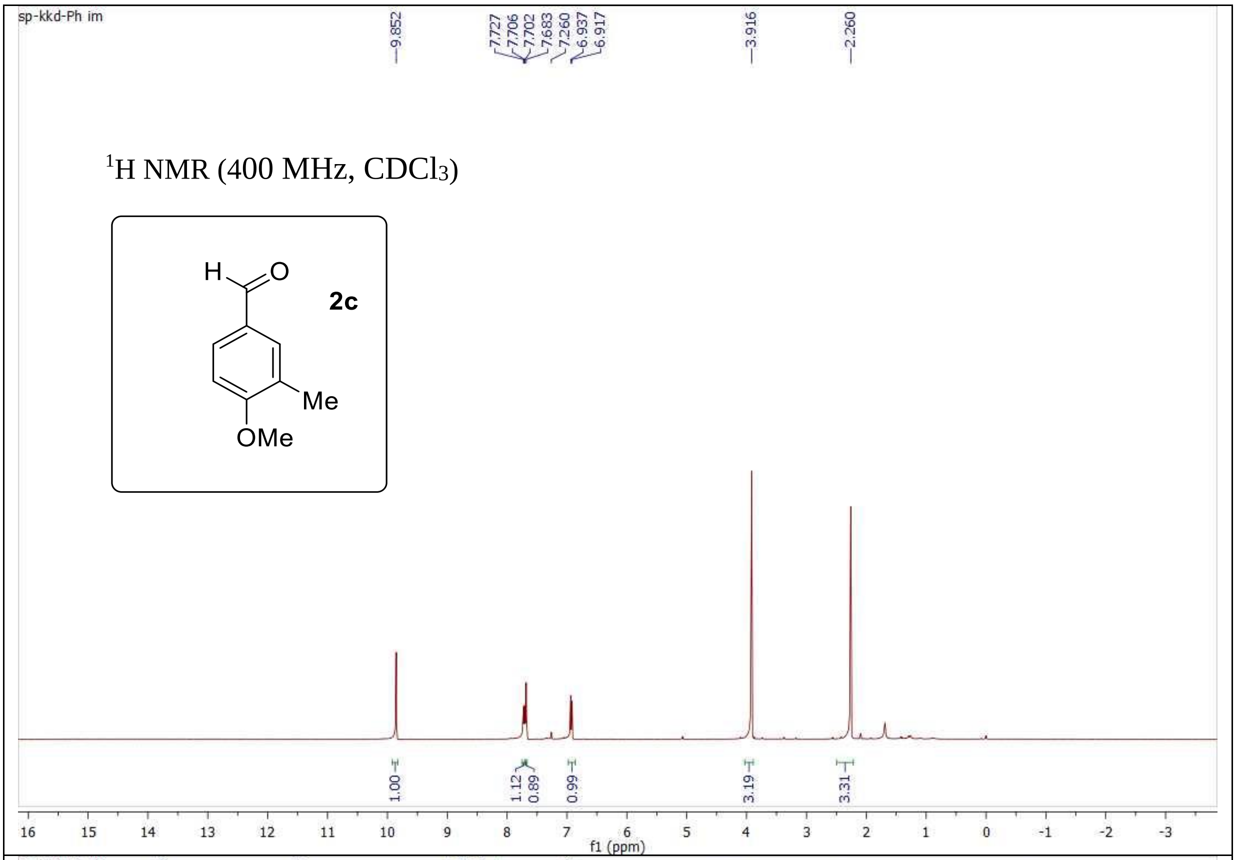
sp-kkd-Ph im
9.852
7.727
7.706
7.702
7.683
7.260
6.937
6.917
3.916
2.260
^{1}H NMR (400 MHz, $CDCl_3$)
H
O
2c
Me
OMe
1.00
1.12
0.89
0.99
3.19
3.31
f1 (ppm)

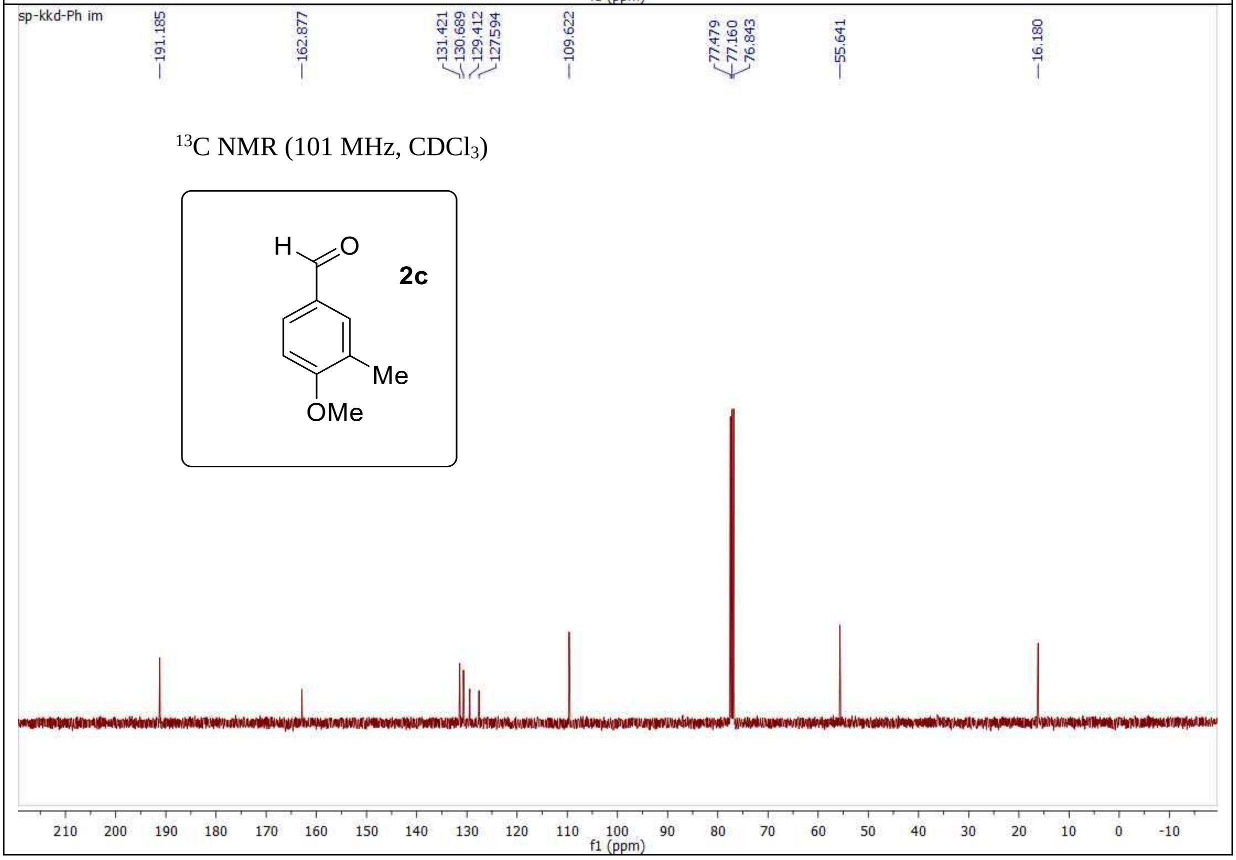
sp-kkd-Ph im
191.185
162.877
131.421
130.689
129.412
127594
109.622
77.479
77.160
76.843
55.641
16.180
^{13}C NMR (101 MHz, $CDCl_3$)
H
O
2c
Me
OMe
f1 (ppm)

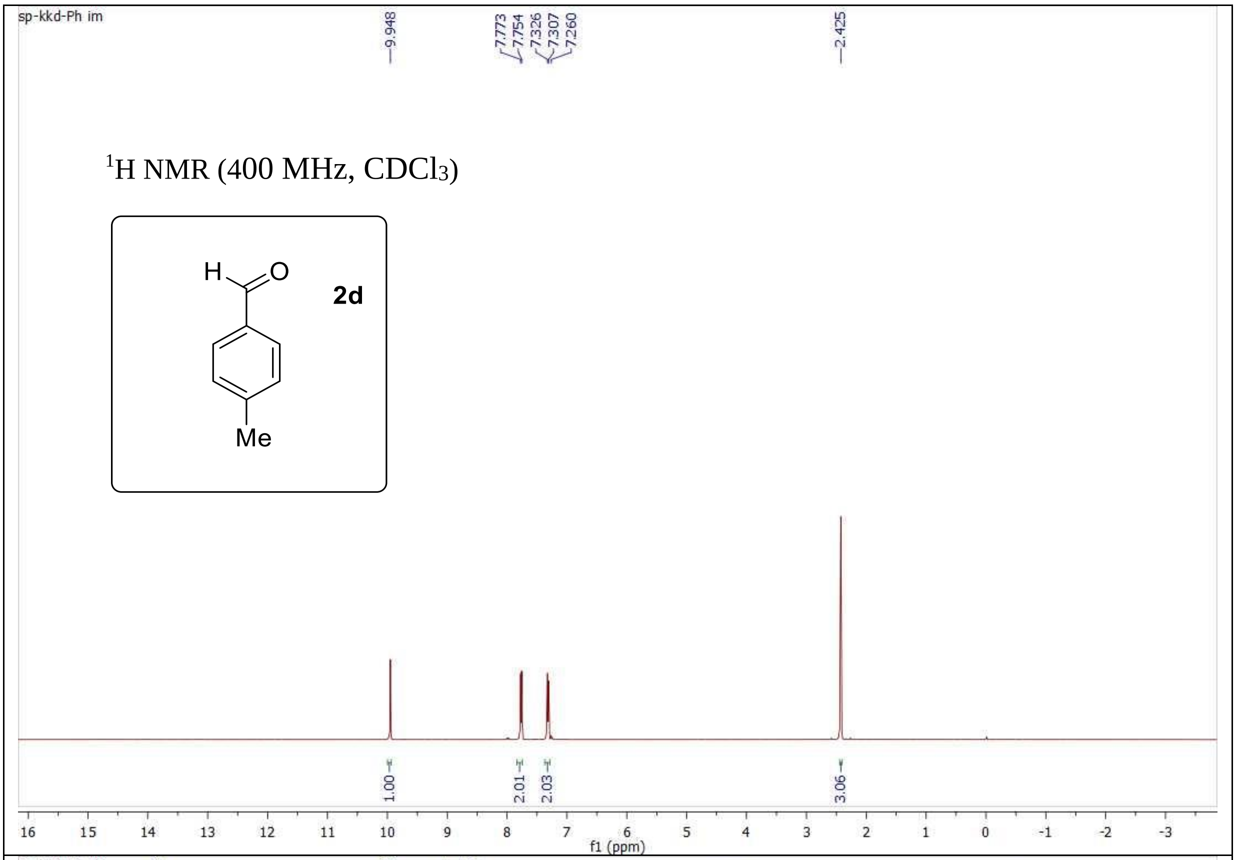
sp-kkd-Ph im
9.948
7.773
7.754
7.326
7.307
7.260
2.425
1H NMR (400 MHz, CDCl3)
H
O
2d
Me
1.00
2.01
2.03
3.06
16 15 14 13 12 11 10 9 8 7 6 5 4 3 2 1 0 -1 -2 -3
f1 (ppm)

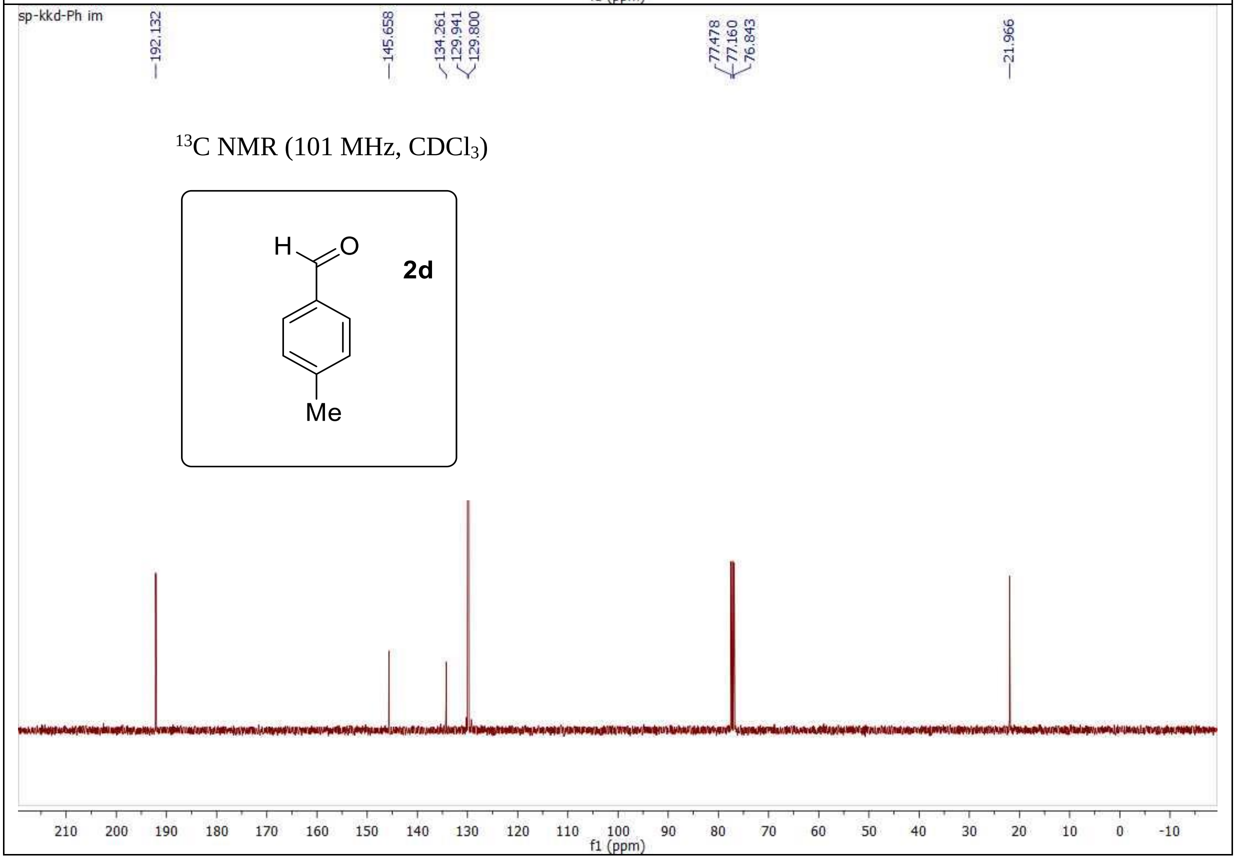
sp-kkd-Ph im
192.132
145.658
134.261
129.941
129.800
77.478
77.160
76.843
21.966
13C NMR (101 MHz, CDCl3)
H
O
2d
Me
210 200 190 180 170 160 150 140 130 120 110 100 90 80 70 60 50 40 30 20 10 0 -10
f1 (ppm)

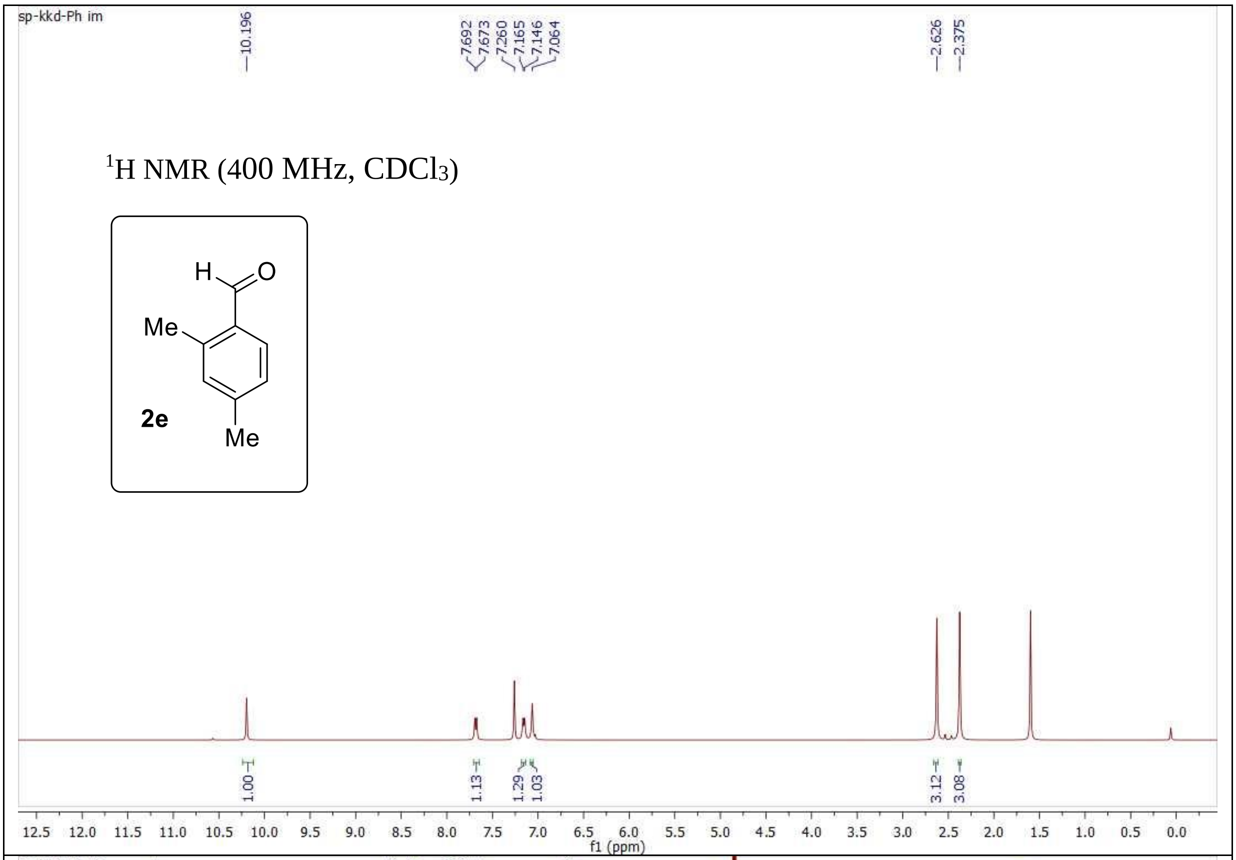
sp-kkd-Ph im
10.196
7.692
7.673
7.260
7.165
7.146
7.064
2.626
2.375
1H NMR (400 MHz, CDCl3)
H
O
Me
2e
Me
1.00
1.13
1.29
1.03
3.12
3.08
12.5 12.0 11.5 11.0 10.5 10.0 9.5 9.0 8.5 8.0 7.5 7.0 6.5 6.0 5.5 5.0 4.5 4.0 3.5 3.0 2.5 2.0 1.5 1.0 0.5 0.0
f1 (ppm)

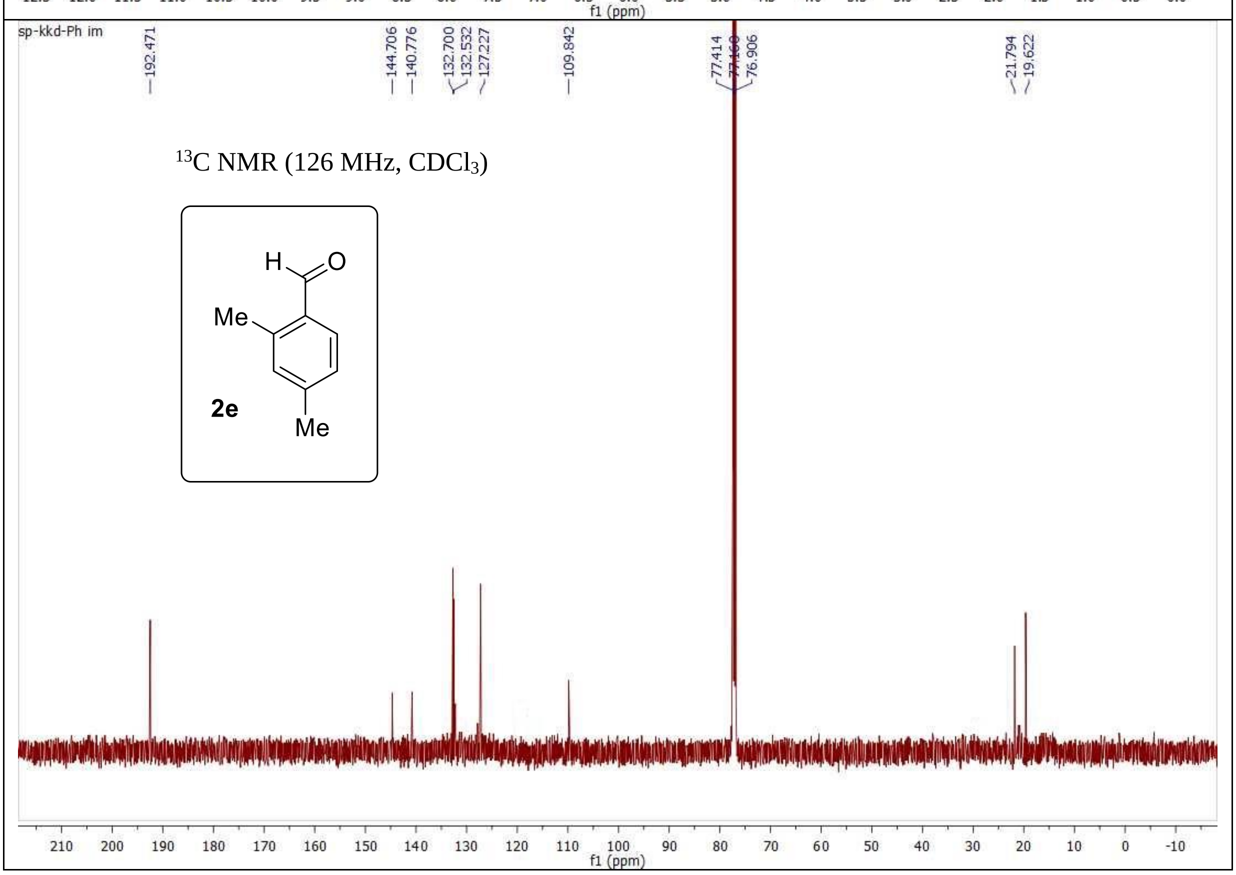
sp-kkd-Ph im
192.471
144.706
140.776
132.700
132.532
127.227
109.842
77.414
77.160
76.906
21.794
19.622
13C NMR (126 MHz, CDCl3)
H
O
Me
2e
Me
210 200 190 180 170 160 150 140 130 120 110 100 90 80 70 60 50 40 30 20 10 0 -10
f1 (ppm)

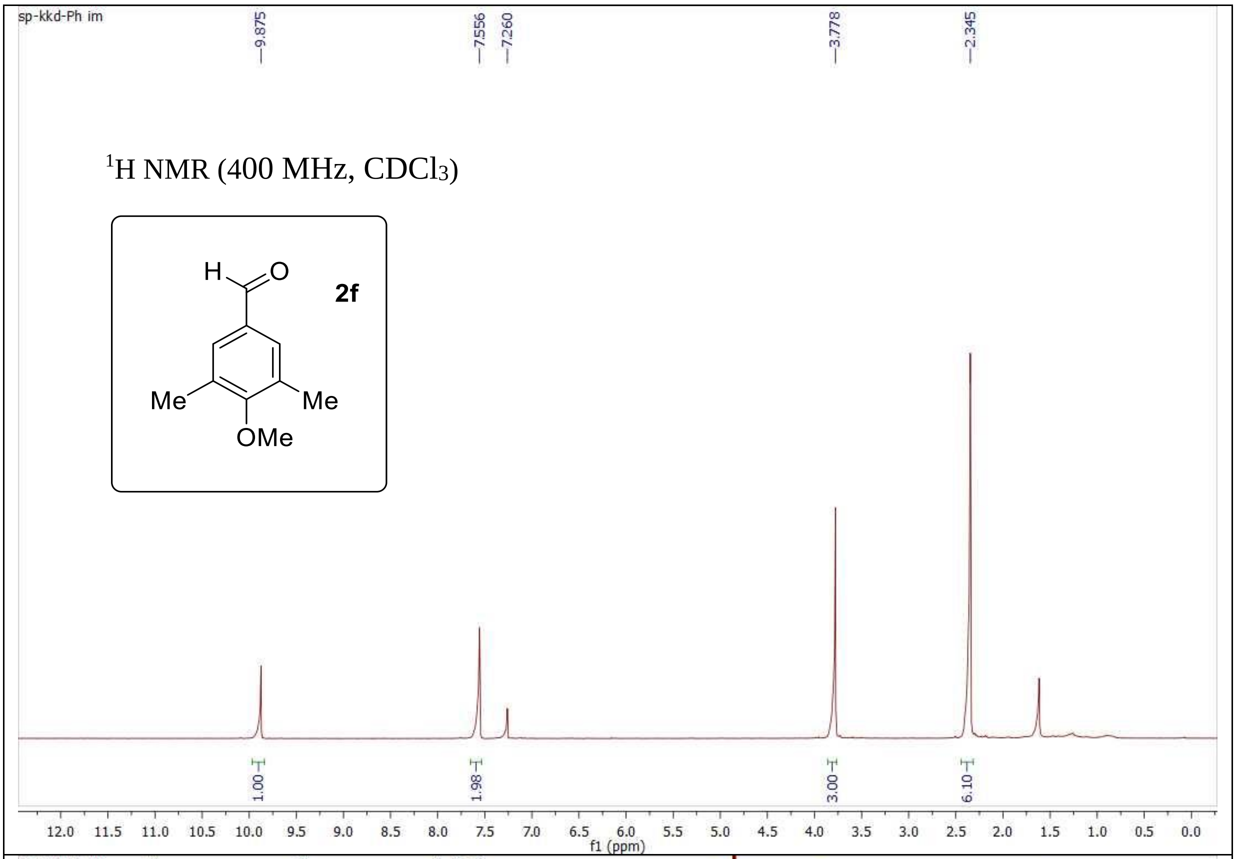
sp-kkd-Ph im
9.875
7.556
7.260
3.778
2.345
1H NMR (400 MHz, CDCl3)
H
O
2f
Me
Me
OMe
1.00
1.98
3.00
6.10
12.0 11.5 11.0 10.5 10.0 9.5 9.0 8.5 8.0 7.5 7.0 6.5 6.0 5.5 5.0 4.5 4.0 3.5 3.0 2.5 2.0 1.5 1.0 0.5 0.0
f1 (ppm)

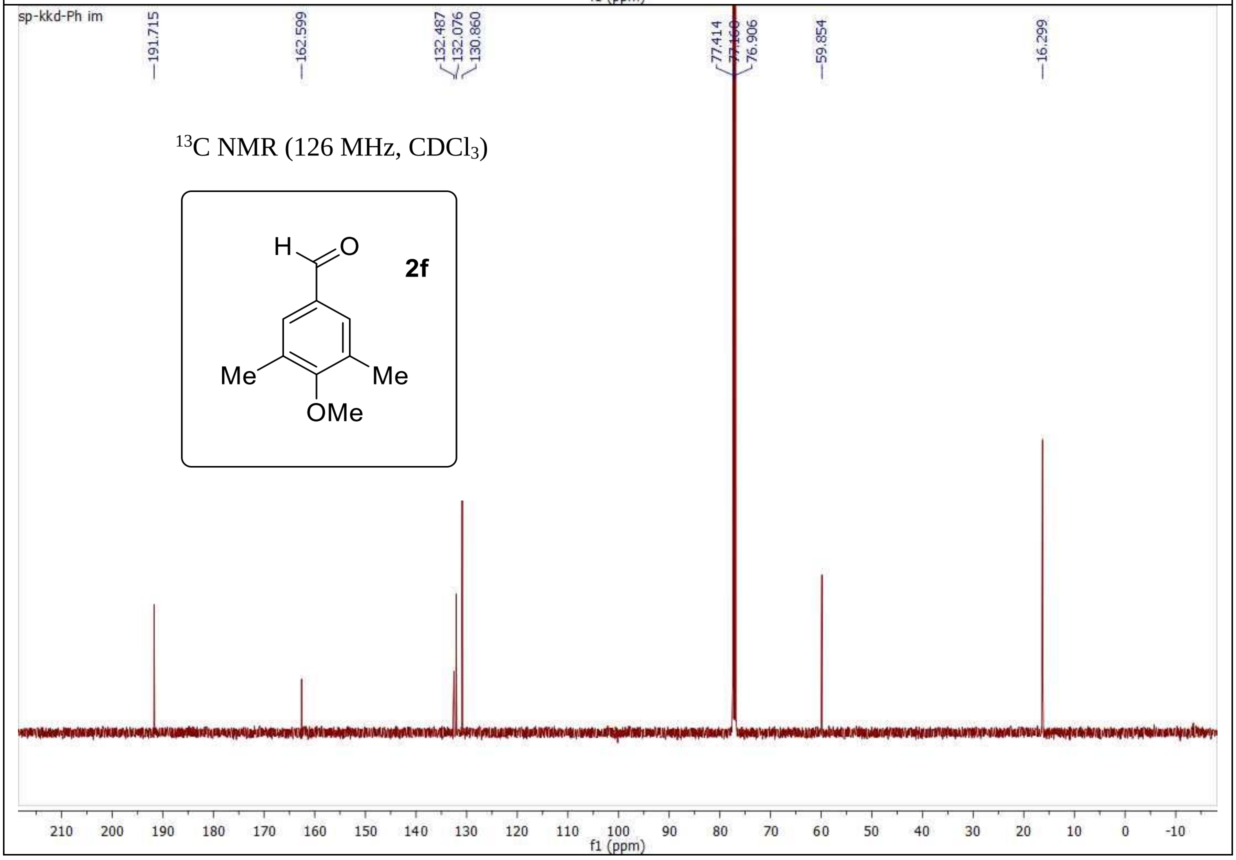
sp-kkd-Ph im
191.715
162.599
132.487
132.076
130.860
77.414
76.906
59.854
16.299
13C NMR (126 MHz, CDCl3)
H
O
2f
Me
Me
OMe
210 200 190 180 170 160 150 140 130 120 110 100 90 80 70 60 50 40 30 20 10 0 -10
f1 (ppm)

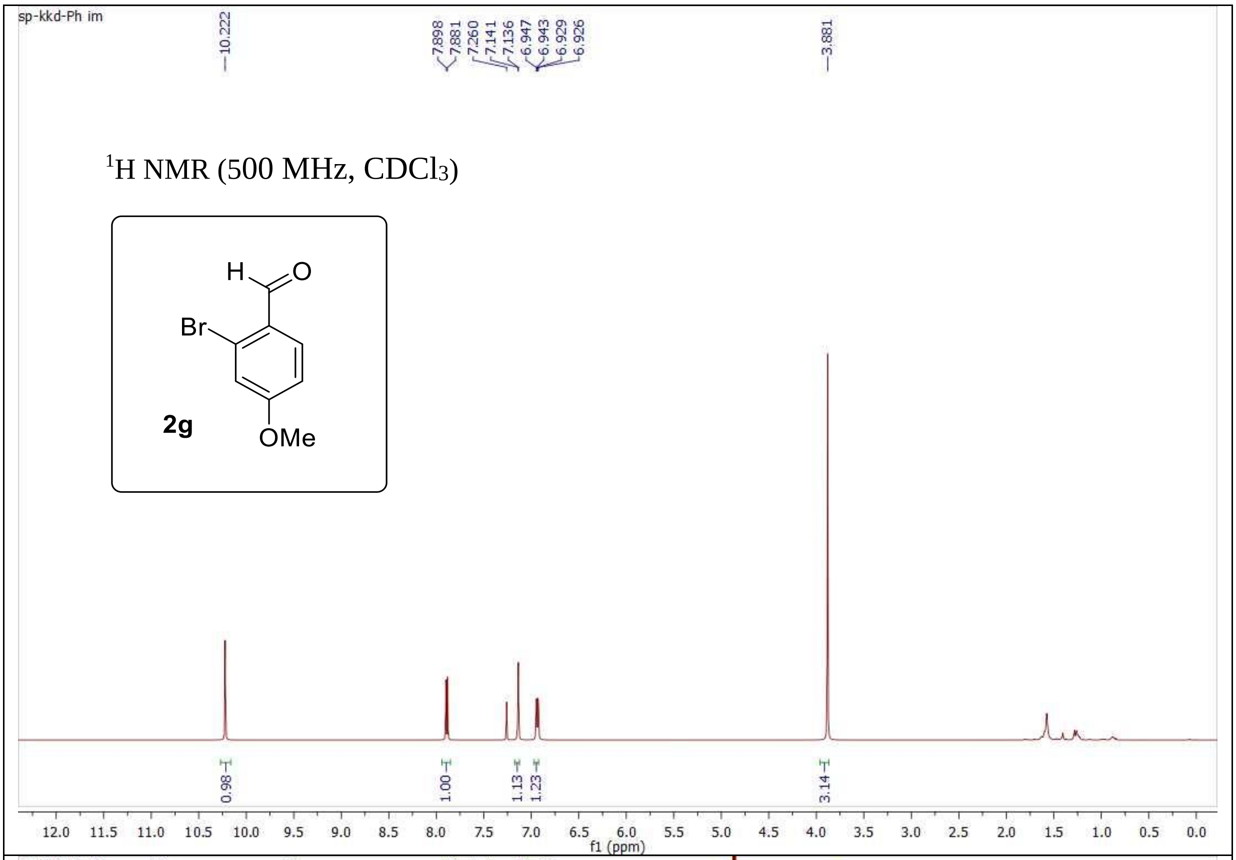
sp-kkd-Ph im
10.222
7.898
7.881
7.260
7.141
7.136
6.947
6.943
6.929
6.926
3.881
^{1}H NMR (500 MHz, CDCl$_3$)
H
O
Br
2g
OMe
0.98
1.00
1.13
1.23
3.14
f1 (ppm)

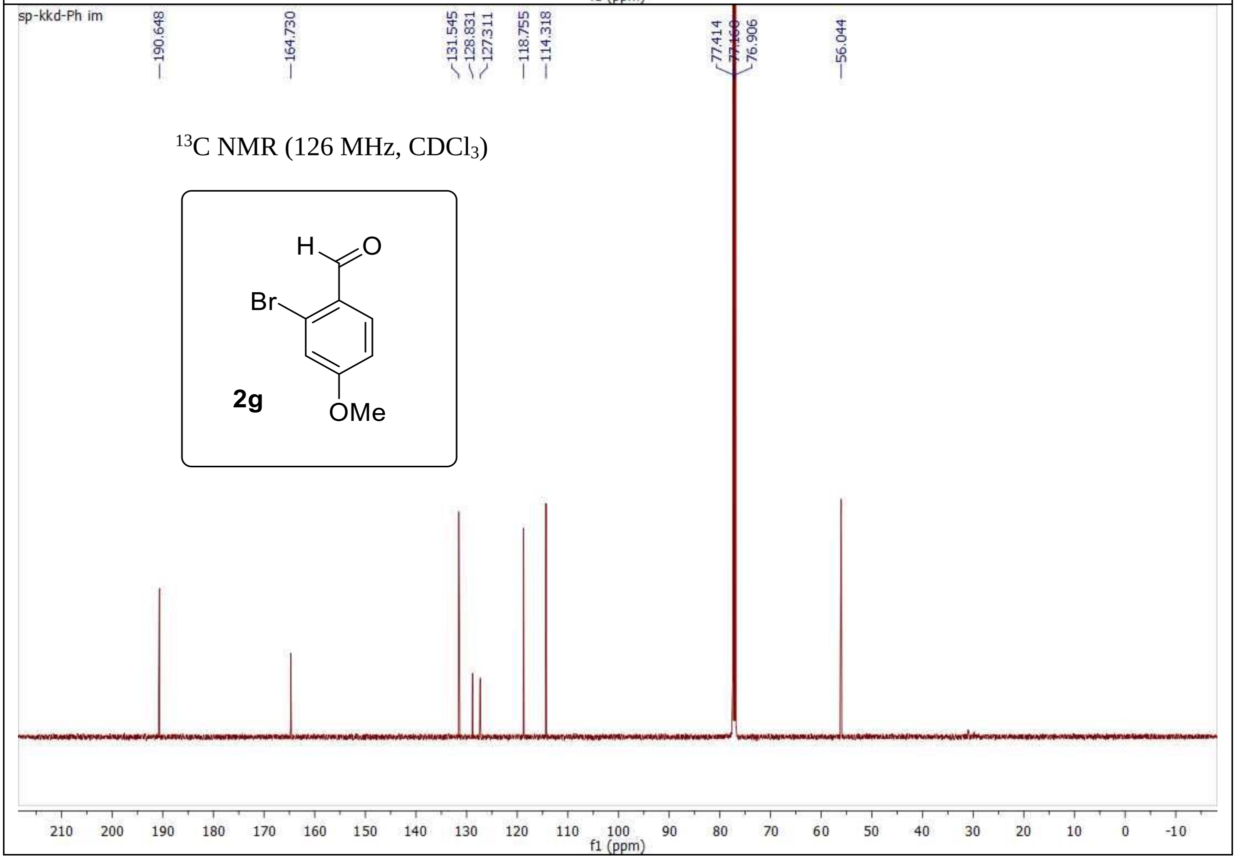
sp-kkd-Ph im
190.648
164.730
131.545
128.831
127.311
118.755
114.318
77.414
77.160
76.906
56.044
^{13}C NMR (126 MHz, CDCl$_3$)
H
O
Br
2g
OMe
f1 (ppm)

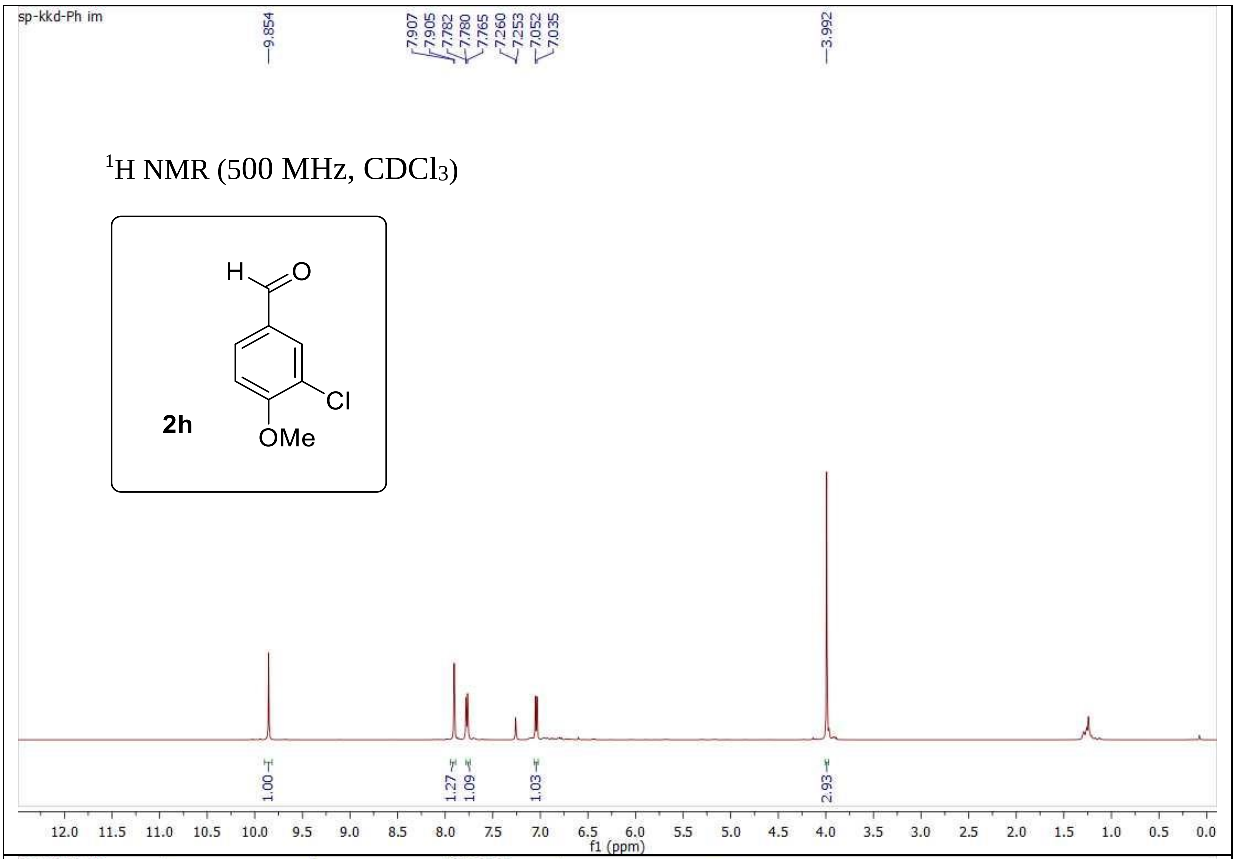
sp-kkd-Ph im
9.854
7.907
7.905
7.782
7.780
7.765
7.260
7.253
7.052
7.035
3.992
^{1}H NMR (500 MHz, CDCl$_3$)
H
O
Cl
2h
OMe
1.00
1.27
1.09
1.03
2.93
f1 (ppm)

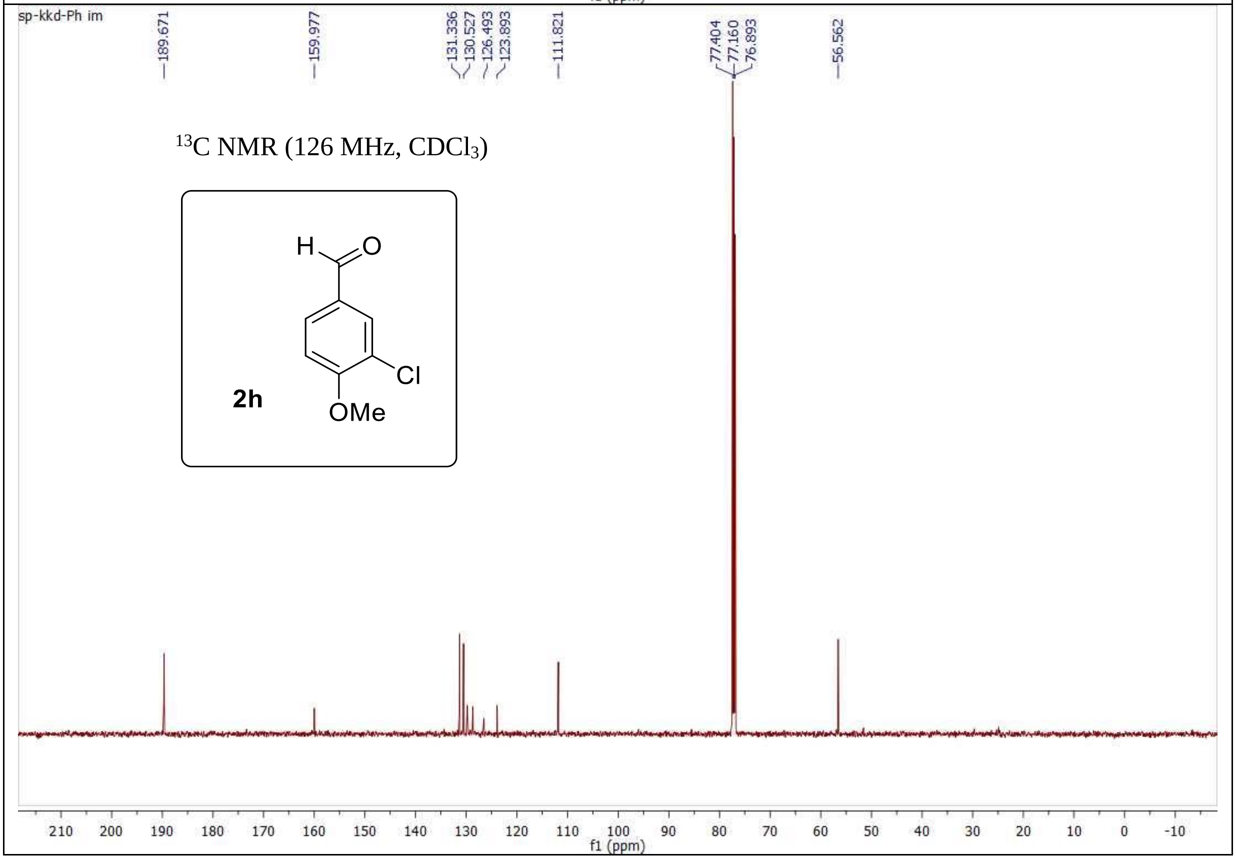
sp-kkd-Ph im
189.671
159.977
131.336
130.527
126.493
123.893
111.821
77.404
77.160
76.893
56.562
^{13}C NMR (126 MHz, CDCl$_3$)
H
O
Cl
2h
OMe
f1 (ppm)

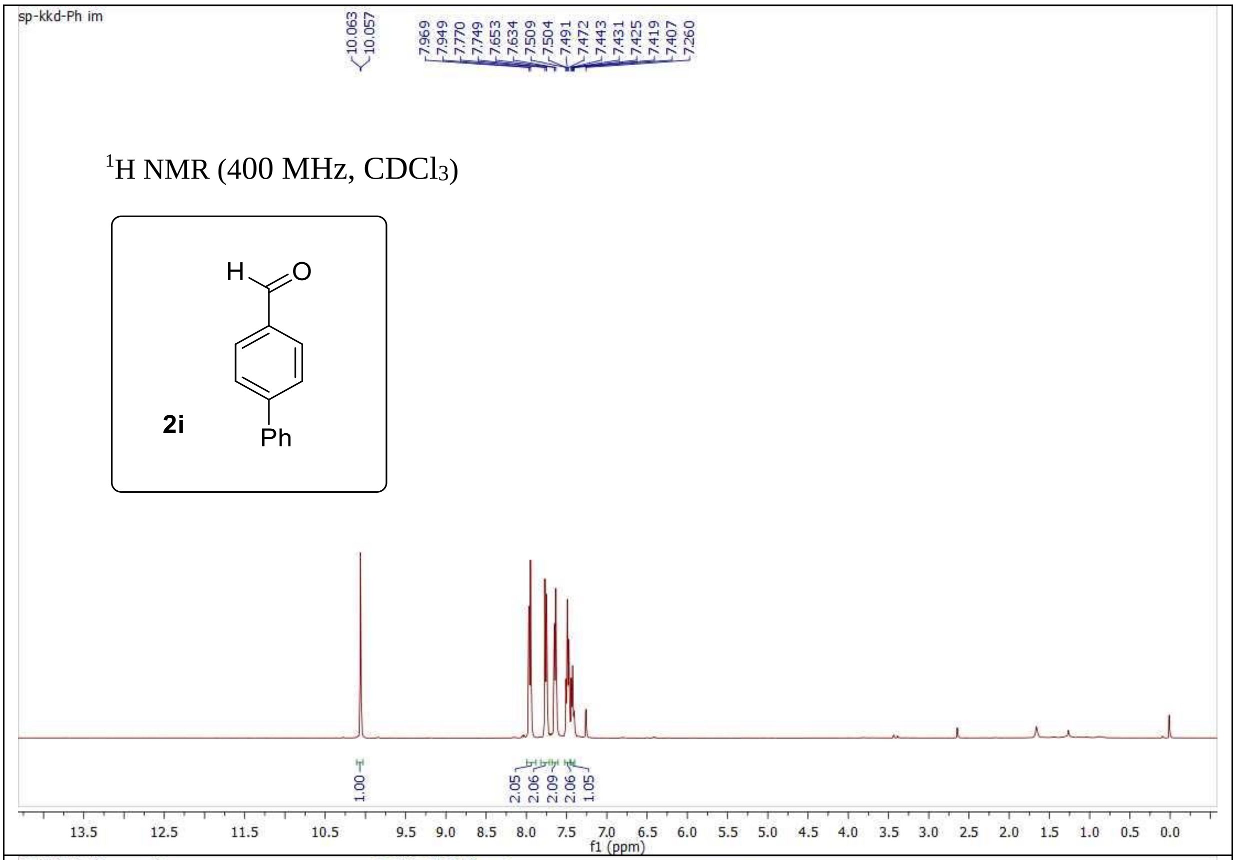
sp-kkd-Ph im
10.063
10.057
7.969
7.949
7.770
7.749
7.653
7.634
7.509
7.504
7.491
7.472
7.443
7.431
7.425
7.419
7.407
7.260
1H NMR (400 MHz, CDCl3)
H
O
2i
Ph
1.00
2.05
2.06
2.09
2.06
1.05
13.5
12.5
11.5
10.5
9.5
9.0
8.5
8.0
7.5
7.0
6.5
6.0
5.5
5.0
4.5
4.0
3.5
3.0
2.5
2.0
1.5
1.0
0.5
0.0
f1 (ppm)

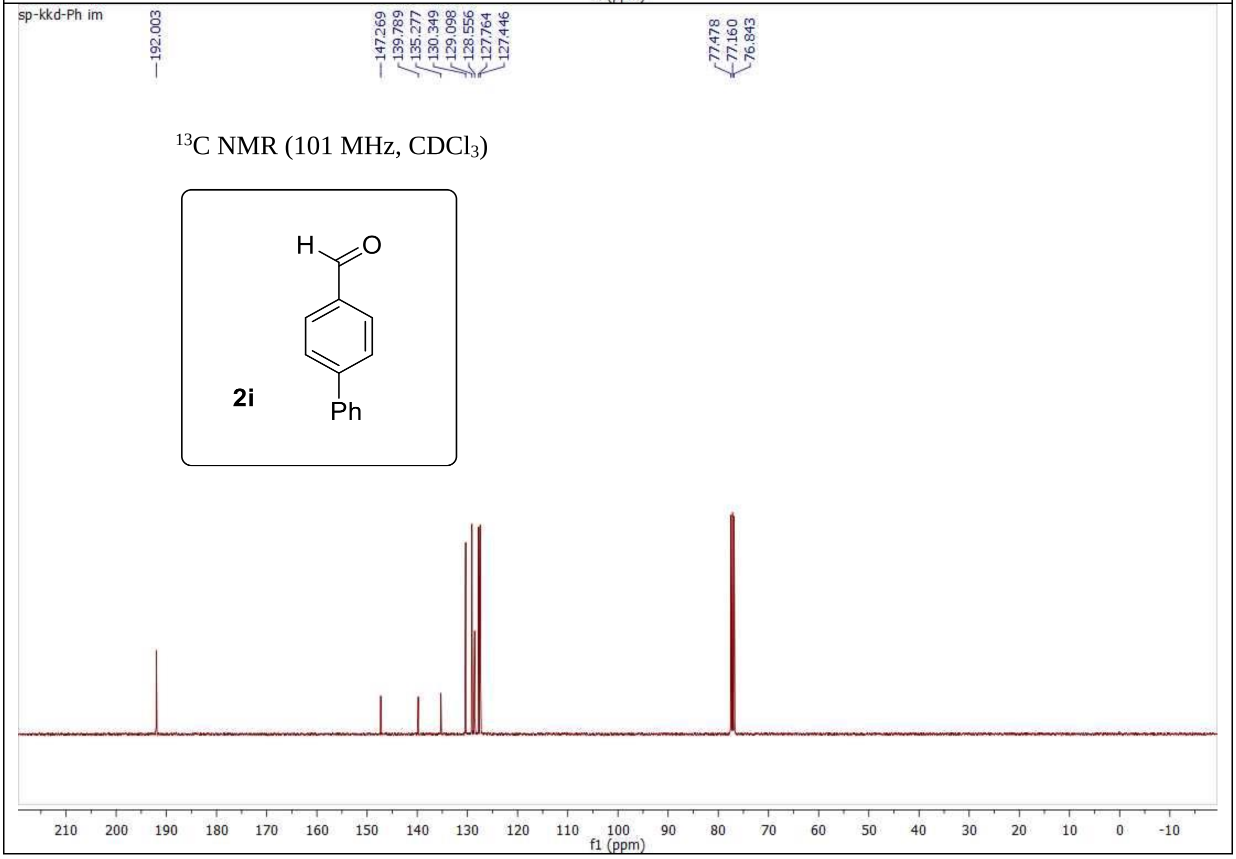
sp-kkd-Ph im
192.003
147.269
139.789
135.277
130.349
129.098
128.556
127.764
127.446
77.478
77.160
76.843
13C NMR (101 MHz, CDCl3)
H
O
2i
Ph
210
200
190
180
170
160
150
140
130
120
110
100
90
80
70
60
50
40
30
20
10
0
-10
f1 (ppm)

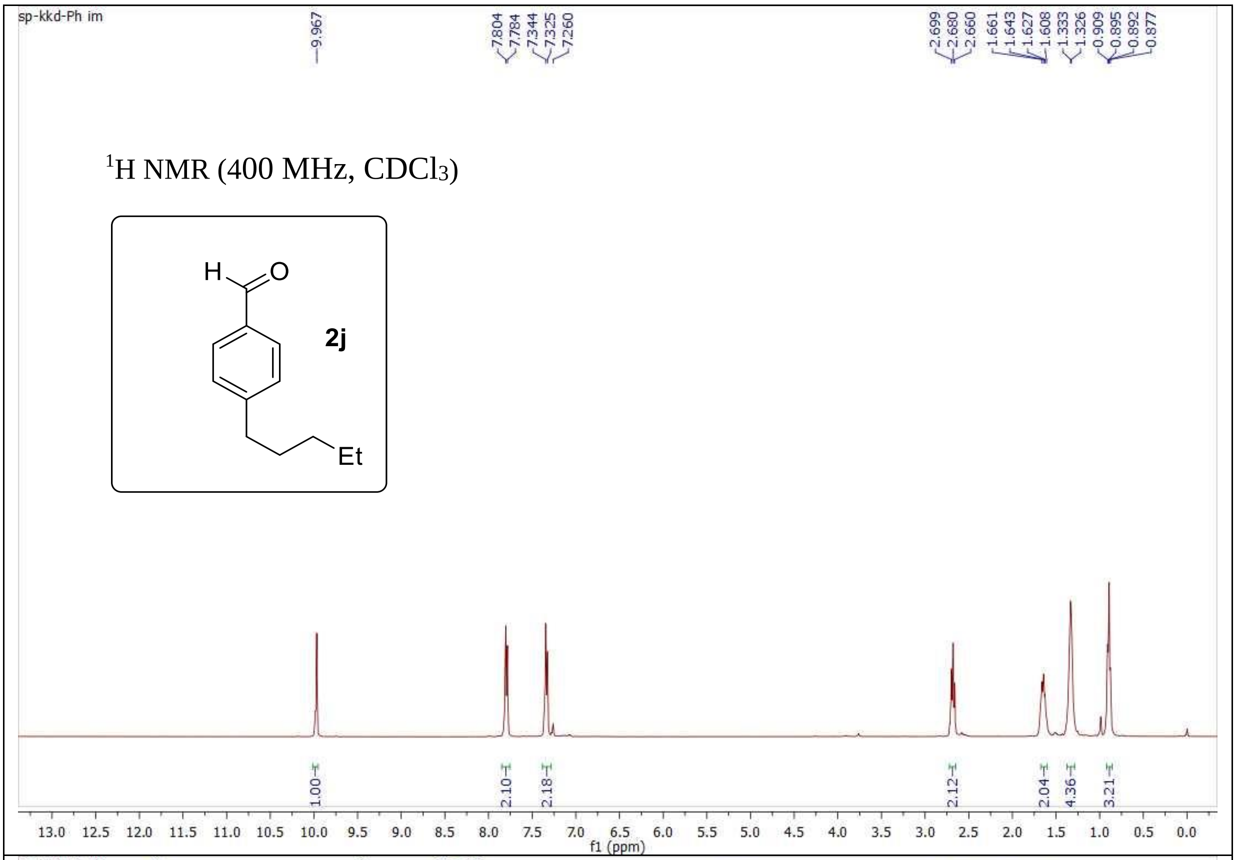
sp-kkd-Ph im
1H NMR (400 MHz, CDCl3)
2j
Et
f1 (ppm)

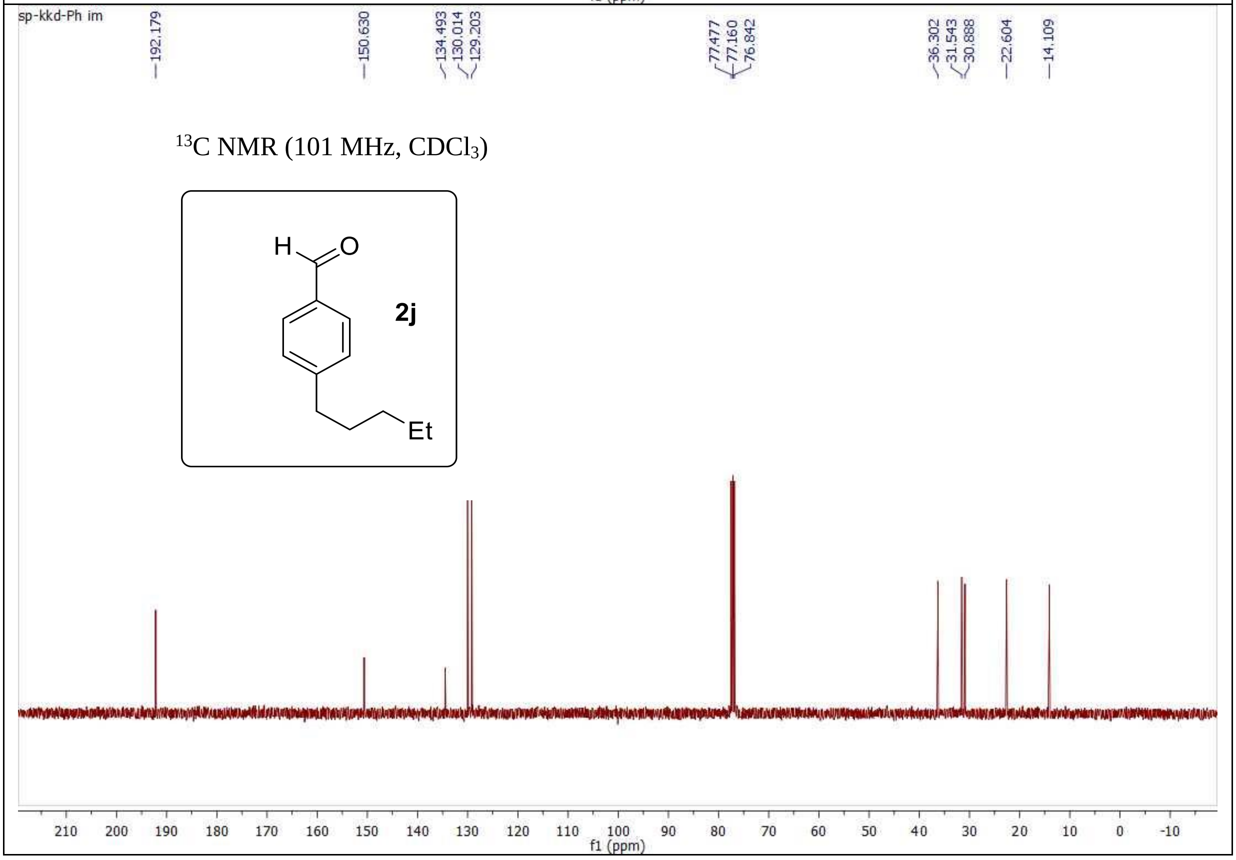
sp-kkd-Ph im
13C NMR (101 MHz, CDCl3)
2j
Et
f1 (ppm)

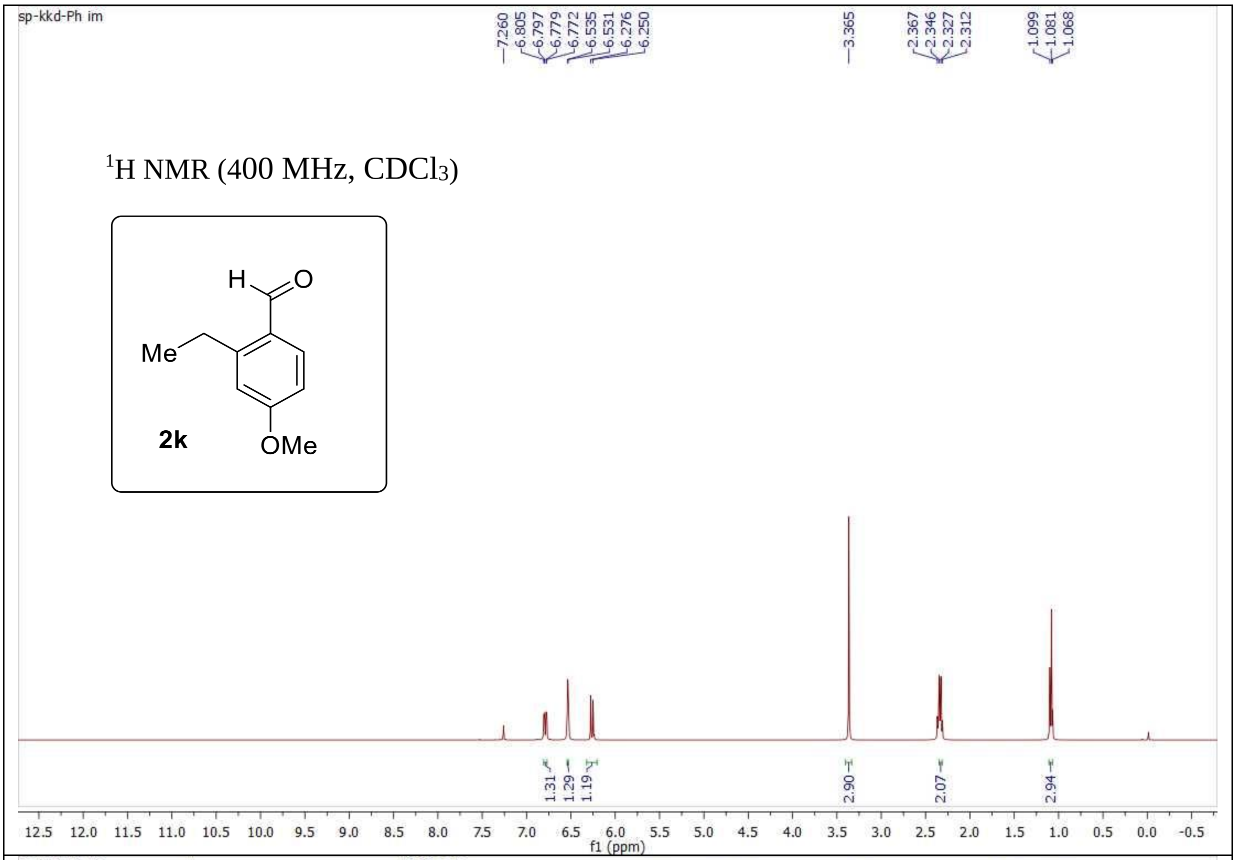
sp-kkd-Ph im
7.260
6.805
6.797
6.779
6.772
6.535
6.531
6.276
6.250
3.365
2.367
2.346
2.327
2.312
1.099
1.081
1.068
¹H NMR (400 MHz, CDCl3)
H
O
Me
2k
OMe
1.31
1.29
1.19
2.90
2.07
2.94
f1 (ppm)

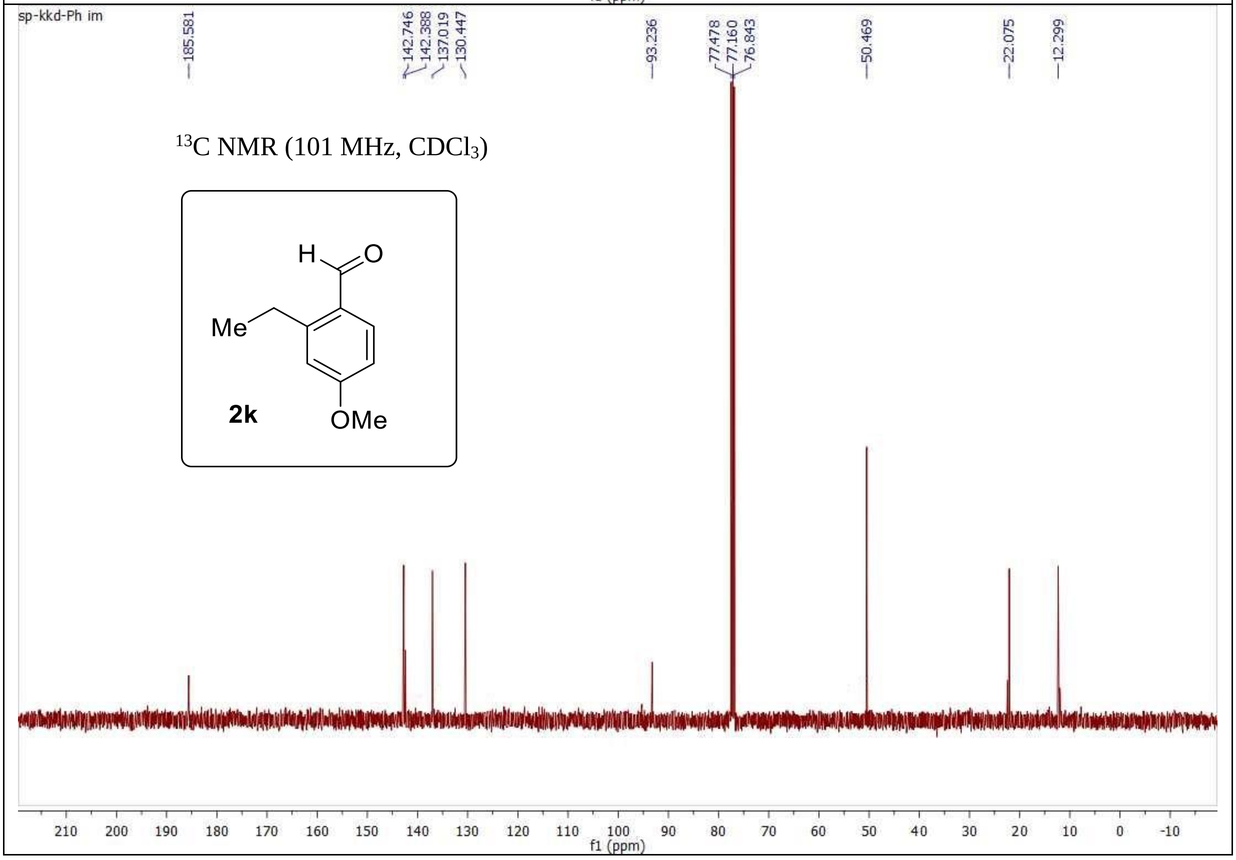
sp-kkd-Ph im
185.581
142.746
142.388
137.019
130.447
93.236
77.478
77.160
76.843
50.469
22.075
12.299
¹³C NMR (101 MHz, CDCl3)
H
O
Me
2k
OMe
f1 (ppm)

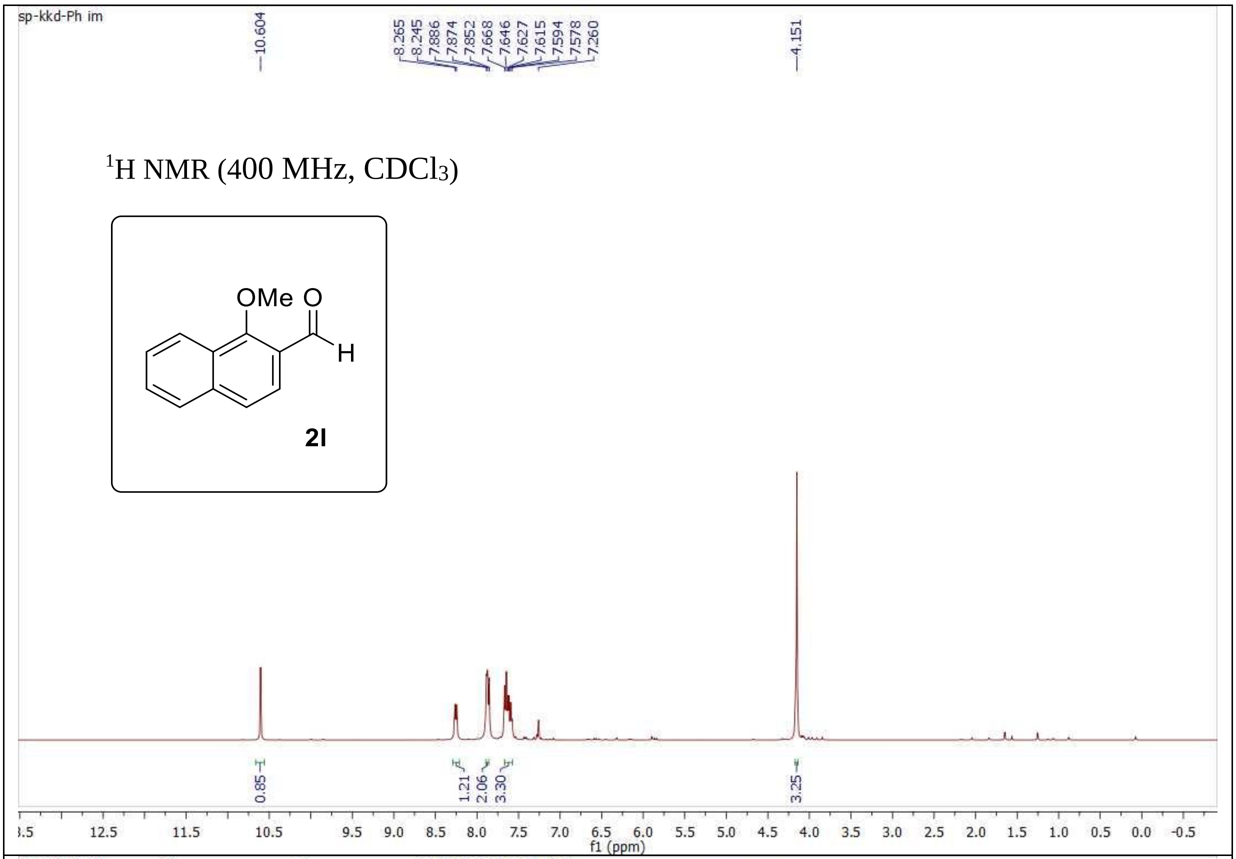
sp-kkd-Ph im
10.604
8.265
8.245
7.886
7.874
7.852
7.668
7.646
7.627
7.615
7.594
7.578
7.260
4.151
1H NMR (400 MHz, CDCl3)
OMe O
H
2l
0.85
1.21
2.06
3.30
3.25
f1 (ppm)

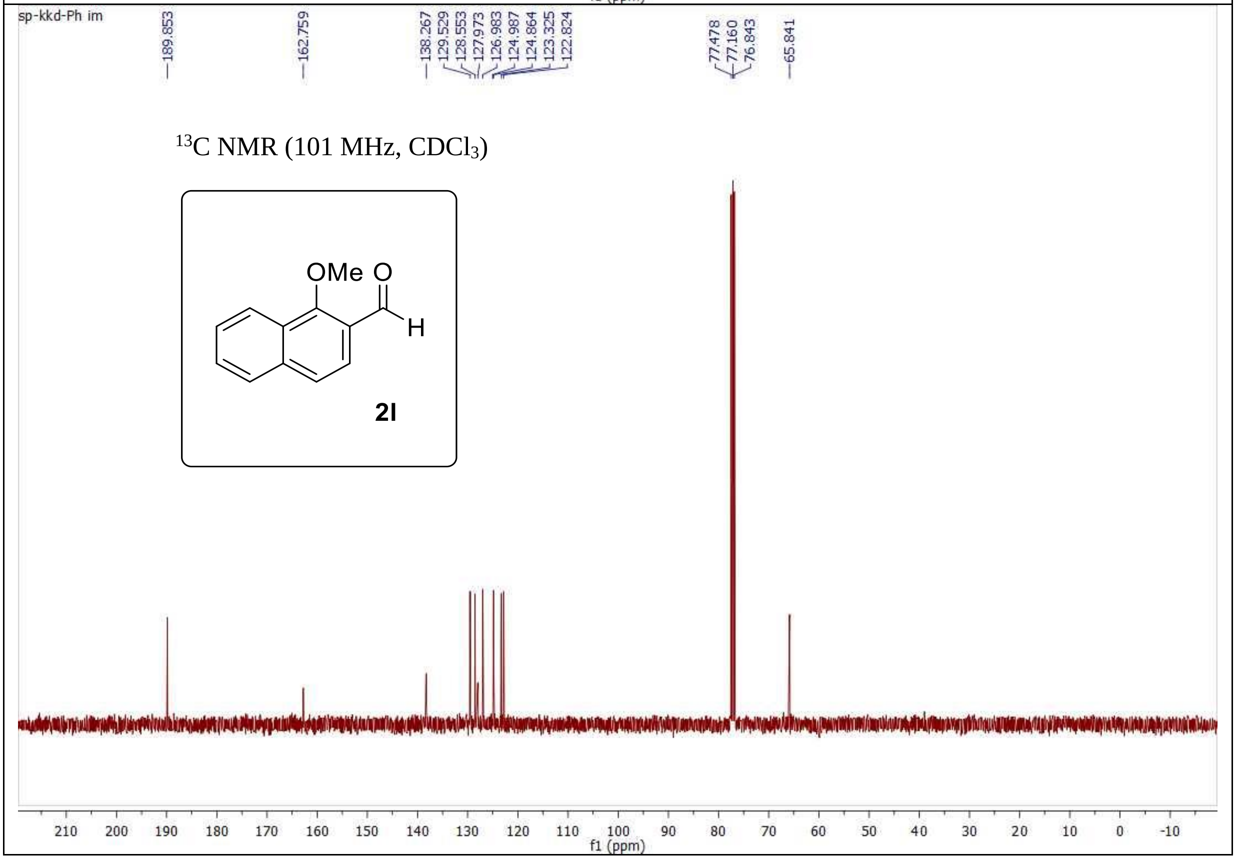
sp-kkd-Ph im
189.853
162.759
138.267
129.529
128.553
127.973
126.983
124.987
124.864
123.325
122.824
77.478
77.160
76.843
65.841
13C NMR (101 MHz, CDCl3)
OMe O
H
2l
f1 (ppm)

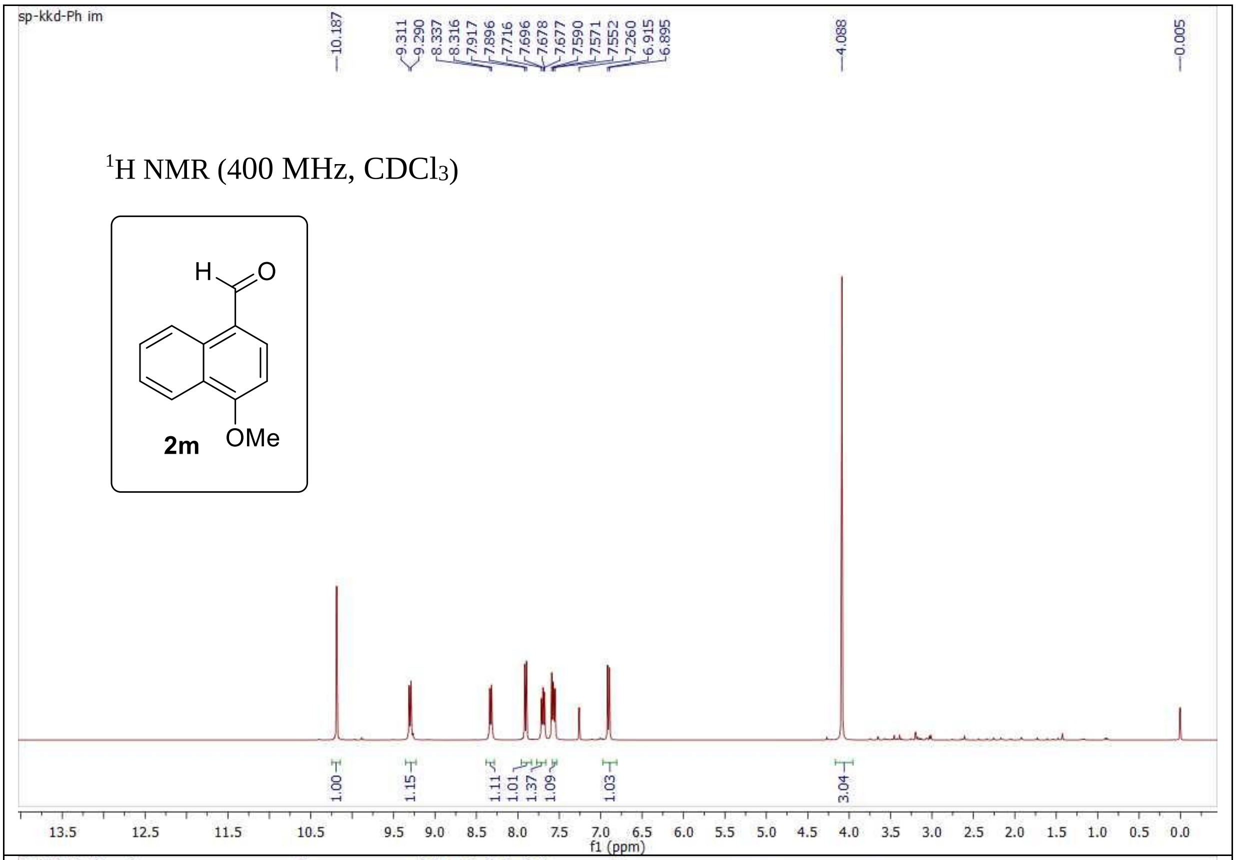
sp-kkd-Ph im
1H NMR (400 MHz, CDCl3)
2m
OMe
f1 (ppm)

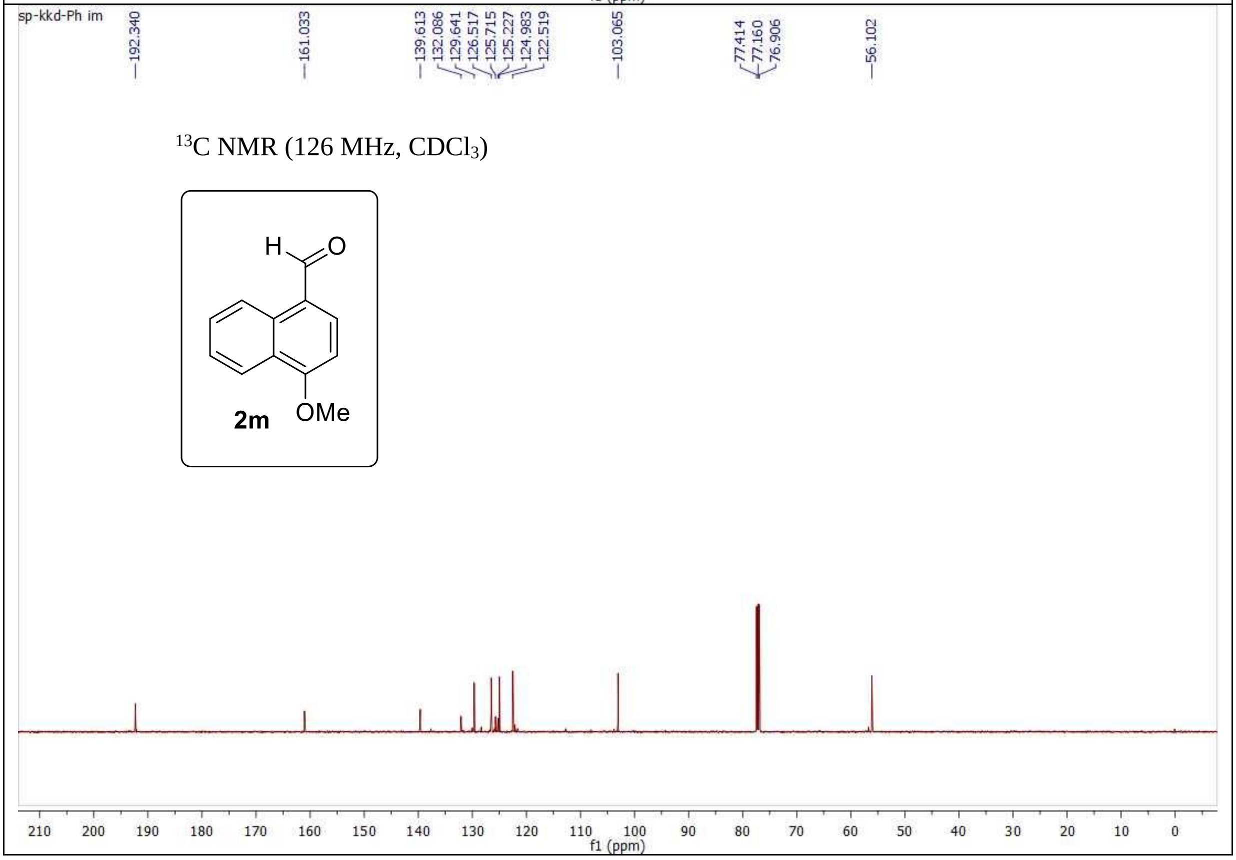
sp-kkd-Ph im
13C NMR (126 MHz, CDCl3)
2m
OMe
f1 (ppm)

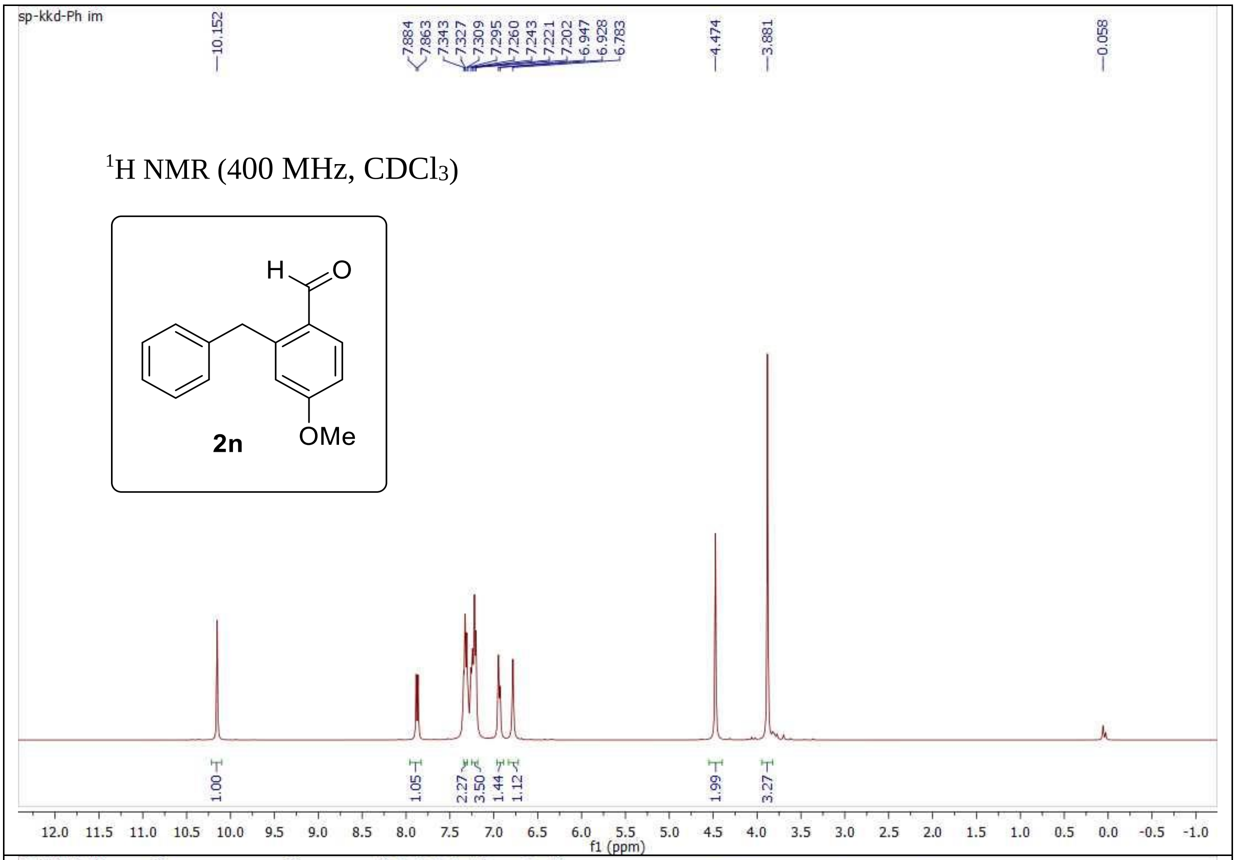
sp-kkd-Ph im
10.152
7.884
7.863
7.343
7.327
7.309
7.295
7.260
7.243
7.221
7.202
6.947
6.928
6.783
4.474
3.881
0.058
1H NMR (400 MHz, CDCl3)
H
O
OMe
2n
1.00
1.05
2.27
3.50
1.44
1.12
1.99
3.27
f1 (ppm)

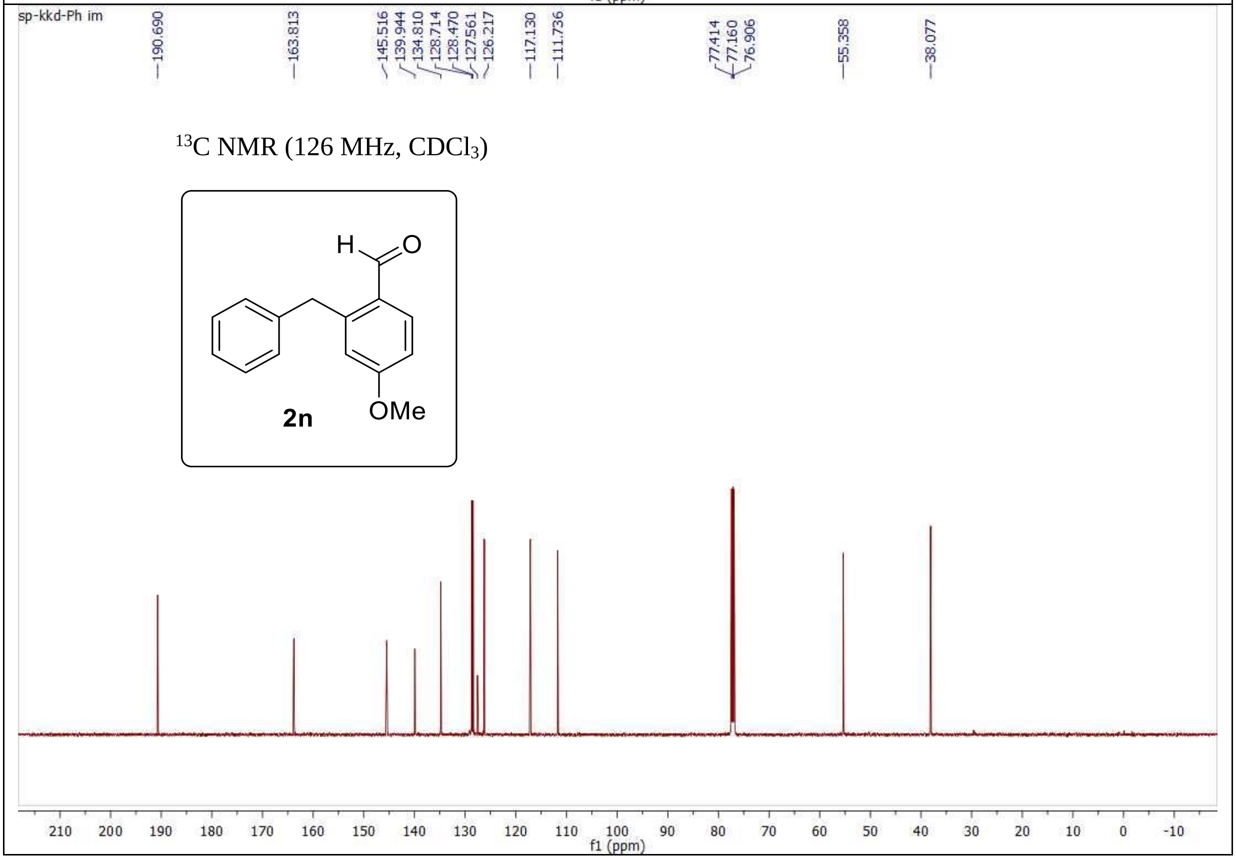
sp-kkd-Ph im
190.690
163.813
145.516
139.944
134.810
128.714
128.470
127.561
126.217
117.130
111.736
77.414
77.160
76.906
55.358
38.077
13C NMR (126 MHz, CDCl3)
H
O
OMe
2n
f1 (ppm)

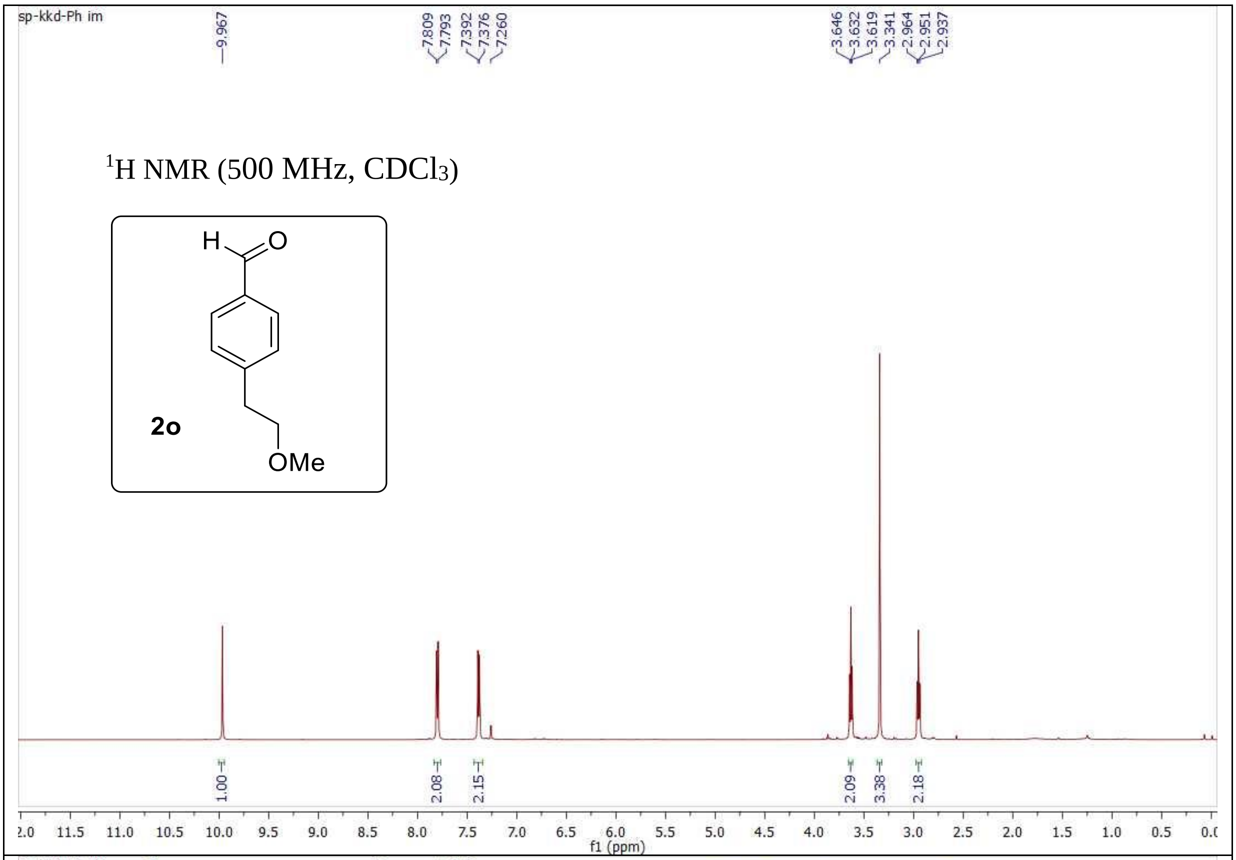
sp-kkd-Ph im
9.967
7.809
7.793
7.392
7.376
7.260
3.646
3.632
3.619
3.341
2.964
2.951
2.937
1H NMR (500 MHz, CDCl3)
H
O
2o
OMe
1.00
2.08
2.15
2.09
3.38
2.18
f1 (ppm)

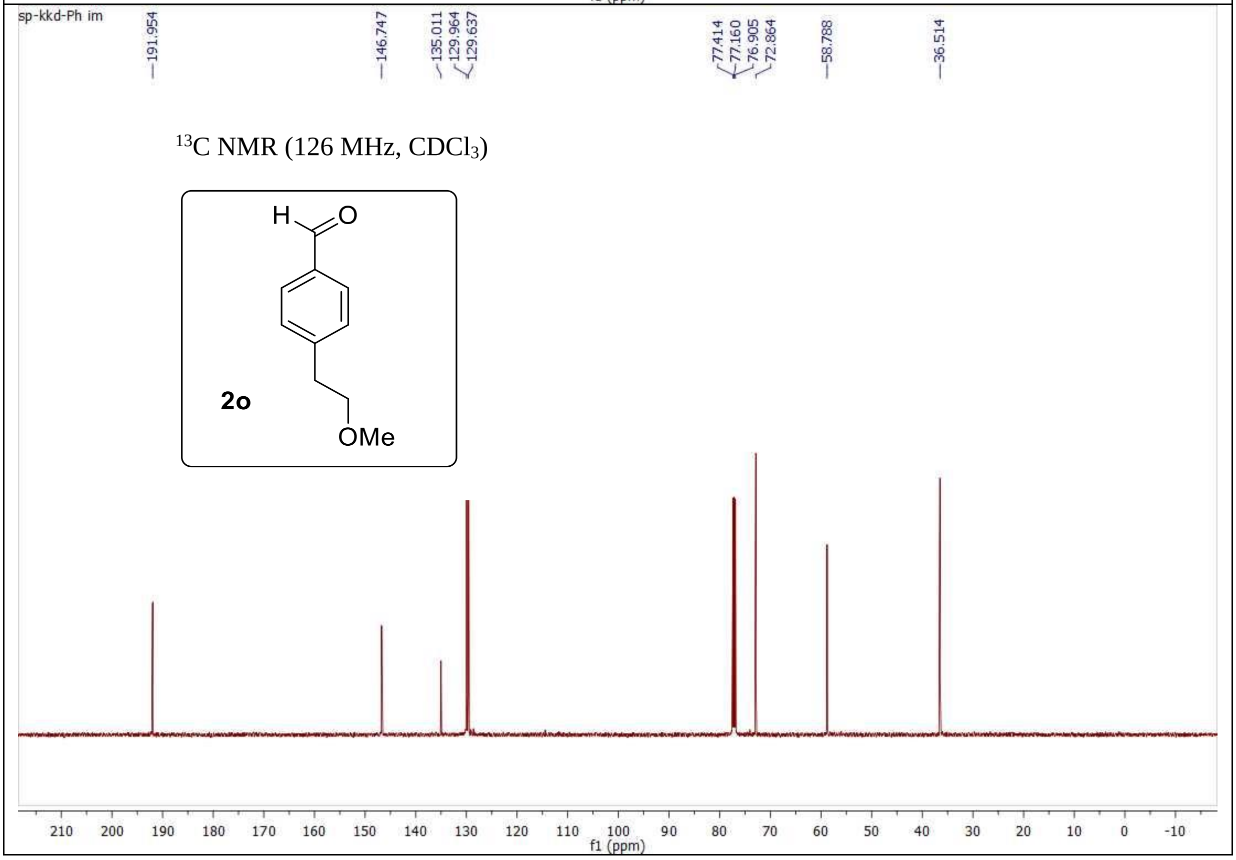
sp-kkd-Ph im
191.954
146.747
135.011
129.964
129.637
77.414
77.160
76.905
72.864
58.788
36.514
13C NMR (126 MHz, CDCl3)
H
O
2o
OMe
f1 (ppm)

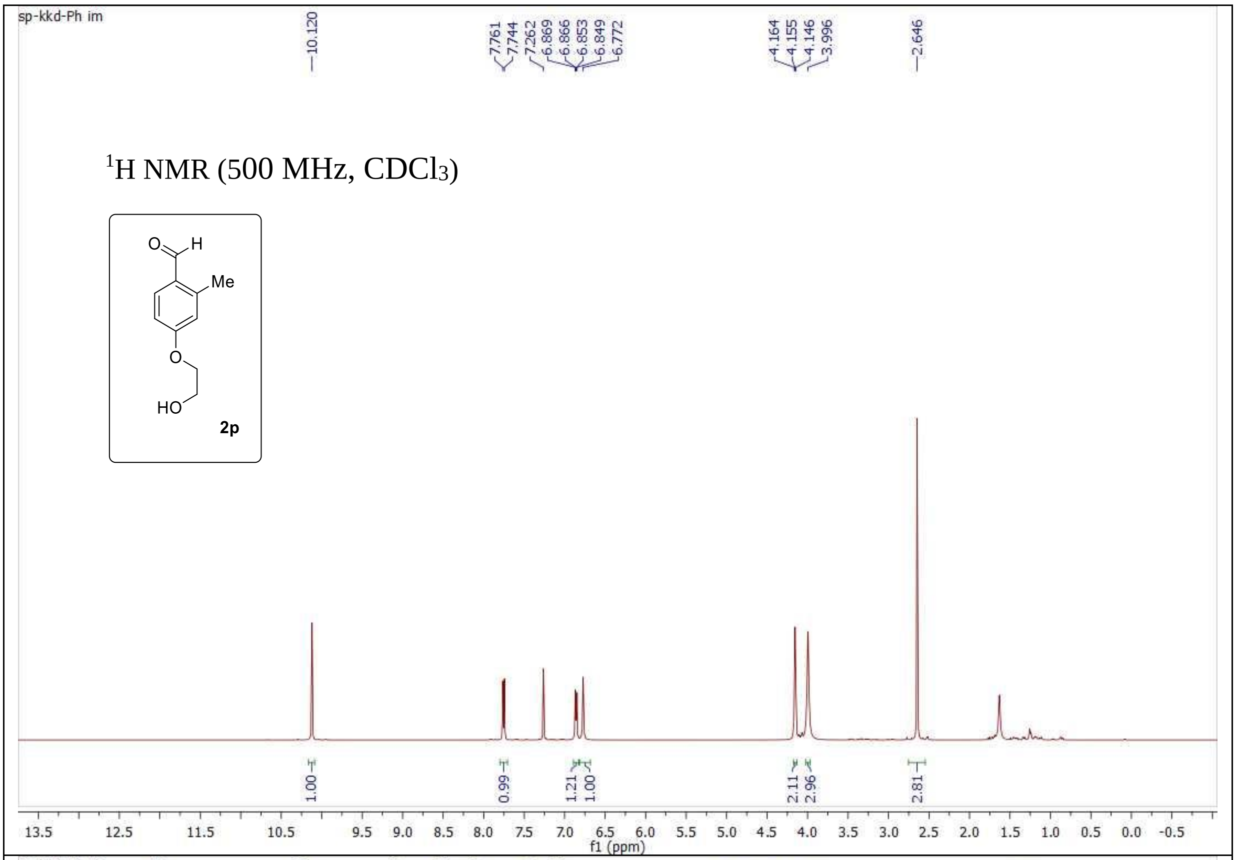
sp-kkd-Ph im
10.120
7.761
7.744
7.262
6.869
6.866
6.853
6.849
6.772
4.164
4.155
4.146
3.996
2.646
^{1}H NMR (500 MHz, $CDCl_3$)
O
H
Me
O
HO
2p
1.00
0.99
1.21
1.00
2.11
2.96
2.81
f1 (ppm)

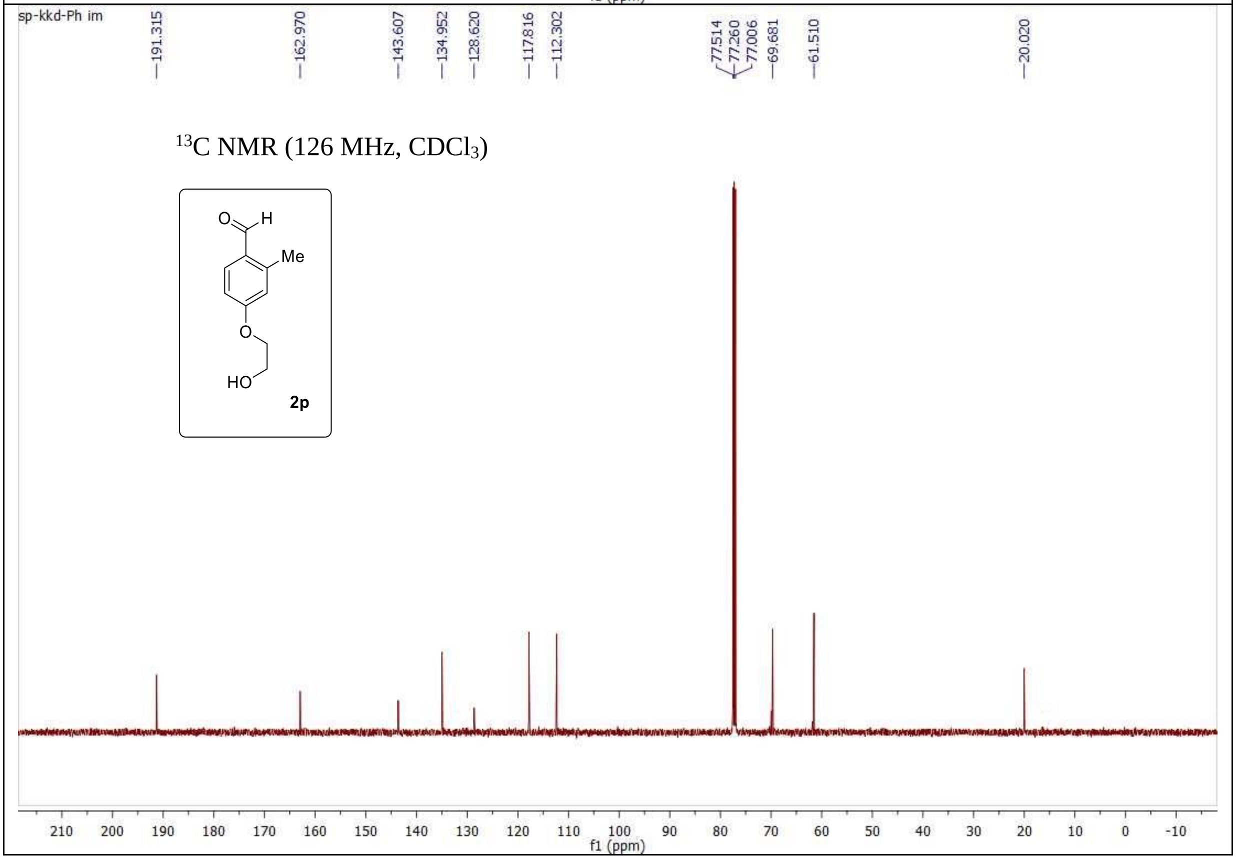
sp-kkd-Ph im
191.315
162.970
143.607
134.952
128.620
117.816
112.302
77.514
77.260
77.006
69.681
61.510
20.020
^{13}C NMR (126 MHz, $CDCl_3$)
O
H
Me
O
HO
2p
f1 (ppm)

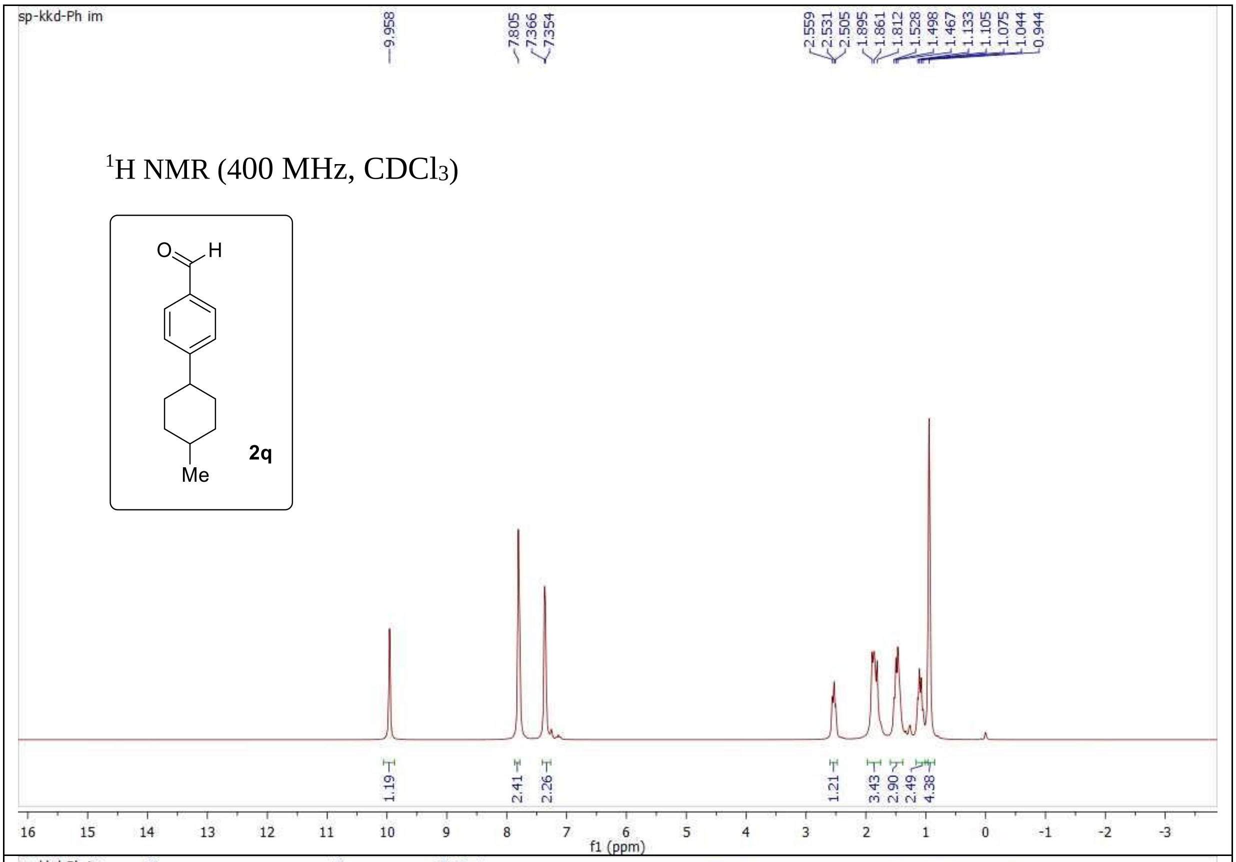
sp-kkd-Ph im
9.958
7.805
7.366
7.354
2.559
2.531
2.505
1.895
1.861
1.812
1.528
1.498
1.467
1.133
1.105
1.075
1.044
0.944
1H NMR (400 MHz, CDCl3)
O
H
2q
Me
1.19
2.41
2.26
1.21
3.43
2.90
2.49
4.38
f1 (ppm)

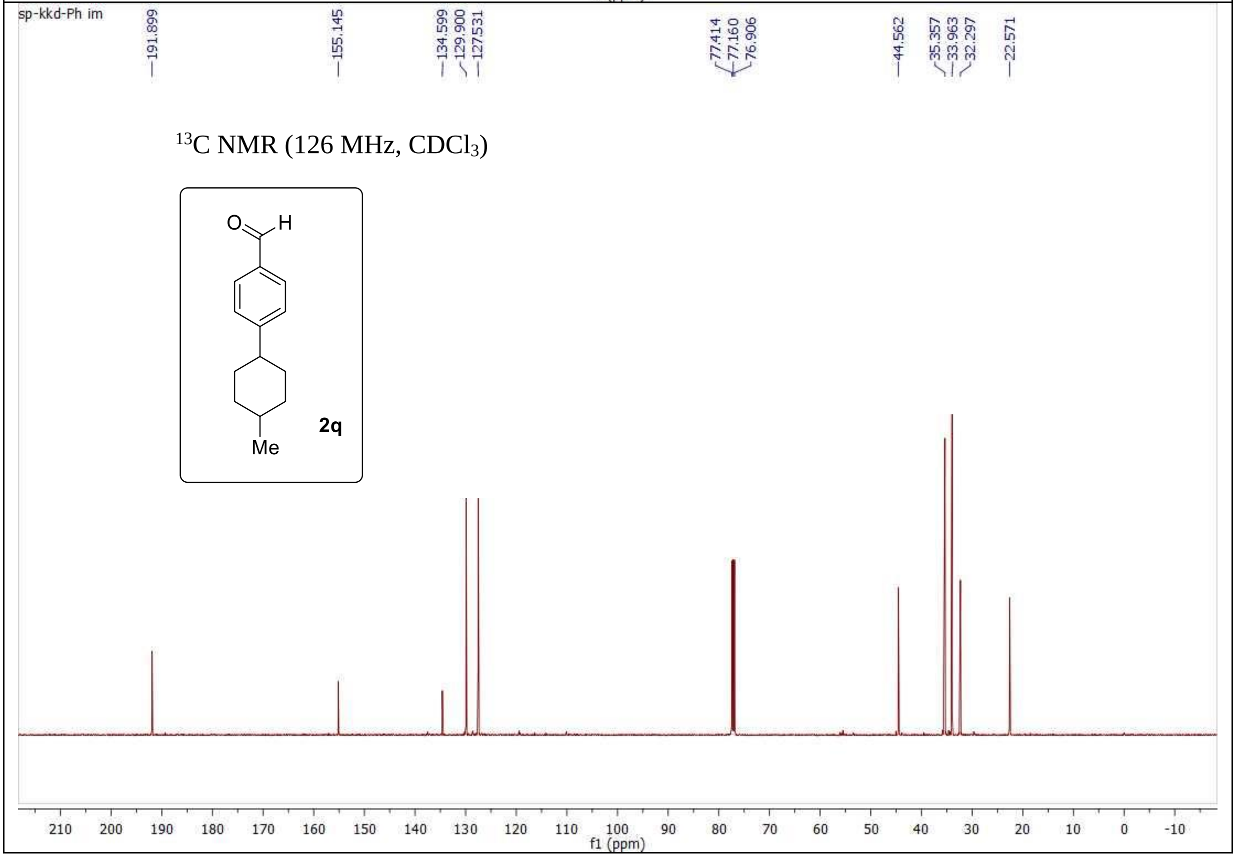
sp-kkd-Ph im
191.899
155.145
134.599
129.900
127.531
77.414
77.160
76.906
44.562
35.357
33.963
32.297
22.571
13C NMR (126 MHz, CDCl3)
O
H
2q
Me
f1 (ppm)

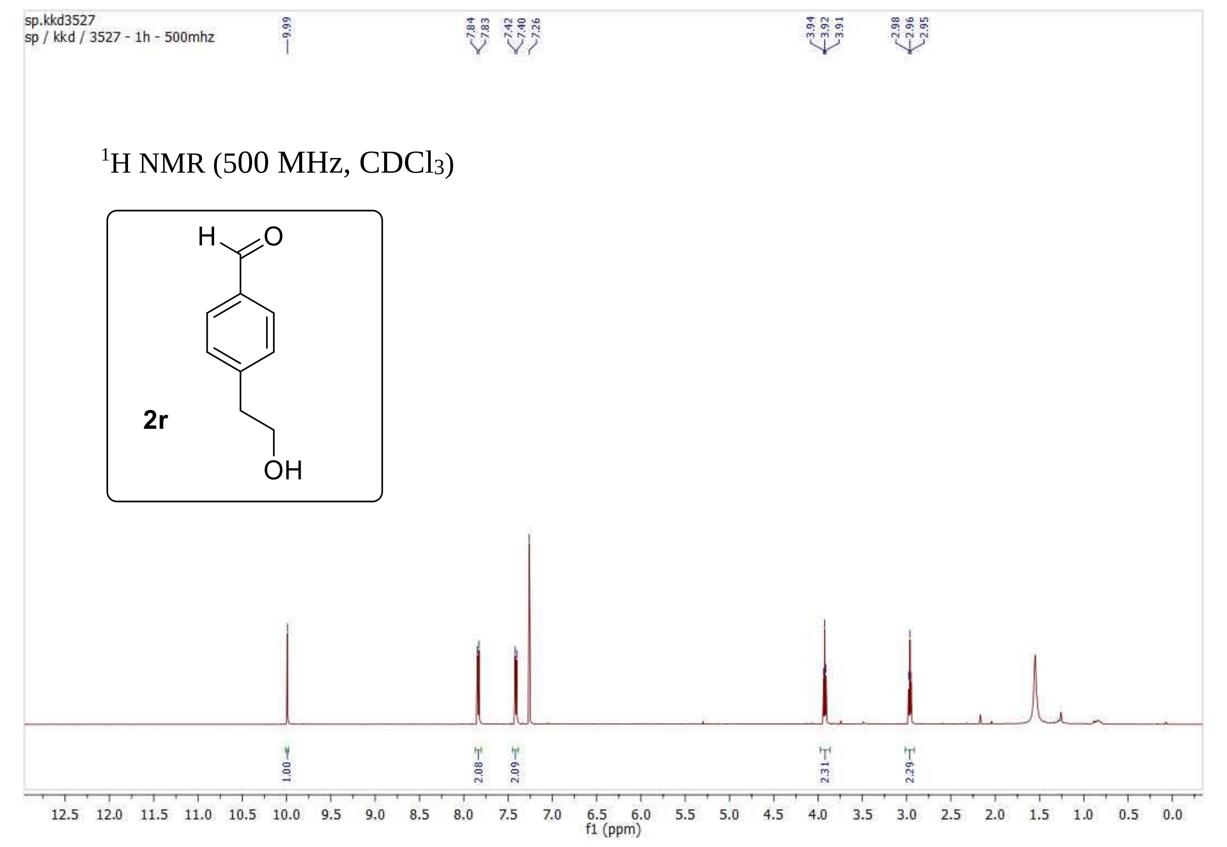
sp.kkd3527
sp / kkd / 3527 - 1h - 500mhz
9.99
7.84
7.83
7.42
7.40
7.26
3.94
3.92
3.91
2.98
2.96
2.95
1H NMR (500 MHz, CDCl3)
H
O
2r
OH
1.00
2.08
2.09
2.31
2.29
f1 (ppm)

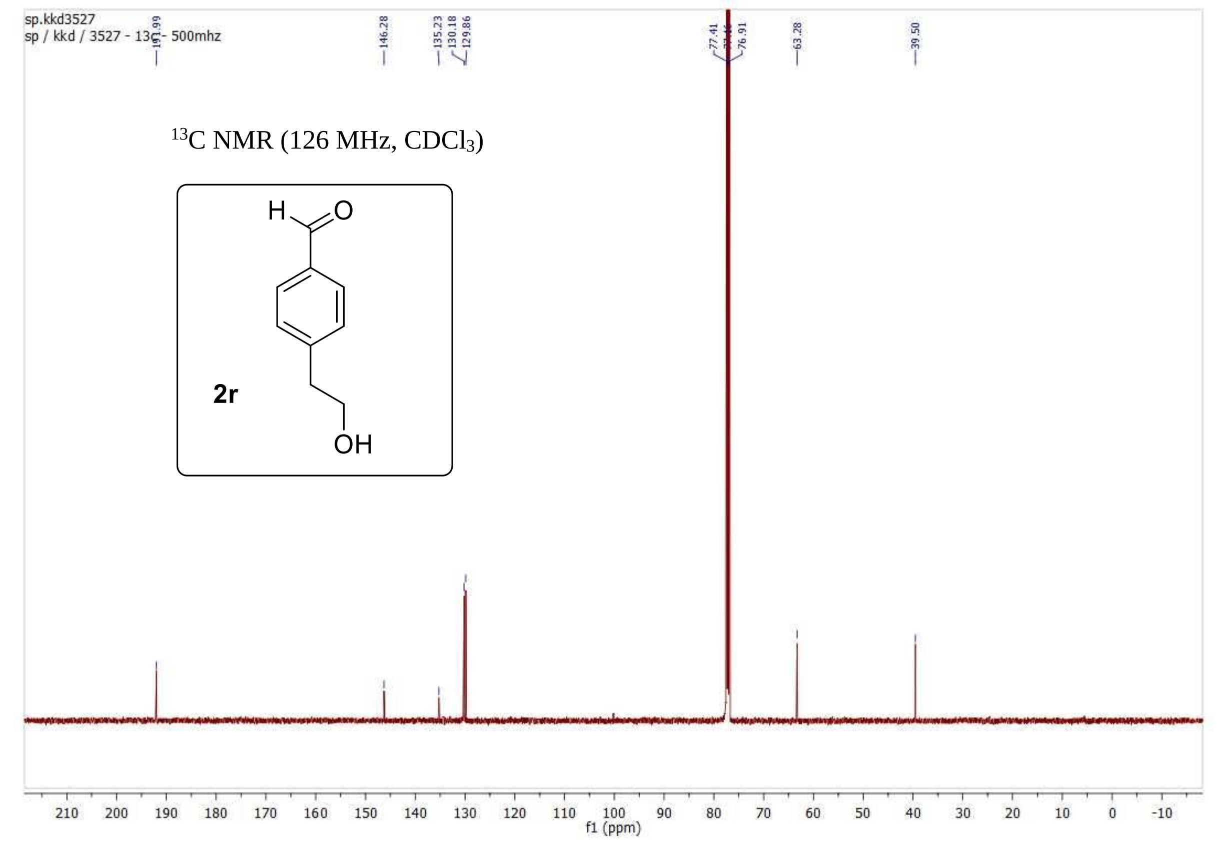
sp.kkd3527
sp / kkd / 3527 - 13c - 500mhz
191.99
146.28
135.23
130.18
129.86
77.41
76.91
63.28
39.50
13C NMR (126 MHz, CDCl3)
H
O
2r
OH
f1 (ppm)

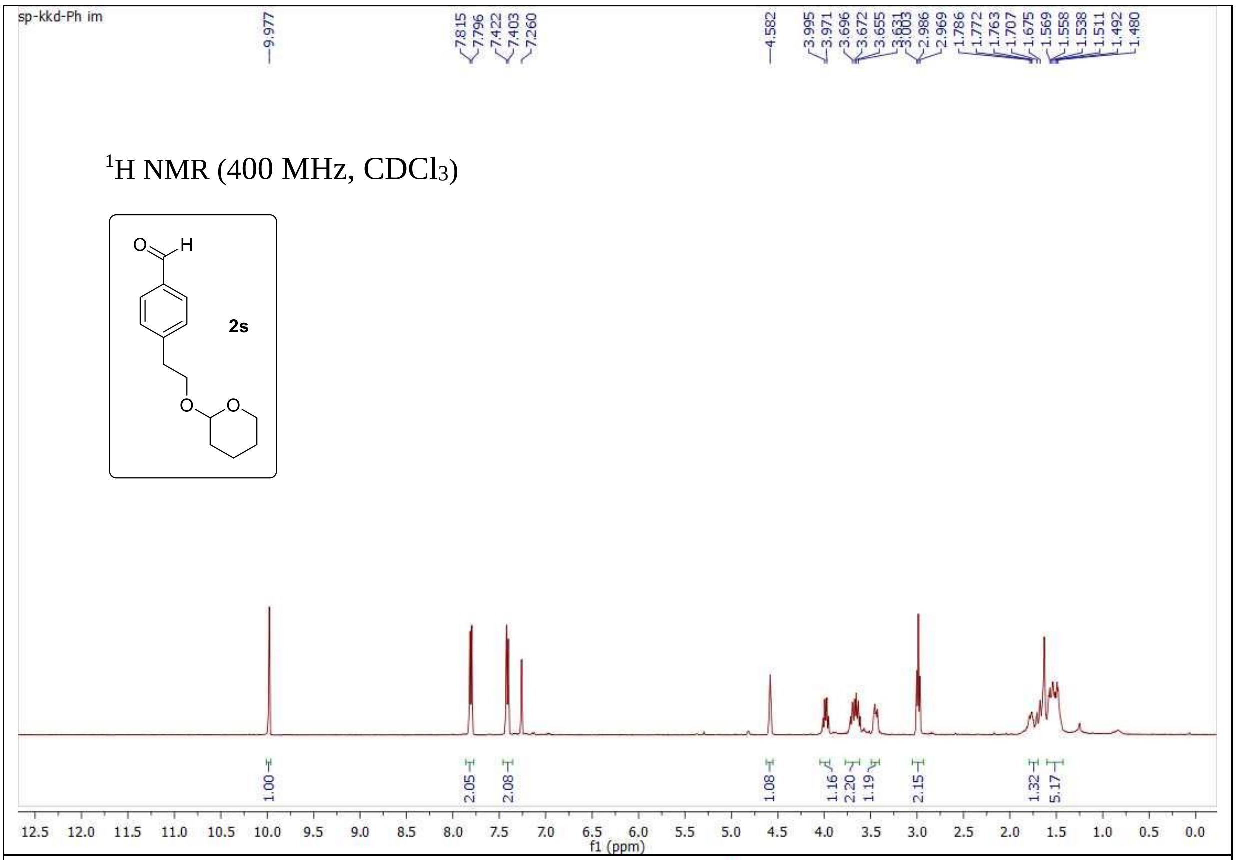
sp-kkd-Ph im
1H NMR (400 MHz, CDCl3)
2s
f1 (ppm)

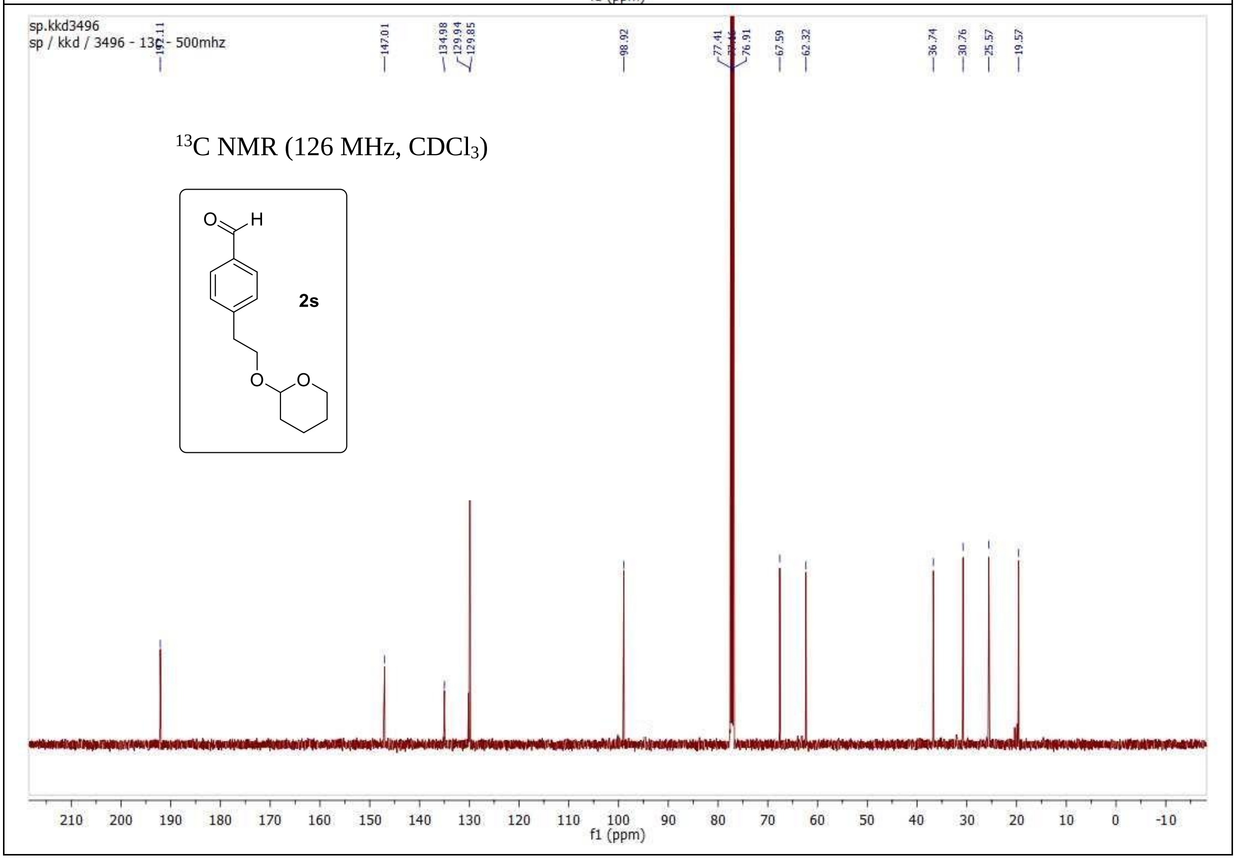
sp.kkd3496
13C NMR (126 MHz, CDCl3)
2s
f1 (ppm)

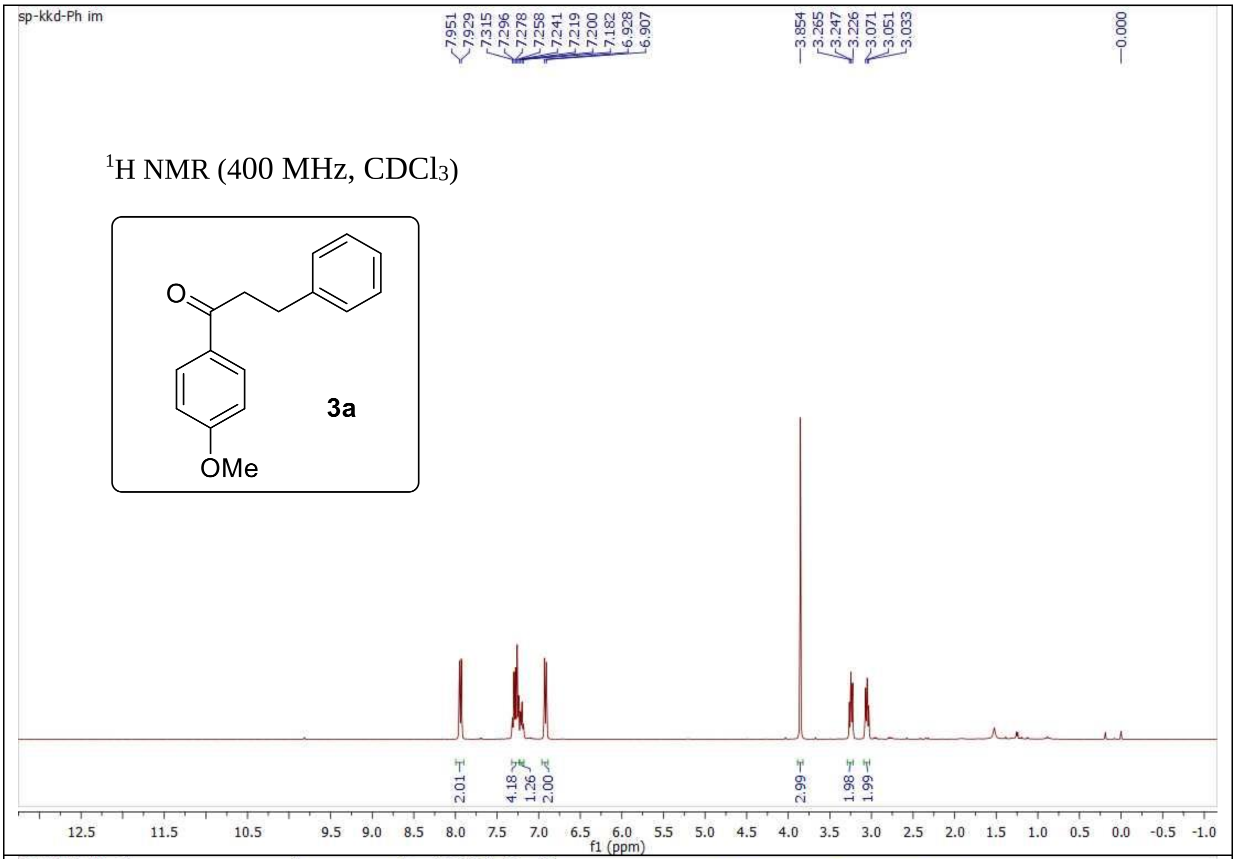
sp-kkd-Ph im
1H NMR (400 MHz, CDCl3)
O
OMe
3a
7.951
7.929
7.315
7.296
7.278
7.258
7.241
7.219
7.200
7.182
6.928
6.907
3.854
3.265
3.247
3.226
3.071
3.051
3.033
0.000
2.01
4.18
1.26
2.00
2.99
1.98
1.99
f1 (ppm)

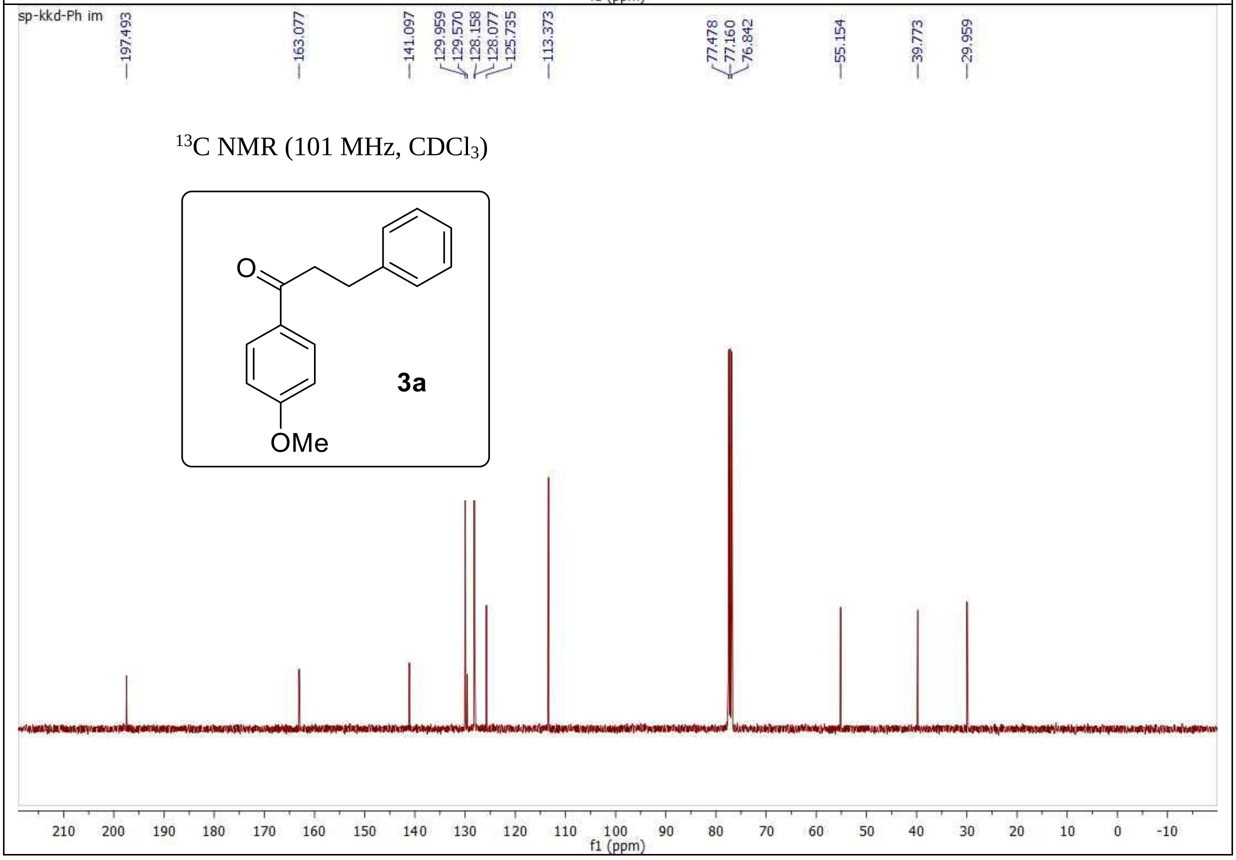
sp-kkd-Ph im
13C NMR (101 MHz, CDCl3)
O
OMe
3a
197.493
163.077
141.097
129.959
129.570
128.158
128.077
125.735
113.373
77.478
77.160
76.842
55.154
39.773
29.959
f1 (ppm)

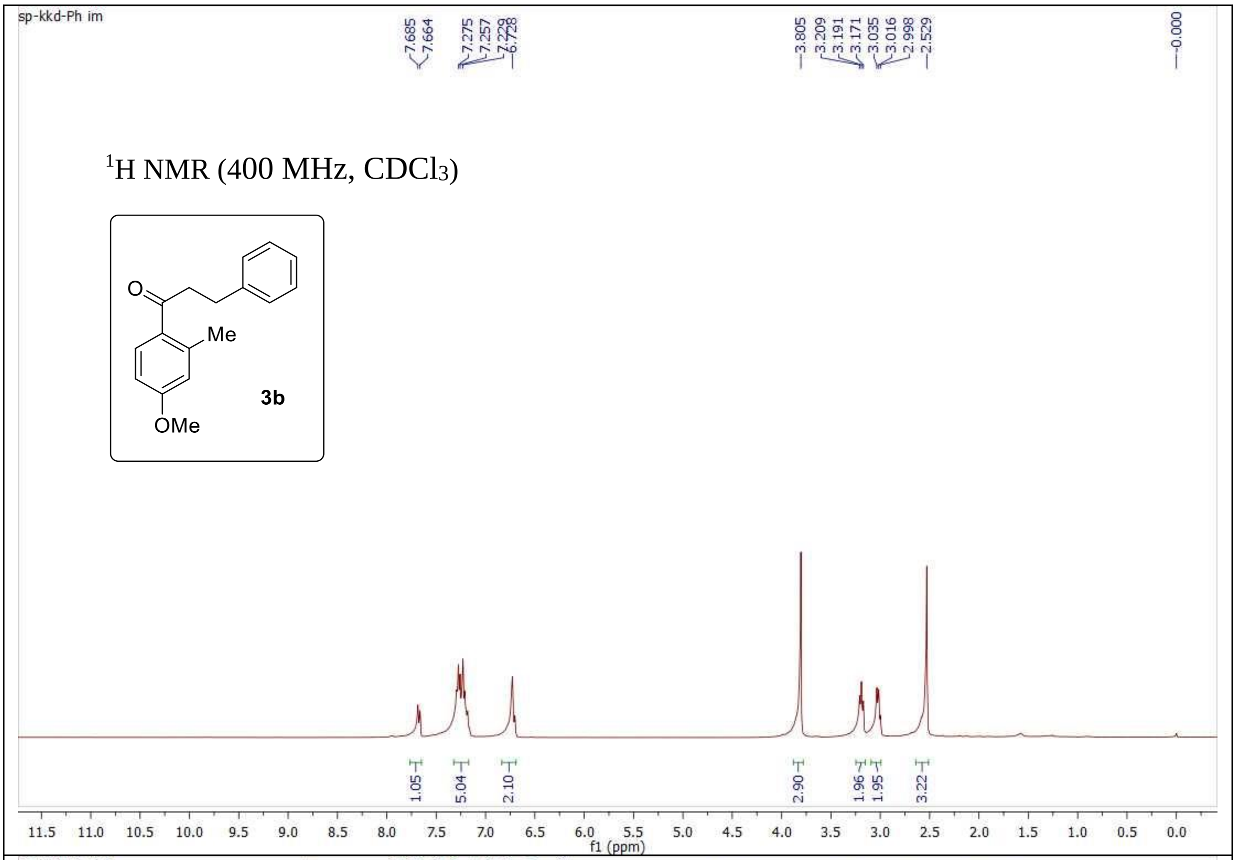
sp-kkd-Ph im
7.685
7.664
7.275
7.257
7.229
6.728
3.805
3.209
3.191
3.171
3.035
3.016
2.998
2.529
0.000
^{1}H NMR (400 MHz, $CDCl_3$)
O
Me
OMe
3b
1.05
5.04
2.10
2.90
1.96
1.95
3.22
11.5 11.0 10.5 10.0 9.5 9.0 8.5 8.0 7.5 7.0 6.5 6.0 5.5 5.0 4.5 4.0 3.5 3.0 2.5 2.0 1.5 1.0 0.5 0.0
f1 (ppm)

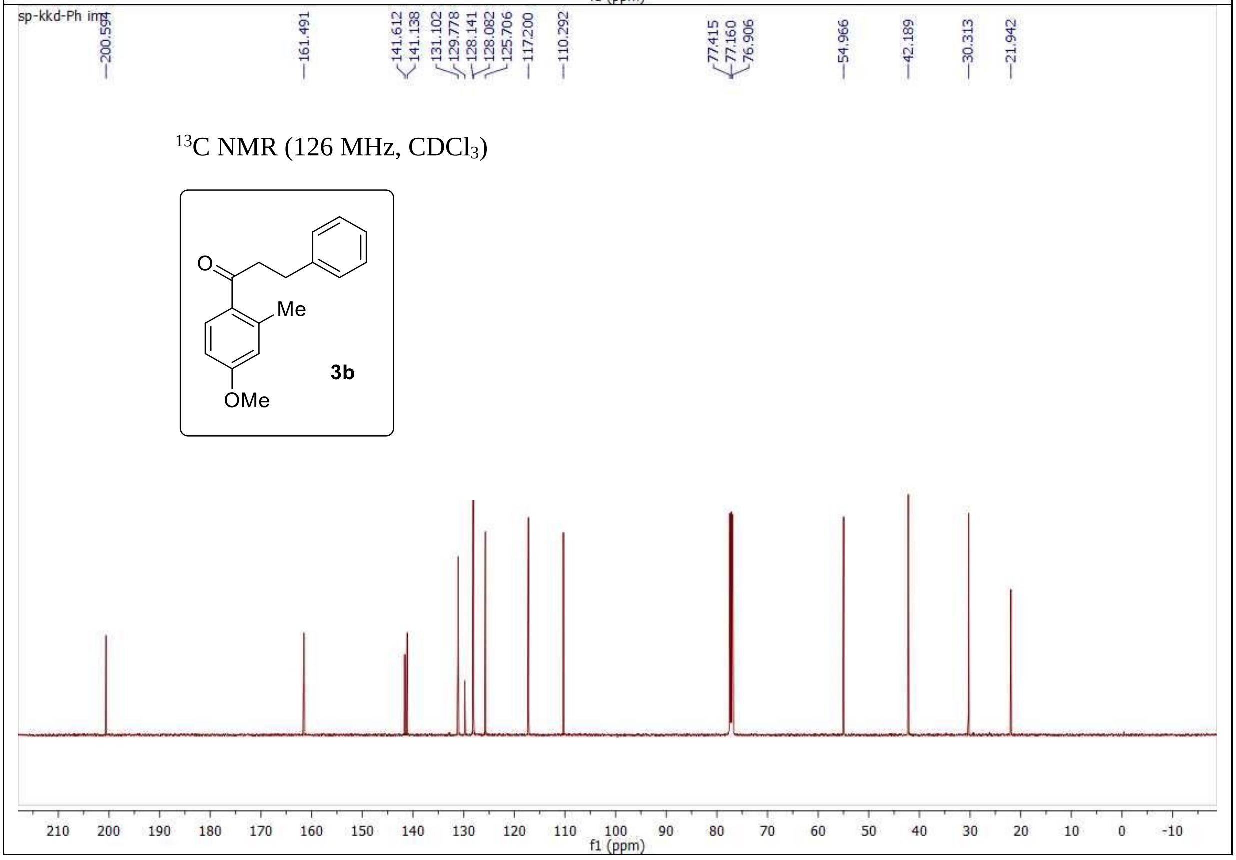
sp-kkd-Ph im
200.594
161.491
141.612
141.138
131.102
129.778
128.141
128.082
125.706
117.200
110.292
77.415
77.160
76.906
54.966
42.189
30.313
21.942
^{13}C NMR (126 MHz, $CDCl_3$)
O
Me
OMe
3b
210 200 190 180 170 160 150 140 130 120 110 100 90 80 70 60 50 40 30 20 10 0 -10
f1 (ppm)

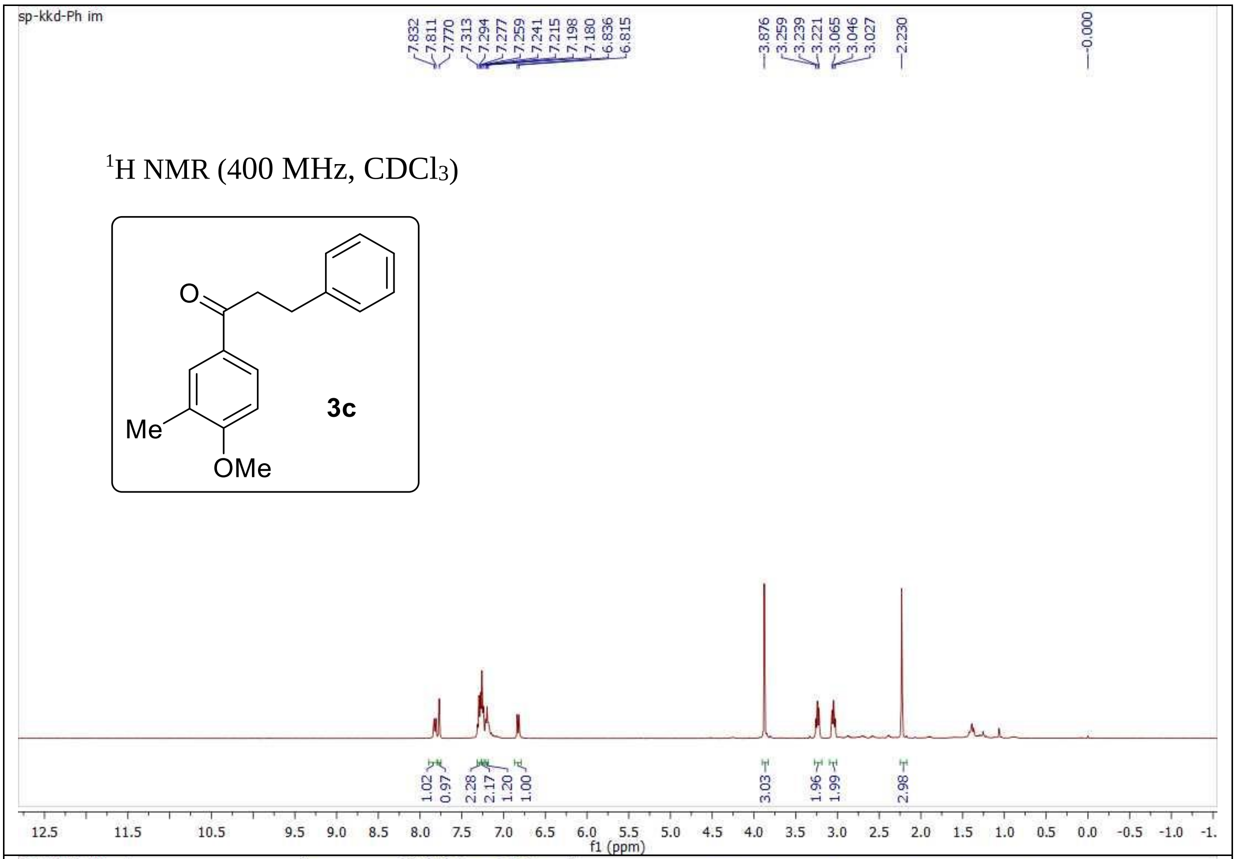
sp-kkd-Ph im
7.832
7.811
7.770
7.313
7.294
7.277
7.259
7.241
7.215
7.198
7.180
6.836
6.815
3.876
3.259
3.239
3.221
3.065
3.046
3.027
2.230
0.000
1H NMR (400 MHz, CDCl3)
O
Me
OMe
3c
1.02
0.97
2.28
2.17
1.20
1.00
3.03
1.96
1.99
2.98
f1 (ppm)

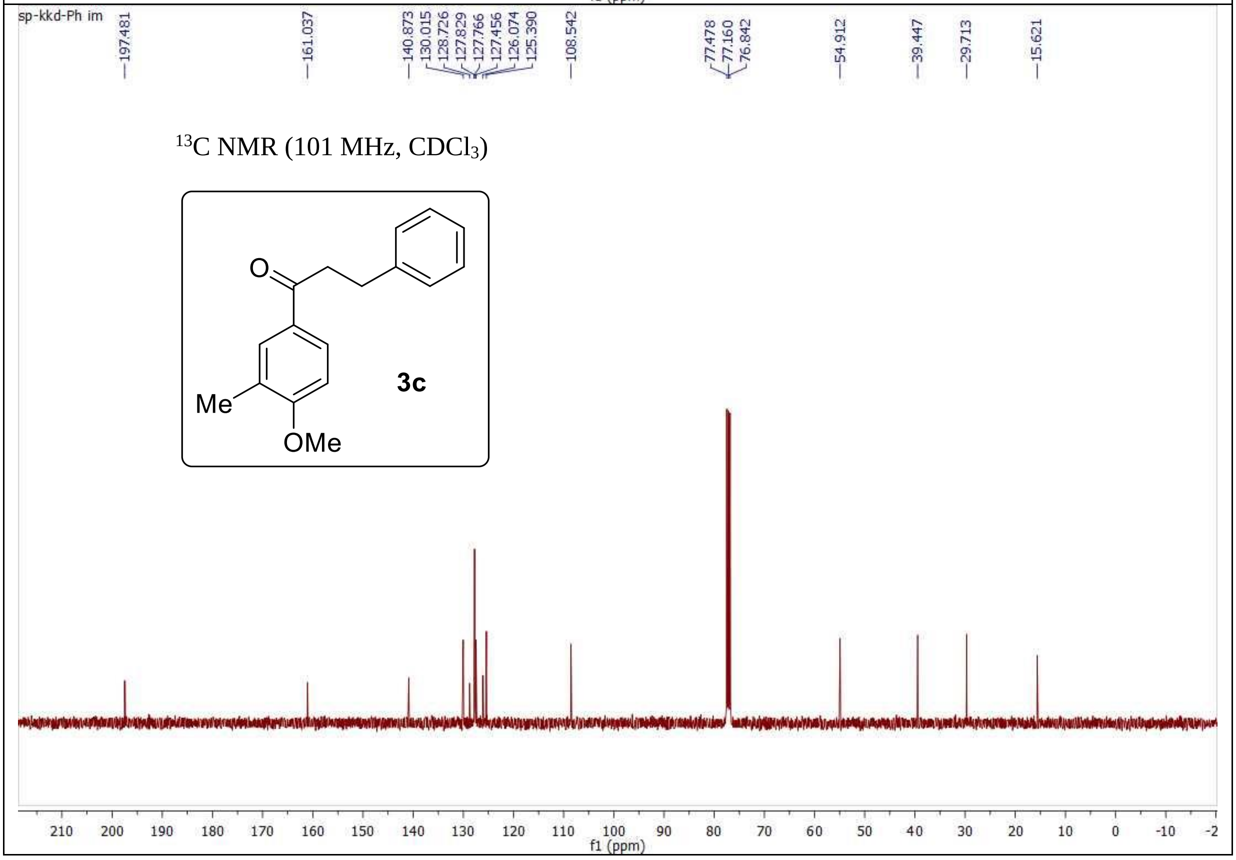
sp-kkd-Ph im
197.481
161.037
140.873
130.015
128.726
127.829
127.766
127.456
126.074
125.390
108.542
77.478
77.160
76.842
54.912
39.447
29.713
15.621
13C NMR (101 MHz, CDCl3)
O
Me
OMe
3c
f1 (ppm)

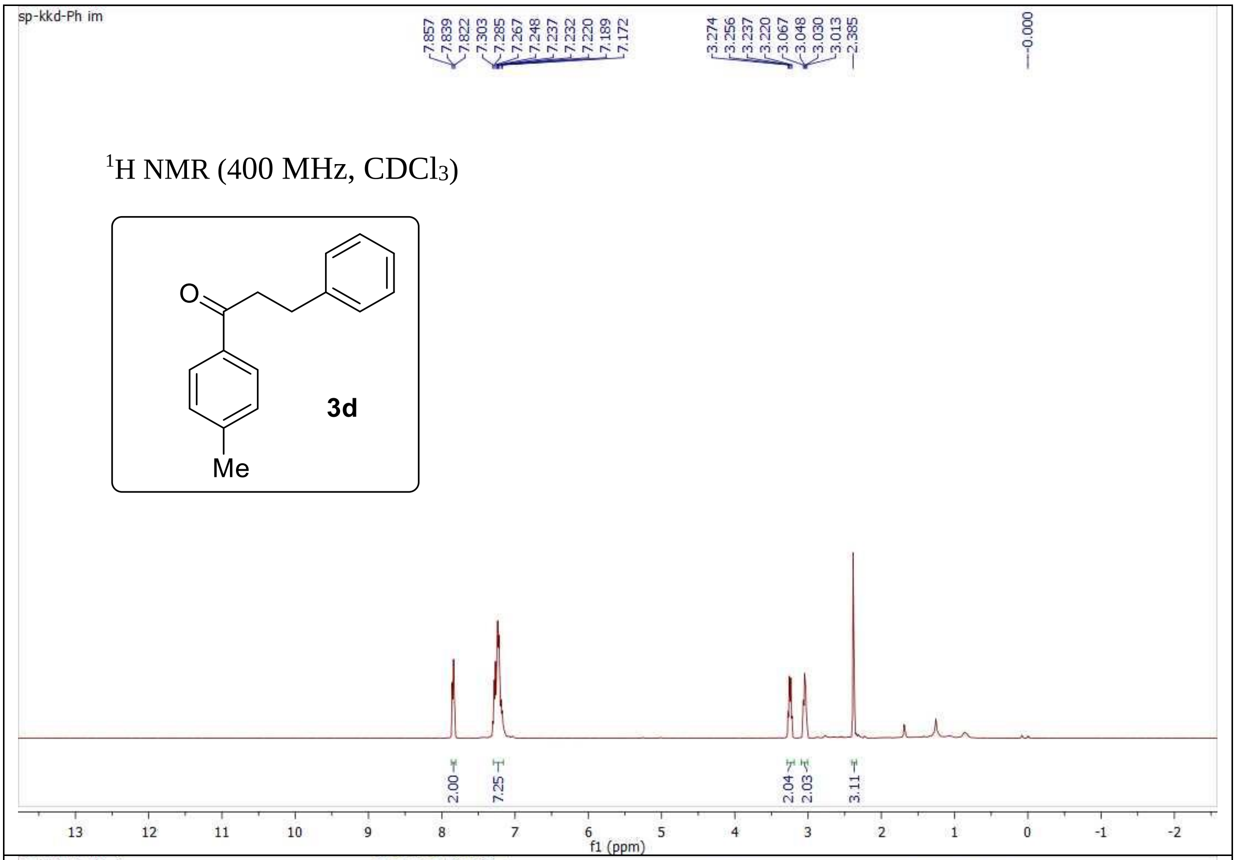
sp-kkd-Ph im
7.857
7.839
7.822
7.303
7.285
7.267
7.248
7.237
7.232
7.220
7.189
7.172
3.274
3.256
3.237
3.220
3.067
3.048
3.030
3.013
2.385
0.000
^{1}H NMR (400 MHz, CDCl$_3$)
O
Me
3d
2.00
7.25
2.04
2.03
3.11
13
12
11
10
9
8
7
6
5
4
3
2
1
0
-1
-2
f1 (ppm)

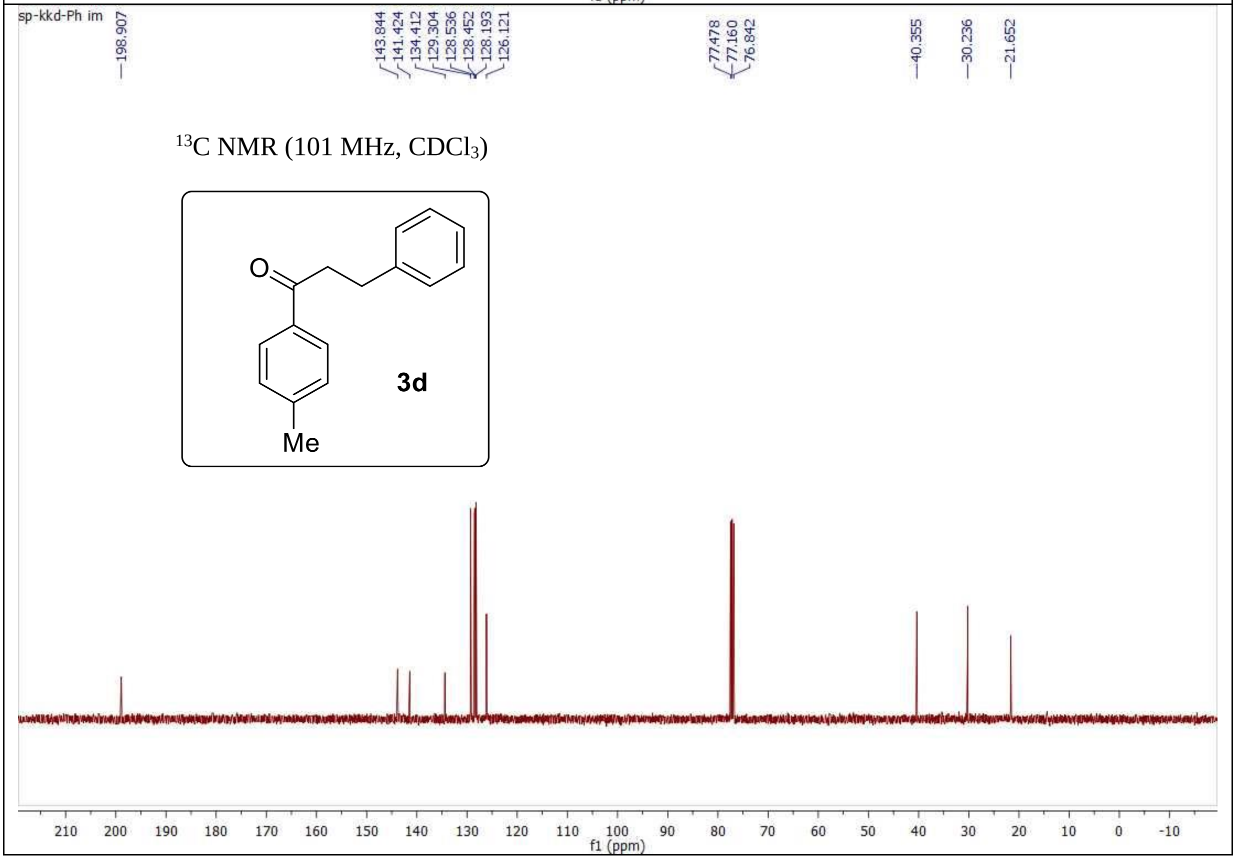
sp-kkd-Ph im
198.907
143.844
141.424
134.412
129.304
128.536
128.452
128.193
126.121
77.478
77.160
76.842
40.355
30.236
21.652
^{13}C NMR (101 MHz, CDCl$_3$)
O
Me
3d
210
200
190
180
170
160
150
140
130
120
110
100
90
80
70
60
50
40
30
20
10
0
-10
f1 (ppm)

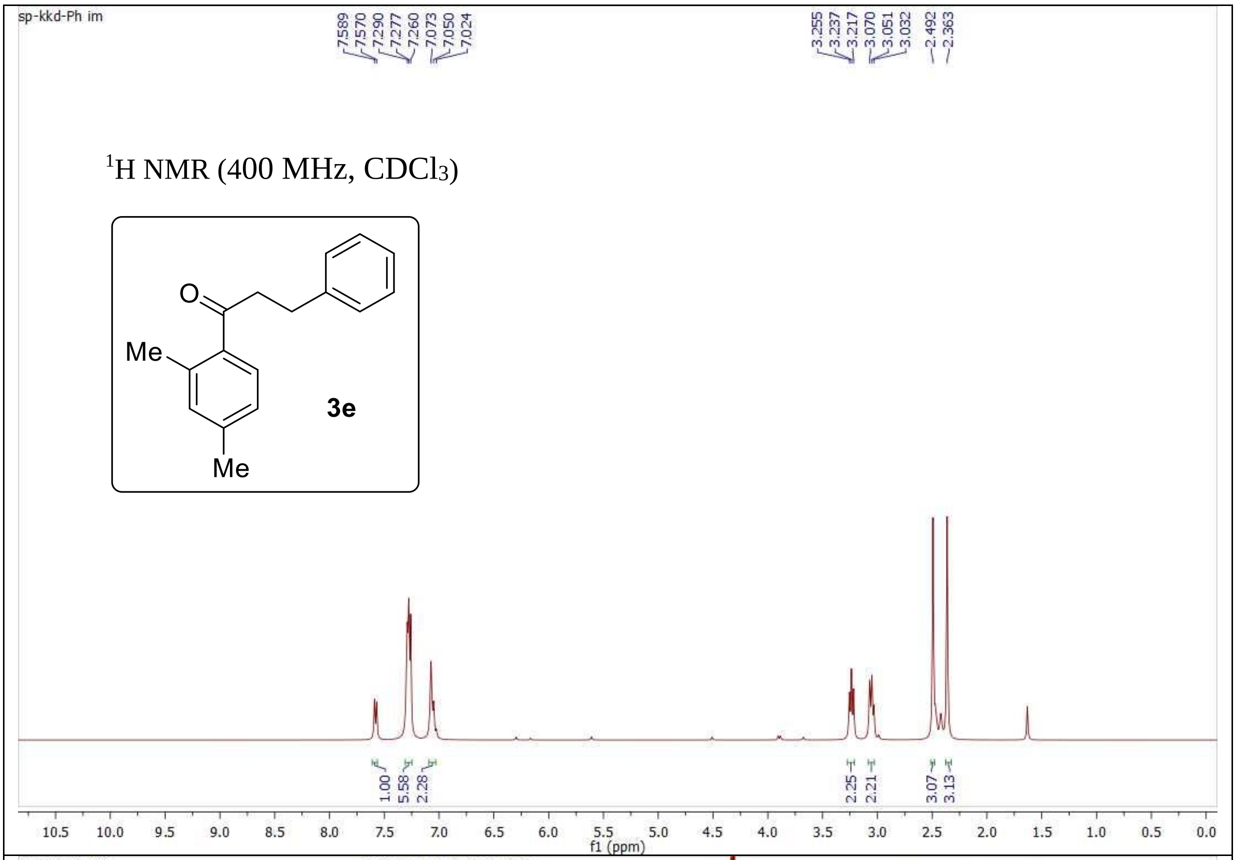
sp-kkd-Ph im
7.589
7.570
7.290
7.277
7.260
7.073
7.050
7.024
3.255
3.237
3.217
3.070
3.051
3.032
2.492
2.363
^{1}H NMR (400 MHz, CDCl$_3$)
O
Me
Me
3e
1.00
5.58
2.28
2.25
2.21
3.07
3.13
10.5 10.0 9.5 9.0 8.5 8.0 7.5 7.0 6.5 6.0 5.5 5.0 4.5 4.0 3.5 3.0 2.5 2.0 1.5 1.0 0.5 0.0
f1 (ppm)

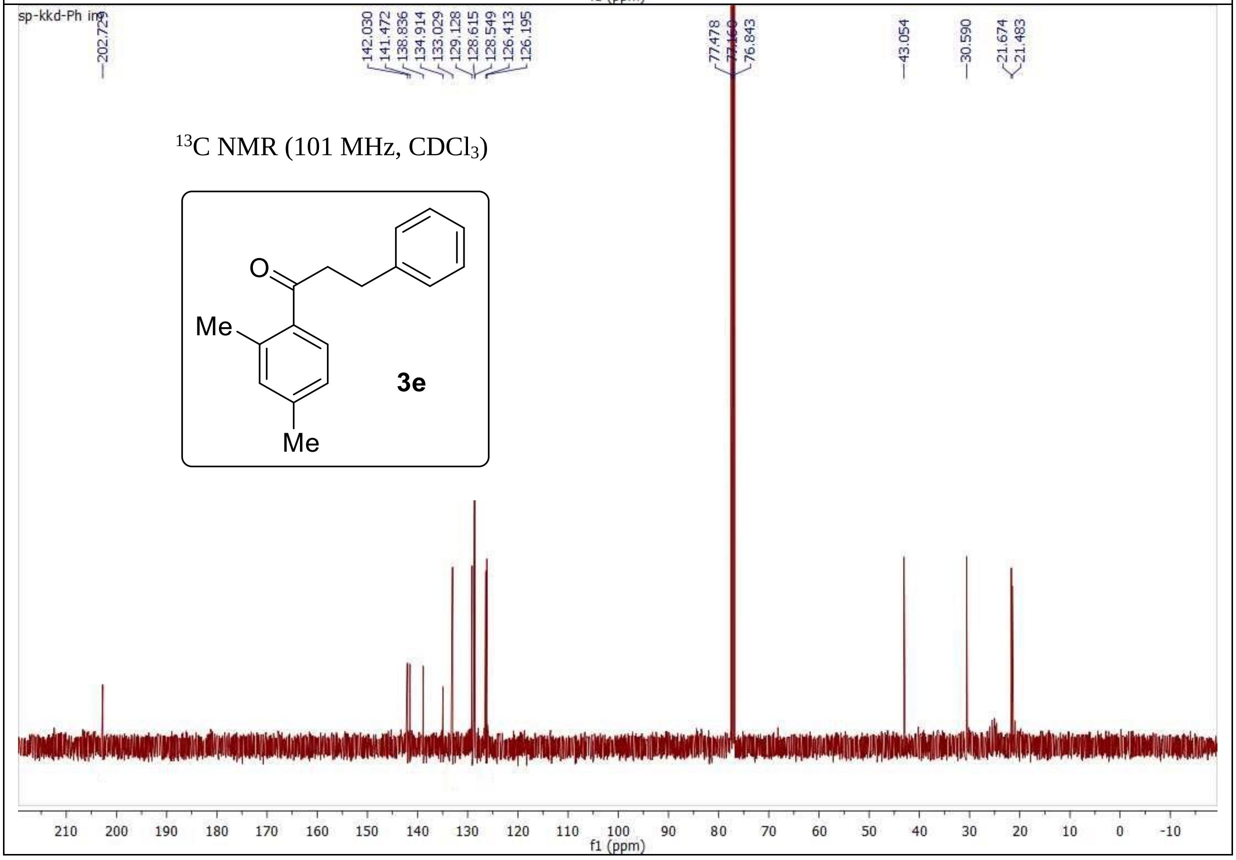
sp-kkd-Ph im
202.729
142.030
141.472
138.836
134.914
133.029
129.128
128.615
128.549
126.413
126.195
77.478
77.160
76.843
43.054
30.590
21.674
21.483
^{13}C NMR (101 MHz, CDCl$_3$)
O
Me
Me
3e
210 200 190 180 170 160 150 140 130 120 110 100 90 80 70 60 50 40 30 20 10 0 -10
f1 (ppm)

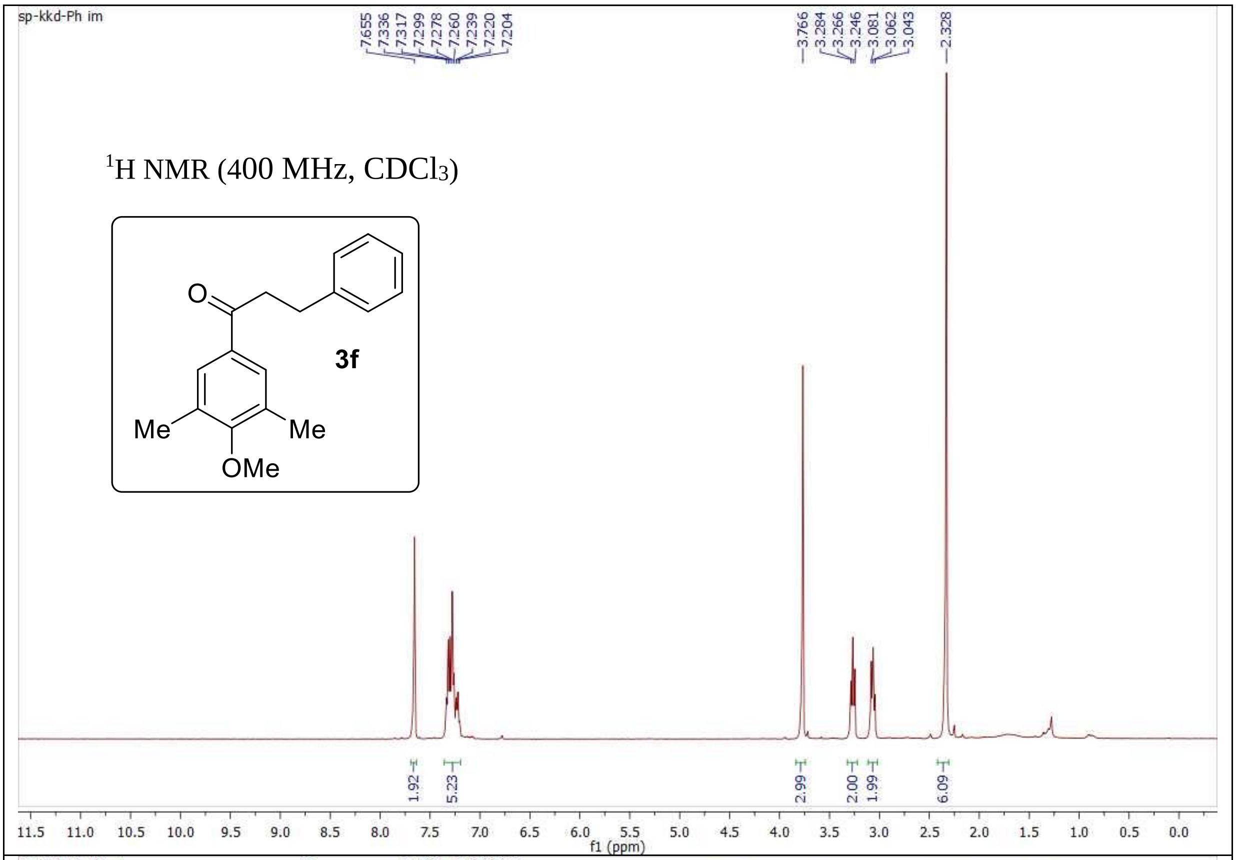

sp-kkd-Ph im
7.655 7.336 7.317 7.299 7.278 7.260 7.239 7.220 7.204
3.766 3.284 3.266 3.246 3.081 3.062 3.043
2.328
1H NMR (400 MHz, CDCl3)
O
3f
Me
Me
OMe
1.92
5.23
2.99
2.00
1.99
6.09
11.5 11.0 10.5 10.0 9.5 9.0 8.5 8.0 7.5 7.0 6.5 6.0 5.5 5.0 4.5 4.0 3.5 3.0 2.5 2.0 1.5 1.0 0.5 0.0
f1 (ppm)

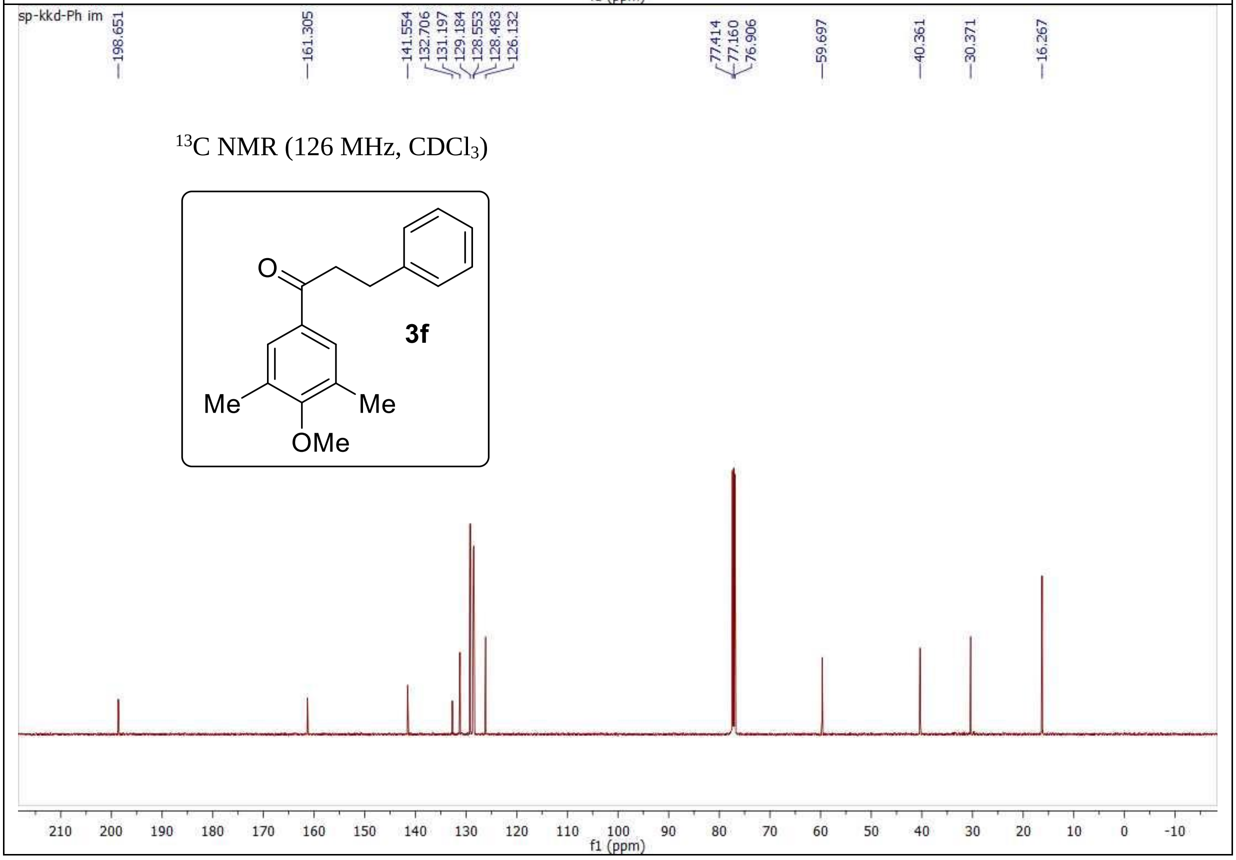

sp-kkd-Ph im
198.651
161.305
141.554 132.706 131.197 129.184 128.553 128.483 126.132
77.414 77.160 76.906
59.697
40.361
30.371
16.267
13C NMR (126 MHz, CDCl3)
O
3f
Me
Me
OMe
210 200 190 180 170 160 150 140 130 120 110 100 90 80 70 60 50 40 30 20 10 0 -10
f1 (ppm)

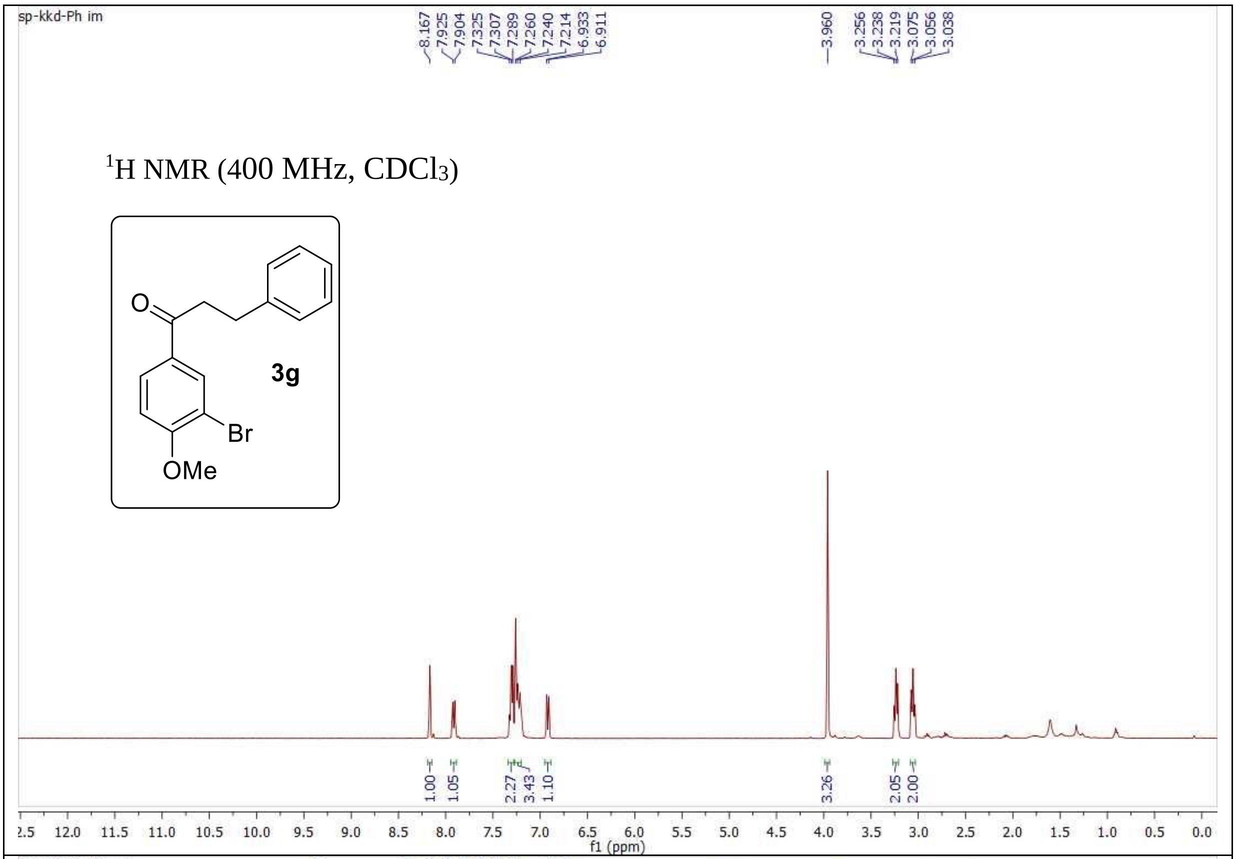
sp-kkd-Ph im
8.167
7.925
7.904
7.325
7.307
7.289
7.260
7.240
7.214
6.933
6.911
3.960
3.256
3.238
3.219
3.075
3.056
3.038
1H NMR (400 MHz, CDCl3)
O
3g
Br
OMe
1.00
1.05
2.27
3.43
1.10
3.26
2.05
2.00
f1 (ppm)

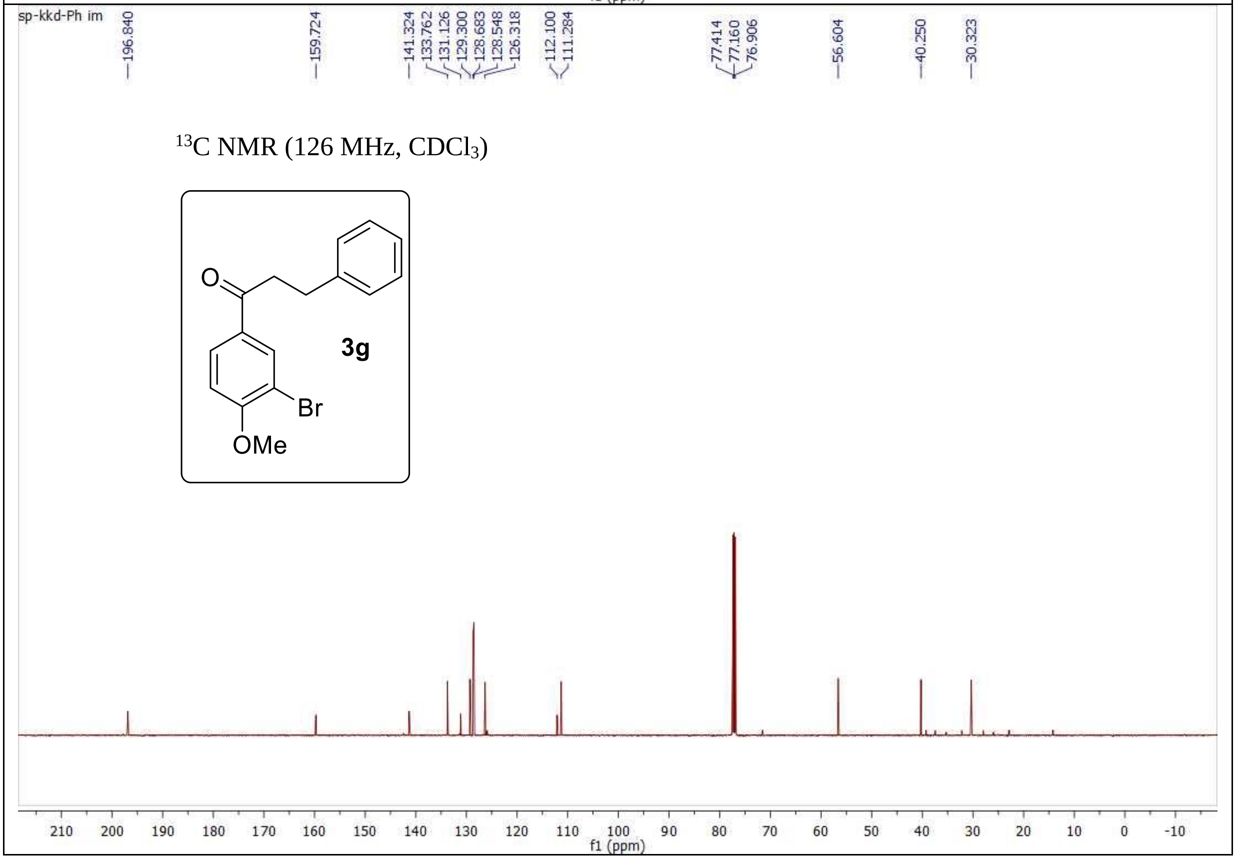
sp-kkd-Ph im
196.840
159.724
141.324
133.762
131.126
129.300
128.683
128.548
126.318
112.100
111.284
77.414
77.160
76.906
56.604
40.250
30.323
13C NMR (126 MHz, CDCl3)
O
3g
Br
OMe
f1 (ppm)

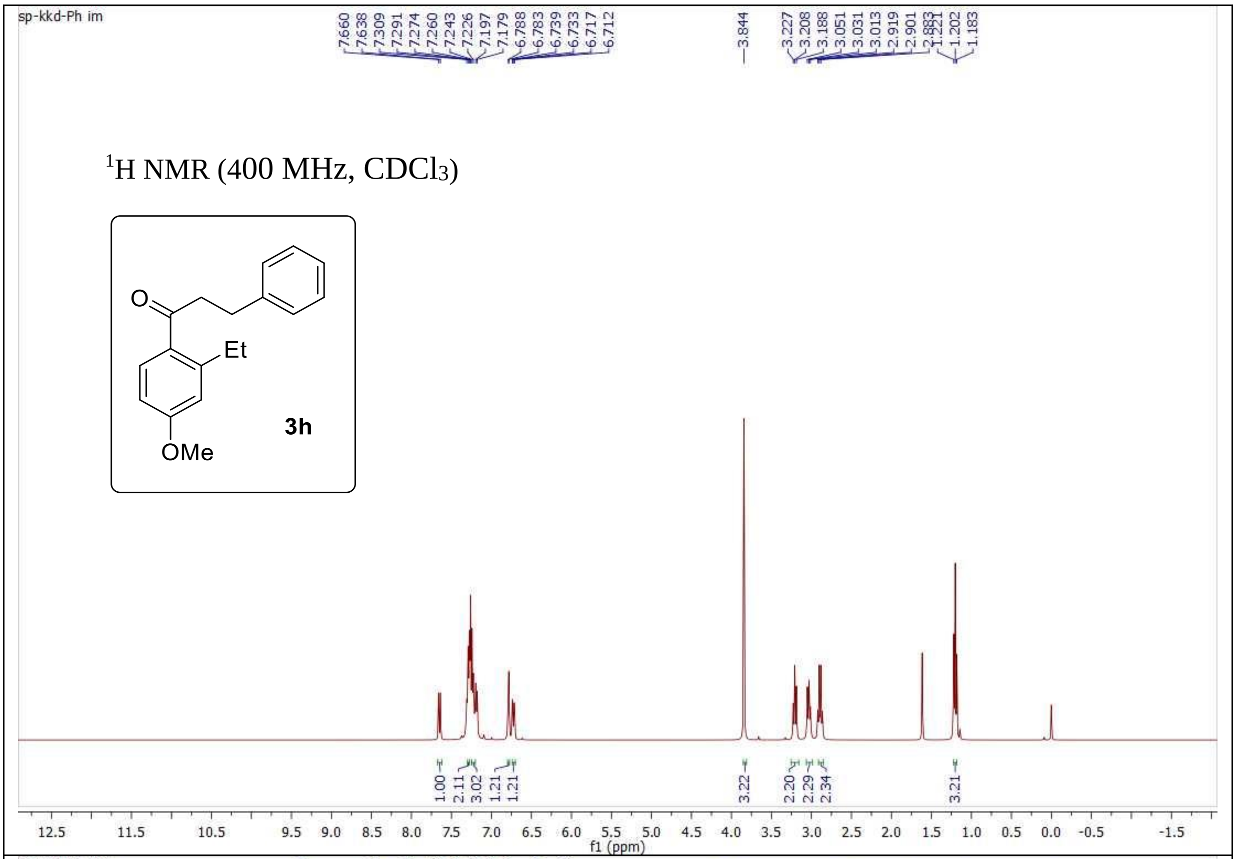

sp-kkd-Ph im
¹H NMR (400 MHz, CDCl3)
Et
O
OMe
3h
f1 (ppm)

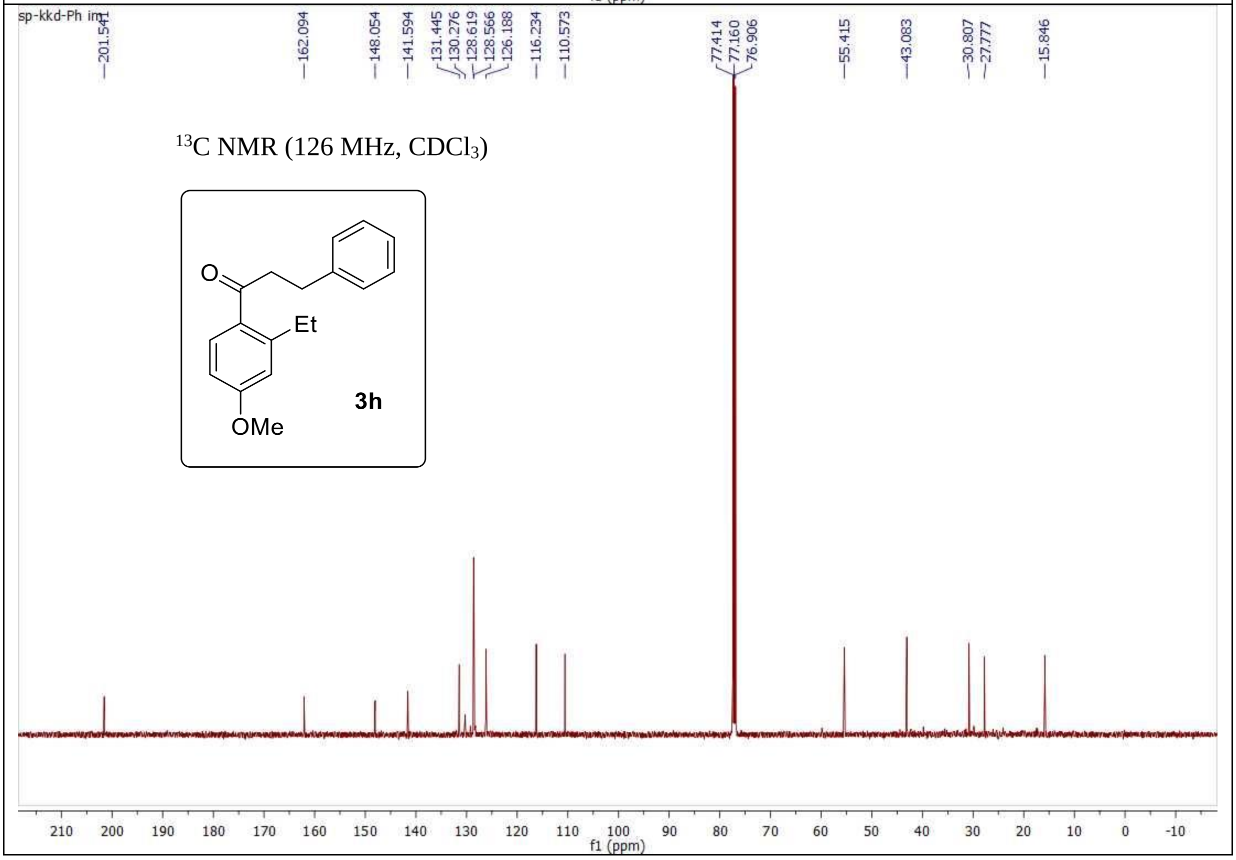

sp-kkd-Ph im
¹³C NMR (126 MHz, CDCl3)
Et
O
OMe
3h
f1 (ppm)

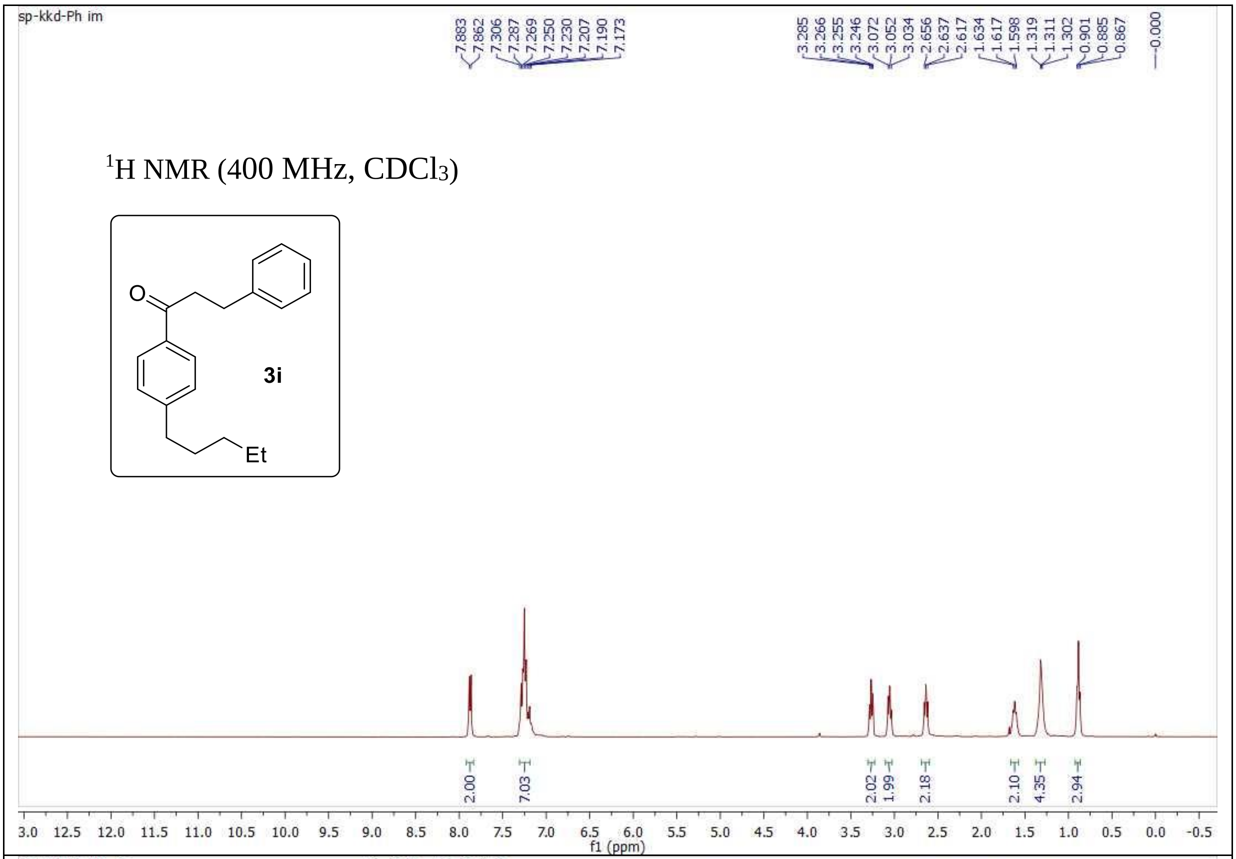
sp-kkd-Ph im
7.883
7.862
7.306
7.287
7.269
7.250
7.230
7.207
7.190
7.173
3.285
3.266
3.255
3.246
3.072
3.052
3.034
2.656
2.637
2.617
1.634
1.617
1.598
1.319
1.311
1.302
0.901
0.885
0.867
0.000
¹H NMR (400 MHz, CDCl3)
O
3i
Et
2.00
7.03
2.02
1.99
2.18
2.10
4.35
2.94
f1 (ppm)

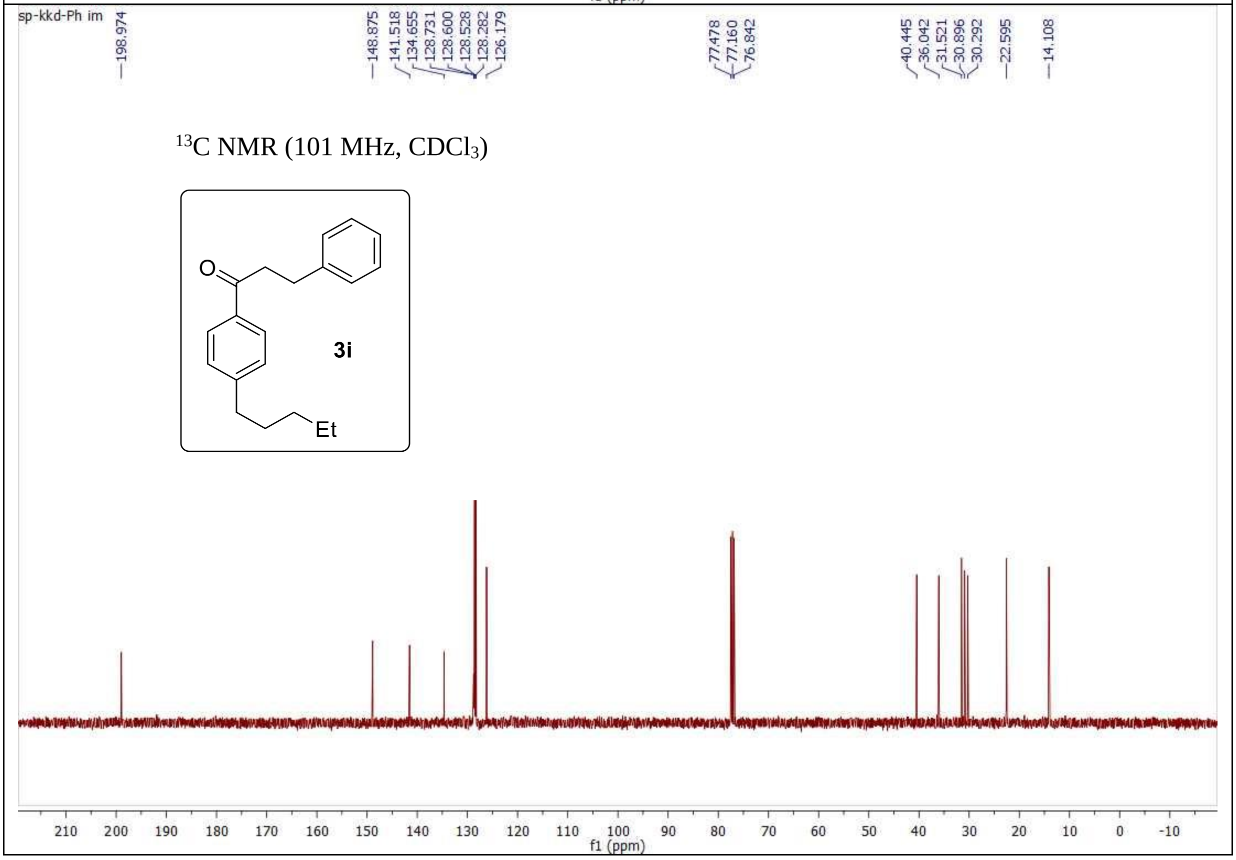
sp-kkd-Ph im
198.974
148.875
141.518
134.655
128.731
128.600
128.528
128.282
126.179
77.478
77.160
76.842
40.445
36.042
31.521
30.896
30.292
22.595
14.108
¹³C NMR (101 MHz, CDCl3)
O
3i
Et
f1 (ppm)

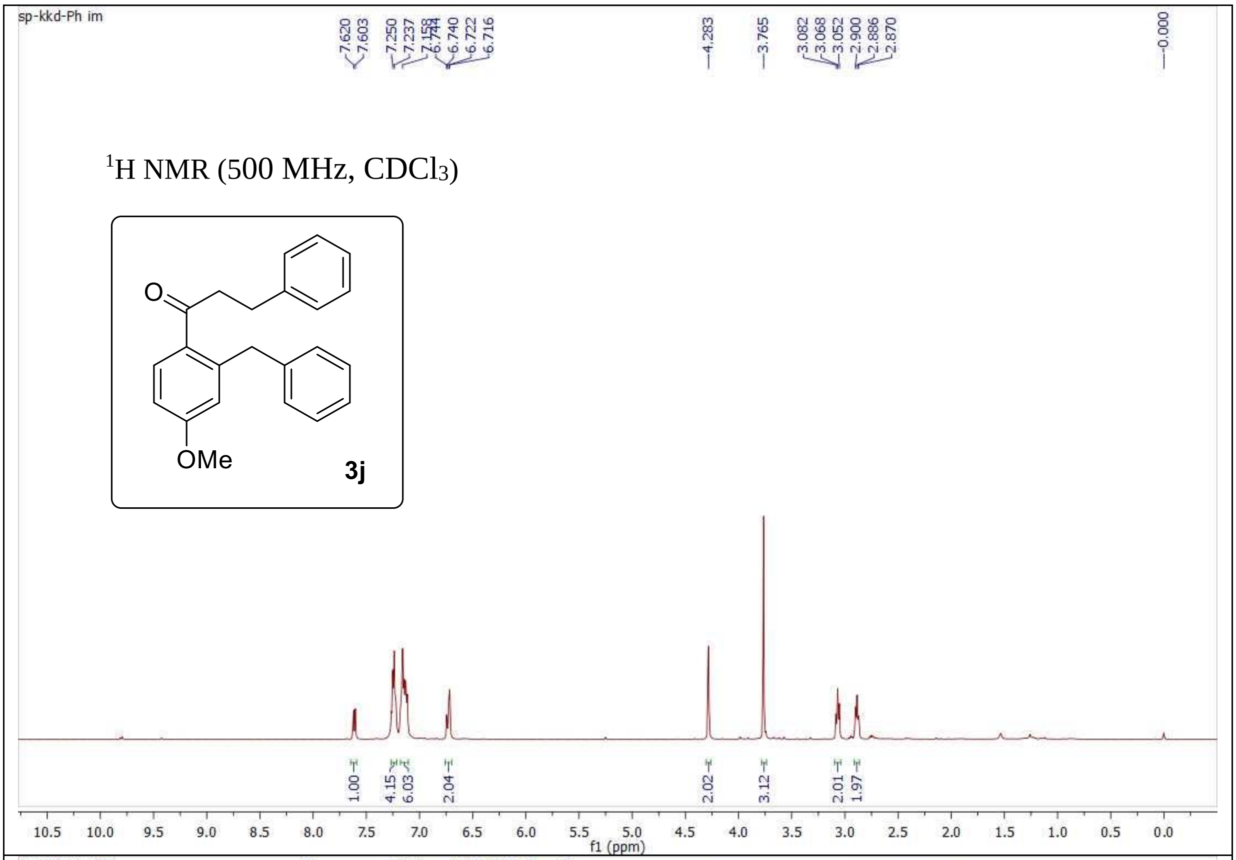
sp-kkd-Ph im
7.620
7.603
7.250
7.237
7.158
7.144
6.740
6.722
6.716
4.283
3.765
3.082
3.068
3.052
2.900
2.886
2.870
0.000
1H NMR (500 MHz, CDCl3)
O
OMe
3j
1.00
4.15
6.03
2.04
2.02
3.12
2.01
1.97
f1 (ppm)

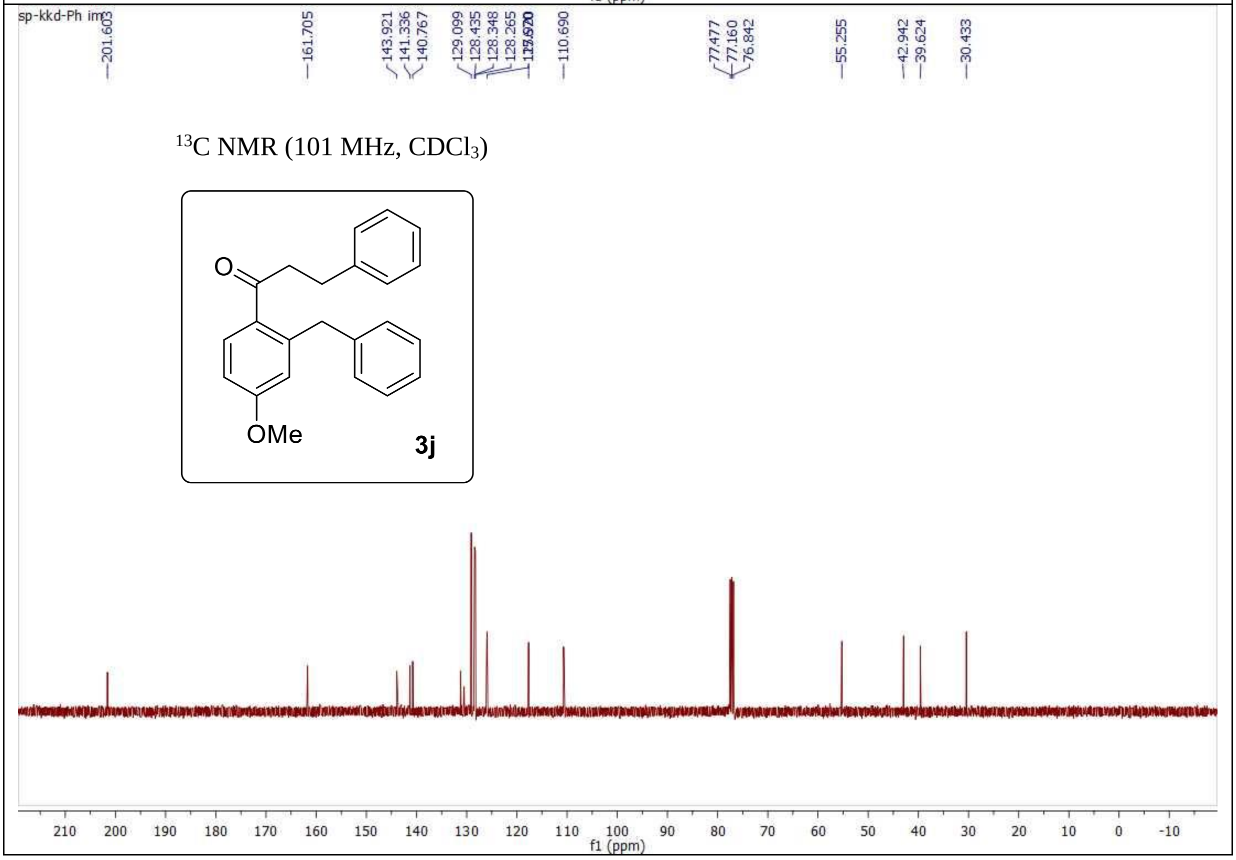
sp-kkd-Ph im
201.603
161.705
143.921
141.336
140.767
129.099
128.435
128.348
128.265
110.690
77.477
77.160
76.842
55.255
42.942
39.624
30.433
13C NMR (101 MHz, CDCl3)
O
OMe
3j
f1 (ppm)

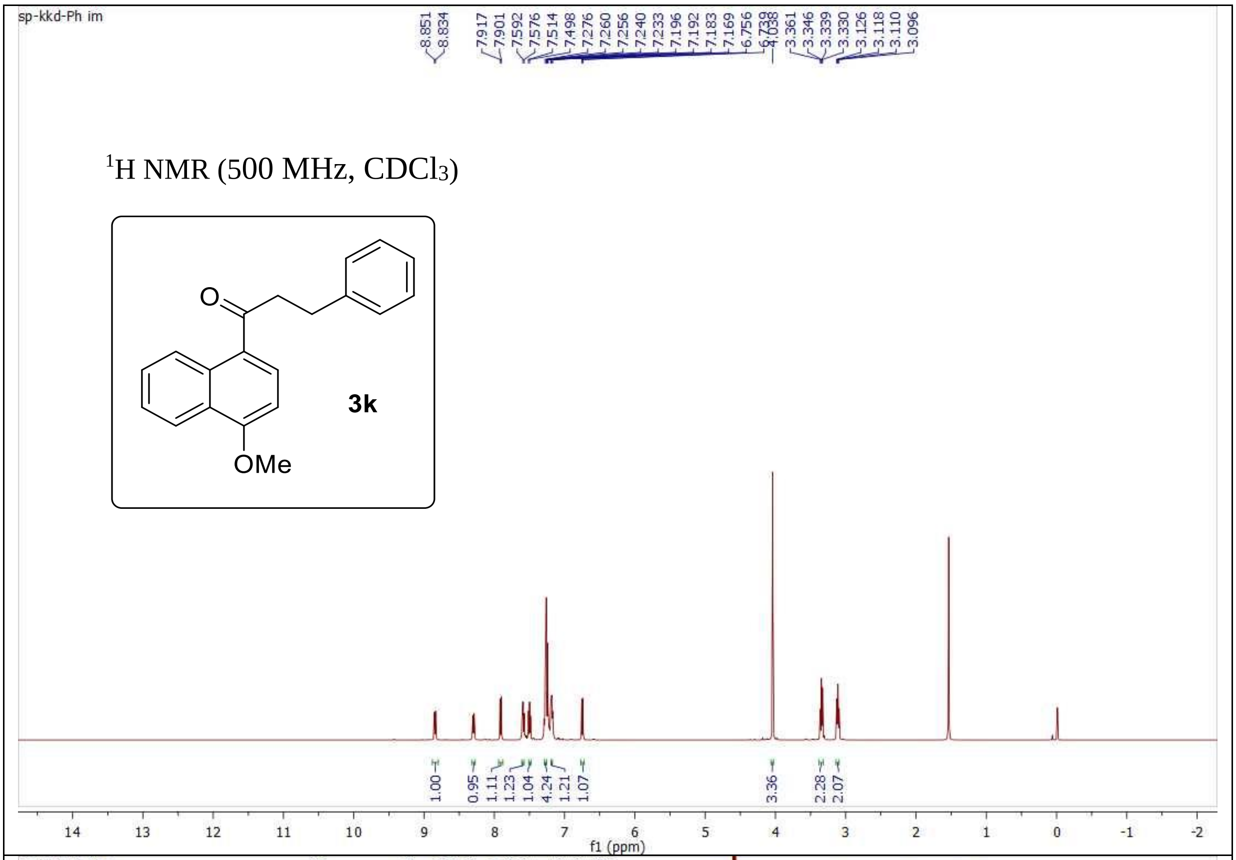
sp-kkd-Ph im
1H NMR (500 MHz, CDCl3)
O
OMe
3k
f1 (ppm)

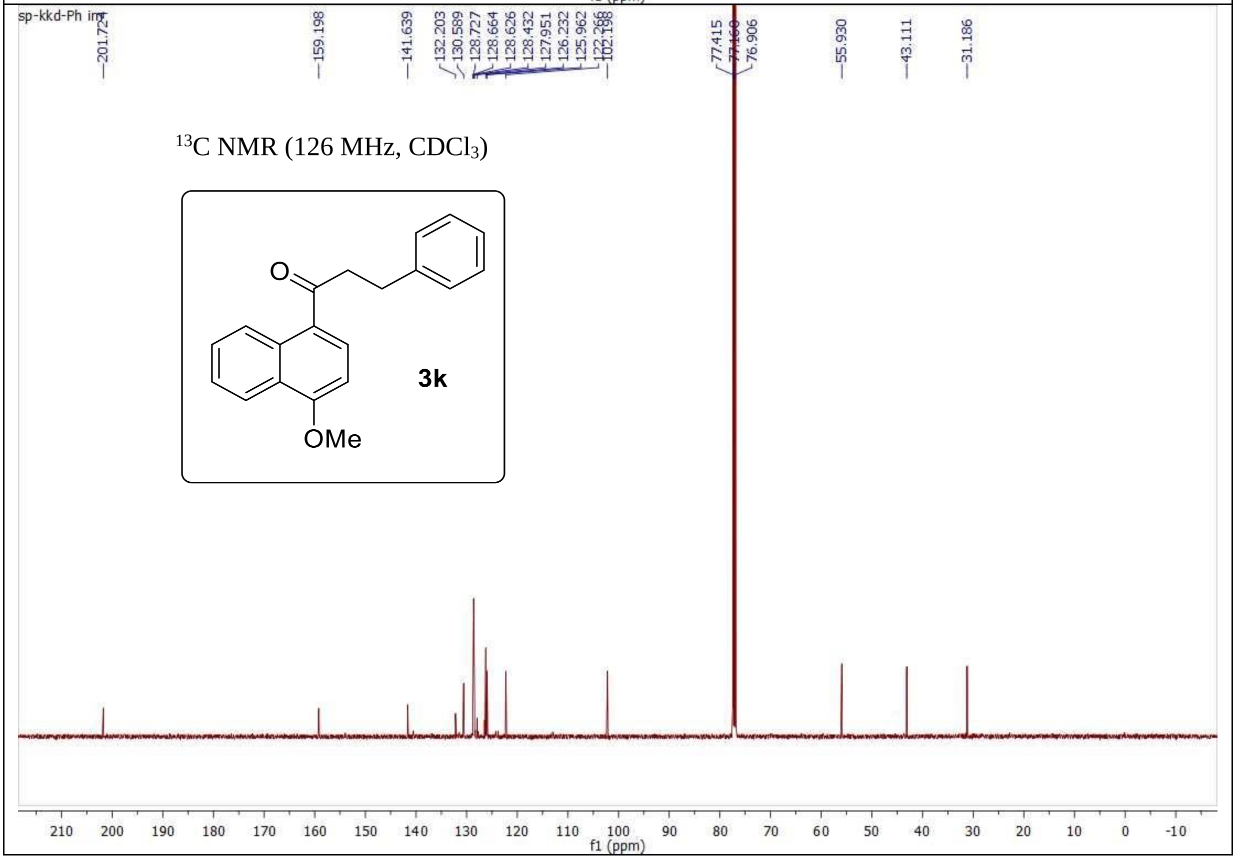
13C NMR (126 MHz, CDCl3)
O
OMe
3k
f1 (ppm)

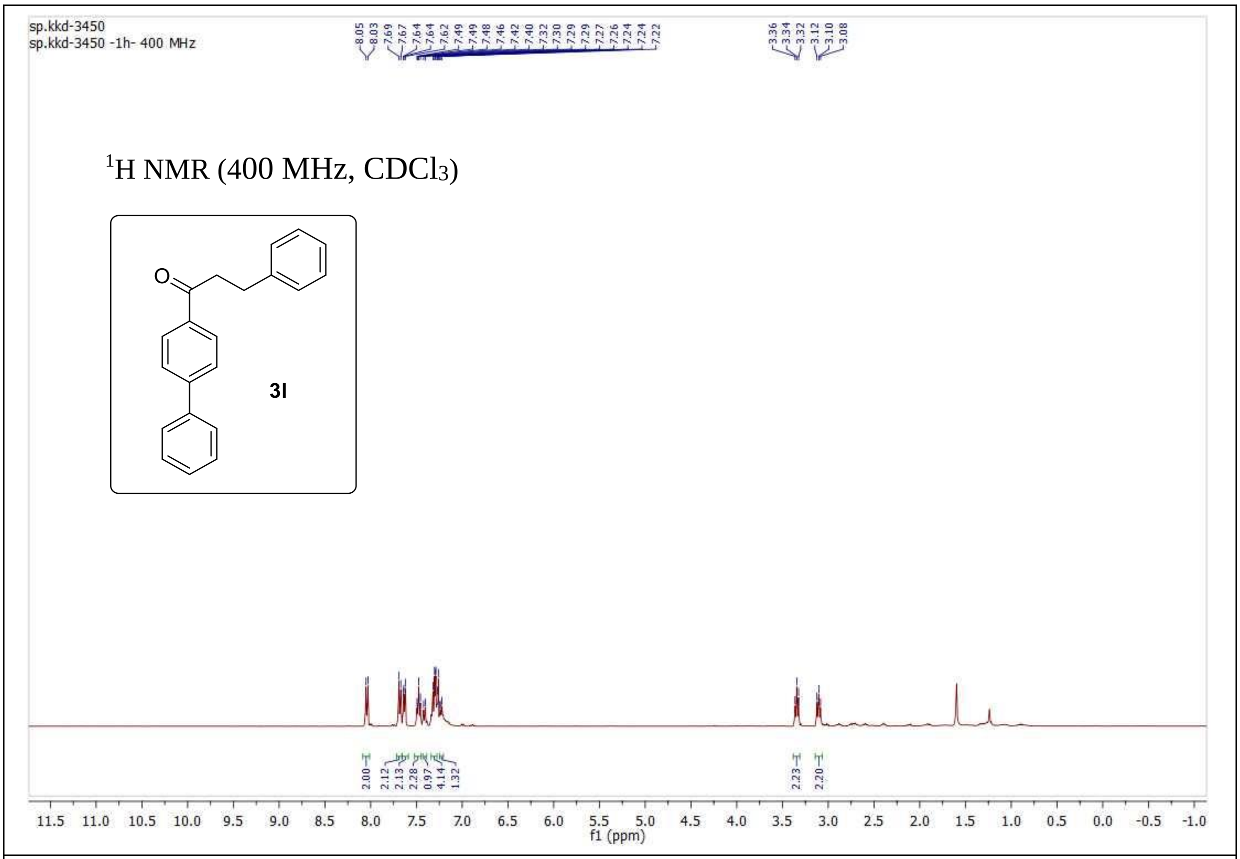
sp.kkd-3450
sp.kkd-3450 -1h- 400 MHz
8.05
8.03
7.69
7.67
7.64
7.64
7.62
7.49
7.49
7.48
7.46
7.42
7.40
7.32
7.30
7.29
7.29
7.27
7.26
7.24
7.24
7.22
3.36
3.34
3.32
3.12
3.10
3.08
1H NMR (400 MHz, CDCl3)
O
3l
2.00
2.12
2.13
2.28
0.97
4.14
1.32
2.23
2.20
11.5 11.0 10.5 10.0 9.5 9.0 8.5 8.0 7.5 7.0 6.5 6.0 5.5 5.0 4.5 4.0 3.5 3.0 2.5 2.0 1.5 1.0 0.5 0.0 -0.5 -1.0
f1 (ppm)

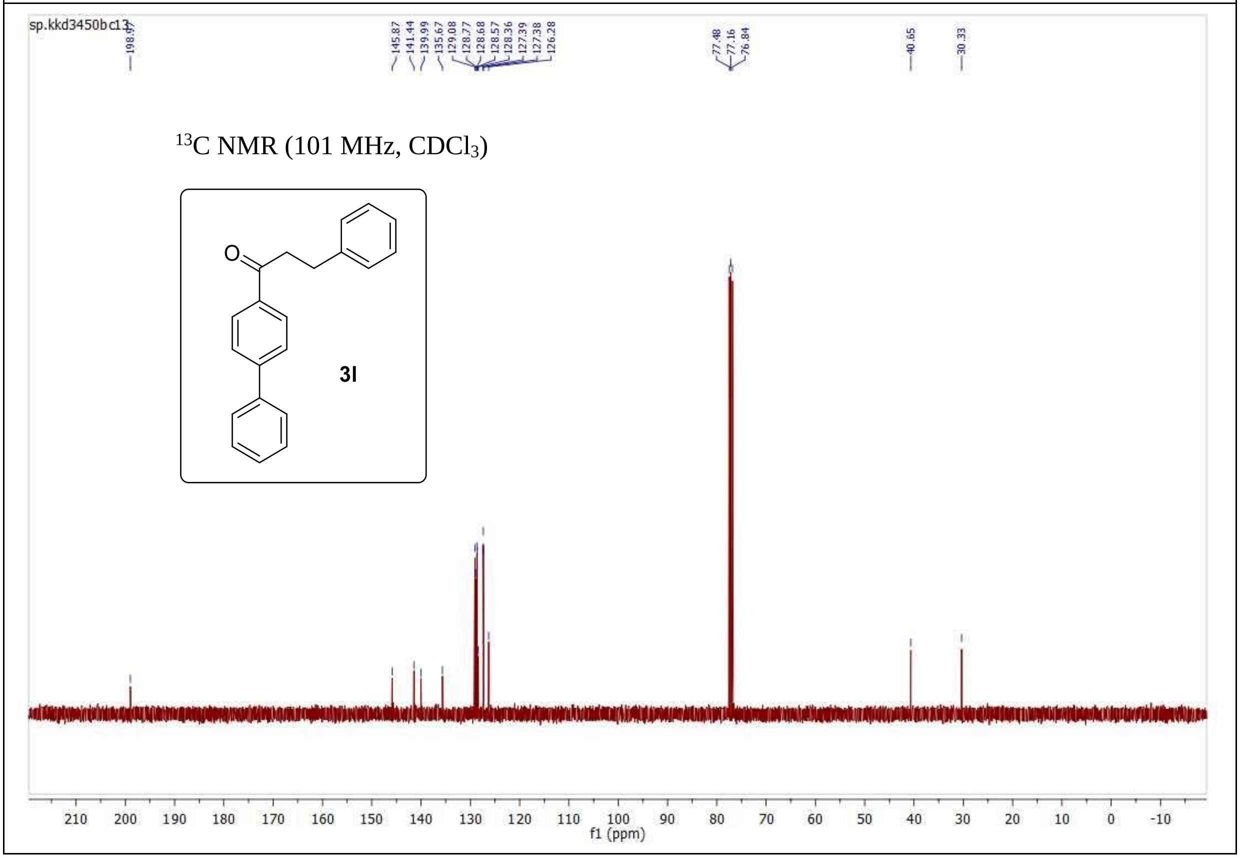
sp.kkd3450bc13
198.97
145.87
141.44
139.99
135.67
129.08
128.77
128.68
128.57
128.36
127.39
127.38
126.28
77.48
77.16
76.84
40.65
30.33
13C NMR (101 MHz, CDCl3)
O
3l
210 200 190 180 170 160 150 140 130 120 110 100 90 80 70 60 50 40 30 20 10 0 -10
f1 (ppm)

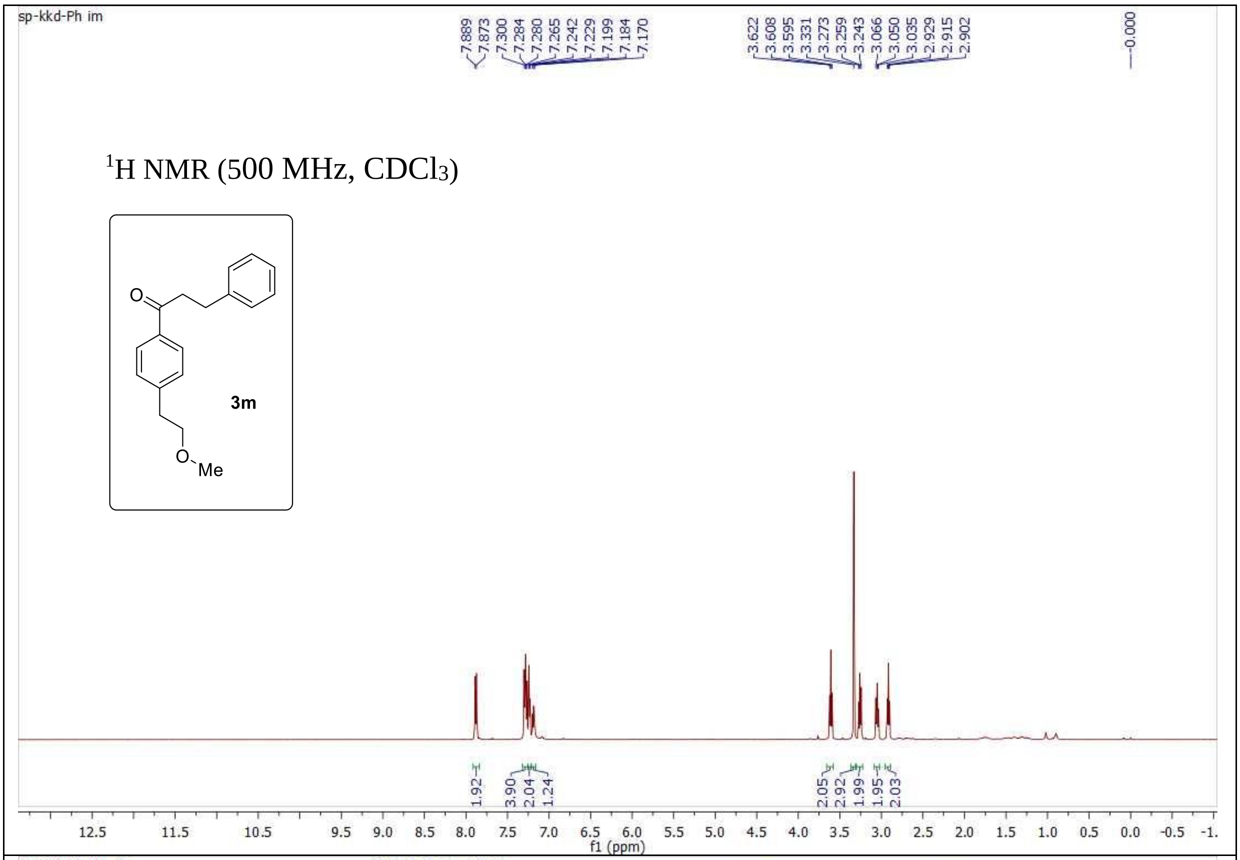
sp-kkd-Ph im
7.889
7.873
7.300
7.284
7.280
7.265
7.242
7.229
7.199
7.184
7.170
3.622
3.608
3.595
3.331
3.273
3.259
3.243
3.066
3.050
3.035
2.929
2.915
2.902
0.000
¹H NMR (500 MHz, CDCl3)
O
3m
O
Me
1.92
3.90
2.04
1.24
2.05
2.92
1.99
1.95
2.03
f1 (ppm)

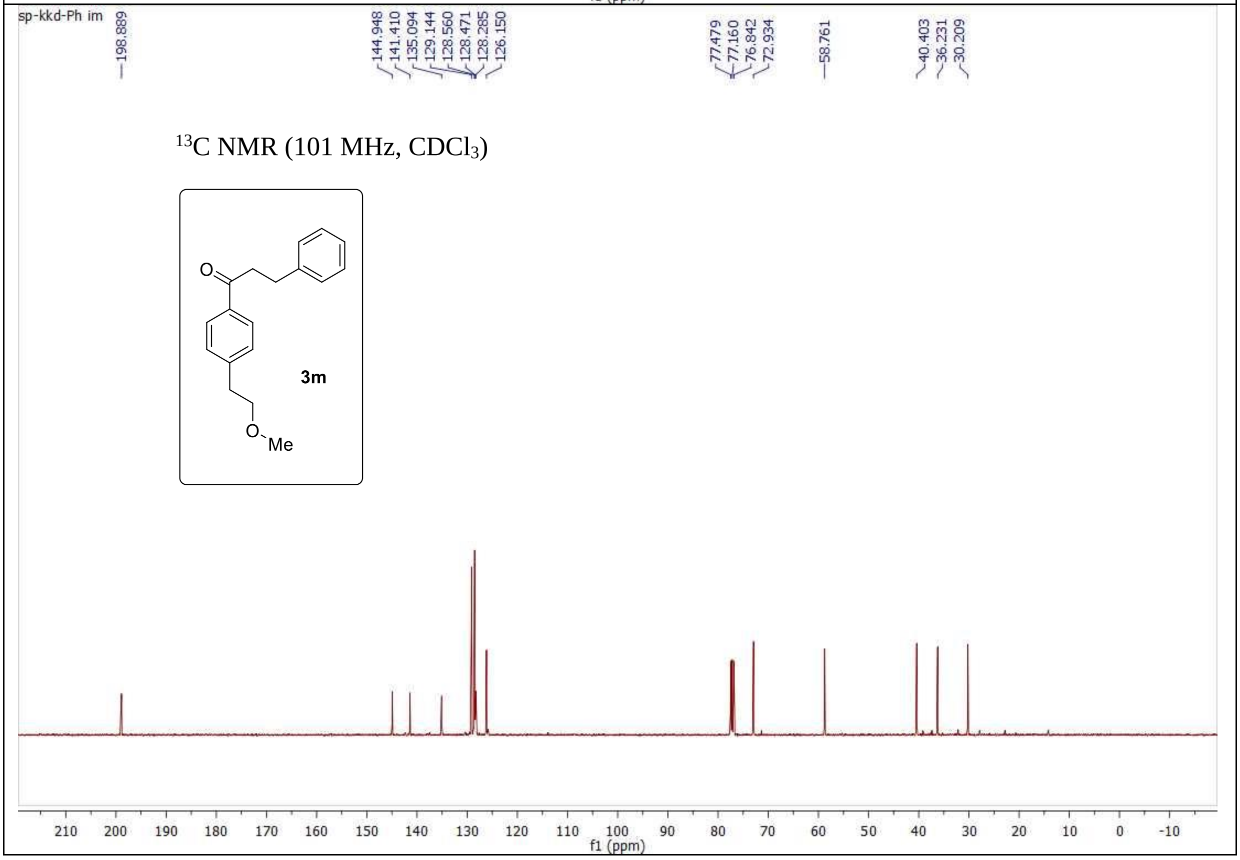
sp-kkd-Ph im
198.889
144.948
141.410
135.094
129.144
128.560
128.471
128.285
126.150
77.479
77.160
76.842
72.934
58.761
40.403
36.231
30.209
¹³C NMR (101 MHz, CDCl3)
O
3m
O
Me
f1 (ppm)

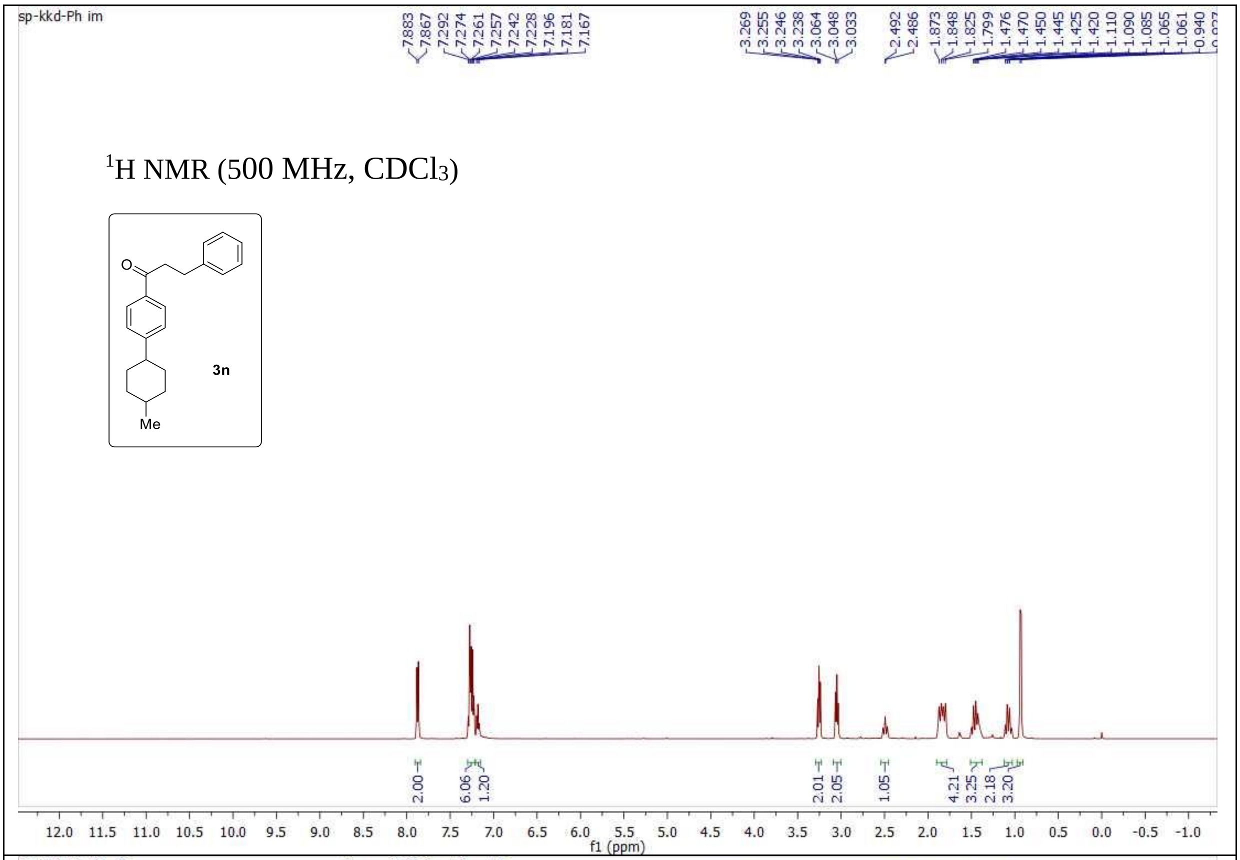
sp-kkd-Ph im
1H NMR (500 MHz, CDCl3)
3n
Me
f1 (ppm)

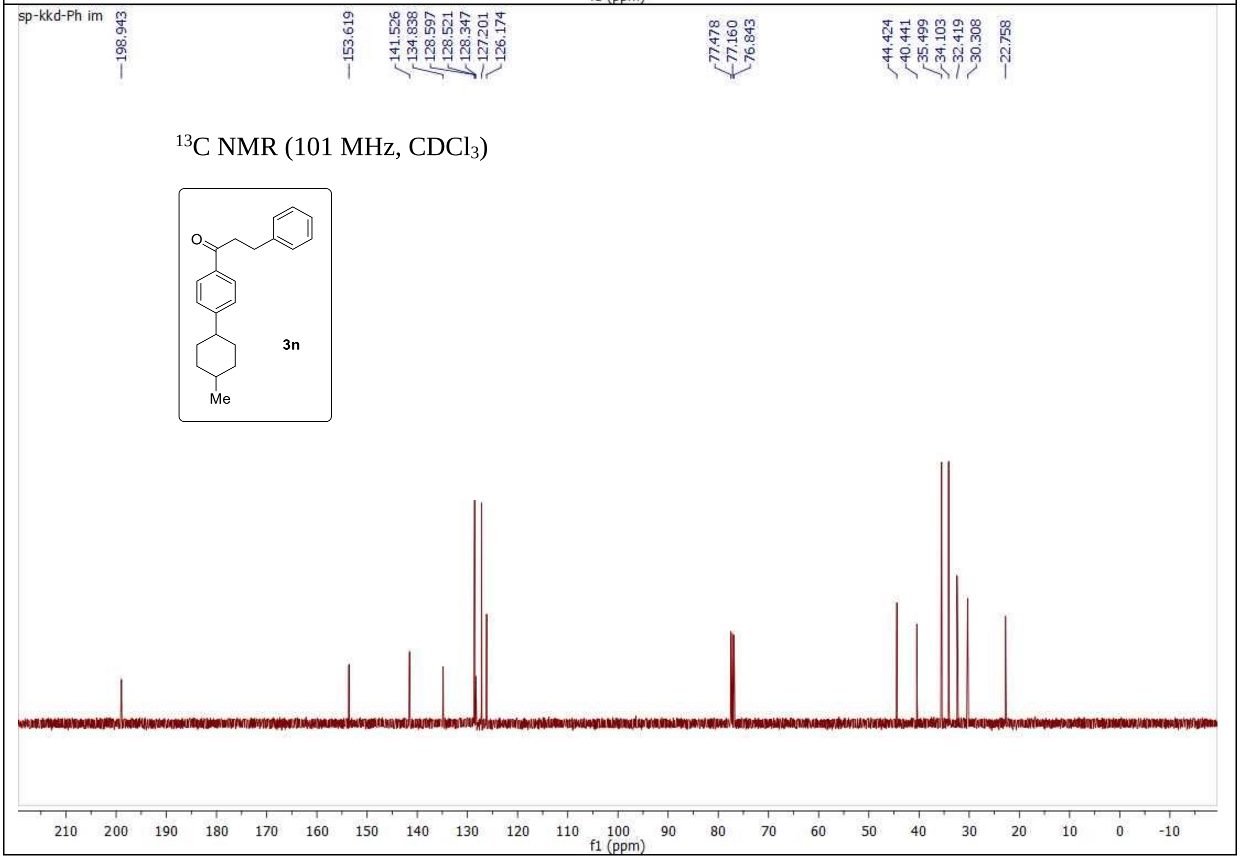
sp-kkd-Ph im
13C NMR (101 MHz, CDCl3)
3n
Me
f1 (ppm)

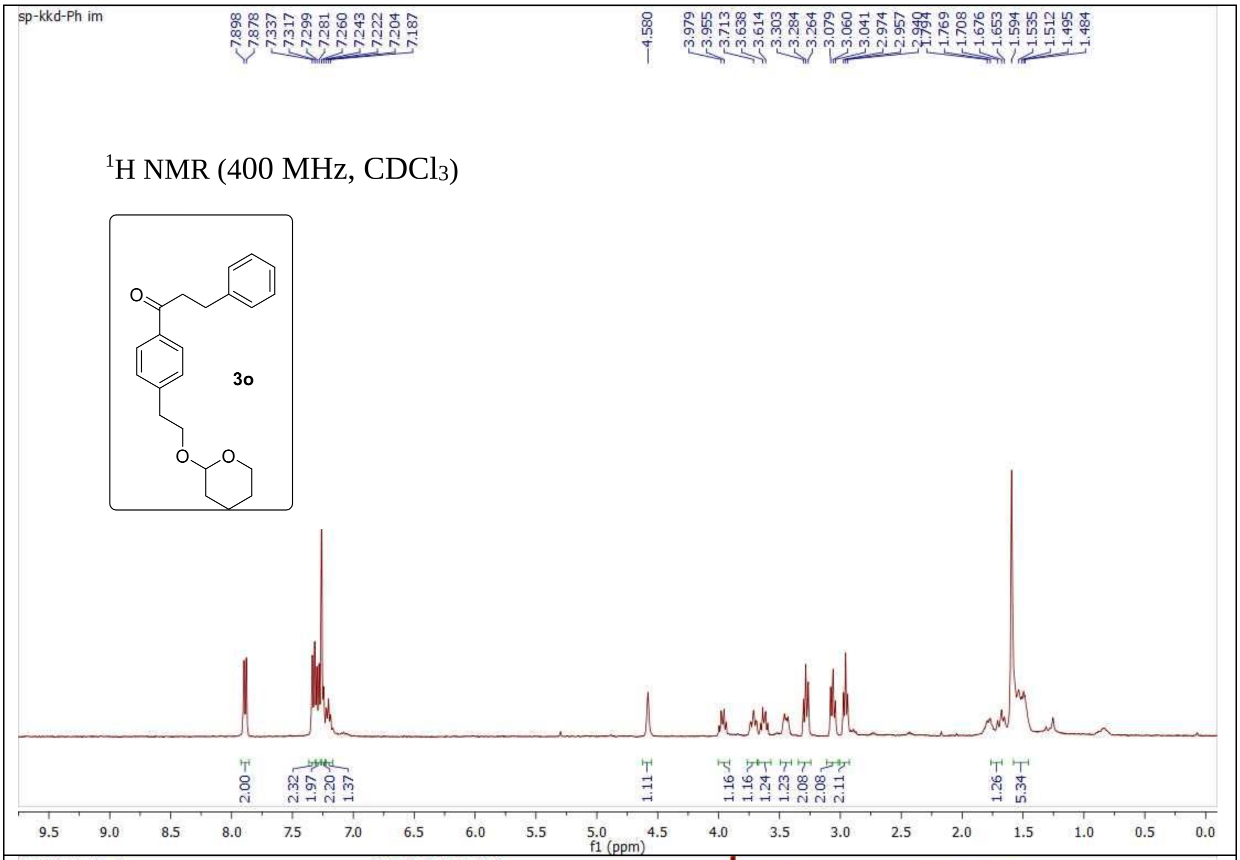

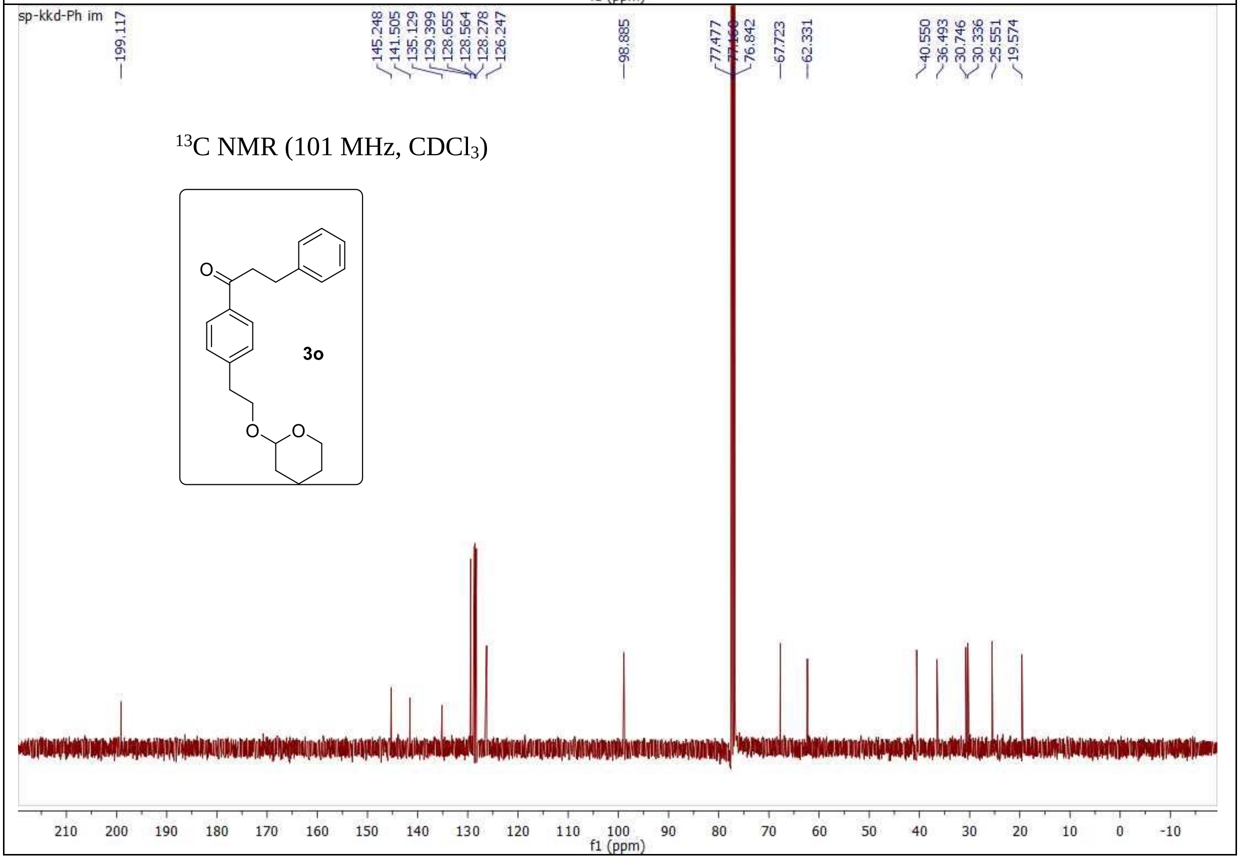

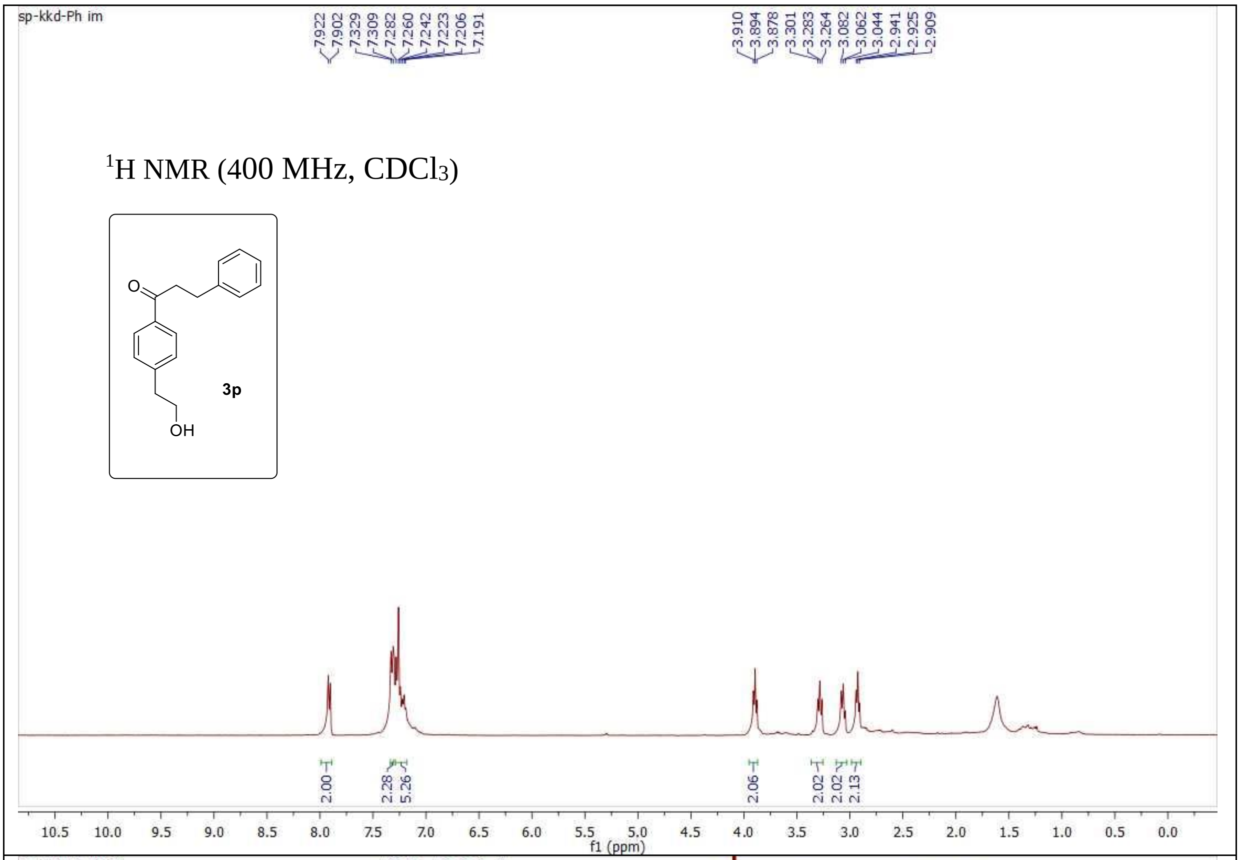
sp-kkd-Ph im
7.922
7.902
7.329
7.309
7.282
7.260
7.242
7.223
7.206
7.191
3.910
3.894
3.878
3.301
3.283
3.264
3.082
3.062
3.044
2.941
2.925
2.909
^{1}H NMR (400 MHz, $CDCl_3$)
O
OH
3p
2.00
2.28
5.26
2.06
2.02
2.02
2.13
f1 (ppm)

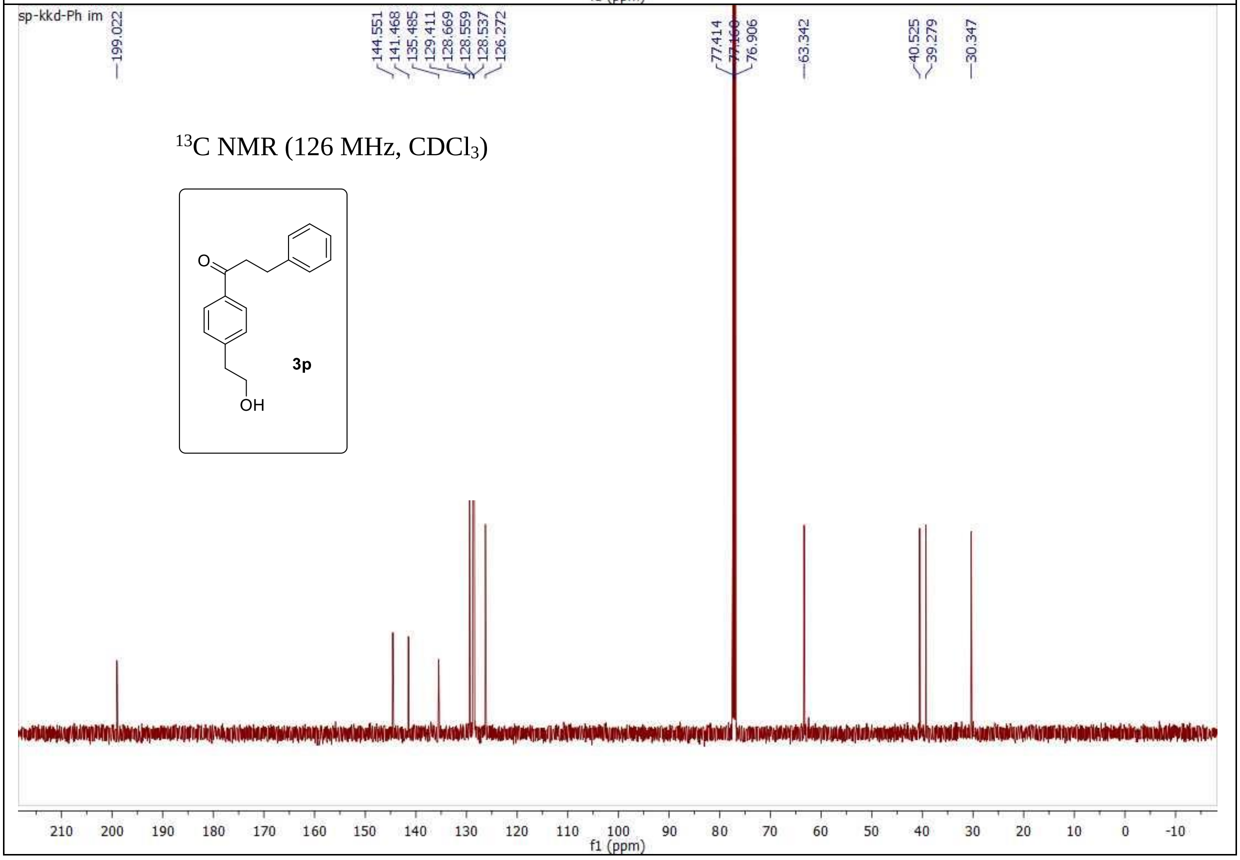
sp-kkd-Ph im
199.022
144.551
141.468
135.485
129.411
128.669
128.559
128.537
126.272
77.414
76.906
63.342
40.525
39.279
30.347
^{13}C NMR (126 MHz, $CDCl_3$)
O
OH
3p
f1 (ppm)

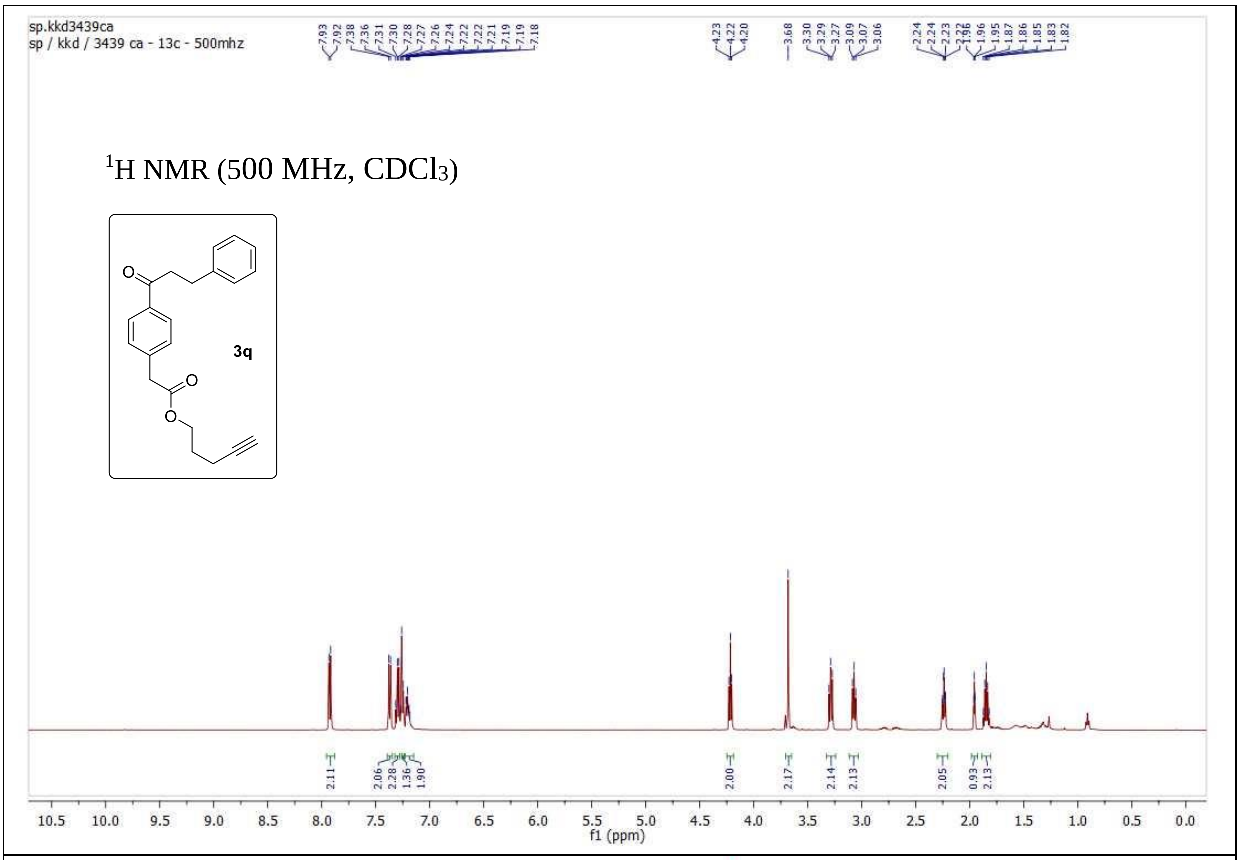
sp.kkd3439ca
sp / kkd / 3439 ca - 13c - 500mhz
1H NMR (500 MHz, CDCl3)
3q
f1 (ppm)

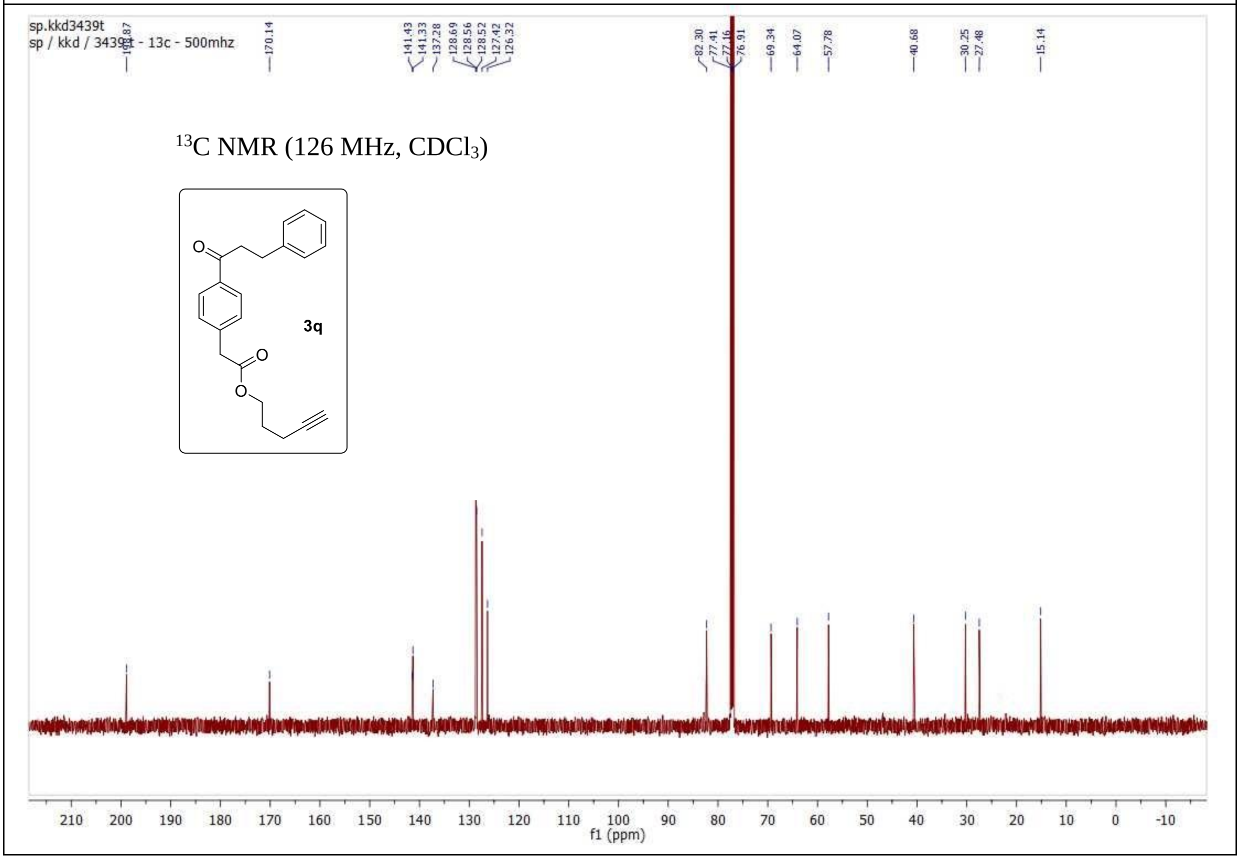
sp.kkd3439t
13C NMR (126 MHz, CDCl3)
3q
f1 (ppm)

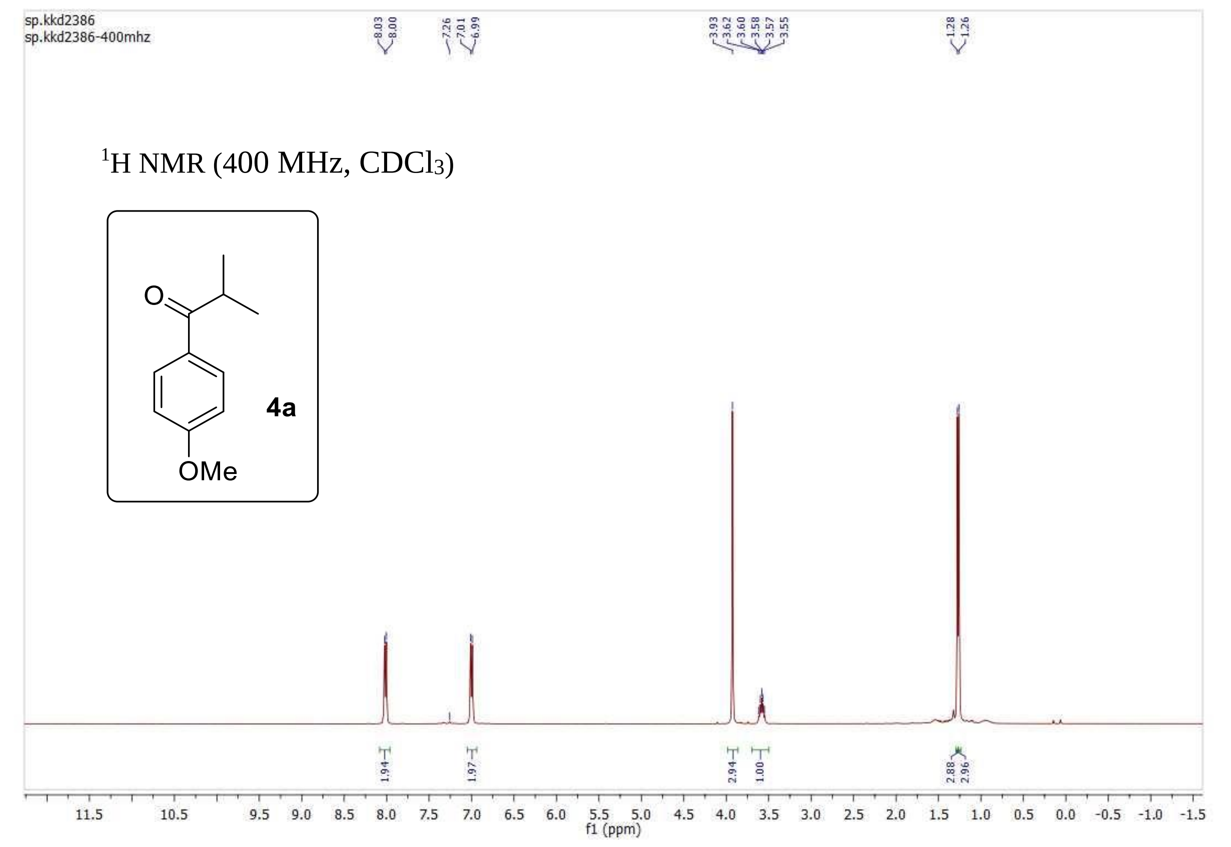
sp.kkd2386
sp.kkd2386-400mhz
1H NMR (400 MHz, CDCl3)
O
4a
OMe
f1 (ppm)

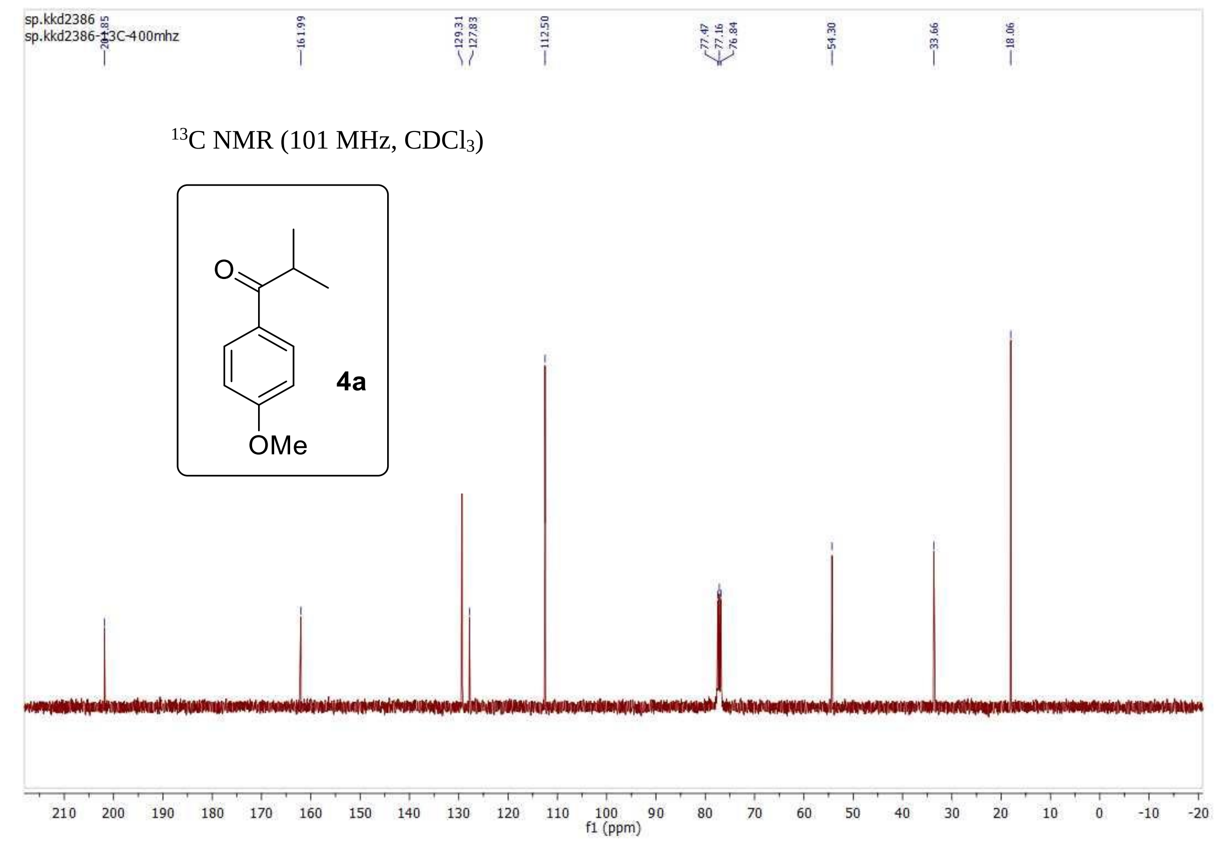
sp.kkd2386
sp.kkd2386-13C-400mhz
13C NMR (101 MHz, CDCl3)
O
4a
OMe
f1 (ppm)

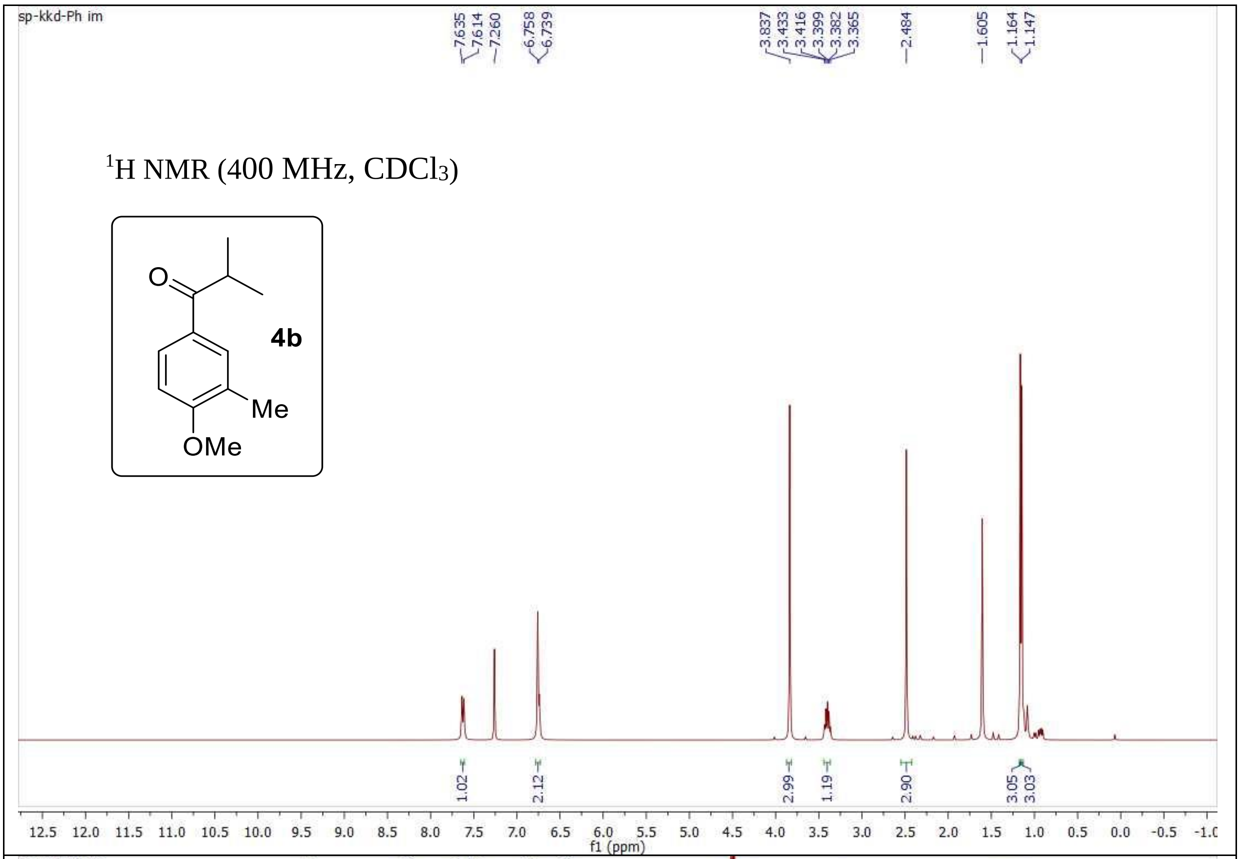
sp-kkd-Ph im
7.635
7.614
7.260
6.758
6.739
3.837
3.433
3.416
3.399
3.382
3.365
2.484
1.605
1.164
1.147
1H NMR (400 MHz, CDCl3)
O
4b
Me
OMe
1.02
2.12
2.99
1.19
2.90
3.05
3.03
12.5 12.0 11.5 11.0 10.5 10.0 9.5 9.0 8.5 8.0 7.5 7.0 6.5 6.0 5.5 5.0 4.5 4.0 3.5 3.0 2.5 2.0 1.5 1.0 0.5 0.0 -0.5
f1 (ppm)

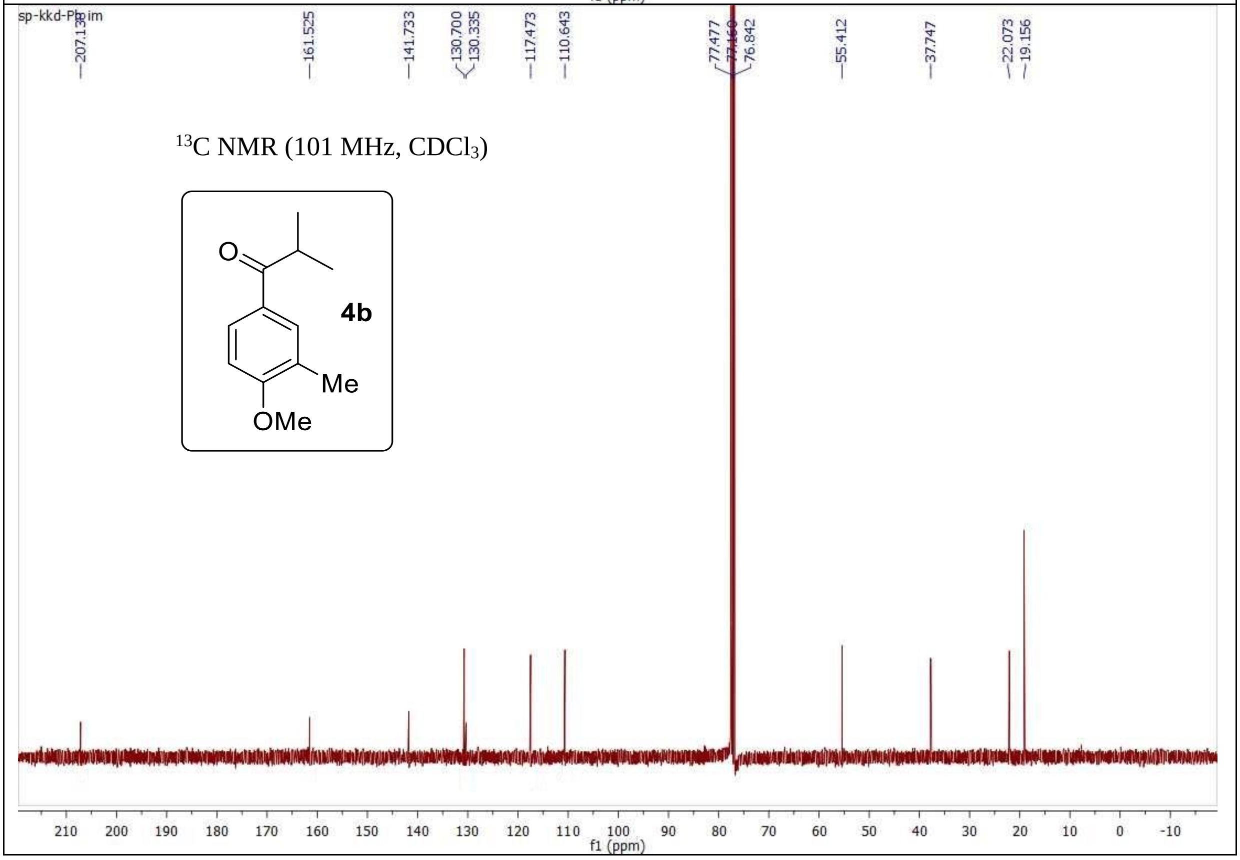
sp-kkd-Ph im
207.138
161.525
141.733
130.700
130.335
117.473
110.643
77.477
77.160
76.842
55.412
37.747
22.073
19.156
13C NMR (101 MHz, CDCl3)
O
4b
Me
OMe
210 200 190 180 170 160 150 140 130 120 110 100 90 80 70 60 50 40 30 20 10 0 -10
f1 (ppm)

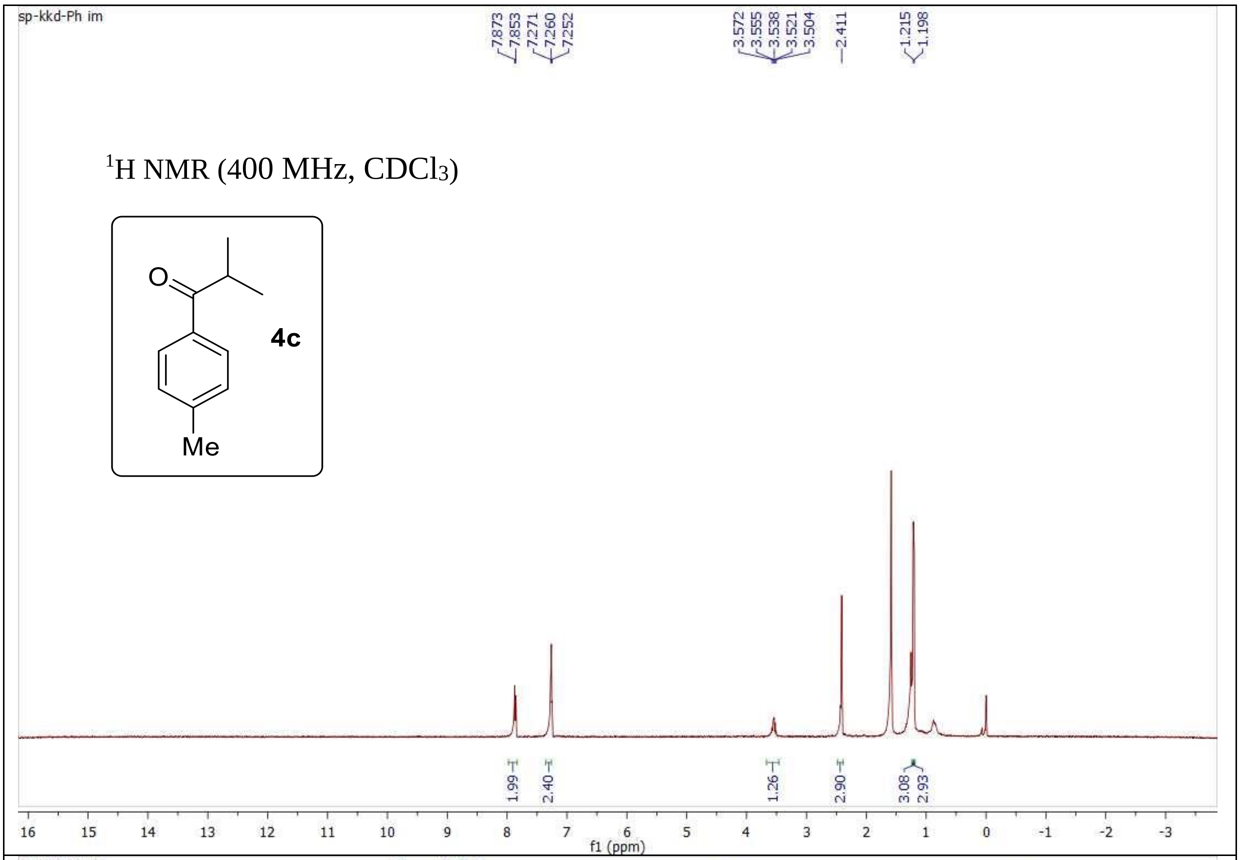

sp-kkd-Ph im
7.873
7.853
7.271
7.260
7.252
3.572
3.555
3.538
3.521
3.504
2.411
1.215
1.198
^{1}H NMR (400 MHz, $CDCl_3$)
O
4c
Me
1.99
2.40
1.26
2.90
3.08
2.93
f1 (ppm)

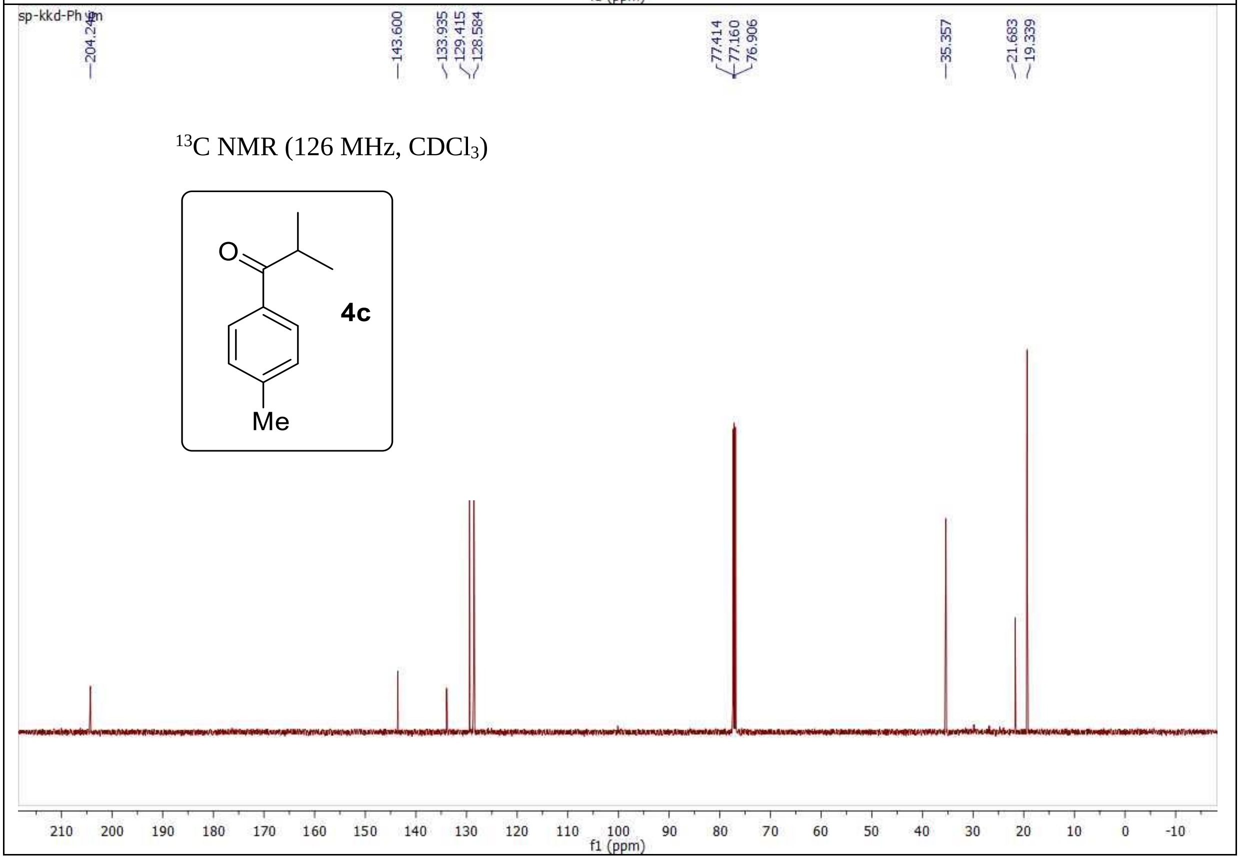

sp-kkd-Ph im
204.249
143.600
133.935
129.415
128.584
77.414
77.160
76.906
35.357
21.683
19.339
^{13}C NMR (126 MHz, $CDCl_3$)
O
4c
Me
f1 (ppm)

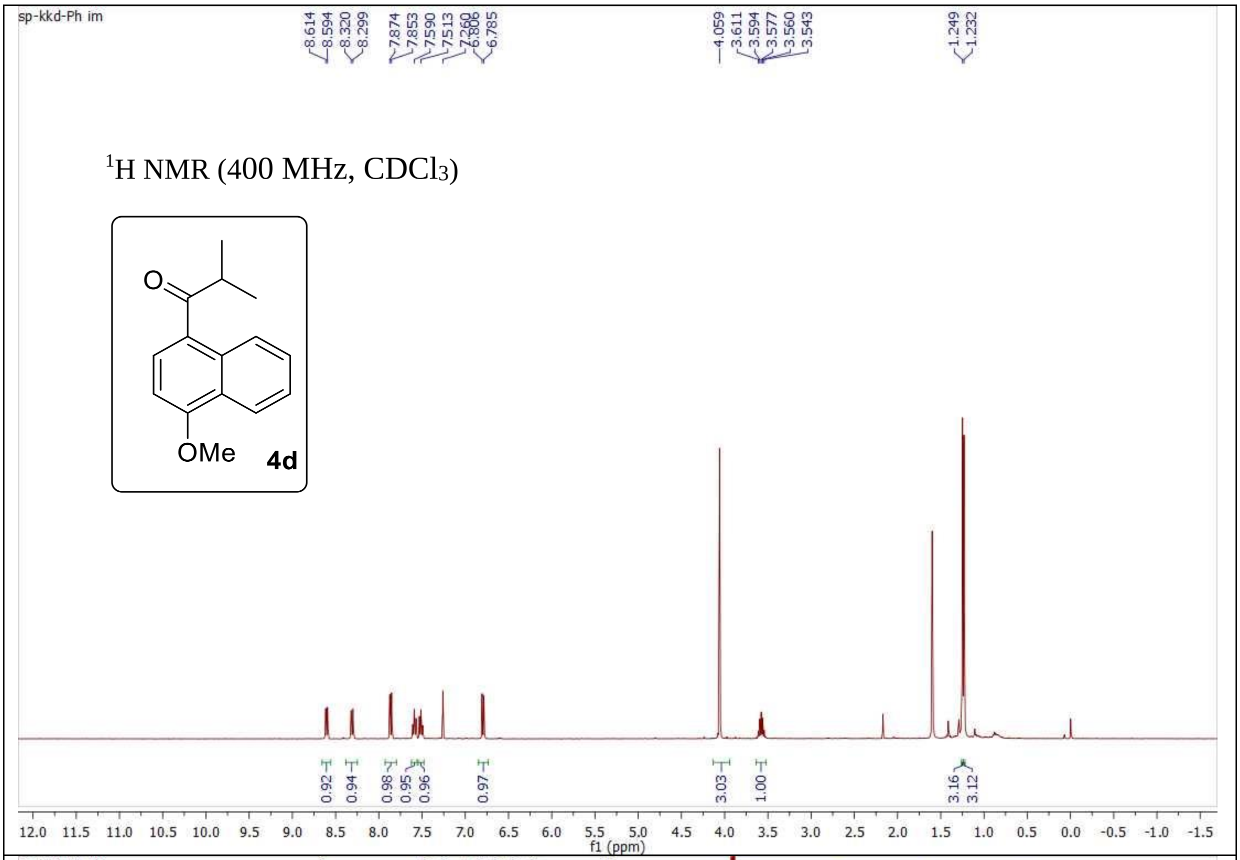
sp-kkd-Ph im
8.614
8.594
8.320
8.299
7.874
7.853
7.590
7.513
7.260
6.806
6.785
4.059
3.611
3.594
3.577
3.560
3.543
1.249
1.232
¹H NMR (400 MHz, CDCl₃)
O
OMe
4d
0.92
0.94
0.98
0.95
0.96
0.97
3.03
1.00
3.16
3.12
f1 (ppm)

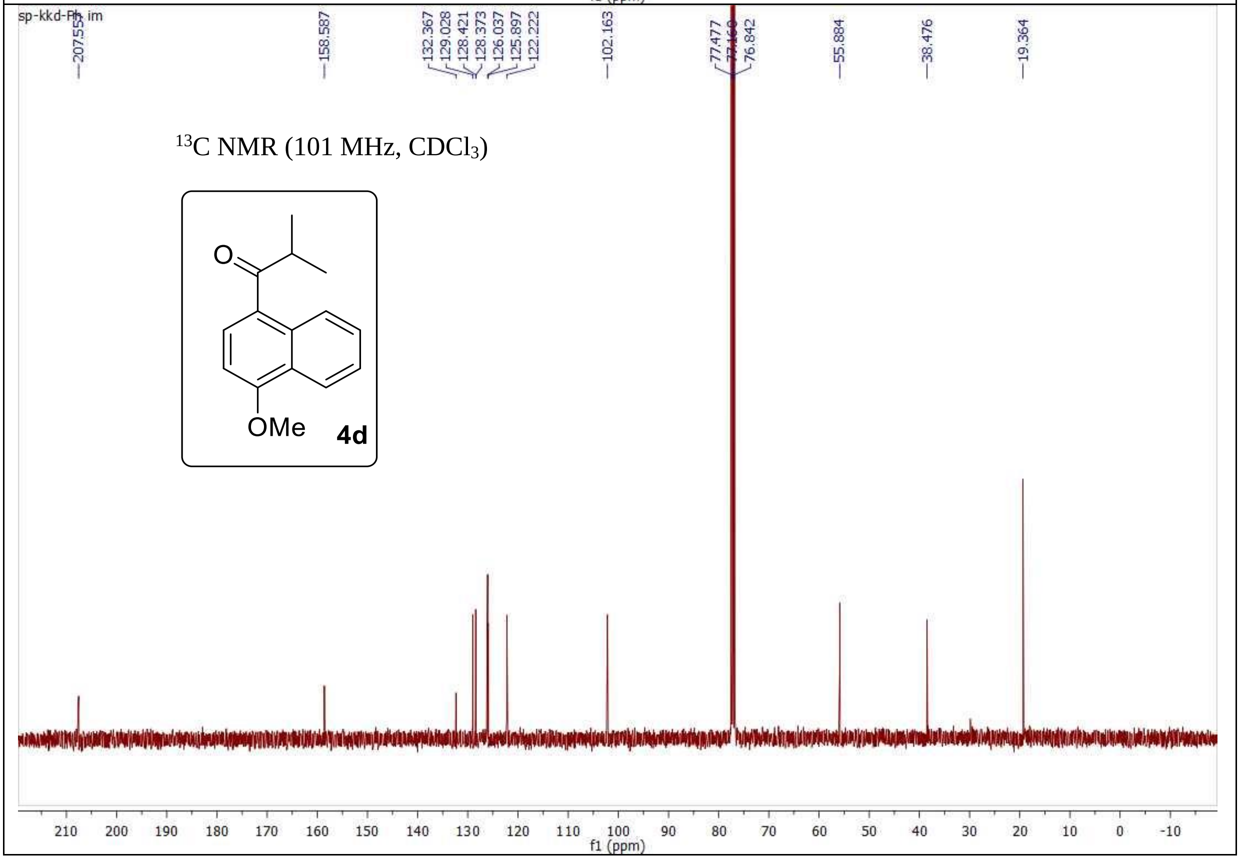
sp-kkd-Ph im
207.553
158.587
132.367
129.028
128.421
128.373
126.037
125.897
122.222
102.163
77.477
76.842
55.884
38.476
19.364
¹³C NMR (101 MHz, CDCl₃)
O
OMe
4d
f1 (ppm)

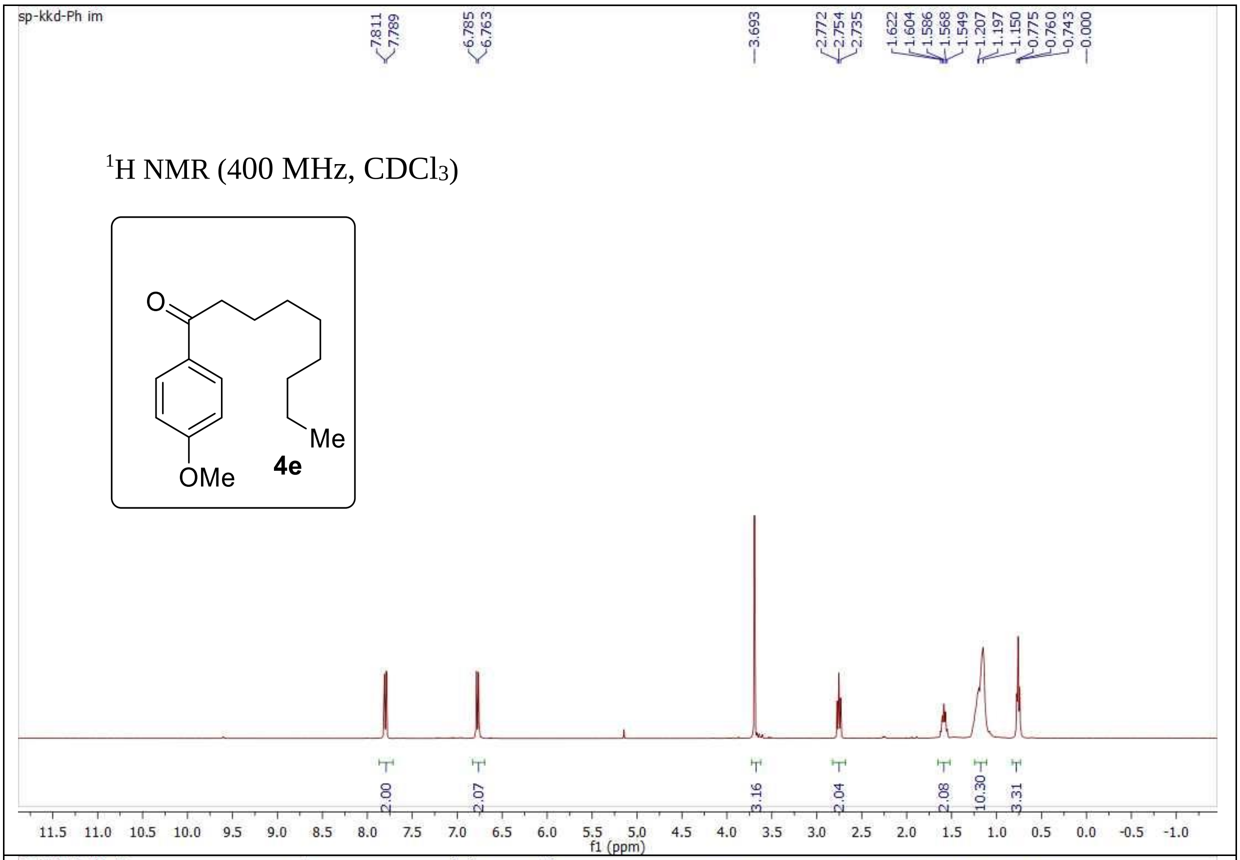
sp-kkd-Ph im
7.811
7.789
6.785
6.763
3.693
2.772
2.754
2.735
1.622
1.604
1.586
1.568
1.549
1.207
1.197
1.150
0.775
0.760
0.743
0.000
1H NMR (400 MHz, CDCl3)
O
Me
OMe
4e
2.00
2.07
3.16
2.04
2.08
10.30
3.31
f1 (ppm)

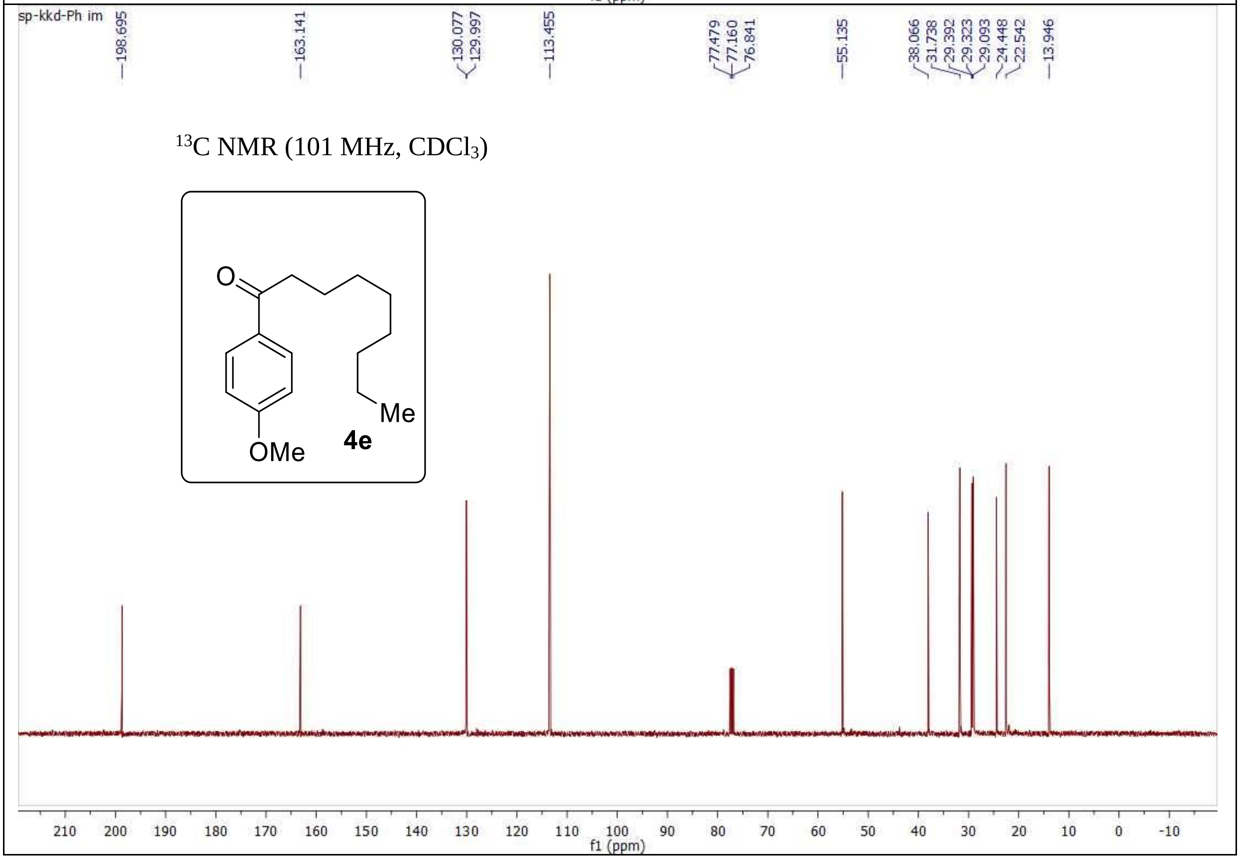
sp-kkd-Ph im
198.695
163.141
130.077
129.997
113.455
77.479
77.160
76.841
55.135
38.066
31.738
29.392
29.323
29.093
24.448
22.542
13.946
13C NMR (101 MHz, CDCl3)
O
Me
OMe
4e
f1 (ppm)

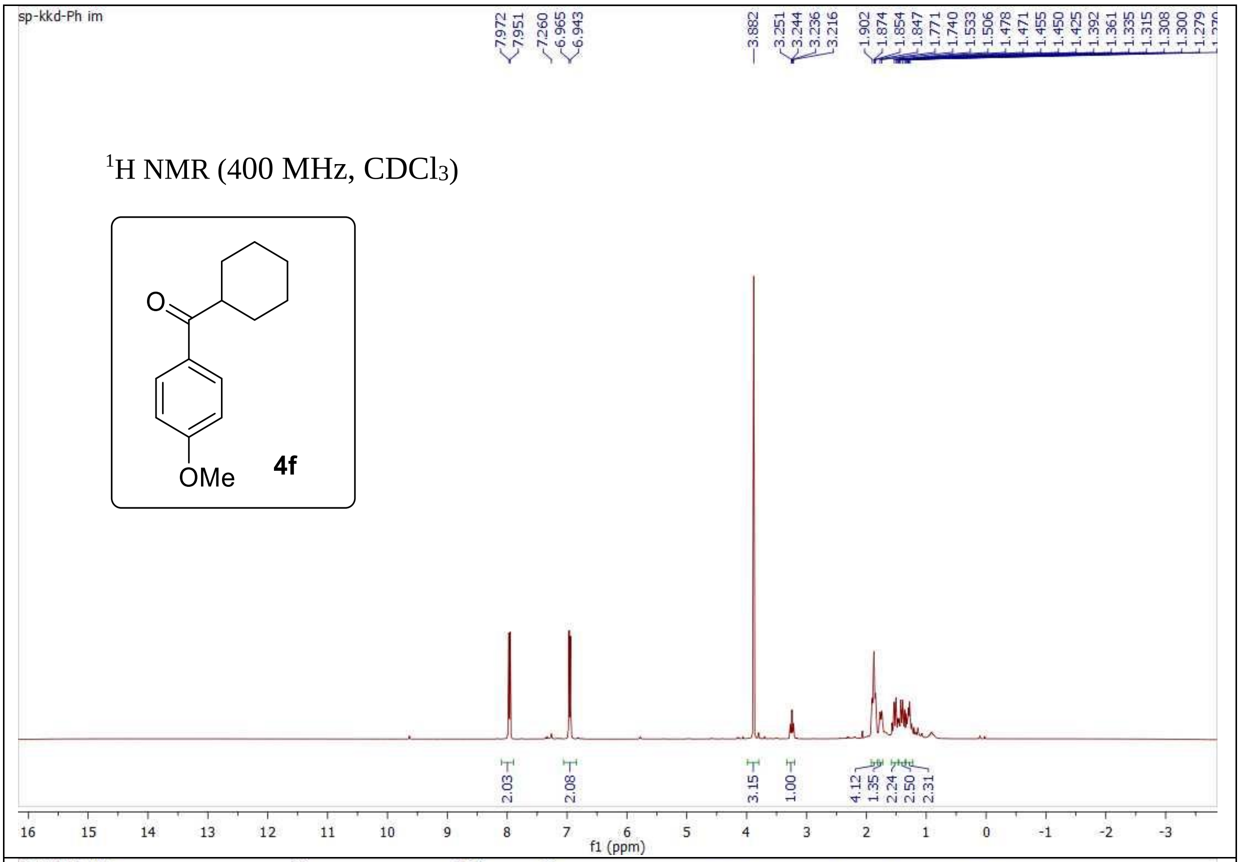
sp-kkd-Ph im
7.972
7.951
7.260
6.965
6.943
3.882
3.251
3.244
3.236
3.216
1.902
1.874
1.854
1.847
1.771
1.740
1.533
1.506
1.478
1.471
1.455
1.450
1.425
1.392
1.361
1.335
1.315
1.308
1.300
1.279
1H NMR (400 MHz, CDCl3)
O
OMe
4f
2.03
2.08
3.15
1.00
4.12
1.35
2.24
2.50
2.31
f1 (ppm)

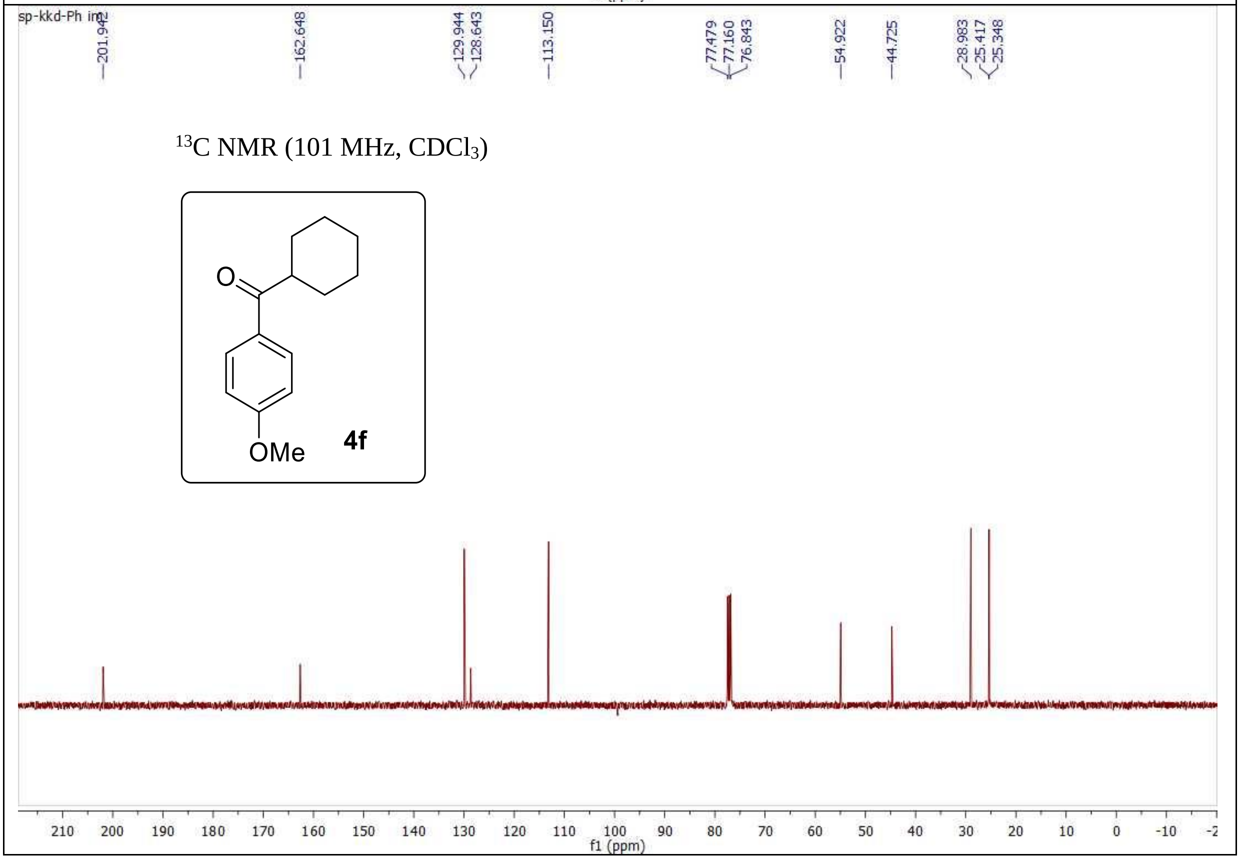
sp-kkd-Ph im
201.942
162.648
129.944
128.643
113.150
77.479
77.160
76.843
54.922
44.725
28.983
25.417
25.348
13C NMR (101 MHz, CDCl3)
O
OMe
4f
f1 (ppm)

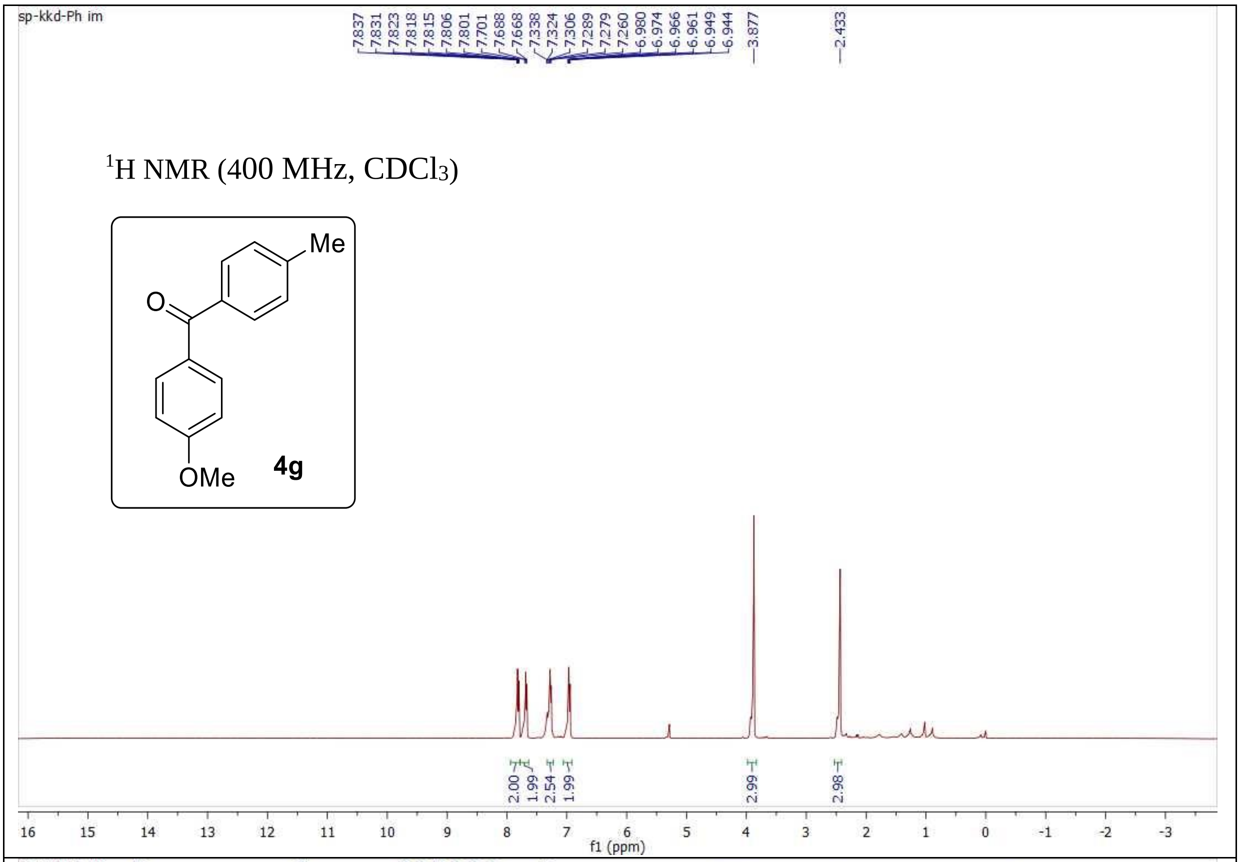

sp-kkd-Ph im
7.837
7.831
7.823
7.818
7.815
7.806
7.801
7.701
7.688
7.668
7.338
7.324
7.306
7.289
7.279
7.260
6.980
6.974
6.966
6.961
6.949
6.944
3.877
2.433
¹H NMR (400 MHz, CDCl₃)
Me
O
OMe
4g
2.00
1.99
2.54
1.99
2.99
2.98
16 15 14 13 12 11 10 9 8 7 6 5 4 3 2 1 0 -1 -2 -3
f1 (ppm)

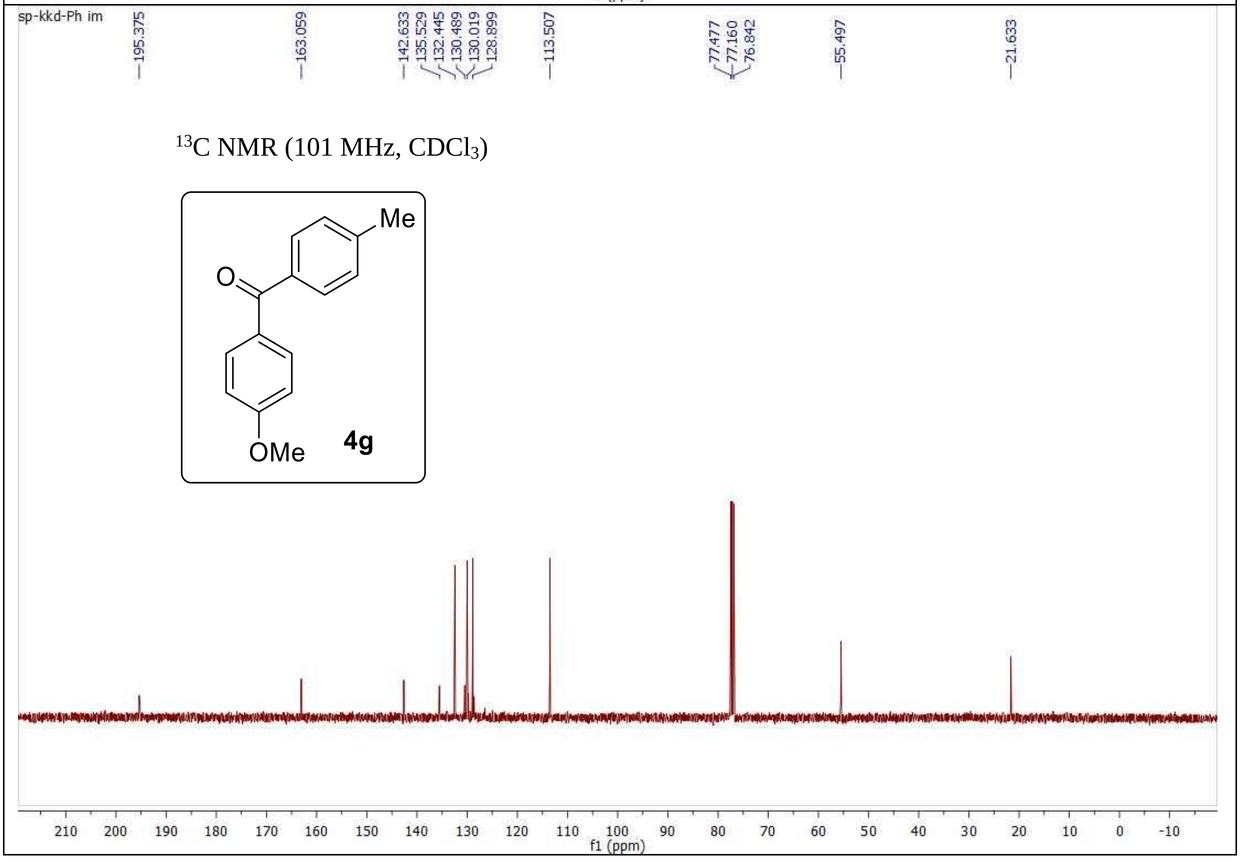

sp-kkd-Ph im
195.375
163.059
142.633
135.529
132.445
130.489
130.019
128.899
113.507
77.477
77.160
76.842
55.497
21.633
¹³C NMR (101 MHz, CDCl₃)
Me
O
OMe
4g
210 200 190 180 170 160 150 140 130 120 110 100 90 80 70 60 50 40 30 20 10 0 -10
f1 (ppm)

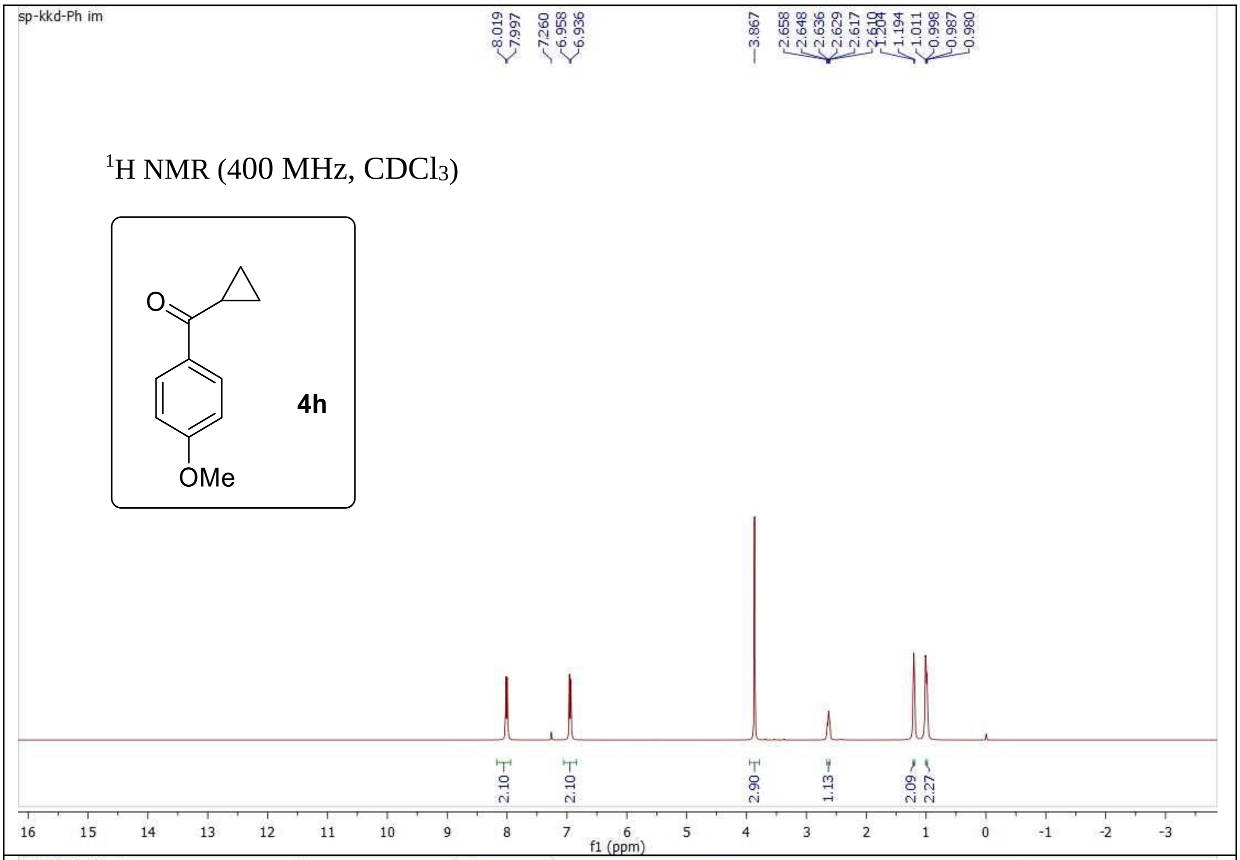
sp-kkd-Ph im
8.019
7.997
7.260
6.958
6.936
3.867
2.658
2.648
2.636
2.629
2.617
2.610
1.204
1.194
1.011
0.998
0.987
0.980
¹H NMR (400 MHz, CDCl3)
O
OMe
4h
2.10
2.10
2.90
1.13
2.09
2.27
16
15
14
13
12
11
10
9
8
7
6
5
4
3
2
1
0
-1
-2
-3
f1 (ppm)

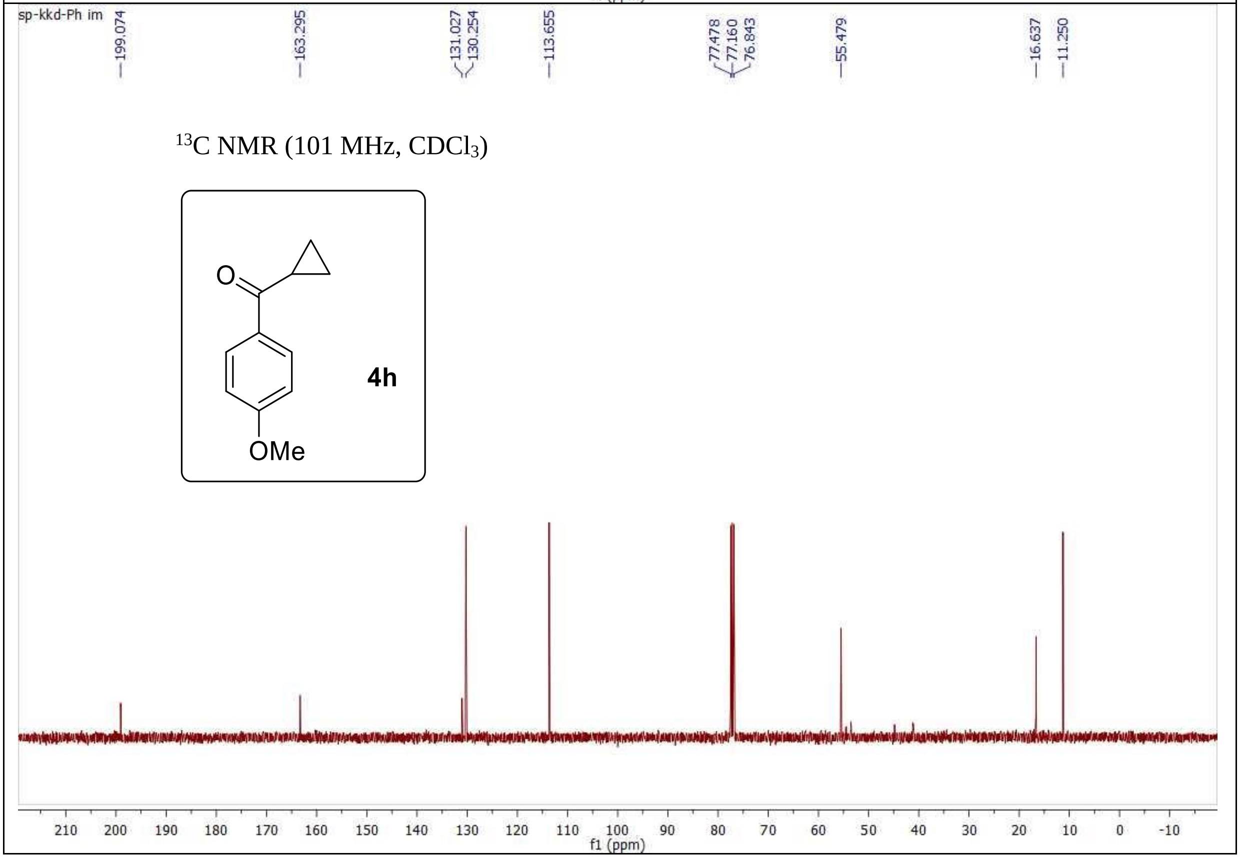
sp-kkd-Ph im
199.074
163.295
131.027
130.254
113.655
77.478
77.160
76.843
55.479
16.637
11.250
¹³C NMR (101 MHz, CDCl3)
O
OMe
4h
210
200
190
180
170
160
150
140
130
120
110
100
90
80
70
60
50
40
30
20
10
0
-10
f1 (ppm)

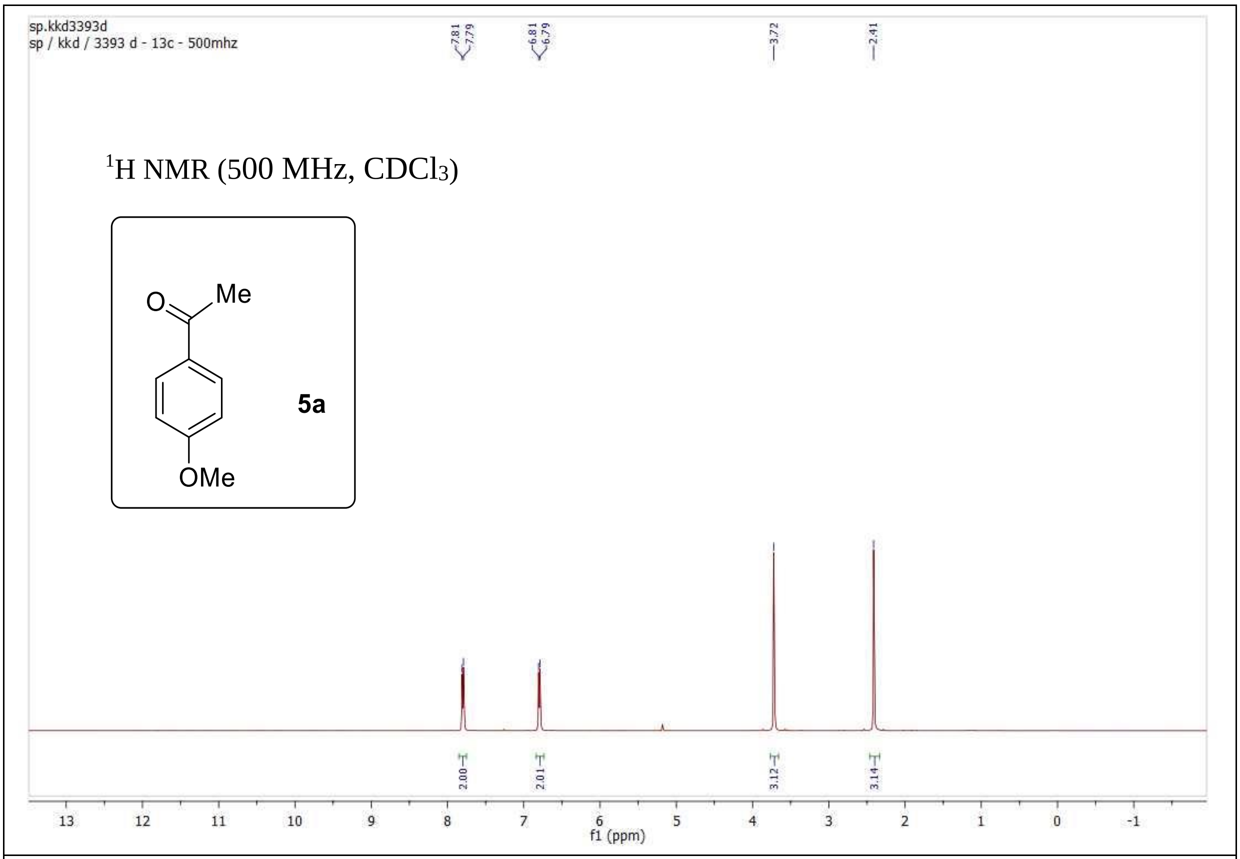
sp.kkd3393d
sp / kkd / 3393 d - 13c - 500mhz
7.81
7.79
6.81
6.79
3.72
2.41
1H NMR (500 MHz, CDCl3)
O
Me
5a
OMe
2.00
2.01
3.12
3.14
f1 (ppm)

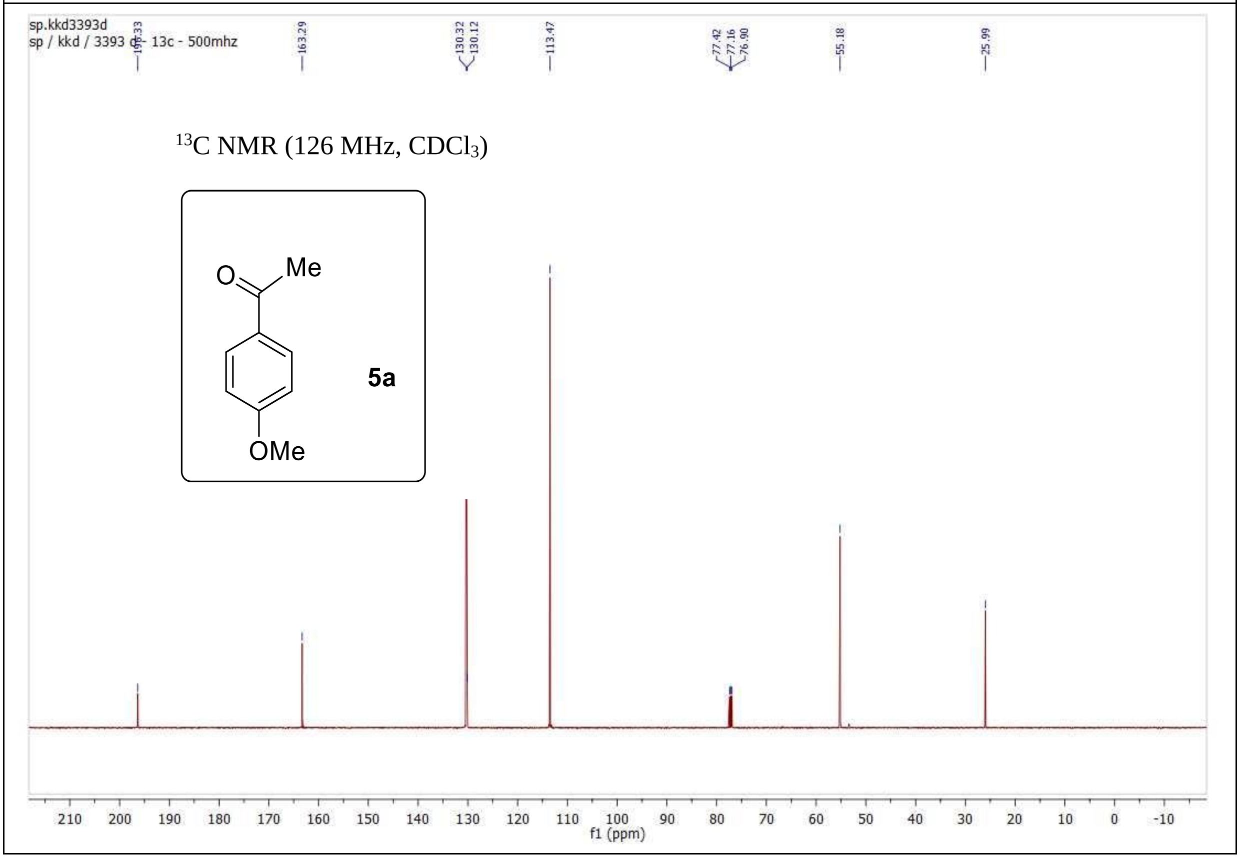
sp.kkd3393d
sp / kkd / 3393 d - 13c - 500mhz
163.29
130.32
130.12
113.47
77.42
77.16
76.90
55.18
25.99
13C NMR (126 MHz, CDCl3)
O
Me
5a
OMe
f1 (ppm)

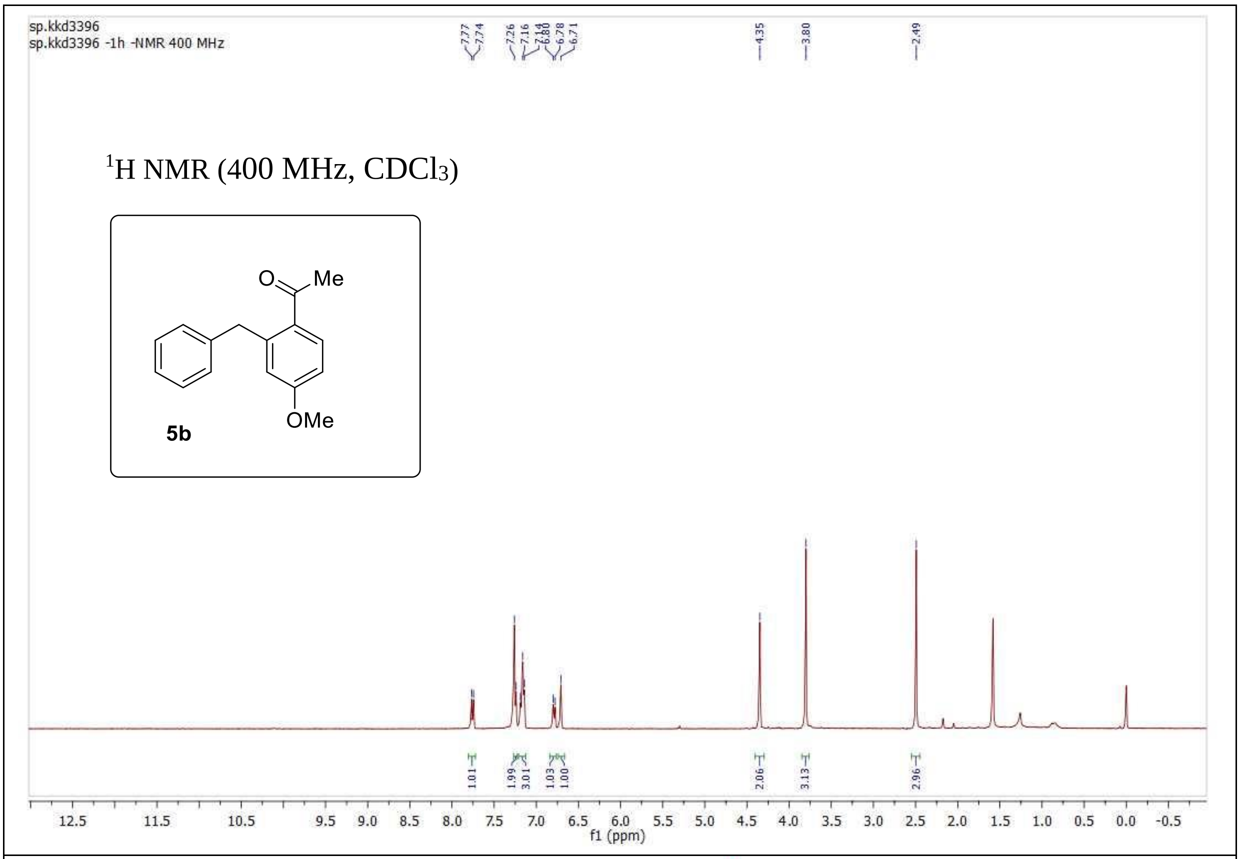
sp.kkd3396
sp.kkd3396 -1h -NMR 400 MHz
7.77
7.74
7.26
7.16
7.14
6.80
6.78
6.71
4.35
3.80
2.49
^{1}H NMR (400 MHz, CDCl$_3$)
O
Me
OMe
5b
1.01
1.99
3.01
1.03
1.00
2.06
3.13
2.96
f1 (ppm)

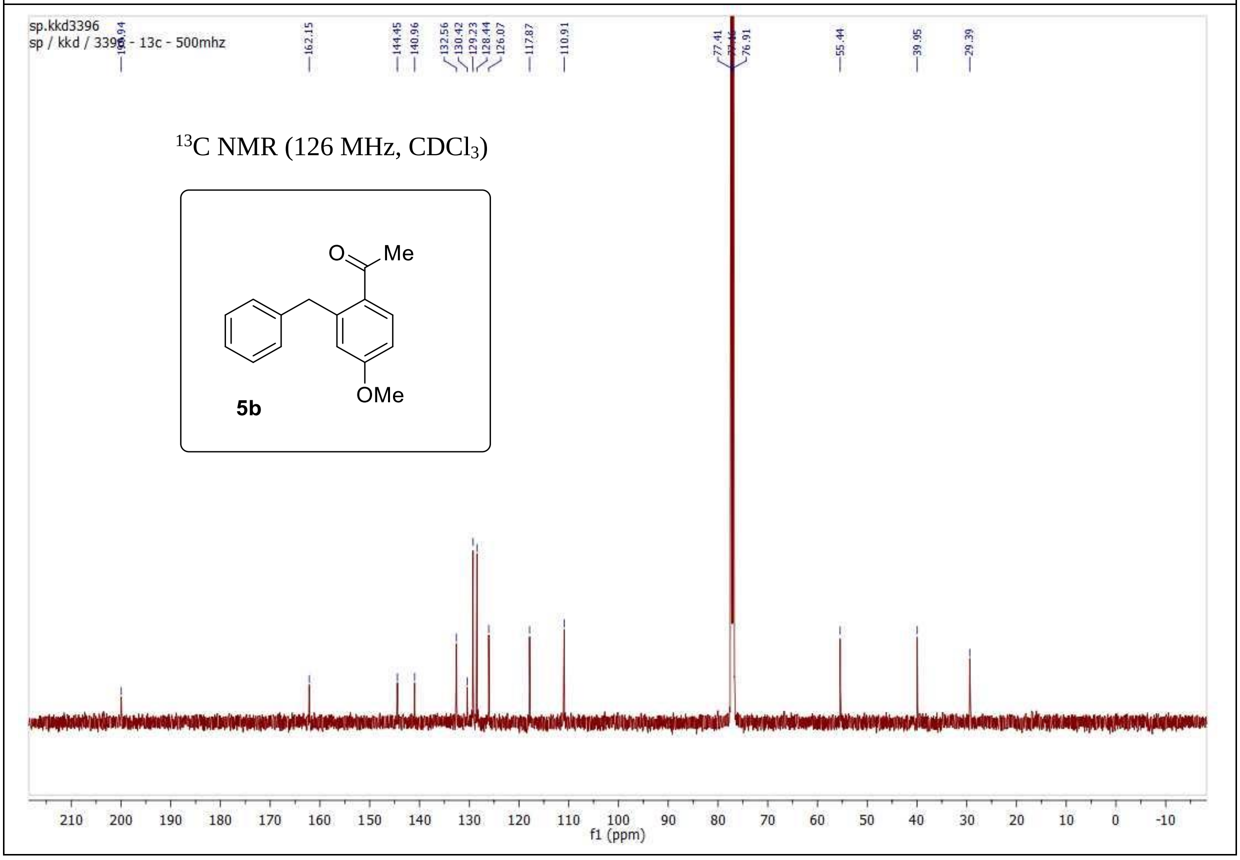
sp.kkd3396
sp / kkd / 3396 - 13c - 500mhz
199.94
162.15
144.45
140.96
132.56
130.42
129.23
128.44
126.07
117.87
110.91
77.41
76.91
55.44
39.95
29.39
^{13}C NMR (126 MHz, CDCl$_3$)
O
Me
OMe
5b
f1 (ppm)

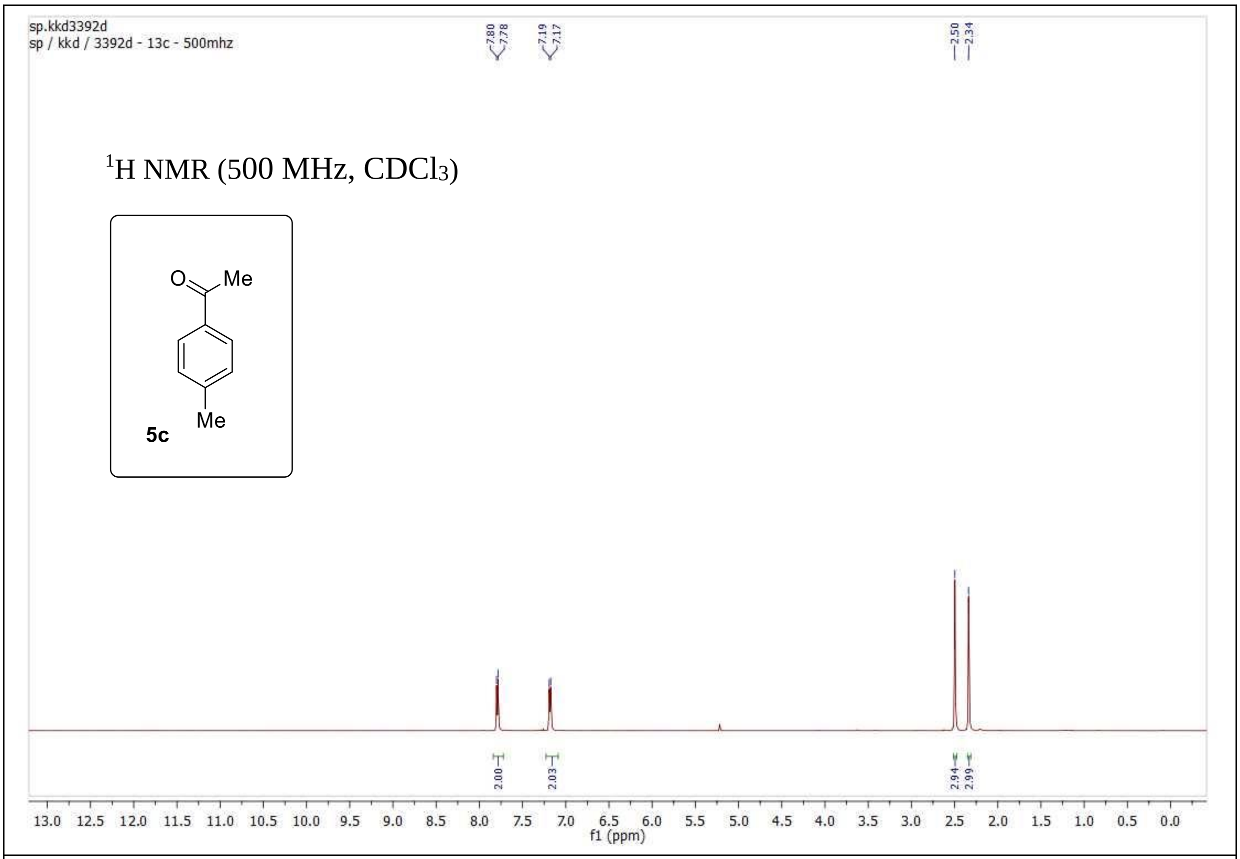
sp.kkd3392d
sp / kkd / 3392d - 13c - 500mhz
7.80
7.78
7.19
7.17
2.50
2.34
^{1}H NMR (500 MHz, $CDCl_3$)
O
Me
Me
5c
2.00
2.03
2.94
2.99
13.0 12.5 12.0 11.5 11.0 10.5 10.0 9.5 9.0 8.5 8.0 7.5 7.0 6.5 6.0 5.5 5.0 4.5 4.0 3.5 3.0 2.5 2.0 1.5 1.0 0.5 0.0
f1 (ppm)

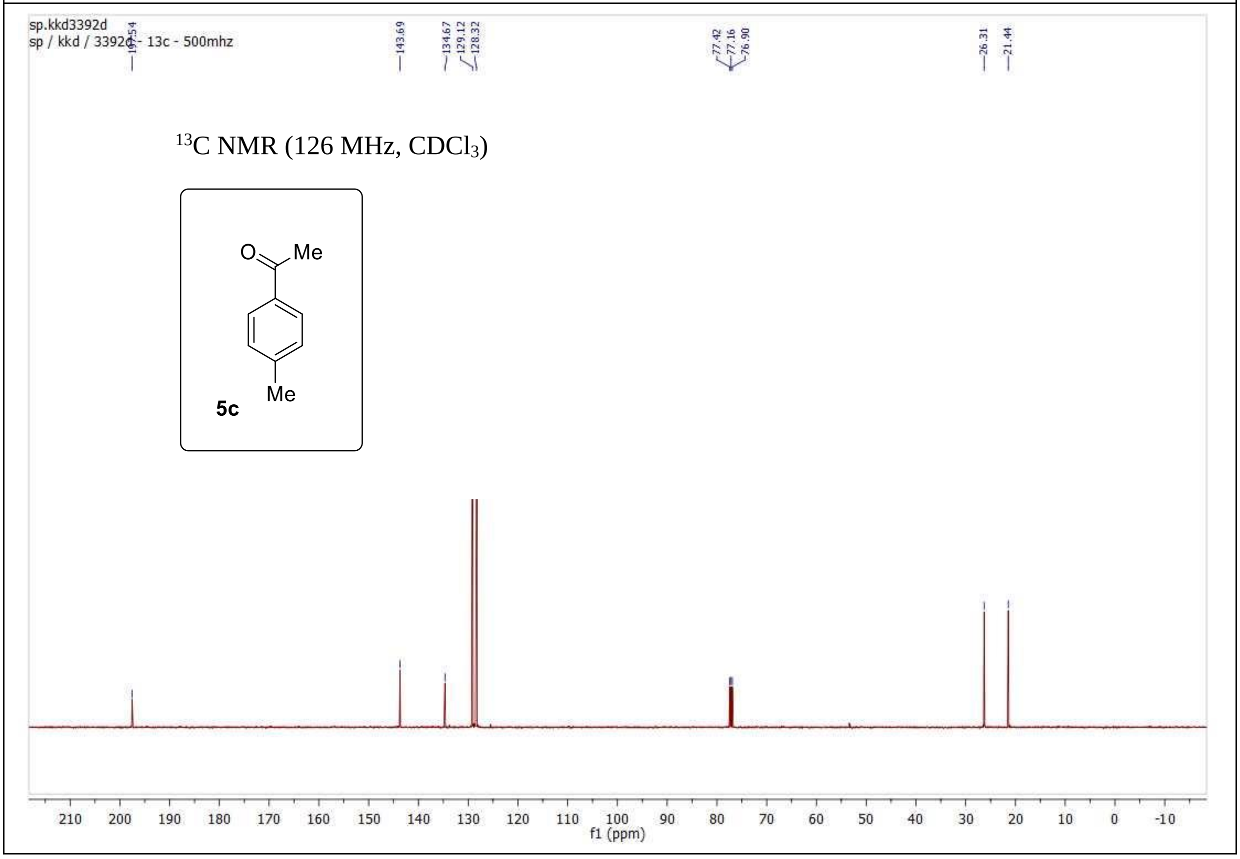
sp.kkd3392d
sp / kkd / 3392d - 13c - 500mhz
197.54
143.69
134.67
129.12
128.32
77.42
77.16
76.90
26.31
21.44
^{13}C NMR (126 MHz, $CDCl_3$)
O
Me
Me
5c
210 200 190 180 170 160 150 140 130 120 110 100 90 80 70 60 50 40 30 20 10 0 -10
f1 (ppm)

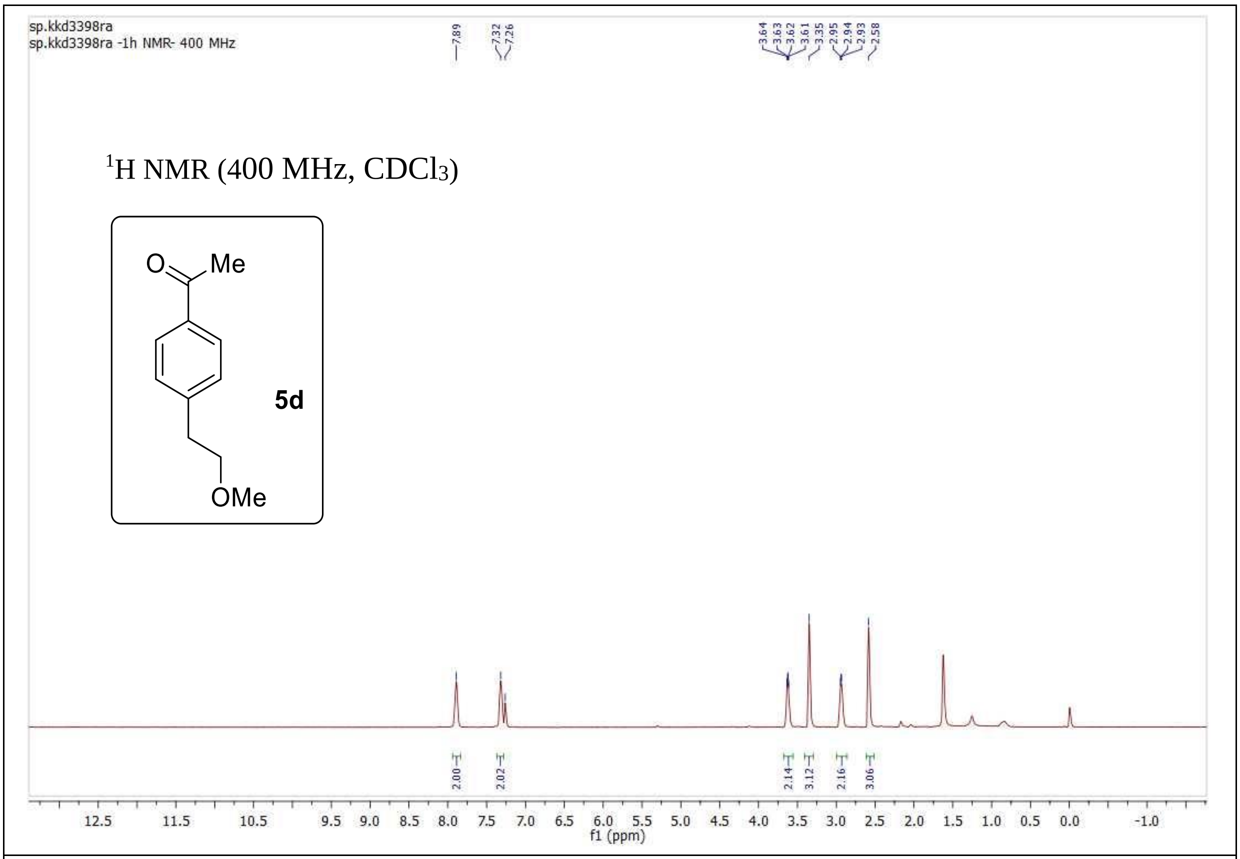
sp.kkd3398ra
sp.kkd3398ra -1h NMR- 400 MHz
1H NMR (400 MHz, CDCl3)
O
Me
5d
OMe
f1 (ppm)

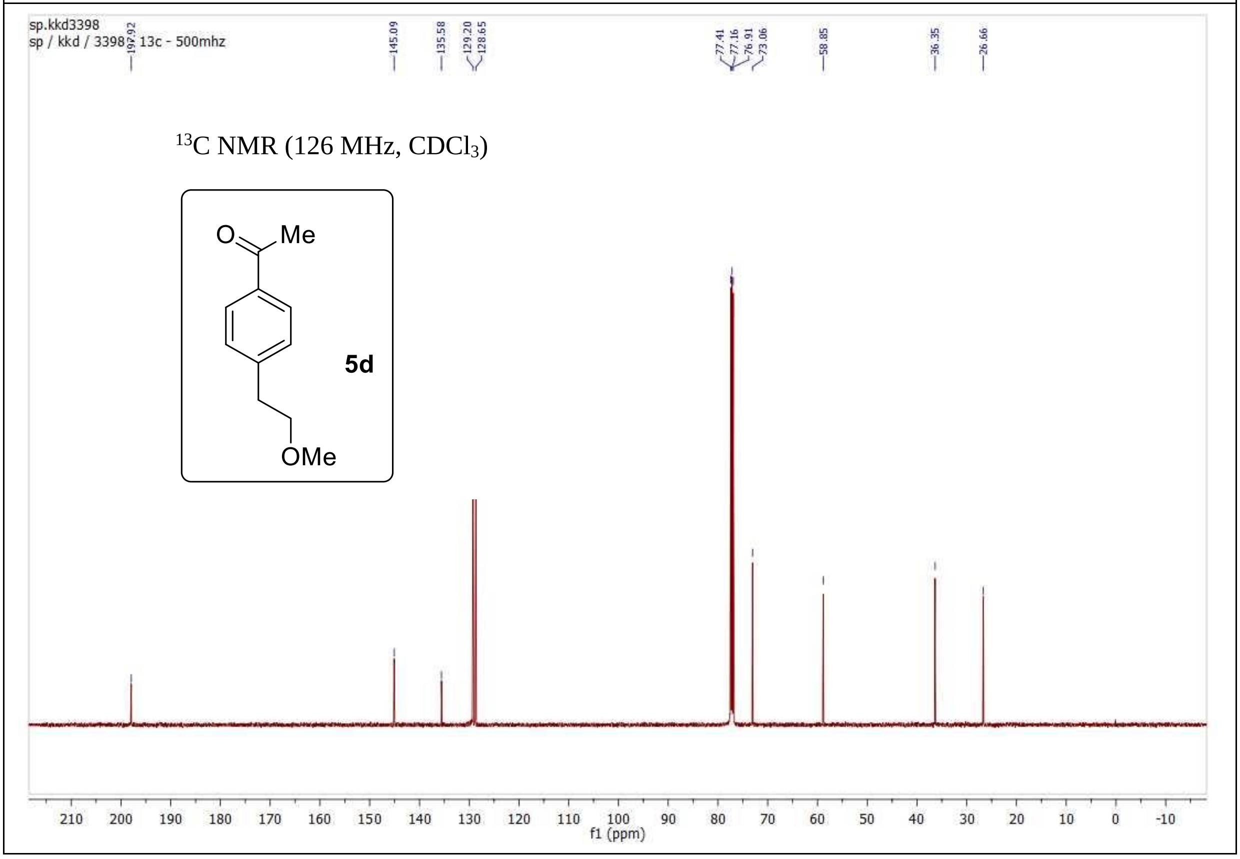
sp.kkd3398
sp / kkd / 3398 13c - 500mhz
13C NMR (126 MHz, CDCl3)
O
Me
5d
OMe
f1 (ppm)

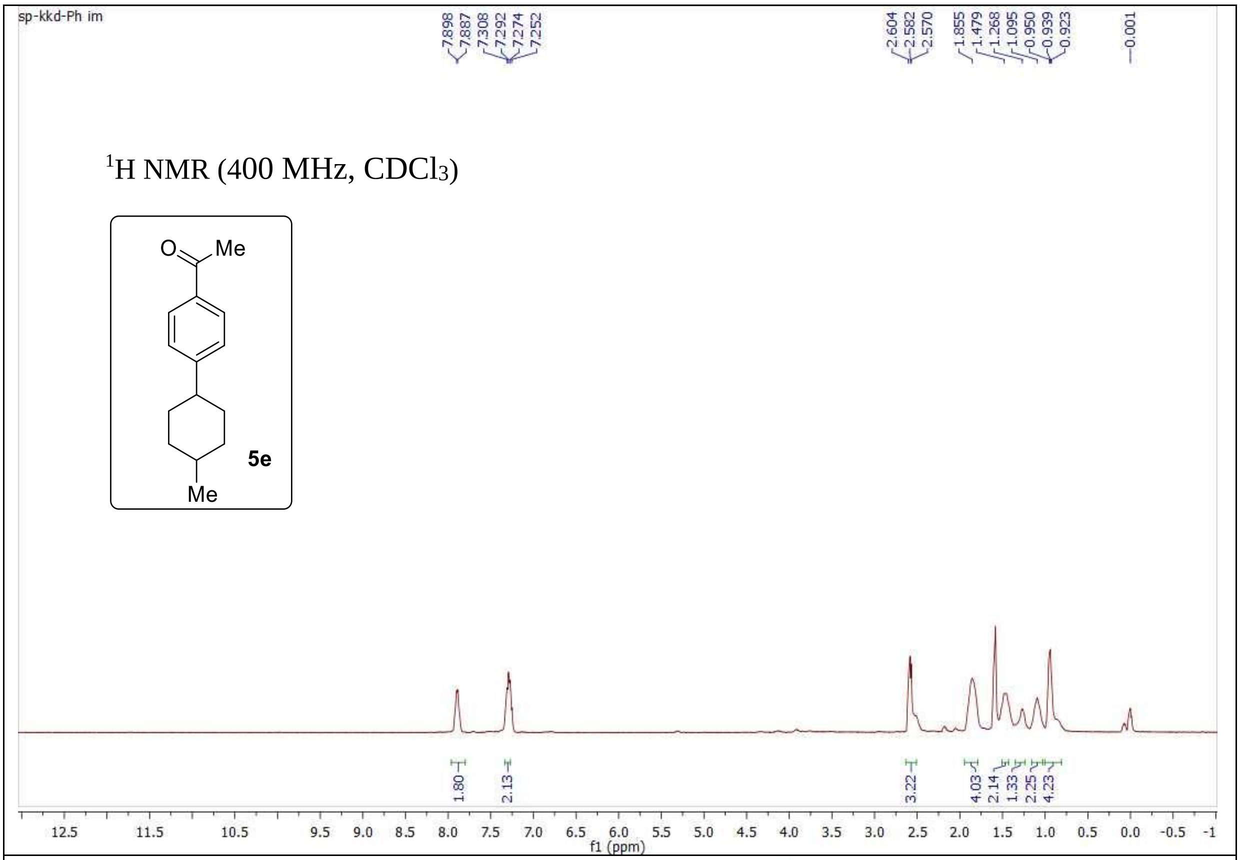
sp-kkd-Ph im
7.898
7.887
7.308
7.292
7.274
7.252
2.604
2.582
2.570
1.855
1.479
1.268
1.095
0.950
0.939
0.923
-0.001
1H NMR (400 MHz, CDCl3)
O
Me
Me
5e
1.80
2.13
3.22
4.03
2.14
1.33
2.25
4.23
f1 (ppm)

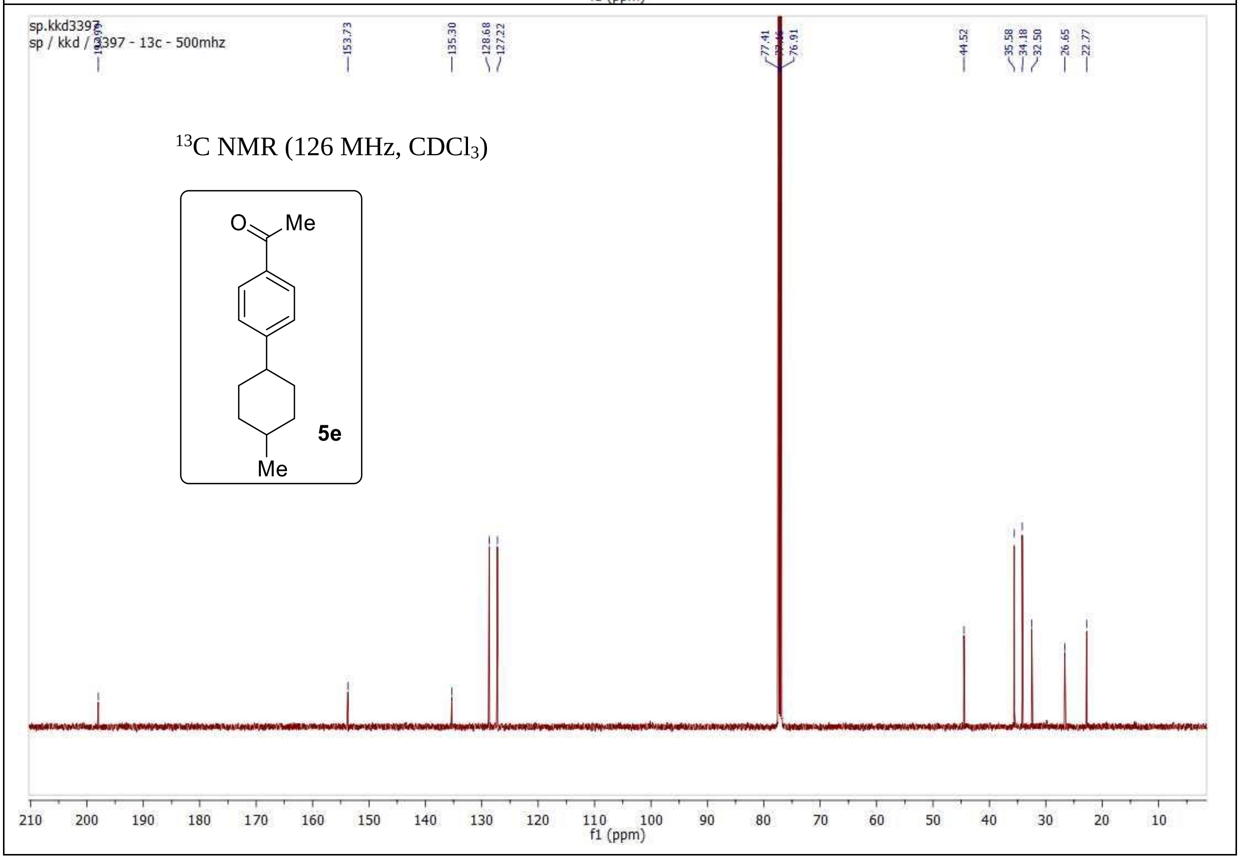
sp.kkd3397
sp / kkd / 3397 - 13c - 500mhz
153.73
135.30
128.68
127.22
77.41
76.91
44.52
35.58
34.18
32.50
26.65
22.77
13C NMR (126 MHz, CDCl3)
O
Me
Me
5e
f1 (ppm)

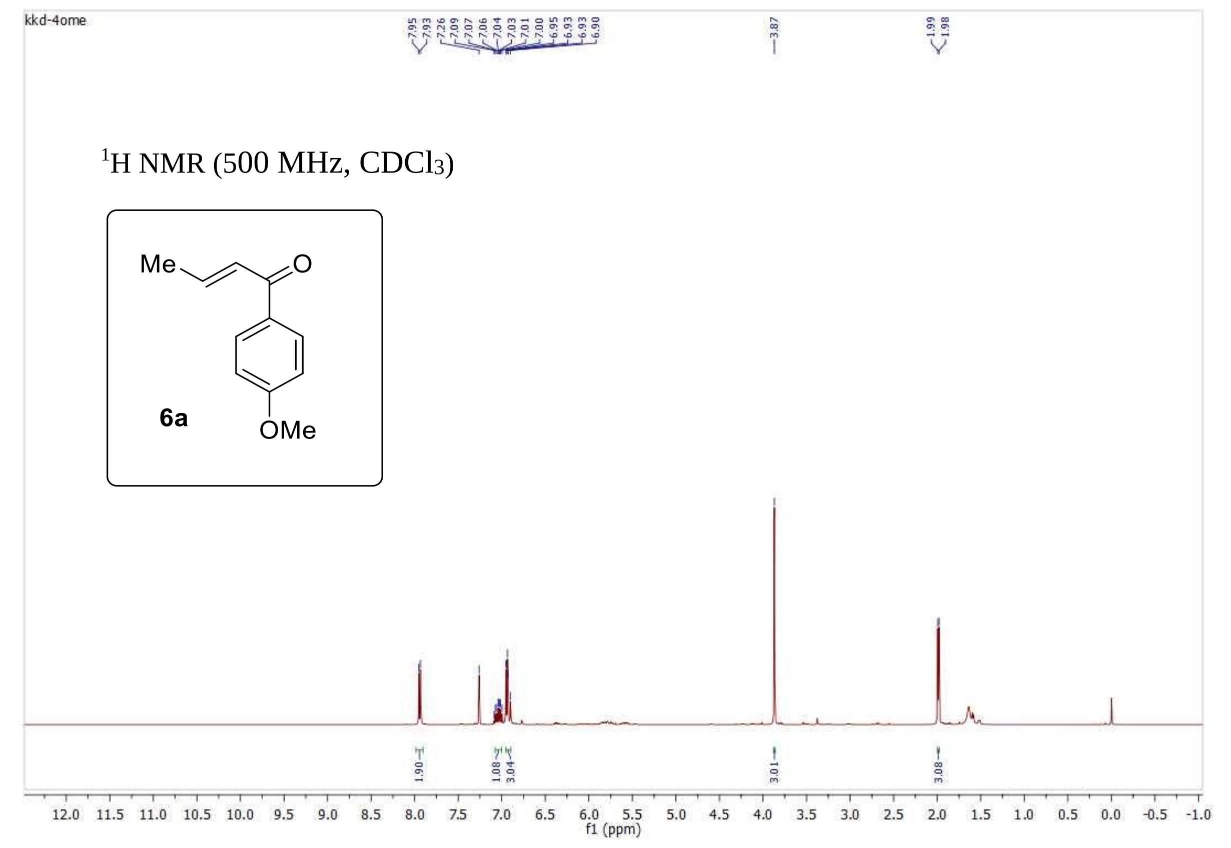
kkd-4ome
1H NMR (500 MHz, CDCl3)
Me
O
6a
OMe
f1 (ppm)

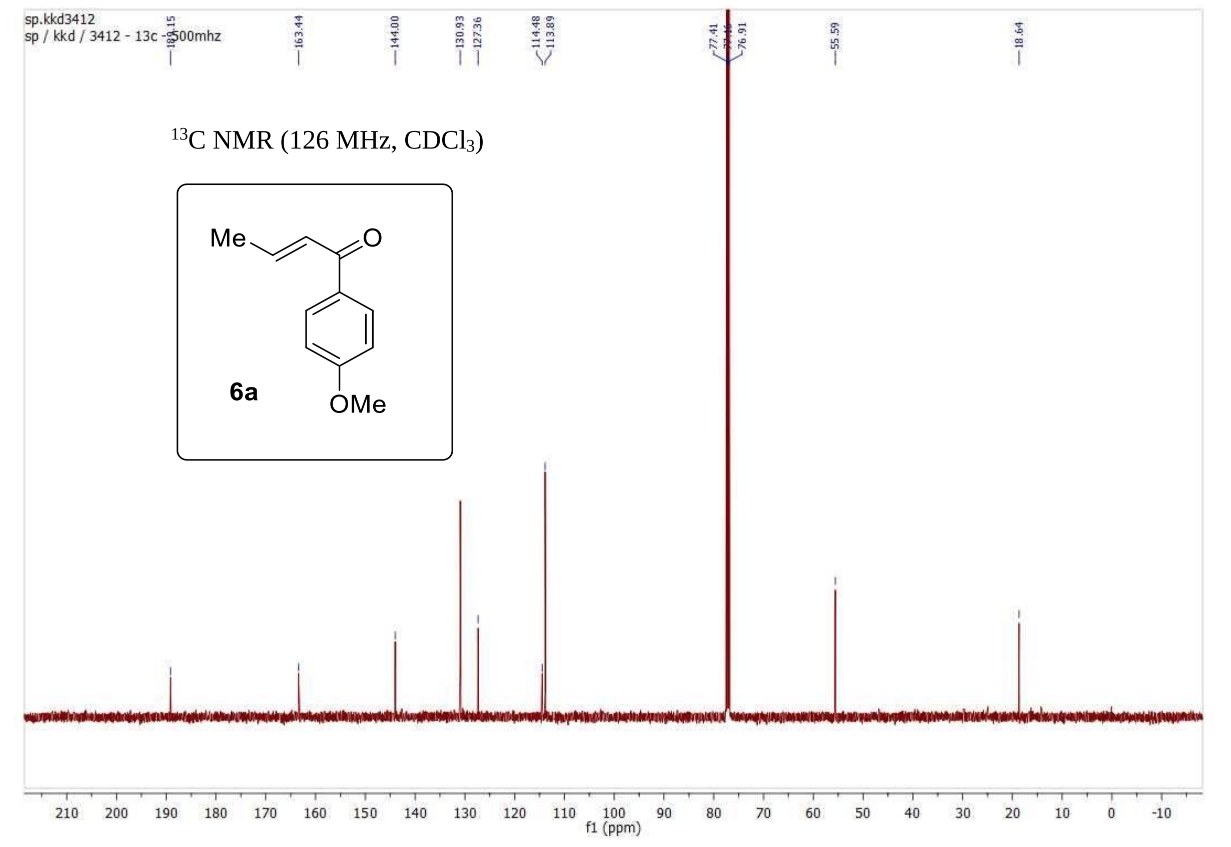
sp.kkd3412
sp / kkd / 3412 - 13c - 500mhz
13C NMR (126 MHz, CDCl3)
Me
O
6a
OMe
f1 (ppm)

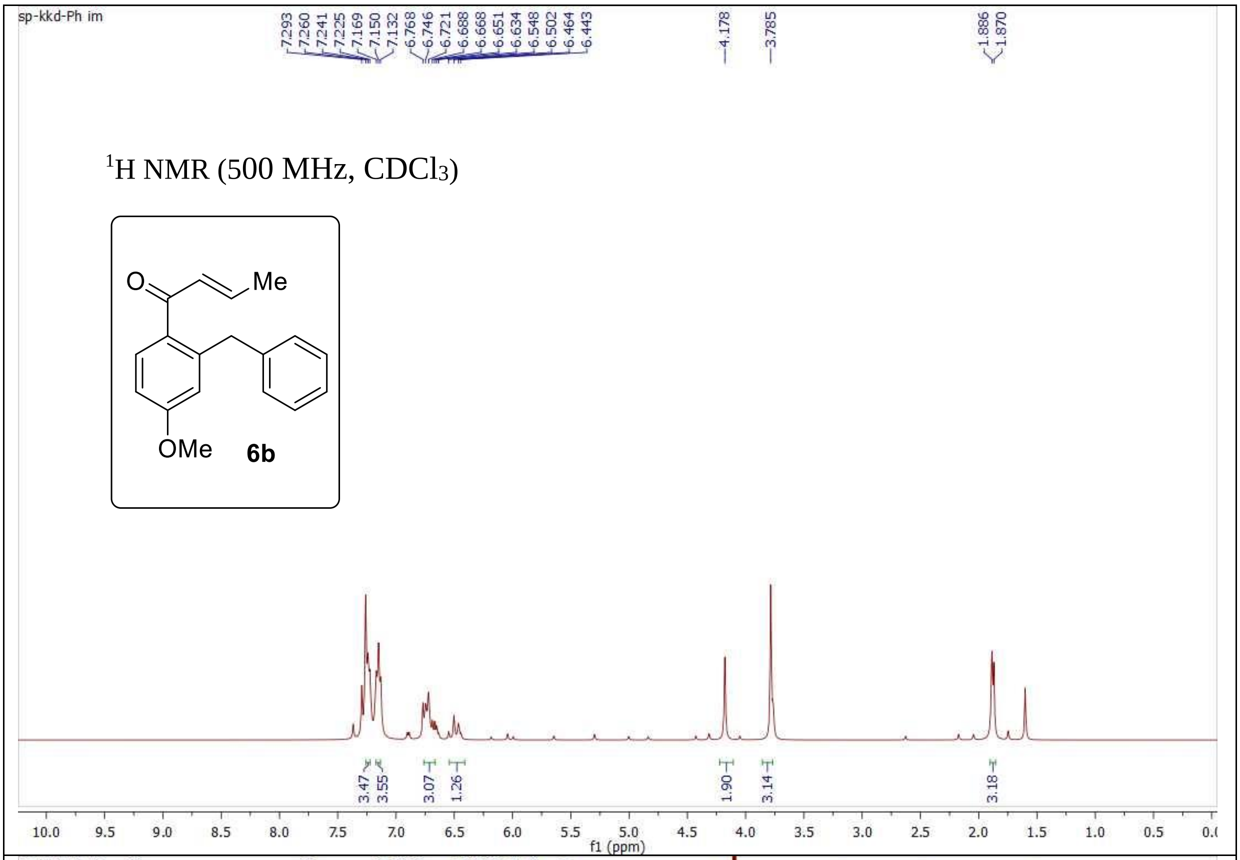
sp-kkd-Ph im
7.293
7.260
7.241
7.225
7.169
7.150
7.132
6.768
6.746
6.721
6.688
6.668
6.651
6.634
6.548
6.502
6.464
6.443
4.178
3.785
1.886
1.870
1H NMR (500 MHz, CDCl3)
O
Me
OMe
6b
3.47
3.55
3.07
1.26
1.90
3.14
3.18
f1 (ppm)

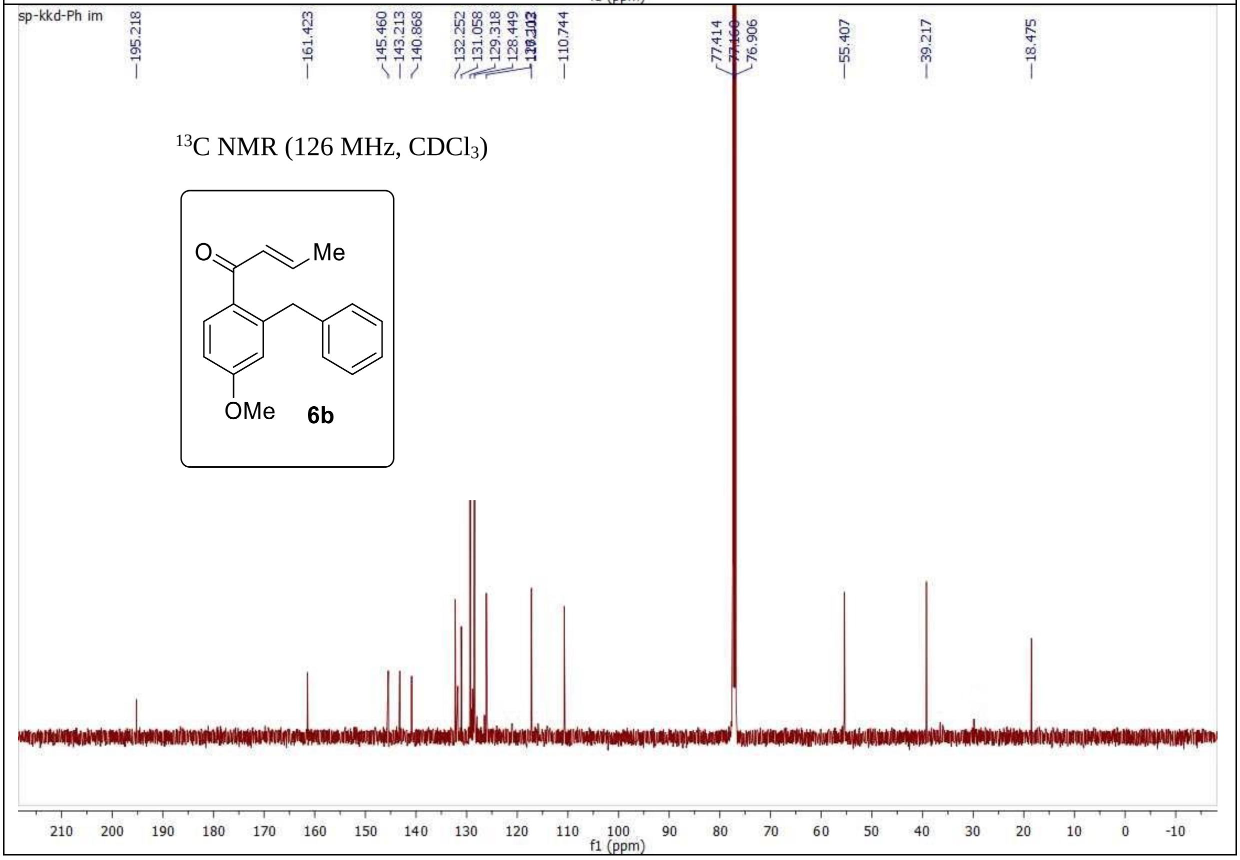
sp-kkd-Ph im
195.218
161.423
145.460
143.213
140.868
132.252
131.058
129.318
128.449
110.744
77.414
76.906
55.407
39.217
18.475
13C NMR (126 MHz, CDCl3)
O
Me
OMe
6b
f1 (ppm)

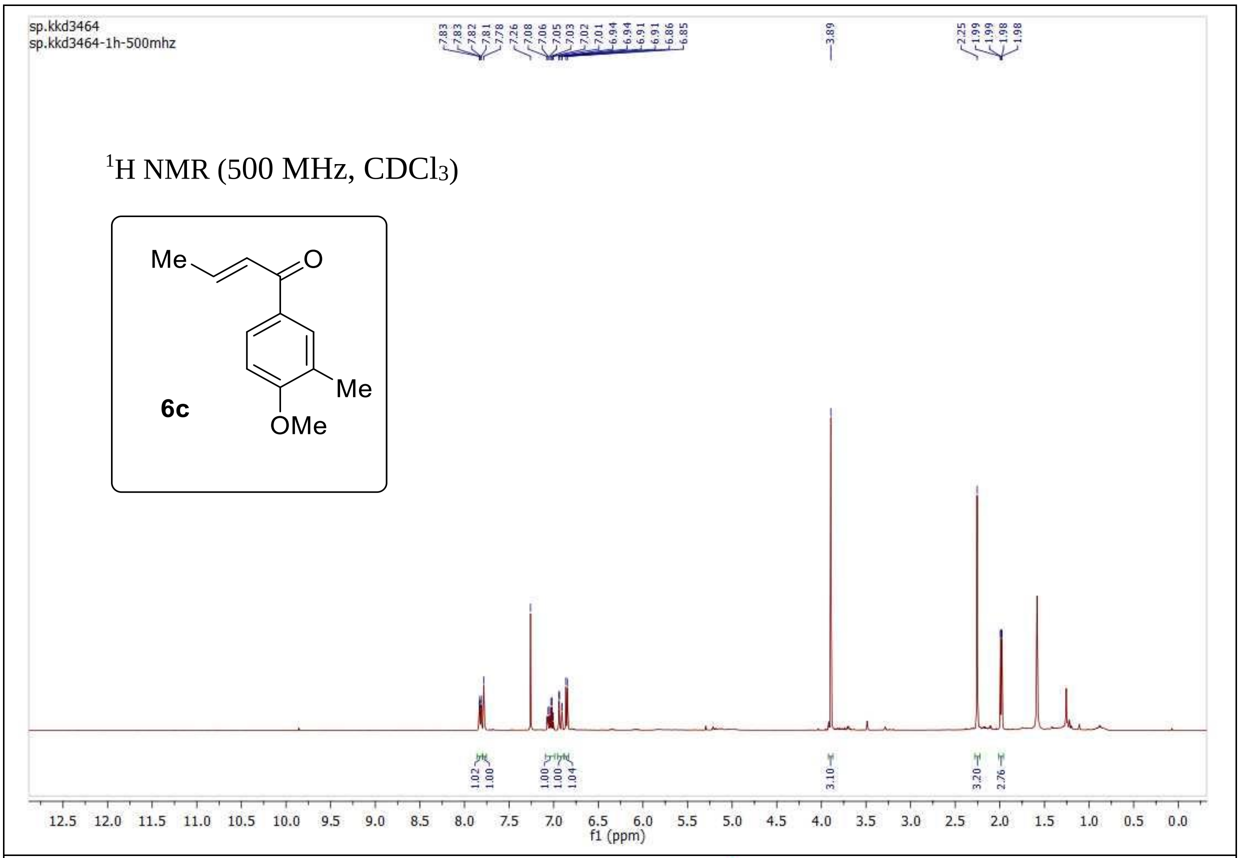
sp.kkd3464
sp.kkd3464-1h-500mhz
¹H NMR (500 MHz, CDCl3)
Me
O
Me
6c
OMe
f1 (ppm)

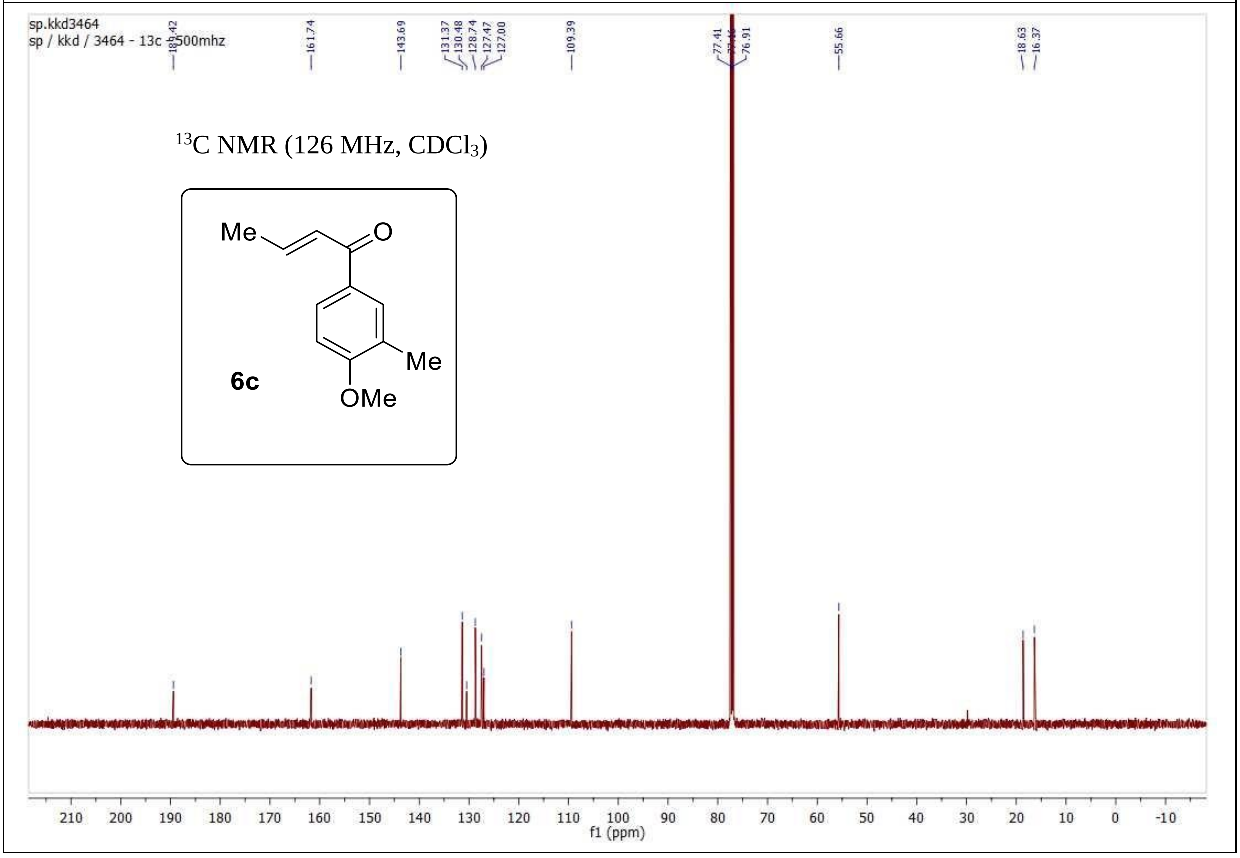
sp.kkd3464
sp / kkd / 3464 - 13c - 500mhz
¹³C NMR (126 MHz, CDCl3)
Me
O
Me
6c
OMe
f1 (ppm)

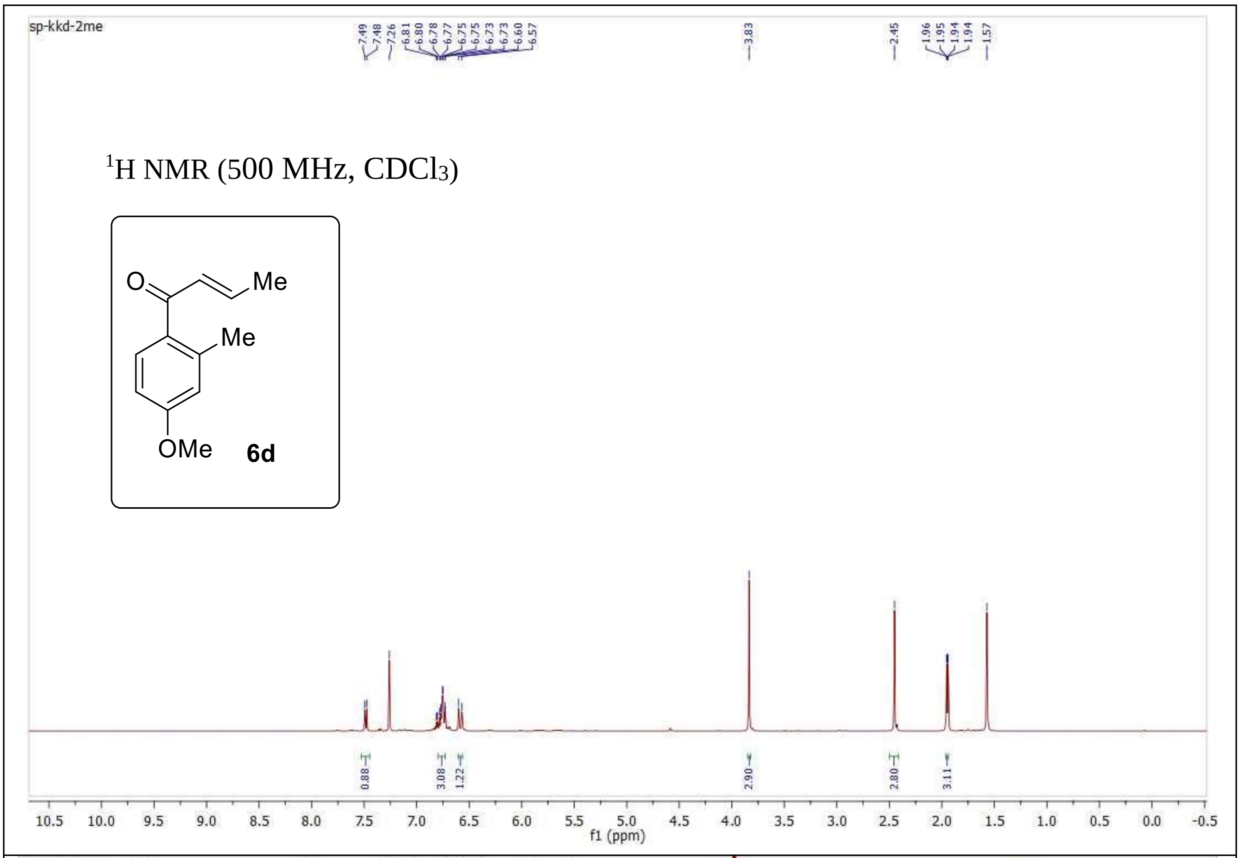
sp-kkd-2me
7.49
7.48
7.26
6.81
6.80
6.78
6.77
6.75
6.75
6.73
6.73
6.60
6.57
3.83
2.45
1.96
1.95
1.94
1.94
1.57
¹H NMR (500 MHz, CDCl₃)
O
Me
Me
OMe
6d
0.88
3.08
1.22
2.90
2.80
3.11
10.5 10.0 9.5 9.0 8.5 8.0 7.5 7.0 6.5 6.0 5.5 5.0 4.5 4.0 3.5 3.0 2.5 2.0 1.5 1.0 0.5 0.0 -0.5
f1 (ppm)

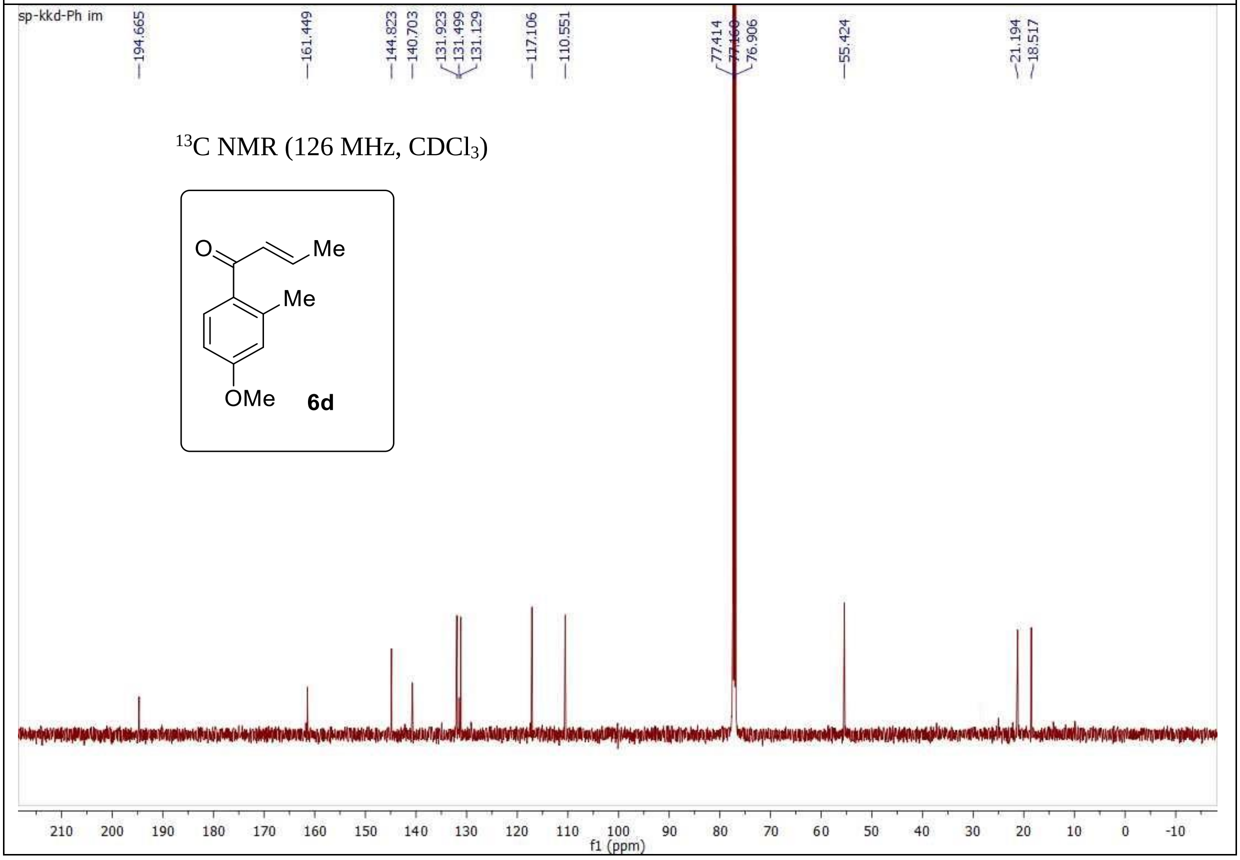
sp-kkd-Ph im
194.665
161.449
144.823
140.703
131.923
131.499
131.129
117.106
110.551
77.414
77.160
76.906
55.424
21.194
18.517
¹³C NMR (126 MHz, CDCl₃)
O
Me
Me
OMe
6d
210 200 190 180 170 160 150 140 130 120 110 100 90 80 70 60 50 40 30 20 10 0 -10
f1 (ppm)

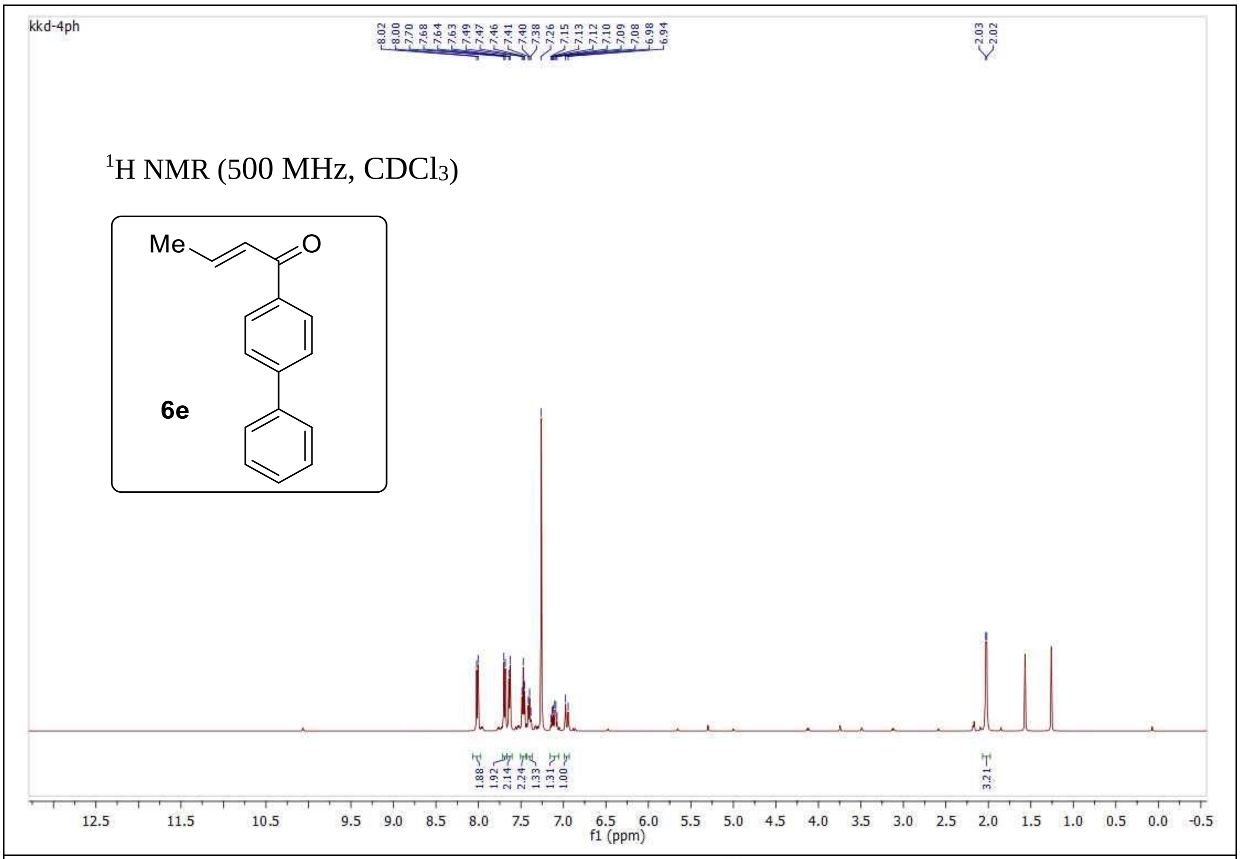

kkd-4ph
^{1}H NMR (500 MHz, $CDCl_3$)
Me
O
6e
f1 (ppm)

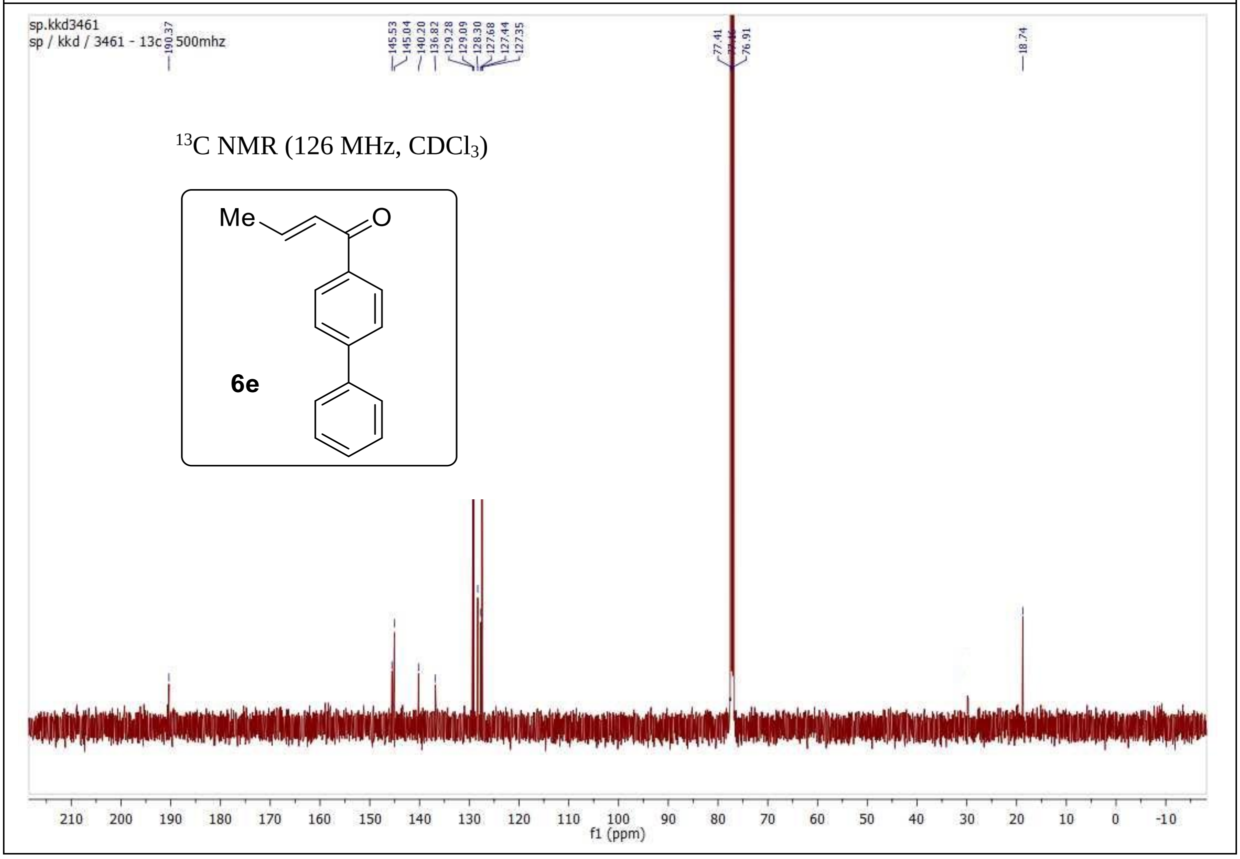

sp.kkd3461
sp / kkd / 3461 - 13c 500mhz
^{13}C NMR (126 MHz, $CDCl_3$)
Me
O
6e
f1 (ppm)

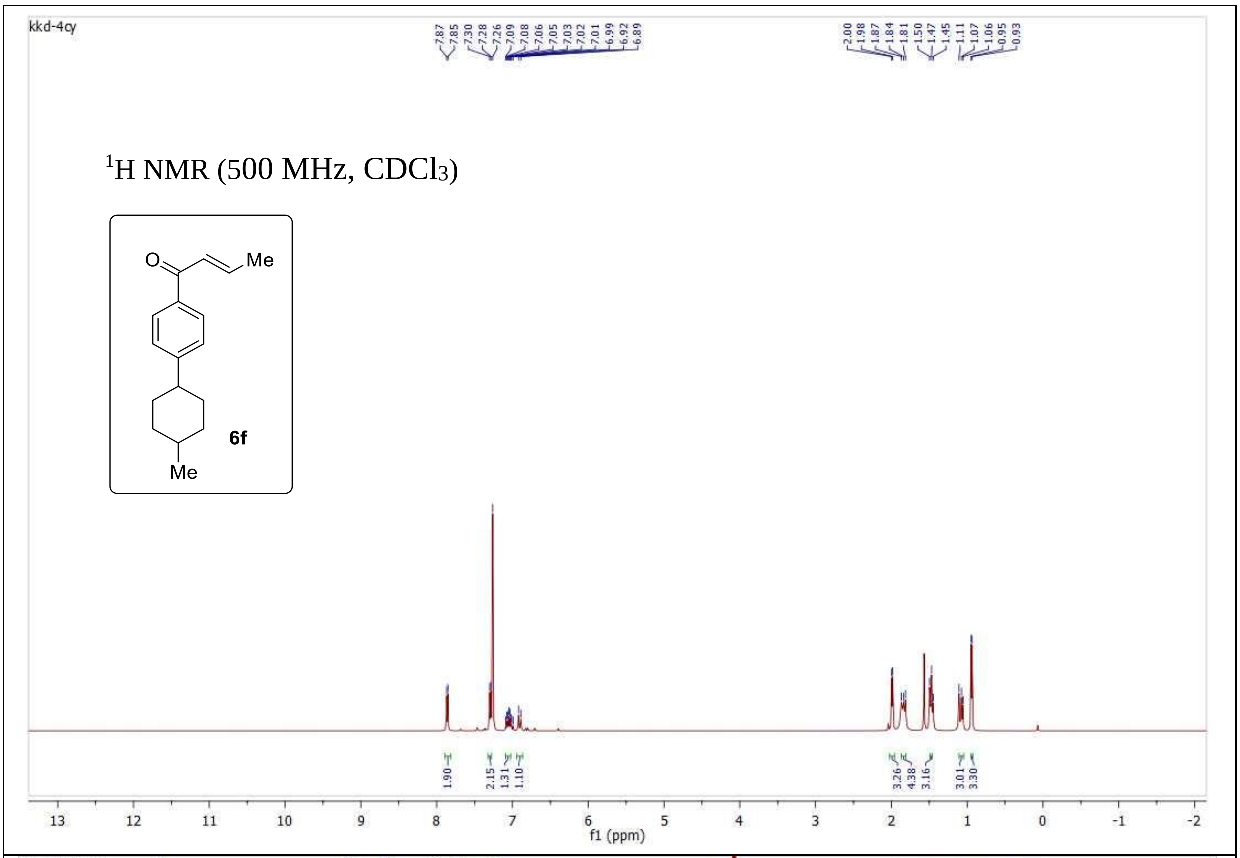

kkd-4cy
1H NMR (500 MHz, CDCl3)
O
Me
6f
Me
f1 (ppm)

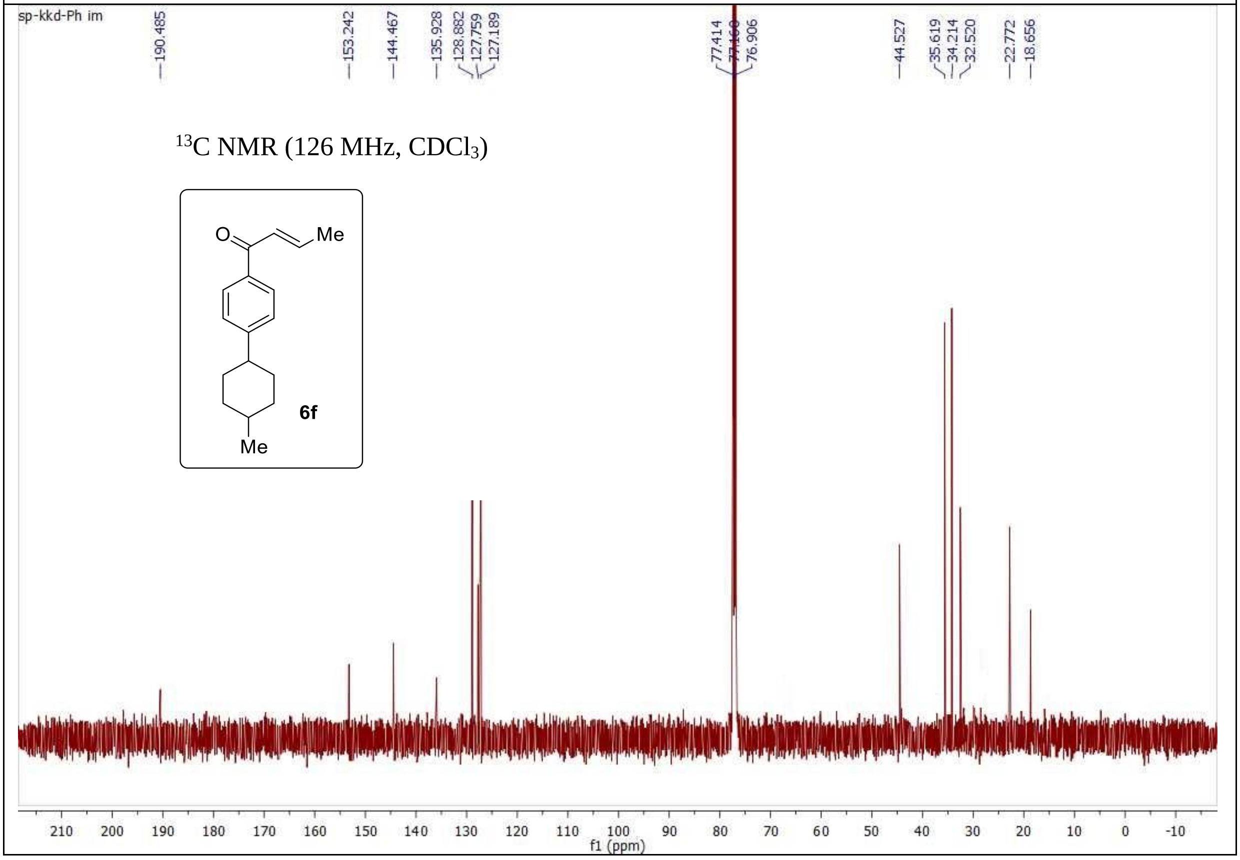

sp-kkd-Ph im
190.485
153.242
144.467
135.928
128.882
127.759
127.189
77.414
76.906
44.527
35.619
34.214
32.520
22.772
18.656
13C NMR (126 MHz, CDCl3)
O
Me
6f
Me
f1 (ppm)

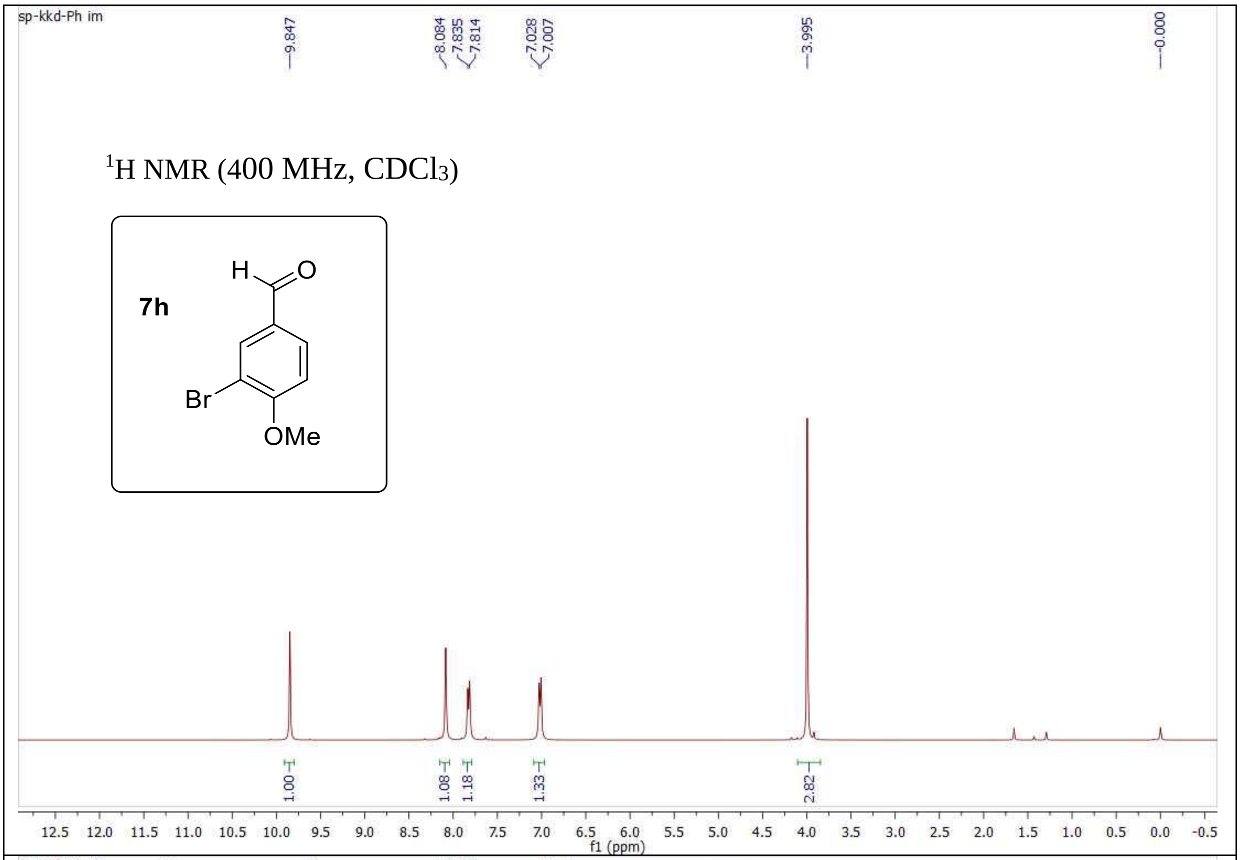
sp-kkd-Ph im
9.847
8.084
7.835
7.814
7.028
7.007
3.995
0.000
1H NMR (400 MHz, CDCl3)
7h
H
O
Br
OMe
1.00
1.08
1.18
1.33
2.82
12.5
12.0
11.5
11.0
10.5
10.0
9.5
9.0
8.5
8.0
7.5
7.0
6.5
6.0
5.5
5.0
4.5
4.0
3.5
3.0
2.5
2.0
1.5
1.0
0.5
0.0
-0.5
f1 (ppm)

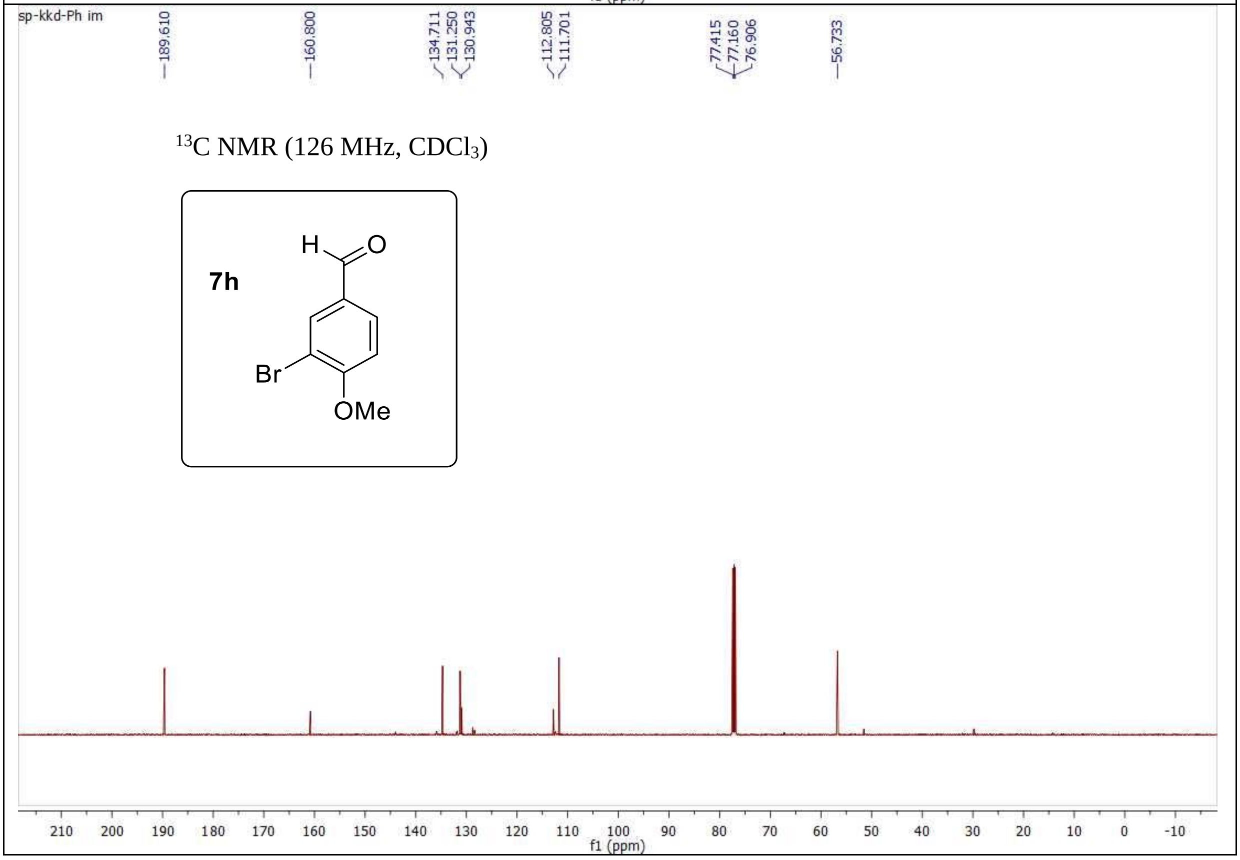
sp-kkd-Ph im
189.610
160.800
134.711
131.250
130.943
112.805
111.701
77.415
77.160
76.906
56.733
13C NMR (126 MHz, CDCl3)
7h
H
O
Br
OMe
210
200
190
180
170
160
150
140
130
120
110
100
90
80
70
60
50
40
30
20
10
0
-10
f1 (ppm)

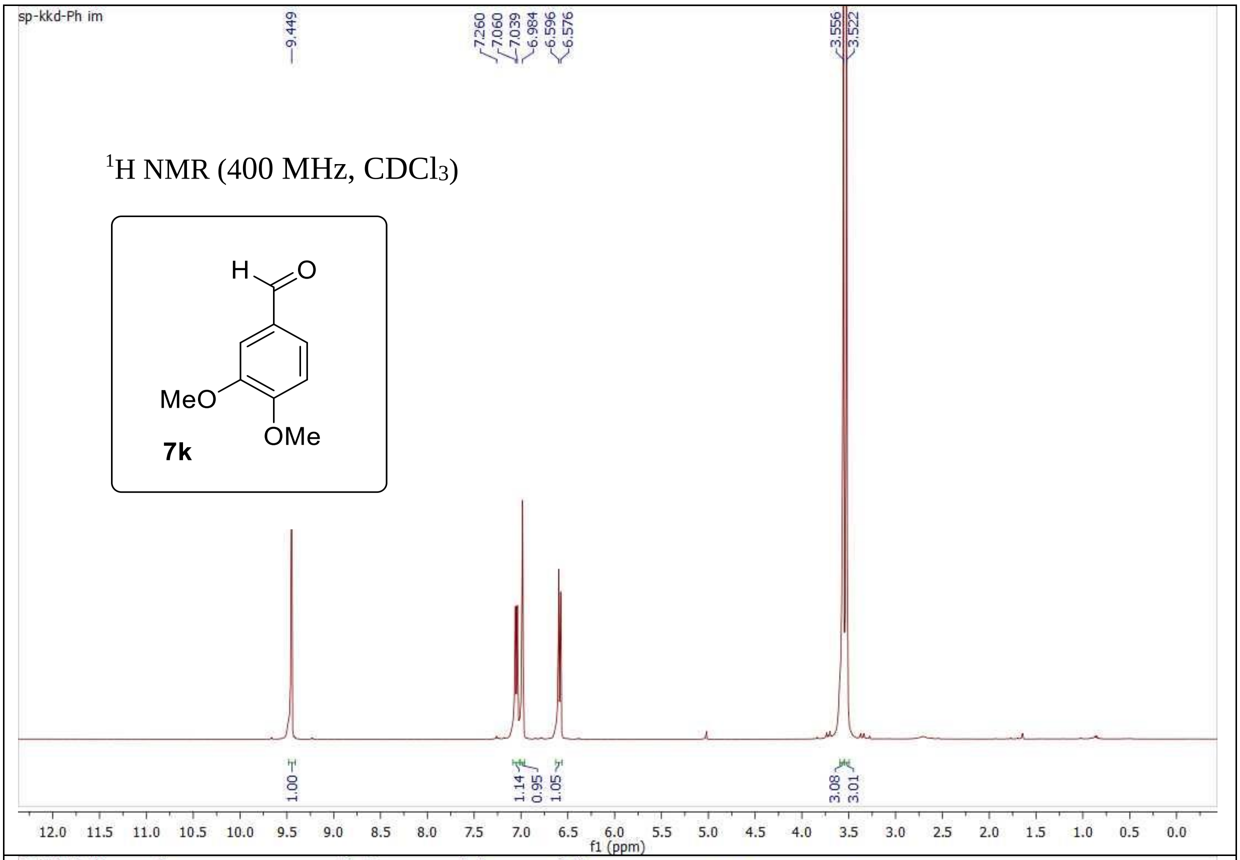
sp-kkd-Ph im
9.449
7.260
7.060
7.039
6.984
6.596
6.576
3.556
3.522
1H NMR (400 MHz, CDCl3)
H
O
MeO
OMe
7k
1.00
1.14
0.95
1.05
3.08
3.01
12.0
11.5
11.0
10.5
10.0
9.5
9.0
8.5
8.0
7.5
7.0
6.5
6.0
5.5
5.0
4.5
4.0
3.5
3.0
2.5
2.0
1.5
1.0
0.5
0.0
f1 (ppm)

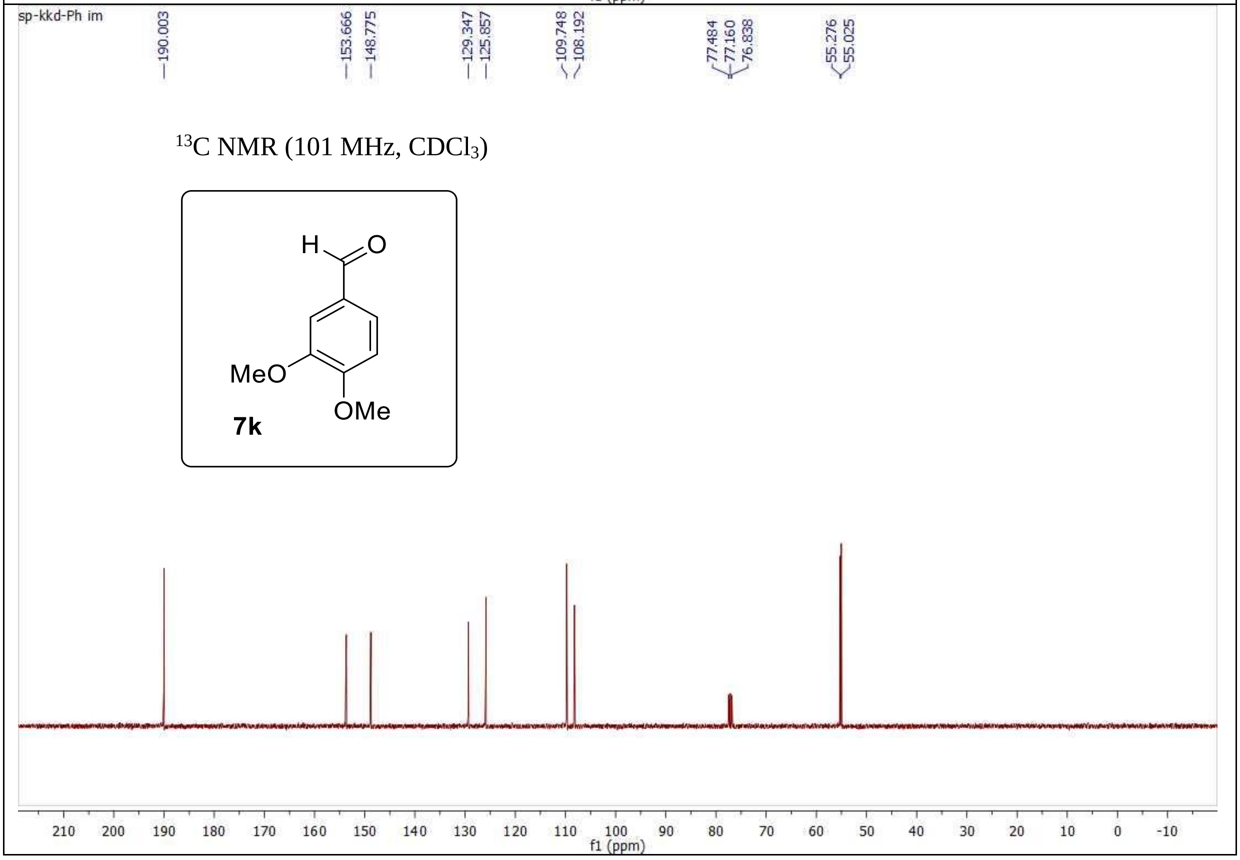
sp-kkd-Ph im
190.003
153.666
148.775
129.347
125.857
109.748
108.192
77.484
77.160
76.838
55.276
55.025
13C NMR (101 MHz, CDCl3)
H
O
MeO
OMe
7k
210
200
190
180
170
160
150
140
130
120
110
100
90
80
70
60
50
40
30
20
10
0
-10
f1 (ppm)

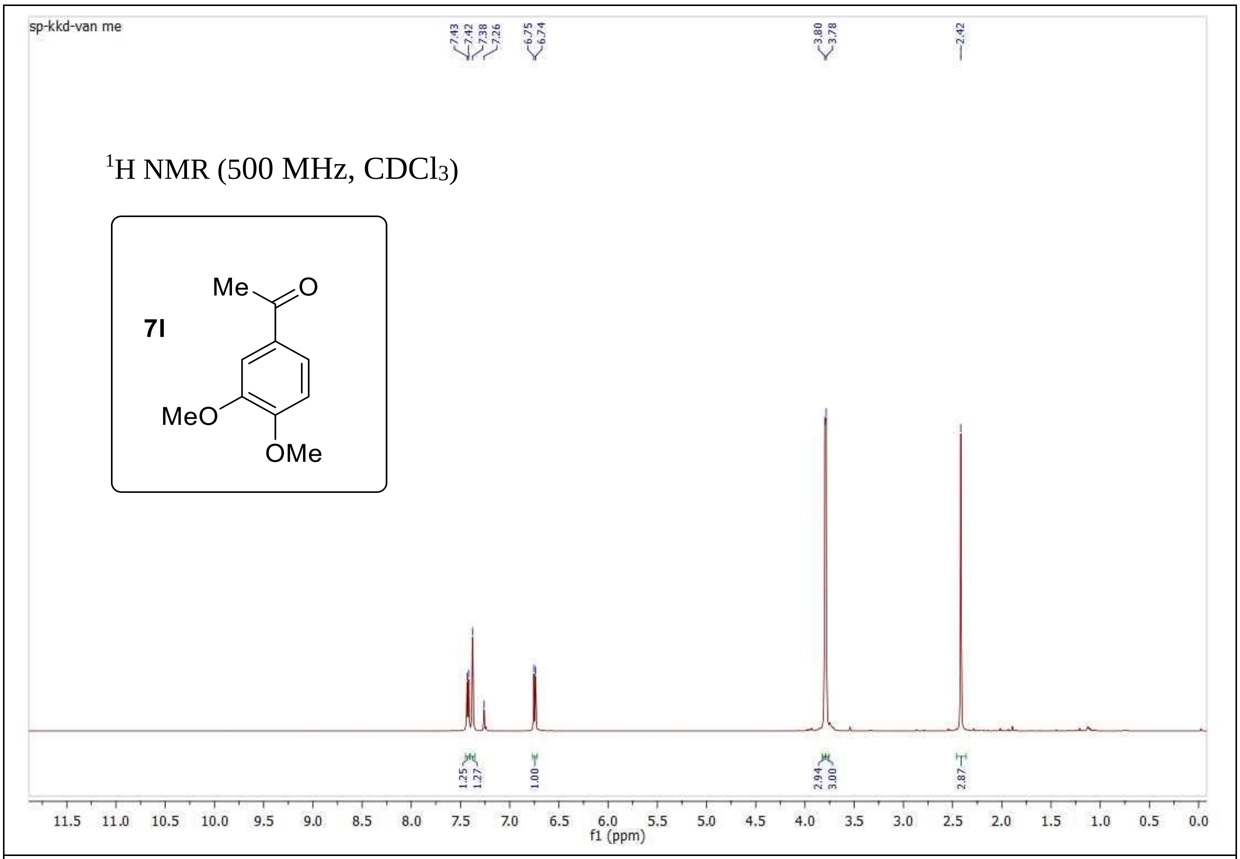
sp-kkd-van me
7.43
7.42
7.38
7.26
6.75
6.74
3.80
3.78
2.42
1H NMR (500 MHz, CDCl3)
Me
O
7l
MeO
OMe
1.25
1.27
1.00
2.94
3.00
2.87
11.5
11.0
10.5
10.0
9.5
9.0
8.5
8.0
7.5
7.0
6.5
6.0
5.5
5.0
4.5
4.0
3.5
3.0
2.5
2.0
1.5
1.0
0.5
0.0
f1 (ppm)

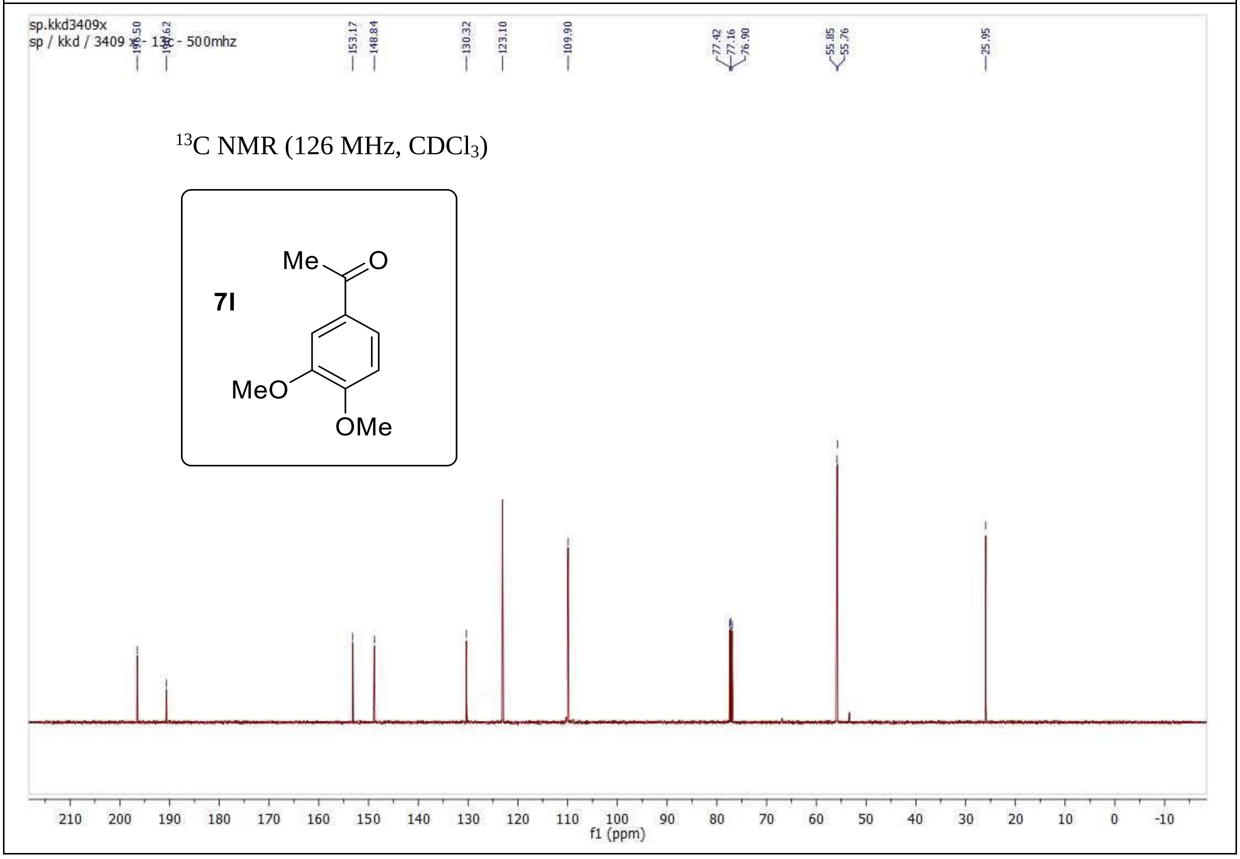
sp.kkd3409x
sp / kkd / 3409 - 13c - 500mhz
153.17
148.84
130.32
123.10
109.90
77.42
77.16
76.90
55.85
55.76
25.95
13C NMR (126 MHz, CDCl3)
Me
O
7l
MeO
OMe
210
200
190
180
170
160
150
140
130
120
110
100
90
80
70
60
50
40
30
20
10
0
-10
f1 (ppm)

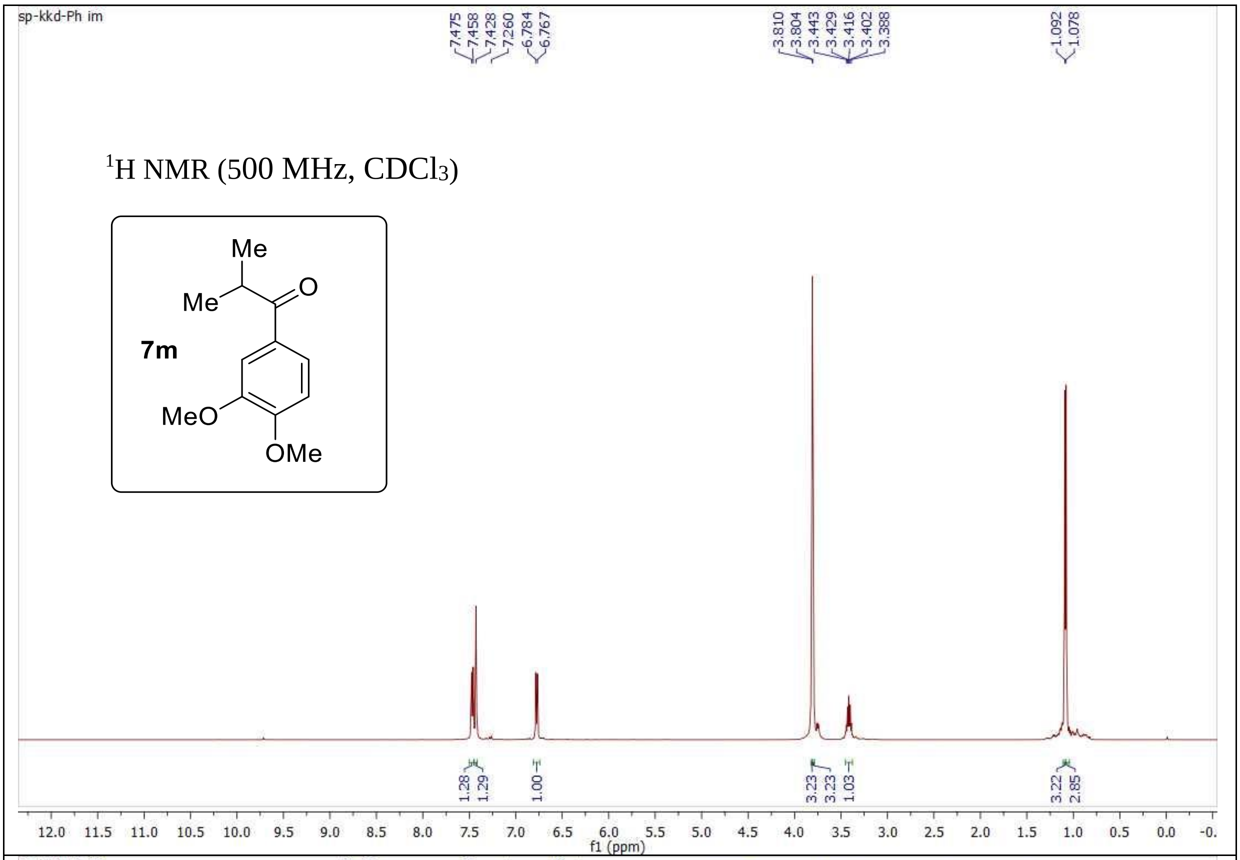
sp-kkd-Ph im
7.475
7.458
7.428
7.260
6.784
6.767
3.810
3.804
3.443
3.429
3.416
3.402
3.388
1.092
1.078
^{1}H NMR (500 MHz, CDCl$_3$)
Me
Me
O
7m
MeO
OMe
1.28
1.29
1.00
3.23
3.23
1.03
3.22
2.85
12.0
11.5
11.0
10.5
10.0
9.5
9.0
8.5
8.0
7.5
7.0
6.5
6.0
5.5
5.0
4.5
4.0
3.5
3.0
2.5
2.0
1.5
1.0
0.5
0.0
f1 (ppm)

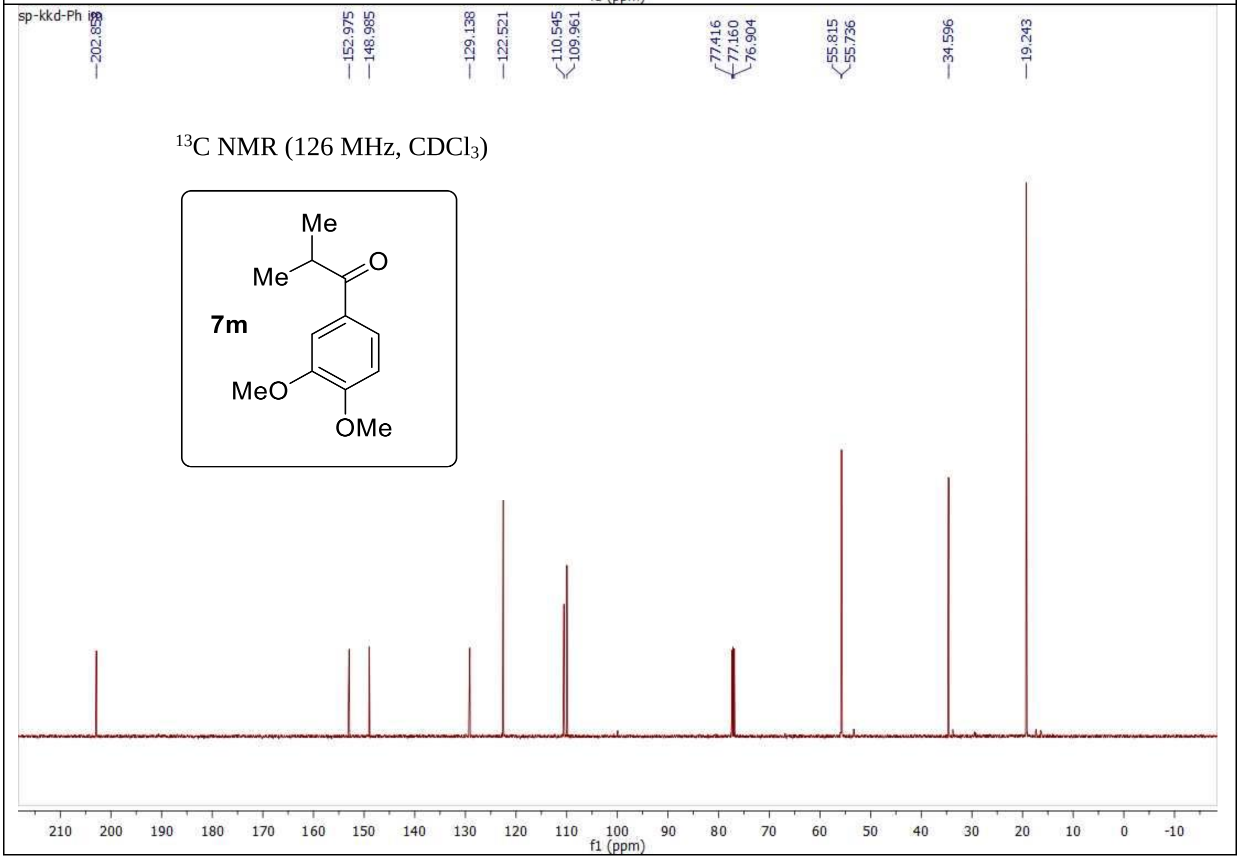
sp-kkd-Ph im
202.858
152.975
148.985
129.138
122.521
110.545
109.961
77.416
77.160
76.904
55.815
55.736
34.596
19.243
^{13}C NMR (126 MHz, CDCl$_3$)
Me
Me
O
7m
MeO
OMe
210
200
190
180
170
160
150
140
130
120
110
100
90
80
70
60
50
40
30
20
10
0
-10
f1 (ppm)

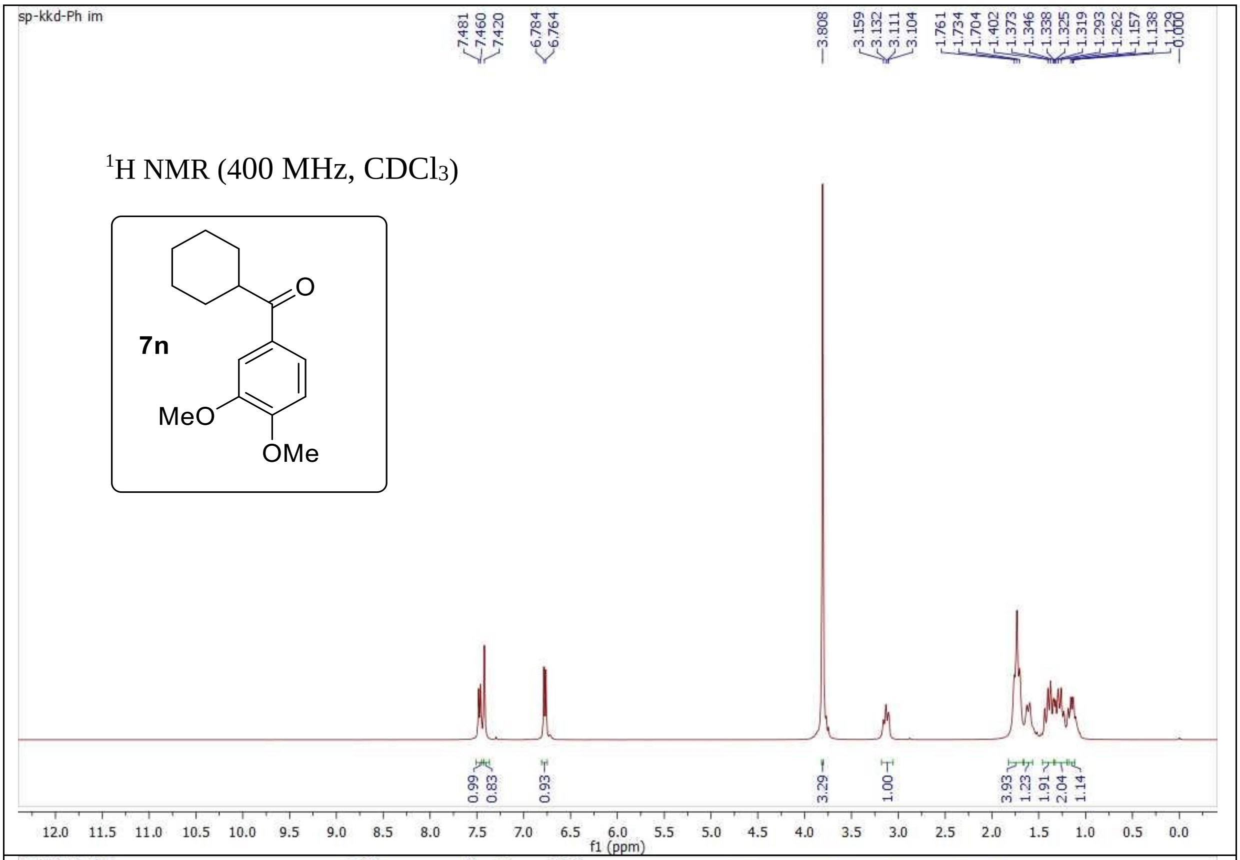
sp-kkd-Ph im
7.481
7.460
7.420
6.784
6.764
3.808
3.159
3.132
3.111
3.104
1.761
1.734
1.704
1.402
1.373
1.346
1.338
1.325
1.319
1.293
1.262
1.157
1.138
1.129
0.000
¹H NMR (400 MHz, CDCl3)
7n
O
MeO
OMe
0.99
0.83
0.93
3.29
1.00
3.93
1.23
1.91
2.04
1.14
f1 (ppm)

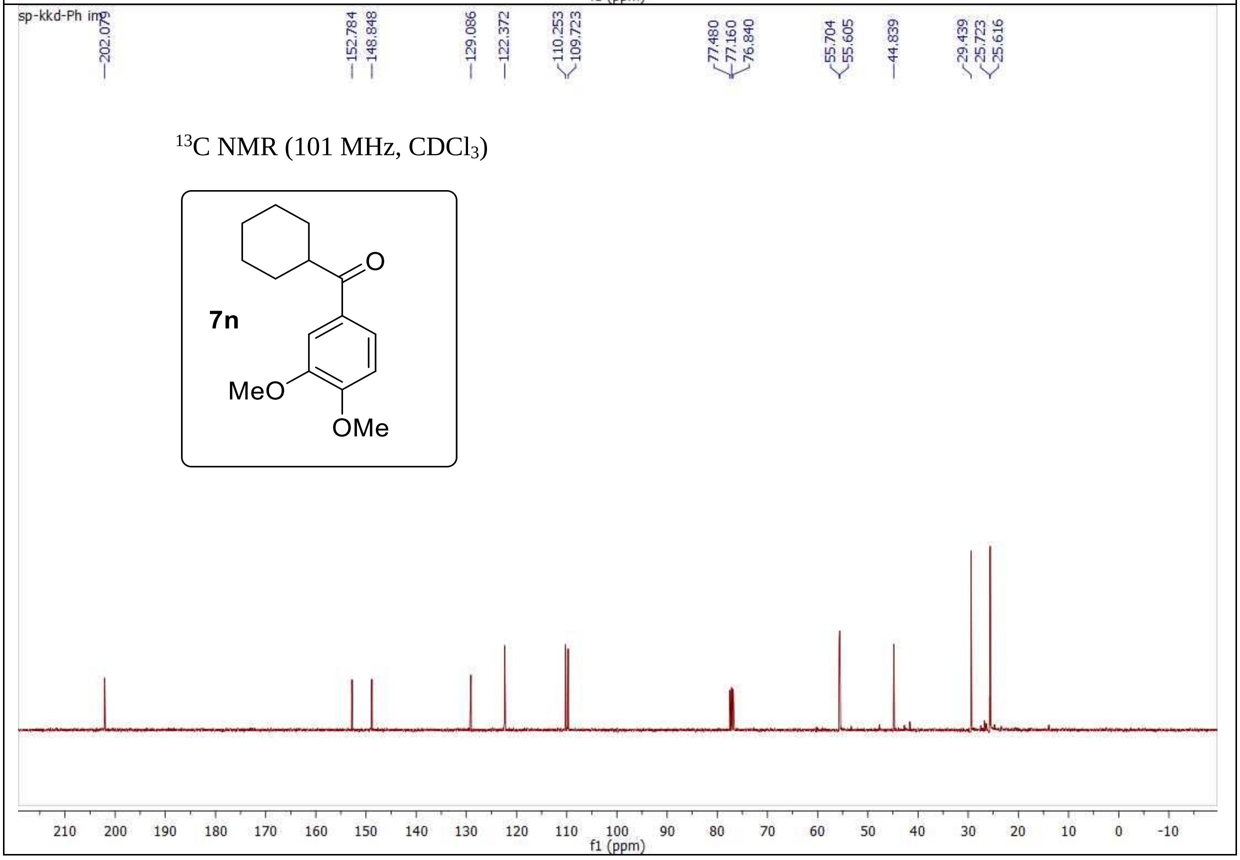
sp-kkd-Ph im
202.079
152.784
148.848
129.086
122.372
110.253
109.723
77.480
77.160
76.840
55.704
55.605
44.839
29.439
25.723
25.616
¹³C NMR (101 MHz, CDCl3)
7n
O
MeO
OMe
f1 (ppm)

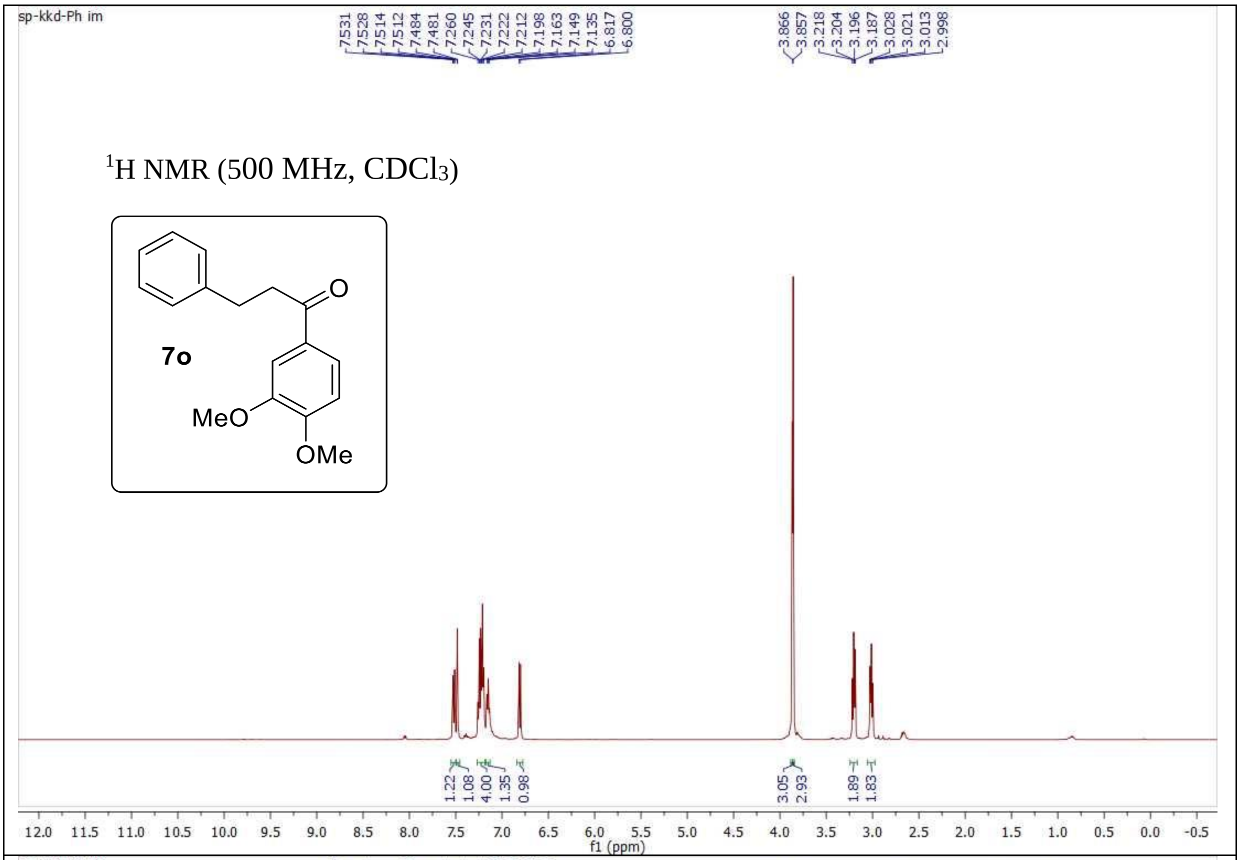
sp-kkd-Ph im
7.531 7.528 7.514 7.512 7.484 7.481 7.260 7.245 7.231 7.222 7.212 7.198 7.163 7.149 7.135 6.817 6.800
3.866 3.857 3.218 3.204 3.196 3.187 3.028 3.021 3.013 2.998
1H NMR (500 MHz, CDCl3)
7o
O
MeO
OMe
1.22 1.08 4.00 1.35 0.98
3.05 2.93
1.89 1.83
f1 (ppm)

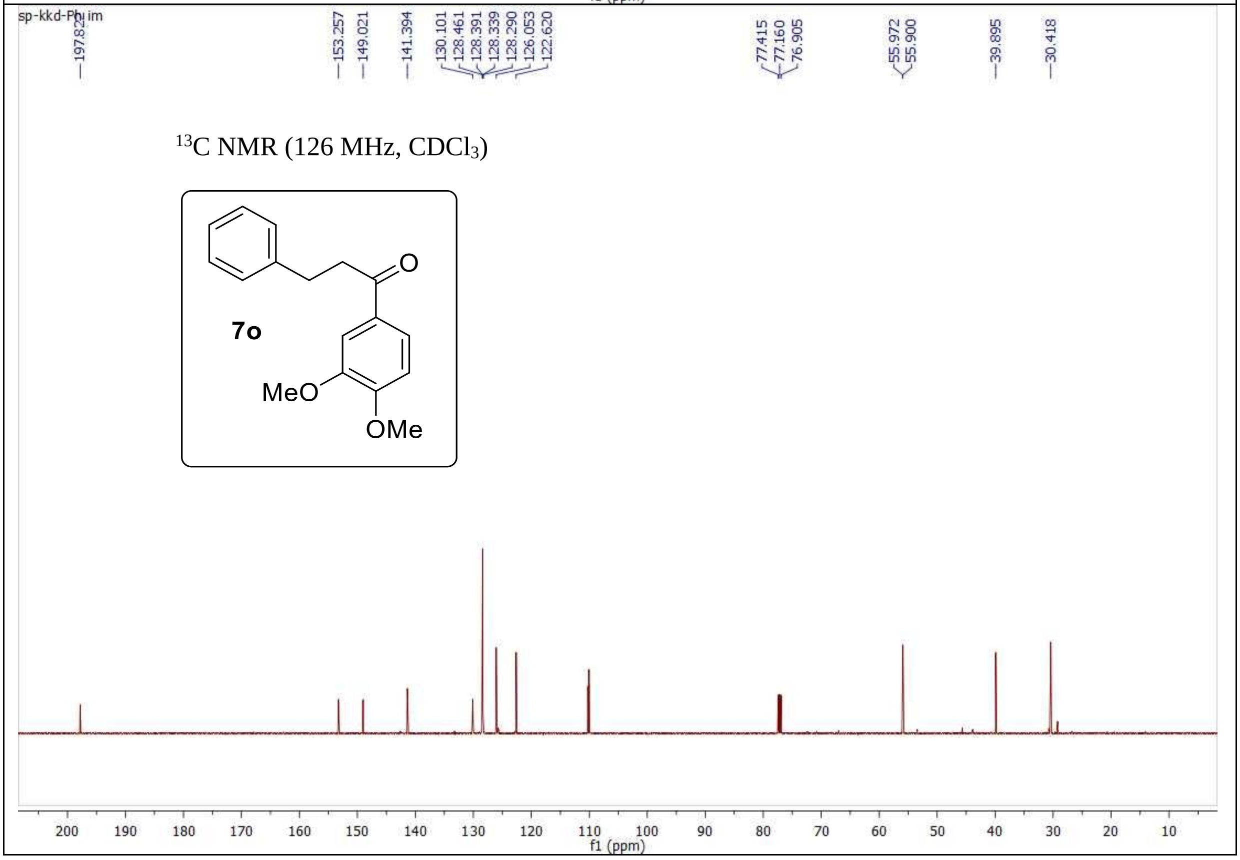
sp-kkd-Ph im
197.822
153.257 149.021 141.394 130.101 128.461 128.391 128.339 128.290 126.053 122.620
77.415 77.160 76.905
55.972 55.900
39.895
30.418
13C NMR (126 MHz, CDCl3)
7o
O
MeO
OMe
f1 (ppm)

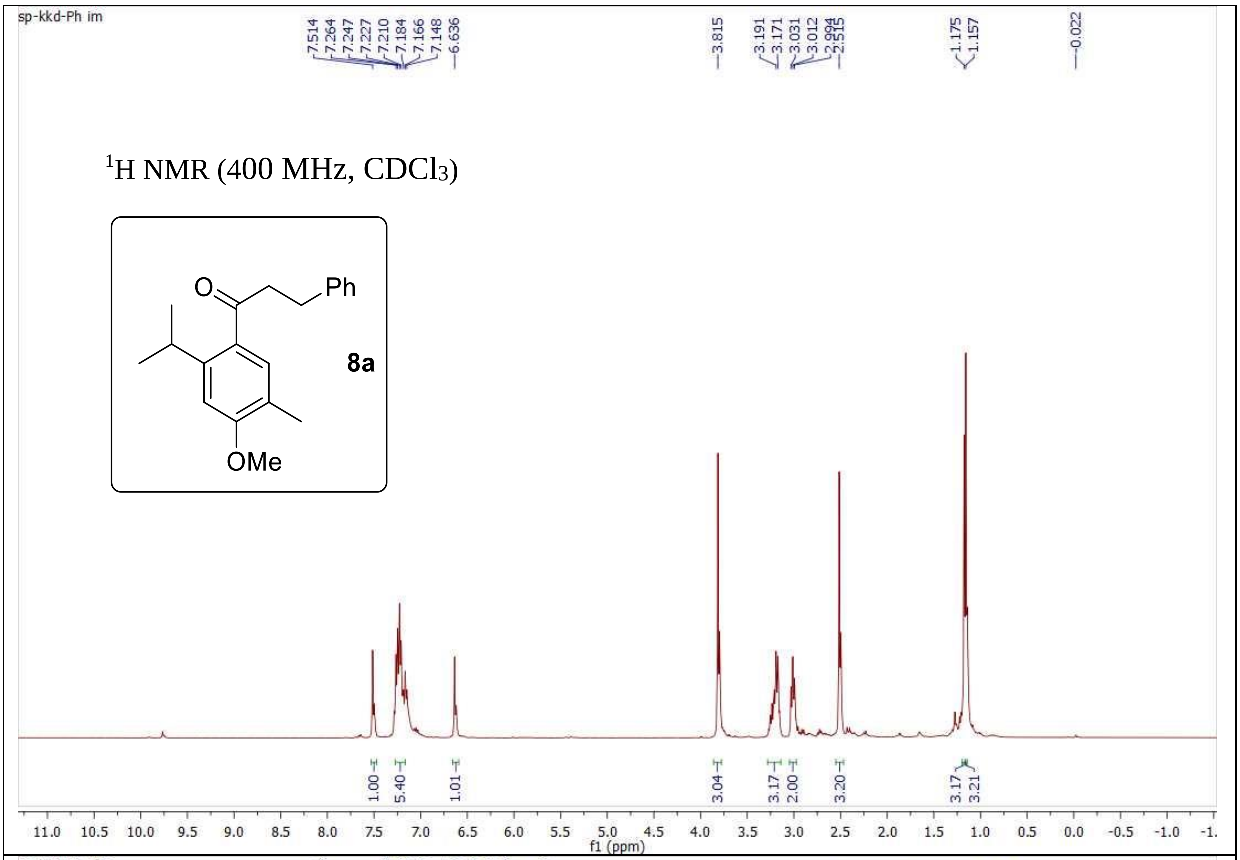

sp-kkd-Ph im
7.514
7.264
7.247
7.227
7.210
7.184
7.166
7.148
6.636
3.815
3.191
3.171
3.031
3.012
2.515
1.175
1.157
0.022
1H NMR (400 MHz, CDCl3)
O
Ph
8a
OMe
1.00
5.40
1.01
3.04
3.17
2.00
3.20
3.17
3.21
11.0 10.5 10.0 9.5 9.0 8.5 8.0 7.5 7.0 6.5 6.0 5.5 5.0 4.5 4.0 3.5 3.0 2.5 2.0 1.5 1.0 0.5 0.0 -0.5 -1.0
f1 (ppm)

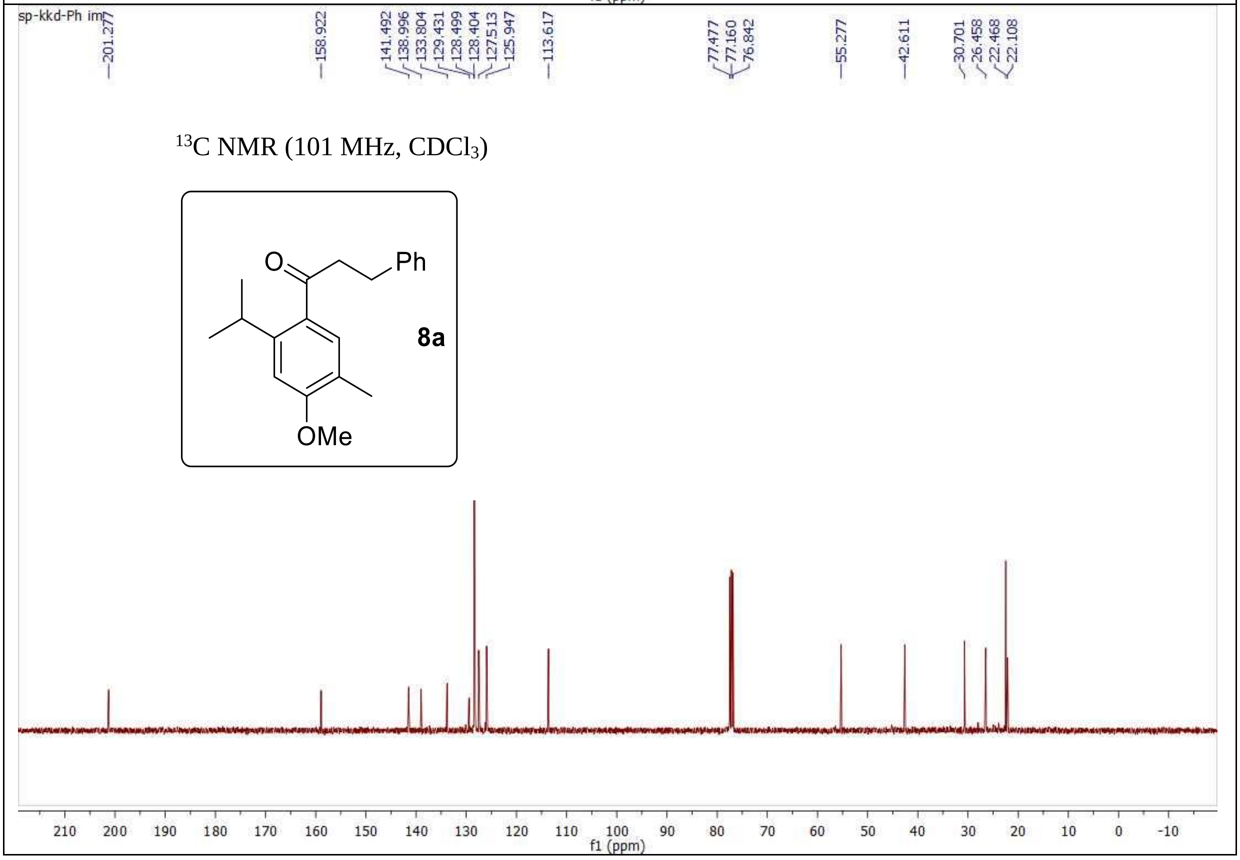

sp-kkd-Ph im
201.277
158.922
141.492
138.996
133.804
129.431
128.499
128.404
127.513
125.947
113.617
77.477
77.160
76.842
55.277
42.611
30.701
26.458
22.468
22.108
13C NMR (101 MHz, CDCl3)
O
Ph
8a
OMe
210 200 190 180 170 160 150 140 130 120 110 100 90 80 70 60 50 40 30 20 10 0 -10
f1 (ppm)

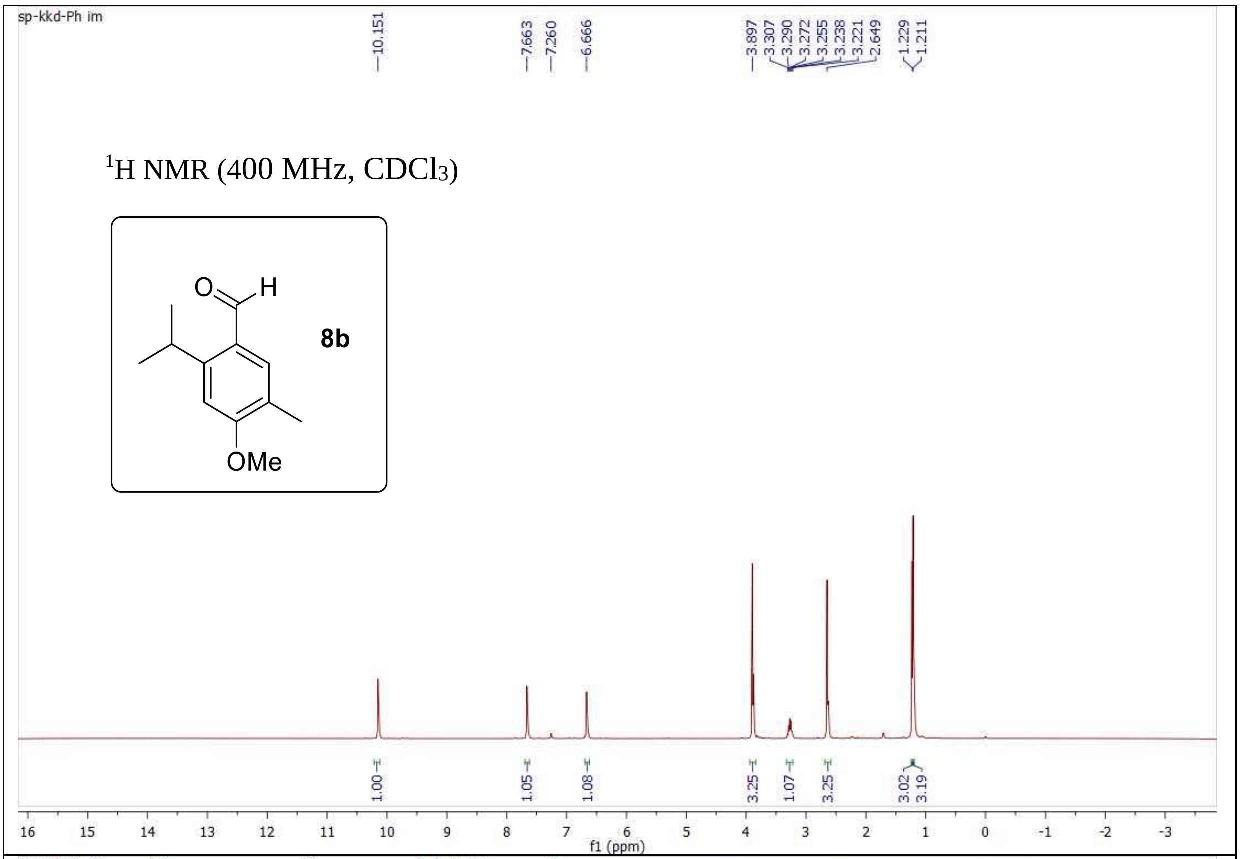
sp-kkd-Ph im
10.151
7.663
7.260
6.666
3.897
3.307
3.290
3.272
3.255
3.238
3.221
2.649
1.229
1.211
1H NMR (400 MHz, CDCl3)
8b
OMe
1.00
1.05
1.08
3.25
1.07
3.25
3.02
3.19
f1 (ppm)

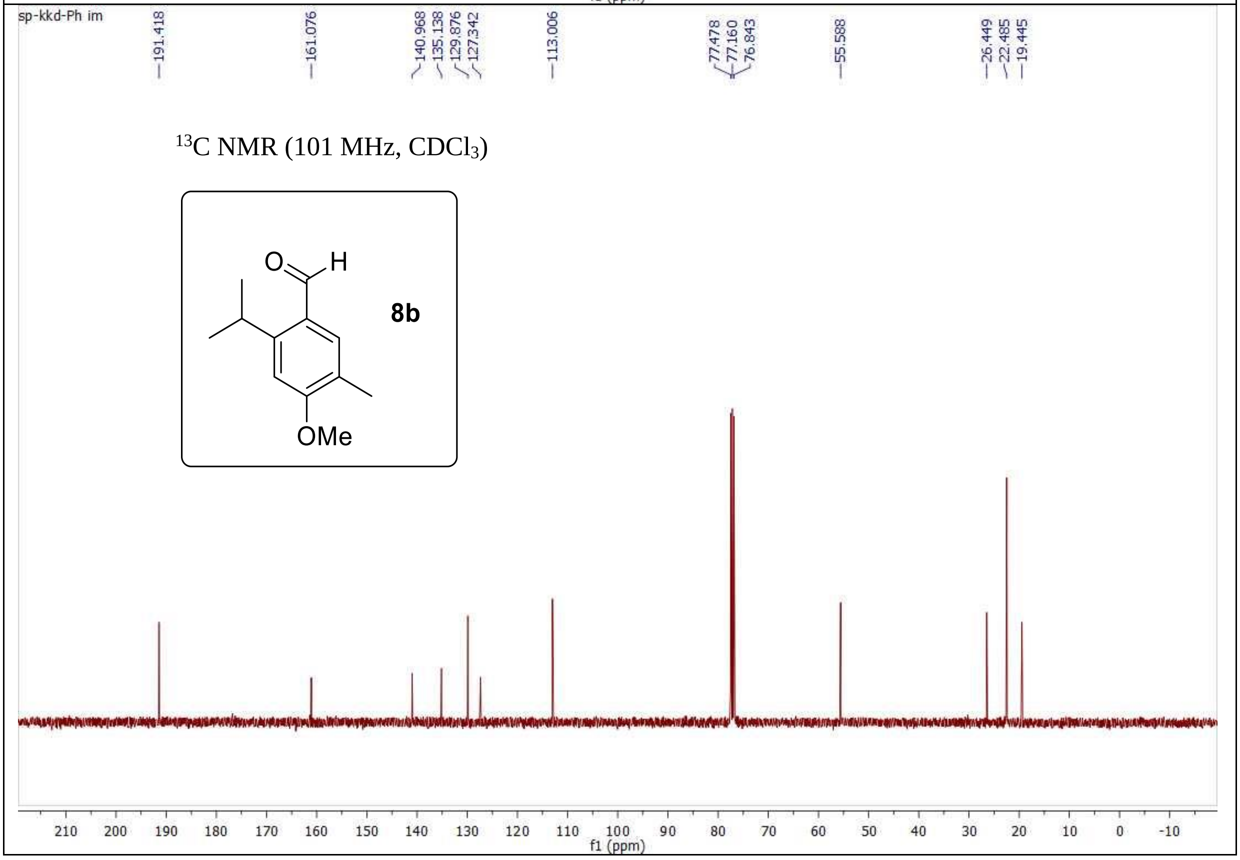
sp-kkd-Ph im
191.418
161.076
140.968
135.138
129.876
127.342
113.006
77.478
77.160
76.843
55.588
26.449
22.485
19.445
13C NMR (101 MHz, CDCl3)
8b
OMe
f1 (ppm)

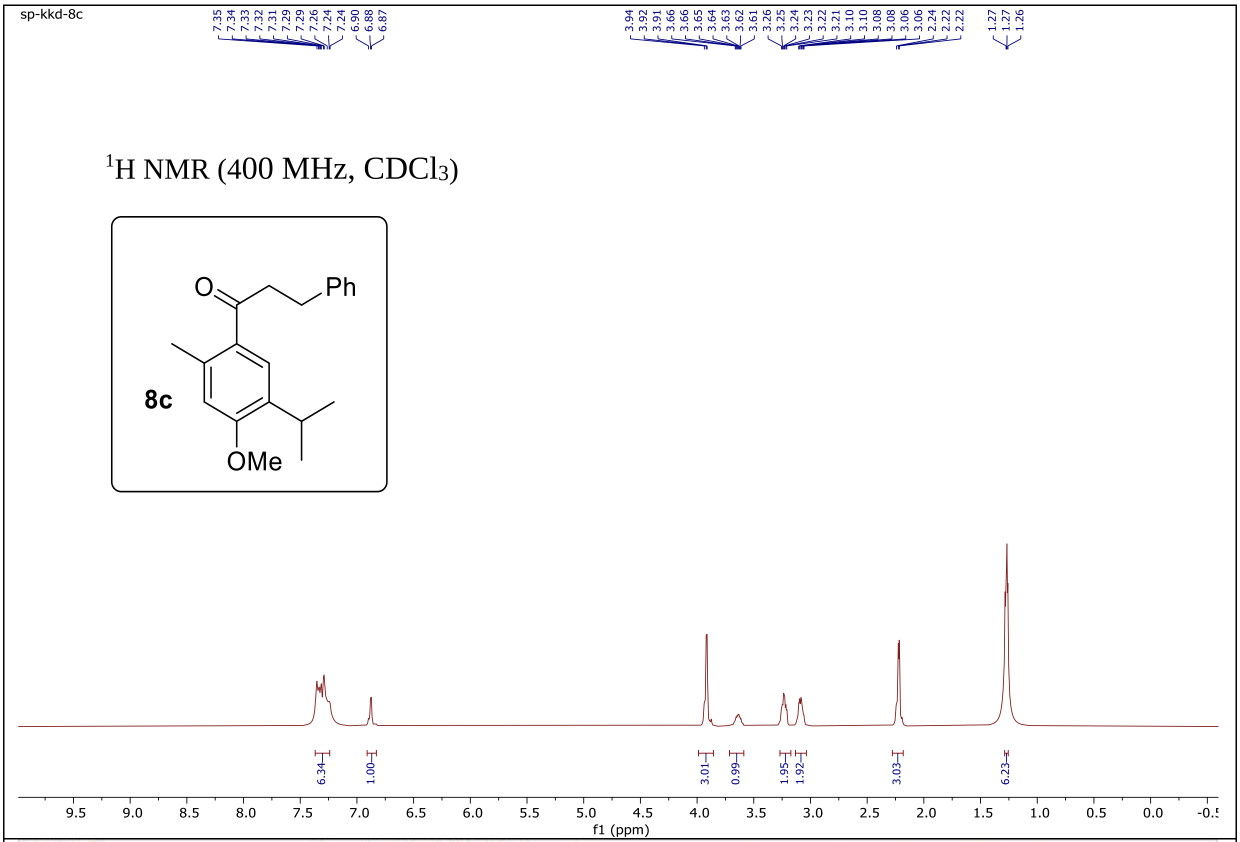
sp-kkd-8c
7.35 7.34 7.33 7.32 7.31 7.29 7.29 7.26 7.24 7.24 6.90 6.88 6.87
3.94 3.92 3.91 3.66 3.66 3.65 3.64 3.63 3.62 3.61 3.26 3.25 3.24 3.23 3.22 3.21 3.10 3.10 3.08 3.08 3.06 3.06 2.24 2.22 2.22
1.27 1.27 1.26
^{1}H NMR (400 MHz, $CDCl_3$)
O
Ph
8c
OMe
6.34
1.00
3.01
0.99
1.95
1.92
3.03
6.23
f1 (ppm)

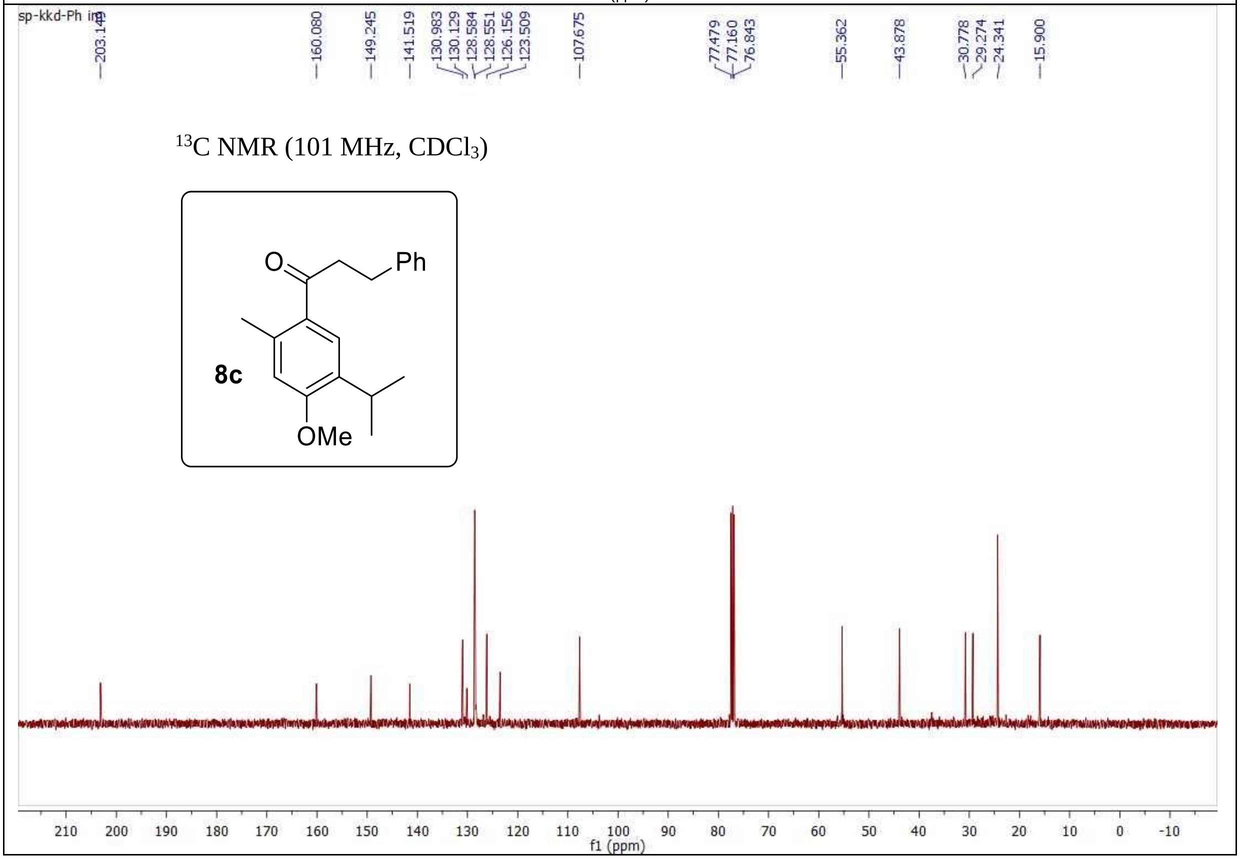
sp-kkd-Ph im
203.149
160.080
149.245
141.519
130.983
130.129
128.584
128.551
126.156
123.509
107.675
77.479
77.160
76.843
55.362
43.878
30.778
29.274
24.341
15.900
^{13}C NMR (101 MHz, $CDCl_3$)
O
Ph
8c
OMe
f1 (ppm)

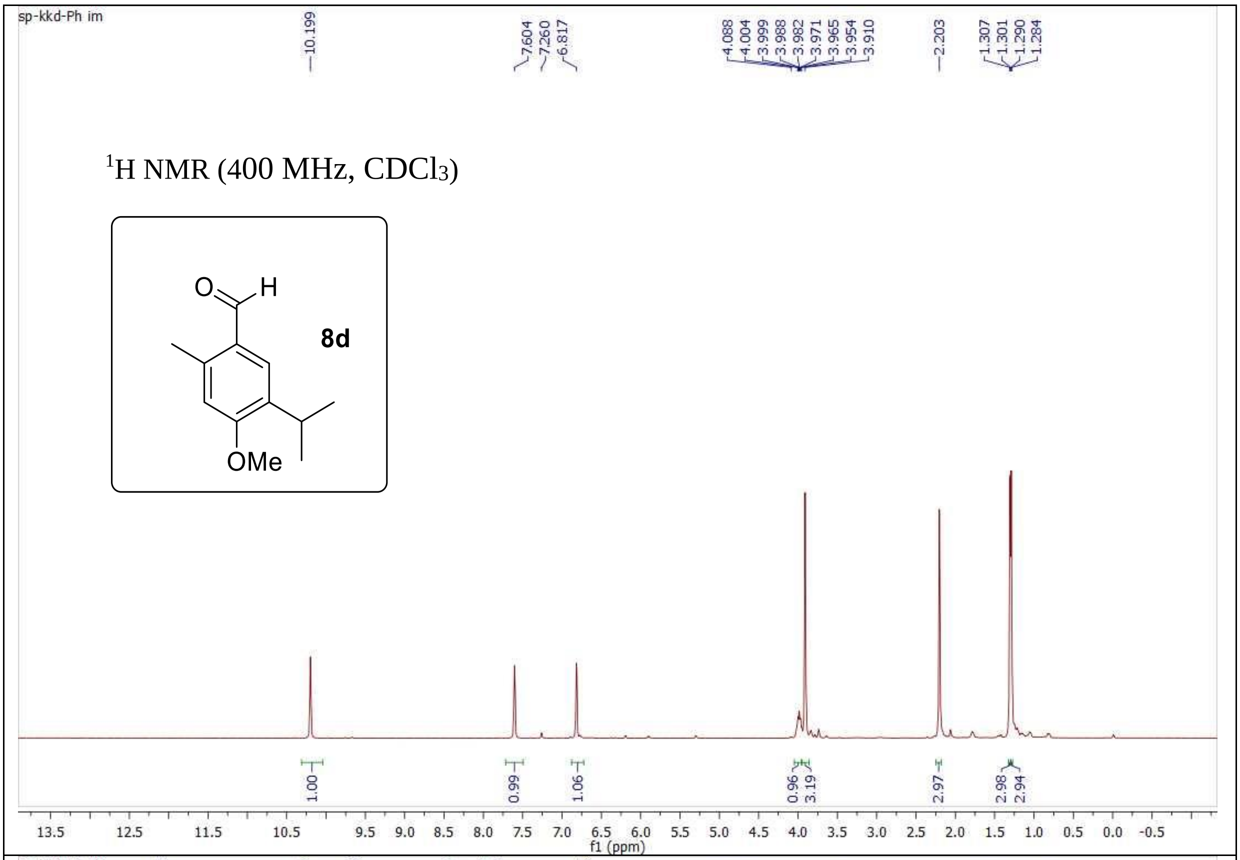
sp-kkd-Ph im
10.199
7.604
7.260
6.817
4.088
4.004
3.999
3.988
3.982
3.971
3.965
3.954
3.910
2.203
1.307
1.301
1.290
1.284
^{1}H NMR (400 MHz, CDCl$_3$)
O
H
8d
OMe
1.00
0.99
1.06
0.96
3.19
2.97
2.98
2.94
f1 (ppm)

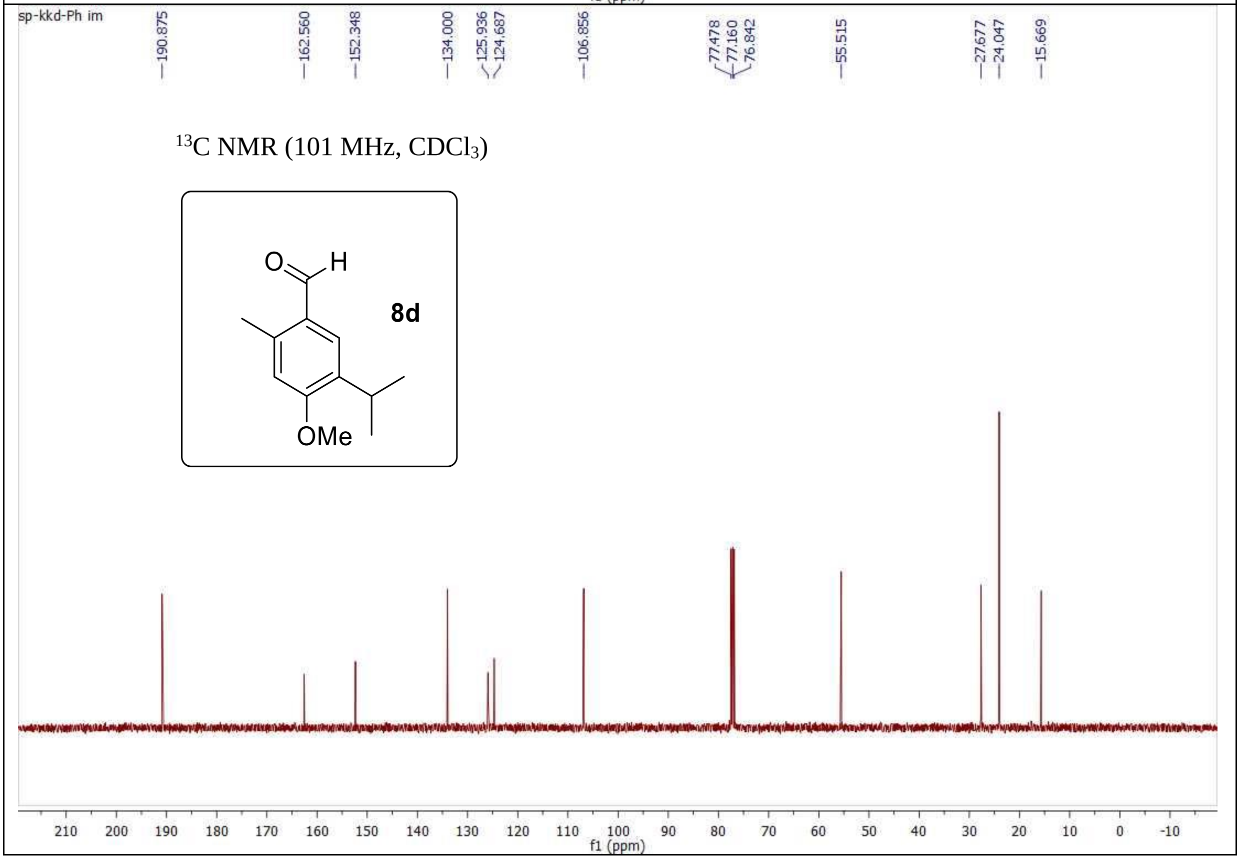
sp-kkd-Ph im
190.875
162.560
152.348
134.000
125.936
124.687
106.856
77.478
77.160
76.842
55.515
27.677
24.047
15.669
^{13}C NMR (101 MHz, CDCl$_3$)
O
H
8d
OMe
f1 (ppm)

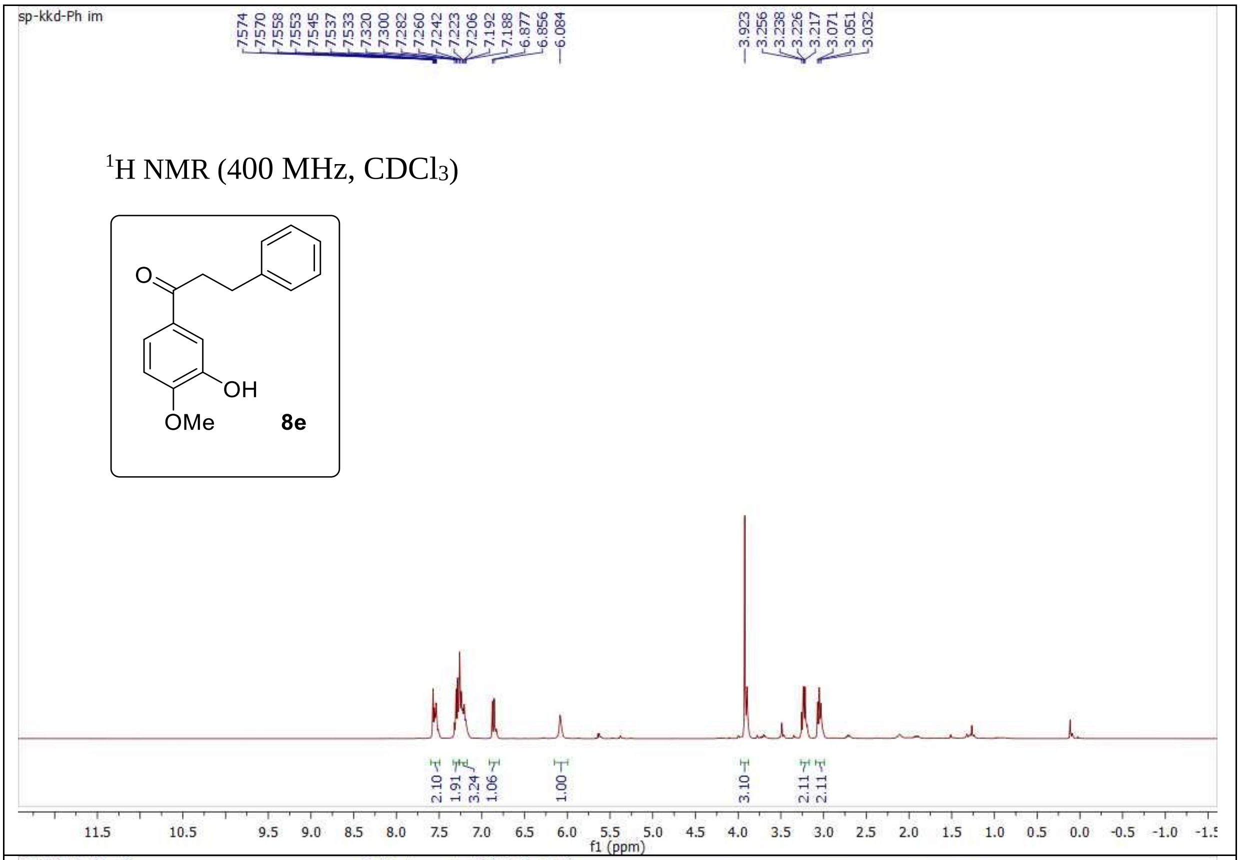
sp-kkd-Ph im
7.574
7.570
7.558
7.553
7.545
7.537
7.533
7.320
7.300
7.282
7.260
7.242
7.223
7.206
7.192
7.188
6.877
6.856
6.084
3.923
3.256
3.238
3.226
3.217
3.071
3.051
3.032
1H NMR (400 MHz, CDCl3)
O
OH
OMe
8e
2.10
1.91
3.24
1.06
1.00
3.10
2.11
2.11
f1 (ppm)

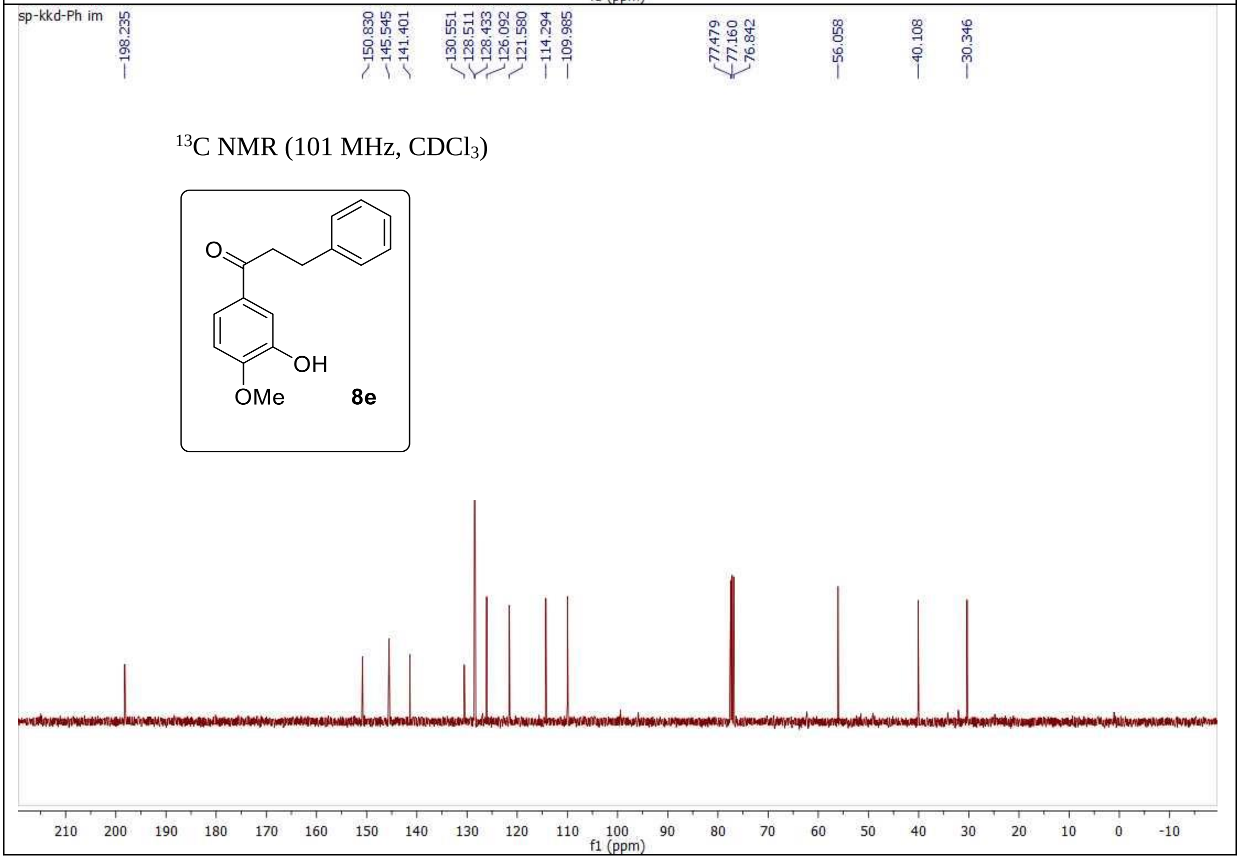
sp-kkd-Ph im
198.235
150.830
145.545
141.401
130.551
128.511
128.433
126.092
121.580
114.294
109.985
77.479
77.160
76.842
56.058
40.108
30.346
13C NMR (101 MHz, CDCl3)
O
OH
OMe
8e
f1 (ppm)

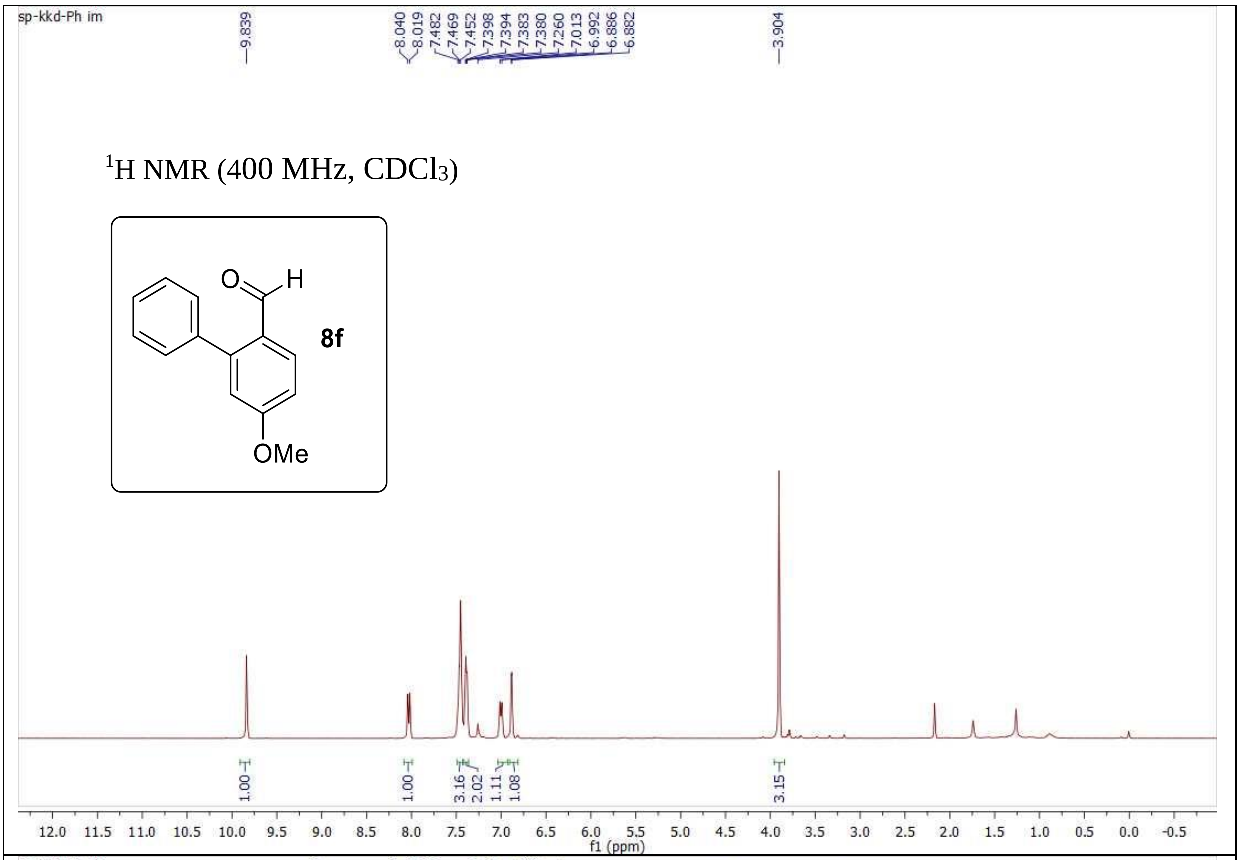
sp-kkd-Ph im
9.839
8.040
8.019
7.482
7.469
7.452
7.398
7.394
7.383
7.380
7.260
7.013
6.992
6.886
6.882
3.904
¹H NMR (400 MHz, CDCl3)
O
H
8f
OMe
1.00
1.00
3.16
2.02
1.11
1.08
3.15
f1 (ppm)

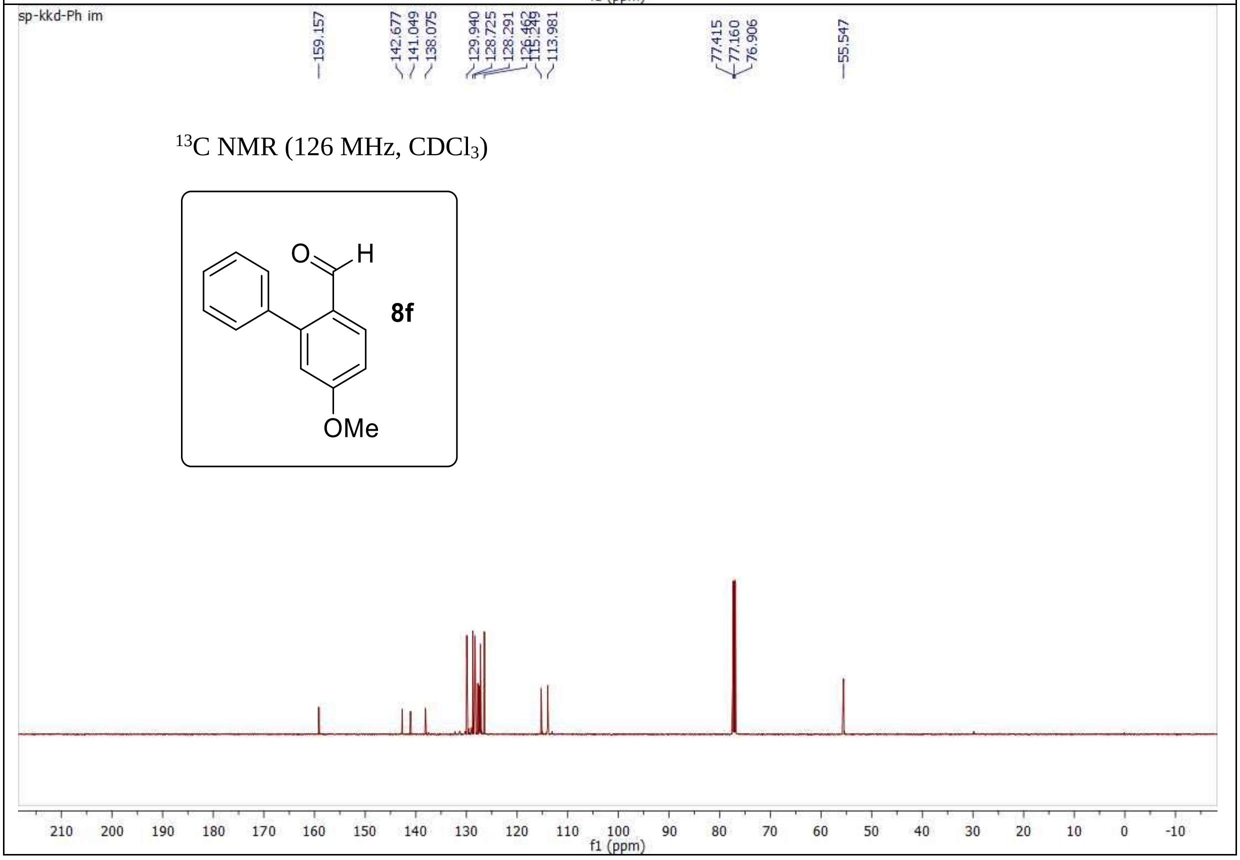
sp-kkd-Ph im
159.157
142.677
141.049
138.075
129.940
128.725
128.291
113.981
77.415
77.160
76.906
55.547
¹³C NMR (126 MHz, CDCl3)
O
H
8f
OMe
f1 (ppm)

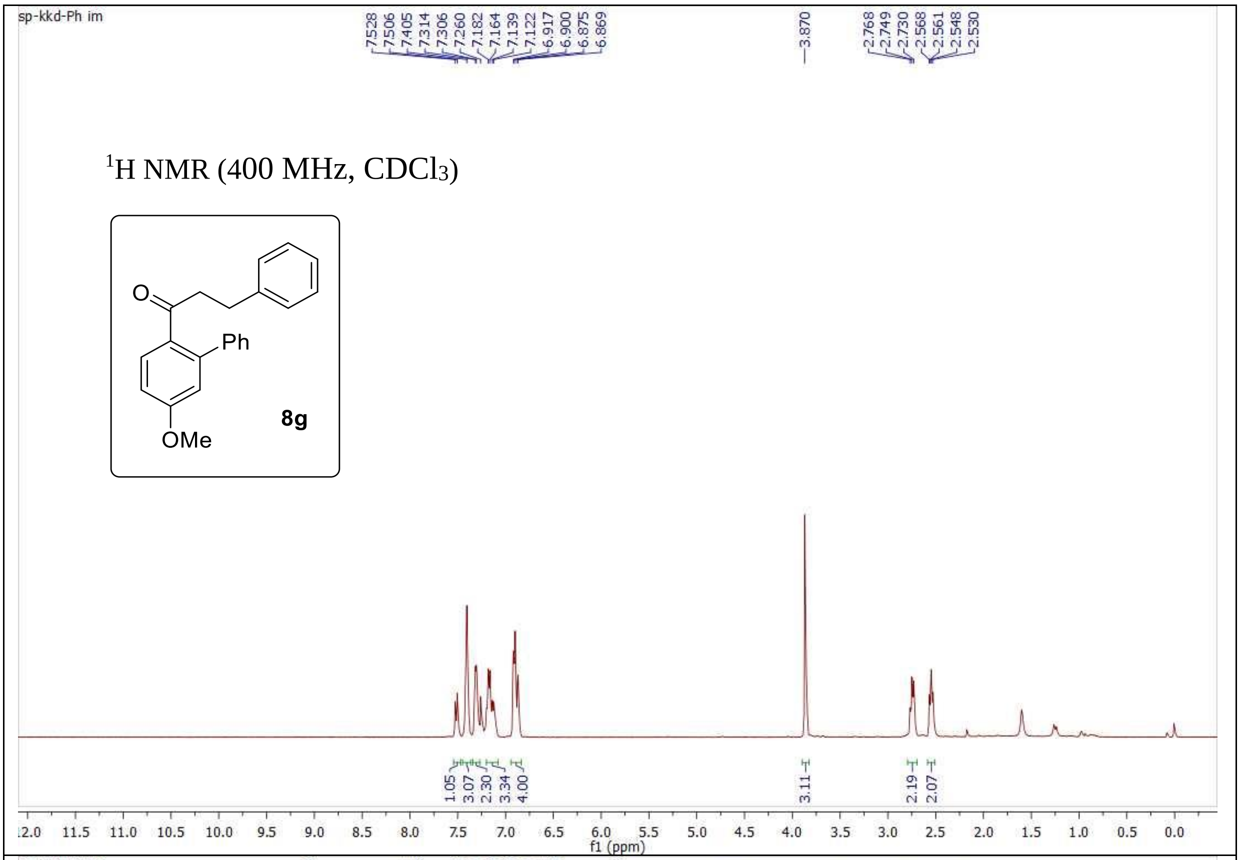
sp-kkd-Ph im
7.528
7.506
7.405
7.314
7.306
7.260
7.182
7.164
7.139
7.122
6.917
6.900
6.875
6.869
3.870
2.768
2.749
2.730
2.568
2.561
2.548
2.530
1H NMR (400 MHz, CDCl3)
O
Ph
OMe
8g
1.05
3.07
2.30
3.34
4.00
3.11
2.19
2.07
f1 (ppm)

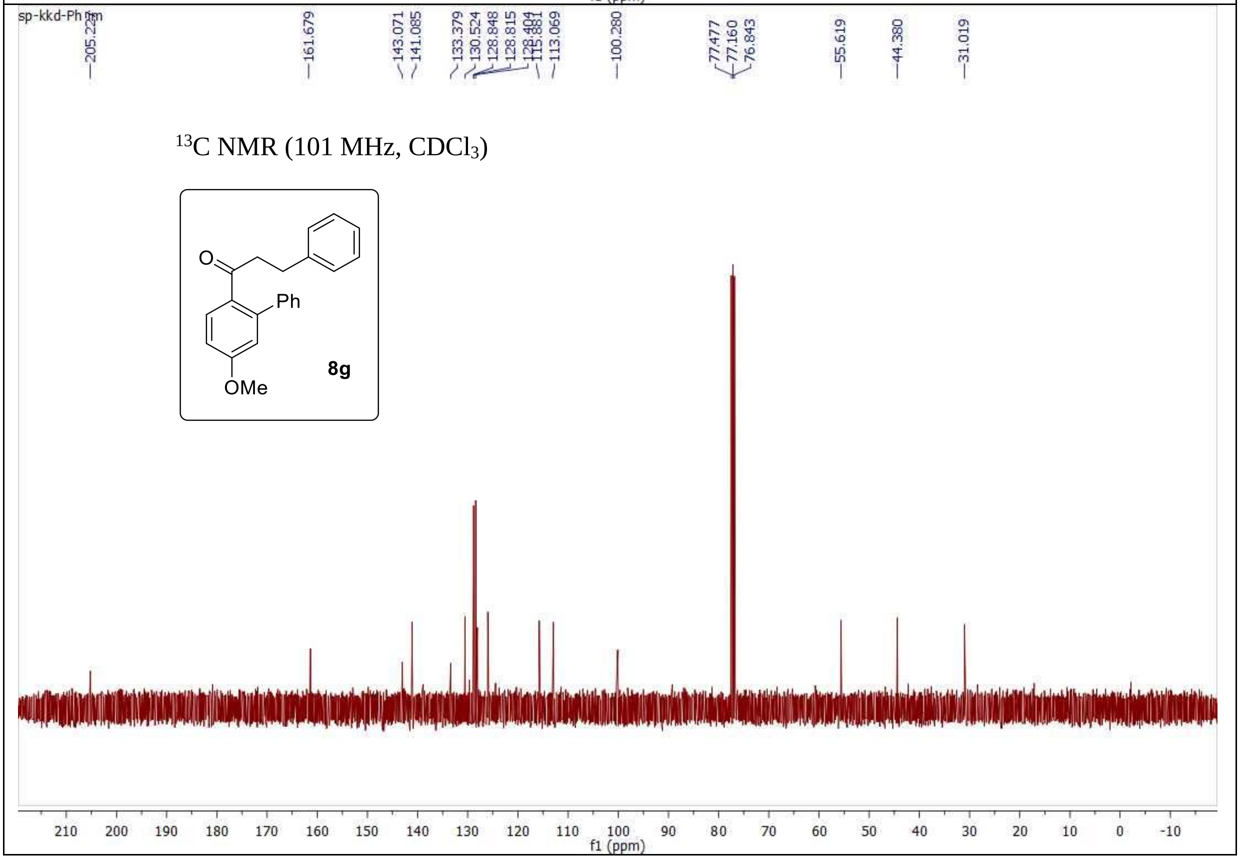
sp-kkd-Ph im
161.679
143.071
141.085
133.379
130.524
128.848
128.815
113.069
100.280
77.477
77.160
76.843
55.619
44.380
31.019
13C NMR (101 MHz, CDCl3)
O
Ph
OMe
8g
f1 (ppm)

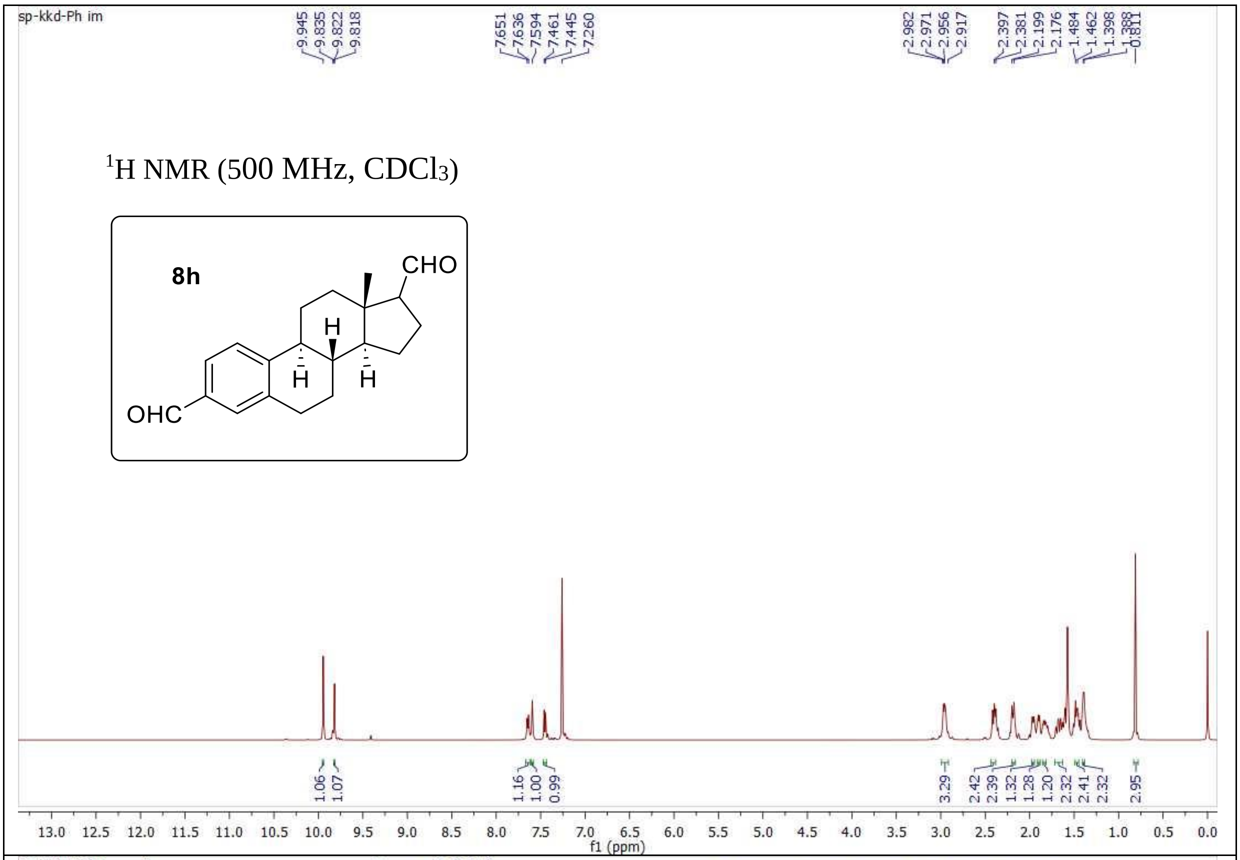
sp-kkd-Ph im
9.945
9.835
9.822
9.818
7.651
7.636
7.594
7.461
7.445
7.260
2.982
2.971
2.956
2.917
2.397
2.381
2.199
2.176
1.484
1.462
1.398
0.811
¹H NMR (500 MHz, CDCl3)
8h
CHO
H
H
H
OHC
1.06
1.07
1.16
1.00
0.99
3.29
2.42
2.39
1.32
1.28
1.20
2.32
2.41
2.32
2.95
13.0 12.5 12.0 11.5 11.0 10.5 10.0 9.5 9.0 8.5 8.0 7.5 7.0 6.5 6.0 5.5 5.0 4.5 4.0 3.5 3.0 2.5 2.0 1.5 1.0 0.5 0.0
f1 (ppm)

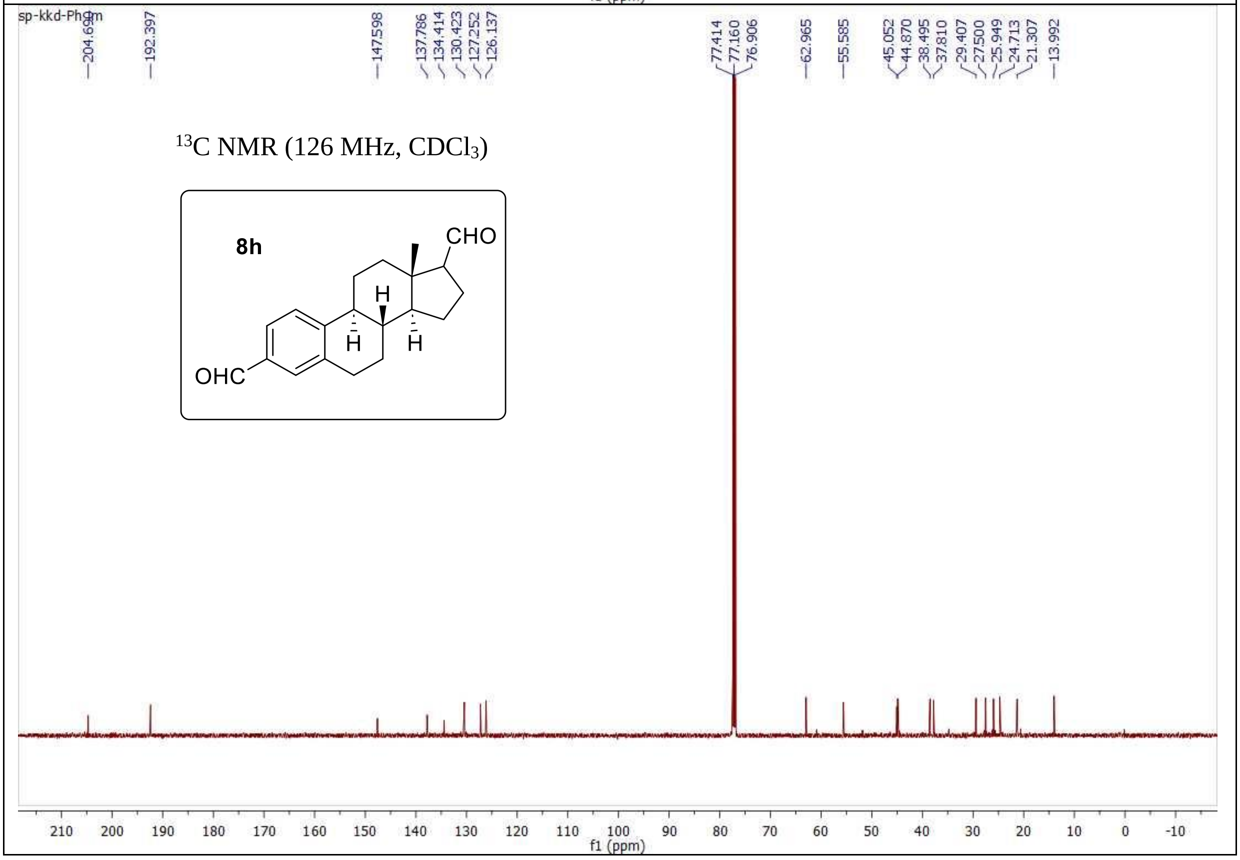
sp-kkd-Ph im
192.397
147.598
137.786
134.414
130.423
127.252
126.137
77.414
77.160
76.906
62.965
55.585
45.052
44.870
38.495
37.810
29.407
27.500
25.949
24.713
21.307
13.992
¹³C NMR (126 MHz, CDCl3)
8h
CHO
H
H
H
OHC
210 200 190 180 170 160 150 140 130 120 110 100 90 80 70 60 50 40 30 20 10 0 -10
f1 (ppm)

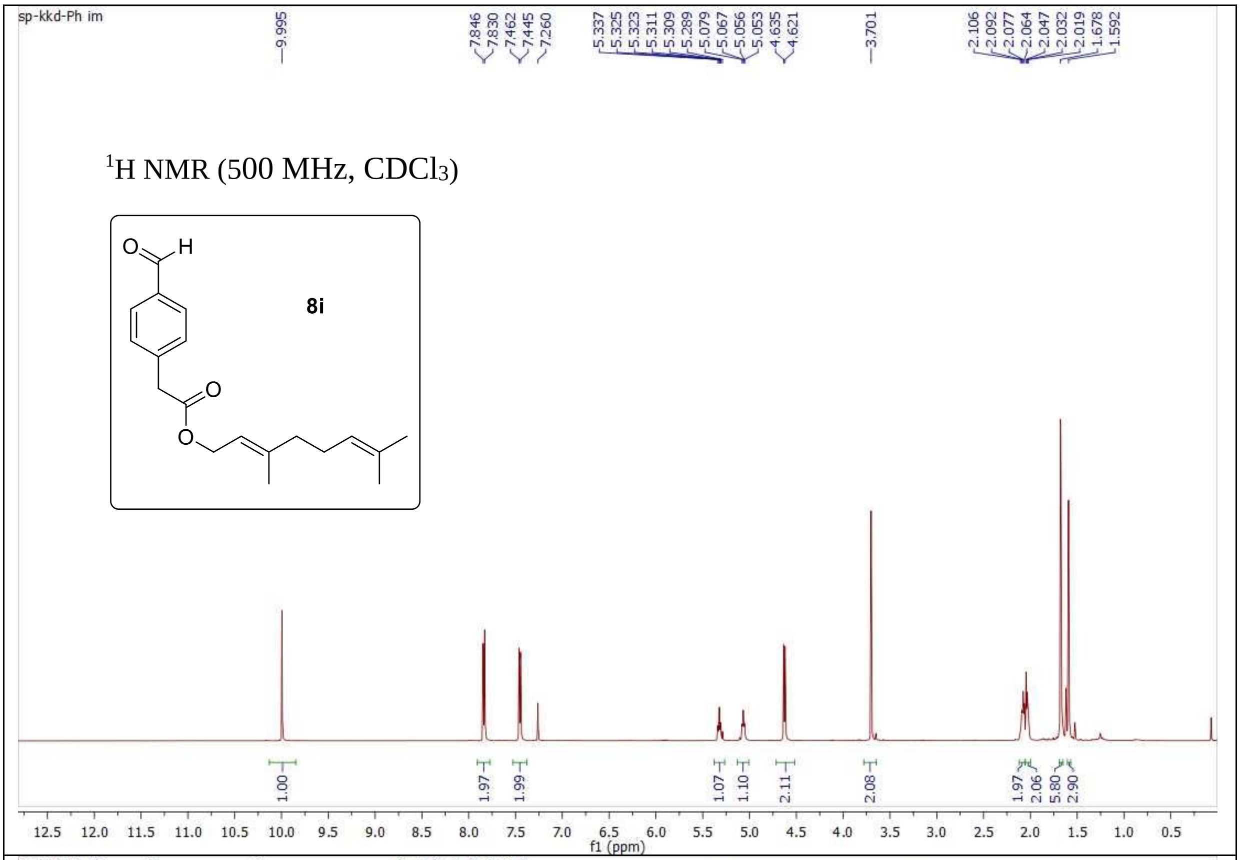
sp-kkd-Ph im
9.995
7.846
7.830
7.462
7.445
7.260
5.337
5.325
5.323
5.311
5.309
5.289
5.079
5.067
5.056
5.053
4.635
4.621
3.701
2.106
2.092
2.077
2.064
2.047
2.032
2.019
1.678
1.592
1H NMR (500 MHz, CDCl3)
8i
1.00
1.97
1.99
1.07
1.10
2.11
2.08
1.97
2.06
5.80
2.90
f1 (ppm)

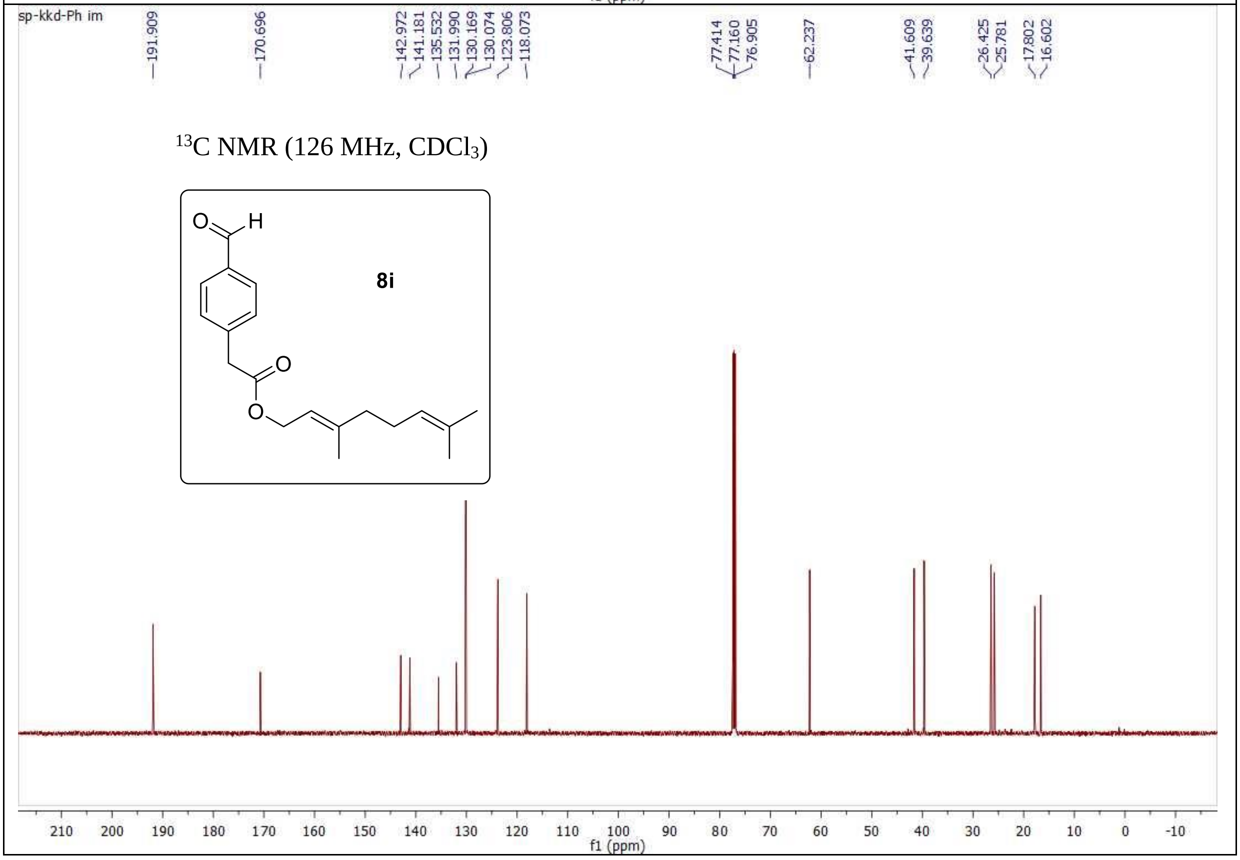
sp-kkd-Ph im
191.909
170.696
142.972
141.181
135.532
131.990
130.169
130.074
123.806
118.073
77.414
77.160
76.905
62.237
41.609
39.639
26.425
25.781
17.802
16.602
13C NMR (126 MHz, CDCl3)
8i
f1 (ppm)

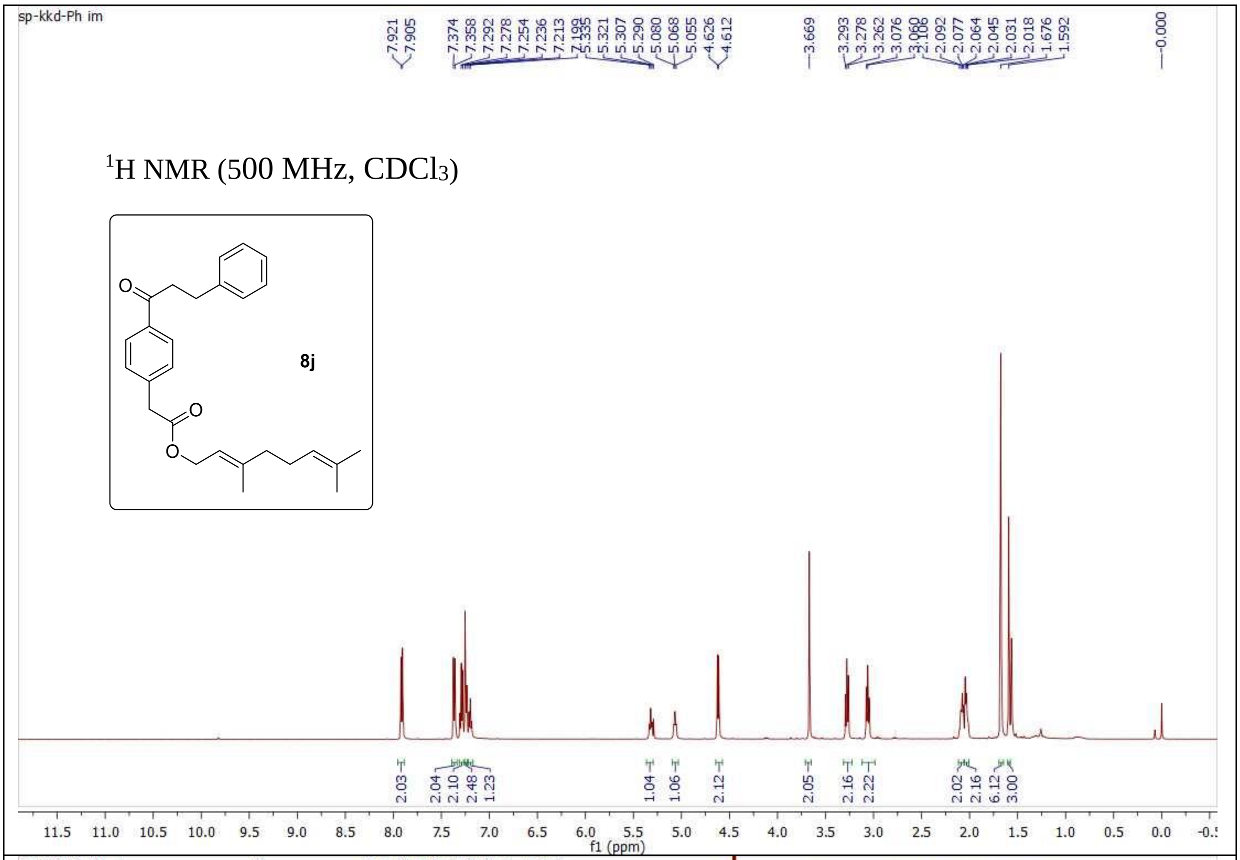
sp-kkd-Ph im
7.921
7.905
7.374
7.358
7.292
7.278
7.254
7.236
7.213
5.335
5.321
5.307
5.290
5.080
5.068
5.055
4.626
4.612
3.669
3.293
3.278
3.262
3.076
2.092
2.077
2.064
2.045
2.031
2.018
1.676
1.592
0.000
1H NMR (500 MHz, CDCl3)
O
8j
O
O
2.03
2.04
2.10
2.48
1.23
1.04
1.06
2.12
2.05
2.16
2.22
2.02
2.16
6.12
3.00
f1 (ppm)

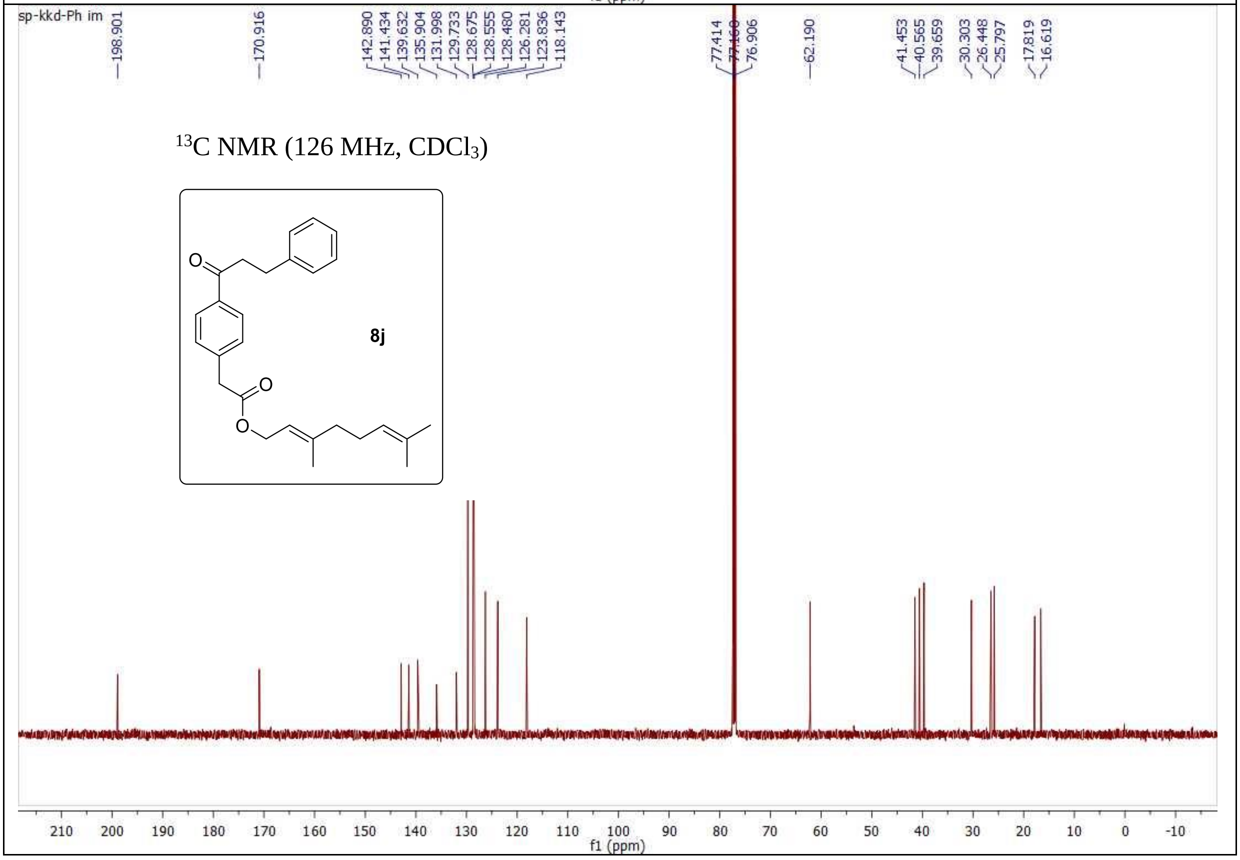
sp-kkd-Ph im
198.901
170.916
142.890
141.434
139.632
135.904
131.998
129.733
128.675
128.555
128.480
126.281
123.836
118.143
77.414
76.906
62.190
41.453
40.565
39.659
30.303
26.448
25.797
17.819
16.619
13C NMR (126 MHz, CDCl3)
O
8j
O
O
f1 (ppm)

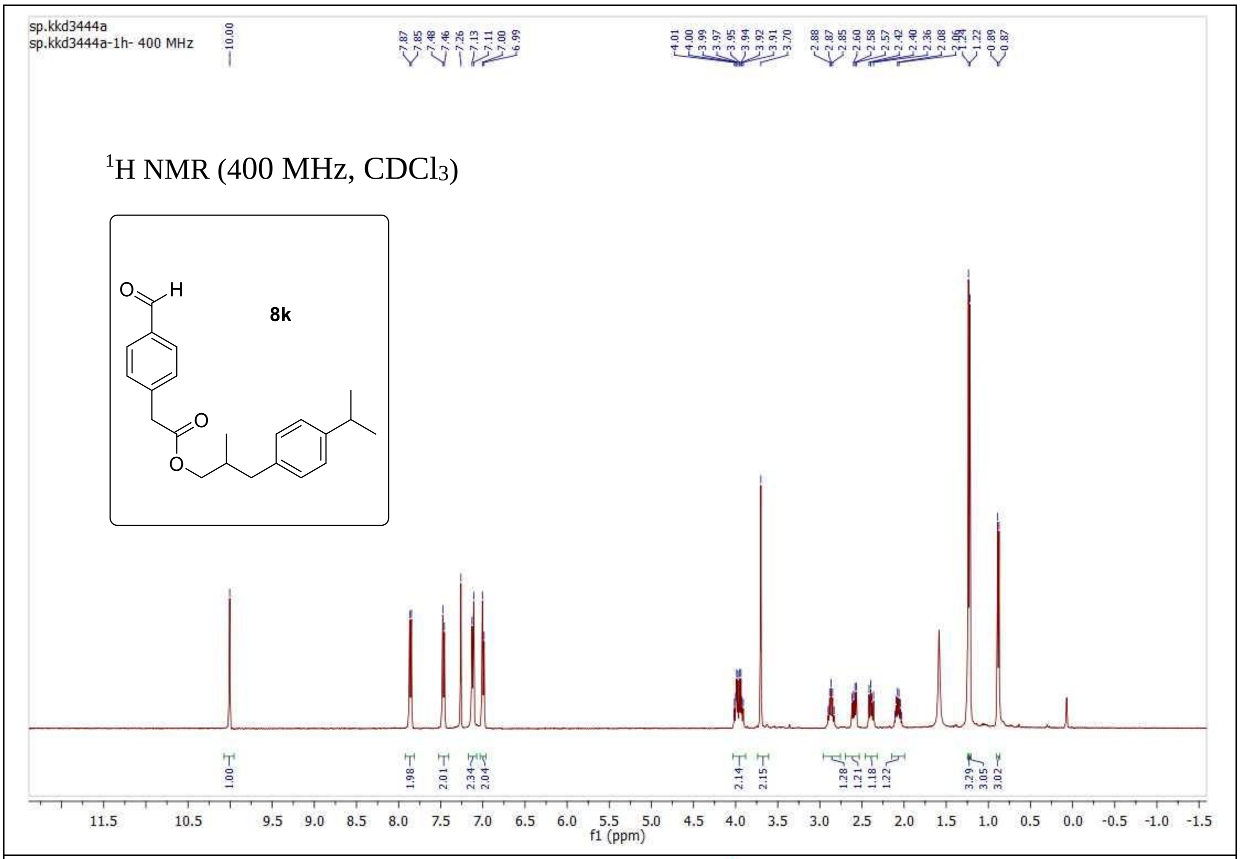
sp.kkd3444a
sp.kkd3444a-1h- 400 MHz
^{1}H NMR (400 MHz, $CDCl_3$)
8k
f1 (ppm)

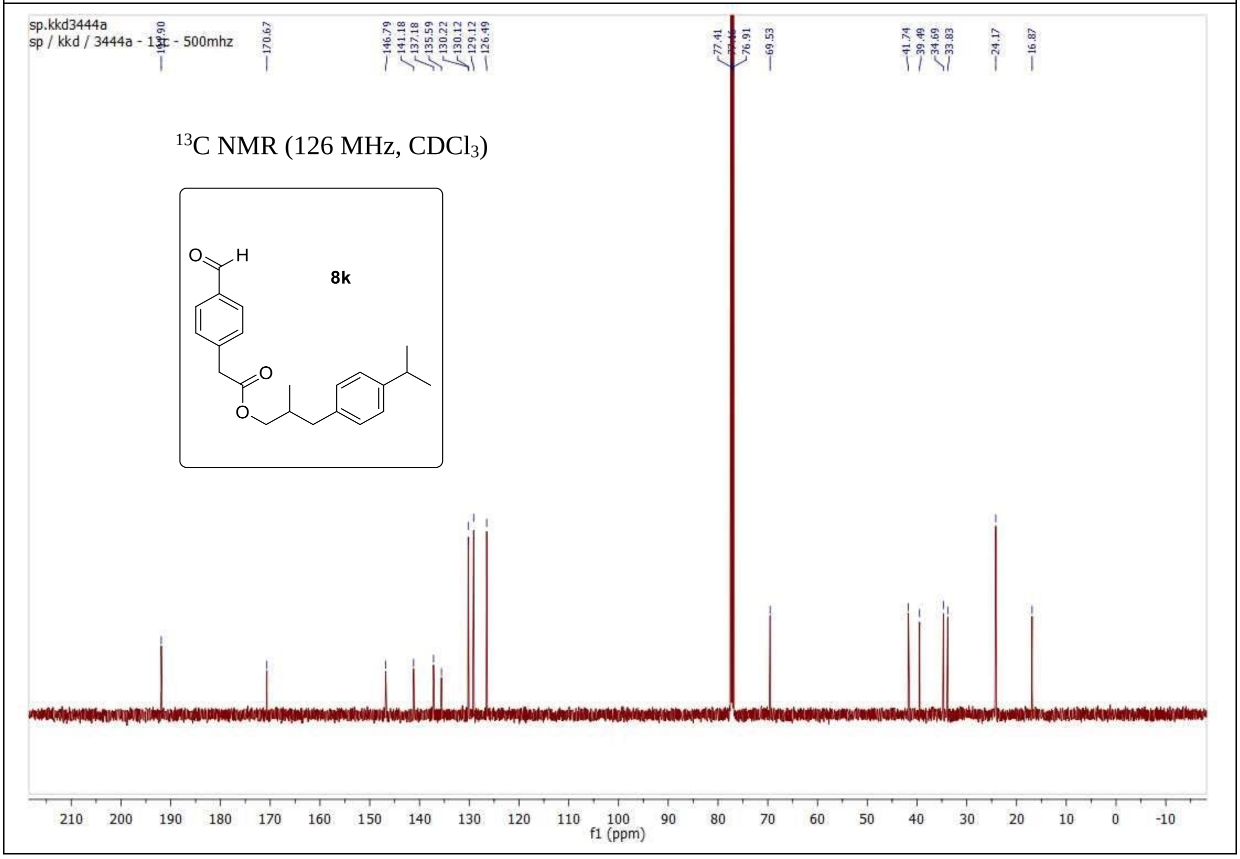
sp.kkd3444a
^{13}C NMR (126 MHz, $CDCl_3$)
8k
f1 (ppm)

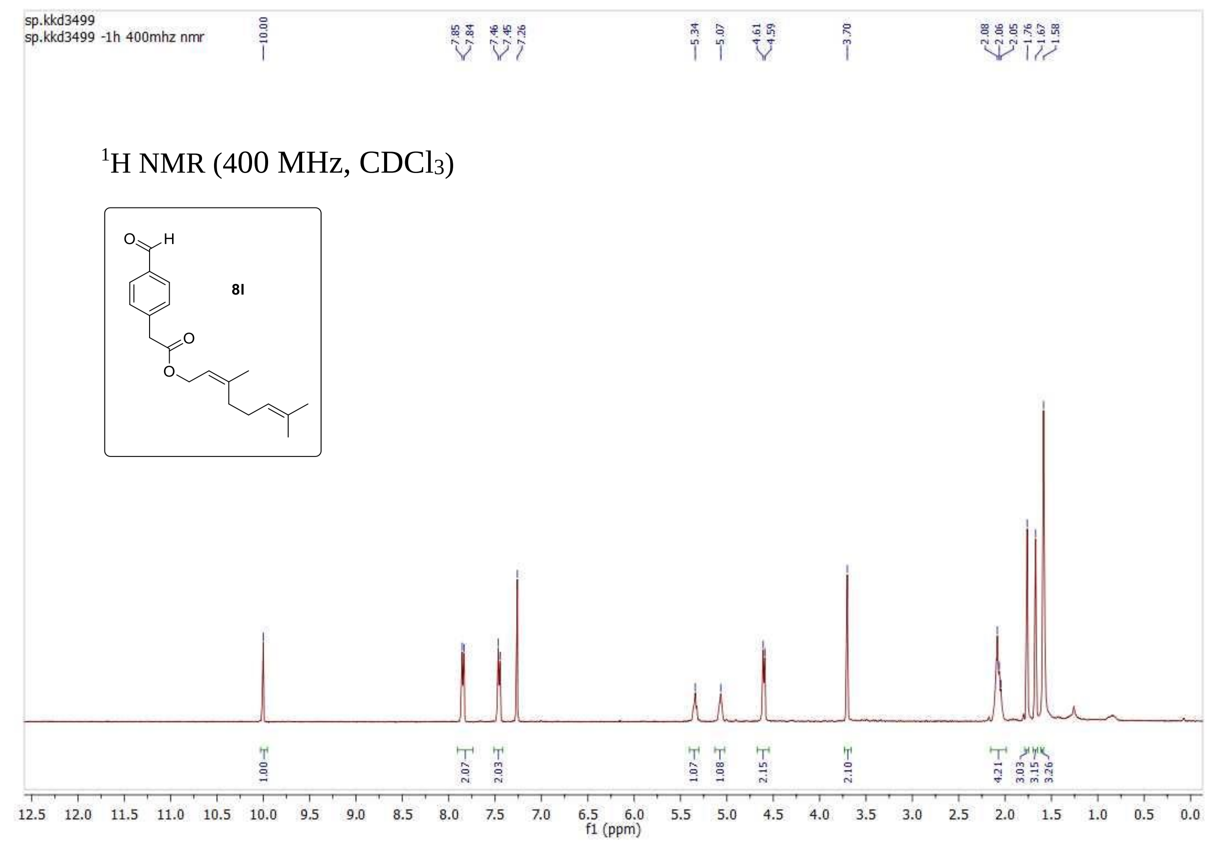
sp.kkd3499
sp.kkd3499 -1h 400mhz nmr
10.00
7.85
7.84
7.46
7.45
7.26
5.34
5.07
4.61
4.59
3.70
2.08
2.06
2.05
1.76
1.67
1.58
1H NMR (400 MHz, CDCl3)
O
H
8l
O
O
1.00
2.07
2.03
1.07
1.08
2.15
2.10
4.21
3.03
3.15
3.26
f1 (ppm)

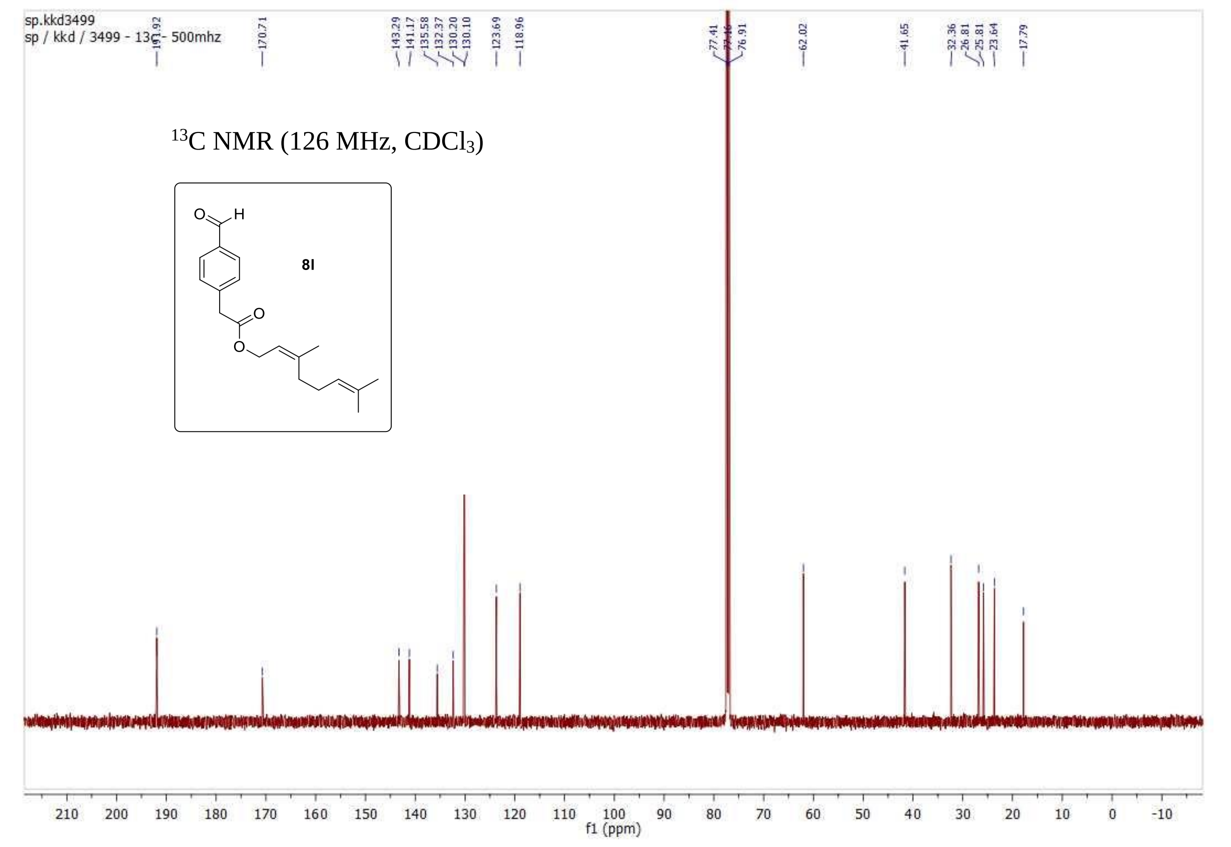
sp.kkd3499
sp / kkd / 3499 - 13c - 500mhz
191.92
170.71
143.29
141.17
135.58
132.37
130.20
130.10
123.69
118.96
77.41
77.16
76.91
62.02
41.65
32.36
26.81
25.81
23.64
17.79
13C NMR (126 MHz, CDCl3)
O
H
8l
O
O
f1 (ppm)

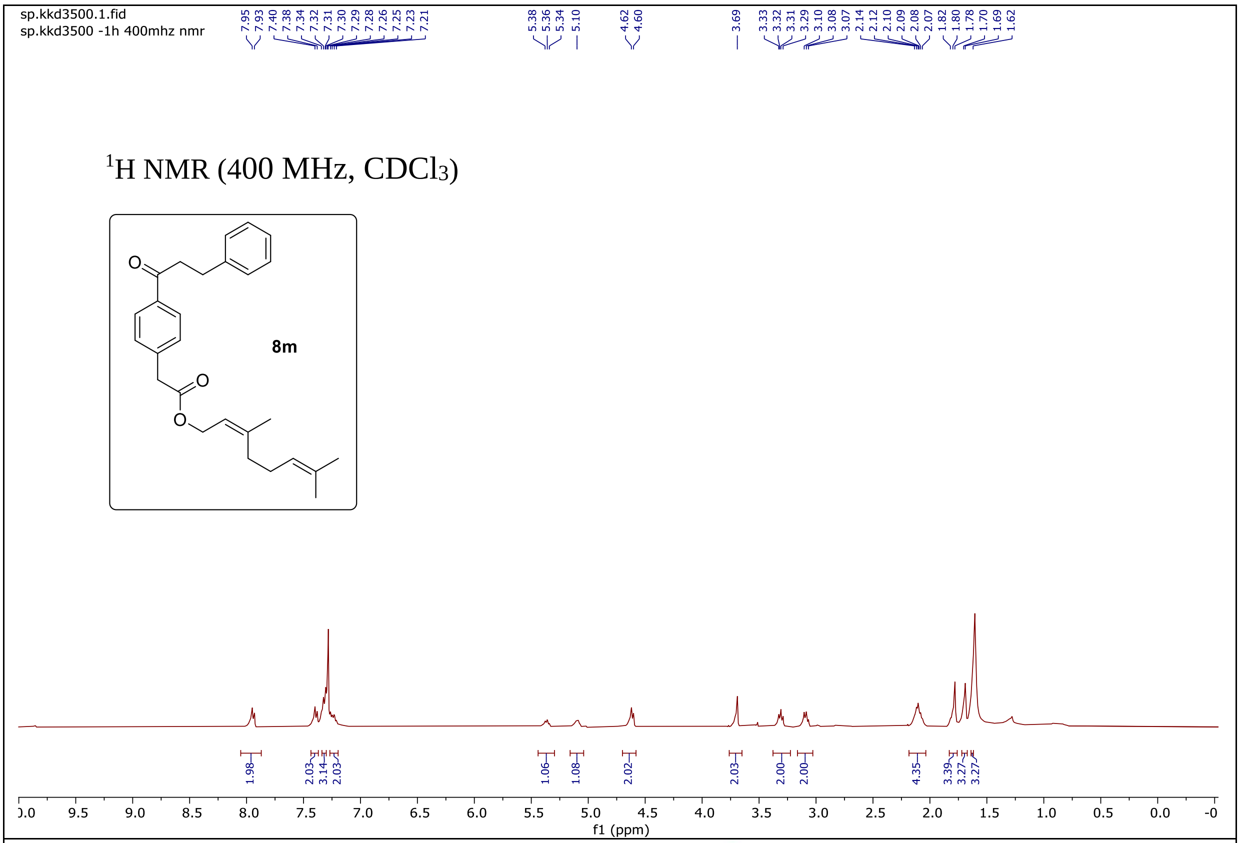

sp.kkd3500.1.fid
sp.kkd3500 -1h 400mhz nmr
1H NMR (400 MHz, CDCl3)
8m
f1 (ppm)

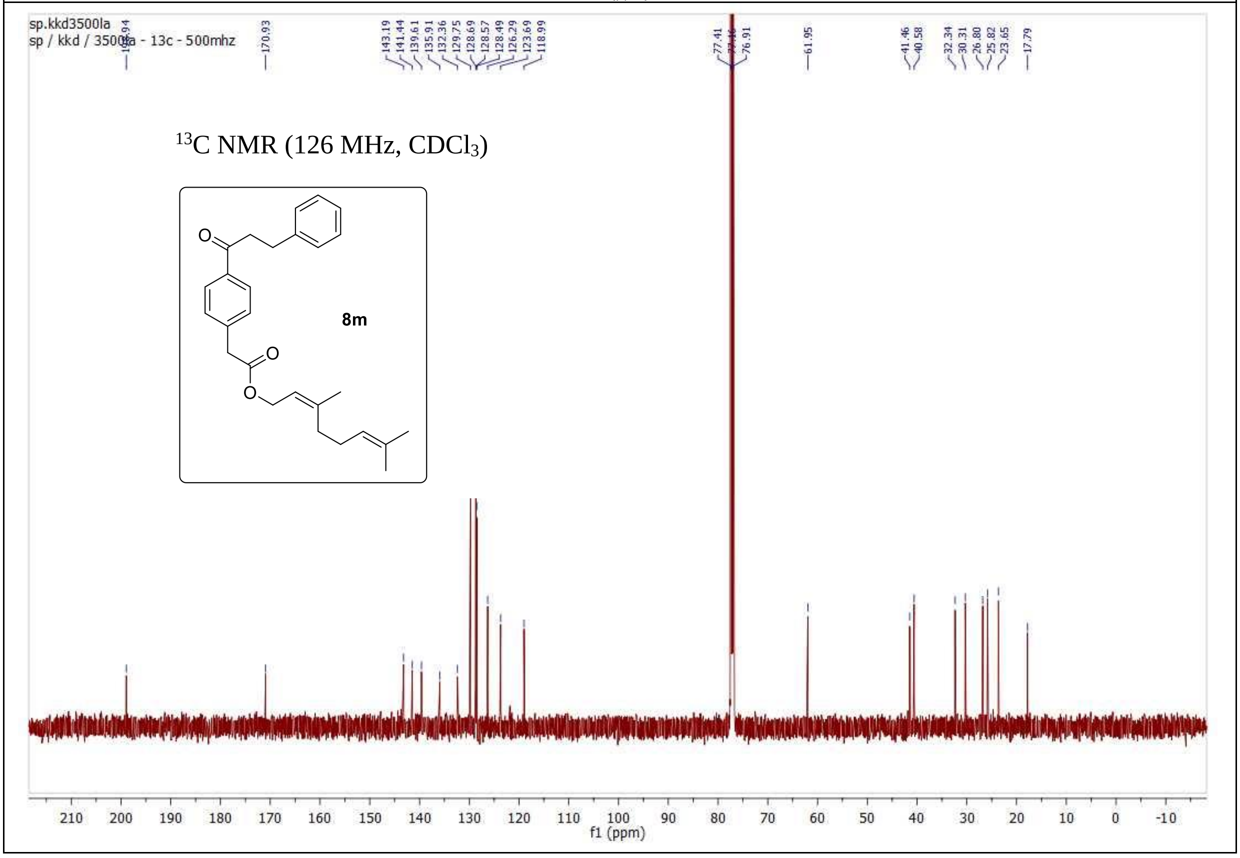

sp.kkd3500la
13C NMR (126 MHz, CDCl3)
8m
f1 (ppm)

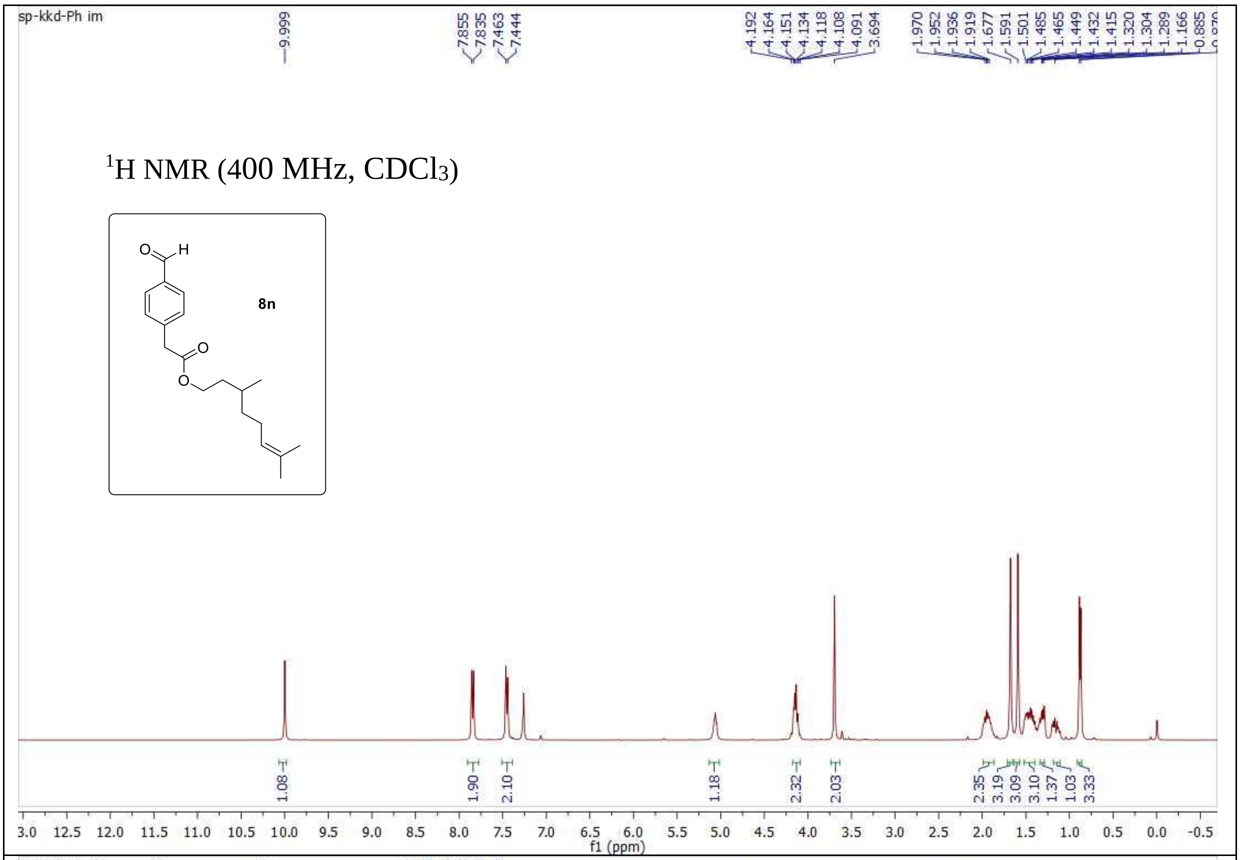
sp-kkd-Ph im
^{1}H NMR (400 MHz, CDCl$_3$)
8n
f1 (ppm)

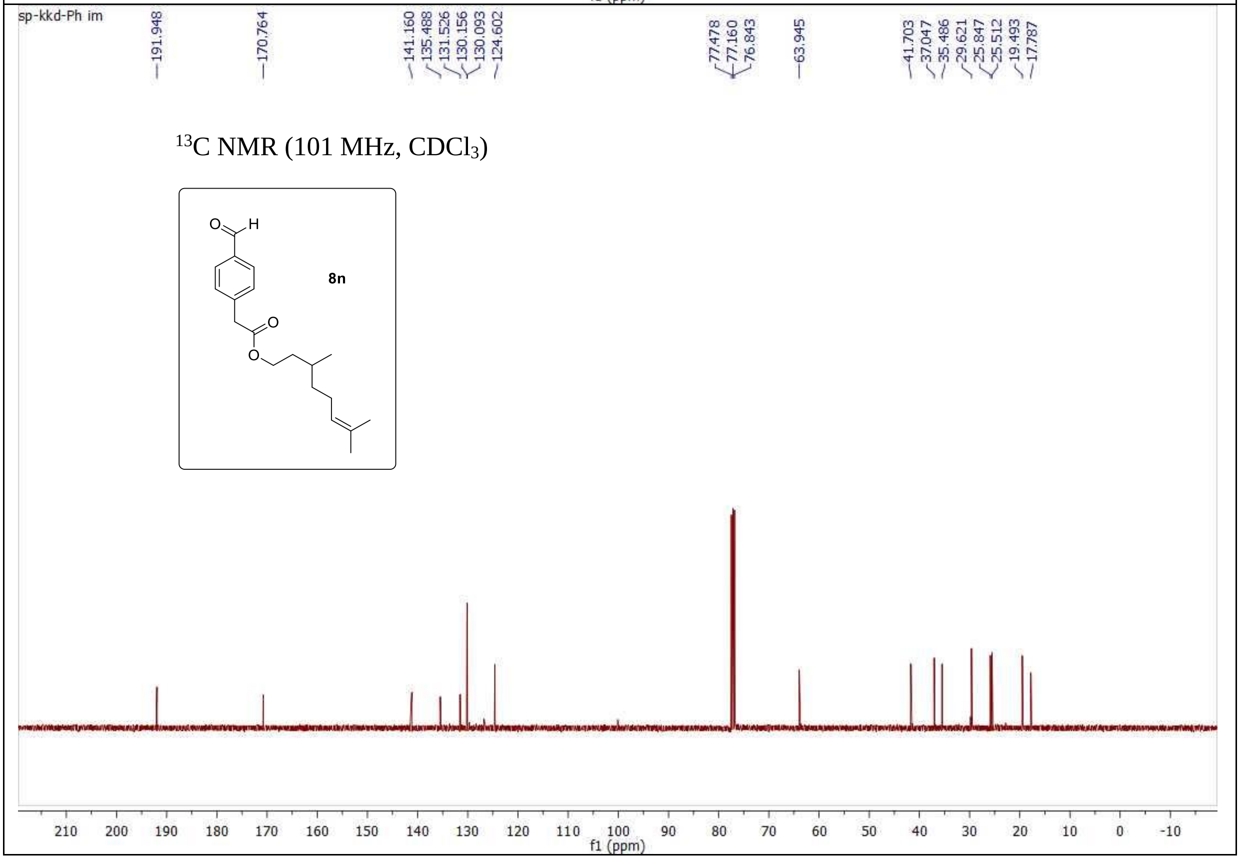
sp-kkd-Ph im
^{13}C NMR (101 MHz, CDCl$_3$)
8n
f1 (ppm)

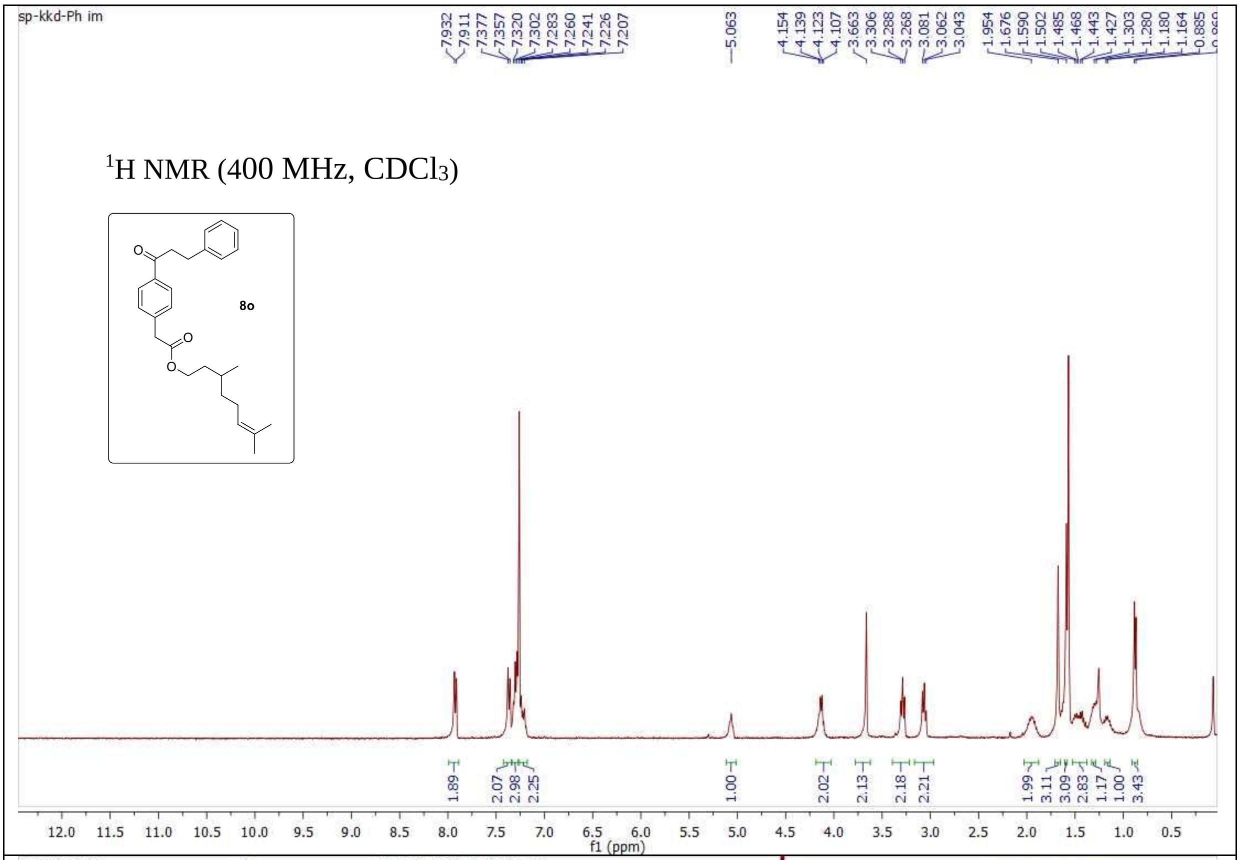
sp-kkd-Ph im
1H NMR (400 MHz, CDCl3)
8o
f1 (ppm)

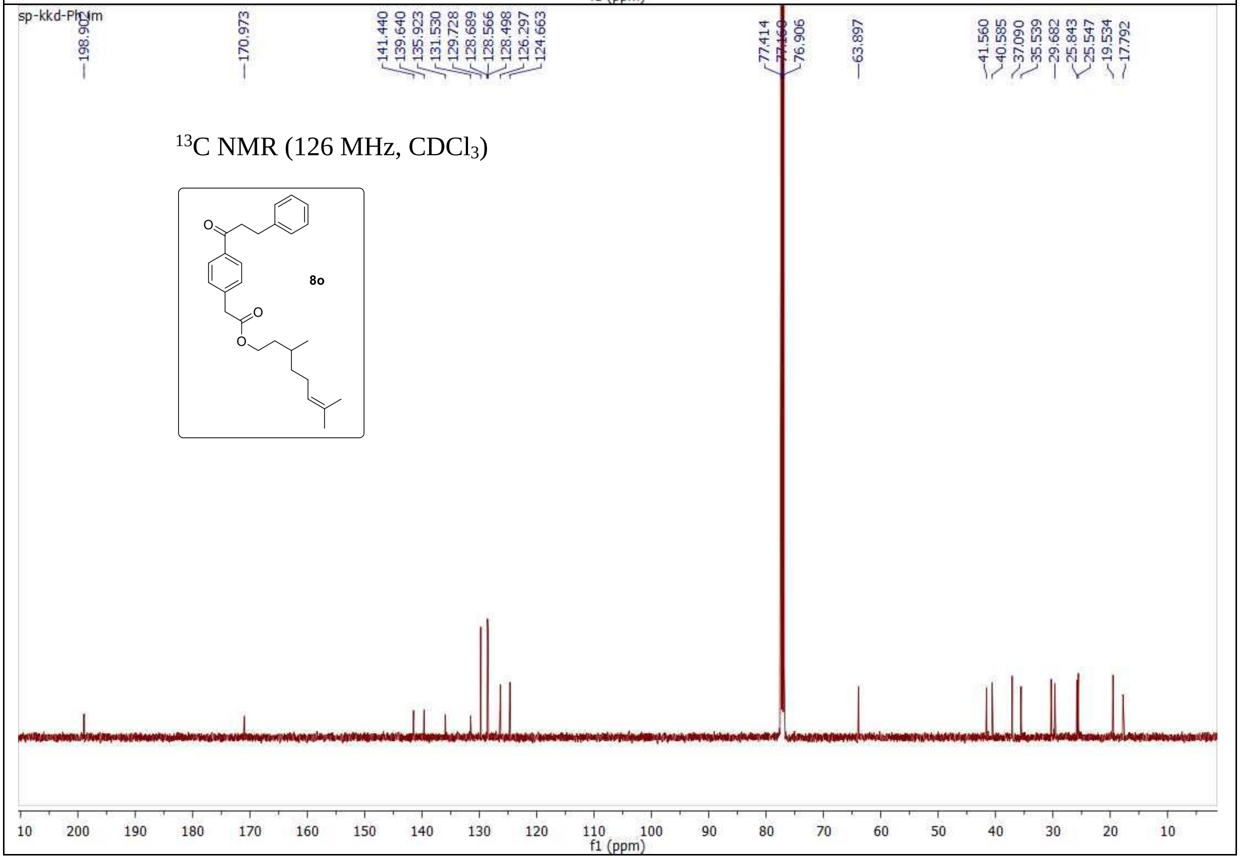
sp-kkd-Ph im
13C NMR (126 MHz, CDCl3)
8o
f1 (ppm)

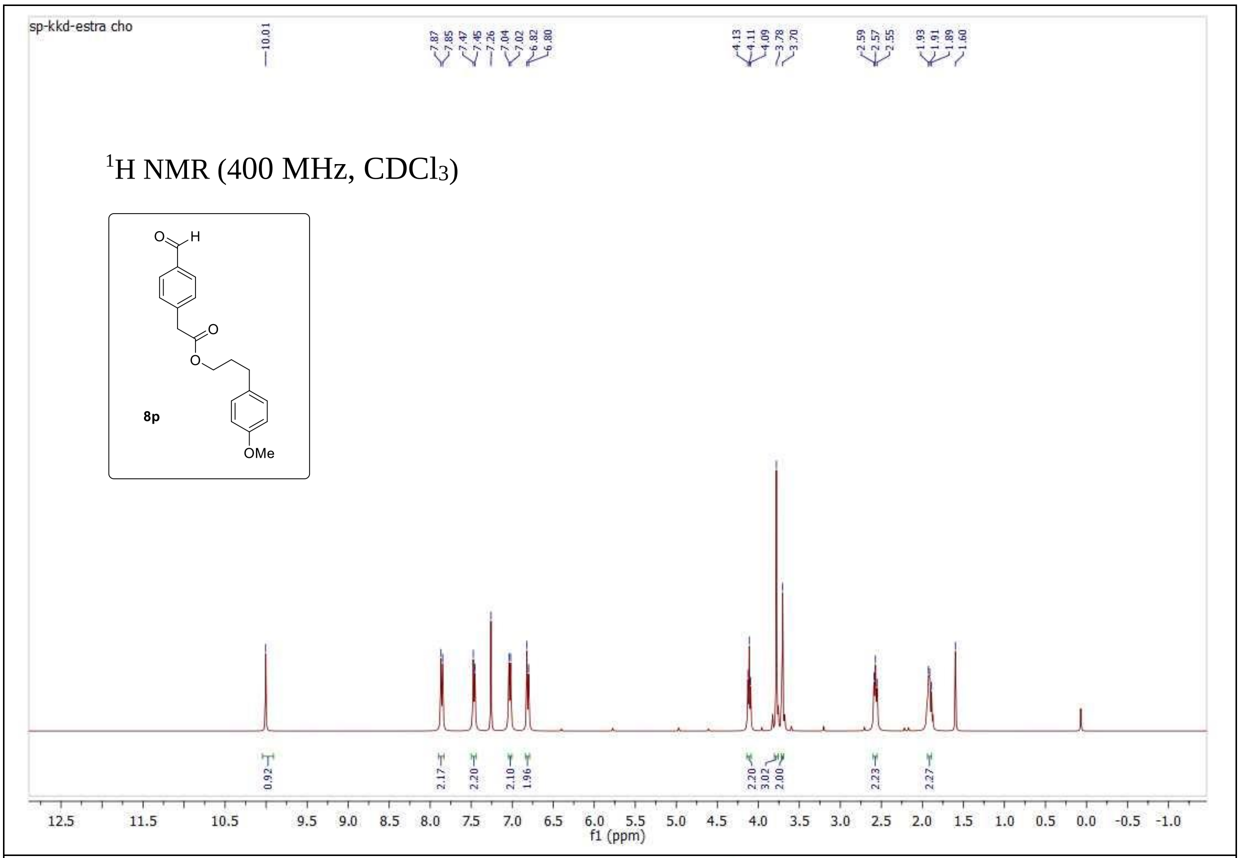
sp-kkd-estra cho
1H NMR (400 MHz, CDCl3)
8p
OMe
f1 (ppm)

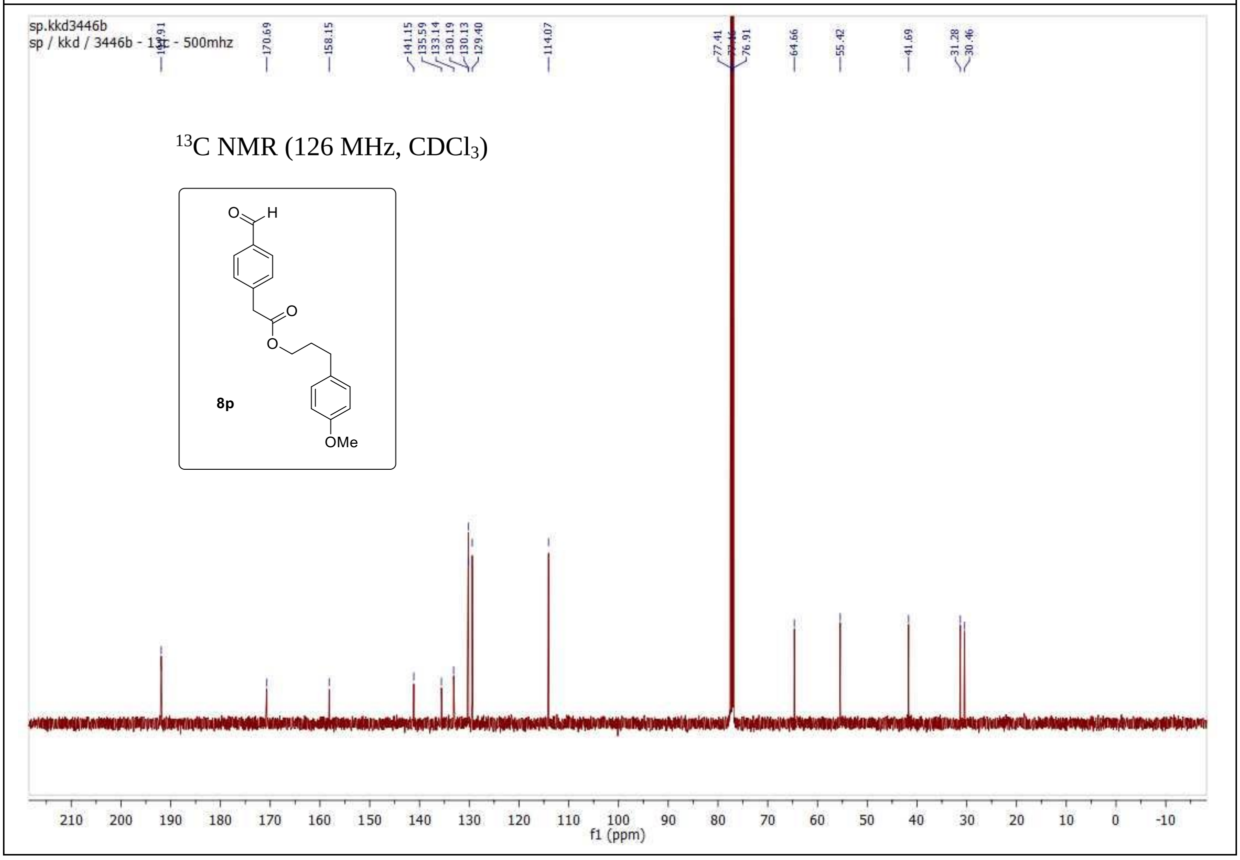
sp.kkd3446b
13C NMR (126 MHz, CDCl3)
8p
OMe
f1 (ppm)

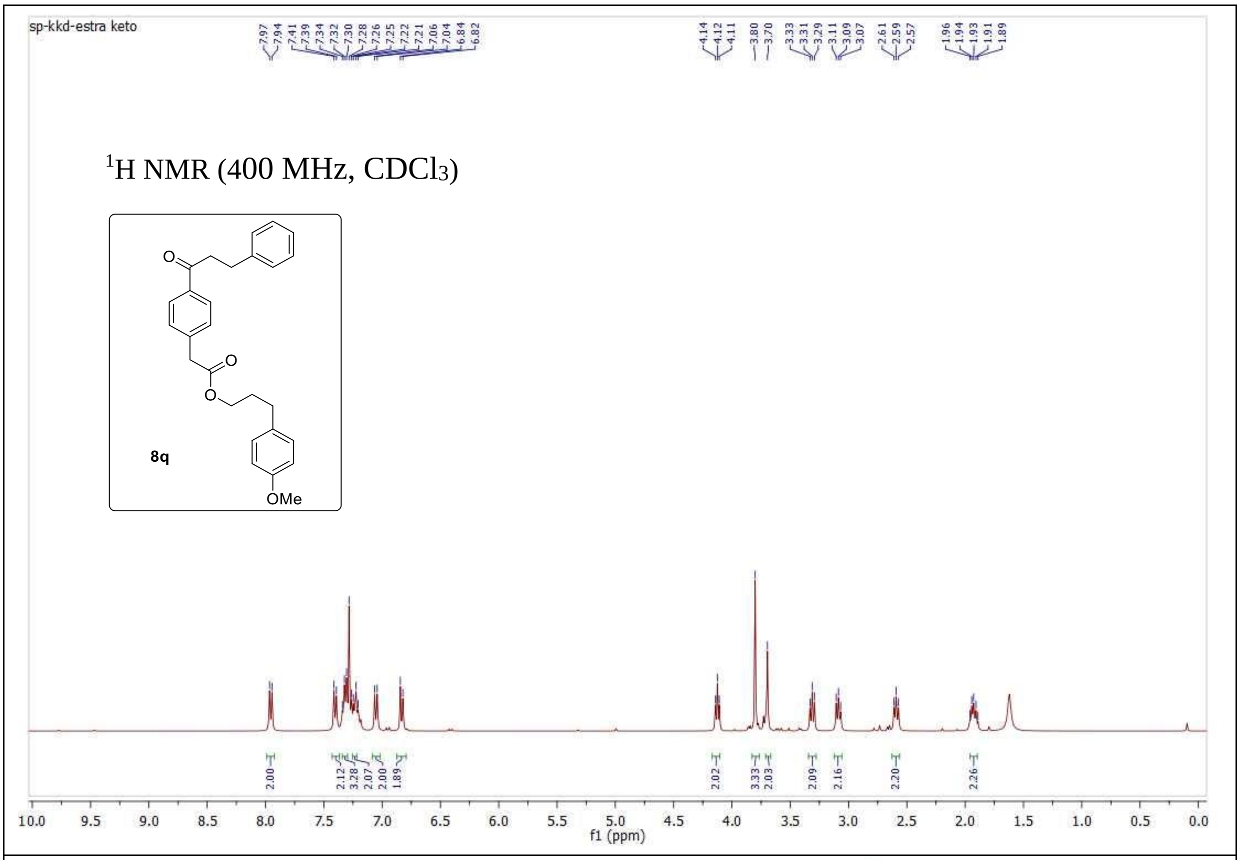
sp-kkd-estra keto
7.97 7.94 7.41 7.39 7.34 7.32 7.30 7.28 7.26 7.25 7.22 7.21 7.06 7.04 6.84 6.82
4.14 4.12 4.11 3.80 3.70 3.33 3.31 3.29 3.11 3.09 3.07 2.61 2.59 2.57 1.96 1.94 1.93 1.91 1.89
1H NMR (400 MHz, CDCl3)
8q
OMe
2.00 2.12 3.28 2.07 2.00 1.89 2.02 3.33 2.03 2.09 2.16 2.20 2.26
f1 (ppm)

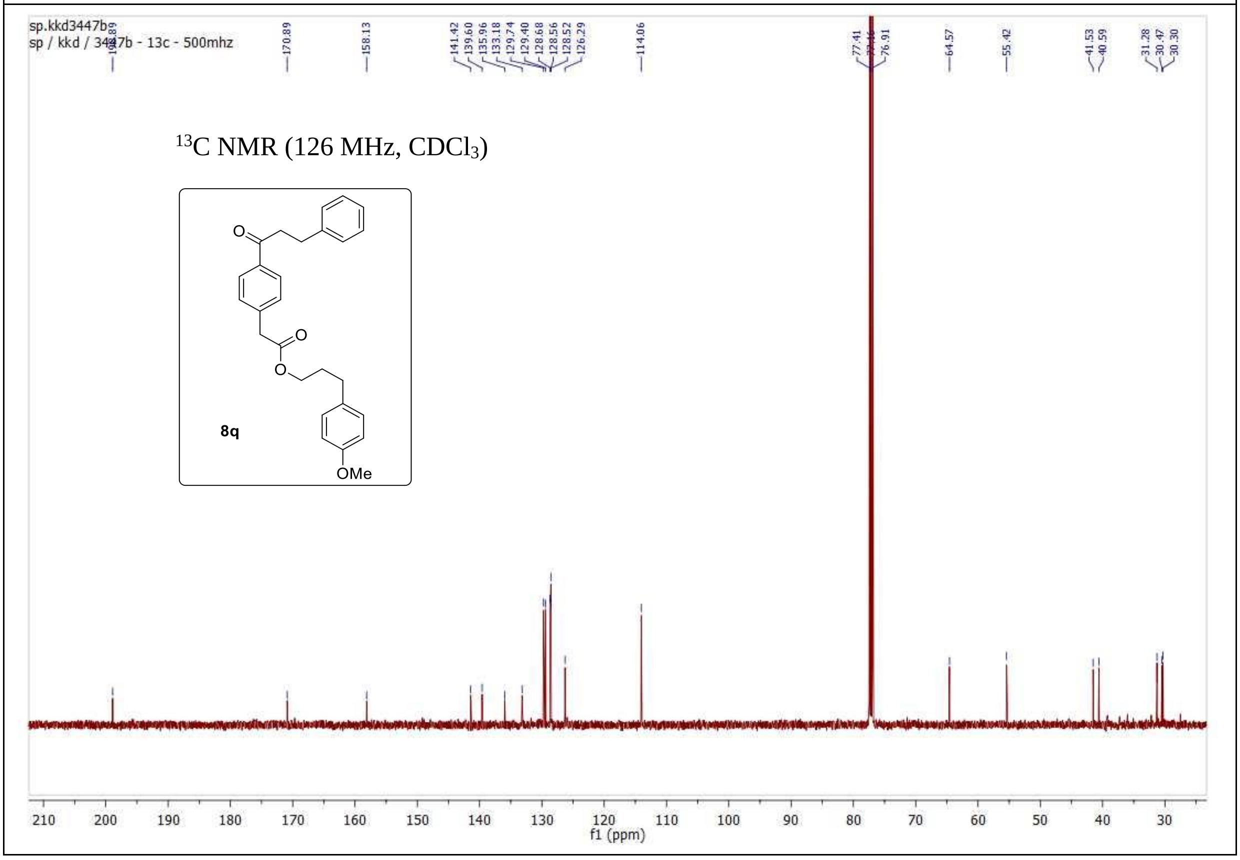
sp.kkd3447b
sp / kkd / 3447b - 13c - 500mhz
170.89 158.13 141.42 139.60 135.96 133.18 129.74 129.40 128.68 128.56 128.52 126.29 114.06 77.41 76.91 64.57 55.42 41.53 40.59 31.28 30.47 30.30
13C NMR (126 MHz, CDCl3)
8q
OMe
f1 (ppm)

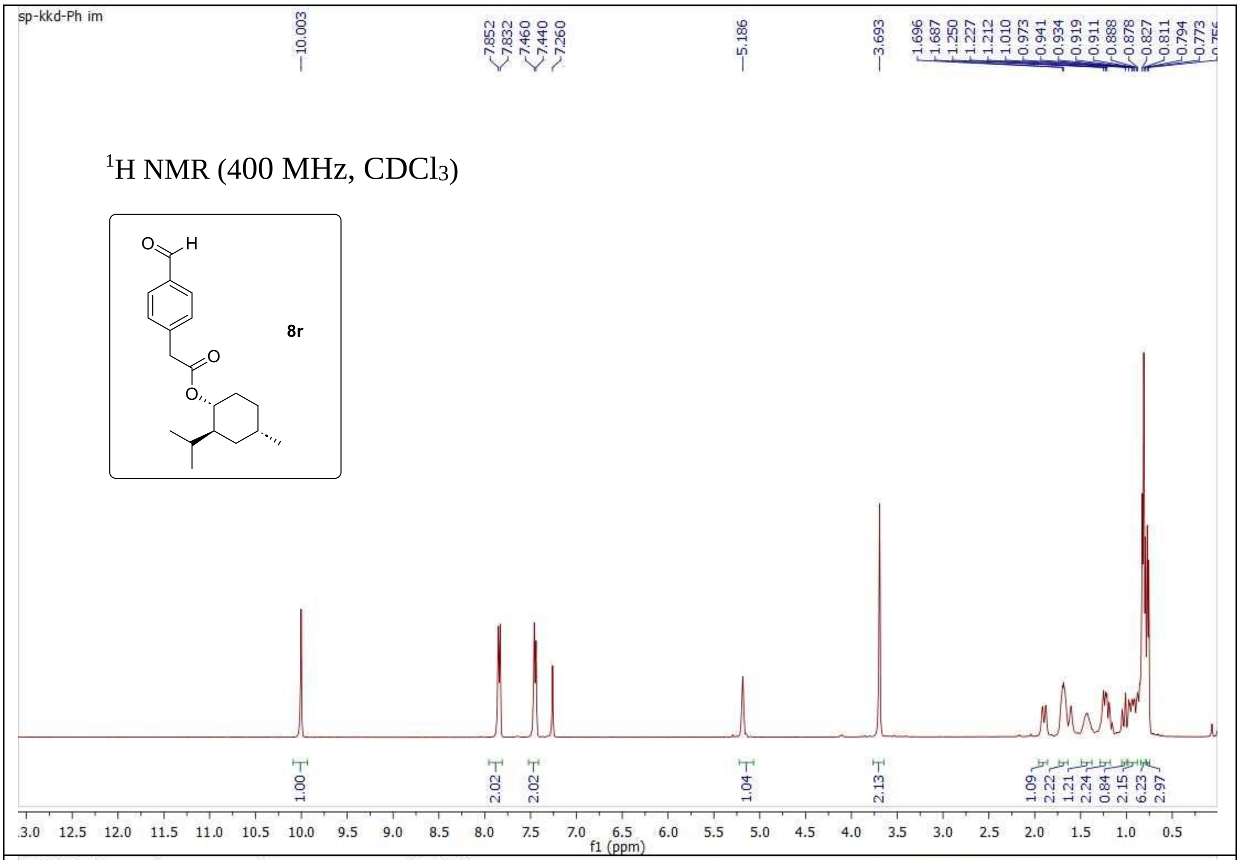
sp-kkd-Ph im
1H NMR (400 MHz, CDCl3)
8r
f1 (ppm)

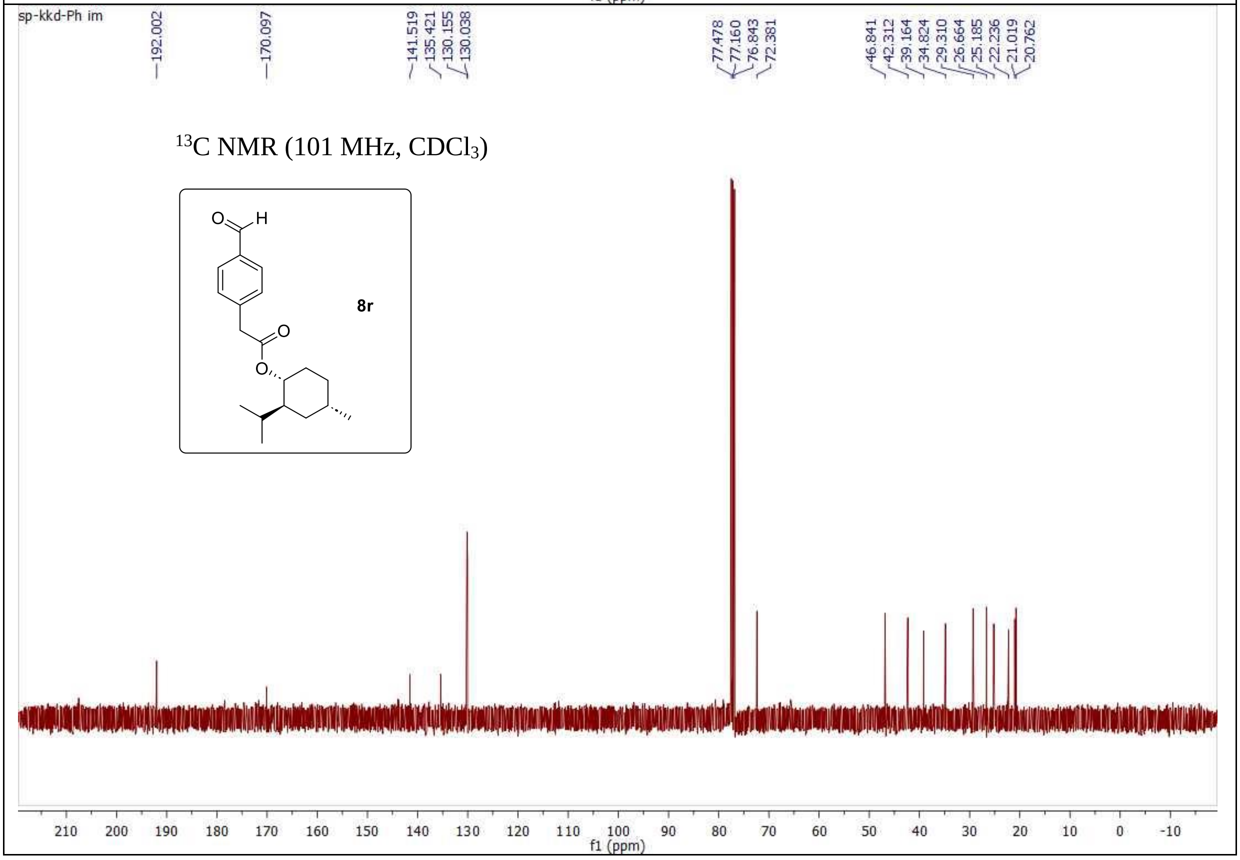
sp-kkd-Ph im
13C NMR (101 MHz, CDCl3)
8r
f1 (ppm)

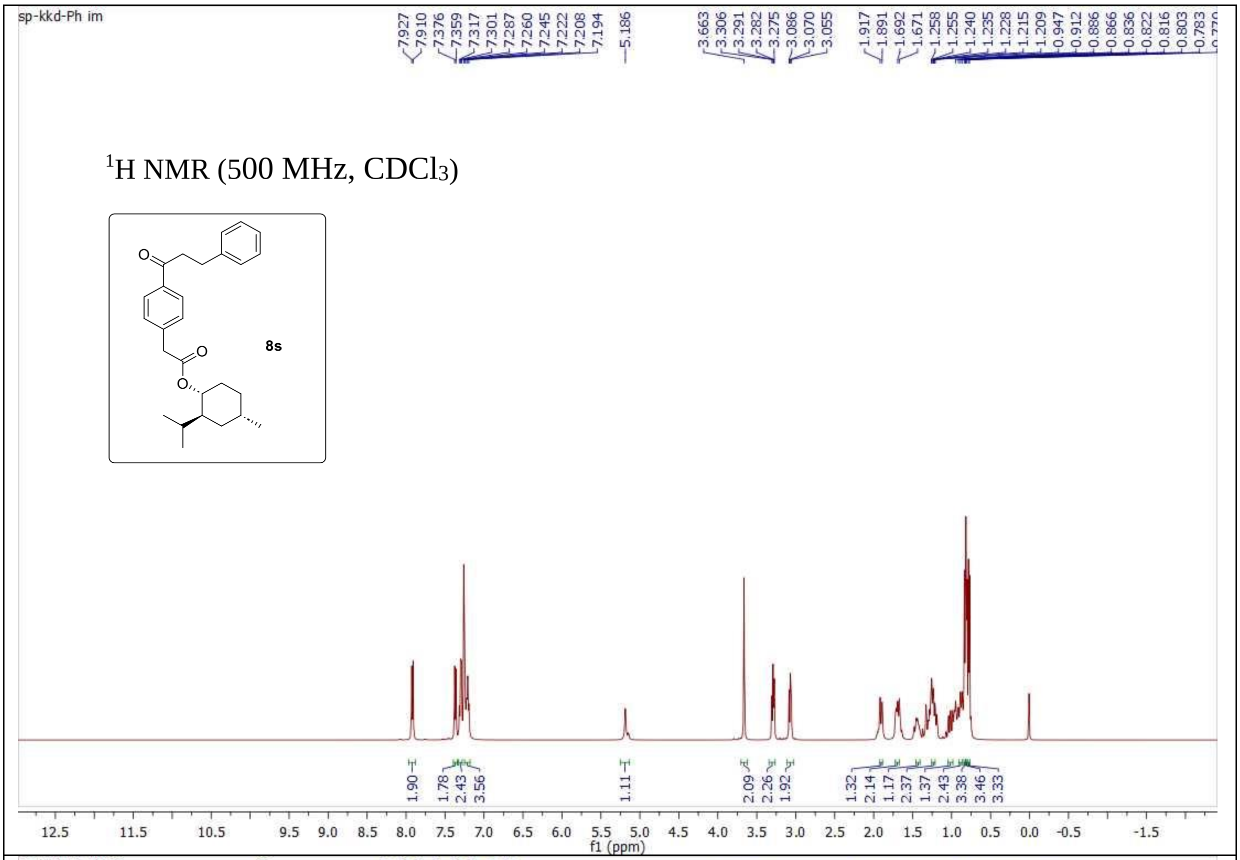

sp-kkd-Ph im
1H NMR (500 MHz, CDCl3)
8s
f1 (ppm)

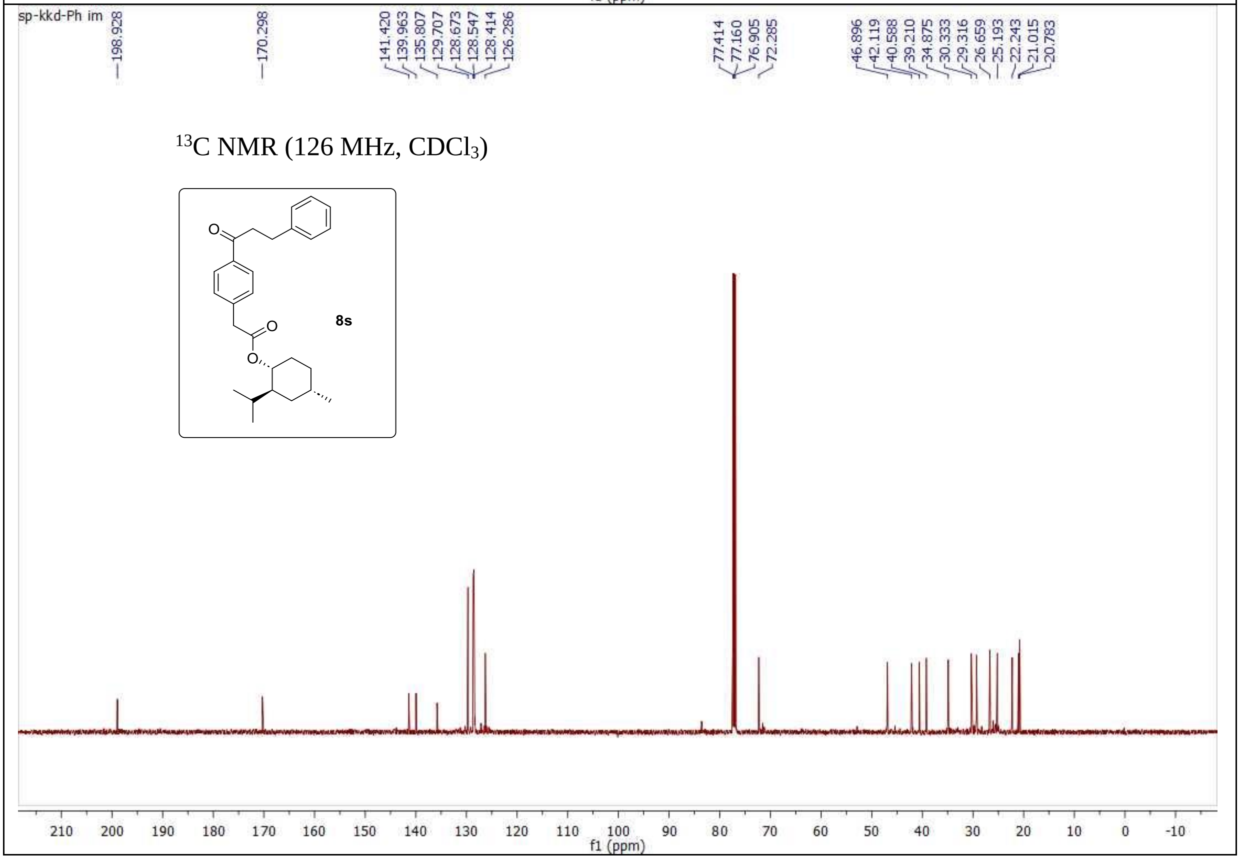

sp-kkd-Ph im
13C NMR (126 MHz, CDCl3)
8s
f1 (ppm)

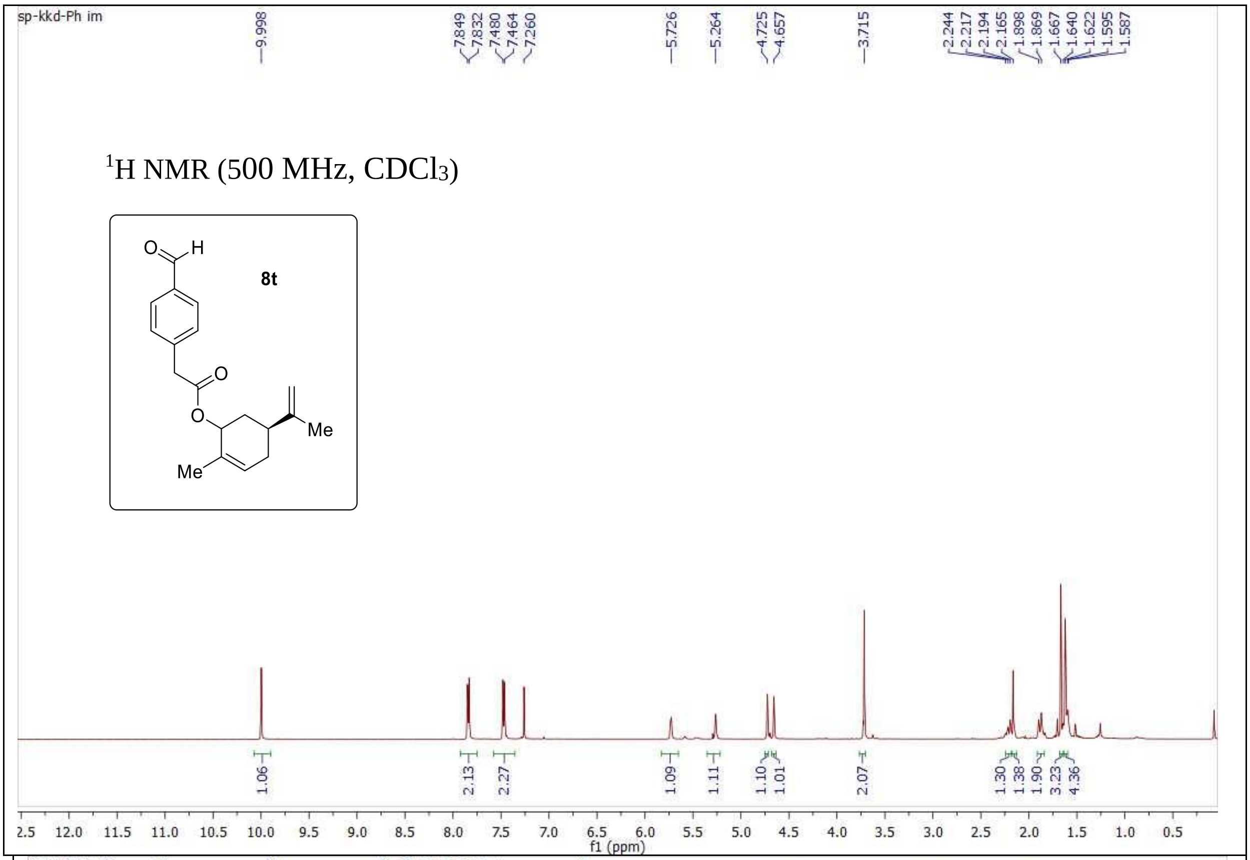
sp-kkd-Ph im
^{1}H NMR (500 MHz, $CDCl_3$)
8t
f1 (ppm)

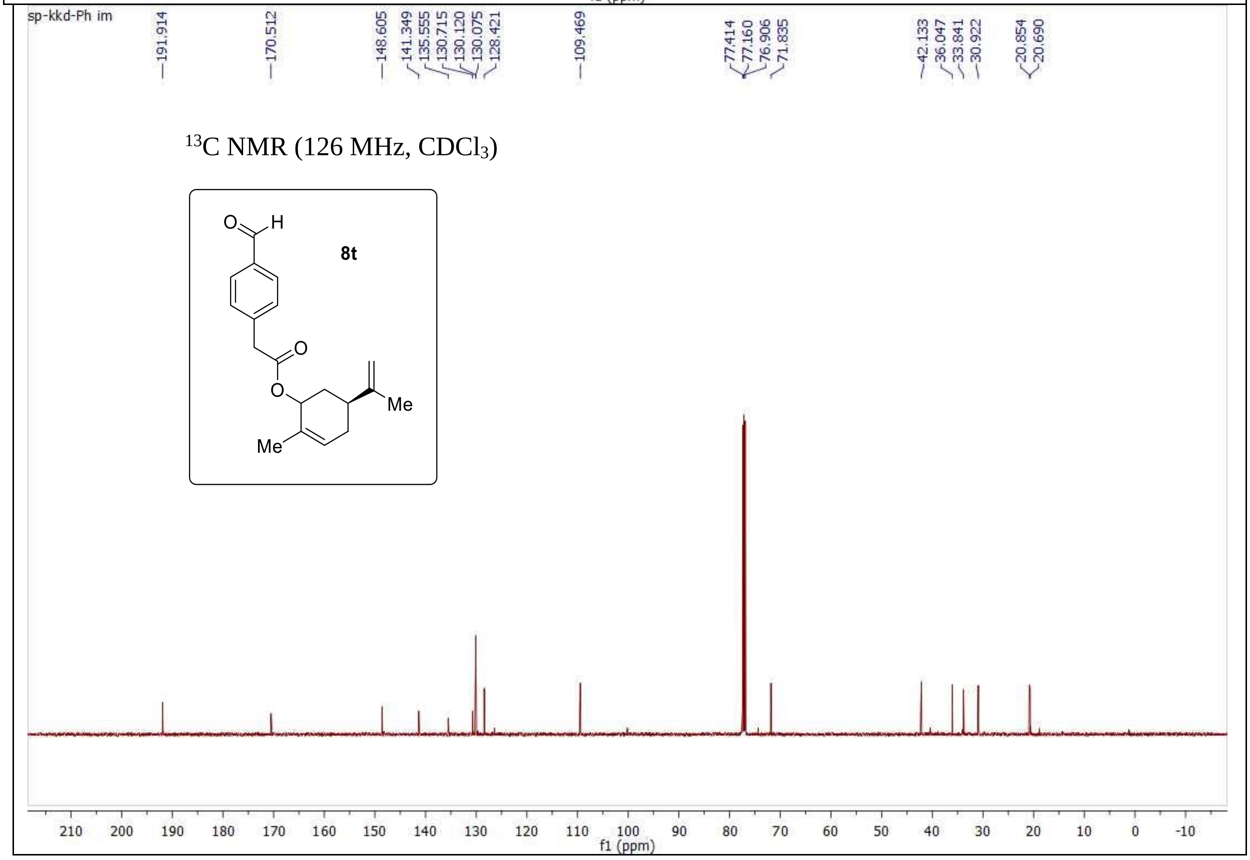
sp-kkd-Ph im
^{13}C NMR (126 MHz, $CDCl_3$)
8t
f1 (ppm)

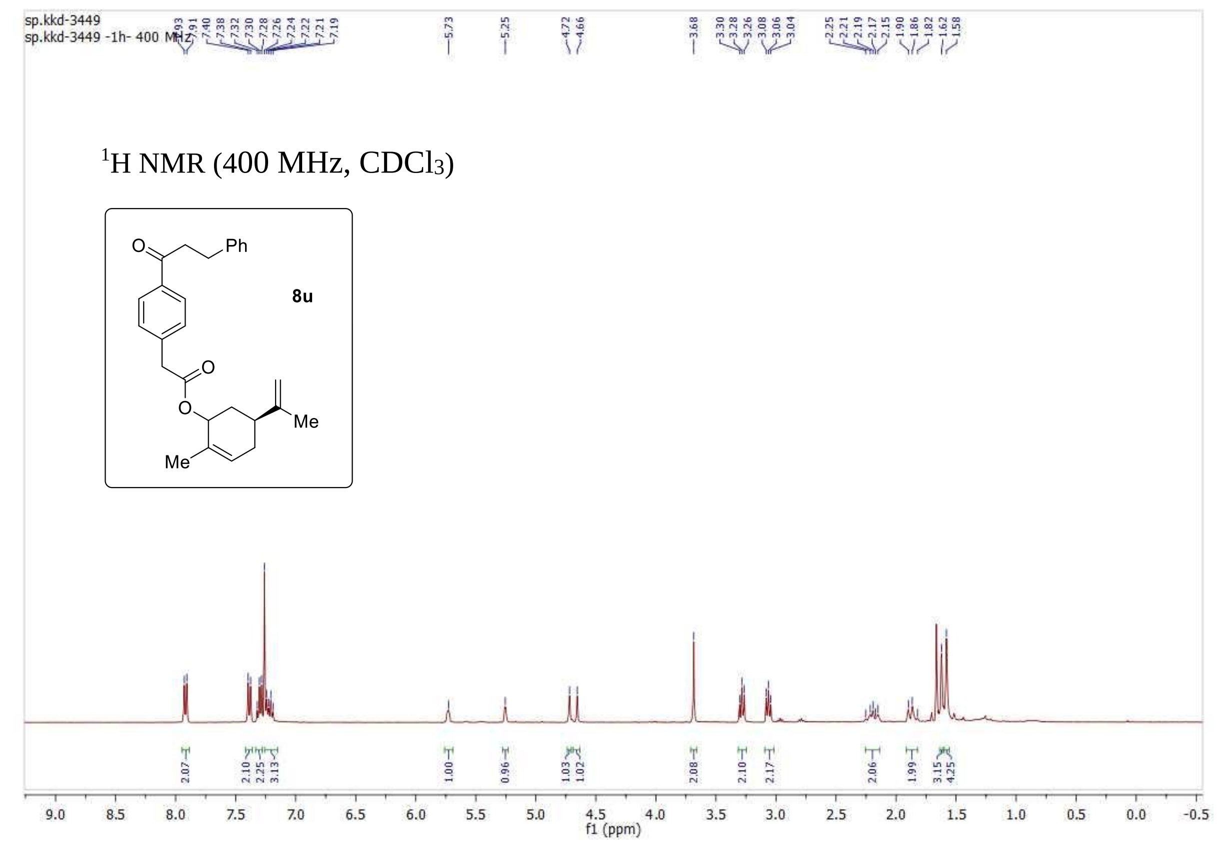

sp.kkd-3449
sp.kkd-3449 -1h- 400 MHz
^{1}H NMR (400 MHz, CDCl$_3$)
8u
f1 (ppm)

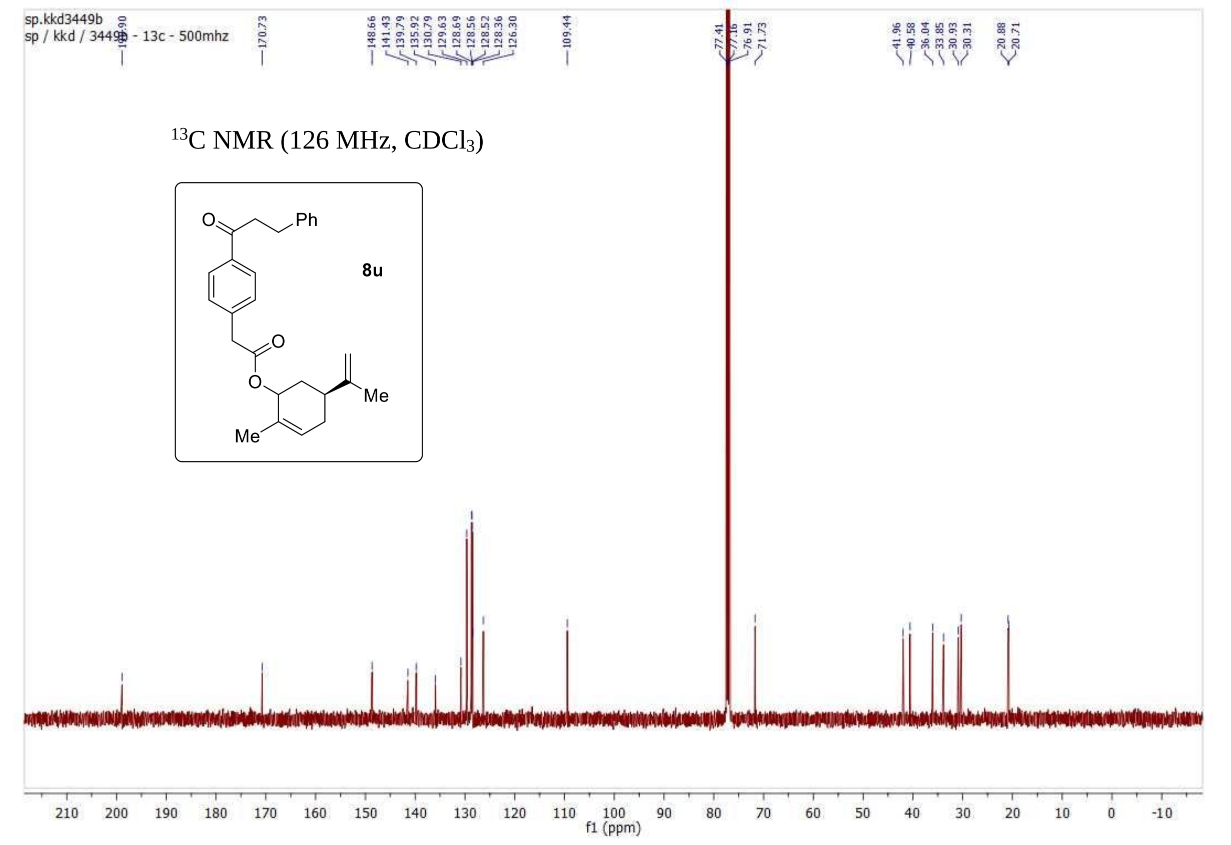

sp.kkd3449b
sp / kkd / 3449b - 13c - 500mhz
^{13}C NMR (126 MHz, CDCl$_3$)
8u
f1 (ppm)

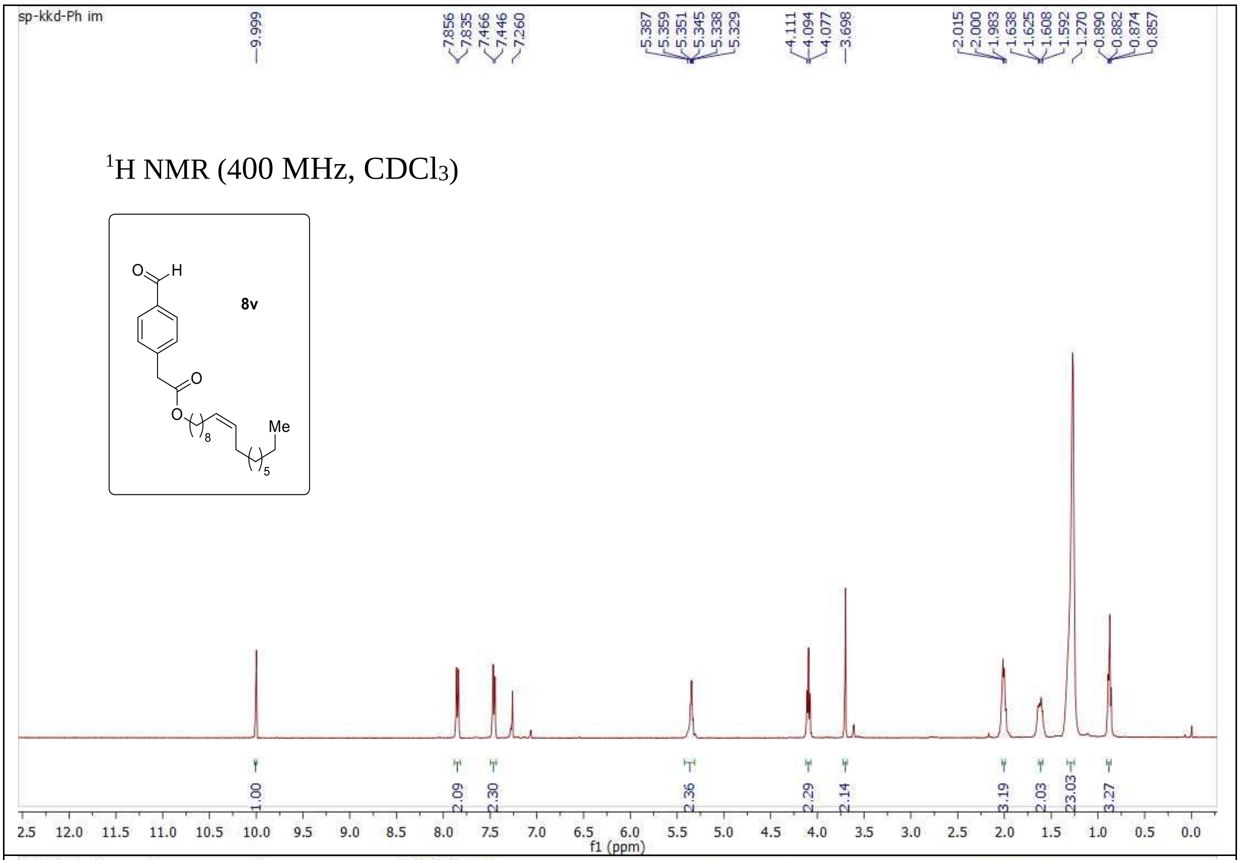
sp-kkd-Ph im
9.999
7.856
7.835
7.466
7.446
7.260
5.387
5.359
5.351
5.345
5.338
5.329
4.111
4.094
4.077
3.698
2.015
2.000
1.983
1.638
1.625
1.608
1.592
1.270
0.890
0.882
0.874
0.857
1H NMR (400 MHz, CDCl3)
8v
Me
1.00
2.09
2.30
2.36
2.29
2.14
3.19
2.03
23.03
3.27
f1 (ppm)

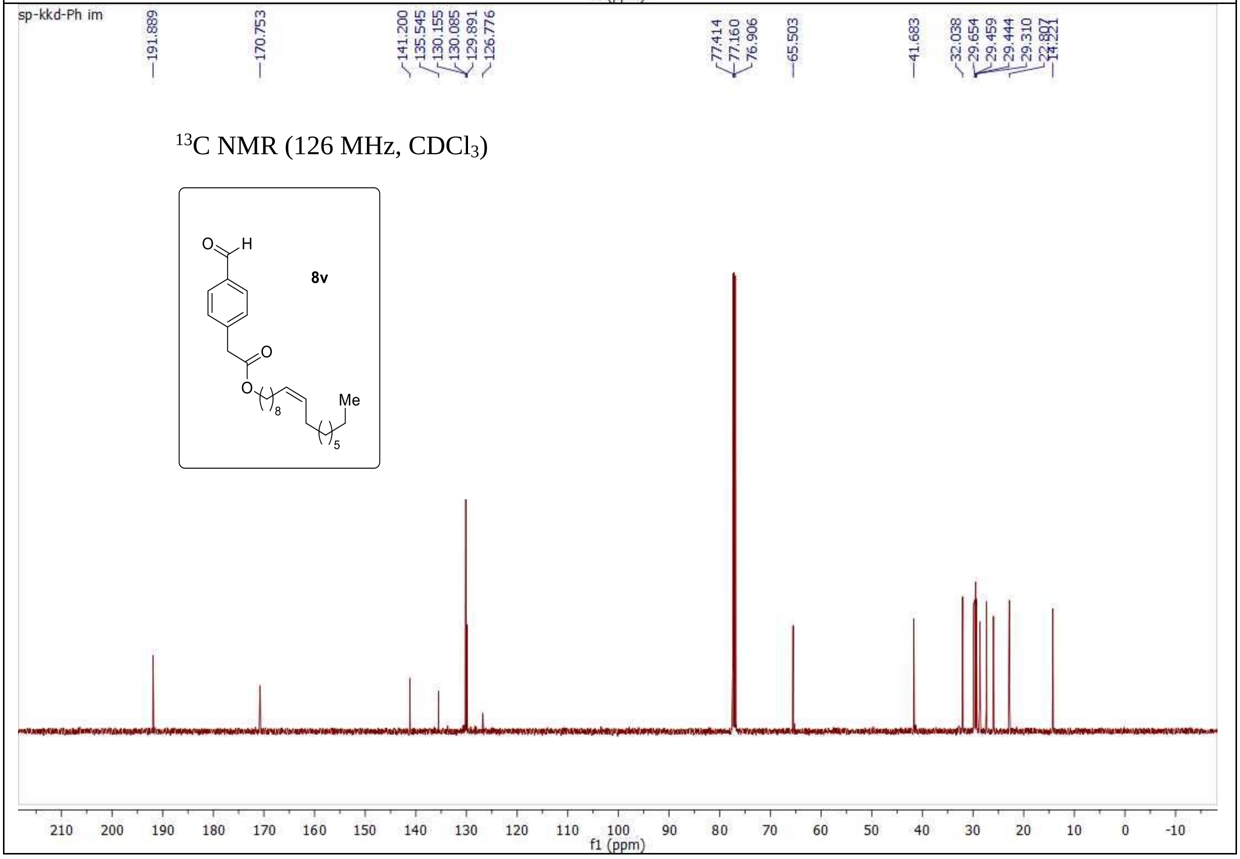
sp-kkd-Ph im
191.889
170.753
141.200
135.545
130.155
130.085
129.891
126.776
77.414
77.160
76.906
65.503
41.683
32.038
29.654
29.459
29.444
29.310
13C NMR (126 MHz, CDCl3)
8v
Me
f1 (ppm)

^1H NMR (400 MHz, $CDCl_3$)

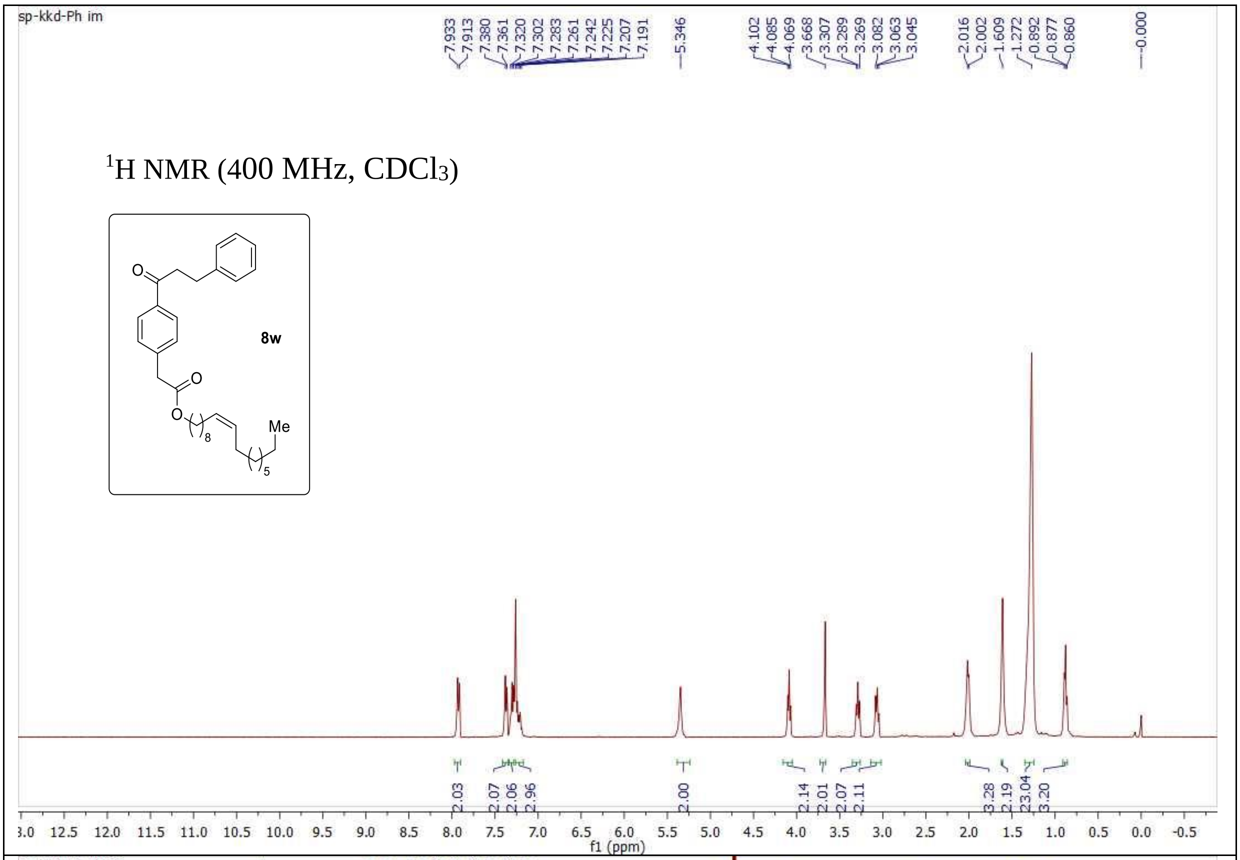

^13C NMR (126 MHz, $CDCl_3$)

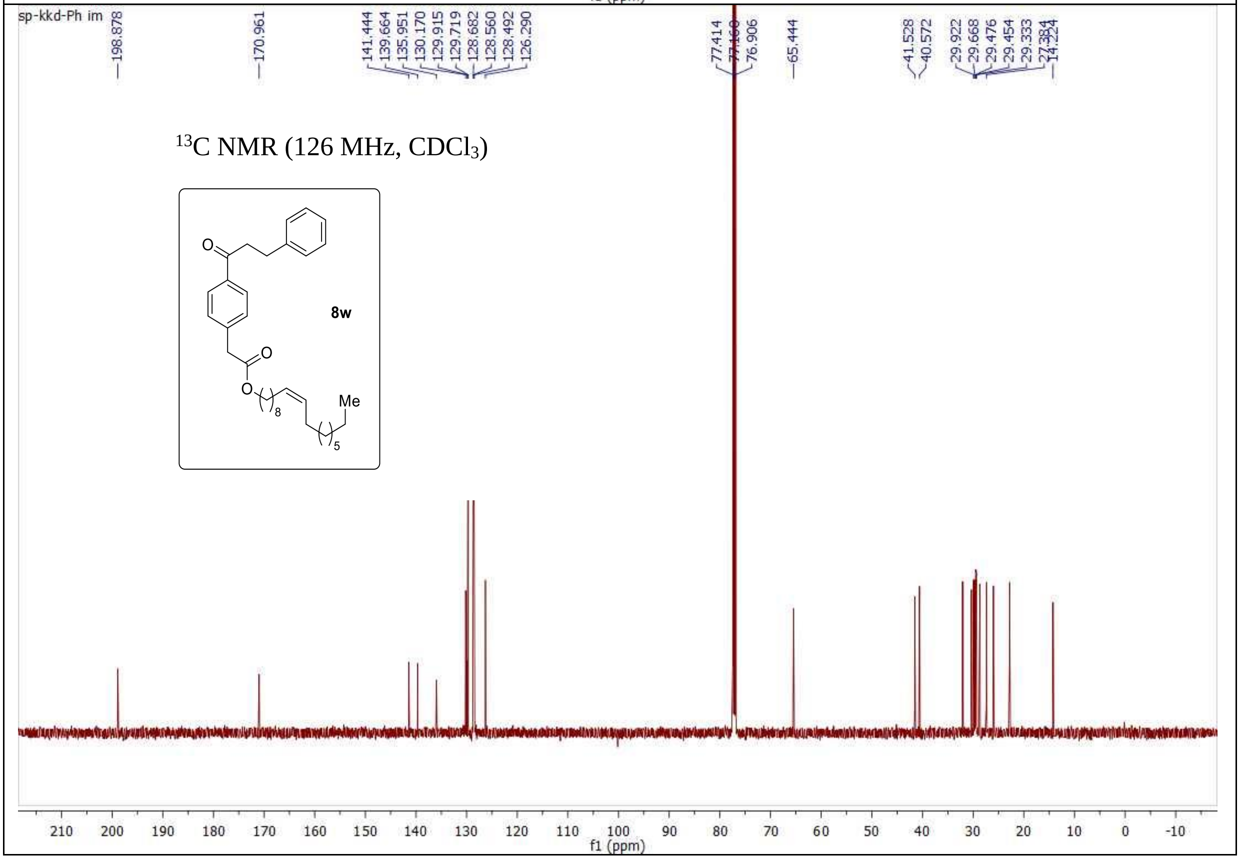

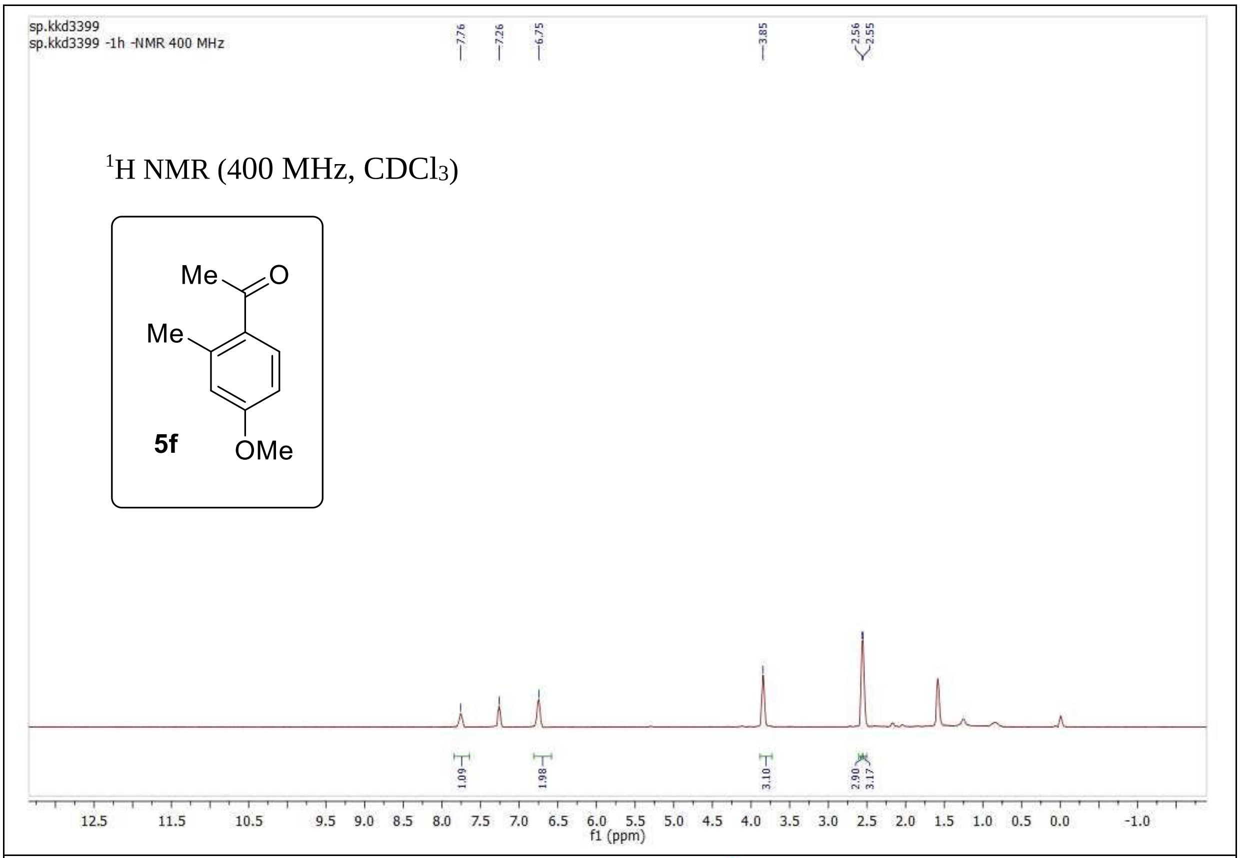
sp.kkd3399
sp.kkd3399 -1h -NMR 400 MHz
7.76
7.26
6.75
3.85
2.56
2.55
^{1}H NMR (400 MHz, CDCl$_3$)
Me
O
Me
5f
OMe
1.09
1.98
3.10
2.90
3.17
f1 (ppm)

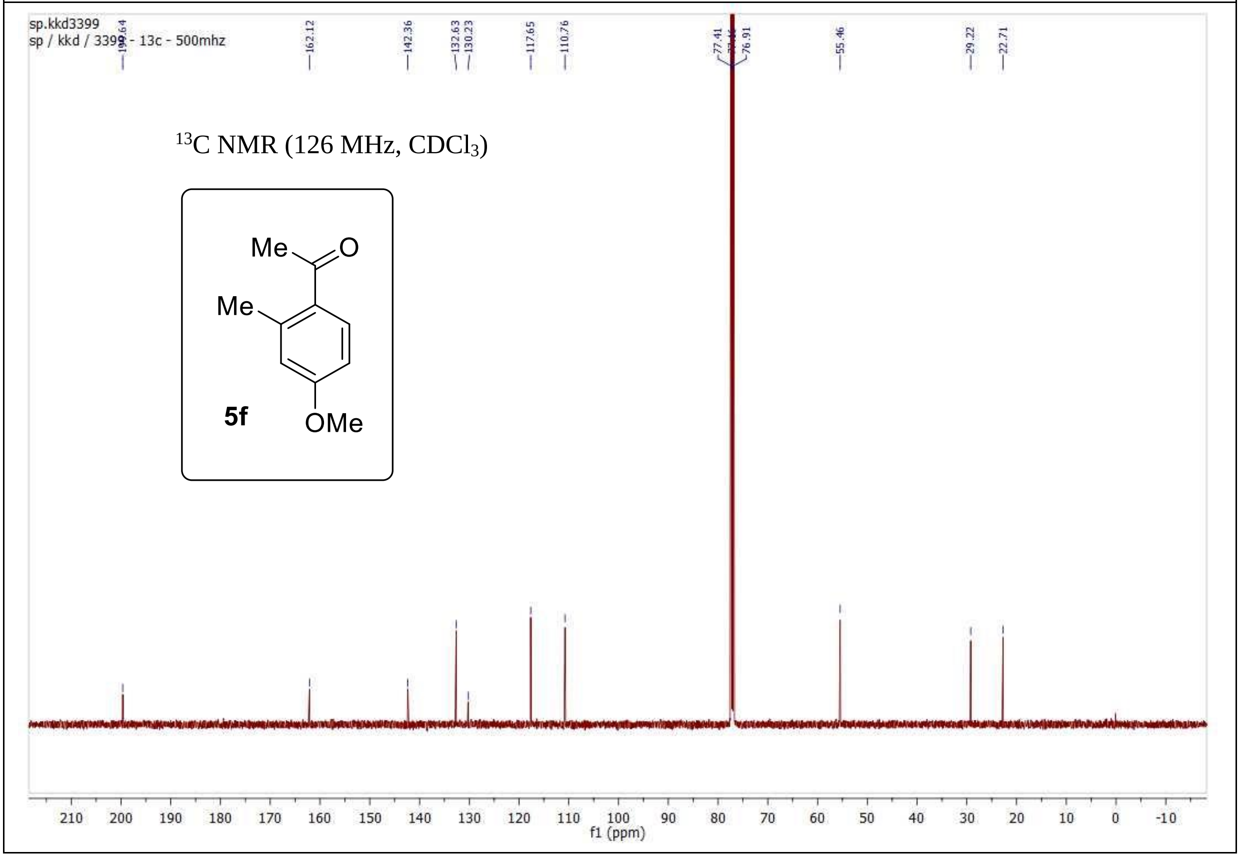
sp.kkd3399
sp / kkd / 3399 - 13c - 500mhz
162.12
142.36
132.63
130.23
117.65
110.76
77.41
76.91
55.46
29.22
22.71
^{13}C NMR (126 MHz, CDCl$_3$)
Me
O
Me
5f
OMe
f1 (ppm)

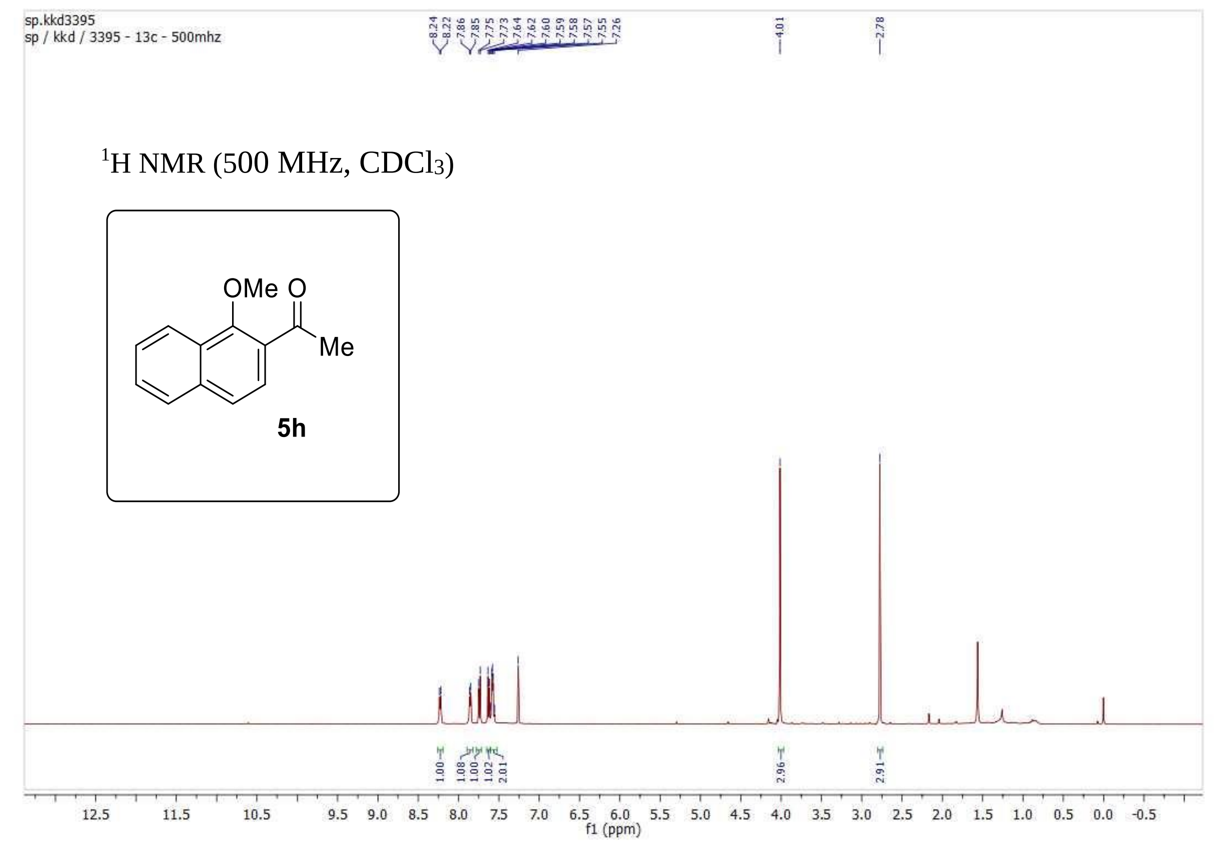
sp.kkd3395
sp / kkd / 3395 - 13c - 500mhz
8.24 8.22 7.86 7.85 7.75 7.73 7.64 7.62 7.60 7.59 7.58 7.57 7.55 7.26
4.01
2.78
1H NMR (500 MHz, CDCl3)
OMe O
Me
5h
1.00 1.08 1.00 1.02 2.01
2.96
2.91
12.5 11.5 10.5 9.5 9.0 8.5 8.0 7.5 7.0 6.5 6.0 5.5 5.0 4.5 4.0 3.5 3.0 2.5 2.0 1.5 1.0 0.5 0.0 -0.5
f1 (ppm)

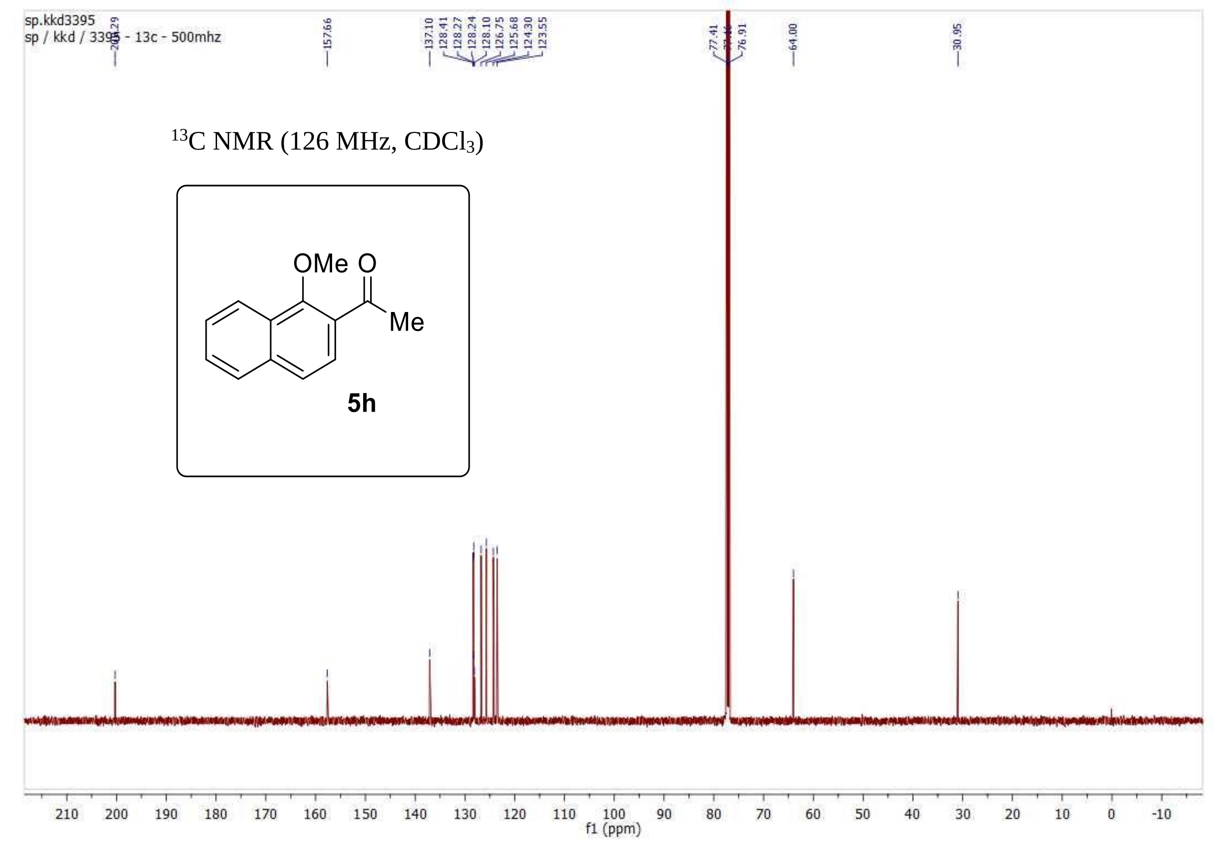
sp.kkd3395
sp / kkd / 3395 - 13c - 500mhz
157.66
137.10 128.41 128.27 128.24 128.10 126.75 125.68 124.30 123.55
77.41 76.91
64.00
30.95
13C NMR (126 MHz, CDCl3)
OMe O
Me
5h
210 200 190 180 170 160 150 140 130 120 110 100 90 80 70 60 50 40 30 20 10 0 -10
f1 (ppm)

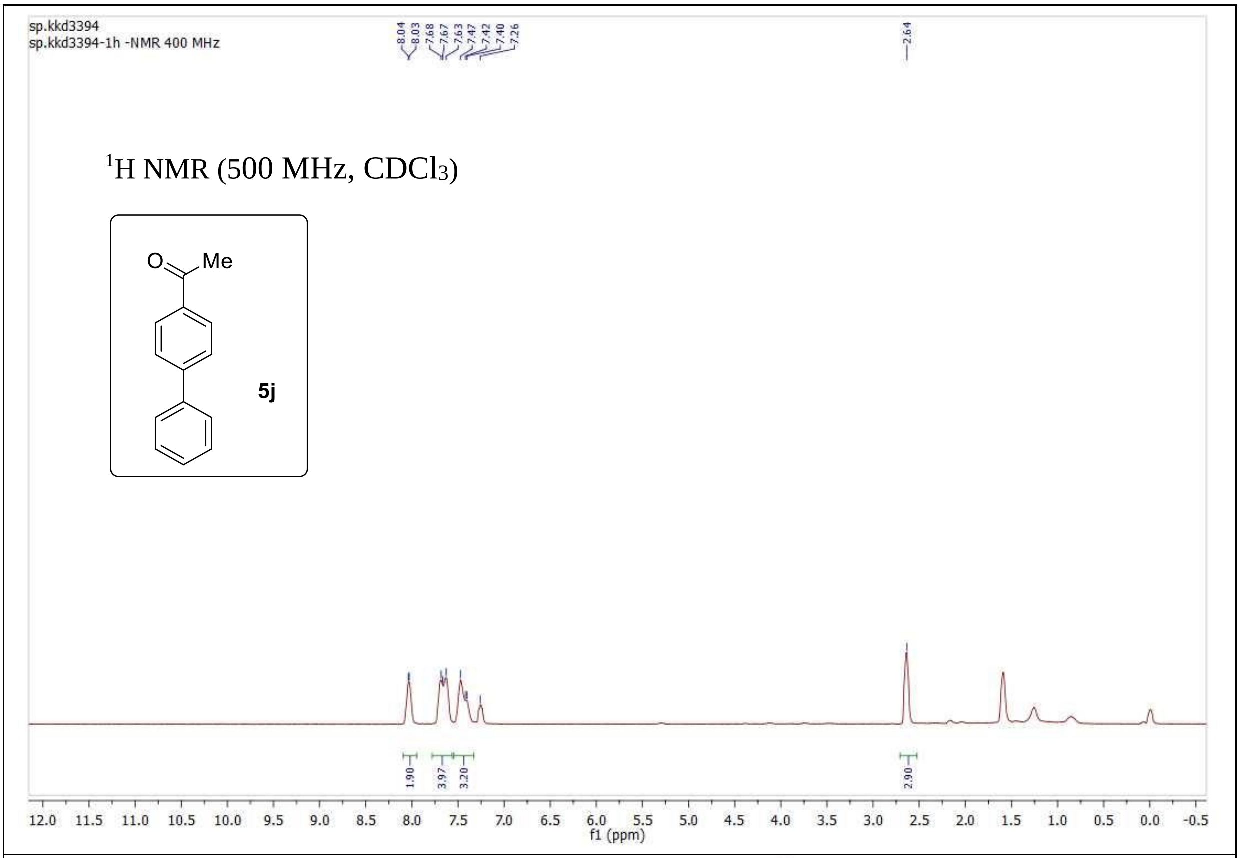
sp.kkd3394
sp.kkd3394-1h -NMR 400 MHz
8.04
8.03
7.68
7.67
7.63
7.47
7.42
7.40
7.26
2.64
^{1}H NMR (500 MHz, $CDCl_3$)
O
Me
5j
1.90
3.97
3.20
2.90
12.0 11.5 11.0 10.5 10.0 9.5 9.0 8.5 8.0 7.5 7.0 6.5 6.0 5.5 5.0 4.5 4.0 3.5 3.0 2.5 2.0 1.5 1.0 0.5 0.0 -0.5
f1 (ppm)

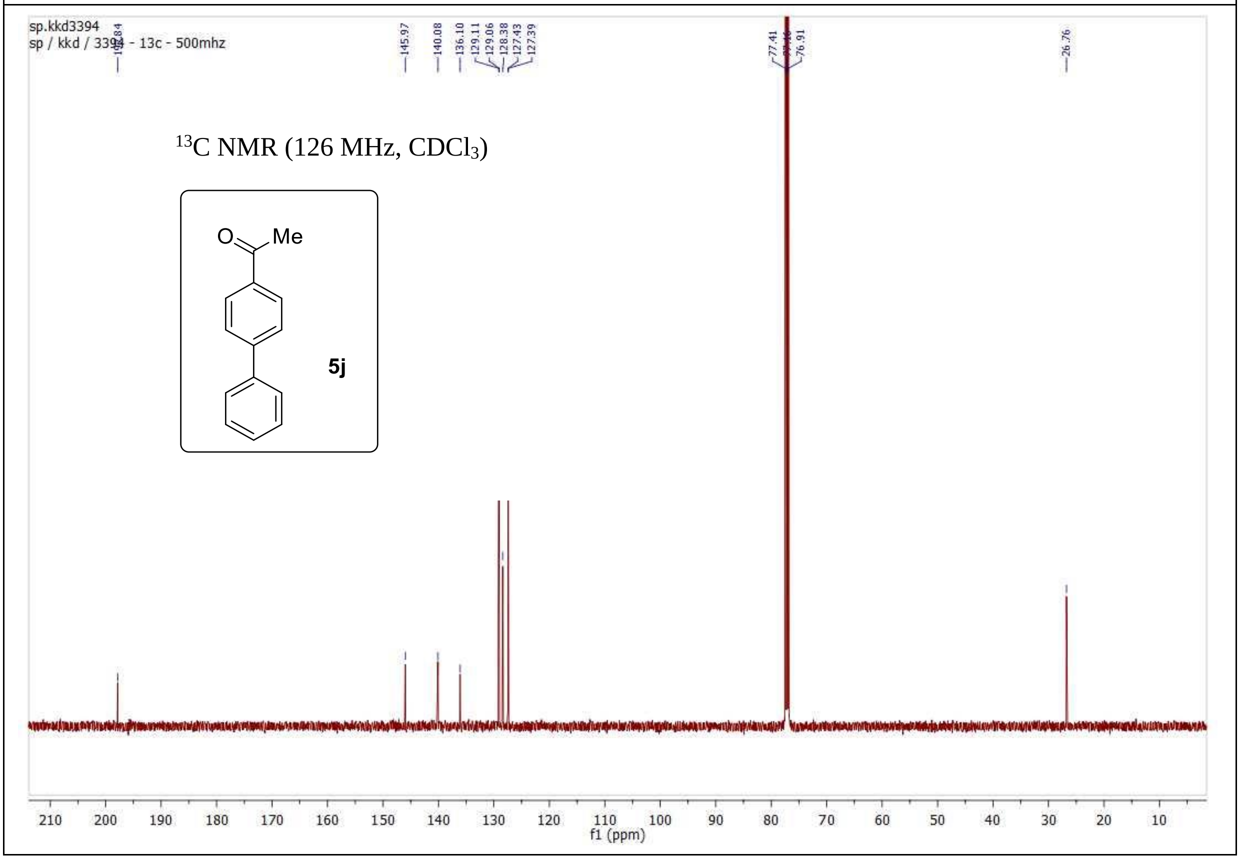
sp.kkd3394
sp / kkd / 3394 - 13c - 500mhz
145.97
140.08
136.10
129.11
129.06
128.38
127.43
127.39
77.41
76.91
26.76
^{13}C NMR (126 MHz, $CDCl_3$)
O
Me
5j
210 200 190 180 170 160 150 140 130 120 110 100 90 80 70 60 50 40 30 20 10
f1 (ppm)

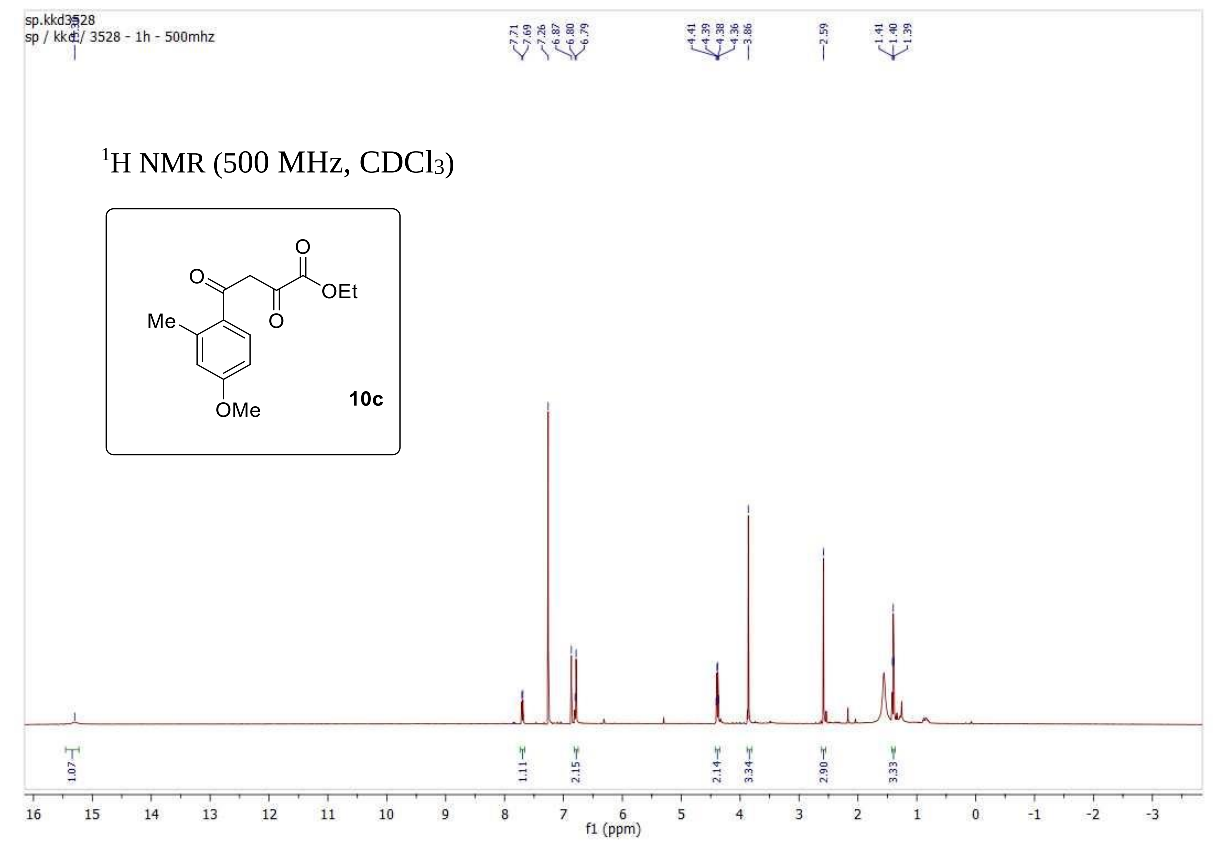
sp.kkd3528
sp / kkd / 3528 - 1h - 500mhz
7.71
7.69
7.26
6.87
6.80
6.79
4.41
4.39
4.38
4.36
3.86
2.59
1.41
1.40
1.39
^{1}H NMR (500 MHz, $CDCl_3$)
O
O
OEt
O
Me
OMe
10c
1.07
1.11
2.15
2.14
3.34
2.90
3.33
f1 (ppm)

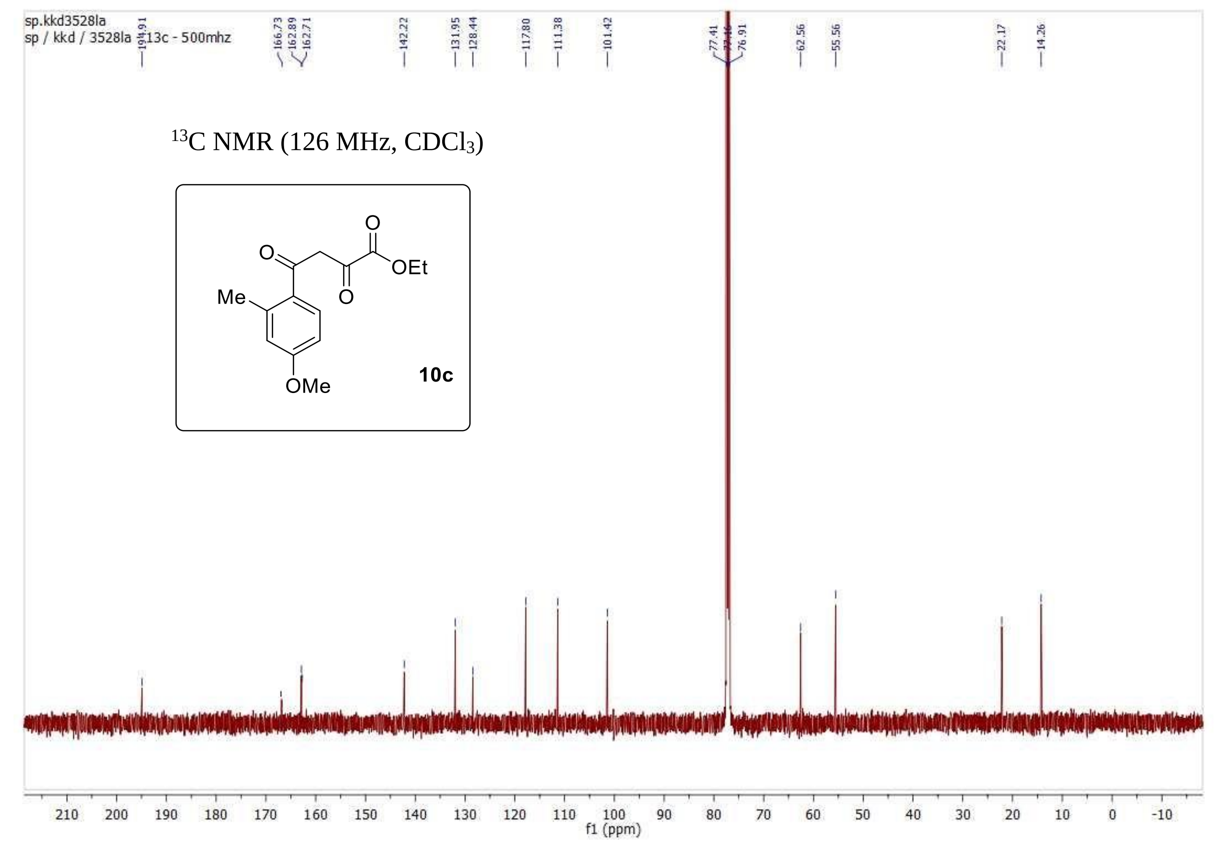
sp.kkd3528la
sp / kkd / 3528la - 13c - 500mhz
194.91
166.73
162.89
162.71
142.22
131.95
128.44
117.80
111.38
101.42
77.41
76.91
62.56
55.56
22.17
14.26
^{13}C NMR (126 MHz, $CDCl_3$)
O
O
OEt
O
Me
OMe
10c
f1 (ppm)

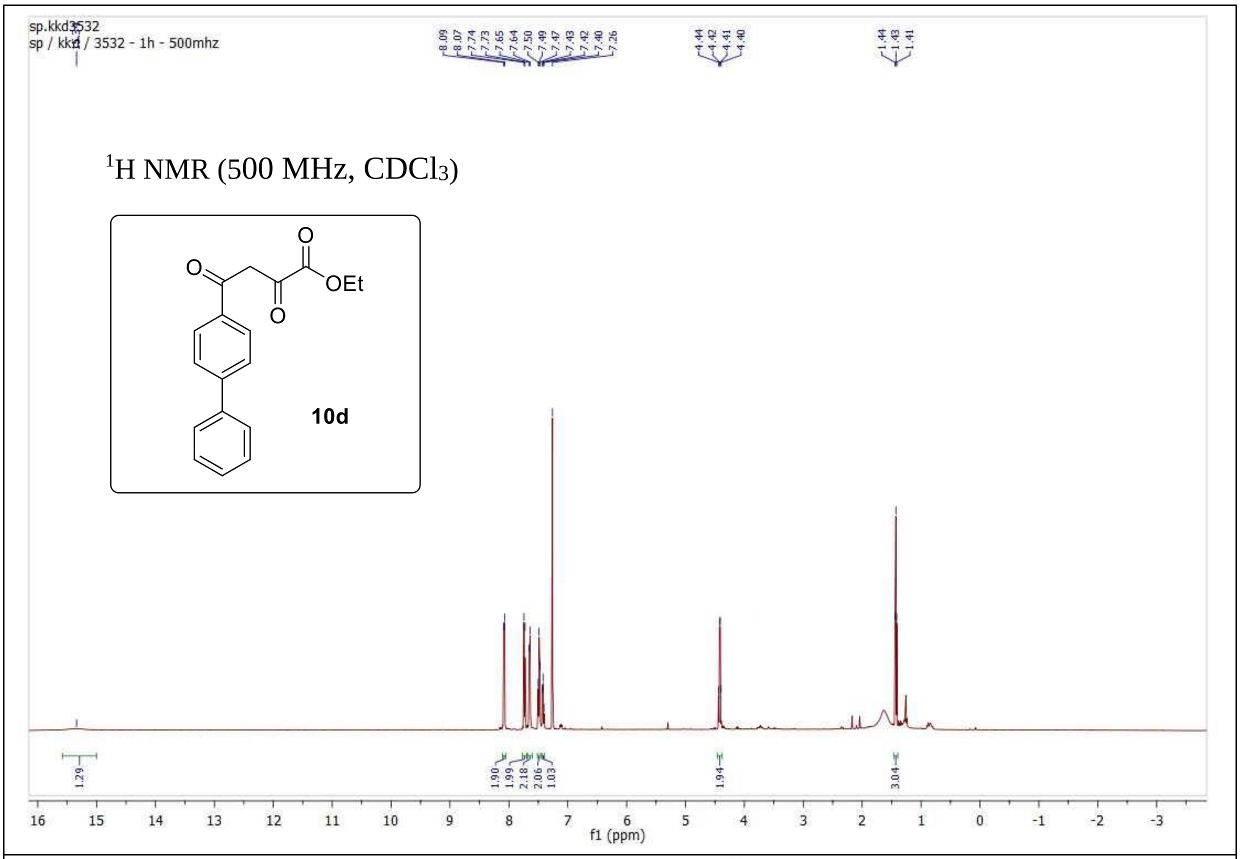
sp.kkd3532
sp / kkd / 3532 - 1h - 500mhz
8.09 8.07 7.74 7.73 7.65 7.64 7.50 7.49 7.47 7.43 7.42 7.40 7.26
4.44 4.42 4.41 4.40
1.44 1.43 1.41
1H NMR (500 MHz, CDCl3)
O
O
OEt
O
10d
1.29
1.90 1.99 2.18 2.06 1.03
1.94
3.04
16 15 14 13 12 11 10 9 8 7 6 5 4 3 2 1 0 -1 -2 -3
f1 (ppm)

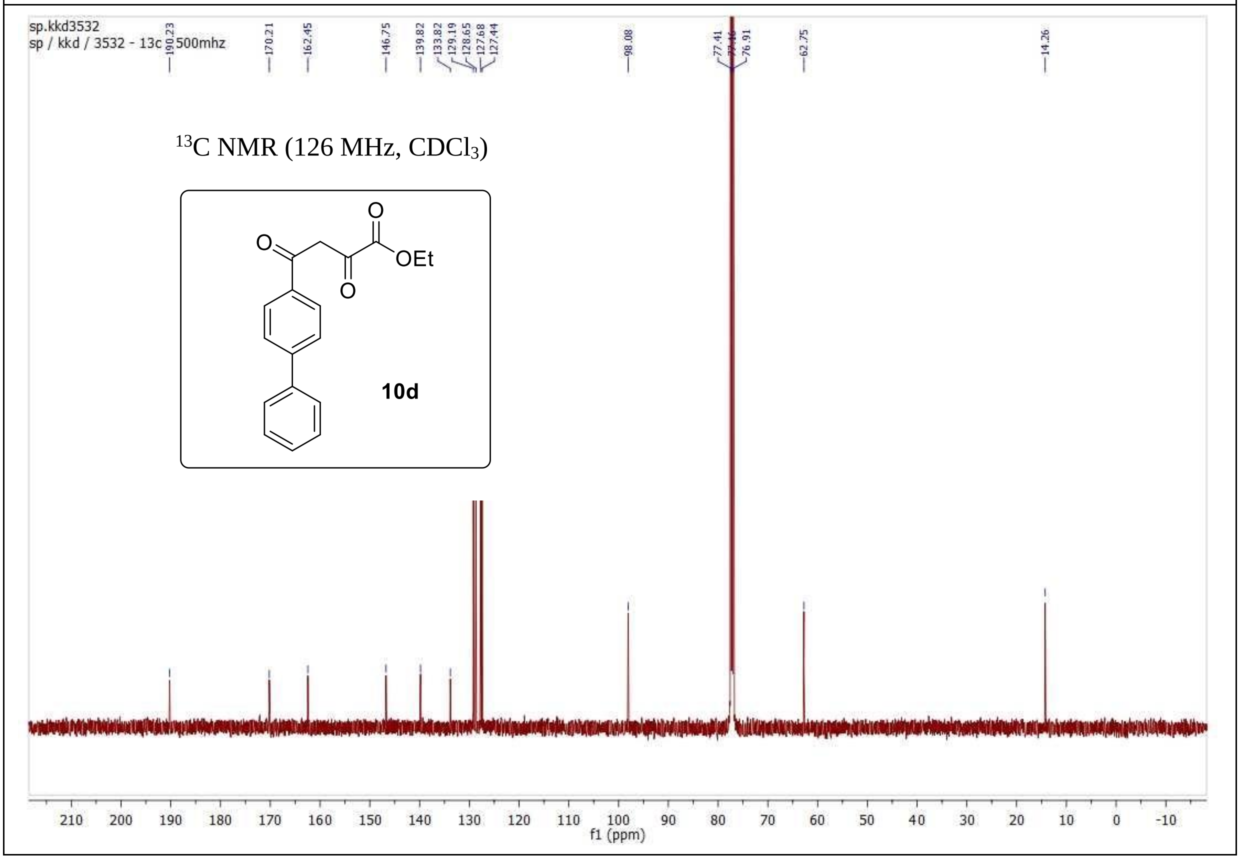
sp.kkd3532
sp / kkd / 3532 - 13c 500mhz
190.23
170.21
162.45
146.75
139.82 133.82 129.19 128.65 127.68 127.44
98.08
77.41 77.16 76.91
62.75
14.26
13C NMR (126 MHz, CDCl3)
O
O
OEt
O
10d
210 200 190 180 170 160 150 140 130 120 110 100 90 80 70 60 50 40 30 20 10 0 -10
f1 (ppm)

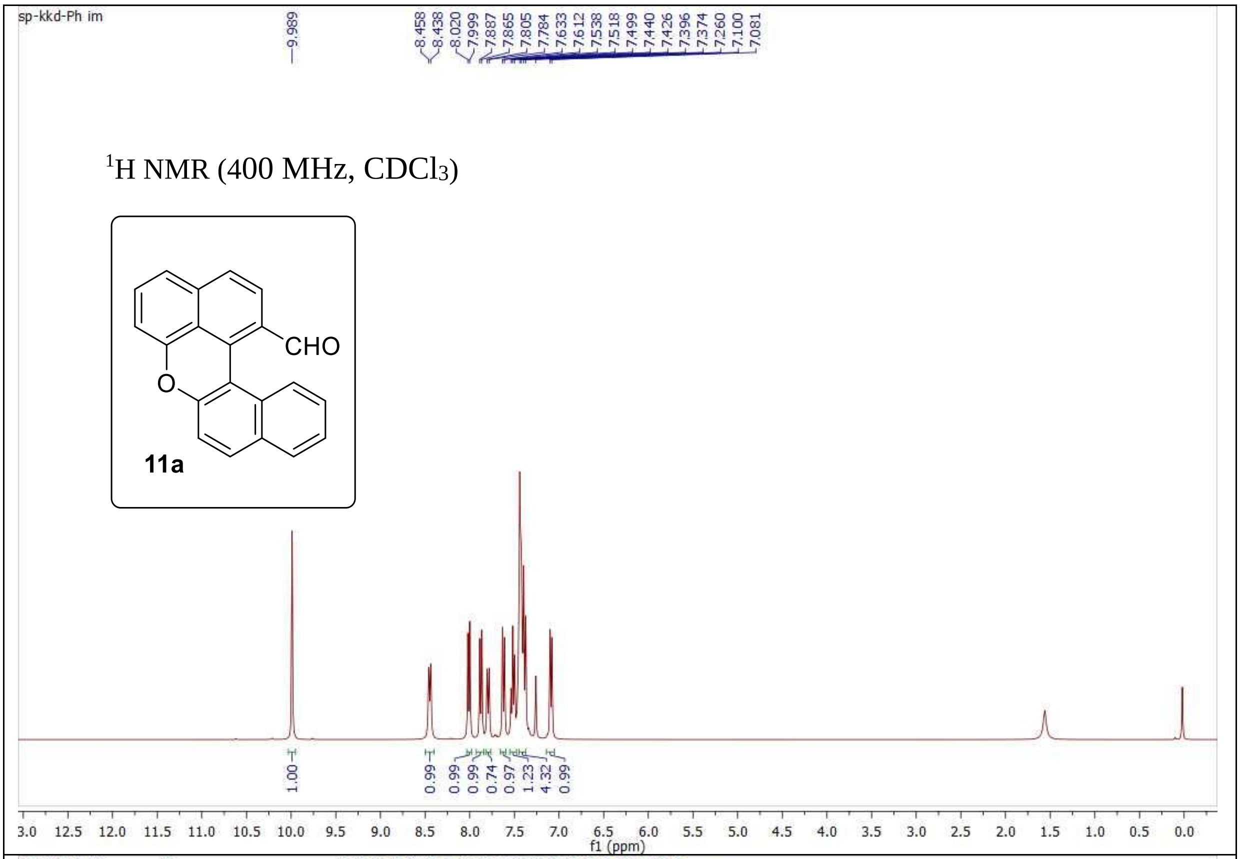
sp-kkd-Ph im
9.989
8.458
8.438
8.020
7.999
7.887
7.865
7.805
7.784
7.633
7.612
7.538
7.518
7.499
7.440
7.426
7.396
7.374
7.260
7.100
7.081
1H NMR (400 MHz, CDCl3)
CHO
O
11a
1.00
0.99
0.99
0.99
0.74
0.97
1.23
4.32
0.99
f1 (ppm)

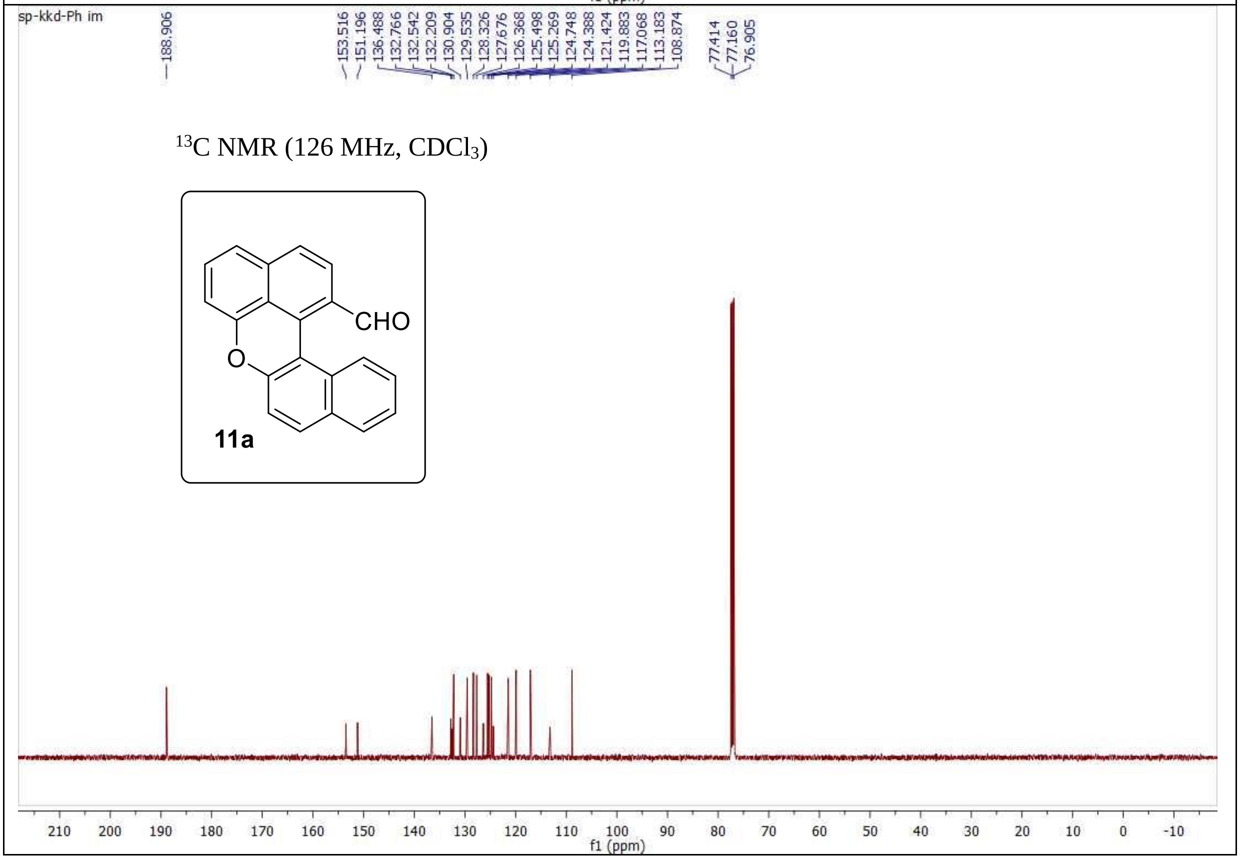
sp-kkd-Ph im
188.906
153.516
151.196
136.488
132.766
132.542
132.209
130.904
129.535
128.326
127.676
126.368
125.498
125.269
124.748
124.388
121.424
119.883
117.068
113.183
108.874
77.414
77.160
76.905
13C NMR (126 MHz, CDCl3)
CHO
O
11a
f1 (ppm)

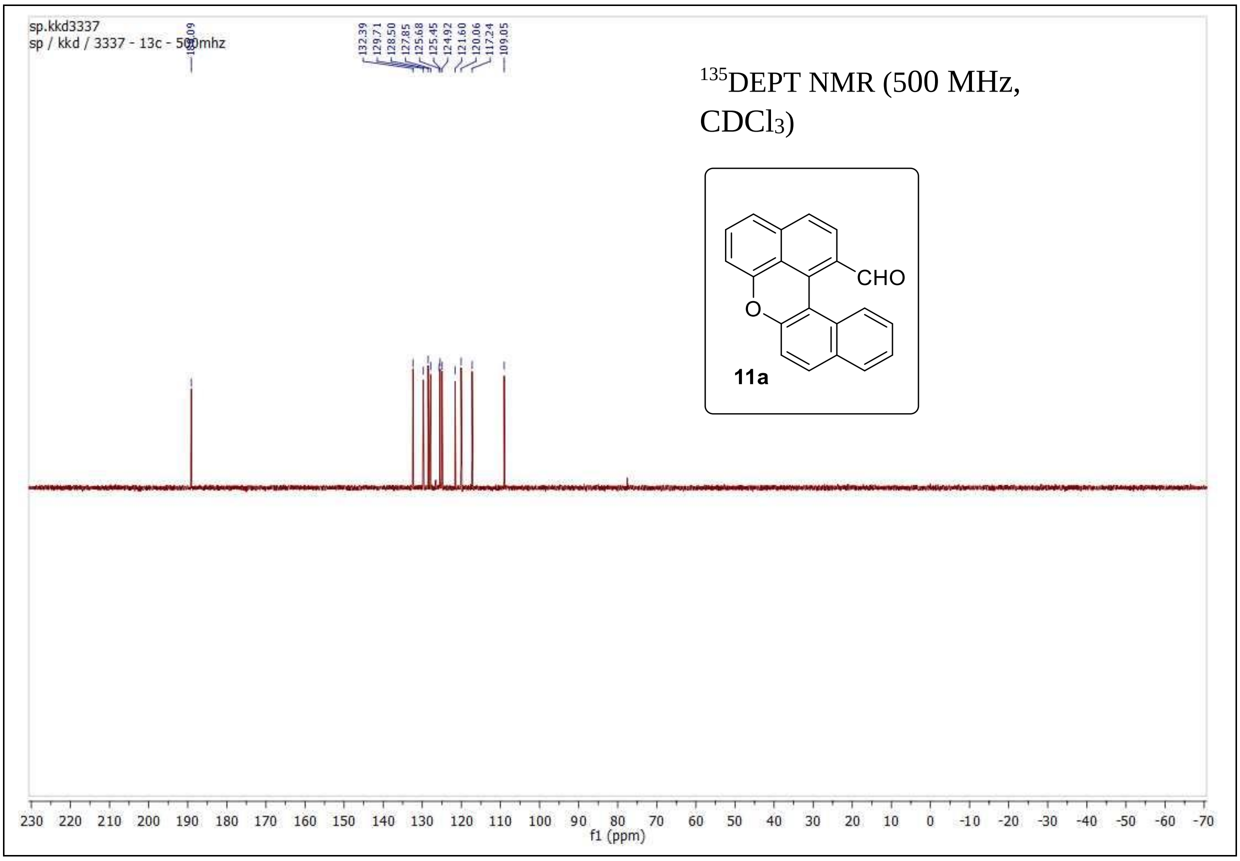
sp.kkd3337
132.39
129.71
128.50
127.85
125.68
125.45
124.92
121.60
120.06
117.24
109.05
135DEPT NMR (500 MHz, CDCl3)
CHO
O
11a
f1 (ppm)

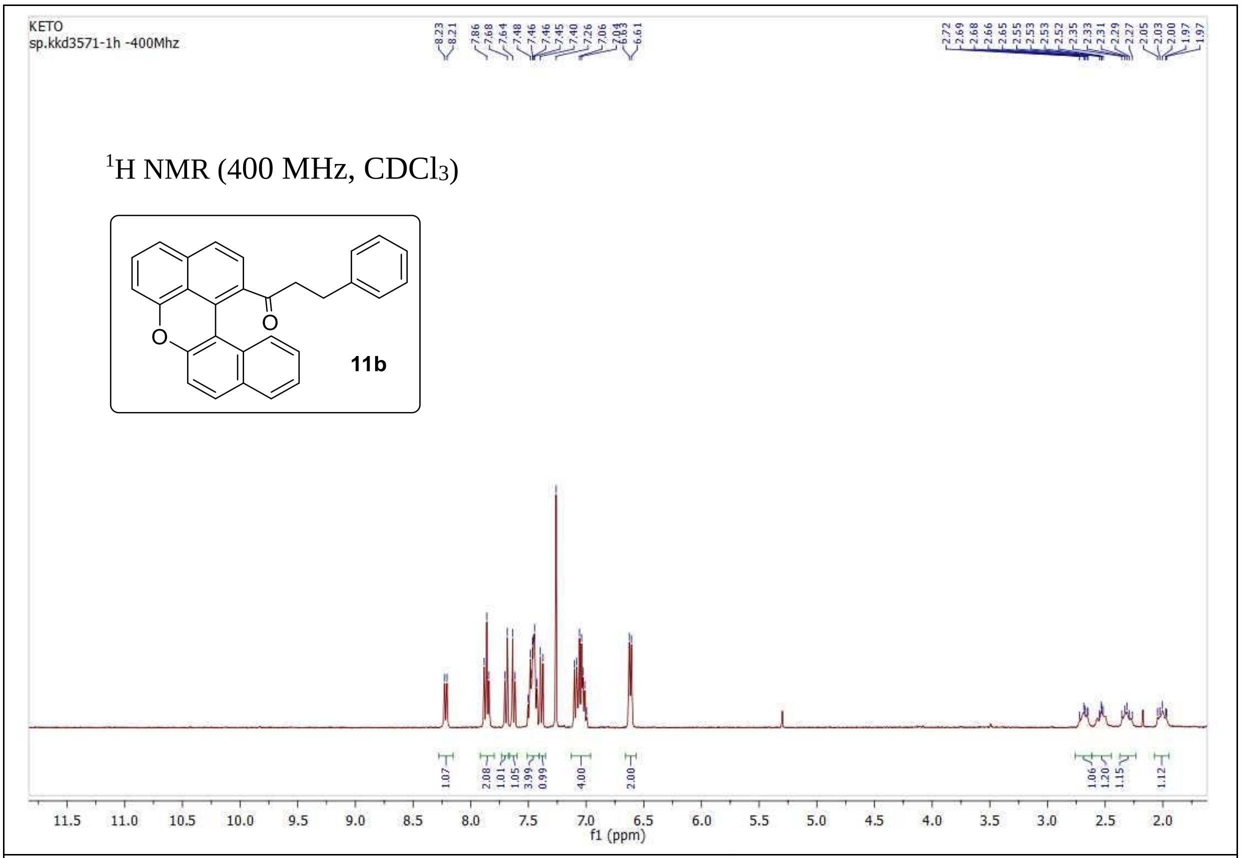

KETO
sp.kkd3571-1h -400Mhz
1H NMR (400 MHz, CDCl3)
11b
f1 (ppm)

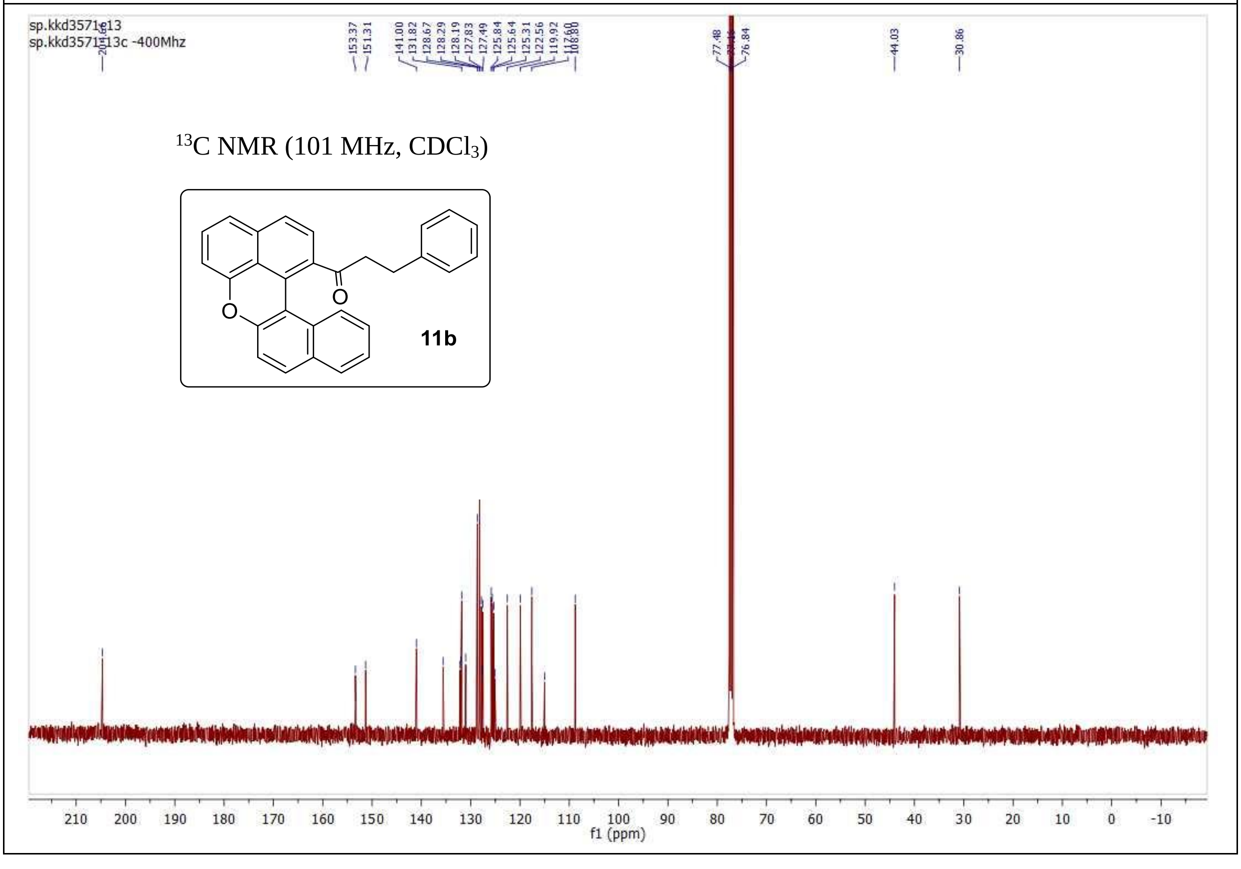

sp.kkd3571c13
sp.kkd3571-13c -400Mhz
13C NMR (101 MHz, CDCl3)
11b
f1 (ppm)

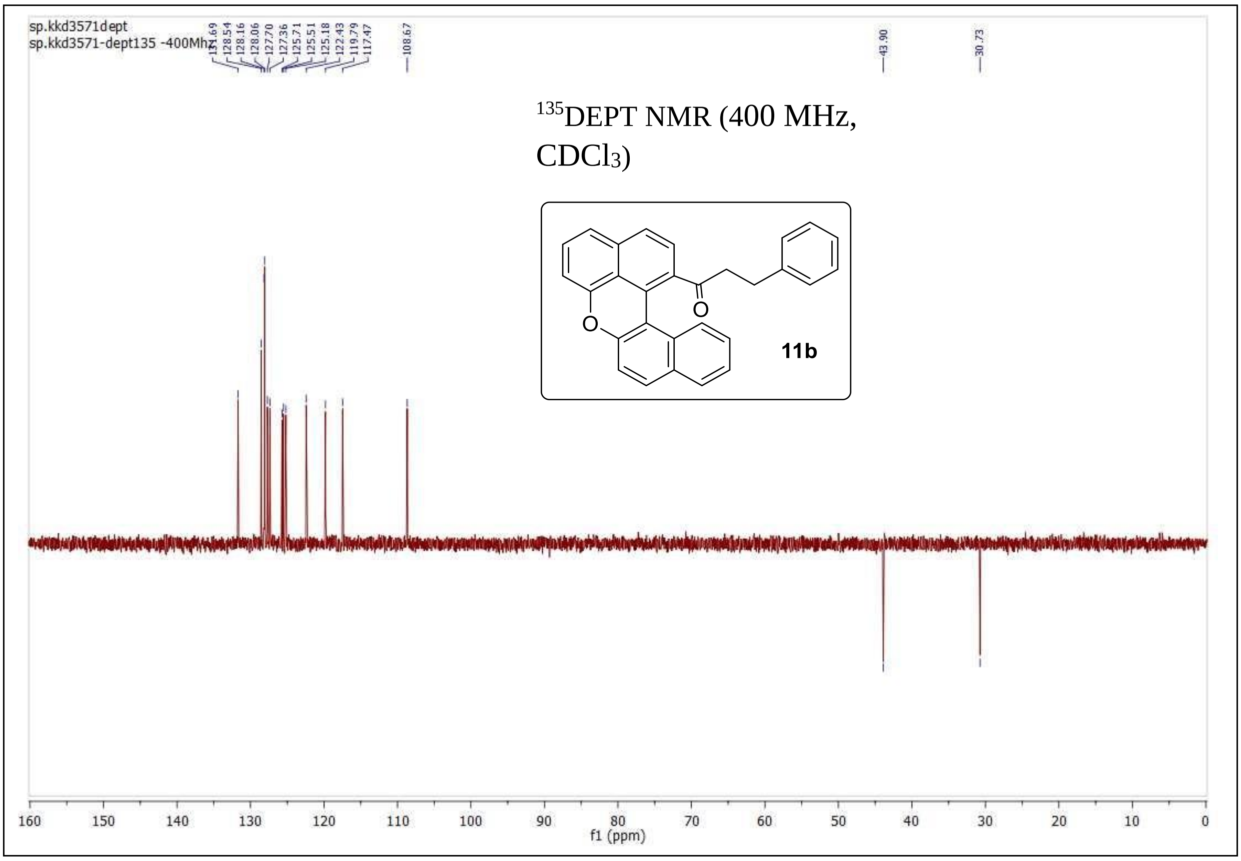
sp.kkd3571dept
sp.kkd3571-dept135 -400Mhz
131.69
128.54
128.16
128.06
127.70
127.36
125.71
125.51
125.18
122.43
119.79
117.47
108.67
43.90
30.73
135DEPT NMR (400 MHz, CDCl3)
11b
f1 (ppm)

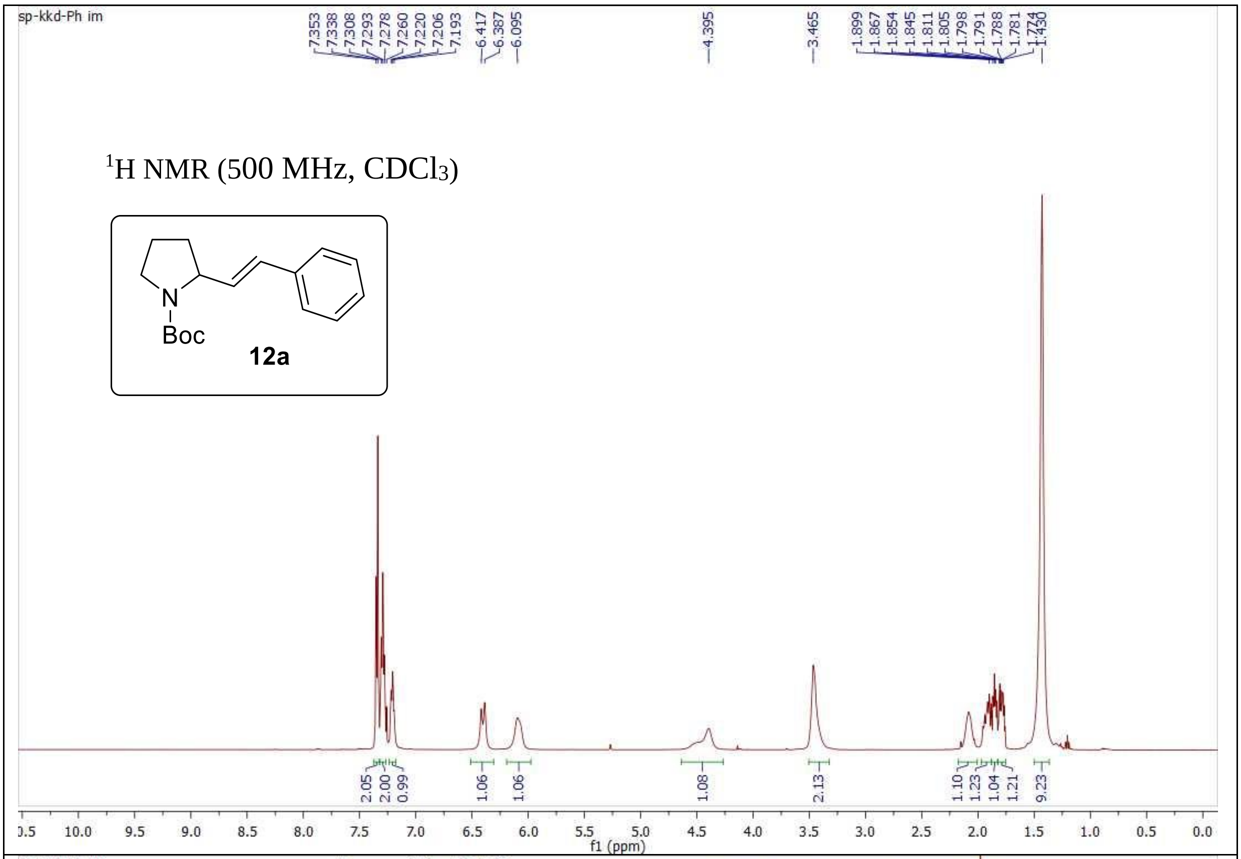
sp-kkd-Ph im
7.353
7.338
7.308
7.293
7.278
7.260
7.220
7.206
7.193
6.417
6.387
6.095
4.395
3.465
1.899
1.867
1.854
1.845
1.811
1.805
1.798
1.791
1.788
1.781
1.774
1.430
^{1}H NMR (500 MHz, CDCl3)
N
Boc
12a
2.05
2.00
0.99
1.06
1.06
1.08
2.13
1.10
1.23
1.04
1.21
9.23
f1 (ppm)

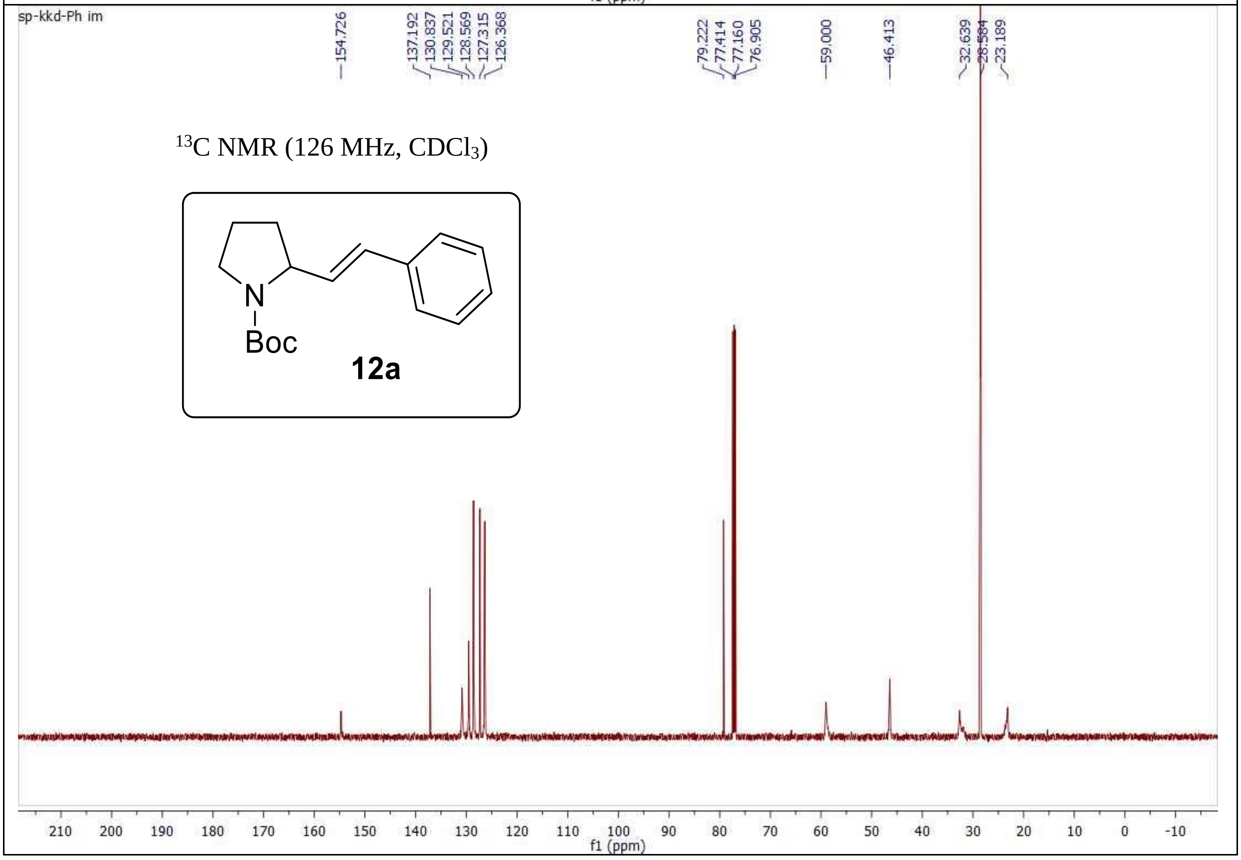
sp-kkd-Ph im
154.726
137.192
130.837
129.521
128.569
127.315
126.368
79.222
77.414
77.160
76.905
59.000
46.413
32.639
28.584
23.189
^{13}C NMR (126 MHz, CDCl3)
N
Boc
12a
f1 (ppm)

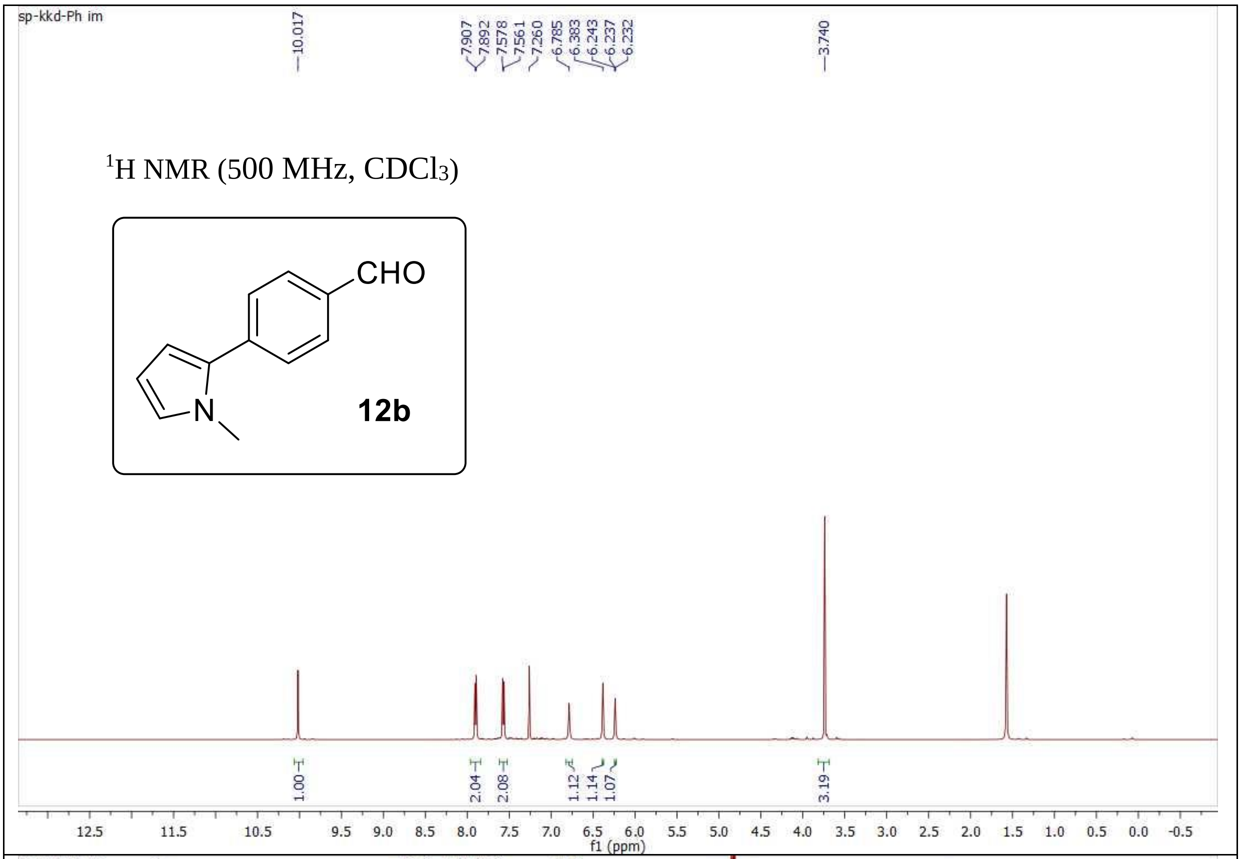
sp-kkd-Ph im
10.017
7.907
7.892
7.578
7.561
7.260
6.785
6.383
6.243
6.237
6.232
3.740
1H NMR (500 MHz, CDCl3)
CHO
N
12b
1.00
2.04
2.08
1.12
1.14
1.07
3.19
f1 (ppm)

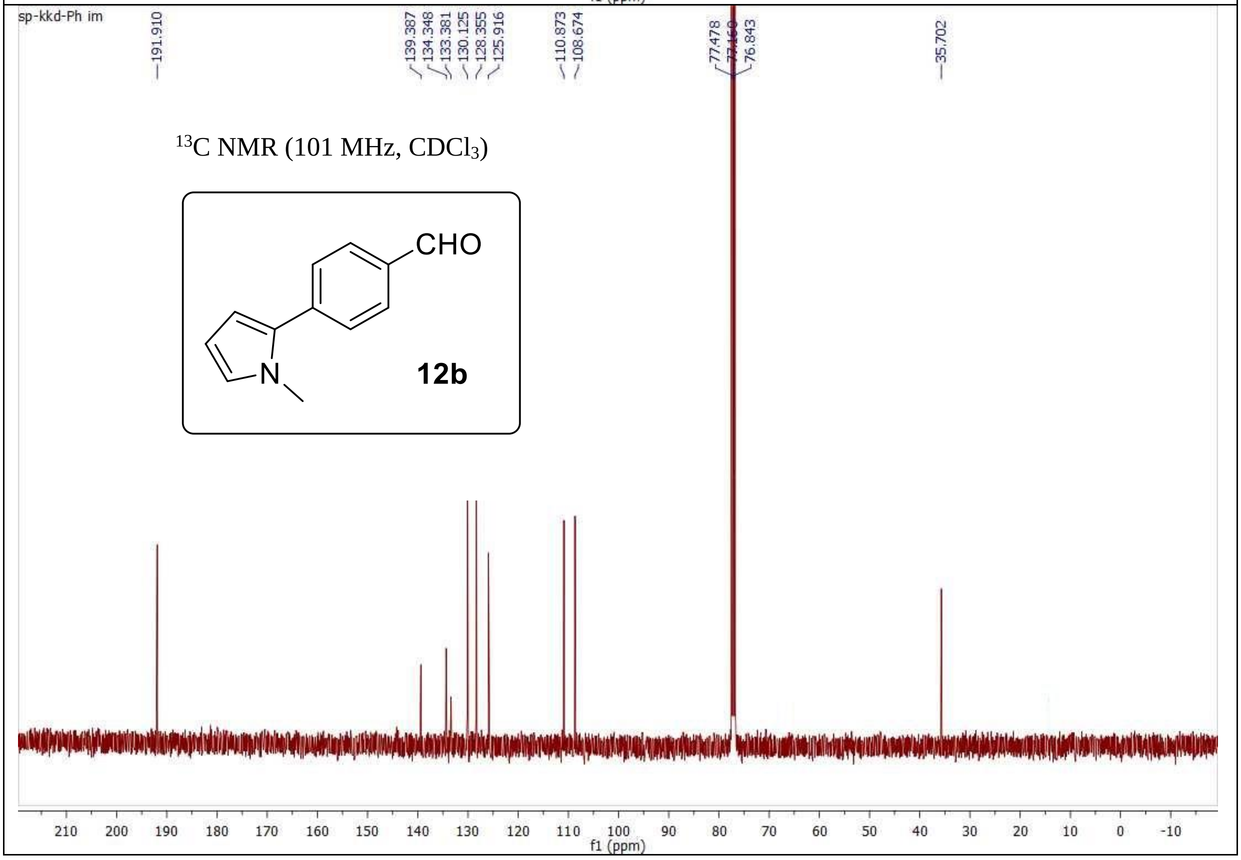
sp-kkd-Ph im
191.910
139.387
134.348
133.381
130.125
128.355
125.916
110.873
108.674
77.478
76.843
35.702
13C NMR (101 MHz, CDCl3)
CHO
N
12b
f1 (ppm)

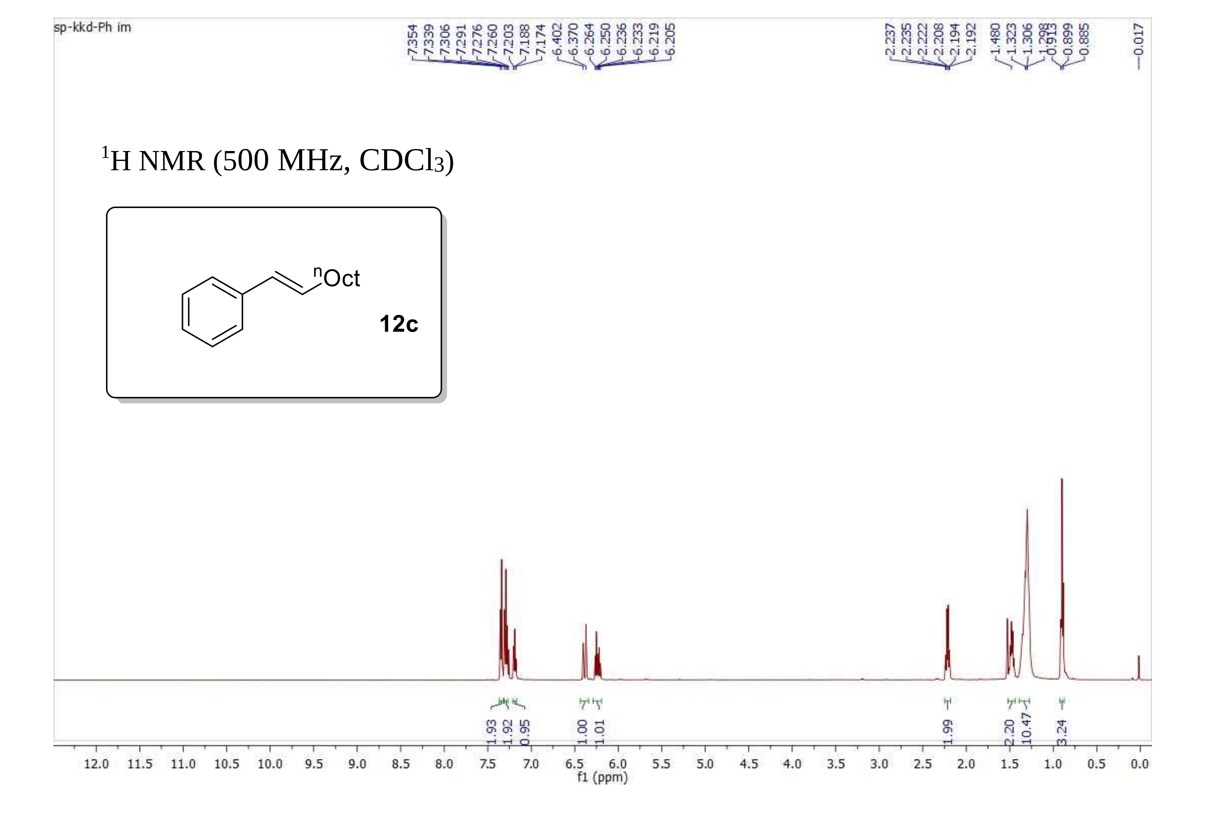
sp-kkd-Ph im
7.354
7.339
7.306
7.291
7.276
7.260
7.203
7.188
7.174
6.402
6.370
6.264
6.250
6.236
6.233
6.219
6.205
2.237
2.235
2.222
2.208
2.194
2.192
1.480
1.323
1.306
0.899
0.885
0.017
^{1}H NMR (500 MHz, CDCl$_3$)
^{n}Oct
12c
1.93
1.92
0.95
1.00
1.01
1.99
2.20
10.47
3.24
f1 (ppm)

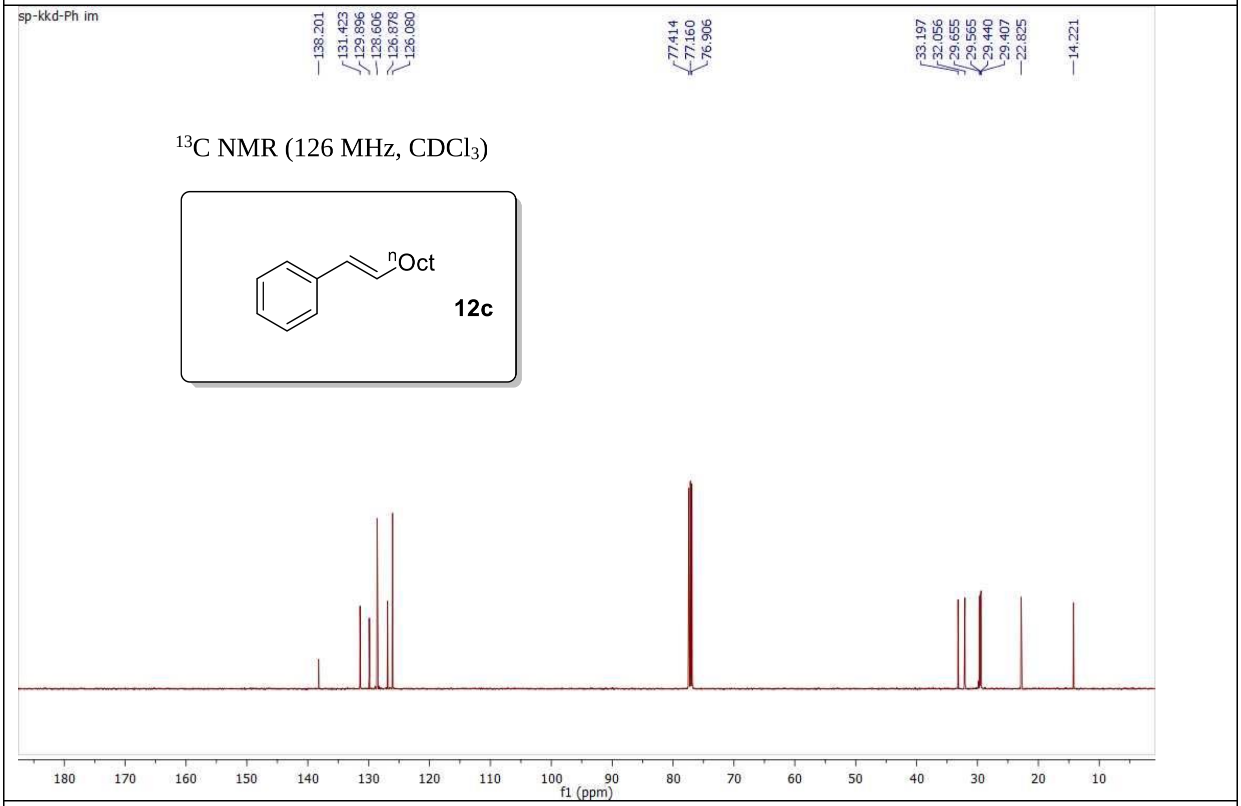
sp-kkd-Ph im
138.201
131.423
129.896
128.606
126.878
126.080
77.414
77.160
76.906
33.197
32.056
29.655
29.565
29.440
29.407
22.825
14.221
^{13}C NMR (126 MHz, CDCl$_3$)
^{n}Oct
12c
f1 (ppm)

Supplementary References:

Ref-1: Compound 2a to 2f: Milas, N. A. The Hydroxylation of Unsaturated Substances. III. The Use of Vanadium Pentoxide and Chromium Trioxide as Catalysts of Hydroxylation. *J. Am. Chem. Soc.* **59**, 2342–2344 (1937).

Ref-2: Compound 2g to 2r: Kumar, B. S. & Negi, A. S. A frank synthesis of alkyl–aryl ethers from 2-halobenzaldehydes and aromatic olefins without transition metal co-catalyst and ligand. *Tetrahedron Lett.* **56**, 2340–2344 (2015).

Ref-3: Compound 2m: Downie, I. M., Earle, M. J., Heaney, H. & Shuhaibar, K. F. Vilsmeier formylation and glyoxylation reactions of nucleophilic aromatic compounds using pyrophosphoryl chloride. *Tetrahedron* **49**, 4015-4034 (1993).

Ref-4: Compound 2n, 2o: Mondal, S., Midya, S. P, Das, S., Mondal, S., Islam, A. S. M. & Ghosh, P. Pd-Catalyzed Tandem Pathway for Stereoselective Synthesis of (*E*)-1,3-Enyne from β-Nitroalkenes by Using a Sacrificial Directing Group. *Chem. Eur. J.* **20**, 3162-3168 (2014).

Ref-5: Compound 3a to 3l; 4a to 4h; Compound 5a: 5b: 5c: 5f: 5h: 5j: 5k: Long, L. M. & Henze, H. R. Synthesis of Ketone Derivatives of Biphenyl by the Friedel-Crafts Reaction *J. Am. Chem. Soc.* **63**, 1939–1940 (1941).

Ref-6: Compound 6a: Compound 6c, 6e: Chavan, S. P., Garai, S., Dutta, A. K. & Pal, S. Friedel–Crafts Acylation Reactions Using Esters. *Eur. J. Org. Chem.* **2012**, 6841–6845 (2012).

Ref-7: Compound 7h; 7i; 7k; 7l to 7o: Adams, R., Harfenist, M. & Loewe, S. New Analogs of Tetrahydrocannabinol. *J. Am. Chem. Soc.* **71**, 1624–1628 (1949).

Ref-8: Compound 8b, 8d, 8f: Campo, et al Aryl to Aryl Palladium Migration in the Heck and Suzuki Coupling of o-Halobiaryls. *J. Am. Chem. Soc.* **129**, 6298–6307 (2007).

Ref-9: Compound 10c and 10d: 29. Gashghaee, M., *et al.* Synthesis, molecular dynamic, and in silico study of new ethyl 4-arylpyrimido [1, 2- b] indazole-2-carboxylate: Potential inhibitors of α -glucosidase. *J. Mol. Struct.* **1257**, 132507 (2022).

Ref-10: Compound 10e: Compound 11b, 11c, 12b, 12c: Pezzetta, C., Folli, A., Matuszewska, O., Murphy, D., Davidson, R. W. M. & Bonifazi, D. peri-Xanthenoxanthene (PXX): A Versatile Organic Photocatalyst in Organic Synthesis. *Adv. Synth. Catal.* **363**, 4740-4753 (2021).

Ref-11: Ameh, E.S. A review of basic crystallography and x-ray diffraction applications. *Int. J. Adv. Manuf. Technol.* **105**, 3289–3302 (2019).

Ref-12: Farrugia, L. J. WinGx suite for small-molecule single crystal crystallography. *J. Appl. Crystallogr*. **32**, 837 (1999).

Ref-13: Bunaciu, A. A., Udrіştioiu, E. G. & Aboul-Enein, H.Y. X-Ray Diffraction: Instrumentation and Applications. *Crit. Rev. Anal. Chem.* **45**, 289–299 (2015).

Ref-14: Ali, A., Chiang, Y. W. & Santos, R. M. X-ray Diffraction Techniques for Mineral Characterization: A Review for Engineers of the Fundamentals, Applications, and Research Directions. *Minerals* **12**, 205 (2022).

Ref-15: Shee, M., Zhang, D., Banerjee, M., Roy, S., Pal, B., Anoop, A., Yuan, Y. & Singh, N. D. P. Interrogating bioinspired ESIPT/PCET-based Ir(iii)-complexes as organelle-targeted

phototherapeutics: a redox-catalysis under hypoxia to evoke synergistic ferroptosis/apoptosis *Chem. Sci*. **14**, 9872-9884 (2023).

Ref-16: Mondal, S., Ballav, T., Mohammad, T. S. & Ganesh, V. Bis-benzofulvenes: Synthesis and Studies on Their Optoelectronic Properties. *Org. Lett.* **25**, 3941-3945 (2023).

Ref-17: Romero, N. A. & Nicewicz, D. A. Organic Photoredox Catalysis. *Chem. Rev*. **116**, 10075-10166 (2016).

Ref-18: Frisch, M. E. et al. Gaussian 16 (Gaussian, Inc., 2016).

Ref-19: Becke, A. D. Density-functional thermochemistry. III. The role of exact exchange. *J. Chem. Phys*. **98**, 5648–5652 (1993).

Ref-20: Lee, C., Yang, W. & Parr, R. G. Development of the Colle-Salvetti correlation-energy formula into a functional of the electron density. *Phys. Rev*. **B37**, 785-789 (1988).

Ref-21: Yadav, N., Taneja, N., Musib, D. & Hazra, C. K. Practical Access to meta-Substituted Anilines by Amination of Quinone Imine Ketals Derived from Anisidines: Efficient Synthesis of Anti-Psychotic Drugs. *Angew. Chem. Int. Ed*. **62**, e202301166 (2023).

Ref-22: Berezin, A., Biot, N., Battisti, T. & Bonifazi, D. Oxygen-Doped Zig-Zag Molecular Ribbons. *Angew. Chem. Int. Ed*. **57**, 8942-8946 (2018).
